# Structure Based Annotation of *Helicobacter pylori* Strain 26695 Proteome

**DOI:** 10.1371/journal.pone.0115020

**Published:** 2014-12-30

**Authors:** Swati Singh, Praveen Kumar Guttula, Lalitha Guruprasad

**Affiliations:** School of Chemistry, University of Hyderabad, Hyderabad, 500046, India; Indian Institute of Science, India

## Abstract

The availability of complete genome sequences of *H. pylori* 26695 has provided a wealth of information enabling us to carry out *in silico* studies to identify new molecular targets for pharmaceutical treatment. In order to construe the structural and functional information of complete proteome, use of computational methods are more relevant since these methods are reliable and provide a solution to the time consuming and expensive experimental methods. Out of 1590 predicted protein coding genes in *H. pylori*, experimentally determined structures are available for only 145 proteins in the PDB. In the absence of experimental structures, computational studies on the three dimensional (3D) structural organization would help in deciphering the protein fold, structure and active site. Functional annotation of each protein was carried out based on structural fold and binding site based ligand association. Most of these proteins are uncharacterized in this proteome and through our annotation pipeline we were able to annotate most of them. We could assign structural folds to 464 uncharacterized proteins from an initial list of 557 sequences. Of the 1195 known structural folds present in the SCOP database, 411 (34% of all known folds) are observed in the whole *H. pylori* 26695 proteome, with greater inclination for domains belonging to α/β class (36.63%). Top folds include P-loop containing nucleoside triphosphate hydrolases (22.6%), TIM barrel (16.7%), transmembrane helix hairpin (16.05%), alpha-alpha superhelix (11.1%) and S-adenosyl-L-methionine-dependent methyltransferases (10.7%).

## Introduction

*Helicobacter pylori* is a spiral shaped, anaerophilic, gram negative and sluggish growing flagellated human pathogen existing in the gastric mucosa of human stomach. This bacterium was identified in 1982 by Barry Marshall and Robin Warren [1]. Around half of the world’s population has an estimated prevalence of *H. pylori* infection, probably reaching up to 70% in developing countries and relatively less in industrialized countries (20–30%) according to World Gastroenterology Organisation [http://www.worldgastroenterology.org/assets/downloads/en/pdf-/guidelines/11_helicobacter_pylori_developing_countries_en.pdf]. In most of the cases, *H. pylori* infections are lingering but asymptomatic, majority of the patients never experience symptoms or some may have only mild gastric inflammation, acute infection may cause clinically relevant chronic active gastritis, peptic ulceration [2], chronic atrophic gastritis that is a sign of gastric adenocarcinoma and mucosa associated lymphoid tissue lymphomas [3].

*H. pylori* possess significant genotypic diversity which engenders various strategies to interact with host cells, manipulate their behaviors in order to survive and propagate. Disease outcome is greatly influenced by bacterial genotype, host- physiology, genotype, dietary habits [4], [5], host genetic diversity, particularly within immune response genes [6]. Since the undissociated form of weak acids can freely pass through the cell membrane of any microbe, weak acids possess potent antimicrobial activity but *H. pylori* has the ability to tolerate acidic conditions in the gastric environment by creating a positive inner-membrane potential at low pH [7]. In the absence of treatment, infection can persist lifelong and are frequently transmitted from person to person, probably by oral to oral and/or fecal to oral. Most of the disease treatment methods for *H. pylori* infections are centered on the use of a proton-pump inhibitors and antibiotics such as metronidazole and clarithromycin [8].

Genome sequence analysis revealed that the circular genome of *H. pylori* strain 26695 consists of 1,667,867 base pairs with 1,590 predicted coding sequences and has well-built systems for motility, scavenging iron, DNA restriction and modification [9]. Several putative adhesins, lipoproteins and other outer membrane proteins have been identified in the complete proteome as possible partners for host-pathogen interactions in *H. pylori* 26695. The annotation of this genome/proteome would help in identifying better drug targets that can be exploited to tackle *H. pylori* infections, and it is one of the ultimate goals of this sequencing project. Often, functional annotation of a proteome is achieved from comparative sequence analysis. These methods have limitations since they mainly rely on the comparisons based on sequence homology. As a result, all proteomes sequenced so far have ∼40–60% unannotated proteins [10].

Focus on three dimensional (3D) structural organizations of proteins would help in deciphering the protein fold, structure and active site, which have applications in the structure based drug design methods that are rational. The availability of complete genome sequences has provided a platform to decipher the structural and functional information of any complete proteome using the computational methods. The results are reliable and provide a solution to the time consuming and expensive experimental methods. The information about function of a protein resides in its structure; the high resolution 3D structures of proteins are determined using X-ray crystallography and NMR techniques. In the absence of experimental structures, sequence homology methods are employed based on the understanding that proteins which share sequence similarity would also have homologous structure and function, barring a few examples [11], [12]. This formalism has a limitation; the numbers of protein sequences available from complete sequencing projects far outweigh the number of available 3D structures and the functionally characterized proteins experimentally. As a result, alternative procedures such as fold recognition for proteins that share low sequence homology are compared to similar 3D structures, and *ab-initio* modelling methods can also be employed. From the validated 3D structures, types of folds and the active site can be characterized. The 3D structures of 145 proteins in *H. pylori* are so far determined experimentally and deposited in protein structure databank (PDB) [13], therefore a wealth of structural information remains to be explored. In this work, we have employed fore-mentioned computational methods to get structural as well as functional insights into the *H. pylori* proteome.

## Materials and Methods

### Directions for structure annotation

**Figure 1:**
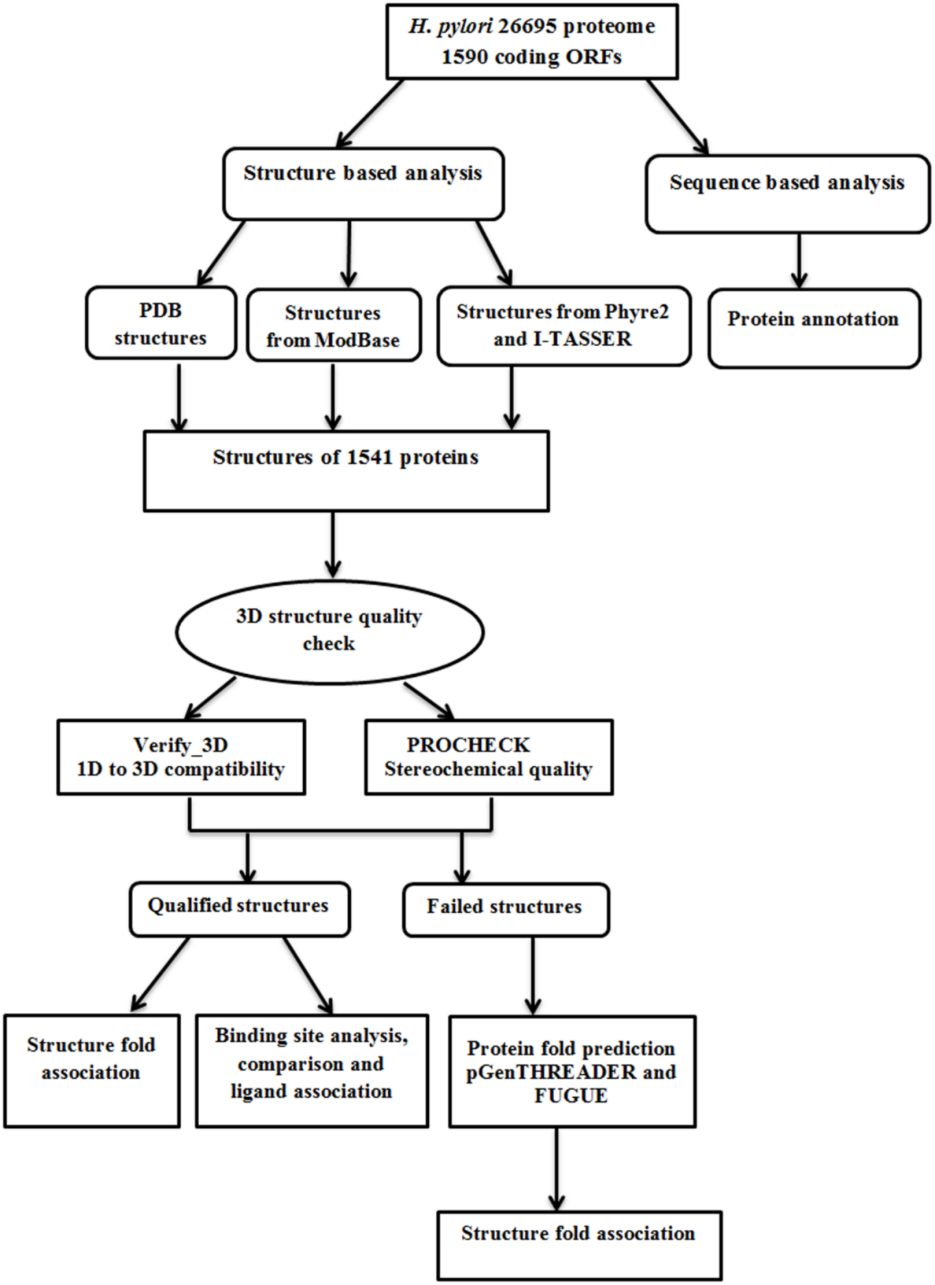
Flow chart for annotating individual proteins in *H. Pylori* 26695 strain.

In order to understand the biological role of large numbers of linear amino acid sequence data generated through genome sequencing projects, we need to have knowledge of their structure. Even though structures determined by experimental methods provide high-resolution data, due to various limitations, structures cannot be determined experimentally for a large proportion of these sequences. Computational structure prediction techniques provide significant and reliable information, and are cost effective as well as less time consuming. Our approach started with finding structural models of the individual Pylorigene database (http://genolist.pasteur.fr/PyloriGene) proteins using different sources in various sequential steps, followed by structure validation. The theoretical models are further subjected to analysis as a way to gain insight into their function. Functional annotation has been assigned through fold to function association as well as by the identification of ligand binding sites and cavities associated with that model. Fold prediction methods attempt to detect structural folds that are compatible with a particular query sequence based on similarities between query protein sequence and proteins of known 3D structure. Since protein surface dictates the type of interaction it can make with its associated ligand or other interacting partners, we further analyzed the protein structures through their binding sites. The overall objective is to predict as accurately as possible the probable function of the protein, at sequence and structure level. At amino acid sequence level we have annotated the protein by gene ontology to decipher the function. At the structure level we assigned structural classification, fold, ligand location (binding site) and ligand type (associated ligand, cofactor, etc.) based on the template structure. The flow chart shown in Fig. 1 depicts various steps adopted for the annotation of *H. pylori* 26695 proteome.

### Protein models

Functional annotation at the sequence level is performed by using AmiGO gene ontology tool [14]. AmiGO is a web application that allows ontologies and related gene product annotation (association) data. Out of 1590 predicted protein coding genes in *H. pylori,* experimentally determined structures are available for 145 proteins in the PDB, proteins with less than 30 amino acid residues were excluded from the study and for rest of the proteins structural models were built using various methods described below.

ModBase [12] (ftp://salilab.org/databases/modbase/projects/tdi/models/) is a database containing comparative protein structure models of various organisms and relies mainly on MODELLER [15] for fold assignment, sequence–structure alignment, model building and model assessment. MODELLER is used for homology or comparative modelling of protein 3D structures by satisfaction of spatial restraints. The main criteria used to judge the quality of the protein model from ModBase was sequence identity and query length coverage (ranking score (Z)  =  product of % sequence identity and % length of query sequence in the alignment), DOPE score (Discrete Optimized Protein Energy <0 is reliable) and MPQS (ModPipe Quality Score of >1.1 is considered to be reliable) [16], [17]. Proteins for which good models were not available in ModBase were submitted to Phyre2 [18] which is based on the principles of homology modelling, and followed by I-TASSER [19] which is a combination of *ab initio* folding and threading methods. Information about all the structures with their respective source are provided in S1 Table. For some protein sequences, the models built were derived from templates without significant sequence similarities, but we were able to accept them based on high compatibility with the structural folds.

### Quality estimation of protein structures

Quality of all the protein models obtained through ModBase, Phyre2 and I-TASSER were analyzed using Verify-3D [20] and PROCHECK [21]. Verify-3D checks the compatibility of the model with its own amino acid sequence and PROCHECK validates stereochemical parameters of the protein models by analyzing residue by residue geometry and overall structural geometry. The S1 Table also provides information about the quality estimation values of all the models which we have obtained from different sources. All the qualified structures can be downloaded from https://sites.google.com/site/lgpscuh/links.

Further the proteins where good quality structures could not be built either by homology modelling or *ab initio* approaches, were further analyzed by PSIPRED which is an accurate secondary structure prediction method that incorporates two feed-forward neural networks and performs an analysis on the output obtained from PSI-BLAST. Here we have used pGenTHREADER method [22] for fold recognition and identification of distant homologues which makes use of profile-profile alignments and predicted secondary structure (using PSIPRED) [23] as inputs. The structures whose confidence were certain or high are selected for annotation and are listed in S2 Table, while rest of the proteins were further subjected to FUGUE [24]. FUGUE is a method for recognizing distant homologues by sequence-structure comparison. It utilizes environment specific substitution tables and structure-dependent gap penalties, where scores for amino acid matching and insertions/deletions are evaluated depending on the local environment of each amino acid residue in a known structure. Given a query sequence (or a sequence alignment), FUGUE searches a database of structural profiles and calculates the sequence-structure compatibility scores and provides a list of potential homologues and their sequence alignment. All the sequences with high confidence in prediction were selected for annotation and are listed in S3 Table. The structures which have high confidence were selected for functional annotation of proteins and were submitted to Dali [25] server which performs structural alignment and carries out comparative analyses of newly discovered protein structures with known PDB structures. The output generated gives the list of structural neighbours and their corresponding structural alignment. From the results, the hit with highest z-score, percentage identity and lowest root mean square deviation (RMSD) were selected for annotating the protein structure. All these validated structures are shown in S4 Table. While the annotations and a broad functional category were available in the databases for many proteins based on literature and sequence analyses, several new associations have been possible through modelling and structural analyses. Of the 557 conserved proteins annotated as “hypothetical” in the Pylorigene database, 464 proteins are now associated with fold-based function annotation through the pipeline described above.

### Assigning associated fold to each structure

The PDB structures and the validated protein model structures of good quality obtained through ModBase, Phyre2, and I-TASSER along with the associated target structures identified by PSIPRED and FUGUE were submitted to 3D-BLAST [26] for SCOP classification [27] which searches for the longest common substructure called SAHSPs (structural alphabet high-scoring segment pair). This is a fast and accurate method for discovering homologous proteins and evolutionary classifications of newly determined structures. It gives a list of homologous protein structures that are similar to the query, ordered by E-values. SCOP database has manual classification of protein structural domains based on similarities of their structures and amino acid sequences. The classification of protein structures in the database is constructed on evolutionary relationships and on the principles that govern their 3D structure. These SCOP-IDs were further submitted to SCOP database in order to retrieve the class, fold, superfamily and family associated with each structure as shown in S5 Table.

### Binding site identification and comparison

Ligand binding is a key aspect of protein function, mediating the ability of proteins to recognize their natural ligands for transport, signal transduction, catalysis and etc. This information also aids in the modulation of their function through the discovery of inhibitors. In COFACTOR method [28], potential ligand binding sites in various models were identified through a consensus ranking based on C-score, RMSD, identity, TM score and coverage. It analyses conserved surface residues and predicts the functional site located to be around a point without giving any boundary definition to pocket. All the pockets predicted by COFACTOR within 2 Å zone of RMSD, and sequence identity greater than 30% were selected and the associated binding sites were compared to known sites for each structure. CASTp [29] server was also used to predict the cavities if the structure fails to have ligand binding site within 2 Å. CASTp gives information regarding the cavity or pocket, domain name and also about the residues present in the cavities.

## Results and Discussion

**Figure 2:**
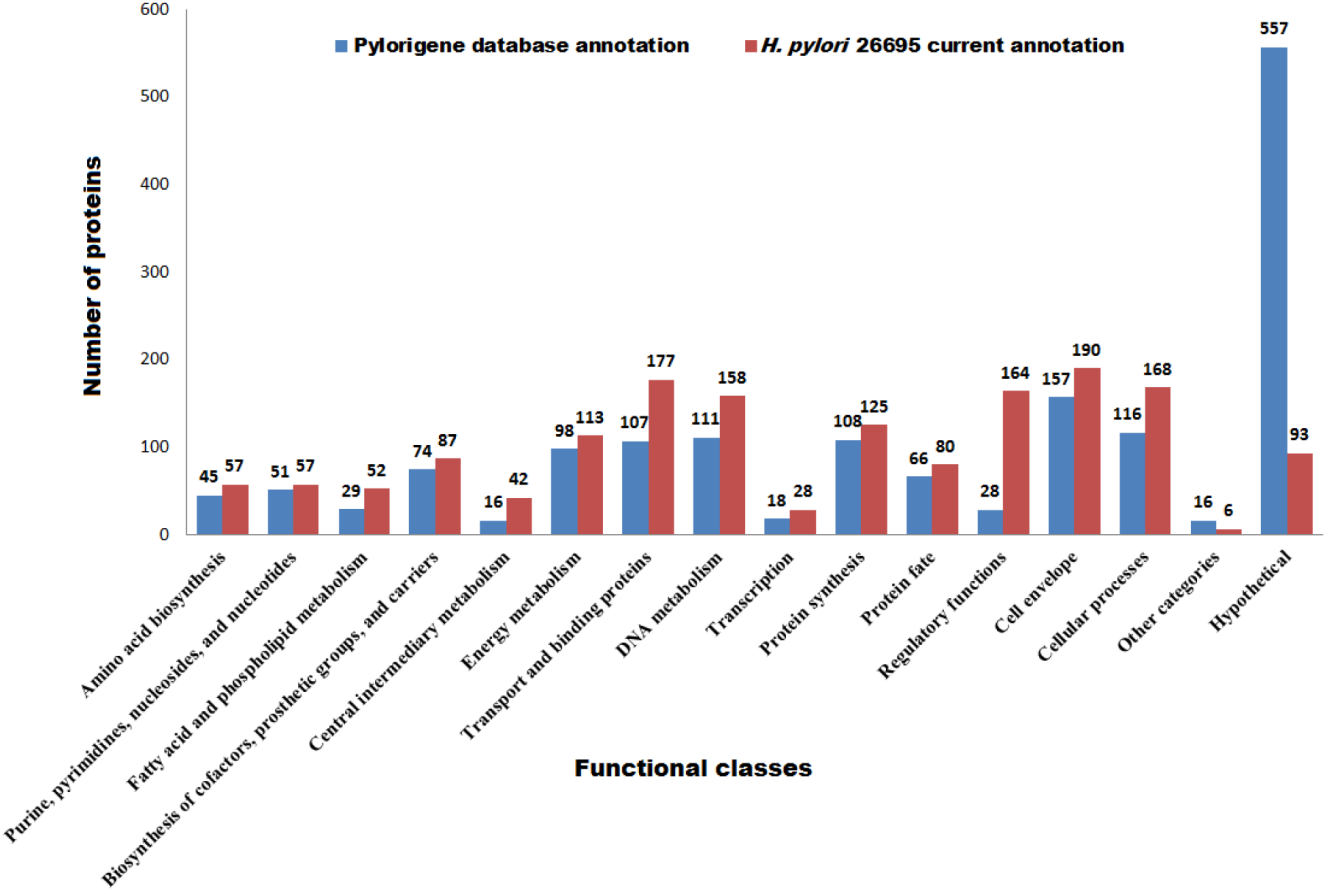
Comparison of *H. pylori* 26695 proteome functional classes in Pylorigene database and current annotation studies.

Pylorigene database, an annotated *H. pylori* proteome consists of large number of weakly annotated proteins termed as ‘predicted’ since they are largely derived through sequence comparisons. The database also has list of proteins which have not been studied in *H. pylori* but homologs have been characterized in other organism or gene has not been studied in any other organisms and hence are annotated as ‘predicted coding region’ and ‘predicted coding regions with no homologs in database’ respectively. Most of these proteins are uncharacterized in Pylorigene database, but through our annotation pipeline we were able to annotate most of them. Previously uncharacterized proteins (557) were listed in the database, and through this methodology adopted by us, we could annotate 464 proteins as shown in Fig. 2. We further compared previous annotation from Pylorigene database and new annotation from our work (S6 Table). Both annotations are agreeable in most of the cases and new annotation has been added to several proteins in *H. pylori* database. Recently, Resende et. al., 2013 [30] have also annotated the proteins of *H. pylori* 26695 using merlin software tools and on-line databases. EC numbers and TC numbers to metabolic gene encoding enzymes and transport proteins, respectively have been assigned. In spite of differences in the methodologies, we found that our functional annotation of nearly 95% proteome is agreeable with that of Resende et al., 2013.

### Fold distribution in the proteome

**Figure 3:**
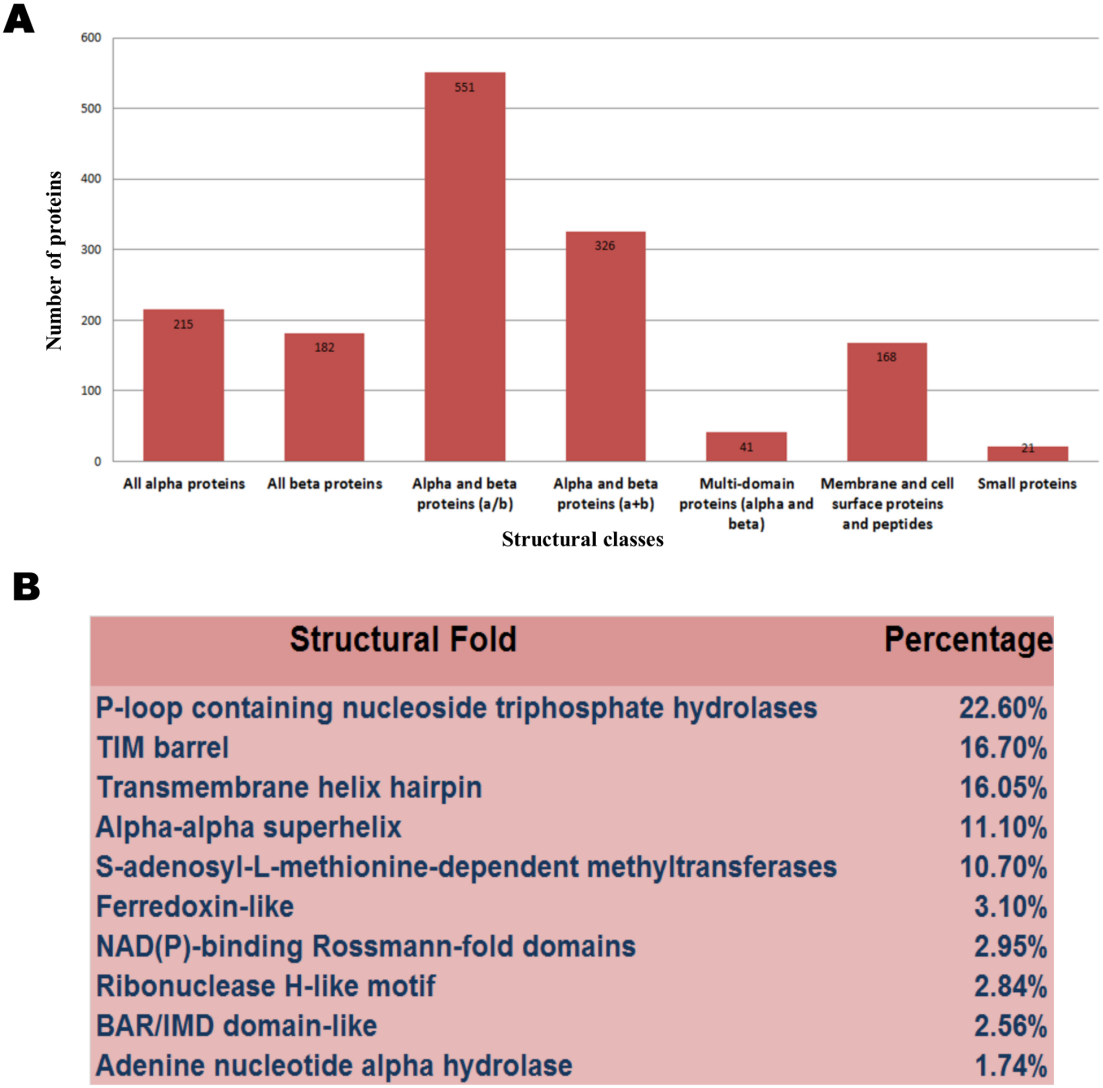
Coverage of *H. pylori* 26695 structural proteome (A) Distribution of structural classes in *H. pylori* 26695 proteome according to SCOP classification. (B) Distribution of major structural folds across *H.pylori* 26695 proteome.

The availability of the structural models covering *H. pylori* complete proteome can be useful in analyzing the fold content and fold preferences of this organism which will be further helpful in identifying important folds that are sufficient for sustaining life and causing infection to the host. Also, the knowledge of structural fold of a protein important in causing disease could be a good target for structure based drug design studies. Assignment of structural folds in the whole proteome is performed through the SCOP database. The fold analysis carried out in this proteome evidently shows the existence of all seven major structural classes (as per SCOP classification) as indicated in Fig. 3A and 3B.

Of the 1195 known structural folds present in the SCOP database, 411 (34% of all known folds) are observed in the whole *H. pylori* proteome, with greater inclination for domains belonging to α/β class (36.63%). The top folds (Fig. 3A) in the modelled *H. pylori* proteome included P-loop containing nucleoside triphosphate hydrolases, TIM barrel, transmembrane helix hairpin, alpha-alpha superhelix, S-adenosyl-L-methionine-dependent methyltransferases and ferredoxin-like, altogether covering almost 75% of all the folds present in this proteome. Predominant folds occurring in various organisms have been studied in their respective proteomes giving rise to a powerlaw distribution of folds [31], which is found to be consistent with the pattern seen here in the *H. pylori* proteome.

### Structure based assessment and functional annotation of *H. pylori* 26695 proteome

A significant feature of this annotation pipeline involved obtaining structures from databases of experimental and *in silico* results. In the absence of such data, 3D structures were either built or at least the fold was predicted. The assessment of each model was made through different estimates of confidence, for example, statistical significance of alignments, extent of sequence similarity, geometry and stereochemistry when compared to high resolution crystal structures, and primary sequence to 3D structure correlation. We therefore believe that the results from these methods are highly reliable and reproducible.

**Figure 4:**
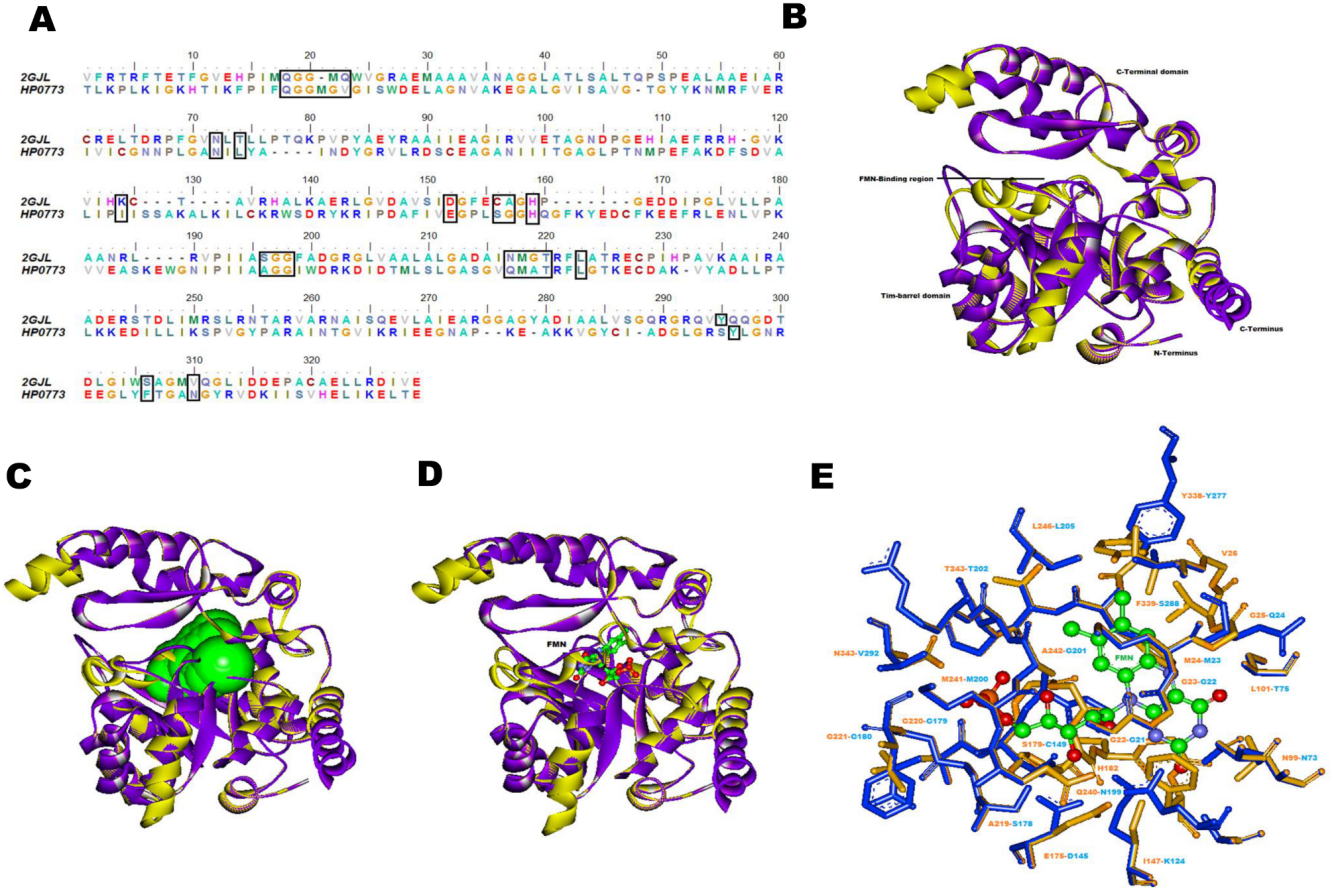
Modelling and structural analysis of HP0773. (A) Sequence alignment of HP0773 and its structural template PDB_ID: 2GJL_A used for modelling. Ligand binding residues are shown in black boxes. (B) Superimposition of HP0773 model (yellow) with its template structure PDB_ID: 2GJL_A (purple). (C) In the HP0773 model, CastP predicted cofactor binding pocket is shown in green surface. (D) Superimposition of the predicted ligand binding site of model with the template structure. (E) Association of the FMN cofactor to the predicted binding site of model (residues in yellow) based on high similarity to a known FMN binding site of template 2GJL (residues in blue). Cofactor FMN is represented in ball and stick model, carbon- green, oxygen- red, nitrogen- blue and phosphorus- orange.

First example we discuss in this category is HP0773 (UniProt ID: O25465, 363 amino acids) that is labeled as predicted coding region HP0773 in Pylorigene and UniProt databases. Our analyses described the protein as nitroalkane dioxygenase that is also known as nitroalkane oxidase (NAO). NAO is a structural member of the flavoenzyme acyl-CoA dehydrogenase (ACAD) superfamily. These enzymes are mainly involved in catalyzing oxidative denitrification of neutral nitroalkanes to their equivalent carbonyl compounds like aldehydes or ketones, hydrogen peroxide and nitrite using FAD or FMN as a cofactor. These nitroalkanes are further used as intermediates for synthesis in chemical industries [32], [33]. Several antibiotics, such as chloramphenicol and azomycin, contain nitro groups, and are also produced by many leguminous plants in the form of nitro toxins such as 3-nitro-1-propionic acid and 3-nitro-1-propanol [34]. HP0773 was modelled on the template PDB_ID: 2GJL which is the crystal structure of 2-nitropropane dioxygenase by the Phyre2 methodology. Both model and template structures superimposed well with low RMSD (0.08 Å) (Fig. 4A and 4B). The stereochemical geometry (0.4% residues are present in disallowed regions of Ramachandran plot) and sequence to structure correlation of the model using Verify-3D (88.13% of the residues had an averaged 3D–1D score >0.2) were confirmed and then subjected to further structural analysis.

NAO comprises of two domains, a triose phosphate isomerase (TIM) barrel domain and C- terminal domain with a novel folding pattern (αααβαβα fold). The TIM barrel domain has eight parallel β strands and eight α-helices. Between these two domains a cleft is located where flavin mononucleotide (FMN) is bound as a cofactor. In the deep binding pocket situated near the boundary of two domains, one molecule of non-covalently bound FMN is present and majority of the binding site residues are contributed by the main (β/α)_8_ barrel domain. The COFACTOR server [28], predicted ligand binding site in the model to be similar to PDB_ID: 2GJL_A that is co-crystallized with cofactor FMN. The CASTp predictions made on the modelled protein also identified pocket that overlaps with the pocket of the template structure. The location of overlapping binding pockets are shown in Fig. 4C and 4D. The phosphate moiety of FMN is buried completely inside the pocket and is solvent inaccessible. This phosphate moiety in the modelled structure makes contacts with the backbone amide atoms of Gly180, Gly221, Ala242 and Thr243 (last three residues Gly221, Ala242, and Thr243 constitute the standard phosphate binding motif characteristic of FMN-dependent oxidoreductase and phosphate-binding enzymes family of (β/α)_8_-barrel proteins).

The edge of isoalloxazine ring of FMN is somewhat accessible from the protein surface. The FMN binding pocket within 5 Å region is lined by amino acid residues, model (template), Gly22 (Gly21), Gly23 (Gly22), Met24 (Met23), Gly25 (Gln24), Val26, Asn99 (Asn73), Leu101 (Thr75), Ile147 (Lys124), Glu175 (Asp145), Ser179 (Cys149), Gly180 (Ala150), Gly181, His182 (His152), Ala219 (Ser178), Gly220 (Gly179), Gly221 (Gly180), Gln240 (Asn199), Met241 (Met200), Ala242 (Gly201), Thr243 (Thr202), Leu246 (Leu205), Tyr338 (Tyr277), Phe339 (Ser288) and Asn343 (Val292) as shown in Fig. 4E. A loop region comprising Phe339 (Ser288) covers the dimethylbenzene part of the isoalloxazine ring and contributes to the formation of binding site for the substrate, 2-nitropropane. Residues involved in interactions with FMN include Gly23, Val26, Gly181, His182, Gly221 and Thr243. Hydrogen bonds that mediate interactions between FMN and enzyme are Gly23:OH…FMN:O2 (2.17 Å), Val26:NH…FMN:N5 (2.33 Å), Gly:181:NH…FMN:O4′ (2.45 Å), Gly:181:NH…FMN:O2P (2.26 Å), His182:HD1…FMN:O5′ (1.68 Å), Gly221:NH…FMN:O3P (1.79 Å), Ala242:NH…FMN:O1P (1.91 Å), Thr243:NH…FMN:O2P (1.94 Å) and Asn343:NH2…FMN:O3P (2.67 Å).

Further oxidation of the neutral nitroalkanes by NAO needs a catalytic base to initiate oxidation of the neutral substrates by abstracting a proton from the substrate α-carbon [35]. There are many studies in which histidine abstracts a proton from the α-carbon thus exhibiting catalytic base like function and further helps in initiating the oxidation of neutral substrates. In our model structure of HP0773, we propose that His182 (His152) located close to the isoalloxazine ring of FMN in the active site may be involved in enzyme catalysis.

**Figure 5:**
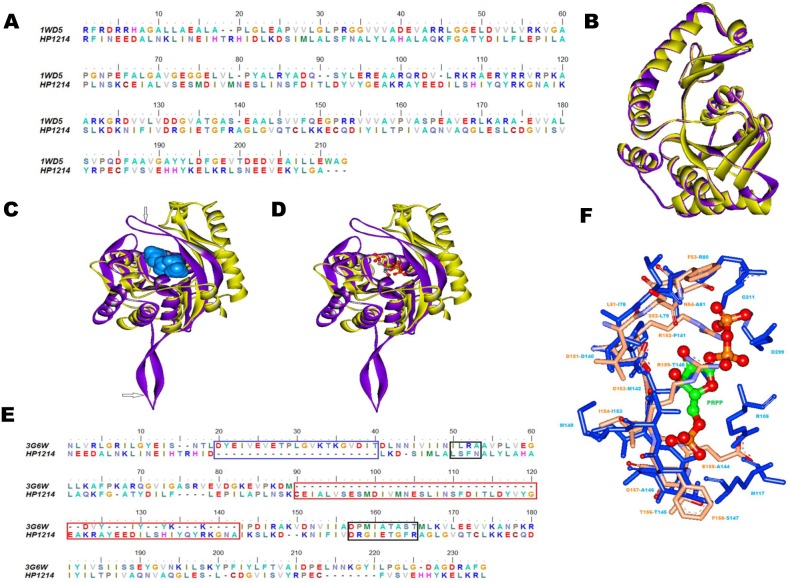
Modelling and structural analysis of HP1214. (A) Sequence alignment of HP1214 and its structural template PDB_ID: 1WD5_A. (B) Superimposition of HP1214 model (yellow) with its template structure PDB_ID: 1WD5_A (purple). (C) In the model structure HP1214, CastP predicted cofactor binding pocket is shown in light blue surface (D) Superimposition of the predicted ligand binding site of model with the template structure. (E) Structure based sequence alignment of HP1214 with PDB_ID: 3G6W_D, residues involved in ligand binding are shown by black boxes, residues of the loop region involved in the dimer formation are shown in blue boxes while red boxes indicate residues involved in the formation of inserted loop. (F) Association of the PRPP ligand to the predicted binding site (residues in yellow) based on high similarity to a known PRPP binding site of template which is PDB_ID: 3G6W_D (residues in blue). PRPP is represented in ball and stick model, carbon- green, oxygen- red, nitrogen- blue and phosphorus- orange.

Another example we discuss is HP1214 (UniProt ID: O25813, 240 amino acids) which is an uncharacterized protein in the database and we annotated it as uracil phosphoribosyltransferases (PRTase). This protein was modelled by Phyre2 server using PDB_ID: 1WD5_A as template which corresponds to crystal structure of a predicted phosphoribosyltransferase from *Thermus thermophiles*. Both model and template structures superimposed with low RMSD (0.09 Å) (Fig. 5A and 5B). According to PROCHECK, the stereochemical quality of the model had no residues in the disallowed regions of Ramachandran plot and Verify-3D showed that 91.47% of the residues had an averaged 3D–1D score >0.2, indicating good quality of the model structure. According to SCOP classification, the structural fold of the model structure was found to be PRTase-like.

The Dali homology search of the model showed close similarity with uracil phosphoribosyltransferase from various organisms like *Sulfolobus solfataricus* (PDB_ID: 3G6W), *Aquifex aeolicus* (PDB_ID: 2E55) and *Bacillus caldolyticus* (PDB_ID: 1I5E). The COFACTOR server predicted inhibitor bound crystal structure PDB_ID: 3G6W as template which has similar binding site as that of model. Analysis of ligand binding residues showed that these regions overlap appreciably well with the modelled protein as shown in Fig. 5C and 5D. Structure based sequence alignment of HP1214 and 3G6W is shown in Fig. 5E. Further comparison of ligand binding residues in both structures showed that most of the residues are same and occupy equivalent positions as that of template as shown in Fig. 5F.

Purine and pyrimidine nucleotides can be synthesized both by *de novo* pathways, from unrelated compounds as well as by salvage pathways by converting the preformed bases and nucleosides to nucleotides. PRTases catalyze the reversible transfer of a phosphoribosyl group from 5-phosphoribosyl-α-1-diphosphate (PRPP) to N1 nitrogen of base resulting in the formation of β-N-riboside monophosphate. The product β-N-riboside monophosphate formed is specified by the base present which can be adenine, guanine, hypoxanthine, xanthine and uracil. Further these PRTs are classified into type I and type II based on the presence and absence of a 13 amino acid sequence (PRPP binding motif), respectively [36]. This well conserved pattern has binding site for PRPP and typically comprises four hydrophobic residues followed by two acidic residues, two hydrophobic residues and four small residues such as glycine [37].

The salvage enzyme uracil phosphoribosyltransferase (UPRTase) belonging to the type I family of PRTases, catalyzes the conversion of uracil and PRPP to uridine monophosphate (UMP) and diphosphate (PPi) [38]. In addition to PRPP binding motif, the sequences of different PRTs reveal little similarity, although a common fold had been predicted for this group of enzymes [39]. Type I PRTs are characterized by the presence of common structural core domain, comprising of four or five parallel β-strands enclosed by at least three α-helices with a subdomain called as hood which includes residues critical for pyrimidine binding. Apart from these conserved core regions, presence of two long loops protruding from the core of protein are also a distinctive feature of all Type I PRTases. Out of the two loops, first one is the β-arm near the N-terminus, which is important for the formation of a stable dimer by embracing a neighboring subunit while the other flexible loop is present close to active site. Like typical type I PRTases, our modelled structure of HP1214 has core region formed by parallel β-sheet (β2, β1, β6, β7, and β8) surrounded by α-helices (α1, α2, α5, and α6), containing the conserved PRPP binding motif (residues 147–159) located in the β6–α5 loop. Since our homology modelling is based on a monomer template, the first long loop which is involved in the formation of a stable dimer was absent, while the other flexible loop (β3–β4) close to the active site was present with additional 43 residue insertion (β5, α3, and α4), which is not present in the other type I PRTases. The flexible loop present above the active site may be involved in closing the active site during catalysis to protect the intermediate/transition state from hydrolysis. In the inserted region, the β5 strand forms an antiparallel sheet (β3, β4 and β5), and the α3-helix interacts with the α6-helix. At the C-terminus, we observed the subdomain region (α7 and α8 helices) that is similar to the hood in the type I PRTases but this region has no significant sequence similarity to those of the other type I PRTases, and these hood structures are completely different suggesting that they may be involved in binding of an unknown substrate [40]. The active site cleft is situated between the hood and the core harboring the PRPP binding sequence.

The COFACTOR server predicted that PRPP binding pocket is enclosed by model (template) Leu51 (Ile78), Ser52 (Leu79), Phe53 (Arg80), Asn54 (Ala81), Asp151 (Asp140), Arg152 (Pro141), Gly153 (Met142), Ile154 (Ile143), Glu155 (Ala144), Thr156 (Thr145), Gly157 (Ala146), Phe158 (Ser147) and Arg159 (Thr148) as shown in Fig. 5E. In the template structure we found that residues Arg80, Asp140, and Thr148 are mostly involved in hydrogen bonding. In HP1214 model structure, side chains of Asp151 and Arg159 are very close to PRPP as compared to template and are involved in hydrogen bond formation with the PRPP. These interactions include Asp151:OH…PRPP:O2 (2.03 Å), Asp151:OH…PRPP:O3 (2.50 Å), Arg159:NH…PRPP:O1P (1.76 Å), Arg159:NH1…PRPP:O3 (1.2 Å), Arg159:NH2…PRPP:O1 (1.8 Å), Arg159:NH1…PRPP:O2 (2.2 Å), Arg159:NH2…PRPP:O3 (2.4 Å), Arg159: NH2…PRPP:O1A (1.01 Å), Arg159:NH2…PRPP:O1 (2.03 Å), Arg159:NH2…PRPP:O1A (0.86 Å), Arg159:NH2…PRPP:O2A (2.08 Å) and Arg159:NH2…PRPP:O3A (1.67 Å). Other hydrogen bonding interactions include Phe53:NH…PRPP:O3B (2.03 Å) and Gly157:NH…PRPP:O2P (1.76 Å) and Thr156:NH…O3P (2.45 Å).

**Figure 6:**
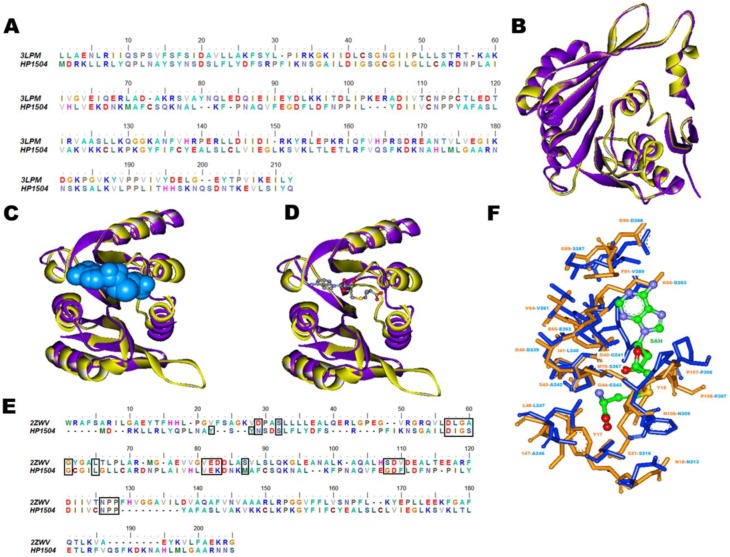
Modelling and structural analysis of HP1504. (A) Sequence alignment of HP1504 and its template PDB_ID: 3LPM_A used for modelling. (B) Structural superimposition of HP1504 model (yellow) with its structural template PDB_ID: 3LPM_A (purple). (C) In the model structure HP1504, CastP predicted ligand binding pocket is shown in blue surface. (D) Superimposition of the predicted ligand binding site of model with the template structure. (E) Structure based sequence alignment of HP1504 sequence with PDB_ID: 2ZWV_A, residues involved in ligand binding are shown in black boxes. (F) Association of the SAH ligand to the predicted binding site (residues in yellow) based on high similarity to a known SAH binding site of template which is PDB_ID: 2ZWV_A (residues in blue). SAH is represented in ball and stick model, carbon- green, oxygen- red, nitrogen- blue and phosphorus- orange.

Another example is HP1504 (UniProt ID: O26034, 238 amino acids) which is unknown in the Pylorigene database but we annotated it as methyltransferase. This protein was modelled by Phyre2 using PDB_ID: 3LPM as template which is the crystal structure of putative methyltransferase small domain protein 2 from *Listeria monocytogenes*. Both model and template structures superimpose with low RMSD (0.13 Å) (Fig. 6A and 6B). Modelled protein had 1.6% residues in the disallowed region of the Ramachandran plot and Verify-3D showed that 90.34% of the residues had an averaged 3D–1D score >0.2, indicating good quality of the model constructed. According to SCOP classification, the predicted fold of the model was S-adenosyl-L-methionine dependent methyltransferases.

Methyltransferases form a large group of enzymes that methylate a variety of substrates but can be segregated into several subclasses based on their structural features. The most common class of methyltransferases is class I that contains a Rossman fold for binding S-adenosyl methionine (SAM) while class II methyltransferases have a SET domain. Methylation of proteins is found to have regulatory role in protein activation, protein-protein interactions and protein-DNA interactions. They also play an important role in methylation of ribosomal RNA (rRNA) nucleotides, which further helps in the biogenesis and activity regulation of the ribosome, such as fine-tuning of local rRNA structure, 30S subunit assembly and antibiotic resistance [41], [42].

The Dali homology search of the model structure showed close resemblance with RNA methyltransferase from various organisms such as *Escherichia coli* (PDB_ID: 2B3T), *Escherichia coli* K-12 (PDB_ID: 4DCM), *Pyrococcus horikoshii* (PDB_ID: 1WY7) and *Thermus thermophiles* (PDB_ID: 3CJT).

Ligand binding site prediction made on the model structure using COFACTOR server identified a pocket that overlaps with the inhibitor bound crystal structure of 16S rRNA methyltransferase belonging to class I of methyltransferases (PDB_ID: 2ZWV_A) which is co-crystallized with ligand SAH (S-adenosyl-L-homocysteine). These sites are also detected by the pocket predictions by the CASTp server as shown in Fig. 6C and 6D. Structure based sequence alignment of HP1504 and 2ZWV_A is shown in Fig. 6E. These comparisons further show an extensive overlap with the binding pocket of other methyltransferases. The residues that constituted the SAH binding site were conserved, these residues (within 5 Å radius of SAH) were extracted and compared to known binding sites of ligands in PDB_ID: 2ZWV. The topologically equivalent positions in both proteins, enclosing SAH are model (template) Tyr15, Tyr17, Asn18 (Asn213), Ser21 (Ser216), Asp40 (Asp239), Ile41 (Leu240), Gly42 (Gly241), Ser43 (Ala242), Gly44 (Gly243), Leu48 (Leu247), Val64 (Val261), Glu65 (Glu262), Lys66 (Asp263), Met70 (Ser267), Gly89 (Ser287), Asp90 (Asp288), Phe91 (Val289), Asn106 (Asn305), Pro107 (Pro306) and Pro108 (Pro307). The alignment of the binding site residues in both structures can be visualized in Fig. 6F.

The proteins for which reliable and validated model structures could not be built, were further analyzed with high confidence using fold-based prediction methods (PSIPRED and FUGUE) and useful annotation could be attributed to some unknown proteins. Some of these examples include, HP0013 (tRNA (5-methylaminomethyl-2-thiouridylate)-methyltransferase), HP0031 (USP like protein, universal stress protein), HP0728 (isoleucyl-tRNA lysidine synthetase), HP1211 (alginate lyase) and HP1413 (NADPH-dependent 7-cyano-7-deazaguanine reductase).

Using the protein sequence based annotation, the function of some proteins could be predicted. Some of these examples include; HP0129 (zinc ion binding protein), HP0130 (DNA binding), HP0158 (N-linked glycosylation), HP0980 (metalloendopeptidase activity), HP0971 (transport protein) and HP0935 (N-acetyltransferase protein).

This systematic structural and functional annotation of *H. pylori* 26695 proteome enhances our knowledge about this pathogenic organism and further provides guidance to find new drug targets.

## Conclusions

In recent years, the need to design and develop novel antibacterial agents has become crucial due to the global outbreak of infectious diseases. Now and in the immediate future, the understanding of the functions of all proteins in *H. pylori* 26695 is not feasible since there are several hundreds of proteins which have to be biologically or biochemically characterized. This current work provides useful information for better understanding of *H. pylori* 26695 strain proteome by providing information on the 3D structure of a protein, which is further useful to predict the structure-function association and cellular functions. The structural annotation reported here covers a significant proportion of the *H. pylori* proteome. This annotation has provided insights about the folds for a significant number of proteins and importantly indicate that cellular metabolism in *H. pylori* 26695 can be achieved with only 411 folds and their various combinations. High confidence molecular models can now be obtained for several proteins, which along with the experimental structures available for that species can provide a first glimpse of the structural proteome as well as key residues and motifs present in the functional sites of that protein.

Assigning of structure-function to several unknown proteins that may be probable virulence determinants will allow critical tests of their functions, cellular targets as well as the innate and adaptive immune responses of the host. This will further aid in novel understanding into their mechanisms of early colonization, persistence of this bacterium during long-term carriage, and the mechanisms by which it promotes various gastroduodenal diseases, and are therefore novel drug targets. The large scale annotation pipeline used here to derive biological insights about *H. pylori* can be readily applied for other organisms as well and will fill the ‘blank spots’ in respective proteomes. At a later stage, the new drug targets can be exploited to identify novel inhibitors using computational methods, as recently demonstrated for *H. pylori* DapE [43].

## Supporting Information

S1 Table **Table showing quality estimation values from Verify_3D and PROCHECK for each protein with their respective source.**
(DOC)

S2 Table **Pgenthreader results for the failed protein models showing template PDB_ID, confidence and P-value.**
(DOC)

S3 Table **FUGUE results for the failed protein models mentioning template PDB_ID, confidence and Z-score.**
(DOC)

S4 Table **DALI results for the passed protein models mentioning Z-score, template PDB_ID and sequence identity.**
(DOCX)

S5 Table **Distribution of various SCOP-ID, class, fold, superfamily and family across whole proteome.**
(DOC)

S6 Table **Comparison of previous annotation from Pylorigene database and new annotation from our work.**
(DOC)
