# Supplementary material for: Structure Based Annotation of Helicobacter pylori Strain 26695 Proteome
Source: PLoS One. 2014 Dec 30;9(12):e115020. doi: 10.1371/journal.pone.0115020 (PMC4280198; doi:10.1371/journal.pone.0115020)
Supplement: S1 Table — Table showing quality estimation values from Verify_3D and PROCHECK for each protein with their respective source. (DOC) [file pone.0115020.s001.doc]

| **Supplementary Table I. Source of the structure and models of *H. pylori* 26695 strain proteins** | | | | | | | | |
| --- | --- | --- | --- | --- | --- | --- | --- | --- |
| **GENE NAME** | **PROCHECK** | | | | | **VERIFY_3D** | | **CLASS** |
|  | CORE | ALLOWED | GENEROUSLY | DISALLOWED | TOTAL ALLOWED | SCORE | STATUS |  |
| HP0001 | 85.2 | 13.9 | 0.0 | 0.8 | 99.1 | 90.51 | PASS | MODBASE |
| HP0002 | 95.5 | 4.5 | 0.0 | 0.0 | 100 | 91.03 | PASS | MODBASE |
| HP0003 | 94.1 | 5.1 | 0.4 | 0.4 | 99.2 | 87.31 | PASS | MODBASE |
| HP0004 | 84.4 | 11.1 | 2.0 | 2.5 | 95.5 | 53.60 | FAIL | PHYRE2 |
| HP0005 | 90.9 | 7.7 | 0.5 | 1.0 | 98.6 | 90.31 | PASS | MODBASE |
| HP0006 | 87.1 | 9.2 | 2.8 | 0.8 | 96.3 | 85.92 | PASS | MODBASE |
| HP0007 | SMALL |  |  |  |  |  |  |  |
| HP0008 | 100.0 | 0.0 | 0.0 | 0.0 | 100 | 0.00 | FAIL | PHYRE2 |
| HP0009 | 71.6 | 18.2 | 4.8 | 5.5 | 89.8 | 44.88 | FAIL | PHYRE2 |
| HP0010 | 94.4 | 4.1 | 0.6 | 0.9 | 98.5 | 92.19 | PASS | MODBASE |
| HP0011 | 88.0 | 9.3 | 1.4 | 1.3 | 98.7 | 48.89 | FAIL | PHYRE2 |
| HP0012 | 92.3 | 7.2 | 0.5 | 0.0 | 100 | 93.00 | PASS | PDB |
| HP0013 | 76.11 | 16.5 | 3.9 | 3.5 | 92.61 | 41.88 | FAIL | PHYRE2 |
| HP0014 | 76.4 | 17.1 | 4.5 | 2.0 | 93.5 | 92.39 | PASS | ITASSER |
| HP0015 | 100 | 0 | 0 | 0 | 100 | 0 | FAIL | PHYRE2 |
| HP0016 | 95.0 | 5.0 | 0.0 | 0 | 100 | 0 | FAIL | PHYRE2 |
| HP0017 | 67.7 | 22.4 | 4.4 | 5.5 | 90.1 | 29.44 | FAIL | PHYRE2 |
| HP0018 | 70.3 | 21.7 | 5.4 | 2.6 | 92 | 37.66 | FAIL | PHYRE2 |
| HP0019 | 80.3 | 14.3 | 2.7 | 2.7 | 94.6 | 64.60 | FAIL | PHYRE2 |
| HP0020 | 86.9 | 10.2 | 1.7 | 1.1 | 97.1 | 87.34 | PASS | MODBASE |
| HP0021 | 87.9 | 8.6 | 2.1 | 1.4 | 98.6 | 42.26 | FAIL | PHYRE2 |
| HP0022 | 71.8 | 17.8 | 5.1 | 5.3 | 89.6 | 49.04 | FAIL | PHYRE2 |
| HP0023 | SMALL |  |  |  |  |  |  |  |
| HP0024 | SMALL |  |  |  |  |  |  |  |
| HP0025 | 67.1 | 20.2 | 6.5 | 6.2 | 87.3 | 50.00 | FAIL | PHYRE2 |
| HP0026 | 90.80 | 6.6 | 2.1 | 0.5 | 97.4 | 80.05 | PASS | MODBASE |
| HP0027 | 89.9 | 7.7 | 1.6 | 0.8 | 97.6 | 91.92 | PASS | MODBASE |
| HP0028 | 66.7 | 19.4 | 9.7 | 4.2 | 86.1 | 0.00 | FAIL | PHYRE2 |
| HP0029 | 89.8 | 10.2 | 0.0 | 0.0 | 100 | 92.00 | PASS | PDB |
| HP0030 | 72.6 | 19.8 | 3.9 | 3.7 | 92.4 | 35.86 | FAIL | PHYRE2 |
| HP0031 | 72.9 | 18.6 | 3.9 | 4.7 | 91.5 | 67.15 | WARNING | MODBASE |
| HP0032 | 96.2 | 3.8 | 0.0 | 0.0 | 100 | 83.33 | PASS | MODBASE |
| HP0033 | 88.2 | 9.8 | 1.2 | 0.8 | 98 | 83.45 | PASS | PHYRE2 |
| HP0034 | 89.7 | 10.3 | 0.0 | 0.0 | 100 | 83.33 | PASS | PDB |
| HP0035 | 100.0 | 0.0 | 0.0 | 0.0 | 100 | 66.47 | WARNING | PDB |
| HP0036 | 78.2 | 17.0 | 2.7 | 2.0 | 95.2 | 41.19 | FAIL | PHYRE2 |
| HP0037 | 100.0 | 0.0 | 0.0 | 0.0 | 100 | 0.00 | FAIL | PHYRE2 |
| HP0037.1 | NO ID |  |  |  |  |  |  |  |
| HP0038 | 70.7 | 21.6 | 3.9 | 3.9 | 92.3 | 31.71 | FAIL | PHYRE2 |
| HP0039 | 61.3 | 29.0 | 0 | 9.7 | 90.3 | 35.14 | FAIL | PHYRE2 |
| HP0040 | 73.3 | 18.7 | 4.0 | 4.0 | 92 | 48.21 | FAIL | PHYRE2 |
| HP0041 | 85.7 | 11.1 | 1.6 | 1.6 | 98.4 | 13.89 | FAIL | PHYRE2 |
| HP0042 | 87.2 | 12.4 | 0.4 | 0.0 | 100 | 0.0 | FAIL | PDB |
| HP0043 | 88.1 | 10.9 | 0.9 | 0.2 | 99 | 92.91 | PASS | PDB |
| HP0044 | 89.5 | 8.4 | 0.6 | 1.5 | 97.9 | 85.79 | PASS | MODBASE |
| HP0045 | 94.1 | 5.9 | 0.0 | 0.0 | 100 | 82.08 | PASS | MODBASE |
| HP0046 | 100 | 0 | 0 | 0 | 100 | 0.0 | FAIL | PHYRE2 |
| HP0047 | 90.6 | 8.3 | 0.0 | 1.0 | 98.9 | 83.38 | PASS | MODBASE |
| HP0048 | 66.5 | 20.8 | 6.2 | 6.5 | 87.3 | 63.51 | FAIL | PHYRE2 |
| HP0049 | 90.9 | 8.8 | 0.3 | 0.0 | 99.7 | 98.79 | PASS | PDB |
| HP0050 | 78.8 | 14.4 | 3.4 | 3.4 | 93.2 | 63.95 | FAIL | PHYRE2 |
| HP0051 | 83.4 | 13.1 | 2.5 | 1.0 | 96.5 | 73.16 | WARNING | PHYRE2 |
| HP0052 | 86.4 | 11.4 | 2.3 | 0.0 | 97.8 | 3.92 | FAIL | PHYRE2 |
| HP0053 | 64.8 | 21.8 | 5.3 | 8.1 | 86.6 | 31.90 | FAIL | PHYRE2 |
| HP0054 | 62.8 | 24.0 | 6.4 | 6.9 | 86.8 | 43.33 | FAIL | PHYRE2 |
| HP0055 | 88.2 | 8.4 | 2.3 | 1.1 | 96.6 | 59.76 | FAIL | PHYRE2 |
| HP0056 | 89.8 | 8.4 | 1.4 | 0.4 | 98.2 | 70.55 | WARNING | PHYRE2 |
| HP0057 | 84.6 | 15.4 | 0.0 | 0.0 | 100 | 0.0 | FAIL | PHYRE2 |
| HP0058 |  |  |  |  | NO PTN SEQ |  |  |  |
| HP0059 | 88.9 | 10.7 | 0.0 | 0.4 | 99.6 | 0.00 | FAIL | PHYRE2 |
| HP0060 | 82.7 | 12.6 | 2.6 | 2.1 | 95.3 | 64.86 | FAIL | PHYRE2 |
| HP0061 | 68.0 | 32.0 | 0.0 | 0.0 | 100 | 0.0 | FAIL | PHYRE2 |
| HP0062 | 96.4 | 3.6 | 0.0 | 0.0 | 100 | 86.63 | PASS | PDB |
| HP0063 | 88.0 | 10.7 | 1.3 | 0.0 | 98.7 | 25.93 | FAIL | PHYRE2 |
| HP0064 | 66.4 | 19.2 | 6.4 | 8.0 | 85.6 | 85.71 | PASS | PHYRE2 |
| HP0065 | 87.1 | 11.0 | 1.9 | 0.0 | 100 | 0.0 | FAIL | PHYRE2 |
| HP0066 | 77.0 | 16.0 | 3.5 | 3.5 | 93 | 34.86 | FAIL | PHYRE2 |
| HP0067 | 87.3 | 10.2 | 2.1 | 0.4 | 97.5 | 96.01 | PASS | PDB |
| HP0068 | 90.6 | 7.6 | 1.2 | 0.6 | 98.2 | 75.00 | WARNING | MODBASE |
| HP0069 | 92.6 | 6.9 | 0.5 | 0.0 | 99.5 | 93.14 | PASS | PDB |
| HP0070 | 81.3 | 12.9 | 2.6 | 3.2 | 94.2 | 90.73 | PASS | PDB |
| HP0071 | 88.9 | 8.9 | 1.5 | 0.7 | 99.3 | 75.14 | WARNING | PDB |
| HP0072 | 86.7 | 12.5 | 0.8 | 0.0 | 99.2 | 85.25 | PASS | PDB |
| HP0073 | 88.0 | 9.6 | 1.9 | 0.5 | 97.6 | 87.11 | PASS | PDB |
| HP0074 | 70.9 | 23.6 | 3.6 | 1.8 | 98.2 | 0.0 | FAIL | PHYRE2 |
| HP0075 | 87.8 | 10.9 | 0.5 | 0.8 | 98.7 | 85.17 | PASS | MODBASE |
| HP0076 | 90.4 | 7.2 | 1.2 | 1.2 | 98.8 | 60.92 | FAIL | PHYRE2 |
| HP0077 | 94.7 | 4.3 | 0.9 | 0.0 | 99 | 53.45 | FAIL | PHYRE2 |
| HP0078 | SMALL |  |  |  |  |  |  |  |
| HP0079 | 63.9 | 23.8 | 6.5 | 5.8 | 87.7 | 32.21 | FAIL | PHYRE2 |
| HP0080 | 93.2 | 4.5 | 2.3 | 0.0 | 97.7 | 36.36 | FAIL | PHYRE2 |
| HP0081 | 92.3 | 0.0 | 7.7 | 0.0 | 100 | 0.0 | FAIL | PHYRE2 |
| HP0082 | 73.2 | 16.8 | 5.9 | 4.1 | 90 | 32.64 | FAIL | PHYRE2 |
| HP0083 | 78.2 | 19.1 | 0.0 | 2.7 | 97.3 | 65.63 | WARNING | PHYRE2 |
| HP0084 | 77.9 | 18.0 | 3.3 | 0.8 | 95.9 | 80.43 | PASS | MODBASE |
| HP0085 | 95.2 | 4.8 | 0.0 | 0.0 | 100 | 0.00 | FAIL | PHYRE2 |
| HP0086 | 74.6 | 17.5 | 5.2 | 2.7 | 92.1 | 48.34 | FAIL | PHYRE2 |
| HP0087 | 88.2 | 10.0 | 1.0 | 0.8 | 98.2 | 86.95 | PASS | PHYRE2 |
| HP0088 | 69.7 | 20.7 | 6.6 | 3.0 | 90.4 | 23.81 | FAIL | PHYRE2 |
| HP0089 | 92.2 | 6.8 | 0.8 | 0.3 | 99.7 | 97.35 | PASS | PDB |
| HP0090 | 89.1 | 10.2 | 0.7 | 0.0 | 100 | 100 | PASS | PDB |
| HP0091 | 62.8 | 21.3 | 6.2 | 9.7 | 84.1 | 15.47 | FAIL | PHYRE2 |
| HP0092 | 78.3 | 17.3 | 2.0 | 2.4 | 95.6 | 51.44 | FAIL | PHYRE2 |
| HP0093 | 76.5 | 15.9 | 4.5 | 3.0 | 92.4 | 45.58 | FAIL | PHYRE2 |
| HP0094 | 86.2 | 12.2 | 0.8 | 0.8 | 99.2 | 19.48 | FAIL | PHYRE2 |
| HP0095 | 83.5 | 14.1 | 0.6 | 1.8 | 98.2 | 0.00 | FAIL | PHYRE2 |
| HP0096 | 86.8 | 10.7 | 2.1 | 0.4 | 97.5 | 77.60 | WARNING | MODBASE |
| HP0097 | 76.0 | 18.8 | 4.3 | 1.0 | 94.8 | 12.18 | FAIL | PHYRE2 |
| HP0098 | 87.6 | 9.9 | 0.9 | 1.6 | 97.5 | 72.02 | WARNING | PHYRE2 |
| HP0099 | 78.1 | 12.6 | 6.2 | 2.9 | 90.7 | 18.79 | FAIL | PHYRE2 |
| HP0100 | 78.0 | 15.2 | 5.6 | 1.2 | 93.2 | 17.89 | FAIL | PHYRE2 |
| HP0101 | 71.2 | 21.0 | 5.2 | 2.6 | 92.2 | 24.41 | FAIL | PHYRE2 |
| HP0102 | 81.9 | 14.3 | 2.5 | 1.3 | 96.2 | 61.92 | FAIL | PHYRE2 |
| HP0103 | 95.5 | 4.5 | 0.0 | 0.0 | 93.7 | 92.73 | PASS | PDB |
| HP0104 | 79.3 | 14.9 | 3.5 | 2.3 | 94.2 | 73.37 | WARNING | PHYRE2 |
| HP0105 | 85.8 | 14.2 | 0.0 | 0.0 | 100 | 95.21 | PASS | PDB |
| HP0106 | 90.0 | 9.4 | 0.6 | 0.0 | 100 | 0.0 | FAIL | PDB |
| HP0107 | 91.9 | 8.1 | 0.0 | 0.0 | 100 | 99.02 | PASS | PDB |
| HP0108 | 78.0 | 20.9 | 1.1 | 0.0 | 100 | 76.69 | WARNING | PHYRE2 |
| HP0109 | 95.3 | 3.0 | 1.5 | 0.2 | 98.3 | 84.59 | PASS | MODBASE |
| HP0110 | 83.7 | 15.6 | 0.7 | 0.0 | 100 | 30.77 | FAIL | PHYRE2 |
| HP0111 | 80.9 | 15.0 | 3.3 | 0.8 | 95.9 | 46.82 | FAIL | PHYRE2 |
| HP0112 | 83.6 | 15.0 | 0.5 | 0.9 | 98.6 | 59.05 | FAIL | PHYRE2 |
| HP0113 | 100 | 0.0 | 0.0 | 0.0 | 100 | 0.0 | FAIL | PHYRE2 |
| HP0114 | 79.5 | 14.6 | 3.0 | 3.0 | 94.1 | 17.17 | FAIL | PHYRE2 |
| HP0115 | 86.4 | 9.7 | 2.2 | 1.7 | 96.1 | 40.19 | FAIL | PHYRE2 |
| HP0116 | 67.0 | 20.8 | 5.2 | 7.0 | 87.8 | 66.62 | WARNING | MODBASE |
| HP0117 | 73.6 | 20.6 | 3.2 | 2.5 | 94.2 | 60.84 | FAIL | PHYRE2 |
| HP0118 | 85.1 | 10.0 | 3.5 | 1.4 | 95.1 | 0 | FAIL | PHYRE2 |
| HP0119 | 92.6 | 5.5 | 0.9 | 0.9 | 98.1 | 9.96 | FAIL | PHYRE2 |
| HP0120 | 63.0 | 18.5 | 14.1 | 4.3 | 81.5 | 11.03 | FAIL | PHYRE2 |
| HP0121 | 89.5 | 8.6 | 101 | 0.8 | 98.1 | 81.47 | PASS | MODBASE |
| HP0122 | SMALL |  |  |  |  |  |  | PHYRE2 |
| HP0123 | 92.5 | 6.2 | 0.7 | 0.5 | 98.7 | 84.83 | PASS | MODBASE |
| HP0124 | 67.9 | 25.5 | 3.3 | 3.3 | 93.4 | 74.03 | WARNING | MODBASE |
| HP0125 | 61.4 | 33.3 | 3.5 | 1.8 | 98.2 | 68.75 | WARNING | PHYRE2 |
| HP0126 | 85.5 | 10.9 | 2.7 | 0.9 | 99.1 | 34.48 | FAIL | PHYRE2 |
| HP0127 | 58.2 | 24.9 | 6.3 | 10.5 | 83.1 | 38.33 | FAIL | PHYRE2 |
| HP0128 | 94.1 | 5.9 | 0.0 | 0.0 | 100 | 0.00 | FAIL | PHYRE2 |
| HP0129 | 91.5 | 7.3 | 0.0 | 1.2 | 98.2 | 78.41 | WARNING | PHYRE2 |
| HP0130 | 74.3 | 16.0 | 4.1 | 5.6 | 90.3 | 32.06 | FAIL | PHYRE2 |
| HP0131 | 83.3 | 12.5 | 0.0 | 4.2 | 95.8 | 0.00 | FAIL | PHYRE2 |
| HP0132 | 70.2 | 20.7 | 3.7 | 5.4 | 90.9 | 50.22 | FAIL | PHYRE2 |
| HP0133 | 85.8 | 9.0 | 2.5 | 2.7 | 94.8 | 68.84 | WARNING | PHYRE2 |
| HP0134 | 90.9 | 6.8 | 1.3 | 1.0 | 97.7 | 86.10 | PASS | MODBASE |
| HP0135 | SMALL |  |  |  |  |  |  | PHYRE2 |
| HP0136 | 89.6 | 9.7 | 0.0 | 0.7 | 99.3 | 86.00 | PASS | MODBASE |
| HP0137 | 90.0 | 8.6 | 1.2 | 0.0 | 100 | 42.22 | FAIL | PHYRE2 |
| HP0138 | 69.3 | 20.8 | 4.7 | 5.2 | 90.1 | 43.36 | FAIL | PHYRE2 |
| HP0139 | 83.7 | 10.0 | 4.1 | 2.3 | 93.7 | 54.32 | FAIL | PHYRE2 |
| HP0140 | 81.4 | 13.9 | 1.7 | 3.0 | 95.3 | 42.91 | FAIL | PHYRE2 |
| HP0141 | 83.6 | 10.6 | 2.5 | 3.3 | 94.2 | 27.36 | FAIL | PHYRE2 |
| HP0142 | 83.1 | 12.8 | 2.7 | 1.4 | 95.9 | 75.91 | WARNING | MODBASE |
| HP0143 |  |  |  |  | NO PTN SEQ |  |  |  |
| HP0144 | 873.9 | 10.5 | 1.4 | 0.2 | 884.4 | 72.39 | WARNING | PHYRE2 |
| HP0145 | 81.5 | 15.1 | 2.4 | 1.0 | 96.6 | 53.22 | FAIL | PHYRE2 |
| HP0146 | 72.4 | 19.0 | 6.9 | 1.7 | 98.3 | 0.0 | FAIL | PHYRE2 |
| HP0147 | 77.2 | 15.2 | 4.0 | 3.6 | 92.4 | 49.13 | FAIL | PHYRE2 |
| HP0148 | 85.2 | 11.1 | 3.7 | 0.0 | 100 | 0.0 | FAIL | PHYRE2 |
| HP0149 | 92.7 | 4.9 | 0.8 | 1.6 | 98.4 | 64.75 | FAIL | PHYRE2 |
| HP0150 | 100 | 0.0 | 0.0 | 0.0 | 100 | 0.0 | FAIL | PHYRE2 |
| HP0151 | 92.7 | 5.7 | 0.8 | 0.8 | 98.4 | 9.59 | FAIL | PHYRE2 |
| HP0152 | 90.1 | 8.7 | 1.2 | 0.0 | 98.8 | 74.06 | WARNING | MODBASE |
| HP0153 | 89.6 | 7.1 | 3.0 | 0.3 | 96.7 | 73.91 | WARNING | MODBASE |
| HP0154 | 89.4 | 9.3 | 0.8 | 0.5 | 98.7 | 84.71 | PASS | MODBASE |
| HP0155 | 86.1 | 13.9 | 0.0 | 0.0 | 100 | 0.0 | FAIL | PHYRE2 |
| HP0156 | 57.5 | 25.1 | 7.3 | 10.1 | 82.6 | 23.88 | FAIL | PHYRE2 |
| HP0157 | 98.5 | 1.5 | 0.0 | 0.0 | 100 | 84.21 | PASS | PDB |
| HP0158 | 73.3 | 17.2 | 4.1 | 5.4 | 90.5 | 56.43 | FAIL | PHYRE2 |
| HP0159 | 77.7 | 11.9 | 5.2 | 5.2 | 89.6 | 63.00 | FAIL | PHYRE2 |
| HP0160 | 92.4 | 7.6 | 0.0 | 0.0 | 100 | 100.00 | PASS | MODBASE |
| HP0161 | SMALL |  |  |  |  |  |  |  |
| HP0162 | 88.6 | 10.9 | 0.5 | 0.0 |  | 92.31 | PASS | PDB |
| HP0163 | 92.3 | 5.6 | 1.5 | 0.5 | 97.9 | 93.30 | PASS | MODBASE |
| HP0164 | 78.5 | 16.3 | 3.0 | 2.1 | 94.8 | 36.08 | FAIL | PHYRE2 |
| HP0165 | 78.0 | 15.9 | 2.4 | 3.7 | 93.9 | 1.15 | FAIL | PHYRE2 |
| HP0166 | 83.0 | 13.8 | 2.1 | 1.1 | 98.9 | 0.0 | FAIL | PDB |
| HP0167 | 77.1 | 14.6 | 3.5 | 4.9 | 91.7 | 1.90 | FAIL | PHYRE2 |
| HP0168 | 94.8 | 5.2 | 0.0 | 0.0 | 100 | 25.88 | FAIL | PHYRE2 |
| HP0169 | 51.7 | 27.7 | 12.1 | 8.4 | 79.4 | 52.01 | FAIL | PHYRE2 |
| HP0170 | 80.0 | 11.9 | 6.0 | 2.1 | 91.9 | 6.30 | FAIL | PHYRE2 |
| HP0171 | 95.3 | 4.4 | 0.3 | 0.0 | 99.7 | 75.70 | WARNING | MODBASE |
| HP0172 | 85.1 | 12.0 | 2.0 | 0.9 | 97.1 | 69.39 | WARNING | MODBASE |
| HP0173 | 86.8 | 8.8 | 3.1 | 1.3 | 95.6 | 12.829 | FAIL | PHYRE2 |
| HP0174 | 88.1 | 10.2 | 1.3 | 0.4 | 98.3 | 1.93 | FAIL | PHYRE2 |
| HP0175 | 86.6 | 10.1 | 1.4 | 1.8 | 96.7 | 54.67 | FAIL | PHYRE2 |
| HP0176 | 91.9 | 7.7 | 0.0 | 0.4 | 99.6 | 99.32 | PASS | PDB |
| HP0177 | 91.4 | 7.4 | 0.6 | 0.6 | 99.4 | 83.96 | PASS | PHYRE2 |
| HP0178 | 92.1 | 6.9 | 1.0 | 0.0 | 99 | 77.68 | WARNING | MODBASE |
| HP0179 | 89.9 | 9.4 | 0.5 | 1.0 | 99.3 | 72.30 | WARNING | MODBASE |
| HP0180 | 87.0 | 9.4 | 2.5 | 1.1 | 96.4 | 63.00 | FAIL | PHYRE2 |
| HP0181 | 81.0 | 15.0 | 1.5 | 2.5 | 96 | 12.44 | FAIL | PHYRE2 |
| HP0182 | 92.0 | 6.6 | 0.9 | 0.5 | 98.6 | 65.99 | WARNING | MODBASE |
| HP0183 | 92.0 | 6.9 | 0.6 | 0.6 | 98.9 | 77.16 | WARNING | MODBASE |
| HP0184 | 87.2 | 11.5 | 0.6 | 0.6 | 98.7 | 100.00 | PASS | PDB |
| HP0185 | 59.8 | 26.4 | 6.9 | 6.9 | 86.2 | 22.01 | FAIL | PHYRE2 |
| HP0186 | 84.8 | 11.7 | 1.1 | 2.4 | 96.5 | 21.23 | FAIL | PHYRE2 |
| HP0187 | 91.7 | 8.3 | 0.0 | 0.0 | 100 | 0.0 | FAIL | PHYRE2 |
| HP0188 | 88.2 | 11.8 | 0.0 | 0.0 | 100 | 0.0 | FAIL | PHYRE2 |
| HP0189 | 84.5 | 10.1 | 1.8 | 3.6 | 94.6 | 0.0 | FAIL | PHYRE2 |
| HP0190 | 76.4 | 16.9 | 4.1 | 2.6 | 93.3 | 47.12 | FAIL | PHYRE2 |
| HP0191 | 89.8 | 9.3 | 0.9 | 0.0 | 99.1 | 90.53 | PASS | MODBASE |
| HP0192 | 91.8 | 7.4 | 0.8 | 0.0 | 99.2 | 85.82 | PASS | MODBASE |
| HP0193 | 95.9 | 3.7 | 0.5 | 0.0 | 99.6 | 40.08 | FAIL | PHYRE2 |
| HP0194 | 89.8 | 9.8 | 0.5 | 0.0 | 99.6 | 92.27 | PASS | PDB |
| HP0195 | 85.8 | 12.8 | 1.4 | 0.0 | 100 | 93.09 | PASS | PDB |
| HP0196 | 86.2 | 11.7 | 1.4 | 0.7 | 97.9 | 76.06 | WARNING | MODBASE |
| HP0197 | 91.6 | 7.5 | 0.9 | 0.0 | 99.1 | 93.47 | PASS | MODBASE |
| HP0198 | 96.7 | 2.5 | 0.0 | 0.8 | 99.2 | 80.88 | PASS | MODBASE |
| HP0199 | SMALL |  |  |  |  |  |  |  |
| HP0200 | 81.0 | 9.5 | 2.4 | 7.1 | 90.5 | 0.0 | FAIL | PHYRE2 |
| HP0201 | 84.5 | 13.1 | 1.0 | 1.3 | 97.6 | 84.21 | PASS | PHYRE2 |
| HP0202 | 92.1 | 6.2 | 1.7 | 0.0 | 98.3 | 92.97 | PASS | MODBASE |
| HP0203 | 79.0 | 17.3 | 0.0 | 3.7 | 96.3 | 50 | FAIL | PHYRE2 |
| HP0204 | 86.7 | 13.3 | 0.0 | 0.0 | 100 | 0.0 | FAIL | PHYRE2 |
| HP0205 | 77.6 | 16.5 | 3.2 | 2.7 | 94.1 | 35064 | FAIL | PHYRE2 |
| HP0206 | 85.0 | 8.3 | 6.7 | 0.0 | 100 | 47.89 | FAIL | PHYRE2 |
| HP0207 | 74.0 | 16.9 | 4.4 | 4.7 | 90.9 | 81.03 | PASS | PHYRE2 |
| HP0208 | 81.2 | 15.3 | 2.0 | 1.5 | 98.5 | 57.01 | FAIL | PHYRE2 |
| HP0209 | 75.3 | 17.6 | 4.8 | 2.3 | 92.9 | 31.04 | FAIL | PHYRE2 |
| HP0210 | 86.1 | 12.4 | 1.0 | 0.5 | 98.5 | 83.87 | PASS | MODBASE |
| HP0211 | 91.3 | 6.7 | 2.1 | 0.0 | 98 | 87.50 | PASS | MODBASE |
| HP0212 | 89.5 | 8.6 | 1.9 | 0.0 | 98.1 | 88.68 | PASS | MODBASE |
| HP0213 | 88.3 | 9.3 | 1.3 | 1.1 | 97.6 | 85.05 | PASS | PHYRE2 |
| HP0214 | 76.0 | 18.8 | 2.1 | 3.1 | 94.8 | 36.71 | FAIL | PHYRE2 |
| HP0215 | 82.5 | 12.7 | 1.3 | 3.5 | 95.2 | 17.98 | FAIL | PHYRE2 |
| HP0216 | 87.7 | 10.2 | 1.2 | 0.9 | 97.9 | 86.72 | PASS | MODBASE |
| HP0217 | 74.2 | 17.4 | 4.3 | 4.1 | 91.6 | 28.08 | FAIL | PHYRE2 |
| HP0218 | 90.2 | 9.6 | 0.2 | 0.0 | 99.8 | 96.56 | PASS | PDB |
| HP0219 | 91.9 | 6.3 | 0.9 | 0.9 | 99.1 | 10.74 | FAIL | PHYRE2 |
| HP0220 | 91.2 | 7.9 | 0.3 | 0.6 | 99.1 | 85.57 | PASS | MODBASE |
| HP0221 | 69.0 | 19.9 | 4.0 | 7.1 | 88.9 | 59.33 | FAIL | PHYRE2 |
| HP0222 | 85.0 | 10.0 | 3.8 | 1.2 | 98.8 | 100 | PASS | PDB |
| HP0223 | 82.2 | 12.6 | 2.0 | 3.3 | 94.8 | 63.70 | FAIL | PHYRE2 |
| HP0224 | 92.4 | 7.2 | 0.0 | 0.4 | 99.6 | 92.97 | PASS | MODBASE |
| HP0225 | SMALL(22) |  |  |  |  |  |  |  |
| HP0226 | 78.8 | 16.5 | 3.4 | 1.3 | 95.3 | 21.94 | FAIL | PHYRE2 |
| HP0227 | 68.4 | 17.5 | 9.3 | 4.7 | 85.9 | 49.28 | FAIL | PHYRE2 |
| HP0228 | 89.3 | 7.8 | 2.0 | 0.9 | 97.1 | 41.43 | FAIL | PHYRE2 |
| HP0229 | 65.7 | 20.6 | 6.4 | 7.3 | 86.3 | 26.24 | FAIL | PHYRE2 |
| HP0230 | 90.9 | 8.2 | 0.9 | 0.0 | 99.1 | 80.00 | PASS | MODBASE |
| HP0231 | 68.4 | 17.6 | 7.0 | 7.0 | 86 | 86.70 | PASS | PDB |
| HP0232 | 65.7 | 23.2 | 5.0 | 6.1 | 88.9 | 40.74 | FAIL | PHYRE2 |
| HP0233 | 84.4 | 12.2 | 1.7 | 1.7 | 96.6 | 70.33 | WARNING | MODBASE |
| HP0234 | 84.9 | 13.2 | 0.0 | 1.9 | 98.1 | 27.42 | FAIL | PHYRE2 |
| HP0235 | 88.2 | 9.8 | 2.1 | 0.0 | 98 | 80.55 | PASS | MODBASE |
| HP0236 | 78.2 | 17.3 | 2.7 | 1.8 | 95.5 | 50.00 | FAIL | PHYRE2 |
| HP0237 | 93.3 | 6.3 | 0.0 | 0.4 | 99.6 | 85.90 | PASS | MODBASE |
| HP0238 | 91.7 | 6.7 | 1.3 | 0.2 | 98.4 | 81.57 | PASS | MODBASE |
| HP0239 | 87.1 | 10.0 | 1.7 | 1.2 | 97.1 | 74.59 | WARNING | PHYRE2 |
| HP0240 | 93.7 | 5.3 | 1.1 | 0.0 | 99 | 100 | PASS | PDB |
| HP0241 | 69.7 | 23.03 | 6.6 | 0.8 | 99.2 | 9.09 | FAIL | PHYRE2 |
| HP0242 | 95.6 | 4.4 | 0.0 | 0.0 | 100 | 95.28 | PASS | PDB |
| HP0243 | 95.7 | 4.3 | 0.0 | 0.0 | 100 | 88.97 | PASS | PDB |
| HP0244 | 76.0 | 15.9 | 4.7 | 3.4 | 91.9 | 36.13 | FAIL | PHYRE2 |
| HP0245 | 72.2 | 27.8 | 0.0 | 0.0 | 100 | 0.0 | FAIL | PHYRE2 |
| HP0246 | 71.0 | 20.8 | 6.2 | 2.0 | 91.8 | 28.57 | FAIL | PHYRE2 |
| HP0247 | 93.3 | 6.1 | 0.3 | 0.3 | 99.4 | 82.72 | PASS | MODBASE |
| HP0248 | 80.6 | 15.3 | 2.2 | 1.9 | 95.9 | 36.09 | FAIL | PHYRE2 |
| HP0249 | 100 | 0.0 | 0.0 | 0.0 | 100 | 0.0 | FAIL | PHYRE2 |
| HP0250 | 80.1 | 14.2 | 3.8 | 1.9 | 94.3 | 64.99 | FAIL | PHYRE2 |
| HP0251 | 84.1 | 12.0 | 2.7 | 1.3 | 96.1 | 39.53 | FAIL | PHYRE2 |
| HP0252 | 69.1 | 20.4 | 4.9 | 5.6 | 89.5 | 43.44 | FAIL | PHYRE2 |
| HP0253 | 85.3 | 8.8 | 5.9 | 0.0 | 94.1 | 0.0 | FAIL | PHYRE2 |
| HP0254 | 67.7 | 21.3 | 3.7 | 7.2 | 89 | 38.66 | FAIL | PHYRE2 |
| HP0255 | 88.2 | 10.7 | 0.8 | 0.3 | 98.9 | 87.04 | PASS | MODBASE |
| HP0256 | 100 | 0.0 | 0.0 | 0.0 | 100 | 0.0 | FAIL | PHYRE2 |
| HP0257 | 86.9 | 6.8 | 4.7 | 1.6 | 93.7 | 11.82 | FAIL | PHYRE2 |
| HP0258 | 63.0 | 25.6 | 5.2 | 6.2 | 88.6 | 22.06 | FAIL | PHYRE2 |
| HP0259 | 71.5 | 20.0 | 3.1 | 5.4 | 91.5 | 45.61 | FAIL | PHYRE2 |
| HP0260 | 75.4 | 18.5 | 4.5 | 1.7 | 93.9 | 38.44 | FAIL | PHYRE2 |
| HP0261 | 80.0 | 17.8 | 0.0 | 2.2 | 97.8 | 0.0 | FAIL | PHYRE2 |
| HP0262 | 69.7 | 20.2 | 6.2 | 3.9 | 89.9 | 23.20 | FAIL | PHYRE2 |
| HP0263 | 79.2 | 15.5 | 4.4 | 0.9 | 94.7 | 79.45 | WARNING | PHYRE2 |
| HP0264 | 88.8 | 8.5 | 1.9 | 0.8 | 97.3 | 74.79 | WARNING | MODBASE |
| HP0265 | 86.9 | 11.3 | 0.5 | 1.4 | 98.2 | 1.24 | FAIL | PHYRE2 |
| HP0266 | 86.2 | 11.1 | 1.8 | 0.9 | 97.3 | 88.36 | PASS | PHYRE2 |
| HP0267 | 76.9 | 20.2 | 1.3 | 1.6 | 97.1 | 79.76 | WARNING | PHYRE2 |
| HP0268 | 81.6 | 15.8 | 2.6 | 0.0 | 97.4 | 35.57 | FAIL | PHYRE2 |
| HP0269 | 68.0 | 19.8 | 5.8 | 6.3 | 87.8 | 72.60 | WARNING | PHYRE2 |
| HP0270 | 85.1 | 10.6 | 2.1 | 2.1 | 97.9 | 24.56 | FAIL | PHYRE2 |
| HP0271 | 70.5 | 18.9 | 9.0 | 1.6 | 89.4 | 28.96 | FAIL | PHYRE2 |
| HP0272 | 92.4 | 7.6 | 0.0 | 0.0 | 100 | 0.0 | FAIL | PHYRE2 |
| HP0273 | 68.1 | 23.5 | 4.2 | 4.2 | 91.6 | 57.78 | FAIL | PHYRE2 |
| HP0274 | 70.3 | 22.0 | 5.9 | 1.7 | 92.3 | 21.05 | FAIL | PHYRE2 |
| HP0275 | 74.4 | 15.7 | 4.9 | 4.9 | 90.1 | 32.48 | FAIL | PHYRE2 |
| HP0276 | 82.4 | 12.7 | 4.2 | 0.6 | 95.1 | 43.01 | FAIL | PHYRE2 |
| HP0277 | 87.3 | 12.7 | 0.0 | 0.0 | 100 | 79.52 | WARNING | MODBASE |
| HP0278 | 91.3 | 8.0 | 0.7 | 0.0 | 99.3 | 81.95 | PASS | MODBASE |
| HP0279 | 90.0 | 8.4 | 0.6 | 1.0 | 98.4 | 65.78 | WARNING | MODBASE |
| HP0280 | 64.6 | 20.9 | 6.6 | 7.9 | 85.5 | 25.53 | FAIL | PHYRE2 |
| HP0281 | 92.7 | 6.1 | 0.6 | 0.6 | 98.8 | 97.83 | PASS | MODBASE |
| HP0282 | 80.2 | 15.1 | 3.4 | 1.4 | 95.3 | 42.83 | FAIL | PHYRE2 |
| HP0283 | 89.9 | 9.4 | 0.7 | 0.0 | 100 | 92.56 | PASS | PDB |
| HP0284 | 72.5 | 19.4 | 3.8 | 4.3 | 91.9 | 22.52 | FAIL | PHYRE2 |
| HP0285 | 77.8 | 15.9 | 5.0 | 1.3 | 93.7 | 57.52 | FAIL | PHYRE2 |
| HP0286 | 78.9 | 13.4 | 4.9 | 2.8 | 92.3 | 58.26 | FAIL | PHYRE2 |
| HP0287 | 78.5 | 16.5 | 3.2 | 1.9 | 95 | 49.13 | FAIL | PHYRE2 |
| HP0288 | 94.1 | 5.9 | 0.0 | 0.0 | 100 | 0.0 | FAIL | PHYRE2 |
| HP0289 | LONG |  |  |  | 0 |  |  |  |
| HP0290 | 90.8 | 9.2 | 0.0 | 0.0 | 100 | 88.35 | PASS | PDB |
| HP0291 | 94.4 | 4.5 | 1.1 | 0.0 | 100 | 20.83 | FAIL | PHYRE2 |
| HP0292 | 90.7 | 8.1 | 0.7 | 0.4 | 99.6 | 69.90 | WARNING | PHYRE2 |
| HP0293 | 87.6 | 9.6 | 1.4 | 1.4 | 97.2 | 70.64 | WARNING | PHYRE2 |
| HP0294 | 92.7 | 6.9 | 0.0 | 0.3 | 99.6 | 77.94 | WARNING | MODBASE |
| HP0295 | 75.4 | 18.0 | 4.0 | 2.6 | 93.4 | 37.88 | FAIL | PHYRE2 |
| HP0296 | 51.7 | 39.3 | 5.6 | 3.4 | 96.6 | 92.23 | PASS | PHYRE2 |
| HP0297 | 81.4 | 17.1 | 1.4 | 0.0 | 98.5 | 89.41 | PASS | MODBASE |
| HP0298 | 93.0 | 6.5 | 0.4 | 0.0 | 99.5 | 91.05 | PASS | MODBASE |
| HP0299 | 79.3 | 15.3 | 2.0 | 3.4 | 94.6 | 21.19 | FAIL | PHYRE2 |
| HP0300 | 83.9 | 10.7 | 2.5 | 2.9 | 94.6 | 24.13 | FAIL | PHYRE2 |
| HP0301 | 79.1 | 14.5 | 4.4 | 2.0 | 93.6 | 71.98 | WARNING | MODBASE |
| HP0302 | 88.3 | 9.9 | 0.9 | 0.9 | 98.2 | 69.80 | WARNING | MODBASE |
| HP0303 | 85.3 | 13.0 | 1.7 | 0.0 | 98.3 | 81.67 | PASS | MODBASE |
| HP0304 | 69.8 | 20.8 | 5.0 | 4.4 | 90.6 | 69.70 | WARNING | PHYRE2 |
| HP0305 | 85.3 | 10.0 | 4.1 | 0.6 | 95.3 | 86.49 | PASS | PHYRE2 |
| HP0306 | 89.5 | 9.5 | 0.5 | 0.5 | 99 | 96.71 | PASS | MODBASE |
| HP0307 | 84.8 | 11.4 | 3.8 | 0.0 | 96.2 | 0.0 | FAIL | PHYRE2 |
| HP0308 | 100 | 0.0 | 0.0 | 0.0 | 100 | 0.0 | FAIL | PHYRE2 |
| HP0309 | 88.4 | 8.0 | 2.0 | 1.6 | 96.4 | 85.25 | PASS | MODBASE |
| HP0310 | 85.0 | 12.5 | 2.3 | 0.2 | 99.8 | 0.0 | FAIL | PDB |
| HP0311 | 100 | 0.0 | 0.0 | 0.0 | 100 | 0.0 | FAIL | PHYRE2 |
| HP0312 | 90.4 | 7.9 | 1.4 | 0.4 | 98.3 | 81.03 | PASS | PHYRE2 |
| HP0313 | 85.1 | 10.1 | 3.9 | 0.9 | 95.2 | 62.04 | FAIL | PHYRE2 |
| HP0314 | 91.7 | 8.3 | 0.0 | 0.0 | 100 | 0.0 | FAIL | PHYRE2 |
| HP0315 | 78.4 | 15.9 | 4.5 | 1.1 | 94.3 | 88.98 | PASS | PDB |
| HP0316 | 89.8 | 10.2 | 0.0 | 0.0 | 100 | 36.54 | FAIL | PHYRE2 |
| HP0317 | 67.7 | 19.7 | 5.5 | 7.1 | 87.4 | 46.78 | FAIL | PHYRE2 |
| HP0318 | 91.2 | 8.6 | 0.2 | 0.0 | 100 | 0.0 | FAIL | PDB |
| HP0319 | 91.0 | 7.0 | 1.4 | 0.6 | 98 | 74.72 | WARNING | PHYRE2 |
| HP0320 | 75.4 | 17.4 | 2.9 | 4.3 | 100 | 0.00 | FAIL | PHYRE2 |
| HP0321 | 95.3 | 4.7 | 0.0 | 0.0 | 100 | 67.89 | WARNING | MODBASE |
| HP0322 | 78.8 | 14.4 | 4.5 | 2.4 | 93.2 | 20.95 | FAIL | PHYRE2 |
| HP0323 | 83.6 | 14.5 | 1.9 | 0.0 | 100 | 35.59 | FAIL | PHYRE2 |
| HP0324 | 68.8 | 18.1 | 9.5 | 3.6 | 86.9 | 36.86 | FAIL | PHYRE2 |
| HP0325 | 56.5 | 26.3 | 5.7 | 11.5 | 82.8 | 32.35 | FAIL | PHYRE2 |
| HP0326 | 74.5 | 17.7 | 4.8 | 2.9 | 92.2 | 46.14 | FAIL | PHYRE2 |
| HP0327 | 82.8 | 14.0 | 2.5 | 0.6 | 99.4 | 35.06 | FAIL | PHYRE2 |
| HP0328 | 59.7 | 29.1 | 6.1 | 5.0 | 88.8 | 24.60 | FAIL | PHYRE2 |
| HP0329 | 93.7 | 5.7 | 0.4 | 0.2 | 99.8 | 91.70 | PASS | PDB |
| HP0330 | 93.6 | 6.4 | 0.0 | 0.0 | 100 | 78.29 | WARNING | MODBASE |
| HP0331 | 86.6 | 10.3 | 1.3 | 1.7 | 96.9 | 65.37 | WARNING | MODBASE |
| HP0332 | 91.8 | 8.2 | 0.0 | 0.0 | 100 | 73.33 | WARNING | PDB |
| HP0333 | 87.7 | 10.2 | 1.7 | 0.4 | 97.9 | 71.43 | WARNING | PHYRE2 |
| HP0334 | 88.0 | 10.3 | 0.0 | 1.7 | 98.3 | 69.77 | WARNING | MODBASE |
| HP0335 | 86.7 | 13.3 | 0.0 | 0.0 | 100 | 100.00 | PASS | MODBASE |
| HP0336 | 94.9 | 5.1 | 0.0 | 0.0 | 100 | 78.36 | WARNING | PDB |
| HP0337 | 85.4 | 11.5 | 2.1 | 1.0 | 96.9 | 0.98 | FAIL | PHYRE2 |
| HP0338 | 79.5 | 18.2 | 0.0 | 2.3 | 97.7 | 0.0 | FAIL | PHYRE2 |
| HP0339 | 90.9 | 6.1 | 1.0 | 2.0 | 98 | 71.30 | FAIL | PHYRE2 |
| HP0340 | 76.9 | 14.1 | 2.6 | 6.4 | 91 | 23.60 | FAIL | PHYRE2 |
| HP0341 | 69.2 | 30.8 | 0.0 | 0.0 | 100 | 0.00 | FAIL | PHYRE2 |
| HP0342 | 90.9 | 8.3 | 0.8 | 0.0 | 99.2 | 0.0 | FAIL | PHYRE2 |
| HP0343 | SMALL |  |  |  |  |  |  |  |
| HP0344 | 90.8 | 7.1 | 0.7 | 1.4 | 97.9 | 2.58 | FAIL | PHYRE2 |
| HP0345 | 76.8 | 20.2 | 0.0 | 3.0 | 97 | 5.36 | FAIL | PHYRE2 |
| HP0346 | 76.0 | 15.0 | 3.4 | 5.6 | 91 | 36.78 | FAIL | PHYRE2 |
| HP0347 | 85.5 | 12.1 | 2.4 | 0.0 | 97.6 | 72.03 | WARNING | PHYRE2 |
| HP0348 | 81.4 | 14.7 | 2.8 | 1.1 | 96.1 | 66.39 | WARNING | PHYRE2 |
| HP0349 | 89.6 | 8.1 | 1.1 | 1.3 | 97.7 | 92.94 | PASS | MODBASE |
| HP0350 | 92.9 | 5.7 | 0.7 | 0.7 | 98.6 | 41.51 | FAIL | PHYRE2 |
| HP0351 | 93.1 | 5.5 | 1.4 | 0.0 | 98.6 | 31.10 | FAIL | PHYRE2 |
| HP0352 | 94.7 | 4.1 | 0.9 | 0.3 | 98.8 | 92.02 | PASS | PDB |
| HP0353 | 75.2 | 18.2 | 2.5 | 4.1 | 93.4 | 19.69 | FAIL | PHYRE2 |
| HP0354 | 88.8 | 7.8 | 2.8 | 0.6 | 96.6 | 88.83 | PASS | PHYRE2 |
| HP0355 | 92.2 | 6.8 | 0.8 | 0.2 | 99 | 83.66 | PASS | MODBASE |
| HP0356 | 67.0 | 21.3 | 6.5 | 5.2 | 88.3 | 35.57 | FAIL | PHYRE2 |
| HP0357 | 89.8 | 7.9 | 1.9 | 0.5 | 97.7 | 85.60 | PASS | PHYRE2 |
| HP0358 | 70.1 | 21.5 | 6.5 | 1.9 | 91.6 | 4.10 | FAIL | PHYRE2 |
| HP0359 | SMALL-21 |  |  |  |  |  |  |  |
| HP0360 | 87.6 | 9.8 | 1.3 | 1.3 | 97.4 | 83.14 | PASS | MODBASE |
| HP0361 | 86.2 | 11.5 | 1.4 | 0.9 | 97.7 | 76.54 | WARNING | PHYRE2 |
| HP0362 | 74.4 | 18.3 | 3.5 | 3.8 | 92.7 | 27.75 | FAIL | PHYRE2 |
| HP0363 | 91.0 | 7.4 | 1.1 | 0.5 | 99.5 | 75 | WARNING | PHYRE2 |
| HP0364 | 94.1 | 5.6 | 0.3 | 0.0 | 99.7 | 77.42 | WARNING | MODBASE |
| HP0365 | 64.0 | 20.0 | 4.0 | 12.0 | 84 | 54.84 | FAIL | PHYRE2 |
| HP0366 | 90.1 | 9.6 | 0.0 | 0.3 | 99.7 | 98.66 | PASS | PDB |
| HP0367 | 90.5 | 4.7 | 1.1 | 3.7 | 95.2 | 10.84 | FAIL | PHYRE2 |
| HP0368 | 71.1 | 21.5 | 4.1 | 3.3 | 92.6 | 15.67 | FAIL | PHYRE2 |
| HP0369 | 78.2 | 15.0 | 3.6 | 3.2 | 93.2 | 13.50 | FAIL | PHYRE2 |
| HP0370 | 92.4 | 5.6 | 1.3 | 0.8 | 98 | 97.75 | PASS | MODBASE |
| HP0371 | 81.2 | 14.3 | 3.0 | 1.5 | 95.5 | 35.67 | FAIL | PHYRE2 |
| HP0372 | 82.0 | 12.4 | 4.3 | 1.2 | 94.4 | 80.95 | PASS | PHYRE2 |
| HP0373 | 55.1 | 31.3 | 6.4 | 7.2 | 86.4 | 22.68 | FAIL | PHYRE2 |
| HP0374 | 87.3 | 8.3 | 2.0 | 2.4 | 95.6 | 79.30 | WARNING | PHYRE2 |
| HP0375 | 100 | 0.0 | 0.0 | 0.0 | 100 | 0.0 | FAIL | PHYRE2 |
| HP0376 | 88.7 | 6.9 | 2.7 | 1.7 | 95.6 | 68.34 | WARNING | MODBASE |
| HP0377 | 91.9 | 8.1 | 0.0 | 0.0 | 100 | 99.38 | PASS | PDB |
| HP0378 | 73.7 | 19.0 | 3.8 | 3.5 | 92.7 | 35.33 | FAIL | PHYRE2 |
| HP0379 | 83.4 | 14.0 | 1.3 | 1.3 | 97.4 | 98.57 | PASS | MODBASE |
| HP0380 | 89.4 | 9.0 | 1.0 | 0.5 | 98.4 | 89.29 | PASS | MODBASE |
| HP0381 | 88.0 | 9.1 | 1.2 | 1.7 | 97.1 | 77.37 | WARNING | PHYRE2 |
| HP0382 | 79.1 | 13.5 | 2.5 | 5.0 | 92.6 | 36.52 | FAIL | PHYRE2 |
| HP0383 | 80.6 | 15.0 | 1.9 | 2.5 | 97.5 | 16.57 | FAIL | PHYRE2 |
| HP0384 | 68.5 | 18.5 | 7.4 | 5.6 | 94.4 | 74.24 | WARNING | PHYRE2 |
| HP0385 | 93.0 | 4.2 | 1.4 | 1.4 | 97.2 | 0.00 | FAIL | PHYRE2 |
| HP0386 | 100 | 0.0 | 0.0 | 0.0 | 100 | 0.0 | FAIL | PHYRE2 |
| HP0387 | 65.7 | 23.2 | 5.8 | 5.3 | 88.9 | 51.61 | FAIL | PHYRE2 |
| HP0388 | 87.7 | 8.0 | 2.8 | 1.4 | 95.7 | 65.22 | WARNING | PHYRE2 |
| HP0389 | 87.0 | 10.2 | 2.8 | 0.0 | 97.2 | 97.41 | PASS | PDB |
| HP0390 | 89.6 | 8.3 | 0.7 | 1.4 | 97.9 | 75.45 | WARNING | MODBASE |
| HP0391 | 90.6 | 7.0 | 0.8 | 1.6 | 97.6 | 85.03 | PASS | PHYRE2 |
| HP0392 | 79.4 | 13.6 | 3.6 | 3.4 | 93 | 56.09 | FAIL | PHYRE2 |
| HP0393 | 81.7 | 12.7 | 2.8 | 2.8 | 94.4 | 78.85 | WARNING | PHYRE2 |
| HP0394 | 53.3 | 29.1 | 5.7 | 11.9 | 82.4 | 64.43 | FAIL | PHYRE2 |
| HP0395 | 81.3 | 12.8 | 3.4 | 2.5 | 94.1 | 74.43 | WARNING | MODBASE |
| HP0396 | 69.6 | 21.0 | 4.3 | 5.1 | 90.6 | 60.45 | FAIL | PHYRE2 |
| HP0397 | 87.8 | 10.9 | 0.9 | 0.4 | 98.7 | 86.71 | PASS | PHYRE2 |
| HP0398 | 69.2 | 25.0 | 2.9 | 2.9 | 97.1 | 7.65 | FAIL | PHYRE2 |
| HP0399 | 63.0 | 25.8 | 6.4 | 4.8 | 88.8 | 43.45 | FAIL | PHYRE2 |
| HP0400 | 92.1 | 5.6 | 1.6 | 0.8 | 97.7 | 69.09 | WARNING | MODBASE |
| HP0401 | 85.9 | 10.7 | 2.6 | 0.8 | 96.6 | 91.75 | PASS | MODBASE |
| HP0402 | 87.1 | 10.6 | 1.4 | 0.9 | 99.1 | 69.99 | WARNING | PHYRE2 |
| HP0403 | 77.4 | 17.2 | 3.0 | 2.4 | 94.6 | 73.36 | WARNING | MODBASE |
| HP0404 | 84.8 | 14.9 | 0.2 | 0.0 | 100 | 89.22 | PASS | PDB |
| HP0405 | 81.1 | 12.4 | 3.9 | 2.6 | 93.5 | 73.24 | WARNING | PHYRE2 |
| HP0406 | 75.7 | 16.8 | 3.2 | 4.3 | 95.7 | 1.02 | FAIL | PHYRE2 |
| HP0407 | 87.5 | 10.2 | 1.6 | 0.6 | 97.7 | 88.77 | PASS | MODBASE |
| HP0408 | 66.4 | 22.1 | 4.0 | 7.4 | 88.5 | 9.82 | FAIL | PHYRE2 |
| HP0409 | 89.5 | 7.8 | 1.1 | 1.6 | 97.3 | 89.98 | PASS | MODBASE |
| HP0410 | 88.9 | 10.5 | 0.6 | 0.0 | 99.4 | 85.03 | PASS | PDB |
| HP0411 | 73.9 | 14.8 | 6.8 | 4.5 | 88.7 | 23.58 | FAIL | PHYRE2 |
| HP0412 | 85.2 | 11.1 | 0.0 | 3.7 | 96.3 | 0.00 | FAIL | PHYRE2 |
| HP0413 | 63.5 | 27.7 | 5.2 | 3.6 | 91.2 | 10.41 | FAIL | PHYRE2 |
| HP0414 | 89.8 | 8.5 | 0.8 | 0.8 | 98.3 | 84.09 | PASS | MODBASE |
| HP0415 | 70.3 | 19.2 | 5.0 | 5.5 | 89.5 | 18.59 | FAIL | PHYRE2 |
| HP0416 | 86.9 | 10.3 | 1.1 | 1.7 | 97.2 | 83.27 | PASS | MODBASE |
| HP0417 | 80.2 | 18.8 | 0.8 | 0.2 | 99 | 76.71 | WARNING | MODBASE |
| HP0418 | 75.6 | 18.5 | 5.2 | 0.6 | 94.1 | 25.60 | FAIL | PHYRE2 |
| HP0419 | 74.7 | 17.9 | 3.9 | 3.5 | 92.6 | 51.91 | FAIL | PHYRE2 |
| HP0420 | 90.7 | 7.0 | 2.3 | 0.0 | 100 | 89.29 | PASS | PHYRE2 |
| HP0421 | 92.9 | 7.1 | 0.0 | 0.0 | 100 | 97.75 | PASS | PDB |
| HP0422 | 91.2 | 7.0 | 1.6 | 0.2 | 98.2 | 82.90 | PASS | MODBASE |
| HP0423 | 69.5 | 22.2 | 4.0 | 4.4 | 91.7 | 24.27 | FAIL | PHYRE2 |
| HP0424 | 71.8 | 20.0 | 3.8 | 4.3 | 91.8 | 28.39 | FAIL | PHYRE2 |
| HP0425 | 69.4 | 20.7 | 5.5 | 4.5 | 90.1 | 59.09 | FAIL | PHYRE2 |
| HP0426 | 71.8 | 19.9 | 3.3 | 4.9 | 91.7 | 24.87 | FAIL | PHYRE2 |
| HP0427 | 100 | 0.0 | 0.0 | 0.0 | 100 | 0.0 | FAIL | PHYRE2 |
| HP0428 | 83.1 | 15.5 | 1.4 | 0.0 | 100 | 49.08 | FAIL | PHYRE2 |
| HP0429 | TOO SMALL |  |  |  | 0 |  |  |  |
| HP0430 | 78.0 | 20.0 | 0.0 | 2.0 | 98 | 10.71 | FAIL | PHYRE2 |
| HP0431 | 87.6 | 6.7 | 3.8 | 1.9 | 94.3 | 62.45 | FAIL | PHYRE2 |
| HP0432 | 79.0 | 13.7 | 4.8 | 2.6 | 92.7 | 54.21 | FAIL | PHYRE2 |
| HP0433 | 87.5 | 8.3 | 0.0 | 4.2 | 95.8 | 0.0 | FAIL | PHYRE2 |
| HP0434 | 71.7 | 19.2 | 5.8 | 3.3 | 90.9 | 11.54 | FAIL | PHYRE2 |
| HP0435 | 90.8 | 7.5 | 0.6 | 1.2 | 98.3 | 34.90 | FAIL | PHYRE2 |
| HP0436 | 96.2 | 2.5 | 1.3 | 0.0 | 100 | 49.43 | FAIL | PHYRE2 |
| HP0437 | 89.5 | 8.9 | 1.6 | 0.0 | 98.4 | 75.37 | WARNING | MODBASE |
| HP0438 | 81.2 | 18.8 | 0.0 | 0.0 | 100 | 21.05 | FAIL | PHYRE2 |
| HP0439 | 74.4 | 17.4 | 2.9 | 5.3 | 91.8 | 10.08 | FAIL | PHYRE2 |
| HP0440 | 74.6 | 16.5 | 5.9 | 3.0 | 91.1 | 64.16 | FAIL | PHYRE2 |
| HP0441 | 71.9 | 19.6 | 3.9 | 4.6 | 91.5 | 27.35 | FAIL | PHYRE2 |
| HP0442 | 85.4 | 12.2 | 0.0 | 2.4 | 97.6 | 2.25 | FAIL | PHYRE2 |
| HP0443 | 88.8 | 11.2 | 0.0 | 0.0 | 100 | 0.00 | FAIL | PHYRE2 |
| HP0444 | 73.0 | 19.4 | 4.8 | 2.8 | 92.4 | 59.06 | FAIL | PHYRE2 |
| HP0445 | 64.8 | 14.8 | 1.9 | 18.5 | 79.6 | 0.0 | FAIL | PHYRE2 |
| HP0446 | 67.6 | 22.7 | 5.9 | 3.8 | 90.3 | 38.08 | FAIL | PHYRE2 |
| HP0447 | 86.7 | 11.8 | 1.5 | 0.0 | 98.5 | 62.61 | FAIL | PHYRE2 |
| HP0448 | 74.1 | 19.8 | 4.3 | 1.7 | 93.9 | 27.91 | FAIL | PHYRE2 |
| HP0449 | 77.8 | 13.9 | 3.1 | 5.2 | 91.7 | 29.39 | FAIL | PHYRE2 |
| HP0450 | 91.4 | 8.6 | 0.0 | 0.0 | 100 | 0.0 | FAIL | PHYRE2 |
| HP0451 | 91.7 | 8.3 | 0.0 | 0.0 | 100 | 0.0 | FAIL | PHYRE2 |
| HP0452 | 72.9 | 17.3 | 5.8 | 4.0 | 90.2 | 35.65 | FAIL | PHYRE2 |
| HP0453 | 83.3 | 14.8 | 0.0 | 1.9 | 98.1 | 98.46 | PASS | I- TASSER |
| HP0454 | 84.1 | 14.3 | 1.1 | 0.5 | 98.4 | 42.00 | FAIL | PHYRE2 |
| HP0455 | 73.1 | 19.2 | 3.8 | 3.8 | 96.2 | 0.0 | FAIL | PHYRE2 |
| HP0456 | 91.7 | 5.6 | 2.8 | 0.0 | 97.3 | 0.0 | FAIL | PHYRE2 |
| HP0457 | 92.6 | 6.2 | 0.0 | 1.2 | 98.8 | 0.0 | FAIL | PHYRE2 |
| HP0458 | 71.2 | 23.3 | 2.7 | 2.7 | 94.5 | 24.05 | FAIL | PHYRE2 |
| HP0459 | 73.2 | 19.3 | 3.3 | 4.2 | 92.5 | 38.18 | FAIL | PHYRE2 |
| HP0460 | 79.5 | 15.4 | 2.6 | 2.6 | 94.4 | 0.0 | FAIL | PHYRE2 |
| HP0461 | 92.4 | 0.0 | 3.6 | 3.6 | 92.4 | 0.00 | FAIL | PHYRE2 |
| HP0462 | 85.2 | 9.8 | 2.8 | 2.2 | 95 | 57.38 | FAIL | PHYRE2 |
| HP0463 | 81.7 | 11.8 | 3.6 | 2.9 | 93.5 | 62.91 | FAIL | PHYRE2 |
| HP0464 | 86.0 | 12.2 | 1.6 | 0.2 | 99.8 | 67.06 | WARNING | PHYRE2 |
| HP0465 | 74.7 | 17.6 | 4.0 | 3.7 | 92.3 | 38.77 | FAIL | PHYRE2 |
| HP0466 | 70.1 | 20.3 | 5.4 | 4.1 | 90.4 | 14.84 | FAIL | PHYRE2 |
| HP0467 | 85.8 | 9.4 | 2.8 | 1.9 | 95.2 | 0.0 | FAIL | PHYRE2 |
| HP0468 | 87.3 | 11.4 | 0.9 | 0.4 | 98.7 | 97.58 | PASS | PHYRE2 |
| HP0469 | 89.4 | 9.4 | 0.0 | 1.2 | 98.8 | 48.98 | FAIL | PHYRE2 |
| HP0470 | 89.5 | 7.2 | 1.7 | 1.7 | 96.7 | 83.59 | PASS | PHYRE2 |
| HP0471 | 90.1 | 7.0 | 1.1 | 1.8 | 97.1 | 77.37 | WARNING | PHYRE2 |
| HP0472 | 86.0 | 9.6 | 2.9 | 1.5 | 98.5 | 42.60 | FAIL | PHYRE2 |
| HP0473 | 90.6 | 6.9 | 2.5 | 0.0 | 97.5 | 90.18 | PASS | MODBASE |
| HP0474 | 92.5 | 4.5 | 2.0 | 1.0 | 97 | 46.22 | FAIL | PHYRE2 |
| HP0475 | 86.4 | 9.5 | 2.5 | 1.5 | 95.9 | 77.73 | WARNING | MODBASE |
| HP0476 | 91.8 | 6.7 | 0.5 | 1.0 | 98.5 | 88.96 | PASS | MODBASE |
| HP0477 | 69.0 | 22.8 | 5.2 | 3.0 | 91.8 | 29.62 | FAIL | PHYRE2 |
| HP0478 | 59.6 | 25.9 | 8.3 | 6.1 | 85.5 | 43.96 | FAIL | PHYRE2 |
| HP0479 | 75.5 | 18.9 | 2.7 | 2.7 | 94.4 | 43.82 | FAIL | PHYRE2 |
| HP0480 | 83.2 | 12.2 | 3.2 | 1.3 | 95.4 | 67.00 | WARNING | PHYRE2 |
| HP0481 | 78.9 | 18.4 | 1.1 | 1.6 | 97.3 | 58.02 | FAIL | PHYRE2 |
| HP0482 | 85.1 | 11.7 | 3.2 | 0.0 | 100 | 17.12 | FAIL | PHYRE2 |
| HP0483 |  |  |  |  | NO PTN SEQ |  |  |  |
| HP0484 | 68.1 | 23.3 | 4.7 | 3.9 | 91.4 | 1.99 | FAIL | PHYRE2 |
| HP0485 | 78.1 | 15.1 | 4.3 | 2.5 | 93.2 | 70.48 | WARNING | PHYRE2 |
| HP0486 | 69.8 | 22.1 | 4.4 | 3.7 | 91.9 | 63.33 | FAIL | PHYRE2 |
| HP0487 | 74.6 | 16.6 | 6.1 | 2.7 | 91.2 | 59.67 | FAIL | PHYRE2 |
| HP0488 | 78.3 | 16.0 | 2.7 | 2.9 | 94.3 | 27.77 | FAIL | PHYRE2 |
| HP0489 | 76.5 | 16.0 | 2.8 | 4.6 | 92.5 | 0.00 | FAIL | PHYRE2 |
| HP0490 | 72.8 | 18.4 | 6.0 | 2.8 | 91.2 | 0.34 | FAIL | PHYRE2 |
| HP0491 | 74.1 | 24.1 | 1.9 | 0.0 | 100 | 16.13 | FAIL | PHYRE2 |
| HP0492 | 96.5 | 3.5 | 0.0 | 0.0 | 100 | 88.74 | PASS | PDB |
| HP0493 | 76.4 | 17.2 | 3.2 | 3.2 | 93.6 | 39.83 | FAIL | PHYRE2 |
| HP0494 | 84.8 | 10.8 | 2.1 | 2.3 | 95.6 | 74.47 | WARNING | PHYRE2 |
| HP0495 | 92.9 | 7.1 | 0.0 | 0.0 | 100 | 97.75 | FAIL | PDB |
| HP0496 | 91.5 | 8.0 | 0.4 | 0.0 | 99.5 | 97.04 | PASS | PDB |
| HP0497 | 91.1 | 8.1 | 0.3 | 0.5 | 99.5 | 72.05 | WARNING | PHYRE2 |
| HP0498 | 87.7 | 9.3 | 1.5 | 1.5 | 97 | 67.95 | WARNING | PHYRE2 |
| HP0499 | 76.8 | 17.9 | 3.0 | 2.3 | 94.7 | 64.04 | FAIL | PHYRE2 |
| HP0500 | 85.3 | 12.1 | 1.7 | 0.9 | 97.4 | 84.00 | PASS | PHYRE2 |
| HP0501 | 85.4 | 10.4 | 2.6 | 1.6 | 95.8 | 75.06 | WARNING | PHYRE2 |
| HP0502 | 51.1 | 31.1 | 4.4 | 13.3 | 82.2 | 30.09 | FAIL | PHYRE2 |
| HP0503 | 75.6 | 16.0 | 3.6 | 4.9 | 91.6 | 18.62 | FAIL | PHYRE2 |
| HP0504 | 65.1 | 20.9 | 4.7 | 9.3 | 86 | 2.00 | FAIL | PHYRE2 |
| HP0505 | 84.0 | 11.8 | 2.8 | 1.4 | 95.8 | 39.35 | FAIL | PHYRE2 |
| HP0506 | 76.7 | 17.0 | 4.5 | 3.9 | 93.7 | 62.13 | FAIL | PHYRE2 |
| HP0507 | 82.7 | 10.7 | 4.1 | 2.5 | 93.4 | 67.14 | WARNING | PHYRE2 |
| HP0508 | 72.0 | 19.5 | 4.1 | 4.3 | 91.5 | 20.75 | FAIL | PHYRE2 |
| HP0509 | 84.0 | 11.7 | 2.5 | 1.7 | 95.7 | 71.52 | WARNING | PHYRE2 |
| HP0510 | 89.8 | 8.0 | 1.3 | 0.9 | 97.8 | 85.49 | PASS | MODBASE |
| HP0511 | 87.5 | 12.5 | 0.0 | 0.0 | 100 | 0.0 | FAIL | PHYRE2 |
| HP0512 | 90.1 | 9.7 | 0.0 | 0.2 | 99.8 | 87.08 | PASS | MODBASE |
| HP0513 | 71.2 | 21.2 | 3.2 | 4.4 | 92.4 | 18.51 | FAIL | PHYRE2 |
| HP0514 | 87.4 | 10.4 | 2.2 | 0.0 | 97.8 | 65.33 | WARNING | MODBASE |
| HP0515 | 90.5 | 8.2 | 0.6 | 0.6 | 98.7 | 74.58 | WARNING | MODBASE |
| HP0516 | 91.6 | 5.7 | 1.6 | 1.1 | 97.3 | 72.95 | WARNING | MODBASE |
| HP0517 | 83.6 | 12.1 | 1.8 | 2.5 | 95.7 | 70.53 | WARNING | PHYRE2 |
| HP0518 | 69.7 | 19.0 | 4.3 | 7.0 | 88.7 | 47.73 | FAIL | PHYRE2 |
| HP0519 | 87.4 | 9.3 | 2.4 | 0.8 | 96.7 | 53.07 | FAIL | PHYRE2 |
| HP0520 | 73.8 | 23.3 | 1.0 | 1.9 | 97.1 | 14.66 | FAIL | PHYRE2 |
| HP0521 | 85.7 | 14.3 | 0.0 | 0.0 | 100 | 44.44 | FAIL | PHYRE2 |
| HP0522 | 71.2 | 19.8 | 4.5 | 4.5 | 91 | 23.24 | FAIL | PHYRE2 |
| HP0523 | 85.7 | 11.0 | 2.2 | 1.1 | 2.2 | 43.56 | FAIL | PHYRE2 |
| HP0524 | 73.4 | 19.3 | 3.7 | 3.6 | 92.7 | 58.08 | FAIL | PHYRE2 |
| HP0525 | 91.2 | 8.1 | 0.7 | 0.0 | 99.3 | 92.77 | PASS | PDB |
| HP0526 | 93.4 | 6.6 | 0.0 | 0.0 | 100 | 92.09 | PASS | PDB |
| HP0527 |  |  |  |  | TOO LONG |  |  | PHYRE2 |
| HP0528 | 69.4 | 22.6 | 4.9 | 3.1 | 92 | 16.63 | FAIL | PHYRE2 |
| HP0529 | 81.9 | 12.8 | 2.1 | 3.2 | 94.7 | 34.89 | FAIL | PHYRE2 |
| HP0530 | 81.5 | 12.6 | 4.2 | 1.7 | 94.1 | 28.85 | FAIL | PHYRE2 |
| HP0531 | 82.4 | 11.2 | 1.6 | 4.8 | 93.6 | 14.61 | FAIL | PHYRE2 |
| HP0532 | 69.9 | 23.9 | 3.1 | 3.1 | 93.8 | 30.25 | FAIL | PHYRE2 |
| HP0533 | SMALL (29) |  |  |  |  |  |  |  |
| HP0534 | 94.0 | 6.0 | 0.0 | 0.0 | 100 | 86.27 | PASS | PDB |
| HP0535 | 100 | 0.0 | 0.0 | 0.0 | 100 | 0.0 | FAIL | PHYRE2 |
| HP0536 | SMALL |  |  |  |  |  |  |  |
| HP0537 | 80.7 | 14.4 | 2.8 | 2.2 | 95.1 | 2.39 | FAIL | PHYRE2 |
| HP0538 | 72.2 | 22.2 | 1.8 | 3.9 | 94.4 | 20.85 | FAIL | PHYRE2 |
| HP0539 | 98.3 | 0.6 | 1.1 | 0.0 | 94. | 60.43 | FAIL | PDB |
| HP0540 | 68.8 | 20.6 | 6.9 | 3.7 | 89.4 | 24.08 | FAIL | PHYRE2 |
| HP0541 | 80.2 | 13.5 | 2.4 | 3.9 | 93.7 | 0 | FAIL | PHYRE2 |
| HP0542 | 65.5 | 22.4 | 3.4 | 8.6 | 91.4 | 4.5 | FAIL | PHYRE2 |
| HP0543 | 88.7 | 7.0 | 1.9 | 2.3 | 95.7 | 49.81 | FAIL | ITASSER |
| HP0544 | 68.1 | 20.3 | 5.8 | 5.9 | 88.4 | 31.91 | FAIL | PHYRE2 |
| HP0545 | 82.9 | 14.8 | 2.3 | 0.0 | 100 | 98.37 | FAIL | PDB |
| HP0546 | 73.2 | 18.6 | 0.0 | 8.2 | 91.8 | 29.31 | FAIL | PHYRE2 |
| HP0547 | 94.8 | 5.2 | 0.0 | 0.0 | 100 | 95.56 | FAIL | PDB |
| HP0548 |  |  |  |  | NO PTN SEQ |  |  |  |
| HP0549 | 88.9 | 10.7 | 0.5 | 0.0 | 100 | 100 | PASS | PDB |
| HP0550 | 87.5 | 7.9 | 2.5 | 2.2 | 95.4 | 88.28 | PASS | MODBASE |
| HP0551 | 27.3 | 43.6 | 10.9 | 18.2 | 94. | 0.0 | FAIL | PHYRE2 |
| HP0552 | 80.8 | 13.5 | 3.8 | 1.9 | 94.3 | 46.18 | FAIL | PHYRE2 |
| HP0553 | 89.1 | 8.5 | 0.5 | 2.0 | 97.6 | 85.90 | PASS | MODBASE |
| HP0554 | 63.6 | 23.9 | 6.7 | 5.7 | 87.5 | 29.19 | FAIL | PHYRE2 |
| HP0555 | 76.1 | 18.7 | 3.6 | 1.6 | 94.8 | 16.36 | FAIL | PHYRE2 |
| HP0556 | 66.7 | 33.3 | 0.0 | 0.0 | 100 | 0.0 | FAIL | PHYRE2 |
| HP0557 | 90.2 | 6.8 | 2.6 | 0.4 | 97 | 72.04 | WARNING | MODBASE |
| HP0558 | 87.8 | 10.5 | 0.3 | 1.5 | 98.3 | 87.41 | PASS | MODBASE |
| HP0559 | 94.0 | 6.0 | 0.0 | 0.0 | 100 | 86.67 | PASS | MODBASE |
| HP0560 | SMALL 26 |  |  |  |  |  |  |  |
| HP0561 | 92.3 | 7.7 | 0.0 | 0.0 | 100 | 100 | PASS | PDB |
| HP0562 | 72.3 | 21.5 | 4.6 | 1.5 | 98.5 | 73.24 | FAIL | PHYRE2 |
| HP0563 | 68.0 | 24.0 | 4.7 | 3.3 | 92 | 22.54 | FAIL | PHYRE2 |
| HP0564 | 84.3 | 15.7 | 0.0 | 0.0 | 100 | 0.0 | FAIL | PDB |
| HP0565 | 84.2 | 11.4 | 2.7 | 1.6 | 95.6 | 2.31 | FAIL | PHYRE2 |
| HP0566 | 85.2 | 10.3 | 2.2 | 2.2 | 95.5 | 95.63 | PASS | MODBASE |
| HP0567 | 83.1 | 12.4 | 2.2 | 2.2 | 95.5 | 28.08 | FAIL | PHYRE2 |
| HP0568 | 82.5 | 15.0 | 1.7 | 0.9 | 97.5 | 48.05 | FAIL | PHYRE2 |
| HP0569 | 92.2 | 6.6 | 0.9 | 0.3 | 98.8 | 81.20 | PASS | MODBASE |
| HP0570 | 86.5 | 11.7 | 1.6 | 0.2 | 98.2 | 84.48 | PASS | MODBASE |
| HP0571 | 88.1 | 8.9 | 3.0 | 0.0 | 100 | 21.05 | FAIL | PHYRE2 |
| HP0572 | 91.7 | 7.0 | 0.6 | 0.6 | 98.7 | 92.74 | PASS | MODBASE |
| HP0573 | 100 | 0.0 | 0.0 | 0.0 | 99 | 0.0 | FAIL | PHYRE2 |
| HP0574 | 95.2 | 3.2 | 1.6 | 0.0 | 98.4 | 84.51 | PASS | MODBASE |
| HP0575 | 78.6 | 12.6 | 4.4 | 4.4 | 91.2 | 10.73 | FAIL | PHYRE2 |
| HP0576 | 78.7 | 16.1 | 3.9 | 1.2 | 94.8 | 25.43 | FAIL | PHYRE2 |
| HP0577 | 91.9 | 7.7 | 0.0 | 0.4 | 99.6 | 91.55 | PASS | MODBASE |
| HP0578 | 71.8 | 17.7 | 5.6 | 4.9 | 89.5 | 45.36 | FAIL | PHYRE2 |
| HP0579 | 94.2 | 4.7 | 0.6 | 0.6 | 98.9 | 8.6 | FAIL | PHYRE2 |
| HP0580 | 74.6 | 18.0 | 3.6 | 3.8 | 92.6 | 63.81 | FAIL | PHYRE2 |
| HP0581 | 89.3 | 8.7 | 1.3 | 0.7 | 98 | 88.10 | PASS | MODBASE |
| HP0582 | 60.3 | 28.1 | 4.9 | 6.7 | 88.4 | 50.15 | FAIL | PHYRE2 |
| HP0583 | 59.6 | 30.0 | 4.9 | 5.6 | 89.6 | 10.54 | FAIL | PHYRE2 |
| HP0584 | 93.2 | 5.4 | 0.0 | 1.4 | 98.6 | 21.18 | FAIL | PHYRE2 |
| HP0585 | 92.2 | 7.3 | 0.0 | 0.5 | 99.5 | 85.10 | PASS | MODBASE |
| HP0586 | 67.3 | 25.3 | 5.7 | 1.7 | 92.6 | 24.54 | FAIL | PHYRE2 |
| HP0587 | 74.9 | 18.6 | 2.7 | 3.7 | 93.5 | 42.73 | FAIL | PHYRE2 |
| HP0588 | 83.1 | 12.0 | 4.8 | 0.0 | 100 | 9.28 | FAIL | PHYRE2 |
| HP0589 | 85.3 | 9.7 | 3.1 | 1.9 | 95 | 59.04 | FAIL | PHYRE2 |
| HP0590 | 78.2 | 16.3 | 3.3 | 2.1 | 94.5 | 68.61 | WARNING | PHYRE2 |
| HP0591 | 94.4 | 5.0 | 0.0 | 0.6 | 99.4 | 77.17 | WARNING | PHYRE2 |
| HP0592 | 84.0 | 14.0 | 1.5 | 0.5 | 98 | 48.36 | FAIL | PHYRE2 |
| HP0593 | 60.5 | 24.0 | 7.6 | 7.9 | 84.5 | 40.23 | FAIL | PHYRE2 |
| HP0594 | 88.9 | 11.1 | 0.0 | 0.0 | 100 | 0.0 | FAIL | PHYRE2 |
| HP0595 | 62.1 | 31.0 | 4.3 | 2.6 | 97.4 | 8.09 | FAIL | PHYRE2 |
| HP0596 | 91.5 | 5.1 | 1.1 | 2.3 | 96.6 | 87.92 | PASS | PDB |
| HP0597 | 79.9 | 13.9 | 4.4 | 1.9 | 93.8 | 67.12 | WARNING | PHYRE2 |
| HP0598 | 88.8 | 8.8 | 1.5 | 0.9 | 99.1 | 75.41 | WARNING | PHYRE2 |
| HP0599 | 89.7 | 5.9 | 2.5 | 2.0 | 95.6 | 24.42 | FAIL | PHYRE2 |
| HP0600 | 84.5 | 9.8 | 3.5 | 2.2 | 94.3 | 44.75 | FAIL | PHYRE2 |
| HP0601 | 83.4 | 11.2 | 2.8 | 2.6 | 94.6 | 34.25 | FAIL | PHYRE2 |
| HP0602 | 92.2 | 6.4 | 0.5 | 1.0 | 98.6 | 100 | PASS | PDB |
| HP0603 | 77.1 | 20.0 | 1.4 | 1.4 | 98.6 | 23.26 | FAIL | PHYRE2 |
| HP0604 | 92.9 | 5.7 | 1.0 | 0.3 | 98.6 | 99.11 | PASS | PHYRE2 |
| HP0605 | 86.0 | 9.8 | 3.1 | 1.1 | 95.8 | 29.92 | FAIL | PHYRE2 |
| HP0606 | 89.7 | 7.5 | 1.9 | 0.9 | 97.2 | 61.70 | FAIL | PHYRE2 |
| HP0607 | 91.7 | 6.4 | 1.0 | 1.0 | 98.1 | 73.66 | WARNING | MODBASE |
| HP0608 | 74.0 | 19.8 | 4.6 | 1.5 | 98.5 | 17.61 | FAIL | PHYRE2 |
| HP0609 | 74.2 | 22.6 | 0.0 | 3.2 | 96.8 | 0.00 | FAIL | PHYRE2 |
| HP0610 | TOO LONG |  |  |  | 0 |  |  |  |
| HP0611 | SMALL |  |  |  |  |  |  |  |
| HP0612 | 92.2 | 5.9 | 2.0 | 0.0 | 100 | 0.0 | FAIL | PHYRE2 |
| HP0613 | 85.1 | 11.0 | 1.5 | 1.5 | 96.1 | 69.47 | WARNING | MODBASE |
| HP0614 | 94.3 | 5.7 | 0.0 | 0.0 | 100 | 25 | FAIL | PHYRE2 |
| HP0615 | 84.5 | 12.1 | 2.4 | 1.0 | 96.6 | 74.43 | WARNING | PHYRE2 |
| HP0616 | 74.2 | 17.5 | 4.1 | 4.1 | 91.7 | 69.11 | WARNING | PHYRE2 |
| HP0617 | 91.4 | 7.8 | 0.4 | 0.4 | 99.2 | 91.70 | PASS | MODBASE |
| HP0618 | 93.5 | 6.0 | 0.6 | 0.0 | 99.5 | 75.13 | WARNING | MODBASE |
| HP0619 |  |  |  |  | NO PTN SEQ |  |  |  |
| HP0620 | 90.6 | 7.4 | 2.0 | 0.0 | 100 | 94.01 | PASS | PDB |
| HP0621 | 78.0 | 13.7 | 4.7 | 3.6 | 91.7 | 57.27 | FAIL | PHYRE2 |
| HP0622 | 100 | 0.0 | 0.0 | 0.0 | 100 | 0.0 | FAIL | PHYRE2 |
| HP0623 | 89.6 | 8.6 | 1.0 | 0.8 | 98.2 | 76.21 | WARNING | MODBASE |
| HP0624 | 91.6 | 7.7 | 0.6 | 0.2 | 99.3 | 95.29 | PASS | PDB |
| HP0625 | 88.8 | 9.4 | 0.6 | 1.2 | 98.2 | 85.28 | PASS | PHYRE2 |
| HP0626 | 89.3 | 8.6 | 0.3 | 1.7 | 97.9 | 80.05 | PASS | MODBASE |
| HP0627 | 94.1 | 5.9 | 5.5 | 2.2 | 92.3 | 95.24 | PASS | PHYRE2 |
| HP0628 | 93.1 | 6.9 | 0.0 | 0.0 | 100 | 89.59 | PASS | MODBASE |
| HP0629 | 69.6 | 20.0 | 4.6 | 5.1 | 89.6 | 42.23 | FAIL | PHYRE2 |
| HP0630 | 89.7 | 7.9 | 1.2 | 1.2 | 97.6 | 97.93 | PASS | MODBASE |
| HP0631 | 76.4 | 16.0 | 6.0 | 1.5 | 92.4 | 93.58 | PASS | MODBASE |
| HP0632 | 88.9 | 8.8 | 1.8 | 0.4 | 99.6 | 90.83 | PASS | MODBASE |
| HP0633 | 80.0 | 10.5 | 2.5 | 7.0 | 90.5 | 37.33 | FAIL | PHYRE2 |
| HP0634 | 94.3 | 3.6 | 1.4 | 0.7 | 99.3 | 90.74 | PASS | PHYRE2 |
| HP0635 | 63.1 | 22.2 | 6.4 | 8.3 | 85.3 | 45.22 | FAIL | PHYRE2 |
| HP0636 | 87.1 | 9.0 | 3.2 | 0.7 | 99.3 | 0.0 | FAIL | PHYRE2 |
| HP0637 | 50.0 | 30.3 | 16.7 | 0.0 | 100 | 0.0 | FAIL | PHYRE2 |
| HP0638 | 69.3 | 22.7 | 7.2 | 0.8 | 92 | 28.43 | FAIL | PHYRE2 |
| HP0639 | 85.1 | 10.9 | 2.5 | 1.5 | 96 | 76.00 | WARNING | MODBASE |
| HP0640 | 86.9 | 9.3 | 2.7 | 1.1 | 96.2 | 74.94 | WARNING | PHYRE2 |
| HP0641 | 90.2 | 7.8 | 2.0 | 0.0 | 100 | 0.0 | FAIL | PHYRE2 |
| HP0642 | 93.4 | 6.6 | 0.0 | 0.0 | 100 | 84.04 | PASS | PDB |
| HP0643 | 88.4 | 9.2 | 1.2 | 1.2 | 97.6 | 84.28 | PASS | MODBASE |
| HP0644 | 90.9 | 0.0 | 9.1 | 0.0 | 100 | 0.0 | FAIL | PHYRE2 |
| HP0645 | 81.7 | 14.4 | 2.1 | 1.7 | 96.1 | 47.77 | FAIL | PHYRE2 |
| HP0646 | 88.3 | 10.9 | 0.6 | 0.1 | 99.9 | 99.81 | PASS | PDB |
| HP0647 | 92.6 | 7.4 | 0.0 | 0.0 | 100 | 0.0 | FAIL | PHYRE2 |
| HP0648 | 92.5 | 6.7 | 0.5 | 0.3 | 99.2 | 94.33 | PASS | MODBASE |
| HP0649 | 89.2 | 8.4 | 1.5 | 1.0 | 97.6 | 68.50 | WARNING | MODBASE |
| HP0650 | 81.4 | 16.1 | 1.2 | 1.2 | 98.8 | 75.29 | WARNING | PHYRE2 |
| HP0651 | 87.4 | 12.2 | 0.3 | 0.0 | 100 | 91.63 | PASS | PDB |
| HP0652 | 90.0 | 9.5 | 0.5 | 0.0 | 99.5 | 92.51 | PASS | PDB |
| HP0653 | 94.5 | 5.0 | 0.4 | 0.0 | 100 | 91.64 | PASS | PDB |
| HP0654 | 77.2 | 16.1 | 3.6 | 3.0 | 93.3 | 59.56 | FAIL | PHYRE2 |
| HP0655 | 74.7 | 17.9 | 5.0 | 2.4 | 92.6 | 65.32 | WARNING | PHYRE2 |
| HP0656 | 77.9 | 14.0 | 4.9 | 3.2 | 91.9 | 64.58 | FAIL | PHYRE2 |
| HP0657 | 92.2 | 6.5 | 0.8 | 0.5 | 98.7 | 70.21 | WARNING | PHYRE2 |
| HP0658 | 88.8 | 9.8 | 0.9 | 0.5 | 98.6 | 75.37 | WARNING | PHYRE2 |
| HP0659 | 73.4 | 19.8 | 2.9 | 4.0 | 93.2 | 33.01 | FAIL | PHYRE2 |
| HP0660 | 70.6 | 19.2 | 5.0 | 5.3 | 89.8 | 27.14 | FAIL | PHYRE2 |
| HP0661 | 92.2 | 7.0 | 0.8 | 0.0 | 99.2 | 86.81 | PASS | MODBASE |
| HP0662 | 91.8 | 7.7 | 0.5 | 0.0 | 99.5 | 78.76 | WARNING | MODBASE |
| HP0663 | 83.0 | 14.0 | 1.6 | 1.4 | 98.6 | 0 | FAIL | PDB |
| HP0664 | 79.7 | 18.6 | 0.0 | 1.7 | 98.3 | 0.0 | FAIL | PHYRE2 |
| HP0665 | 93.3 | 5.7 | 0.7 | 0.2 | 99 | 89.24 | PASS | MODBASE |
| HP0666 | 63.1 | 20.1 | 8.1 | 8.7 | 83.2 | 43.09 | FAIL | PHYRE2 |
| HP0667 | 92.6 | 7.4 | 0.0 | 0.0 | 100 | 0.0 | FAIL | PHYRE2 |
| HP0668 | 73.1 | 19.8 | 5.3 | 1.8 | 92.9 | 43.26 | FAIL | PHYRE2 |
| HP0669 | 71.5 | 20.4 | 5.5 | 2.6 | 91.9 | 44.0 | FAIL | PHYRE2 |
| HP0670 | 61.0 | 29.2 | 4.8 | 5.0 | 90.2 | 17.56 | FAIL | PHYRE2 |
| HP0671 | 66.8 | 26.2 | 4.8 | 2.2 | 93 | 50.55 | FAIL | PHYRE2 |
| HP0672 | 94.2 | 4.9 | 0.6 | 0.3 | 99.1 | 89.97 | PASS | MODBASE |
| HP0673 | 71.5 | 21.3 | 5.9 | 1.3 | 92.8 | 14.99 | FAIL | PHYRE2 |
| HP0674 | 71.0 | 22.4 | 4.8 | 1.9 | 93.4 | 6.99 | FAIL | PHYRE2 |
| HP0675 | 66.4 | 18.0 | 6.2 | 9.4 | 84.4 | 34.71 | FAIL | PHYRE2 |
| HP0676 | 88.4 | 8.7 | 2.2 | 0.7 | 97.1 | 86.79 | PASS | MODBASE |
| HP0677 | 79.2 | 13.4 | 2.3 | 5.1 | 92.6 | 47.66 | FAIL | PHYRE2 |
| HP0678 | 92.6 | 7.4 | 0.0 | 0.0 | 100 | 0.0 | FAIL | PHYRE2 |
| HP0679 | 89.1 | 9.7 | 0.0 | 1.2 | 98.8 | 97.93 | PASS | PHYRE2 |
| HP0680 | 85.6 | 11.0 | 2.4 | 1.0 | 96.6 | 75.36 | WARNING | PHYRE2 |
| HP0681 | 87.5 | 12.5 | 0.0 | 0.0 | 100 | 0.0 | FAIL | PHYRE2 |
| HP0682 | 87.5 | 12.5 | 0.0 | 0.0 | 100 | 0.0 | FAIL | PHYRE2 |
| HP0683 | 85.3 | 11.8 | 2.1 | 0.8 | 97.1 | 75.41 | WARNING | MODBASE |
| HP0684 | NO ID |  |  |  |  |  |  |  |
| HP0685 | 78.7 | 18.0 | 3.3 | 0.0 | 100 | 0.0 | FAIL | PHYRE2 |
| HP0686 | 74.9 | 18.1 | 4.5 | 2.5 | 93 | 56.12 | FAIL | PHYRE2 |
| HP0687 | 73.7 | 18.1 | 3.6 | 4.5 | 91.8 | 34.06 | FAIL | PHYRE2 |
| HP0688 | 86.2 | 11.7 | 1.4 | 0.7 | 99.3 | 33.96 | FAIL | PHYRE2 |
| HP0689 | 85.1 | 12.8 | 2.1 | 0.0 | 100 | 0.0 | FAIL | PHYRE2 |
| HP0690 | 93.5 | 6.2 | 0.0 | 0.3 | 99.7 | 80.56 | PASS | MODBASE |
| HP0691 | 96.4 | 2.6 | 0.5 | 0.5 | 99 | 98.12 | PASS | PDB |
| HP0692 | 93.2 | 6.2 | 0.6 | 0.0 | 99.4 | 98.12 | PASS | PDB |
| HP0693 | 79.8 | 14.6 | 2.8 | 2.8 | 94.4 | 44.40 | FAIL | PHYRE2 |
| HP0694 | 80.9 | 11.6 | 3.6 | 4.0 | 92.5 | 25.97 | FAIL | PHYRE2 |
| HP0695 | 62.1 | 23.9 | 6.4 | 7.5 | 86 | 44.12 | FAIL | PHYRE2 |
| HP0696 | 55.8 | 26.7 | 8.2 | 9.3 | 82.5 | 35.51 | FAIL | PHYRE2 |
| HP0697 | 69.2 | 20.5 | 2.6 | 7.7 | 92.3 | 32.61 | FAIL | PHYRE2 |
| HP0698 | SMALL 29 |  |  |  |  |  |  |  |
| HP0699 | 70.4 | 21.5 | 3.7 | 4.4 | 91.9 | 13.99 | FAIL | PHYRE2 |
| HP0700 | 78.7 | 15.7 | 4.6 | 0.9 | 99.1 | 0.0 | FAIL | PHYRE2 |
| HP0701 | 82.9 | 12.7 | 2.8 | 1.6 | 95.6 | 87.31 | PASS | MODBASE |
| HP0702 | 100 | 0.0 | 0.0 | 0.0 | 100 | 0.0 | FAIL | PHYRE2 |
| HP0703 | 93.6 | 5.2 | 0.9 | 0.3 | 98.8 | 84.13 | PASS | MODBASE |
| HP0704 | 59.4 | 31.2 | 3.1 | 6.2 | 90.6 | 8.11 | FAIL | PHYRE2 |
| HP0705 | 89.5 | 8.7 | 0.8 | 1.0 | 98.2 | 77.09 | WARNING | PHYRE2 |
| HP0706 | 70.9 | 18.5 | 6.2 | 4.4 | 89.4 | 42.34 | FAIL | PHYRE2 |
| HP0707 | 90.7 | 7.1 | 1.1 | 1.1 | 97.8 | 76.47 | WARNING | MODBASE |
| HP0708 | 100 | 0.0 | 0.0 | 0.0 | 100 | 0.0 | FAIL | PHYRE2 |
| HP0709 | 89.7 | 2.6 | 7.3 | 0.4 | 92.3 | 81.18 | PASS | MODBASE |
| HP0710 | 71.1 | 20.7 | 5.3 | 2.9 | 91.8 | 25.42 | FAIL | PHYRE2 |
| HP0711 | 79.2 | 13.3 | 5.6 | 1.9 | 92.5 | 28.26 | FAIL | PHYRE2 |
| HP0712 | 87.8 | 11.1 | 1.2 | 0.0 | 100 | 8.85 | FAIL | PHYRE2 |
| HP0713 | NO ID |  |  |  |  |  |  |  |
| HP0714 | 71.6 | 18.3 | 3.9 | 6.2 | 89.9 | 34.22 | FAIL | PHYRE2 |
| HP0715 | 87.9 | 8.7 | 2.4 | 1.0 | 96.6 | 91.56 | PASS | MODBASE |
| HP0716 | 83.2 | 14.3 | 0.0 | 2.5 | 97.5 | 83.21 | PASS | PHYRE2 |
| HP0717 | 78.1 | 14.8 | 3.1 | 3.9 | 92.9 | 44.39 | FAIL | PHYRE2 |
| HP0718 | 88.0 | 6.3 | 1.6 | 4.2 | 94.3 | 6.64 | FAIL | PHYRE2 |
| HP0719 | 95.1 | 4.9 | 0.0 | 0.0 | 100 | 50.75 | FAIL | PHYRE2 |
| HP0720 | 76.9 | 23.1 | 0.0 | 0.0 | 100 | 45.16 | FAIL | PHYRE2 |
| HP0721 | 93.5 | 5.4 | 0.0 | 1.1 | 98.9 | 89.11 | PASS | PDB |
| HP0722 | NO ID 77.4 | 18.4 | 1.8 | 2.4 | 95.8 | 58.95 | FAIL | I-TASSER |
| HP0723 | 91.3 | 8.4 | 0.0 | 0.3 | 99.7 | 99.69 | PASS | PDB |
| HP0724 | 73.4 | 18.7 | 3.3 | 4.6 | 92.1 | 27.70 | FAIL | PHYRE2 |
| HP0725 | 92.9 | 6.8 | 0.3 | 0.0 | 100 | 96.77 | PASS | PDB |
| HP0726 | 74.9 | 18.1 | 4.6 | 2.3 | 93 | 38.56 | FAIL | PHYRE2 |
| HP0727 | 88.4 | 9.8 | 1.1 | 0.7 | 98.2 | 90.46 | PASS | MODBASE |
| HP0728 | 80.5 | 14.1 | 4.5 | 1.0 | 94.6 | 55.49 | FAIL | PHYRE2 |
| HP0729 | 81.6 | 13.6 | 1.8 | 3.0 | 95.2 | 21.63 | FAIL | PHYRE2 |
| HP0730 | 89.8 | 6.1 | 2.0 | 2.0 | 98 | 98.31 | PASS | PHYRE2 |
| HP0731 | 79.2 | 13.8 | 3.2 | 3.8 | 93 | 39.20 | FAIL | PHYRE2 |
| HP0732 | 93.6 | 6.4 | 0.0 | 0.0 | 100 | 0.0 | FAIL | PHYRE2 |
| HP0733 | 85.7 | 11.4 | 1.0 | 1.9 | 97.1 | 35.44 | FAIL | PHYRE2 |
| HP0734 | 64.5 | 23.9 | 7.6 | 3.9 | 88.4 | 57.27 | FAIL | PHYRE2 |
| HP0735 | 88.4 | 9.4 | 1.4 | 0.7 | 99.3 | 69.33 | WARNING | PHYRE2 |
| HP0736 | 86.1 | 10.5 | 2.4 | 1.0 | 96.6 | 69.16 | WARNING | MODBASE |
| HP0737 | 85.9 | 10.9 | 3.1 | 0.0 | 100 | 5.19 | FAIL | PHYRE2 |
| HP0738 | 88.3 | 8.7 | 2.0 | 1.0 | 97 | 96.28 | PASS | PDB |
| HP0739 | 84.5 | 12.3 | 1.4 | 1.8 | 96.8 | 78.93 | WARNING | PHYRE2 |
| HP0740 | 73.8 | 18.0 | 3.3 | 4.9 | 91.8 | 48.38 | FAIL | PHYRE2 |
| HP0741 | 67.6 | 23.2 | 7.7 | 1.4 | 90.8 | 77.78 | WARNING | PHYRE2 |
| HP0742 | 87.9 | 11.0 | 0.7 | 0.4 | 98.9 | 81.11 | PASS | MODBASE |
| HP0743 | 83.7 | 10.8 | 1.8 | 3.6 | 94.5 | 31.94 | FAIL | PHYRE2 |
| HP0744 | 89.9 | 8.2 | 1.5 | 0.4 | 99.6 | 31.91 | FAIL | PHYRE2 |
| HP0745 | 86.6 | 11.2 | 1.1 | 1.1 | 97.8 | 70.16 | WARNING | PHYRE2 |
| HP0746 | 70.5 | 18.0 | 6.8 | 4.7 | 88.5 | 32.38 | FAIL | PHYRE2 |
| HP0747 | 75.0 | 16.1 | 6.9 | 1.9 | 91.1 | 50.25 | FAIL | PHYRE2 |
| HP0748 | 89.8 | 7.7 | 2.0 | 0.5 | 97.5 | 72.15 | WARNING | MODBASE |
| HP0749 | 88.4 | 6.8 | 1.6 | 3.2 | 95.2 | 4.46 | FAIL | PHYRE2 |
| HP0750 | 85.5 | 9.2 | 3.2 | 2.1 | 94.7 | 7.98 | FAIL | PHYRE2 |
| HP0751 | 78.3 | 20.7 | 0.0 | 1.1 | 98.9 | 59.62 | FAIL | PHYRE2 |
| HP0752 | 55.0 | 31.7 | 7.1 | 6.1 | 86.7 | 30.03 | FAIL | PHYRE2 |
| HP0753 | 95.1 | 4.9 | 0.0 | 0.0 | 100 | 76.92 | WARNING | PDB |
| HP0754 | 90.9 | 6.1 | 3.0 | 0.0 | 100 | 0.0 | FAIL | PHYRE2 |
| HP0755 | 80.1 | 16.1 | 2.7 | 1.1 | 96.2 | 53.55 | FAIL | PHYRE2 |
| HP0756 | 50.0 | 28.6 | 21.4 | 0.0 | 100 | 0.0 | FAIL | PHYRE2 |
| HP0757 | 87.2 | 7.4 | 3.5 | 1.9 | 94.6 | 74.40 | WARNING | MODBASE |
| HP0758 | 85.2 | 9.9 | 3.6 | 1.3 | 95.1 | 46.12 | FAIL | PHYRE2 |
| HP0759 | 79.6 | 15.6 | 3.2 | 1.6 | 95.2 | 38.52 | FAIL | PHYRE2 |
| HP0760 | 79.2 | 11.9 | 3.0 | 5.9 | 91.1 | 31.89 | FAIL | PHYRE2 |
| HP0761 | 66.1 | 23.3 | 5.0 | 5.6 | 89.4 | 49.25 | FAIL | PHYRE2 |
| HP0762 | 81.2 | 15.6 | 3.1 | 0.0 | 100 | 37.84 | FAIL | PHYRE2 |
| HP0763 | 91.9 | 7.3 | 0.0 | 0.8 | 99.2 | 95.16 | PASS | MODBASE |
| HP0764 | 73.1 | 18.9 | 2.6 | 5.4 | 92 | 14.92 | FAIL | PHYRE2 |
| HP0765 | 89.5 | 10.5 | 0.0 | 0.0 | 100 | 0.0 | FAIL | PHYRE2 |
| HP0766 | 75.3 | 16.6 | 3.6 | 4.5 | 91.9 | 16.73 | FAIL | PHYRE2 |
| HP0767 | SMALL 24 |  |  |  |  |  |  |  |
| HP0768 | 88.6 | 9.3 | 2.1 | 0.0 | 97.9 | 78.66 | WARNING | MODBASE |
| HP0769 | 81.7 | 13.4 | 2.2 | 2.7 | 95.1 | 66.34 | WARNING | PHYRE2 |
| HP0770 | 79.0 | 13.7 | 3.0 | 4.3 | 92.7 | 25.63 | FAIL | PHYRE2 |
| HP0771 | 92.6 | 6.5 | 0.4 | 0.4 | 99.1 | 0.00 | FAIL | PHYRE2 |
| HP0772 | 67.0 | 22.3 | 6.2 | 4.5 | 89.3 | 39.23 | FAIL | PHYRE2 |
| HP0773 | 86.8 | 10.3 | 2.0 | 1.0 | 97.1 | 78.30 | WARNING | PHYRE2 |
| HP0774 | 93.3 | 5.8 | 0.3 | 0.6 | 99.1 | 71.14 | WARNING | MODBASE |
| HP0775 | 74.1 | 18.1 | 4.2 | 3.6 | 92.2 | 48.84 | FAIL | PHYRE2 |
| HP0776 | 60.4 | 34.0 | 1.9 | 3.8 | 96.2 | 3.28 | FAIL | PHYRE2 |
| HP0777 | 95.0 | 5.0 | 0.0 | 0.0 | 100 | 98.68 | PASS | PDB |
| HP0778 | 80.1 | 13.6 | 2.4 | 3.9 | 93.7 | 72.81 | WARNING | PHYRE2 |
| HP0779 | 91.1 | 7.8 | 1.1 | 0.0 | 98.9 | 94.69 | PASS | MODBASE |
| HP0780 | 87.8 | 12.2 | 0.0 | 0.0 | 100 | 47.83 | FAIL | PHYRE2 |
| HP0781 | 69.4 | 20.0 | 3.3 | 7.3 | 89.4 | 41.40 | FAIL | PHYRE2 |
| HP0782 | 75.9 | 15.3 | 6.0 | 2.8 | 91.2 | 63.16 | FAIL | PHYRE2 |
| HP0783 | 100 | 0.0 | 0.0 | 0.0 | 100 | 0.0 | FAIL | PHYRE2 |
| HP0784 | SMALL |  |  |  |  |  |  |  |
| HP0785 | 87.2 | 11.4 | 1.3 | 0.0 | 100 | 81.71 | PASS | PHYRE2 |
| HP0786 | 85.4 | 10.8 | 1.9 | 1.9 | 96.2 | 68.82 | WARNING | MODBASE |
| HP0787 | 72.4 | 18.3 | 4.4 | 4.9 | 90.7 | 39.90 | FAIL | PHYRE2 |
| HP0788 | 59.6 | 25.1 | 7.0 | 8.4 | 84.7 | 37.00 | FAIL | PHYRE2 |
| HP0789 | 84.2 | 11.5 | 2.2 | 2.2 | 95.7 | 73.58 | WARNING | PHYRE2 |
| HP0790 | 82.9 | 10.6 | 4.7 | 1.8 | 93.5 | 50.46 | FAIL | PHYRE2 |
| HP0791 | 81.4 | 13.4 | 3.1 | 2.1 | 94.8 | 64.48 | FAIL | PHYRE2 |
| HP0792 | 76.6 | 16.4 | 5.0 | 2.0 | 93 | 59.63 | FAIL | PHYRE2 |
| HP0793 | 89.3 | 10.0 | 0.7 | 0.0 | 100 | 76.05 | WARNING | PDB |
| HP0794 | 89.4 | 9.6 | 0.9 | 0.1 | 99.9 | 0 | FAIL | PDB |
| HP0795 | 79.6 | 14.3 | 2.6 | 3.6 | 93.9 | 51.99 | FAIL | PHYRE2 |
| HP0796 | 74.3 | 18.1 | 5.6 | 2.0 | 92.4 | 21.86 | FAIL | PHYRE2 |
| HP0797 | 70.8 | 19.9 | 5.5 | 3.8 | 90.7 | 43.30 | FAIL | PHYRE2 |
| HP0798 | 80.6 | 10.8 | 5.0 | 3.6 | 91.4 | 82.17 | PASS | MOD |
| HP0799 | 94.7 | 4.6 | 0.7 | 0.0 | 99.3 | 81.82 | PASS | MODBASE |
| HP0800 | 92.5 | 7.2 | 0.3 | 0.0 | 99.7 | 100.00 | PASS | PDB |
| HP0801 | 92.5 | 7.2 | 0.3 | 0.0 | 99.7 | 100 | PASS | PDB |
| HP0802 | 90.6 | 7.4 | 1.3 | 0.7 | 98.0 | 77.19 | WARNING | MODBASE |
| HP0803 | 69.1 | 25.9 | 2.5 | 2.5 | 95.0 | 0 | FAIL | PHYRE2 |
| HP0804 | 88.5 | 8.2 | 2.7 | 0.5 | 96.7 | 96.10 | PASS | MODBASE |
| HP0805 | 85.8 | 11.9 | 2.3 | 0.0 | 97.7 | 52.7 | FAIL | PHYRE2 |
| HP0806 | 85.3 | 13.2 | 1.2 | 0.3 | 98.5 | 66.76 | WARNING | PHYRE2 |
| HP0807 | 85.6 | 12.9 | 1.3 | 0.2 | 98.5 | 64.06 | FAIL | PHYRE2 |
| HP0808 | 91.4 | 5.7 | 1.9 | 1.0 | 97.1 | 79.13 | WARNING | MODBASE |
| HP0809 | 83.3 | 16.7 | 0.0 | 0.0 | 100 | 6.38 | FAIL | PHYRE2 |
| HP0810 | 83.1 | 12.8 | 2.7 | 1.4 | 95.9 | 88.41 | PASS | MODBASE |
| HP0811 | 89.7 | 8.0 | 1.1 | 1.1 | 97.7 | 20.21 | FAIL | PHYRE2 |
| HP0812 | 86.8 | 7.9 | 3.6 | 1.7 | 94.7 | 63.6 | FAIL | PHYRE2 |
| HP0813 | 85.5 | 11.0 | 1.7 | 1.7 | 96.5 | 77.61 | WARNING | MODBASE |
| HP0814 | 90.6 | 8.0 | 1.4 | 0.0 | 98.6 | 69.67 | WARNING | MODBASE |
| HP0815 |  |  |  |  |  | 10.29 | FAIL | PHYRE2 |
| HP0816 | 93.4 | 6.6 | 0.0 | 0.0 | 100 | 94.7 | PASS | PDB |
| HP0817 | 71.6 | 20.3 | 5.4 | 2.7 | 97.3 | 65.77 | WARNING | PHYRE2 |
| HP0818 | 89.2 | 9.1 | 0.8 | 0.8 | 98.3 | 98.5 | PASS | PHYRE2 |
| HP0819 | 94.2 | 4.2 | 1.6 | 0.0 | 98.4 | 76.06 | WARNING | MODBASE |
| HP0820 | 80.6 | 16.5 | 0.7 | 2.2 | 97.1 | 15.69 | FAIL | PHYRE2 |
| HP0821 | 90.1 | 8.4 | 0.5 | 1.0 | 98.5 | 65.32 | WARNING | PHYRE2 |
| HP0822 | 92.2 | 6.7 | 1.1 | 0.0 | 98.9 | 83.85 | PASS | MODBASE |
| HP0823 | 90.0 | 8.8 | 1.2 | 0.0 | 98.8 | 48.35 | FAIL | PHYRE2 |
| HP0824 | 93.5 | 4.3 | 1.1 | 1.1 | 97.8 | 79.25 | WARNING | PHYRE2 |
| HP0825 | 90.0 | 9.6 | 0.4 | 0.0 | 99.6 | 100 | PASS | PDB |
| HP0826 | 85.5 | 11.6 | 1.2 | 1.7 | 97.1 | 49.24 | FAIL | PHYRE2 |
| HP0827 | 75.4 | 18.8 | 4.3 | 1.4 | 94.2 | 100 | PASS | PDB |
| HP0828 | 98.0 | 1.0 | 0.0 | 1.0 | 99.0 | 15.7 | FAIL | PHYRE2 |
| HP0829 | 91.1 | 8.5 | 0.4 | 0.0 | 99.6 | 74.84 | WARNING | PHYRE2 |
| HP0830 | 91.7 | 8.1 | 0.3 | 0.0 | 99.8 | 77.56 | WARNING | MODBASE |
| HP0831 | 92.5 | 4.0 | 1.7 | 1.7 | 96.5 | 74.47 | WARNING | MODBASE |
| HP0832 | 85.7 | 13.8 | 0.4 | 0.0 | 99.5 | 84.79 | PASS | PDB |
| HP0833 | 82.2 | 14.6 | 2.3 | 0.9 | 96.8 | 17.80 | FAIL | PHYRE2 |
| HP0834 | 88.9 | 7.4 | 2.0 | 1.7 | 96.3 | 73.03 | WARNING | MODBASE |
| HP0835 | 89.9 | 6.3 | 2.5 | 1.3 | 98.7 | 92.47 | PASS | PHYRE2 |
| HP0836 | 78.3 | 17.4 | 0.0 | 4.3 | 95.7 | 0 | FAIL | PHYRE2 |
| HP0837 | 84.1 | 15.9 | 0.0 | 0.0 | 100 | 26.92 | FAIL | PHYRE2 |
| HP0838 | 80.8 | 16.2 | 1.5 | 1.5 | 97.0 | 59.18 | FAIL | PHYRE2 |
| HP0839 | 83.0 | 15.9 | 1.2 | 0.0 | 98.9 | 60.34 | FAIL | PHYRE2 |
| HP0840 | 89.7 | 10.1 | 0.2 | 0.0 | 99.8 | 86.97 | PASS | PDB |
| HP0841 | 88.2 | 9.8 | 1.0 | 1.0 | 98.0 | 53.1 | FAIL | PHYRE2 |
| HP0842 | 75.9 | 20.4 | 1.9 | 1.9 | 96.3 | 0 | FAIL | PHYRE2 |
| HP0843 | 92.1 | 6.3 | 0.5 | 1.1 | 98.4 | 77.88 | WARNING | MODBASE |
| HP0844 | 88.9 | 8.4 | 1.8 | 0.9 | 97.3 | 90.08 | PASS | MODBASE |
| HP0845 | 86.9 | 9.6 | 3.1 | 0.4 | 96.5 | 76.26 | WARNING | MODBASE |
| HP0846 | 83.0 | 13.8 | 2.1 | 1.1 | 96.8 | 73.54 | WARNING | MODBASE |
| HP0847 | 69.2 | 30.8 | 0.0 | 0.0 | 100 | 0.0 | FAIL | PHYRE2 |
| HP0848 | 83.5 | 12.5 | 2.0 | 2.0 | 96.0 | 46.06 | FAIL | PHYRE2 |
| HP0849 | 74.4 | 23.2 | 0.0 | 2.4 | 97.6 | 4.40 | FAIL | PHYRE2 |
| HP0850 | 89.4 | 7.8 | 2.4 | 0.5 | 97.2 | 94.70 | PASS | MODBASE |
| HP0851 | 91.3 | 8.7 | 0.0 | 0.0 | 100 | 30.98 | FAIL | PHYRE2 |
| HP0852 | 84.1 | 12.0 | 2.7 | 1.2 | 96.1 | 23.96 | FAIL | PHYRE2 |
| HP0853 | 87.3 | 10.4 | 1.5 | 0.8 | 97.7 | 80.73 | PASS | ITASSER |
| HP0854 | 91.7 | 6.9 | 0.0 | 1,4 | 98.6 | 84.64 | PASS | MODBASE |
| HP0855 | 67.3 | 29.1 | 2.8 | 0.8 | 96.4 | 44.39 | FAIL | PHYRE2 |
| HP0856 | 83.2 | 13.6 | 1.6 | 1.6 | 96.8 | 60.75 | FAIL | PHYRE2 |
| HP0857 | 92.3 | 7.7 | 0.0 | 0.0 | 100 | 82.98 | PASS | MODBASE |
| HP0858 | 87.1 | 10.4 | 2.1 | 0.4 | 97.5 | 72.13 | WARNING | PHYRE2 |
| HP0859 | 81.8 | 16.4 | 1.4 | 0.4 | 99.6 | 0.0 | FAIL | PDB |
| HP0860 | 89.6 | 9.1 | 1.3 | 0.0 | 98.7 | 76.16 | WARNING | MODBASE |
| HP0861 | 77.8 | 16.8 | 2.7 | 2.7 | 94.6 | 10.23 | FAIL | PHYRE2 |
| HP0862 | 82.6 | 15.8 | 1.6 | 0.0 | 98.4 | 63.94 | FAIL | PHYRE2 |
| HP0863 | 84.7 | 12.2 | 2.1 | 0.9 | 96.9 | 56.64 | FAIL | PHYRE2 |
| HP0864 | 79.9 | 17.2 | 3.0 | 0.0 | 97.1 | 29.45 | FAIL | PHYRE2 |
| HP0865 | 91.5 | 8.5 | 0.0 | 0.0 | 100 | 79.71 | WARNING | MODBASE |
| HP0866 | 85.5 | 13.8 | 0.0 | 0.7 | 99.3 | 68.79 | WARNING | PHYRE2 |
| HP0867 | 86.4 | 9.8 | 3.1 | 0.7 | 96.2 | 77.11 | WARNING | PHYRE2 |
| HP0868 | 91.5 | 7.7 | 0.8 | 0.0 | 99.2 | 0 | FAIL | PHYRE2 |
| HP0869 | 76.5 | 18.8 | 2.4 | 2.4 | 95.3 | 21.28 | FAIL | PHYRE2 |
| HP0870 | 88.8 | 9.4 | 1.9 | 0.0 | 100 | 67.19 | WARNING | PHYRE2 |
| HP0871 | 92.2 | 7.3 | 0.5 | 0.0 | 99.5 | 100 | PASS | MODBASE |
| HP0872 | 80.0 | 18.5 | 0.0 | 1.5 | 98.5 | 78.87 | WARNING | MODBASE |
| HP0873 | 79.6 | 18.5 | 1.9 | 0.0 | 98.1 | 0.0 | FAIL | PHYRE2 |
| HP0874 | 83.3 | 15.4 | 0.9 | 0.5 | 98.7 | 14.40 | FAIL | PHYRE2 |
| HP0875 | 87.0 | 12.5 | 0.2 | 0.2 | 99.5 | 90.04 | PASS | PDB |
| HP0876 | 81.7 | 14.0 | 3.5 | 0.8 | 99.2 | 53.97 | FAIL | PHYRE2 |
| HP0877 | 90.6 | 6.5 | 2.9 | 0.0 | 97.1 | 67.74 | WARNING | MODBASE |
| HP0878 | 83.3 | 16.7 | 0.0 | 0.0 | 100 | 0 | FAIL | PHYRE2 |
| HP0879 | 96.3 | 3.7 | 0.0 | 0.0 | 100 | 56.04 | FAIL | MODBASE |
| HP0880 | 100.0 | 0.0 | 0.0 | 0.0 | 100 | 0.0 | FAIL | PHYRE2 |
| HP0881 | 86.7 | 13.3 | 0.0 | 0.0 | 100 | 0 | FAIL | PHYRE2 |
| HP0882 | 100.0 | 0.0 | 0.0 | 0.0 | 100 | 0.0 | FAIL | PHYRE2 |
| HP0883 | 85.8 | 13.0 | 1.2 | 0.0 | 100 | 85.25 | PASS | PHYRE2 |
| HP0884 | 93.3 | 6.7 | 0.0 | 0.0 | 100 | 0 | FAIL | PHYRE2 |
| HP0885 | 69.4 | 26.4 | 2.8 | 1.4 | 95.8 | 36.8 | FAIL | PHYRE2 |
| HP0886 | 90.5 | 7.1 | 1.7 | 0.7 | 97.6 | 75.22 | WARNING | MODBASE |
| HP0887 | 83.4 | 14.3 | 1.8 | 0.5 | 99.5 | 95.54 | PASS | PDB |
| HP0888 | 89.6 | 8.3 | 1.7 | 0.4 | 99.6 | 73.02 | WARNING | PHYRE2 |
| HP0889 | 91.7 | 7.9 | 0.0 | 0.4 | 99.6 | 42.31 | FAIL | PHYRE2 |
| HP0890 | 87.1 | 11.2 | 0.9 | 0.9 | 98.3 | 65.18 | WARNING | PHYRE2 |
| HP0891 | 91.8 | 7.5 | 0.7 | 0.0 | 99.3 | 67.53 | WARNING | PHYRE2 |
| HP0892 | 70.9 | 25.3 | 2.5 | 1.3 | 96.2 | 0 | FAIL | PDB |
| HP0893 | 85.9 | 14.1 | 0.0 | 0.0 | 100 | 0 | FAIL | PHYRE2 |
| HP0894 | 91.5 | 8.5 | 0.0 | 0.0 | 100 | 100 | PASS | PDB |
| HP0895 | 83.2 | 14.2 | 1.8 | 0.9 | 97.4 | 0.0 | FAIL | PHYRE2 |
| HP0896 | 96.6 | 3.0 | 0.4 | 0.0 | 99.6 | 0 | FAIL | PHYRE2 |
| HP0897 | 78.4 | 19.8 | 0.9 | 0.9 | 98.2 | 11.29 | FAIL | PHYRE2 |
| HP0898 | 92.2 | 6.2 | 1.2 | 0.3 | 98.4 | 91.44 | PASS | MODBASE |
| HP0899 | 87.9 | 12.0 | 0.0 | 0.0 | 100 | 8.11 | FAIL | PHYRE2 |
| HP0900 | 93.6 | 5.9 | 0.0 | 0.5 | 99.5 | 100 | PASS | PDB |
| HP0901 | 100 | 0.0 | 0.0 | 0.0 | 100 | 0 | FAIL | PHYRE2 |
| HP0902 | 84.9 | 12.8 | 0.0 | 2.3 | 97.7 | 65.31 | WARNING | PHYRE2 |
| HP0903 | NO SEQUENCE |  |  |  |  |  |  |  |
| HP0904 | NO SEQUENCE |  |  |  |  |  |  |  |
| HP0905 | 82.6 | 14.2 | 1.3 | 1.9 | 96.8 | 33.5 | FAIL | PHYRE2 |
| HP0906 | 82.6 | 16.5 | 0.8 | 0.0 | 99.1 | 30.46 | FAIL | PHYRE2 |
| HP0907 | 78.2 | 15.6 | 4.1 | 2.0 | 93.8 | 22.62 | FAIL | PHYRE2 |
| HP0908 | 81.6 | 14.1 | 2.4 | 2.0 | 95.7 | 57.3 | FAIL | PHYRE2 |
| HP0909 | 88.2 | 10.5 | 0.7 | 0.7 | 98.7 | 19.3 | FAIL | PHYRE2 |
| HP0910 | 83.6 | 12.7 | 1.9 | 1.9 | 96.3 | 67.67 | WARNING | PHYRE2 |
| HP0911 | 84.2 | 13.2 | 2.1 | 0.5 | 97.4 | 72.38 | WARNING | PHYRE2 |
| HP0912 | 84.2 | 13.2 | 2.1 | 0.5 | 97.4 | 72.38 | FAIL | PHYRE2 |
| HP0913 | 85.8 | 12.6 | 0.8 | 0.8 | 98.4 | 37.82 | FAIL | PHYRE2 |
| HP0914 | 73.7 | 21.5 | 3.1 | 1.8 | 95.2 | 32.8 | FAIL | PHYRE2 |
| HP0915 | 84.9 | 12.6 | 1.3 | 1.1 | 97.5 | 26.94 | FAIL | PHYRE2 |
| HP0916 | 84.1 | 12.1 | 2.5 | 1.3 | 96.2 | 47.57 | FAIL | PHYRE2 |
| HP0917 | TOO SMALL |  |  |  |  |  |  |  |
| HP0918 | 69.5 | 29.0 | 0.8 | 0.8 | 98.5 | 52.45 | FAIL | PHYRE2 |
| HP0919 | 92.0 | 5.8 | 1.5 | 0.6 | 97.8 | 73.11 | WARNING | MODBASE |
| HP0920 | 84.8 | 14.0 | 0.6 | 0.6 | 98.8 | 20.31 | FAIL | PHYRE2 |
| HP0921 | 86.1 | 9.5 | 3.0 | 1.4 | 95.6 | 91.87 | PASS | MODBASE |
| HP0922 | TOO LONG |  |  |  |  |  |  |  |
| HP0923 | 89.1 | 8.4 | 1.7 | 0.8 | 97.5 | 27.46 | FAIL | PHYRE2 |
| HP0924 | 94.9 | 5.1 | 0.0 | 0.0 | 100 | 100 | PASS | PDB |
| HP0925 | 84.4 | 15.1 | 0.0 | 0.6 | 99.5 | 41.97 | FAIL | PHYRE2 |
| HP0926 | 88.0 | 9.4 | 1.0 | 1.7 | 97.4 | 75.98 | WARNING | PHYRE2 |
| HP0927 | 75.4 | 18.8 | 4.3 | 1.4 | 94.2 | 50.79 | FAIL | PHYRE2 |
| HP0928 | 95.1 | 4.3 | 0.6 | 0.0 | 99.4 | 80.00 | PASS | MODBASE |
| HP0929 | 93.9 | 5.5 | 0.5 | 0.1 | 99.4 | 98.79 | PASS | PDB |
| HP0930 | 90.1 | 8.2 | 1.7 | 0.0 | 98.3 | 74.90 | WARNING | MODBASE |
| HP0931 | 84.8 | 12.1 | 3.0 | 0.0 | 96.9 | 0.0 | FAIL | PHYRE2 |
| HP0932 | 82.5 | 13.8 | 1.2 | 2.5 | 96.3 | 5.49 | FAIL | PHYRE2 |
| HP0933 | 89.5 | 8.1 | 2.4 | 0.0 | 97.6 | 68.94 | WARNING | PHYRE2 |
| HP0934 | 85.5 | 11.8 | 2.2 | 0.5 | 97.3 | 36.84 | FAIL | PHYRE2 |
| HP0935 | 92.9 | 7.1 | 0.0 | 0.0 | 100 | 68.32 | WARNING | PHYRE2 |
| HP0936 | 86.9 | 12.7 | 0.3 | 0.0 | 99.6 | 40.29 | FAIL | PHYRE2 |
| HP0937 | 81.1 | 18.9 | 0.0 | 0.0 | 100 | 0 | FAIL | PHYRE2 |
| HP0938 | 77.5 | 18.3 | 1.4 | 2.8 | 95.8 | 25.32 | FAIL | PHYRE2 |
| HP0939 | 81.7 | 15.9 | 0.5 | 1.9 | 97.6 | 24.78 | FAIL | PHYRE2 |
| HP0940 | 88.8 | 9.3 | 0.5 | 1.4 | 98.1 | 78.26 | WARNING | MODBASE |
| HP0941 | 84.6 | 13.0 | 1.8 | 0.6 | 97.6 | 98.92 | PASS | PHYRE2 |
| HP0942 | 92.8 | 6.2 | 0.3 | 0.7 | 99.0 | 63.78 | FAIL | PHYRE2 |
| HP0943 | 81.8 | 15.7 | 2.2 | 0.3 | 97.5 | 66.48 | WARNING | PHYRE2 |
| HP0944 | 92.7 | 5.5 | 1.8 | 0.0 | 98.2 | 68.25 | WARNING | MODBASE |
| HP0945 | 76.1 | 21.7 | 0.0 | 2.2 | 97.8 | 60.71 | FAIL | PHYRE2 |
| HP0946 | 83.7 | 13.2 | 2.4 | 0.8 | 96.9 | 38.23 | FAIL | PHYRE2 |
| HP0947 | 72.5 | 22.5 | 5.0 | 0.0 | 95.0 | 0.0 | FAIL | PHYRE2 |
| HP0948 | 88.7 | 8.9 | 2.0 | 0.4 | 97.6 | 0 | FAIL | PHYRE2 |
| HP0949 | 89.5 | 8.3 | 1.5 | 0.8 | 97.8 | 71.23 | WARNING | PHYRE2 |
| HP0950 | 87.4 | 9.5 | 2.2 | 0.9 | 96.9 | 72.24 | WARNING | MODBASE |
| HP0951 | 80.5 | 15.1 | 3.2 | 1.1 | 95.6 | 49.25 | FAIL | PHYRE2 |
| HP0952 | 94.4 | 4.9 | 0.7 | 0.0 | 99.3 | 87.73 | PASS | PHYRE2 |
| HP0953 | 84.1 | 10.2 | 2.3 | 3.4 | 94.3 | 0.0 | FAIL | PHYRE2 |
| HP0954 | 92.6 | 6.8 | 0.5 | 0.2 | 99.8 | 100 | PASS | PDB |
| HP0955 | 62.7 | 31.1 | 4.2 | 1.9 | 93.8 | 22.58 | FAIL | PHYRE2 |
| HP0956 | 86.6 | 12.4 | 0.5 | 0.5 | 99.0 | 65.42 | WARNING | PHYRE2 |
| HP0957 | 85.5 | 11.7 | 2.1 | 0.7 | 97.3 | 43.75 | FAIL | PHYRE2 |
| HP0958 | 94.5 | 4.4 | 0.5 | 0.5 | 98.9 | 0 | FAIL | PHYRE2 |
| HP0959 | 85.3 | 13.8 | 0.0 | 0.9 | 99.1 | 65.98 | WARNING | PHYRE2 |
| HP0960 | 93.3 | 5.1 | 0.8 | 0.8 | 98.4 | 85.66 | PASS | MODBASE |
| HP0961 | 87.8 | 8.9 | 3.3 | 0.0 | 96.7 | 74.44 | WARNING | MODBASE |
| HP0962 | 91.4 | 8.6 | 0.0 | 0.0 | 100 | 100 | PASS | PHYRE2 |
| HP0963 | 85.4 | 13.3 | 0.5 | 0.8 | 98.7 | 39.56 | FAIL | PHYRE2 |
| HP0964 | 90.1 | 9.9 | 0.0 | 0.0 | 100 | 0 | FAIL | PHYRE2 |
| HP0965 | 87.7 | 8.0 | 3.1 | 1.2 | 95.7 | 21.43 | FAIL | PHYRE2 |
| HP0966 | 85.3 | 13.3 | 0.6 | 0.8 | 98.6 | 40.33 | FAIL | PHYRE2 |
| HP0967 | 84.0 | 14.7 | 1.3 | 0.0 | 98.7 | 60.71 | FAIL | PHYRE2 |
| HP0968 | 98.1 | 1.9 | 0.0 | 0.0 | 100 | 0.0 | FAIL | PHYRE2 |
| HP0969 | 72.6 | 21.2 | 3.8 | 2.4 | 93.8 | 66.36 | WARNING | PHYRE2 |
| HP0970 | 91.1 | 6.4 | 2.0 | 0.5 | 97.5 | 43.17 | FAIL | PHYRE2 |
| HP0971 | 93.6 | 5.1 | 1.1 | 0.3 | 98.7 | 27.86 | FAIL | PHYRE2 |
| HP0972 | 81.8 | 16.2 | 1.0 | 1.0 | 98.0 | 61.11 | FAIL | PHYRE2 |
| HP0973 | 91.4 | 7.9 | 0.7 | 0.0 | 99.3 | 58.17 | FAIL | PHYRE2 |
| HP0974 | 89.0 | 10.1 | 0.7 | 0.2 | 99.1 | 89.59 | PASS | PDB |
| HP0975 | 79.3 | 17.1 | 3.7 | 0.0 | 96.4 | 8.79 | FAIL | PHYRE2 |
| HP0976 | 87.2 | 10.8 | 1.3 | 0.8 | 98.0 | 83.64 | PASS | MODBASE |
| HP0977 | 79.7 | 17.0 | 2.2 | 1.1 | 96.7 | 55.01 | FAIL | PHYRE2 |
| HP0978 | 88.8 | 10.3 | 0.0 | 0.9 | 99.1 | 88.03 | PASS | PHYRE2 |
| HP0979 | 93.5 | 6.1 | 0.0 | 0.4 | 99.6 | 95.47 | PASS | MODBASE |
| HP0980 | 75.0 | 21.2 | 3.8 | 0.0 | 96.2 | 0.0 | FAIL | PHYRE2 |
| HP0981 | 78.6 | 17.9 | 3.6 | 0.0 | 96.5 | 0 | FAIL | PHYRE2 |
| HP0982 | 83.1 | 12.3 | 3.1 | 1.5 | 95.4 | 27.92 | FAIL | PHYRE2 |
| HP0983 | 89.3 | 10.2 | 0.4 | 0.1 | 99.9 | 0.0 | FAIL | PDB |
| HP0984 | 79.2 | 20.8 | 0.0 | 0.0 | 100 | 0 | FAIL | PHYRE2 |
| HP0985 | 90.0 | 10.0 | 0.0 | 0.0 | 100 | 0 | FAIL | PHYRE2 |
| HP0986 | 84.9 | 13.9 | 0.0 | 1.2 | 98.8 | 56.04 | FAIL | PHYRE2 |
| HP0987 | 88.5 | 9.8 | 1.6 | 0.0 | 98.3 | 13.04 | FAIL | PHYRE2 |
| HP0988 | 85.5 | 12.1 | 1.6 | 0.8 | 99.2 | 77.44 | WARNING | PHYRE2 |
| HP0989 | 91.3 | 7.2 | 1.4 | 0.0 | 98.5 | 9.57 | FAIL | PHYRE2 |
| HP0990 | 84.2 | 15.8 | 0.0 | 0.0 | 100 | 45.78 | FAIL | PHYRE2 |
| HP0991 | 84.4 | 14.4 | 0.6 | 0.6 | 98.8 | 40.01 | FAIL | PHYRE2 |
| HP0992 | 83.6 | 14.9 | 1.5 | 0.0 | 98.5 | 5.26 | FAIL | PHYRE2 |
| HP0993 | 80.0 | 14.5 | 3.6 | 1.8 | 94.5 | 0.0 | FAIL | PHYRE2 |
| HP0994 | 87.2 | 10.7 | 2.1 | 0.0 | 97.9 | 19.12 | FAIL | PHYRE2 |
| HP0995 | 85.1 | 12.3 | 0.7 | 1.8 | 97.4 | 70.41 | WARNING | PHYRE2 |
| HP0996 | 84.9 | 13.8 | 1.3 | 0.0 | 98.7 | 1.19 | FAIL | PHYRE2 |
| HP0997 | 91.7 | 7.5 | 0.8 | 0.0 | 99.3 | 4.96 | FAIL | PHYRE2 |
| HP0998 | 85.5 | 12.1 | 1.6 | 0.8 | 99.2 | 77.44 | WARNING | PHYRE2 |
| HP0999 | 91.7 | 8.3 | 0.0 | 0.0 | 100 | 0 | FAIL | PHYRE2 |
| HP1000 | 88.9 | 10.1 | 0.5 | 0.5 | 99.5 | 66.05 | WARNING | PHYRE2 |
| HP1001 | 83.1 | 14.3 | 2.6 | 0.0 | 97.4 | 2.47 | FAIL | PHYRE2 |
| HP1002 | 83.2 | 15.2 | 1.0 | 0.6 | 98.4 | 0 | FAIL | PHYRE2 |
| HP1003 | 87.7 | 10.6 | 1.4 | 0.3 | 98.3 | 0 | FAIL | PHYRE2 |
| HP1004 | 86.6 | 12.2 | 0.6 | 0.6 | 98.8 | 26.92 | FAIL | PHYRE2 |
| HP1005 | 88.9 | 11.1 | 0.0 | 0.0 | 100 | 5.15 | FAIL | PHYRE2 |
| HP1006 | 91.0 | 6.9 | 1.4 | 0.7 | 97.9 | 35.85 | FAIL | PHYRE2 |
| HP1007 | NO SEQUENCE |  |  |  |  |  |  |  |
| HP1008 | 89.8 | 8.5 | 0.8 | 0.8 | 98.3 | 84.09 | PASS | MODBASE |
| HP1009 | 86.3 | 12.0 | 0.0 | 1.7 | 98.3 | 36.07 | FAIL | PHYRE2 |
| HP1010 | 88.9 | 9.0 | 1.5 | 0.7 | 97.9 | 70.18 | WARNING | MODBASE |
| HP1011 | 91.0 | 8.4 | 0.3 | 0.3 | 99.4 | 73.58 | WARNING | PDB |
| HP1012 | 88.9 | 8.4 | 1.8 | 0.8 | 97.3 | 79.09 | WARNING | PHYRE2 |
| HP1013 | 93.2 | 6.0 | 0.8 | 0.0 | 99.2 | 86.24 | PASS | MODBASE |
| HP1014 | 90.9 | 6.8 | 1.8 | 0.5 | 97.7 | 92.16 | PASS | MODBASE |
| HP1015 | 79.4 | 16.7 | 3.2 | 0.8 | 96.1 | 1.46 | FAIL | PHYRE2 |
| HP1016 | 78.5 | 18.4 | 2.5 | 0.6 | 96.9 | 8.38 | FAIL | PHYRE2 |
| HP1017 | 88.3 | 9.4 | 1.3 | 1.0 | 97.7 | 57.87 | FAIL | PHYRE2 |
| HP1018 | 87.5 | 12.5 | 0.0 | 0.0 | 100 | 0.0 | FAIL | PHYRE2 |
| HP1019 | 78.9 | 17.0 | 2.9 | 1.2 | 95.9 | 86.33 | PASS | PHYRE2 |
| HP1020 | 90.7 | 8.1 | 1.2 | 0.0 | 98.8 | 85.48 | PASS | MODBASE |
| HP1021 | 88.9 | 6.7 | 2.2 | 2.2 | 95.6 | 22.84 | FAIL | PHYRE2 |
| HP1022 | 81.1 | 16.7 | 1.8 | 0.5 | 97.8 | 29.58 | FAIL | PHYRE2 |
| HP1023 | 84.0 | 13.2 | 1.2 | 1.6 | 97.2 | 16.39 | FAIL | PHYRE2 |
| HP1024 | 78.9 | 16.2 | 4.2 | 0.7 | 95.1 | 66.88 | WARNING | PHYRE2 |
| HP1025 | 85.6 | 13.4 | 1.0 | 0.0 | 100 | 72.48 | WARNING | PHYRE2 |
| HP1026 | 83.6 | 14.6 | 1,8 | 0.0 | 98.2 | 68.63 | WARNING | PHYRE2 |
| HP1027 | 93.9 | 6.1 | 0.0 | 0.0 | 100 | 91.25 | PASS | PDB |
| HP1028 | 92.3 | 6.6 | 1.2 | 0.0 | 100 | 93.10 | PASS | PDB |
| HP1029 | 87.3 | 11.2 | 0.7 | 0.7 | 98.5 | 39.22 | FAIL | PHYRE2 |
| HP1030 | 92.3 | 6.5 | 0.6 | 0.6 | 98.8 | 70.18 | WARNING | PHYRE2 |
| HP1031 | 89.3 | 10.0 | 0.0 | 0.0 | 100 | 92.10 | PASS | PDB |
| HP1032 | 86.8 | 12.3 | 0.9 | 0.0 | 100 | 36.10 | FAIL | PHYRE2 |
| HP1033 | 85.5 | 10.9 | 3.6 | 0.0 | 96.4 | 0.0 | FAIL | PHYRE2 |
| HP1034 | 89.5 | 8.6 | 1.4 | 0.5 | 98.1 | 75.21 | WARNING | MODBASE |
| HP1035 | 87.0 | 9.6 | 2.3 | 1.1 | 96.6 | 79.44 | WARNING | PHYRE2 |
| HP1036 | 89.4 | 8.1 | 1.6 | 0.8 | 97.5 | 72.34 | WARNING | MODBASE |
| HP1037 | 88.0 | 8.9 | 2.1 | 0.9 | 96.9 | 78.21 | WARNING | MODBASE |
| HP1038 | 87.2 | 9.2 | 2.1 | 1.4 | 96.4 | 89.94 | PASS | PDB |
| HP1039 | 73.7 | 21.9 | 3.2 | 1.2 | 95.6 | 11.61 | FAIL | PHYRE2 |
| HP1040 | 93.8 | 6.2 | 0.0 | 0.0 | 100 | 78.41 | WARNING | MODBASE |
| HP1041 | 91.1 | 8.9 | 0.0 | 0.0 | 100 | 91.12 | PASS | PDB |
| HP1042 | 81.4 | 15.1 | 1.9 | 1.6 | 96.5 | 31.95 | FAIL | PHYRE2 |
| HP1043 | 95.3 | 4.7 | 0.0 | 0.0 | 100 | 99.13 | PASS | PDB |
| HP1044 | 84.7 | 12.4 | 1.8 | 1.2 | 97.1 | 80.71 | PASS | PHYRE2 |
| HP1045 | 91.1 | 8.1 | 0.4 | 0.5 | 99.2 | 87.21 | PASS | MODBASE |
| HP1046 | 72.4 | 20.9 | 6.0 | 0.7 | 93.3 | 80.82 | PASS | PHYRE2 |
| HP1047 | 90.6 | 7.8 | 1.6 | 0.0 | 98.4 | 50.70 | FAIL | PHYRE2 |
| HP1048 | 75.7 | 16.8 | 4.8 | 2.7 | 92.5 | 39.72 | FAIL | PHYRE2 |
| HP1049 | 81.4 | 15.7 | 1.4 | 1.4 | 97.1 | 32.91 | FAIL | PHYRE2 |
| HP1050() | 84.1 | 13.6 | 1.2 | 1.2 | 97.7 | 76.71 | WARNING | PHYRE2 |
| HP1051 | 86.3 | 8.5 | 1.7 | 3.4 | 94.8 | 30.53 | FAIL | PHYRE2 |
| HP1052 | 89.2 | 9.3 | 1.5 | 0.0 | 98.5 | 85.91 | PASS | MODBASE |
| HP1053 | 83.0 | 12.4 | 3.3 | 1.3 | 95.4 | 48.24 | FAIL | PHYRE2 |
| HP1054 | 83.5 | 11.2 | 3.7 | 1.6 | 94.7 | 50.68 | FAIL | PHYRE2 |
| HP1055 | 83.7 | 13.1 | 2.0 | 1.3 | 96.8 | 42.62 | FAIL | PHYRE2 |
| HP1056 | 83.7 | 11.8 | 3.3 | 1.3 | 95.5 | 50.83 | FAIL | PHYRE2 |
| HP1057 | 79.9 | 18.4 | 1.1 | 0.6 | 98.3 | 3.38 | FAIL | PHYRE2 |
| HP1058 | 93.9 | 3.9 | 1.3 | 0.9 | 97.8 | 85.88 | PASS | MODBASE |
| HP1059 | 92.8 | 5.8 | 0.4 | 1.1 | 98.6 | 92.33 | PASS | MODBASE |
| HP1060 | 90.8 | 8.5 | 0.0 | 0.8 | 99.3 | 0.0 | FAIL | PHYRE2 |
| HP1061 | 83.1 | 15.3 | 0.6 | 1.1 | 98.4 | 15.87 | FAIL | PHYRE2 |
| HP1062 | 89.0 | 8.7 | 1.9 | 0.4 | 97.7 | 73.96 | WARNING | PHYRE2 |
| HP1063 | 89.4 | 8.1 | 2.5 | 0.0 | 97.5 | 69.49 | WARNING | MODBASE |
| HP1064 | 82.6 | 13.0 | 0.0 | 4.3 | 95.6 | 0.0 | FAIL | PHYRE2 |
| HP1065 | 85.1 | 11.9 | 3.0 | 0.0 | 97.0 | 48.62 | FAIL | PHYRE2 |
| HP1066 | 92.4 | 5.9 | 0.8 | 0.8 | 98.3 | 5.22 | FAIL | PHYRE2 |
| HP1067 | 90.9 | 8.2 | 0.9 | 0.0 | 99.1 | 87.10 | PASS | PDB |
| HP1068 | 90.7 | 7.4 | 1.9 | 0.0 | 98.1 | 54.43 | FAIL | PHYRE2 |
| HP1069 | 59.0 | 33.4 | 7.6 | 0.0 | 92.4 | 83.20 | PASS | PDB |
| HP1070 | 88.9 | 11.1 | 0.0 | 0.0 | 100 | 0 | FAIL | PHYRE2 |
| HP1071 | 62.4 | 32.0 | 4.6 | 1.0 | 94.4 | 2.71 | FAIL | PHYRE2 |
| HP1072 | 57.0 | 33.1 | 7.9 | 2.0 | 90.1 | 47.34 | FAIL | PHYRE2 |
| HP1073 | 74.6 | 25.4 | 0.0 | 0.0 | 100 | 80.60 | PASS | PDB |
| HP1074 | 95.8 | 4.2 | 0.0 | 0.0 | 100 | 0.0 | FAIL | PHYRE2 |
| HP1075 | 81.2 | 15.0 | 2.1 | 1.7 | 96.2 | 44.93 | FAIL | PHYRE2 |
| HP1076 | 95.3 | 4.7 | 0.0 | 0.0 | 100 | 91.38 | PASS | PDB |
| HP1077 | 80.4 | 15.4 | 2.8 | 1.4 | 95.8 | 13.02 | FAIL | PHYRE2 |
| HP1078 | 87.7 | 7.4 | 1.6 | 3.3 | 95.1 | 10.45 | FAIL | PHYRE2 |
| HP1079 | 88.2 | 9.1 | 2.8 | 0.0 | 97.3 | 24.24 | FAIL | PHYRE2 |
| HP1080 | 80.4 | 14.7 | 3.9 | 1.0 | 95.1 | 34.55 | FAIL | PHYRE2 |
| HP1081 | 87.0 | 11.1 | 1.2 | 0.6 | 98.1 | 49.73 | FAIL | PHYRE2 |
| HP1082 | 76.4 | 20.0 | 3.2 | 0.4 | 96.4 | 54.55 | FAIL | PHYRE2 |
| HP1083 | 71.4 | 24.1 | 1.8 | 2.7 | 95.5 | 59.93 | FAIL | PHYRE2 |
| HP1084 | 88.3 | 9.6 | 1.1 | 1.1 | 97.9 | 68.52 | WARNING | MODBASE |
| HP1085 | 73.9 | 23.9 | 2.2 | 0.0 | 97.8 | 5.26 | FAIL | PHYRE2 |
| HP1086 | 87.9 | 8.9 | 1.9 | 1.4 | 98.6 | 84.26 | PASS | PHYRE2 |
| HP1087 | 82.8 | 14.6 | 2.1 | 0.4 | 97.4 | 59.18 | FAIL | PHYRE2 |
| HP1088 | 91.8 | 6.8 | 0.5 | 0.9 | 98.6 | 85.94 | PASS | MODBASE |
| HP1089 | 76.1 | 17.5 | 3.5 | 2.9 | 93.6 | 28.09 | FAIL | PHYRE2 |
| HP1090 | 87.2 | 11.3 | 0.9 | 0.6 | 98.5 | 92.76 | PASS | PHYRE2 |
| HP1091 | 80.9 | 16.6 | 1.7 | 0.8 | 97.5 | 52.36 | FAIL | PHYRE2 |
| HP1092 | 79.2 | 15.1 | 2.8 | 2.8 | 94.3 | 67.69 | WARNING | PHYRE2 |
| HP1093 | SMALL -28 |  |  |  |  |  |  |  |
| HP1094 | 63.6 | 30.3 | 0.0 | 6.1 | 93.9 | 0 | FAIL | PHYRE2 |
| HP1095 | 92.1 | 7.1 | 0.8 | 0.0 | 99.2 | 4.96 | FAIL | PHYRE2 |
| HP1096 | 85.5 | 12.1 | 1.6 | 0.8 | 99.2 | 77.44 | WARNING | PHYRE2 |
| HP1097 | 90.0 | 10.0 | 0.0 | 0.0 | 100 | 0.0 | FAIL | PHYRE2 |
| HP1098 | 90.8 | 9.2 | 0.0 | 0.0 | 100 | 95.49 | PASS | PDB |
| HP1099 | 94.4 | 5.6 | 0.0 | 0.0 | 100 | 100 | PASS | MODBASE |
| HP1100 | 87.9 | 9.5 | 1.9 | 0.8 | 97.4 | 74.17 | WARNING | MODBASE |
| HP1101 | 88.0 | 9.7 | 1.5 | 0.8 | 97.7 | 79.06 | WARNING | MODBASE |
| HP1102 | 88.8 | 8.7 | 1.9 | 0,5 | 97.5 | 95.11 | PASS | MODBASE |
| HP1103 | 90.0 | 9.3 | 0.7 | 0.0 | 99.3 | 86.89 | PASS | MODBASE |
| HP1104 | 88.5 | 11.0 | 0.5 | 0.0 | 100 | 99.14 | PASS | PDB |
| HP1105 | 85.1 | 11.6 | 2.1 | 1.2 | 96.7 | 59.25 | FAIL | PHYRE2 |
| HP1106 | 81.4 | 17.5 | 1.0 | 0.0 | 98.9 | 42.86 | FAIL | PHYRE2 |
| HP1107 | 87.9 | 10.1 | 0.7 | 1.3 | 98.0 | 18.54 | FAIL | PHYRE2 |
| HP1108 | 86.0 | 10.4 | 2.4 | 1.2 | 96.4 | 91.83 | PASS | MODBASE |
| HP1109 | 79.6 | 16.3 | 4.1 | 0.0 | 95.9 | 30.97 | FAIL | PHYRE2 |
| HP1110 | 88.7 | 10.7 | 0.7 | 0.0 | 99.4 | 76.88 | WARNING | PHYRE2 |
| HP1111 | 89.7 | 7.9 | 1.6 | 0.8 | 97.6 | 77.88 | WARNING | PHYRE2 |
| HP1112 | 94.6 | 4.7 | 0.5 | 0.2 | 99.3 | 79.91 | WARNING | MODBASE |
| HP1113 | 88.5 | 10.8 | 0.0 | 0.8 | 99.3 | 32.92 | FAIL | PHYRE2 |
| HP1114 | 86.8 | 9.9 | 1.8 | 1.5 | 96.7 | 67.50 | WARNING | MODBASE |
| HP1115 | 79.9 | 15.8 | 2.4 | 1.9 | 95.7 | 0 | FAIL | PHYRE2 |
| HP1116 | 73.4 | 25.0 | 1.3 | 0.3 | 98.4 | 4.75 | FAIL | PHYRE2 |
| HP1117 | 92.2 | 6.8 | 1.0 | 0.0 | 99.0 | 91.40 | PASS | MODBASE |
| HP1118 | 90.9 | 8.6 | 0.1 | 0.4 | 99.5 | 95.45 | PASS | PDB |
| HP1119 | 90.3 | 7.3 | 1.9 | 0.5 | 97.6 | 64.75 | FAIL | PHYRE2 |
| HP1120 | 96.6 | 3.4 | 0.0 | 0.0 | 100 | 31.67 | FAIL | PHYRE2 |
| HP1121 | 87.2 | 10.7 | 1.4 | 0.7 | 97.9 | 76.04 | WARNING | MODBASE |
| HP1122 | 84.8 | 15.2 | 0.0 | 0.0 | 100 | 27.03 | FAIL | PHYRE2 |
| HP1123 | 83.7 | 16.3 | 0.0 | 0.0 | 100 | 91.83 | PASS | PDB |
| HP1124 | 76.9 | 20.4 | 1,4 | 1.4 | 97.3 | 66.88 | WARNING | PHYRE2 |
| HP1125 | 95.9 | 3.1 | 1.0 | 0.0 | 99.0 | 70.09 | WARNING | MODBASE |
| HP1126 | 86.4 | 10.7 | 2.0 | 0.8 | 97.1 | 77.75 | WARNING | PHYRE2 |
| HP1127 | 93.0 | 4.0 | 2.0 | 1.0 | 97.0 | 76.11 | WARNING | PHYRE2 |
| HP1128 | 92.9 | 3.6 | 3.6 | 0.0 | 96.5 | 0 | FAIL | PHYRE2 |
| HP1129 | 78.4 | 18.2 | 3.4 | 0.0 | 96.6 | 47.37 | FAIL | PHYRE2 |
| HP1130 | 86.5 | 12.3 | 0.6 | 0.6 | 98.8 | 18.33 | FAIL | PHYRE2 |
| HP1131 | 84.4 | 8.9 | 4.4 | 2.2 | 93.3 | 70.19 | WARNING | MODBASE |
| HP1132 | 91.9 | 6.3 | 1.3 | 0.5 | 98.2 | 91.63 | PASS | MODBASE |
| HP1133 | 90.6 | 6.7 | 1.8 | 0.9 | 97.3 | 70.90 | WARNING | MODBASE |
| HP1134 | 88.5 | 8.9 | 1.4 | 1.8 | 97.4 | 84.91 | PASS | MODBASE |
| HP1135 | 81.2 | 14.6 | 1.0 | 3.1 | 95.8 | 59.41 | FAIL | PHYRE2 |
| HP1136 | 91.7 | 4.2 | 0.0 | 4.2 | 95.9 | 0.0 | FAIL | PHYRE2 |
| HP1137 | 92.4 | 7.6 | 0.0 | 0.0 | 100 | 0.0 | FAIL | PHYRE2 |
| HP1138 | 95.9 | 2.3 | 0.6 | 1.2 | 98.2 | 76.19 | WARNING | MODBASE |
| HP1139 | 87.8 | 10.0 | 1.3 | 0.9 | 97.8 | 80.46 | PASS | MODBASE |
| HP1140 | 82.1 | 13.2 | 3.3 | 1.3 | 95.3 | 97.11 | PASS | MODBASE |
| HP1141 | 88.3 | 9.8 | 0.8 | 1.1 | 98.1 | 85.48 | PASS | MODBASE |
| HP1142 | 92.6 | 7.1 | 0.0 | 0.4 | 99.7 | 0 | FAIL | PHYRE2 |
| HP1143 | 91.4 | 7.9 | 0.7 | 0.0 | 99.3 | 0 | FAIL | PHYRE2 |
| HP1144 | 86.2 | 12.1 | 0.0 | 1.7 | 98.3 | 20.90 | FAIL | PHYRE2 |
| HP1145 | 86.2 | 12.1 | 0.0 | 1.7 | 98.3 | 20.90 | FAIL | PHYRE2 |
| HP1146 | 83.8 | 13.2 | 2.9 | 0.0 | 97.0 | 5.26 | FAIL | PHYRE2 |
| HP1147 | 37.3 | 40.2 | 16.7 | 5.9 | 77.5 | 12.82 | FAIL | PHYRE2 |
| HP1148 | 92.0 | 6.5 | 1.0 | 0.5 | 98.5 | 77.73 | WARNING | PHYRE2 |
| HP1149 | 84.7 | 10.8 | 1.9 | 2.5 | 95.5 | 52.57 | WARNING | PHYRE2 |
| HP1150 | 83.0 | 14.9 | 0.0 | 2.1 | 97.9 | 12.28 | WARNING | PHYRE2 |
| HP1151 | 93.8 | 4.6 | 1.5 | 0.0 | 98.4 | 100 | PASS | MODBASE |
| HP1152 | 89.0 | 8.1 | 0.8 | 2.1 | 97.1 | 78.87 | WARNING | MODBASE |
| HP1153 | 85.5 | 12.4 | 1.4 | 0.8 | 97.9 | 83.29 | PASS | MODBASE |
| HP1154 | 84.6 | 13.7 | 1.7 | 0.0 | 98.3 | 70.23 | WARNING | PHYRE2 |
| HP1155 | 88.7 | 8.1 | 1.9 | 1.3 | 96.8 | 79.26 | WARNING | PHYRE2 |
| HP1156 | 88.5 | 10.8 | 0.8 | 0.0 | 99.3 | 22.93 | FAIL | PHYRE2 |
| HP1157 | TOO LONG |  |  |  |  | 3.38 | FAIL | PHYRE2 |
| HP1158 | 90.7 | 7.1 | 1.3 | 0.9 | 97.8 | 38.43 | FAIL | PHYRE2 |
| HP1159 | 94.2 | 5.8 | 0.0 | 0.0 | 100 | 89.27 | PASS | PDB |
| HP1160 | 87.0 | 9.6 | 1.7 | 1.7 | 96.6 | 99.23 | PASS | MODBASE |
| HP1161 | 88.7 | 11.3 | 0.0 | 0.0 | 100 | 100 | PASS | PDB |
| HP1162 | 78.6 | 19.6 | 2.4 | 1.2 | 98.2 | 24.86 | FAIL | PHYRE2 |
| HP1163 | 89.3 | 10.7 | 0.0 | 0.0 | 100 | 0.0 | FAIL | PHYRE2 |
| HP1164 | 84.1 | 10.9 | 3.3 | 1.8 | 95.0 | 83.87 | PASS | PHYRE2 |
| HP1165 | 76.9 | 18.6 | 4.1 | 0.3 | 95.5 | 26.17 | FAIL | PHYRE2 |
| HP1166 | 93.5 | 5.8 | 0.4 | 0.2 | 99.3 | 86.08 | PASS | MODBASE |
| HP1167 | 71.9 | 23.7 | 3.3 | 1.1 | 95.6 | 37.76 | FAIL | PHYRE2 |
| HP1168 | 69.4 | 26.7 | 2.0 | 2.0 | 96.0 | 53.52 | FAIL | PHYRE2 |
| HP1169 | 84.8 | 14.7 | 0.0 | 0.5 | 99.5 | 25.84 | FAIL | PHYRE2 |
| HP1170 | 83.1 | 15.1 | 0.0 | 1.8 | 98.2 | 36.41 | FAIL | PHYRE2 |
| HP1171 | 92.3 | 6.4 | 0.9 | 0.5 | 98.7 | 86.89 | PASS | MODBASE |
| HP1172 | 92.7 | 6.4 | 0.5 | 0.5 | 99.1 | 100 | PASS | MODBASE |
| HP1173 | 75.0 | 18.8 | 2.1 | 4.2 | 93.8 | 0.0 | FAIL | PHYRE2 |
| HP1174 | 86.4 | 12.2 | 0.9 | 0.6 | 98.6 | 59.19 | FAIL | PHYRE2 |
| HP1175 | 71.6 | 25.1 | 3.0 | 0.3 | 96.7 | 42.27 | FAIL | PHYRE2 |
| HP1176 | 83.3 | 16.7 | 0.0 | 0.0 | 100 | 0.0 | FAIL | PHYRE2 |
| HP1177 | 79.8 | 13.9 | 5.6 | 0.7 | 93.7 | 21.09 | FAIL | PHYRE2 |
| HP1178 | 93.2 | 6.3 | 0.5 | 0.0 | 99.5 | 85.34 | PASS | MODBASE |
| HP1179 | 84.8 | 11.6 | 2.8 | 0.8 | 96.4 | 97.56 | PASS | MODBASE |
| HP1180 | 82.4 | 12.5 | 2.8 | 2.3 | 94.9 | 13.66 | FAIL | PHYRE2 |
| HP1181 | 74.4 | 20.5 | 4.7 | 0.3 | 94.9 | 40.82 | FAIL | PHYRE2 |
| HP1182 | 85.4 | 11.9 | 1.8 | 0.9 | 97.3 | 70.52 | WARNING | PHYRE2 |
| HP1183 | 70.7 | 26.1 | 2.2 | 1.0 | 96.8 | 42.82 | FAIL | PHYRE2 |
| HP1184 | 85.2 | 11.6 | 1.3 | 2.0 | 96.8 | 45.39 | FAIL | PHYRE2 |
| HP1185 | 78.0 | 16.8 | 4.7 | 0.6 | 94.8 | 45.41 | FAIL | PHYRE2 |
| HP1186 | 85.8 | 13.0 | 1.2 | 0.0 | 98.8 | 86.41 | PASS | MODBASE |
| HP1187 | 93.6 | 6.0 | 0.0 | 0.4 | 99.6 | 2.54 | FAIL | PHYRE2 |
| HP1188 | 82.7 | 14.5 | 1.7 | 1.1 | 97.2 | 27.36 | FAIL | PHYRE2 |
| HP1189 | 89.7 | 6.5 | 1.9 | 1.9 | 96.2 | 68.90 | WARNING | MODBASE |
| HP1190 | 86.0 | 11.7 | 1.6 | 0.8 | 97.7 | 76.69 | WARNING | PHYRE2 |
| HP1191 | 90.0 | 9.0 | 0.7 | 0.3 | 99.0 | 59.0 | FAIL | PHYRE2 |
| HP1192 | 91.8 | 6.1 | 2.0 | 0.0 | 97.9 | 0.0 | FAIL | PHYRE2 |
| HP1193 | 87.5 | 10.0 | 1.4 | 1.1 | 97.5 | 87.77 | PASS | PHYRE2 |
| HP1194 | SMALL-28 |  |  |  |  |  |  |  |
| HP1195 | 0.0 | 0.0 | 0.0 | 100.0 | 0.0 | 6.71 | FAIL | PHYRE2 |
| HP1196 | 77.5 | 19.6 | 1.4 | 1.4 | 97.1 | 76.47 | WARNING | PHYRE2 |
| HP1197 | 70.3 | 21.8 | 5.9 | 2.0 | 92.1 | 62.18 | FAIL | PHYRE2 |
| HP1198 | TOO LONG |  |  |  |  |  |  |  |
| HP1199 | 95.4 | 3.7 | 0.9 | 0.0 | 99.1 | 79.84 | WARNING | MODBASE |
| HP1200 | 88.8 | 7.7 | 1.4 | 2.1 | 97.9 | 66.67 | WARNING | PHYRE2 |
| HP1201 | 94.1 | 5.9 | 0.0 | 0.0 | 100 | 0 | FAIL | PDB |
| HP1202 | 76.9 | 19.7 | 1.7 | 1.7 | 96.6 | 90.71 | PASS | ITASSER |
| HP1203 | 81.4 | 16.7 | 2.0 | 0.0 | 100 | 0.0 | FAIL | PDB |
| HP1203.1 | 0.0 | 0.0 | 0.0 | 100.0 | 0.0 | 0.0 | FAIL | PHYRE2 |
| HP1204 | 40.0 | 45.0 | 7.5 | 7.5 | 85.0 | 2.22 | FAIL | PHYRE2 |
| HP1205 | 89.9 | 7.4 | 1.2 | 1.5 | 97.3 | 90.23 | PASS | MODBASE |
| HP1206 | 76.4 | 20.2 | 3.2 | 0.2 | 96.6 | 50.83 | FAIL | PHYRE2 |
| HP1207 | 84.8 | 13.1 | 1.0 | 1.0 | 97.9 | 88.24 | PASS | PHYRE2 |
| HP1208 | 86.5 | 11.4 | 1.2 | 0.8 | 97.9 | 43.7 | FAIL | PHYRE2 |
| HP1209 | 82.3 | 12.9 | 4.8 | 0.0 | 95.2 | 76.40 | WARNING | PHYRE2 |
| HP1210 | 89.4 | 9.2 | 0.7 | 0.7 | 98.6 | 75 | WARNING | MODBASE |
| HP1211 | 92.1 | 4.8 | 1.6 | 1.6 | 96.9 | 40.51 | FAIL | PHYRE2 |
| HP1212 | 95.4 | 4.6 | 0.0 | 0.0 | 100 | 25.93 | FAIL | PHYRE2 |
| HP1213 | 91.1 | 7.7 | 0.6 | 0.6 | 98.8 | 75.19 | WARNING | MODBASE |
| HP1214 | 86.1 | 10.8 | 2.6 | 0.5 | 96.9 | 81.5 | PASS | PHYRE2 |
| HP1215 | 76.3 | 13.6 | 5.1 | 5.1 | 89.9 | 36.62 | FAIL | PHYRE2 |
| HP1216 | 82.6 | 11.8 | 3.9 | 1.7 | 94.4 | 17.57 | FAIL | PHYRE2 |
| HP1217 | 83.1 | 15.4 | 1.5 | 0.0 | 98.5 | 0.0 | FAIL | PHYRE2 |
| HP1218 | 90.1 | 8.9 | 0.5 | 0.5 | 99.0 | 94.94 | PASS | MODBASE |
| HP1219 | 85.1 | 10.6 | 4.3 | 0.0 | 95.7 | 0.0 | FAIL | PHYRE2 |
| HP1220 | 88.6 | 8.4 | 1.5 | 1.5 | 98.5 | 89.87 | PASS | PHYRE2 |
| HP1221 | 91.6 | 8.4 | 0.0 | 0.0 | 100 | 96.42 | PASS | PDB |
| HP1222 | 85.2 | 13.0 | 1.0 | 0.8 | 98.2 | 74.7 | WARNING | PHYRE2 |
| HP1223 | 81.4 | 16.7 | 2.0 | 0.0 | 98.1 | 0 | FAIL | PDB |
| HP1224 | 94.8 | 4.7 | 0.5 | 0.0 | 99.5 | 76.63 | WARNING | PDB |
| HP1225 | 81.0 | 14.0 | 3.0 | 2.0 | 95.0 | 1.63 | FAIL | PHYRE2 |
| HP1226 | 87.6 | 9.5 | 1.3 | 1.6 | 97.1 | 65.5 | WARNING | PHYRE2 |
| HP1227 | 85.7 | 11.4 | 2.9 | 0.0 | 97.1 | 73.08 | WARNING | MODBASE |
| HP1228 | 87.2 | 9.8 | 1.5 | 1.5 | 97.0 | 92.05 | PASS | MODBASE |
| HP1229 | 91.3 | 7.1 | 1.4 | 0.3 | 98.4 | 79.46 | WARNING | MODBASE |
| HP1230 | 91.0 | 6.9 | 0.7 | 1.3 | 97.9 | 100 | PASS | PDB |
| HP1231 | 88.2 | 9.3 | 1.5 | 1.0 | 97.5 | 26.05 | FAIL | ITASSER |
| HP1232 | 88.0 | 10.0 | 1.2 | 0.8 | 98.0 | 81.18 | PASS | MODBASE |
| HP1233 | 81.8 | 15.2 | 3.0 | 0.0 | 97.0 | 0.0 | FAIL | PHYRE2 |
| HP1234 | 79.1 | 17.5 | 3.4 | 0.0 | 96.6 | 3.3 | FAIL | PHYRE2 |
| HP1235 | 86.2 | 9.9 | 1.5 | 2.5 | 96.1 | 23.33 | FAIL | PHYRE2 |
| HP1236 | 79.9 | 15.4 | 4.0 | 0.7 | 95.3 | 33.55 | FAIL | PHYRE2 |
| HP1237 | 89.6 | 8.2 | 1.2 | 0.9 | 97.8 | 82.13 | PASS | MODBASE |
| HP1238 | 88.2 | 10.6 | 0.7 | 0.4 | 98.8 | 97.58 | PASS | PDB |
| HP1239 | SMALL-29 |  |  |  |  |  |  |  |
| HP1240 | 93.2 | 5.4 | 1.4 | 0.0 | 98.6 | 76.07 | WARNING | PHYRE2 |
| HP1241 | 87.4 | 10.8 | 1.8 | 0.0 | 98.2 | 85.24 | PASS | PHYRE2 |
| HP1242 | 94.5 | 5.5 | 0.0 | 0.0 | 100 | 0 | FAIL | PDB |
| HP1243 | 82.1 | 13.7 | 3.1 | 1.0 | 95.8 | 46.80 | FAIL | PHYRE2 |
| HP1244 | 74.0 | 22.0 | 2.0 | 2.0 | 96.0 | 0 | FAIL | PHYRE2 |
| HP1245 | 91.6 | 8.4 | 0.0 | 0.0 | 100 | 97.22 | PASS | PDB |
| HP1246 | 80.3 | 13.4 | 4.7 | 1.6 | 98.4 | 37.50 | FAIL | PHYRE2 |
| HP1247 | 84.9 | 13.1 | 1.3 | 0.7 | 98.0 | 61.98 | FAIL | PHYRE2 |
| HP1248 | 84.8 | 13.2 | 1.3 | 0.7 | 98.0 | 68.48 | WARNING | PHYRE2 |
| HP1249 | 90.9 | 7.8 | 0.9 | 0.4 | 98.7 | 90.49 | PASS | MODBASE |
| HP1250 | 68.7 | 26.9 | 1.5 | 3.0 | 95.6 | 47.56 | FAIL | PHYRE2 |
| HP1251 | 83.3 | 13.2 | 1.8 | 1.8 | 96.5 | 42.97 | FAIL | PHYRE2 |
| HP1252 | 85.5 | 13.2 | 1.1 | 0.2 | 98.7 | 86.98 | PASS | PHYRE2 |
| HP1253 | 94.7 | 4.6 | 0.7 | 0.0 | 99.3 | 8545 | PASS | MODBASE |
| HP1254 | 82.4 | 14.6 | 2.0 | 1.0 | 97.0 | 54.42 | FAIL | PHYRE2 |
| HP1255 | 0.0 | 0.0 | 0.0 | 100.0 | 0.0 | 34.62 | FAIL | PHYRE2 |
| HP1256 | 98.3 | 1.7 | 0.0 | 0.0 | 100 | 90.86 | PASS | MODBASE |
| HP1257 | 88.5 | 8.3 | 2.6 | 0.6 | 96.8 | 80.57 | PASS | PHYRE2 |
| HP1258 | 82.0 | 15.0 | 2.0 | 1.0 | 97.0 | 5.41 | FAIL | PHYRE2 |
| HP1259 | 83.3 | 14.4 | 1.7 | 0.6 | 97.7 | 68.50 | WARNING | PHYRE2 |
| HP1260 | 87.0 | 10.4 | 1.3 | 1.3 | 97.4 | 0.0 | FAIL | PHYRE2 |
| HP1261 | 85.1 | 11.9 | 2.2 | 0.9 | 97.0 | 66.46 | WARNING | MODBASE |
| HP1262 | 63.6 | 29.4 | 5.6 | 1.4 | 93.0 | 46.06 | FAIL | PHYRE2 |
| HP1263 | 91.3 | 8.7 | 0.0 | 0.0 | 100 | 84.24 | PASS | MODBASE |
| HP1264 | 76.5 | 16.2 | 7.4 | 0.0 | 100 | 0.0 | FAIL | PDB |
| HP1265 | 72.8 | 20.8 | 5.0 | 1.5 | 93.6 | 46.33 | FAIL | PHYRE2 |
| HP1266 | 70.3 | 24.6 | 4.5 | 0.6 | 94.9 | 50.84 | FAIL | PHYRE2 |
| HP1267 | 66.4 | 29.6 | 4.0 | 0.0 | 96.0 | 29.38 | FAIL | PHYRE2 |
| HP1268 | 75.8 | 20.0 | 3.2 | 1.1 | 95.8 | 79.09 | WARNING | PHYRE2 |
| HP1269 | 81.6 | 13.6 | 4.1 | 0.7 | 95.2 | 0.0 | FAIL | PHYRE2 |
| HP1270 | 96.5 | 3.5 | 0.0 | 0.0 | 100 | 8.82 | FAIL | PHYRE2 |
| HP1271 | 70.1 | 28.0 | 2.0 | 0.0 | 98.1 | 37.84 | FAIL | PHYRE2 |
| HP1272 | 87.4 | 11.6 | 0.7 | 0.4 | 99.0 | 36.72 | FAIL | PHYRE2 |
| HP1273 | 82.1 | 15.2 | 1.8 | 0.9 | 97.3 | 49.2 | FAIL | PHYRE2 |
| HP1274 | 81.2 | 15.0 | 2.5 | 1.3 | 96.2 | 47.78 | FAIL | PHYRE2 |
| HP1275 | 88.4 | 9.9 | 1.5 | 0.2 | 98.3 | 79.78 | WARNING | MODBASE |
| HP1276 | 98.5 | 1.5 | 0.0 | 0.0 | 100 | 0.0 | FAIL | PHYRE2 |
| HP1277 | 94.8 | 4.4 | 0.4 | 0.4 | 99.2 | 86.54 | PASS | MODBASE |
| HP1278 | 92.6 | 6.8 | 0.6 | 0.0 | 99.4 | 91 | PASS | MODBASE |
| HP1279 | 91.5 | 7.5 | 0.2 | 0.7 | 99.0 | 81.90 | PASS | MODBASE |
| HP1280 | 87.2 | 11.4 | 1.3 | 0.0 | 98.6 | 97.31 | PASS | PHYRE2 |
| HP1281 | 90.3 | 7.9 | 0.6 | 1.2 | 98.2 | 81.68 | PASS | MODBASE |
| HP1282 | 90.6 | 7.9 | 1.5 | 0.0 | 98.5 | 81.60 | PASS | MODBASE |
| HP1283 | 88.1 | 8.7 | 2.2 | 1.0 | 96.8 | 63.98 | FAIL | PHYRE2 |
| HP1284 | 89.0 | 8.9 | 1.7 | 0.3 | 97.9 | 79.57 | WARNING | PHYRE2 |
| HP1285 | 86.1 | 12.0 | 1.4 | 0.5 | 98.1 | 76.32 | WARNING | MODBASE |
| HP1286 | 85.9 | 11.4 | 1.7 | 1.0 | 99 | 98.79 | PASS | PDB |
| HP1287 | 90.2 | 8.1 | 1.7 | 0.0 | 100 | 98.2 | PASS | PDB |
| HP1288 | 71.4 | 14.3 | 9.5 | 4.8 | 85.7 | 0.0 | FAIL | PHYRE2 |
| HP1289 | 83.2 | 10.9 | 3.0 | 3.0 | 94.1 | 38.60 | FAIL | PHYRE2 |
| HP1290 | 77.4 | 17.7 | 3.2 | 1.6 | 95.1 | 16.26 | FAIL | PHYRE2 |
| HP1291 | 86.0 | 9.5 | 1.7 | 2.8 | 95.5 | 71.86 | WARNING | MODBASE |
| HP1292 | 82.9 | 13.3 | 1.9 | 1.9 | 96.2 | 89.66 | PASS | MODBASE |
| HP1293 | 86.7 | 13.3 | 0.0 | 0.0 | 100 | 0.0 | FAIL | PDB |
| HP1294 | 85.8 | 8.2 | 4.4 | 1.6 | 94.0 | 84.13 | PASS | MODBASE |
| HP1295 | 84.4 | 12.5 | 2.1 | 1.0 | 96.9 | 78.99 | WARNING | MODBASE |
| HP1296 | 78.8 | 18.3 | 2.9 | 0.0 | 100 | 70.0 | WARNING | PHYRE2 |
| HP1297 | 46.9 | 40.6 | 6.2 | 6.2 | 87.5 | 0.0 | FAIL | PHYRE2 |
| HP1298 | 57.1 | 41.3 | 1.6 | 0.0 | 98.4 | 72.22 | WARNING | PHYRE2 |
| HP1299 | 94.3 | 4.7 | 0.9 | 0.0 | 99.0 | 97.23 | PASS | MODBASE |
| HP1300 | 71.0 | 17.1 | 8.1 | 3.8 | 96.2 | 41.63 | FAIL | PHYRE2 |
| HP1301 | 0.0 | 0.0 | 0.0 | 100.0 | 0.0 | 42.19 | FAIL | PHYRE2 |
| HP1302 | 0.0 | 0.0 | 0.0 | 100.0 | 0.0 | 25.52 | FAIL | PHYRE2 |
| HP1303 | 90.0 | 7.0 | 2.0 | 1.0 | 97.0 | 70.54 | WARNING | MODBASE |
| HP1304 | 90.6 | 6.5 | 2.9 | 0.0 | 97.1 | 100 | PASS | MODBASE |
| HP1305 | 67.5 | 25.8 | 5.0 | 1.7 | 93.3 | 83.33 | PASS | PHYRE2 |
| HP1306 | 81.6 | 14.3 | 2.0 | 2.0 | 95.9 | 56.67 | FAIL | PHYRE2 |
| HP1307 | 92.4 | 6.3 | 0.6 | 0.6 | 98.7 | 88.89 | PASS | MODBASE |
| HP1308 | 31.7 | 48.3 | 13.3 | 6.7 | 80.0 | 33.33 | FAIL | PHYRE2 |
| HP1309 | 82.2 | 16.8 | 0.9 | 0.0 | 99.0 | 98.37 | PASS | MODBASE |
| HP1310 | 8.1 | 32.4 | 25.7 | 33.8 | 40.5 | 19.75 | FAIL | PHYRE2 |
| HP1311 | 84.1 | 11.1 | 1.6 | 3.2 | 96.8 | 47.76 | FAIL | PHYRE2 |
| HP1312 | 86.1 | 7.0 | 3.5 | 3.5 | 93.1 | 83.82 | PASS | MODBASE |
| HP1313 | 87.9 | 10.4 | 1.1 | 0.5 | 98.3 | 83.57 | PASS | MODBASE |
| HP1314 | 87.1 | 10.8 | 1.1 | 1.1 | 97.9 | 81.90 | PASS | MODBASE |
| HP1315 | 72.7 | 22.1 | 3.9 | 1.3 | 98.7 | 58.06 | FAIL | PHYRE2 |
| HP1316 | 30.0 | 41.4 | 20.0 | 8.6 | 71.4 | 71.64 | WARNING | PHYRE2 |
| HP1317 | 0.0 | 0.0 | 0.0 | 100.0 | 0.0 | 0.0 | FAIL | PHYRE2 |
| HP1318 | 72.8 | 21.5 | 3.7 | 2.1 | 94.3 | 46.19 | FAIL | PHYRE2 |
| HP1319 | 52.8 | 34.2 | 9.9 | 3.1 | 87.0 | 60.94 | FAIL | PHYRE2 |
| HP1320 | 70.5 | 19.3 | 5.7 | 4.5 | 89.8 | 45.45 | FAIL | PHYRE2 |
| HP1321 | 86.2 | 10.7 | 1.3 | 1.8 | 96.9 | 41.83 | FAIL | ITASSER |
| HP1322 | 80.2 | 17.0 | 0.9 | 1.9 | 97.2 | 4.96 | FAIL | PHYRE2 |
| HP1323 | 92.2 | 6.6 | 0.0 | 1.2 | 98.8 | 68.75 | WARNING | PHYRE2 |
| HP1324 | 89.6 | 7.5 | 3.0 | 0.0 | 97.1 | 22.67 | FAIL | PHYRE2 |
| HP1325 | 94.6 | 4.7 | 0.5 | 0.2 | 99.3 | 80.96 | PASS | MODBASE |
| HP1326 | 81.7 | 15.9 | 1.2 | 1.2 | 97.6 | 55.67 | FAIL | PHYRE2 |
| HP1327 | 92.2 | 6.1 | 1.1 | 0.6 | 98.3 | 0 | FAIL | PHYRE2 |
| HP1328 | 87.9 | 9.1 | 1.5 | 1.5 | 97.0 | 48.62 | FAIL | PHYRE2 |
| HP1329 | 70.1 | 28.0 | 2.0 | 0.0 | 98.1 | 37.84 | FAIL | PHYRE2 |
| HP1330 | 78.4 | 14.9 | 4.1 | 2.7 | 93.3 | 0.0 | FAIL | PHYRE2 |
| HP1331 | 83.5 | 14.2 | 1.1 | 1.1 | 97.7 | 21.35 | FAIL | PHYRE2 |
| HP1332 | 74.9 | 16.8 | 4.2 | 4.2 | 91.7 | 73.3 | WARNING | PHYRE2 |
| HP1333 | 90.5 | 8.1 | 1.1 | 0.4 | 98.6 | 0 | FAIL | PHYRE2 |
| HP1334 | 92.4 | 5.6 | 0.7 | 1.4 | 98.0 | 48.41 | FAIL | PHYRE2 |
| HP1335 | 88.2 | 9.2 | 1.6 | 1.0 | 97.4 | 81.74 | PASS | PHYRE2 |
| HP1336 | 77.6 | 19.4 | 1.5 | 1.5 | 97.0 | 51.35 | FAIL | PHYRE2 |
| HP1337 | 91.0 | 8.3 | 0.7 | 0.0 | 99.3 | 67.68 | WARNING | PHYRE2 |
| HP1338 | 93.6 | 5.6 | 0.8 | 0.0 | 99.2 | 100 | PASS | PDB |
| HP1339 | 75.9 | 17.3 | 4.5 | 2.3 | 93.2 | 15.54 | FAIL | PHYRE2 |
| HP1340 | 70.9 | 24.4 | 2.3 | 2.3 | 95.3 | 34.41 | FAIL | PHYRE2 |
| HP1341 | 91.9 | 5.4 | 1.4 | 1.4 | 97.3 | 33.33 | FAIL | PHYRE2 |
| HP1342 | 77.6 | 15.5 | 6.2 | 0.7 | 93.1 | 14.15 | FAIL | PHYRE2 |
| HP1343 | 85.9 | 10.5 | 2.1 | 1.6 | 96.4 | 17.54 | FAIL | PHYRE2 |
| HP1344 | 74.1 | 22.6 | 2.6 | 0.8 | 96.7 | 50.17 | FAIL | PHYRE2 |
| HP1345 | 90.4 | 9.3 | 0.0 | 0.3 | 99.7 | 84.77 | PASS | MODBASE |
| HP1346 | 90.8 | 7.9 | 0.7 | 0.7 | 98.7 | 79.39 | WARNING | MODBASE |
| HP1347 | 85.2 | 9.9 | 3.4 | 1.5 | 95.1 | 94.87 | PASS | MODBASE |
| HP1348 | 85.1 | 12.8 | 1.4 | 0.7 | 97.9 | 60.36 | FAIL | PHYRE2 |
| HP1349 | 86.7 | 10.0 | 3.3 | 0.0 | 96.7 | 0 | FAIL | PHYRE2 |
| HP1350 | 88.3 | 10.0 | 1.7 | 0.7 | 98.3 | 78.72 | WARNING | PHYRE2 |
| HP1351 | 88.7 | 10.5 | 0.8 | 0.0 | 99.2 | 37.84 | FAIL | PHYRE2 |
| HP1352 | 90.8 | 7.4 | 0.5 | 1.4 | 98.2 | 37.55 | FAIL | PHYRE2 |
| HP1353 | 83.0 | 13.6 | 2.0 | 1.4 | 95.6 | 34.85 | PASS | PHYRE2 |
| HP1354 | 80.6 | 15.9 | 2.9 | 0.6 | 96.5 | 54.30 | FAIL | PHYRE2 |
| HP1355 | 90.1 | 9.1 | 0.8 | 0.0 | 99.2 | 76.28 | WARNING | PDB |
| HP1356 | 88.9 | 9.8 | 0.3 | 1.0 | 98.7 | 88.64 | PASS | PHYRE2 |
| HP1357 | 80.2 | 15.3 | 2.3 | 2.3 | 95.5 | 83.55 | PASS | PHYRE2 |
| HP1358 | 96.6 | 3.4 | 0.0 | 0.0 | 100 | 0.81 | FAIL | PHYRE2 |
| HP1359 | 75.7 | 16.2 | 5.4 | 2.7 | 91.9 | 0.0 | FAIL | PHYRE2 |
| HP1360 | 68.9 | 27.2 | 3.1 | 0.8 | 96.1 | 22.84 | FAIL | PHYRE2 |
| HP1361 | 62.7 | 33.5 | 2.7 | 1.1 | 96.2 | 16.67 | FAIL | PHYRE2 |
| HP1362 | 90.6 | 7.5 | 1.9 | 0.0 | 98.1 | 85.77 | PASS | PDB |
| HP1363 | 86.7 | 11.1 | 1.2 | 1.0 | 97.8 | 91.87 | PASS | PDB |
| HP1364 | 84.5 | 10.7 | 2.9 | 1.9 | 95.2 | 40.99 | FAIL | PHYRE2 |
| HP1365 | 93.9 | 5.3 | 0.0 | 0.9 | 99.2 | 85.37 | PASS | MODBASE |
| HP1366 | 79.4 | 17.4 | 1.7 | 1.5 | 96.8 | 13.51 | FAIL | PHYRE2 |
| HP1367 | 88.2 | 10.9 | 0.8 | 0.0 | 99.1 | 69.50 | WARNING | MODBASE |
| HP1368 | 83.9 | 13.4 | 1.8 | 0.9 | 97.3 | 54.44 | FAIL | PHYRE2 |
| HP1369 | 87.1 | 11.8 | 1.2 | 0.0 | 98.9 | 43.96 | FAIL | PHYRE2 |
| HP1370 | 89.2 | 8.4 | 2.4 | 0.0 | 97.6 | 28.57 | FAIL | ITASSER |
| HP1371 | 84.3 | 12.4 | 1.8 | 1.5 | 96.7 | 28.6 | FAIL | PHYRE2 |
| HP1372 | 80.4 | 16.7 | 2.4 | 0.6 | 97.1 | 56.32 | FAIL | PHYRE2 |
| HP1373 | 92.3 | 7.3 | 0.0 | 0.3 | 99.6 | 91.32 | PASS | MODBASE |
| HP1374 | 86.8 | 12.9 | 0.3 | 0.0 | 99.7 | 85.06 | PASS | PDB |
| HP1375 | 85.6 | 14.4 | 0.0 | 0.0 | 100 | 95 | PASS | PDB |
| HP1376 | 84.9 | 14.8 | 0.3 | 0.0 | 99.7 | 100 | PASS | PDB |
| HP1377 | 84.9 | 13.5 | 0.0 | 1.6 | 98.4 | 74.13 | WARNING | PHYRE2 |
| HP1378 | 92.8 | 5.4 | 1.8 | 0.0 | 98.2 | 56.28 | FAIL | PHYRE2 |
| HP1379 | 73.7 | 21.0 | 3.4 | 2.0 | 94.7 | 47.16 | FAIL | PHYRE2 |
| HP1380 | 86.0 | 12.3 | 1.3 | 0.4 |  | 44.47 | FAIL | PHYRE2 |
| HP1381 | 87.5 | 4.2 | 4.2 | 4.2 | 91.7 | 0.0 | FAIL | PHYRE2 |
| HP1382 | 90.0 | 8.0 | 2.0 | 0.0 | 98.0 | 49.09 | FAIL | PHYRE2 |
| HP1383 | 89.7 | 7.4 | 2.2 | 0.7 | 97.1 | 6.49 | FAIL | PHYRE2 |
| HP1384 | 85.4 | 10.4 | 4.2 | 0.0 | 95.8 | 0.0 | FAIL | PHYRE2 |
| HP1385 | 85.0 | 11.0 | 2.8 | 1.2 | 96.0 | 61.90 | FAIL | PHYRE2 |
| HP1386 | 93.7 | 5.8 | 0.0 | 0.5 | 99.5 | 96.77 | PASS | MODBASE |
| HP1387 | 85.1 | 12.3 | 0.6 | 1.9 | 97.4 | 46.2 | FAIL | PHYRE2 |
| HP1388 | 84.9 | 13.7 | 1.4 | 0.0 | 98.6 | 0.0 | FAIL | PHYRE2 |
| HP1389 | 94.6 | 5.4 | 0.0 | 0.0 | 100 | 68.85 | FAIL | PHYRE2 |
| HP1390 | 91.1 | 7.6 | 1.3 | 0.0 | 98.7 | 0.0 | FAIL | PHYRE2 |
| HP1391 | 92.3 | 7.7 | 0.0 | 0.0 | 100 | 0 | FAIL | PHYRE2 |
| HP1392 | 83.2 | 15.8 | 1.0 | 0.0 | 99.0 | 1.44 | FAIL | PHYRE2 |
| HP1393 | 79.9 | 15.8 | 3.9 | 0.4 | 95.7 | 72.03 | WARNING | PHYRE2 |
| HP1394 | 88.4 | 9.3 | 1.8 | 0.4 | 97.7 | 82.44 | PASS | PHYRE2 |
| HP1395 | 83.9 | 12.6 | 2.8 | 0.7 | 96.5 | 19.3 | FAIL | PHYRE2 |
| HP1396 | 80.1 | 19.1 | 0.4 | 0.4 | 99.2 | 52.31 | FAIL | PHYRE2 |
| HP1397 | 83.5 | 14.6 | 1.9 | 0.0 | 98.1 | 11.21 | FAIL | PHYRE2 |
| HP1398 | 90.9 | 6.7 | 1.8 | 0.6 | 97.6 | 80.21 | PASS | MODBASE |
| HP1399 | 91.4 | 7.8 | 0.2 | 0.5 | 99.5 | 0.0 | FAIL | PDB |
| HP1400 | 74.2 | 23.3 | 1.2 | 1.2 | 97.5 | 67.95 | WARNING | PHYRE2 |
| HP1401 | 87.1 | 11.0 | 1.3 | 0.6 | 98.1 | 37.07 | FAIL | PHYRE2 |
| HP1402 | 73.5 | 21.0 | 3.7 | 1.7 | 94.5 | 43.38 | FAIL | PHYRE2 |
| HP1403 | 79.9 | 17.0 | 1.9 | 1.2 | 96.9 | 63.45 | FAIL | PHYRE2 |
| HP1404 | 86.5 | 10.8 | 1.4 | 1.4 | 97.3 | 48.35 | FAIL | PHYRE2 |
| HP1405 | 78.9 | 15.8 | 5.3 | 0.0 | 94.7 | 0 | FAIL | PHYRE2 |
| HP1406 | 77.2 | 19.1 | 2.8 | 0.8 | 96.3 | 82.91 | PASS | PHYRE2 |
| HP1407 | 80.6 | 15.6 | 1.7 | 2.1 | 96.2 | 35.34 | FAIL | PHYRE2 |
| HP1408 | 100.0 | 0.0 | 0.0 | 0.0 | 100 | 0.0 | FAIL | PHYRE2 |
| HP1409 | 86.4 | 10.5 | 1.0 | 1.1 | 96.9 | 49.05 | FAIL | PHYRE2 |
| HP1410 | 79.8 | 17.4 | 0.9 | 1.9 | 97.2 | 59.13 | FAIL | PHYRE2 |
| HP1411 | 81.4 | 15.8 | 2.1 | 0.6 | 97.2 | 17.43 | FAIL | PHYRE2 |
| HP1412 | 84.2 | 12.9 | 1.0 | 2.0 | 97.1 | 33.18 | FAIL | PHYRE2 |
| HP1413 | 87.9 | 8.9 | 3.2 | 0.0 | 96.8 | 46.53 | FAIL | PHYRE2 |
| HP1414 | 92.2 | 6.9 | 1.0 | 0.0 | 99.1 | 58.04 | FAIL | PHYRE2 |
| HP1415 | 90.2 | 7.7 | 1.6 | 0.5 | 97.9 | 42.50 | FAIL | PHYRE2 |
| HP1416 | 86.3 | 11.8 | 1.5 | 0.4 | 98.1 | 62.64 | FAIL | PHYRE2 |
| HP1417 | NO SEQUENCE |  |  |  |  |  |  |  |
| HP1418 | 85.9 | 10.5 | 3.2 | 0.5 | 96.4 | 72.87 | WARNING | PHYRE2 |
| HP1419 | 68.3 | 25.4 | 3.2 | 3.2 | 93.7 | 0.0 | FAIL | PHYRE2 |
| HP1420 | 89.6 | 8.4 | 2.0 | 0.0 | 98.0 | 86.60 | PASS | MODBASE |
| HP1421 | 84.4 | 12.2 | 3.0 | 0.4 | 96.6 | 79.32 | WARNING | PHYRE2 |
| HP1422 | 83.7 | 12.7 | 1.7 | 1.9 | 96.4 | 81.92 | PASS | MODBASE |
| HP1423 | 73.0 | 27.0 | 0.0 | 0.0 | 100 | 0 | FAIL | PDB |
| HP1424 | 81.2 | 18.8 | 0.0 | 0.0 | 100 | 15.29 | FAIL | PHYRE2 |
| HP1425 | 71.1 | 26.3 | 0.0 | 2.6 | 97.4 | 0.0 | FAIL | PHYRE2 |
| HP1426 | 79.2 | 15.6 | 1.3 | 3.9 | 94.8 | 65.96 | WARNING | PHYRE2 |
| HP1427 | 75.0 | 25.0 | 0.0 | 0.0 | 100 | 0.0 | FAIL | PHYRE2 |
| HP1428 | 86.0 | 11.4 | 2.6 | 0.0 | 97.4 | 47.22 | FAIL | PHYRE2 |
| HP1429 | 90.9 | 6.7 | 1.2 | 1.2 | 97.6 | 80.43 | PASS | PHYRE2 |
| HP1430 | 89.7 | 8.9 | 0.4 | 1.0 | 98.6 | 89.09 | PASS | MODBASE |
| HP1431 | 88.5 | 9.8 | 0.8 | 0.8 | 98.3 | 74.17 | WARNING | MODBASE |
| HP1432 | 76.1 | 17.9 | 0.8 | 0.0 | 94.0 | 13.94 | FAIL | PHYRE2 |
| HP1433 | 81.0 | 14.3 | 3.0 | 1.7 | 95.3 | 74.7 | WARNING | PHYRE2 |
| HP1434 | 89.6 | 10.0 | 0.4 | 0.0 | 98.5 | 67.58 | WARNING | PHYRE2 |
| HP1435 | 86.6 | 10.2 | 2.3 | 0.9 | 96.8 | 54.13 | FAIL | PHYRE2 |
| HP1436 | 90.0 | 10.0 | 0.0 | 0.0 | 100 | 0.0 | FAIL | PHYRE2 |
| HP1437 | 88.2 | 10.6 | 0.0 | 1.2 | 98.8 | 58.43 | FAIL | PHYRE2 |
| HP1438 | 90.2 | 8.2 | 1.5 | 0.0 | 98.4 | .48 | FAIL | PHYRE2 |
| HP1439 | 91.8 | 8.2 | 0.0 | 0.0 | 100 | 0.0 | FAIL | PHYRE2 |
| HP1439.1 | 87.6 | 11.2 | 1.2 | 0.0 | 98.8 | 49.47 | FAIL | PHYRE2 |
| HP1440 | 87.6 | 11.2 | 1.2 | 0.0 | 98.8 | 49.47 | FAIL | PHYRE2 |
| HP1441 | 85.8 | 13.3 | 0.8 | 0.0 | 99.1 | 90.48 | PASS | MODBASE |
| HP1442 | 60.9 | 30.4 | 2.9 | 5.8 | 91.3 | 15.79 | FAIL | PHYRE2 |
| HP1443 | 84.5 | 12.5 | 3.0 | 0.0 | 97.0 | 57.25 | FAIL | PHYRE2 |
| HP1444 | 76.5 | 16.5 | 3.5 | 3.5 | 93.0 | 85.60 | PASS | PHYRE2 |
| HP1445 | 74.5 | 16.7 | 4.9 | 3.9 | 91.2 | 6.84 | FAIL | PHYRE2 |
| HP1446 | 75.0 | 21.7 | 2.2 | 1.1 | 96.7 | 15.0 | FAIL | PHYRE2 |
| HP1447 | 0.0 | 0.0 | 0.0 | 100.0 | 0.0 | 0.0 | FAIL | PHYRE2 |
| HP1448 | 71.4 | 22.9 | 4.8 | 1.0 | 94.3 | 81.74 | PASS | PHYRE2 |
| HP1449 | 70.4 | 22.2 | 3.7 | 3.7 | 92.6 | 0.0 | FAIL | PHYRE2 |
| HP1450 | 82.8 | 13.3 | 2.1 | 1.7 | 96.1 | 69.32 | WARNING | PHYRE2 |
| HP1451 | 91.2 | 8.1 | 0..7 | 0.0 | 99.3 | 0 | FAIL | PDB |
| HP1452 | 88.6 | 8.8 | 2.4 | 0.2 | 97.4 | 61.84 | FAIL | PHYRE2 |
| HP1453 | 100 | 0 | 0 | 0 | 100 | 0 | FAIL | PHYRE2 |
| HP1454 | 77.7 | 18.1 | 3.6 | 0.9 | 99.1 | 65.43 | WARNING | PDB |
| HP1455 | 77.1 | 19.3 | 1.2 | 2.4 | 96.4 | 17.78 | FAIL | PHYRE2 |
| HP1456 | 89.1 | 8.7 | 2.2 | 0.0 | 97.8 | 0.0 | FAIL | PHYRE2 |
| HP1457 | 85.2 | 12.2 | 0.0 | 2.6 | 97.4 | 66.15 | WARNING | PHYRE2 |
| HP1458 | 92.3 | 5.5 | 0.0 | 2.2 | 97.8 | 85.38 | PASS | MODBASE |
| HP1459 | 82.1 | 17.0 | 0.5 | 0.5 | 99.7 | 75.43 | WARNING | PHYRE2 |
| HP1460 | 71.4 | 22.9 | 4.8 | 1.0 | 94.3 | 0/70.76 | WARNING | PHYRE2 |
| HP1461 | 87.8 | 11.9 | 0.0 | 0.4 | 99.7 | 69.91 | WARNING | MODBASE |
| HP1462 | 77.1 | 19.3 | 2.4 | 1.2 | 96.4 | 6.38 | FAIL | PHYRE2 |
| HP1463 | 84.3 | 14.0 | 1.7 | 0.0 | 98.3 | 59.79 | FAIL | PHYRE2 |
| HP1464 | 95.5 | 4.5 | 0.0 | 0.0 | 100 | 0 | FAIL | PHYRE2 |
| HP1465 | 88.7 | 10.3 | 0.5 | 0.5 | 99.0 | 69.36 | WARNING | MODBASE |
| HP1466 | 86.9 | 10.7 | 0.0 | 2.4 | 97.6 | 45.16 | FAIL | PHYRE2 |
| HP1467 | 91.0 | 7.5 | 0.7 | 0.7 | 98.5 | 41.46 | FAIL | PHYRE2 |
| HP1468 | 89.8 | 7.5 | 2.0 | 0.7 | 97.3 | 86.76 | PASS | MODBASE |
| HP1469 | 90.2 | 6.7 | 1.2 | 1.8 | 96.9 | 20.74 | FAIL | PHYRE2 |
| HP1470 | 79.8 | 15.8 | 2.2 | 2.2 | 95.6 | 78.09 | WARNING | PHYRE2 |
| HP1471 | 87.1 | 8.9 | 2.2 | 1.8 | 96.0 | 60.16 | FAIL | PHYRE2 |
| HP1472 | 82.8 | 14.2 | 2.4 | 0.5 | 97.0 | 47.92 | FAIL | PHYRE2 |
| HP1473 | 79.7 | 16.3 | 2.4 | 1.6 | 96.0 | 63.19 | FAIL | PHYRE2 |
| HP1474 | 89.0 | 7.0 | 1.7 | 2.3 | 96.0 | 59.57 | FAIL | PHYRE2 |
| HP1475 | 95.4 | 4.6 | 0.0 | 0.0 | 100 | 97.77 | PASS | PDB |
| HP1476 | 89.5 | 7.4 | 2.5 | 0.6 | 96.9 | 73.26 | WARNING | MODBASE |
| HP1477 | 86.3 | 7.8 | 3.9 | 2.0 | 94.1 | 76.27 | WARNING | PHYRE2 |
| HP1478 | 88.2 | 9.2 | 1.7 | 0.9 | 97.4 | 73.66 | WARNING | MODBASE |
| HP1479 | 81.5 | 15.5 | 1.9 | 1.1 | 97.0 | 68.56 | WARNING | PHYRE2 |
| HP1480 | 91.3 | 7.6 | 0.3 | 0.8 | 98.9 | 80.77 | PASS | MODBASE |
| HP1481 | 85.5 | 12.8 | 1.3 | 0.4 | 98.3 | 66.41 | WARNING | PHYRE2 |
| HP1482 | 94.5 | 3.6 | 1.8 | 0.0 | 98.1 | 0.0 | FAIL | PHYRE2 |
| HP1483 | 83.1 | 14.0 | 2.3 | 0.6 | 97.1 | 64.92 | FAIL | PHYRE2 |
| HP1484 | 90.7 | 6.2 | 3.1 | 0.0 | 96.9 | 0.92 | FAIL | PHYRE2 |
| HP1485 | 81.7 | 16.6 | 1.2 | 0.6 | 98.3 | 86.70 | PASS | PHYRE2 |
| HP1486 | 81.6 | 15.5 | 2.4 | 0.5 | 97.1 | 44.93 | FAIL | PHYRE2 |
| HP1487 | 86.6 | 10.1 | 2.5 | 0.8 | 96.7 | 50.77 | FAIL | PHYRE2 |
| HP1488 | 89.8 | 9.3 | 0.5 | 0.5 | 99.0 | 64.88 | FAIL | PHYRE2 |
| HP1489 | 93.5 | 4.7 | 1.3 | 0.5 | 98.2 | 24.03 | FAIL | PHYRE2 |
| HP1490 | 87.5 | 8.7 | 1.0 | 2.9 | 96.2 | 47.01 | FAIL | PHYRE2 |
| HP1491 | 68.7 | 28.2 | 2.1 | 1.1 | 96.9 | 25.31 | FAIL | PHYRE2 |
| HP1492 | 68.4 | 27.6 | 1.3 | 2.6 | 97.4 | 29.07 | FAIL | PHYRE2 |
| HP1493 | 85.4 | 13.4 | 0.6 | 0.6 | 98.8 | 23.95 | FAIL | PHYRE2 |
| HP1494 | 85.1 | 10.3 | 3.0 | 1.5 | 95.4 | 68.60 | WARNING | MODBASE |
| HP1495 | 91.8 | 7.2 | 0.7 | 0.3 | 99.0 | 86.44 | PASS | MODBASE |
| HP1496 | 84.0 | 12.8 | 3.2 | 0.0 | 96.8 | 84.27 | PASS | PHYRE2 |
| HP1497 | 87.5 | 10.0 | 0.6 | 1.9 | 97.5 | 78.57 | WARNING | MODBASE |
| HP1498 | 82.9 | 15.2 | 1.0 | 1.0 | 98.1 | 0 | FAIL | PHYRE2 |
| HP1499 | 80.4 | 17.5 | 1.4 | 0.7 | 97.9 | 73.89 | WARNING | PHYRE2 |
| HP1500 | SMALL-23 |  |  |  |  |  |  |  |
| HP1501 | 81.9 | 14.4 | 3.7 | 0.0 | 96.3 | 30.35 | FAIL | PHYRE2 |
| HP1502 | 0.0 | 0.0 | 0.0 | 100.0 | 0.0 | 0.0 | FAIL | PHYRE2 |
| HP1503 | 73.8 | 18.1 | 5.1 | 3.0 | 91.9 | 35.18 | FAIL | PHYRE2 |
| HP1504 | 87.7 | 10.4 | 0.6 | 1.2 | 98.1 | 75.66 | WARNING | PHYRE2 |
| HP1505 | 85.0 | 11.5 | 2.1 | 1.4 | 96.5 | 59.48 | FAIL | PHYRE2 |
| HP1506 | 83.9 | 11.6 | 3.3 | 1.2 | 95.5 | 57.73 | FAIL | PHYRE2 |
| HP1507 | 87.9 | 10.0 | 1.5 | 0.6 | 97.9 | 50.52 | FAIL | PHYRE2 |
| HP1508 | 89.7 | 9.3 | 0.0 | 0.9 | 99.0 | 67.52 | WARNING | PHYRE2 |
| HP1509 | 84.8 | 11.6 | 2.4 | 1.2 | 96.4 | 0 | FAIL | PHYRE2 |
| HP1510 | 87.1 | 8.9 | 2.0 | 2.0 | 96.0 | 58.18 | FAIL | PHYRE2 |
| HP1511 | 86.2 | 10.8 | 3.1 | 0.0 | 97.0 | 32.88 | FAIL | PHYRE2 |
| HP1512 | 72.7 | 22.5 | 3.7 | 1.2 | 95.2 | 50.43 | FAIL | PHYRE2 |
| HP1513 | 84.9 | 11.5 | 1.8 | 1.8 | 96.4 | 72.51 | WARNING | PHYRE2 |
| HP1514 | 81.7 | 15.5 | 2.5 | 0.3 | 97.2 | 45.53 | FAIL | PHYRE2 |
| HP1515 | SMALL 30 |  |  |  |  |  |  |  |
| HP1516 | 88.6 | 7.2 | 1.8 | 2.4 | 95.8 | 16.85 | FAIL | PHYRE2 |
| HP1517 | TOO LONG |  |  |  |  |  |  |  |
| HP1518 | 85.5 | 12.0 | 1.2 | 1.2 | 97.5 | 0.0 | FAIL | PHYRE2 |
| HP1519 | 89.3 | 4.9 | 3.9 | 1.9 | 94.2 | 12.61 | FAIL | PHYRE2 |
| HP1520 | 81.9 | 14.3 | 2.7 | 1.1 | 96.2 | 29.7 | FAIL | PHYRE2 |
| HP1521 | 69.6 | 23.4 | 4.2 | 2.8 | 93.0 | 30.1 | FAIL | PHYRE2 |
| HP1522 | NO SEQ |  |  |  |  |  |  |  |
| HP1523 | 63.8 | 27.4 | 7.2 | 1.6 | 91.2 | 54.71 | FAIL | PHYRE2 |
| HP1524 | 82.6 | 13.0 | 0.0 | 4.3 | 95.6 | 0.0 | FAIL | PHYRE2 |
| HP1525 | 76.5 | 17.4 | 2.7 | 3.4 | 93.9 | 25.0 | FAIL | PHYRE2 |
| HP1526 | 89.4 | 8.8 | 1.8 | 0.0 | 98.2 | 88.40 | PASS | MODBASE |
| HP1527 | 84.6 | 15.4 | 0.0 | 0.0 | 100 | 0 | FAIL | PHYRE2 |
| HP1528 | SMALL-25 |  |  |  |  |  |  |  |
| HP1529 | 83.5 | 13.2 | 2.2 | 1.0 | 96.7 | 95.11 | PASS | PDB |
| HP1530 | 81.5 | 14.2 | 1.9 | 2.5 | 95.7 | 38.20 | FAIL | PHYRE2 |
| HP1531 | 97.7 | 2.3 | 0.0 | 0.0 | 100 | 100 | PASS | PDB |
| HP1532 | 67.1 | 27.1 | 3,8 | 2.0 | 94.2 | 83.75 | PASS | PHYRE2 |
| HP1533 | 91.1 | 8.8 | 0.1 | 0.0 | 99.9 | 98.33 | PASS | PDB |
| HP1534 | 91.3 | 7.2 | 1.4 | 0.0 | 98.5 | 7.92 | FAIL | PHYRE2 |
| HP1535 | 88.6 | 8.9 | 1.6 | 0.8 | 97.5 | 64.39 | PASS | PHYRE2 |
| HP1536 | TOO SMALL(>20) |  |  |  |  |  |  |  |
| HP1537 | 84.6 | 12.8 | 0.7 | 2.0 | 97.4 | 39.29 | FAIL | PHYRE2 |
| HP1538 | 82.4 | 13.2 | 3.1 | 1.3 | 95.6 | 66.67 | WARNING | PHYRE2 |
| HP1539 | 86.5 | 11.4 | 1.8 | 0.3 | 97.9 | 49.16 | FAIL | PHYRE2 |
| HP1540 | 55.8 | 34.2 | 8.3 | 1.7 | 90.0 | 25.52 | FAIL | PHYRE2 |
| HP1541 | 80.6 | 16.5 | 2.5 | 0.4 | 97.1 | 68.69 | WARNING | PHYRE2 |
| HP1542 | 81.5 | 14.1 | 4.3 | 0.0 | 95.6 | 60.34 | FAIL | PHYRE2 |
| HP1543 | 82.8 | 13.1 | 3.4 | 0.7 | 95.9 | 50.29 | FAIL | PHYRE2 |
| HP1544 | 84.8 | 9.5 | 4.4 | 1.3 | 94.3 | 49.2 | FAIL | PHYRE2 |
| HP1545 | 89.0 | 10.2 | 0.3 | 0.6 | 99.2 | 85.49 | PASS | PHYRE2 |
| HP1546 | 83.9 | 15.3 | 0.8 | 0.0 | 99.2 | 16.54 | FAIL | PHYRE2 |
| HP1547 | 87.9 | 10.0 | 1.1 | 1.0 | 97.9 | 77.02 | WARNING | MODBASE |
| HP1548 | 67.2 | 22.4 | 7.5 | 3.0 | 89.6 | 0.0 | FAIL | PHYRE2 |
| HP1549 | 75.3 | 18.3 | 2.8 | 3.6 | 93.6 | 15.44 | FAIL | PHYRE2 |
| HP1550 | 75.2 | 19.1 | 4.6 | 1.1 | 94.3 | 36.65 | FAIL | PHYRE2 |
| HP1551 | 76.9 | 23.1 | 0.0 | 0.0 | 100 | 0.0 | FAIL | PHYRE2 |
| HP1552 | 67.9 | 26.2 | 4.6 | 1.2 | 94.1 | 48.93 | FAIL | PHYRE2 |
| HP1553 | 78.9 | 16.6 | 3.2 | 1.3 | 95.5 | 41.95 | FAIL | PHYRE2 |
| HP1554 | 79.1 | 16.3 | 3.1 | 1.5 | 95.4 | 81.74 | PASS | MODBASE |
| HP1555 | 89.0 | 9.2 | 1.1 | 0.7 | 98.2 | 70.36 | WARNING | PHYRE2 |
| HP1556 | 80.7 | 16.7 | 1.3 | 1.3 | 97.4 | 69.94 | WARNING | PHYRE2 |
| HP1557 | 90.2 | 8.2 | 1.6 | 0.0 | 100 | 0.0 | FAIL | PHYRE2 |
| HP1558 | 85.6 | 11.4 | 1.5 | 1.5 | 98.5 | 5.61 | FAIL | PHYRE2 |
| HP1559 | 84.4 | 15.6 | 0 | 0 | 100 | 0.0 | FAIL | PHYRE2 |
| HP1560 | 83.3 | 16.7 | 0.0 | 0.O | 100 | 38.04 | FAIL | PHYRE2 |
| HP1561 | 91.7 | 7.2 | 1.1 | 0.0 | 100 | 99.67 | PASS | PDB |
| HP1562 | 82.8 | 13.8 | 2.7 | 0.8 | 96.6 | 81.76 | PASS | ITASSER |
| HP1563 | 77.2 | 20.2 | 2.6 | 0.0 | 100 | 0.0 | FAIL | PDB |
| HP1564 | 89.0 | 9.1 | 1.9 | 0.0 | 98.1 | 95.36 | PASS | MODBASE |
| HP1565 | 87.2 | 10.6 | 1.6 | 0.7 | 97.8 | 71.21 | WARNING | ITASSER |
| HP1566 | 92.9 | 5.9 | 0.0 | 1.2 | 98.8 | 16.83 | FAIL | PHYRE2 |
| HP1567 | 86.0 | 12.9 | 1.1 | 0.0 | 98.9 | 80.51 | PASS | ITASSER |
| HP1568 | 74.4 | 20.8 | 2.4 | 2.4 | 97.6 | 72.14 | WARNING | PHYRE2 |
| HP1569 | 82.1 | 16.7 | 1.2 | 0.0 | 98.8 | 32.98 | FAIL | PHYRE2 |
| HP1570 | 95.9 | 4.1 | 0.0 | 0.0 | 100 | 79.88 | WARNING | MODBASE |
| HP1571 | 91.2 | 2.9 | 5.9 | 0.0 | 94.1 | 94.81 | PASS | PHYRE2 |
| HP1572 | 91.5 | 6.5 | 2.0 | 0.0 | 98.0 | 50.19 | FAIL | PHYRE2 |
| HP1573 | 89.7 | 8.0 | 1.3 | 0.9 | 97.7 | 85.32 | PASS | MODBASE |
| HP1574 | 91.0 | 8.4 | 0.0 | 0.6 | 99.6 | 85.05 | PASS | MODBASE |
| HP1575 | 83.7 | 10.2 | 6.1 | 0.6 | 93.9 | 0.0 | FAIL | PHYRE2 |
| HP1576 | 89.8 | 8.8 | 1.4 | 0.0 | 98.6 | 79.38 | WARNING | MODBASE |
| HP1577 | 88.1 | 10.8 | 0.6 | 0.6 | 98.9 | 30.69 | FAIL | PHYRE2 |
| HP1578 | 89.8 | 7.6 | 2.1 | 0.4 | 97.4 | 55.56 | FAIL | PHYRE2 |
| HP1579 | 95.8 | 4.2 | 0.0 | 0.0 | 100 | 13.71 | FAIL | PHYRE2 |
| HP1580 | 88.2 | 8.8 | 1.5 | 1.5 | 97.0 | 0 | FAIL | PHYRE2 |
| HP1581 | 65.0 | 31.7 | 2.5 | 0.8 | 96.7 | 27.24 | FAIL | PHYRE2 |
| HP1582 | 87.5 | 10.4 | 1.7 | 0.4 | 97.9 | 80.61 | PASS | MODBASE |
| HP1583 | 90.0 | 7.7 | 1.5 | 0.7 | 97.7 | 77.20 | WARNING | MODBASE |
| HP1584 | 89.1 | 9.5 | 1.0 | 0.3 | 98.6 | 75.07 | WARNING | PHYRE2 |
| HP1585 | 87.2 | 9.9 | 2.1 | 0.7 | 97.1 | 27.84 | FAIL | PHYRE2 |
| HP1586 | 90.0 | 5.0 | 2.5 | 2.5 | 95.0 | 0.0 | FAIL | PHYRE2 |
| HP1587 | 90.3 | 8.0 | 0.9 | 0.9 | 98.3 | 36.22 | FAIL | PHYRE2 |
| HP1588 | 82.9 | 13.3 | 2.2 | 1.7 | 96.2 | 10.55 | FAIL | PHYRE2 |
| HP1589 | 90.3 | 9.7 | 0.0 | 0.0 | 100 | 20.37 | FAIL | PHYRE2 |
| HP1590 | 85.7 | 14.3 | 0.0 | 0.0 | 100 | 0.0 | FAIL | PHYRE2 |
