# Supplementary material for: Structure Based Annotation of Helicobacter pylori Strain 26695 Proteome
Source: PLoS One. 2014 Dec 30;9(12):e115020. doi: 10.1371/journal.pone.0115020 (PMC4280198; doi:10.1371/journal.pone.0115020)
Supplement: S2 Table — Pgenthreader results for the failed protein models showing template PDB_ID, confidence and P-value. (DOC) [file pone.0115020.s002.doc]

| **Supplementary Table II. List of *H. pylori* 26695 strain proteins with fold predicted using Pgenthreader** | | | | |
| --- | --- | --- | --- | --- |
| **GENE NAME** | **DESCRIPTION** | **CONFIDENCE** | **P-VALUE** | **PDB_ID** |
| HP0004 | BETA CARBONIC ANHYDRASE | CERTAIN | 2E-10 | 1EKJ |
| HP0009 | ENDOGLUCANASE 9G | CERTAIN | 2E-05 | 1G87 |
| HP0011 | chaperonin | CERTAIN | 9e-06 | 1P3H |
| HP0013 | (5-METHYLAMINOMETHYL-2-THIOURIDYLATE)-METHYLTRANSFERASE TRMU | CERTAIN | 4E-07 | 2HMA |
| HP0017 | TYPE IV SECRETORY PATHWAY VIRB4 COMPONENTS-LIKE PROTEIN | CERTAIN | 3E-11 | 4AG6 |
| HP0021 | HYDROLASE/PHON PROTEIN | HIGH | 4E-04 | 2IPB |
| HP0022 | PROTEIN IN THE ALKALINE PHOSPHATASE SUPEFAMILY/PREDICTED INTEGRAL MEMBRANE PROTEIN | CERTAIN | 2E-08 | 3LXQ |
| HP0025 | METAL BINDING PROTEIN | HIGH | 1E-03 | 3CGH |
| HP0031 | USP-LIKE PROTEIN | CERTAIN | 5E-08 | 3IDF |
| HP0034 | ASPARTATE 1-DECARBOXYLASE PRECURSOR | CERTAIN | 7E-10 | 2C45 |
| HP0035 | UNCHARACTERIZED PROTEIN HP0035 FROM HEL PYLORI | CERTAIN | 5E-07 | 3F42 |
| HP0036 | HYDROLASE/HTRA PROTEASES | CERTAIN | 3E-09 | 3MH6 |
| HP0037 | NUCLEAR TRANSPORT | CERTAIN | 7E-06 | 1WA5 |
| HP0038 | COMB8 COMPETENCE PROTEIN INVOLVED IN A TYPE IV SECRETION SYSTEM THAT MEDIATES NATURAL TRANSFORMATION | CERTAIN | 1E-07 | 2BHM |
| HP0042 | COMB10 OF THE COM TYPE IV SECRETION SYSTEM | CERTAIN | 3E-10 | 2BHV |
| HP0048 | HYPF, HYDROGENASE MATURATION FACTOR | CERTAIN | 8E-35 | 3TTC |
| HP0051 | DNA (CYTOSINE-5) METHYLASE | CERTAIN | 5E-11 | 1DCT |
| HP0050 | GAGG SITE-SPECIFIC ADENINE-METHYLTRANSFERASE INVOLVED IN TYPE II RESTRICTION/MODIFICATION SYSTEM | CERTAIN | 2E-07 | 1G60 |
| HP0054 | |  | CYTOSINE-SPECIFIC METHYLTRANSFERASE | | --- | --- | | CERTAIN | 3E-09 | 3G7U |
| HP0055 | PREDICTED SODIUM/PROLINE SYMPORTER | CERTAIN | 1E-18 | 2XQ2 |
| HP0059 | CONTRACTILE PROTEIN | CERTAIN | 1E-05 | 2EFR |
| HP0060 | CHAPERONE/ | CERTAIN | 5E-11 | 1YUW |
| HP0062 | PUTATIVE UNCHARACTERIZED PROTEIN | CERTAIN | 1E-07 | 3FX7 |
| HP0063 | CELL ADHESION/ VINCULIN ISOFORM | CERTAIN | 7E-05 | 1TR2 |
| HP0066 | DNA TRANSLOCASE FTSK/ PREDICTED ATP-BINDING PROTEIN | CERTAIN | 4E-13 | 2IUT |
| HP0068 | PROBABLE HYDROGENASE NICKEL INCORPORATION PROTEIN | CERTAIN | 1E-10 | 2HF9 |
| HP0070 | UREASE ACCESSORY PROTEIN/PREDICTED NICKEL-ION METALLOCHAPERONE PROTEIN | CERTAIN | 7E-10 | 3TJ8 |
| HP0071 | Acid-activated urea channel | CERTAIN | 3e-12 | 3UX4 |
| HP0077 | 16S RRNA | CERTAIN | 2e-14 | 3D5A |
| HP0079 | UV EXCISION REPAIR PROTEIN/ OUTER MEMBRANE PROTEIN HORA | HIGH | 4E-04 | 1OGY |
| HP0080 | CARTILAGE OLIGOMERIC MATRIX PROTEIN/ CELL ADHESION | CERTAIN | 3E-05 | 3FBY |
| HP0082 | |  | HAMP, METHYL-ACCEPTING CHEMOTAXIS PROTEIN I | | --- | --- | | CERTAIN | 3E-08 | 3LI9 |
| HP0083 | 16S RRNA/RIBOSOME | CERTAIN | 2E-09 | 2VQE |
| HP0086 | FAD-DEPENDENT ALPHA-GLYCEROPHOSPHATE OXIDASE (GLPO) | CERTAIN | 1E-06 | 2RGH |
| HP0088 | DNA-DIRECTED RNA POLYMERASE ALPHA/ MAJOR TRANSCRIPTION INITIATION FACTOR SIGMA-80 | CERTAIN | 1E-12 | 2A6H |
| HP0092 | ADENINE-SPECIFIC METHYLTRANSFERASE MBOIIA | CERTAIN | 2E-07 | 1G60 |
| HP0093 | Alpha-(1,6)-fucosyltransferase | HIGH | 6e-04 | 2DE0 |
| HP0096 | PHOSPHOGLYCERATE DEHYDROGENASE. | CERTAIN | 2E-14 | 1WWK |
| HP0099 | HAMP, METHYL-ACCEPTING CHEMOTAXIS PROTEIN I | CERTAIN | 5E-09 | 3C8C |
| HP0102 | GLYCOSYLTRANSFERASE PROTEIN | CERTAIN | 9E-09 | 3BCV |
| HP0103 | HAMP, METHYL-ACCEPTING CHEMOTAXIS PROTEIN I | CERTAIN | 4E-13 | 3ZX6 |
| HP0104 | 5'-NUCLEOTIDASE | CERTAIN | 6E-19 | 3IVE |
| HP0107 | CYSTEINE SYNTHASE | CERTAIN | 5E-13 | 2Q3B |
| HP0110 | NUCLEOTIDE EXCHANGE FACTOR GRPE | CERTAIN | 6E-09 | 1DKG |
| HP0111 | PREDICTED HEAT-INDUCIBLE TRANSCRIPTION REPRESSOR OF CLASS I HEAT SHOCK GENES | CERTAIN | 1E-05 | 1STZ |
| HP0112 | |  | L-FUCULOSE 1-PHOSPHATE ALDOLASE | | --- | --- | | CERTAIN | 5E-09 | 1E4C |
| HP0114 | HYDROLASE /BACTERIAL DYNAMIN-LIKE PROTEIN | CERTAIN | 6E-05 | 2J69 |
| HP0115 | FLAGELLIN B | CERTAIN | 3E-09 | 1IO1 |
| HP0117 | METAL BINDING PROTEIN | CERTAIN | 4E-08 | 2YX0 |
| HP0119 | CONTRACTILE PROTEIN/ GENERAL CONTROL PROTEIN GCN4 AND TROPOMYOSIN 1 | CERTAIN | 4E-05 | 2EFR |
| HP0120 | PREDICTED PHOSPHOENOLPYRUVATE SYNTHASE/ PROTEIN TRANSPORT/VIRAL PROTEIN | HIGH | 8E-04 | 2XS1 |
| HP0124 | PREDICTED TRANSLATION INITIATION FACTOR IF-3 | CERTAIN | 2E-06 | 1TIF |
| HP0125 | 23S RRNA. | HIGH | 1E-04 | 3R8S |
| HP0126 | 23S RRNA. | CERTAIN | 8E-10 | 3R8S |
| HP0129 | TRANSCRIPTION ANTITERMINATION PROTEIN NUSG | CERTAIN | 3E-05 | 2XHA |
| HP0132 | PREDICTED L-SERINE/L-THREONINE DEAMINASE | CERTAIN | 8E-09 | 2IAF |
| HP0133 | APCT TRANSPORTER | CERTAIN | 4E-08 | 3GIA |
| HP0138 | PREDICTED IRON-SULFUR PROTEIN/UNKNOWN IN PSIPRED | CERTAIN | 1E-06 | 2G40 |
| HP0139 | PREDICTED SECRETED PROTEIN/ UNKNOWN IN PSIPRED | CERTAIN | 3E-06 | 3KWL |
| HP0140 | NA+-INDEPENDENT AMINO ACID TRANSPORTER | CERTAIN | 4E-07 | 3GIA |
| HP0141 | CULLIN-DEPENDENT UBIQUITIN LIGASES. | CERTAIN | 2E-06 | 1U6G |
| HP0145 | PREDICTED CYTOCHROME C OXIDASE MONOHEME SUBUNIT | CERTAIN | 3E-11 | 3MK7 |
| HP0147 | PREDICTED CYTOCHROME C OXIDASE DIHEME SUBUNIT | CERTAIN | 8E-10 | 3MK7 |
| HP0151 | TRANSCRIPTION REPRESSOR, CELL CYCLE/ RETINOBLASTOMA-ASSOCIATED PROTEIN | HIGH | 4E-04 | 2R7G |
| HP0152 | HYPOTHETICAL PROTEIN AF1704 | CERTAIN | 9E-14 | 1ZBM |
| HP0158 | TRANSFERASE/ SENSOR PROTEIN. | HIGH | 2E-04 | 3FOS |
| HP0159 | PREDICTED LPS 1,2-GLYCOSYLTRANSFERASE | CERTAIN | 6E-11 | 1GA8 |
| HP0164 | SIGNAL-TRANSDUCING PROTEIN, HISTIDINE KINASE | CERTAIN | 2E-09 | 2C2A |
| HP0169 | COPPER HOMEOSTASIS PROTEIN/ PREDICTED COLLAGENASE | HIGH | 1E-04 | 3IWP |
| HP0170 | SIGNALING PROTEIN/ CHEMOTAXIS PROTEIN CHEY | HIGH | 9E-04 | 1KMI |
| HP0171 | POLYPEPTIDE CHAIN RELEASE FACTOR 2 | CERTAIN | 6E-20 | 1GQE |
| HP0173 | LIGASE/NUCLEAR PROTEIN/ CONJUGATING ENZYME UBC9/ PREDICTED FLAGELLAR BIOSYNTHETIC PROTEIN | HIGH | 2E-05 | 2XWU |
| HP0175 | PREDICTED PEPTIDYL-PROLYL CIS-TRANS ISOMERASE C INVOLVED IN PROTEIN MATURATION/ CHAPERONE | CERTAIN | 6E-12 | 3RFW |
| HP0180 | NITRILASE HOMOLOG 2 | CERTAIN | 3E-07 | 2W1V |
| HP0190 | PHOSPHATIDYL SERINE SYNTHASE | CERTAIN | 1E-10 | 3HSI |
| HP0193 | QUINOL:FUMARATE REDUCTASE | CERTAIN | 8E-17 | 2BS2 |
| HP0201 | FATTY ACID/PHOSPHOLIPID SYNTHESIS PROTEIN PLSX | CERTAIN | 1E-18 | 1U7N |
| HP0205 | LIGASE | CERTAIN | 7E-06 | 1LDJ |
| HP0209 | METAL BINDING PROTEIN/ PREDICTED OUTER MEMBRANE PROTEIN HOFA | CERTAIN | 8E-05 | 2JD4 |
| HP0214 | NADH-QUINONE OXIDOREDUCTASE SUBUNIT/ PREDICTED SODIUM-DEPENDENT TRANSPORTER | CERTAIN | 4E-08 | 3RKO |
| HP0215 | PREDICTED CDP-DIACYLGLYCEROL SYNTHETASE / OXIDOREDUCTASE | CERTAIN | 7E-05 | 3RKO |
| HP0221 | NIFU-LIKE PROTEIN | CERTAIN | 2E-06 | 2Z7E |
| HP0222 | HYPOTHETICAL PROTEIN HP0222 | CERTAIN | 1E-04 | 1X93 |
| HP0223 | PREDICTED DNA REPAIR PROTEIN/RECA PROTEIN | CERTAIN | 4E-08 | 1U94 |
| HP0226 | MEMBRANE PROTEIN | HIGH | 3E-04 | 1OTS |
| HP0229 | CRY4AA DELTA-ENDOTOXIN | HIGH | 8E-04 | 2C9K |
| HP0231 | OXIDOREDUCTASE | CERTAIN | 6E-16 | 3TDG |
| HP0233 | BIFUNCTIONAL GLUTATHIONYLSPERMIDINE SYNTHETASE/AMIDASE. | CERTAIN | 6E-15 | 2IO8 |
| HP0239 | GLUTAMYL-TRNA REDUCTASE. | CERTAIN | 6E-18 | 1GPJ |
| HP0244 | SENSORY HISTIDINE KINASE | CERTAIN | 6E-11 | 2C2A |
| HP0246 | PREDICTED FLAGELLAR BASAL-BODY P-RING PROTEIN /UDP-N-ACETYLGLUCOSAMINE 1-CARBOXYVINYLTRANSFERASE | HIGH | 9E-04 | 2YVW |
| HP0248 | MEMBRANE PROTEIN | CERTAIN | 1E-05 | 3BK6 |
| HP0250 | D-METHIONINE TRANSPORT SYSTEM PERMEASE PROTEIN METI | CERTAIN | 8E-17 | 3TUI |
| HP0251 | D-METHIONINE TRANSPORT SYSTEM PERMEASE PROTEIN | HIGH | 3E-04 | 3TUI |
| HP0255 | PREDICTED ADENYLOSUCCINATE SYNTHETASE INVOLVED IN DE NOVO BIOSYNTHESIS OF AMP | CERTAIN | 2E-26 | 3R7T |
| HP0259 | PROTEIN TRANSPORT/ PREDICTED LARGE SUBUNIT OF THE SINGLE-STRANDED DNA-SPECIFIC DEXODEOXYRIBONUCLEASE VII | HIGH | 8E-04 | 2XXA |
| HP0260 | TCGA SITE-SPECIFIC M6A METHYLTRANSFERASE INVOLVED IN TYPE III RESTRICTION-MODIFICATION SYSTEMS/PVUII DNA METHYLTRANSFERASE | CERTAIN | 1E-06 | 1BOO |
| HP0263 | TCGA SITE-SPECIFIC M6A METHYLTRANSFERASE INVOLVED IN TYPE III RESTRICTION-MODIFICATION SYSTEMS/PVUII DNA METHYLTRANSFERASE | CERTAIN | 1E-07 | 1BOO |
| HP0264 | CLPB PROTEIN | CERTAIN | 6E-38 | 1QVR |
| HP0265 | PREDICTED CYTOCHROME C BIOGENESIS PROTEIN//LIGASE/NUCLEAR PROTEIN | HIGH | 3E-04 | 2XWU |
| HP0267 | AMIDOHYDROLASE FAMILY PROTEIN | CERTAIN | 8E-14 | 3V7P |
| HP0269 | UNKNOWN FUNCTION(PROTEIN TM_1862 | CERTAIN | 1E-12 | 2QGK |
| HP0271 | EXOPOLYPHOSPHATASE | CERTAIN | 1E-04 | 1T6C |
| HP0275 | UDP-N-ACETYLGLUCOSAMINE--PEPTIDE N-ACETYLGLUCOSAMINYLTRANSFERASE | CERTAIN | 2E-10 | 1W3B |
| HP0276 | INDOLE-3-GLYCEROL PHOSPHATE SYNTHASE | CERTAIN | 4E-06 | 3TSM |
| HP0277 | PREDICTED FERRODOXIN | HIGH | 5E-04 | 1G87 |
| HP0278 | EXOPOLYPHOSPHATASE/GUANOSINE PENTAPHOSPHATE PHOSPHOHYDROLASE (PPX/GPPA) ENZYMES PLAY CENTRAL ROLES IN THE BACTERIAL STRINGENT RESPONSE INDUCED BY STARVATION | CERTAIN | 2E-16 | 1T6C |
| HP0280 | GLYCEROL-3-PHOSPHATE ACYLTRANSFERASE | HIGH | 8E-04 | 1IUQ |
| HP0282 | METAL TRANSPORT, MEMBRANE PROTEIN/CALCIUM-GATED POTASSIUM CHANNEL MTHK | CERTAIN | 4E-07 | 2AEF |
| HP0283 | 3-DEHYDROQUINATE SYNTHASE | CERTAIN | 2E-17 | 3OKF |
| HP0284 | MEMBRANE PROTEIN/SMALL-CONDUCTANCE MECHANOSENSITIVE CHANNEL. | CERTAIN | 2E-10 | 2W5A |
| HP0285 | BIOTIN SYNTHETASE | CERTAIN | 1E-10 | 3IIX |
| HP0286 | CELL DIVISION PROTEIN FTSH | CERTAIN | 2E-17 | 2CE7 |
| HP0291 | CHORISMATE MUTASE | CERTAIN | 4E-05 | 1ECM |
| HP0292 | CONSERVED HYPOTHETICAL PROTEIN | CERTAIN | 2E-16 | 2HQY |
| HP0293 | PROTEIN (ANTHRANILATE SYNTHASE (TRPE-SUBUNIT)). | CERTAIN | 4E-12 | 1QDL |
| HP0295 | |  | FLAGELLAR HOOK-ASSOCIATED PROTEIN 3 | | --- | --- | | CERTAIN | 2E-06 | 2D4X |
| HP0296 | RIBOSOMAL PROTEIN L21 | CERTAIN | 2E-08 | 3R8S |
| HP0299 | D-METHIONINE TRANSPORT SYSTEM PERMEASE PROTEIN | HIGH | 2E-04 | 3TUI |
| HP0300 | D-METHIONINE TRANSPORT SYSTEM PERMEASE PROTEIN | CERTAIN | 5E-05 | 3TUI |
| HP0301 | PREDICTED DIPEPTIDE TRANSPORTE SYSTEM ATP-BINDING PROTEIN | CERTAIN | 3E-18 | 3TUI |
| HP0304 | ALGINATE LYASE | CERTAIN | 8E-07 | 3NNB |
| HP0313 | GLYCEROL-3-PHOSPHATE TRANSPORTER | CERTAIN | 3E-10 | 1PW4 |
| HP0317 | GLYCEROL-3-PHOSPHATE TRANSPORTER/TOXIN | HIGH | 5E-04 | 1W99 |
| HP0322 | PREDICTED POLY E-RICH PROTEIN/STRUCTURE OF THE DNA REPAIR PROTEIN HHR23A | CERTAIN | 3E-05 | 1OQY |
| HP0323 | ENDONUCLEASE | CERTAIN | 2E-06 | 1BYR |
| HP0326 | CYTIDINE MONOPHOSPHO-N-ACETYLNEURAMINIC ACID SYNTHETASE | CERTAIN | 2E-09 | 1QWJ |
| HP0327 | ACETYLTRANSFERASE/PREDICTED FLAGELLAR BIOSYNTHESIS PROTEIN G | CERTAIN | 2E-06 | 2I79 |
| HP0328 | TETRAACYLDISACCHARIDE 4'-KINASE | CERTAIN | 5E-13 | 4EHX |
| HP0332 | PREDICTED MINE PROTEIN INVOLVED IN SEPTUM LOCALIZATION/ | HIGH | 2E-04 | 3KU7 |
| HP0334 | PUTATIVE HOLLIDAY JUNCTION RESOLVASE | CERTAIN | 9E-07 |  |
| HP0338 | LYSOZYME | HIGH | 2E-04 | 1SX7 |
| HP0339 | LYSOZYME | HIGH | 2E-04 | 1SX7 |
| HP0350 | HYDROLASE | CERTAIN | 3E-05 | 2IPB |
| HP0351 | PREDICTED FLAGELLAR BASAL-BODY M-RING PROTEIN/ PROTEIN TRANSPORT | CERTAIN | 7E-08 | 1YJ7 |
| HP0352 | PREDICTED FLAGELLAR MOTOR SWITCH PROTEIN | CERTAIN | 3E-18 | 3HJL |
| HP0353 | V-TYPE ATP SYNTHASE/PREDICTED FLAGELLAR EXPORT PROTEIN | HIGH | 7E-04 | 3K5B |
| HP0357 | PREDICTED SHORT CHAIN DEHYDROGENASE | CERTAIN | 1E-15 | 3ASV |
| HP0363 | PREDICTED L-ISOASPARTYL-PROTEIN CARBOXYL METHYLTRANSFERASE | CERTAIN | 3E-10 | 3LBF |
| HP0371 | BIOTINYL DOMAIN OF ACETYL-COENZYME A CARBOXYLASE | CERTAIN | 1E-05 | 1BDO |
| HP0373 | PREDICTED OUTER MEMBRANE PROTEIN HOMC | HIGH | 9E-04 | 1R6V |
| HP0374 | HYPOTHETICAL PROTEIN HI0303. | CERTAIN | 5E-10 | 1VHY |
| HP0377 | |  | THIOREDOXIN DISULFIDE ISOMERASE | | --- | --- |   (DSBH OXIDOREDUCTASE) | CERTAIN | 5E-06 | 2JU5 |
| HP0378 | PREDICTED BIOGENESIS PROTEIN INVOLVED IN CYTOCHROME C-TYPE MATURATION | CERTAIN | 4E-07 | 2XWU |
| HP0381 | PROTEIN METHYLTRANSFERASE HEMK | CERTAIN | 2E-11 | 2B3T |
| HP0382 | PREDICTED ZINC METALLOPROTEASE | CERTAIN | 1E-05 | 3C37 |
| HP0387 | TRANSCRIPTION-REPAIR COUPLING FACTOR | CERTAIN | 2E-08 | 2EYQ |
| HP0388 | TRANSFERASE | CERTAIN | 1E-12 | 1IM8 |
| HP0392 | SIGNAL-TRANSDUCING HISTIDINE KINASE | CERTAIN | 7E-17 | 1B3Q |
| HP0393 | RESPONSE REGULATOR RECEIVER | CERTAIN | 1E-07 | 3T6K |
| HP0394 | HYDROLASE/EXONUCLEASE | HIGH | 1E-04 | 2Q8U |
| HP0395 | UNCHARACTERIZED PROTEIN | CERTAIN | 5E-10 | 3R79 |
| HP0396 | 3-OCTAPRENYL-4-HYDROXYBENZOATE CARBOXY-LYASE | CERTAIN | 3E-25 | 2IDB |
| HP0399 | PREDICTED 30S RIBOSOMAL PROTEIN S1 | CERTAIN | 4E-08 | 3GO5 |
| HP0403 | PREDICTED PHENYLALANYL-TRNA SYNTHETASE ALPHA SUBUNIT | CERTAIN | 3E-13 | 2IY5 |
| HP0405 | SELENOCYSTEINE LYASE | CERTAIN | 1E-12 | 1JF9 |
| HP0415 | SMALL-CONDUCTANCE MECHANOSENSITIVE CHANNEL IN MEMBRANE | CERTAIN | 6E-12 | 2VV5 |
| HP0416 | PREDICTED CYCLOPROPANE FATTY ACID SYNTHASE | CERTAIN | 1E-13 | 1L1E |
| HP0419 | |  | S-ADENOSYLMETHIONINE DEPENDENT METHYLTRANSFERASE | | --- | --- | | CERTAIN | 5E-06 | 3DLI |
| HP0420 | PHENYLACETIC ACID DEGRADATION PROTEIN PAAI | CERTAIN | 3E-05 | 2FS2 |
| HP0424 | CLPB PROTEIN/CHAPERONE | HIGH | 4E-04 | 1QVR |
| HP0425 | EXONUCLEASE RECJ | CERTAIN | 3E-13 | 1IR6 |
| HP0426 | DNA BINDING PROTEIN | CERTAIN | 9E-05 | 3S4W |
| HP0427 | HYDROLASE | CERTAIN | 3E-05 | 3IBS |
| HP0428 | INTEGRIN ALPHA-X | |  | | --- |   CERTAIN | 8E-05 | 1N3Y |
| HP0431 | PREDICTED SERINE/THREONINE PHOSPHATASE 2C HOMOLOG | CERTAIN | 5E-08 | 2J82 |
| HP0432 | |  | PHOSPHORYLASE KINASE | | --- | --- | | CERTAIN | 2E-09 | 1PHK |
| HP0435 | UPF1-RNA COMPLEX/HYDROLASE | HIGH | 1E-04 | 2XZL |
| HP0437 | TRANSPOSASE | CERTAIN | 2E-05 | 2EC2 |
| HP0439 | PREDICTED DNA TRANSFORMATION COMPETENCE COMB8 HOMOLOGUE/TYPE IV SECRETION SYSTEM PROTEIN VIRB8 | CERTAIN | 8E-07 | 2BHM |
| HP0440 | |  | DNA TOPOISOMERASE I | | --- | --- | | CERTAIN | 4E-22 | 1MW9 |
| HP0441 | CONJUGAL TRANSFER PROTEIN TRWB | CERTAIN | 3E-06 | 1E9R |
| HP0447 | ATP-DEPENDENT HELICASE NAM7 | CERTAIN | 2E-10 | 2XZL |
| HP0449 | RNA-BINDING PROTEIN | HIGH | 4E-04 | 2UY1 |
| HP0452 | ATPASE RAVA(REGULATORY ATPASE VARIANT COMPLEX WITH ADP), HYDROLASE | CERTAIN | 6E-06 | 3NBX |
| HP0454 | TRANSFERASE, HYDROLASE | CERTAIN | 2E-05 | 2AXN |
| HP0459 | BACTERIAL CONJUGATION PROTEIN TRWB RESEMBLING RING HELICASES AND F1-ATPASE | CERTAIN | 3E-06 | 1E9R |
| HP0462 | TYPE I RESTRICTION-MODIFICATION ENZYME, S SUBUNIT | CERTAIN | 3E-11 | 1YF2 |
| HP0463 | TYPE I RESTRICTION-MODIFICATION SYSTEM METHYLTRANSFERASE SUBUNIT | CERTAIN | 2E-15 | 3LKD |
| HP0465 | PUTATIVE SKI2-TYPE HELICASE | CERTAIN | 8E-06 | 2ZJ8 |
| HP0469 | TYPE I RESTRICTION-MODIFICATION ENZYME | CERTAIN | 3E-11 | 1YF2 |
| HP0471 | NADH-QUINONE OXIDOREDUCTASE SUBUNIT L. | CERTAIN | 4E-07 | 3RKO |
| HP0474 | SULFATE/MOLYBDATE ABC TRANSPORTER, ATP-BINDING PROTEIN | CERTAIN | 5E-07 | 3D31 |
| HP0475 | PREDICTED MOLYBDENUM ABC TRANSPORTER, ATP-BINDING PROTEIN | CERTAIN | 5E-09 | 3D31 |
| HP0478 | TYPE IIG RESTRICTION ENDONUCLEASE | CERTAIN | 2E-08 | 3S1S |
| HP0479 | NON-FUNCTIONAL ATTAAT SITE-SPECIFIC TYPE II RESTRICTION ENDONUCLEASE IN A SILENT STATE OR DEGENERATED ENOUGH TO BE CONSIDERED AS A PSEUDOGENE | CERTAIN | 4E-07 | 2GT1 |
| HP0480 | |  | ELONGATION FACTOR G | | --- | --- | | CERTAIN | 2E-15 | 2XEX |
| HP0481 | PROTEIN (ADENINE-SPECIFIC METHYLTRANSFERASE DPNII 1) | CERTAIN | 3E-06 | 2DPM |
| HP0485 | CATALASE | CERTAIN | 4E-12 | QWL |
| HP0486 | INTEGRIN ALPHA-X | CERTAIN | 1E-04 | 3K6S |
| HP0487 | INTEGRIN ALPHAVBETA3 ECTODOMAIN PLUS AN ALPHA/BETA TRANSMEMBRANE FRAGMENT | CERTAIN | 2E-.05 | 3IJE |
| HP0488 | SECA-SIGNAL PEPTIDE COMPLEX/PROTEIN TRANSPORT | HIGH | 7E-07 | 2VDA |
| HP0490 | CALCIUM-GATED POTASSIUM CHANNEL MTHK | CERTAIN | 5E-08 | 2AEF |
| HP0491 | |  | 50S RIBOSOMAL PROTEIN L28 | | --- | --- | | CERTAIN | 5E-05 | 2JZ6 |
| HP0492 | NEURAMINYLLACTOSE-BINDING HEMAGGLUTININ HOMOLOG | CERTAIN | 1E-12 | 3BGH |
| HP0493 | |  | NADH-QUINONE OXIDOREDUCTASE SUBUNIT L | | --- | --- | | CERTAIN | 6E-06 | 3RKO |
| HP0497 | PREDICTED SODIUM- AND CHLORIDE-DEPENDENT TRANSPORTER | CERTAIN | 4E-16 | 2A65 |
| HP0499 | OUTER MEMBRANE PHOSPHOLIPASE (OMPLA) | CERTAIN | 1E-11 | 1QD6 |
| HP0505 | RESTRICTION ENDONUCLEASE PABI | CERTAIN | 2E-07 | 2DVY |
| HP0506 | PEPTIDASE DOMAIN PROTEIN | CERTAIN | 4E-13 | 3SLU |
| HP0507 | URIDINE DIPHOSPHATE GLUCOSE PYROPHOSPHATASE. | CERTAIN | 2E-08 | 3Q91 |
| HP0508 | ALPHA-2-MACROGLOBULIN RECEPTOR-ASSOCIATED PROTEIN | HIGH | 6E-04 | 2P01 |
| HP0509 | OXIDOREDUCTASE | CERTAIN | 5E-17 | 3PM9 |
| HP0513 | CULLIN-ASSOCIATED NEDD8-DISSOCIATED PROTEIN | HIGH | 2E-04 | 4A0C |
| HP0519 |  | CERTAIN | 4E-07 | 2XM6 |
| HP0524 | CAG PATHOGENICITY ISLAND PROTEIN 5/BACTERIAL CONJUGATIVE COUPLING PROTEIN TRWBDELTAN70 | CERTAIN | 2E-09 | 1E9R |
| HP0525 | CAG PATHOGENICITY ISLAND ENCODED PROTEIN/ATPASE PROTEIN | CERTAIN | 7E-18 | 2PT7 |
| HP0529 | CAG PATHOGENICITY ISLAND PROTEIN W | HIGH | 6E-04 | 1E6Y |
| HP0530 | CAG PATHOGENICITY ISLAND PROTEIN V/TYPE IV SECRETION SYSTEM PROTEIN VIRB8 | CERTAIN | 1E-05 | 2BHM |
| HP0540 | CAG PATHOGENICITY ISLAND PROTEIN I/VINCULIN ISOFORM 1 | HIGH | 3E-04 | 1TR2 |
| HP0544 | CAG PATHOGENICITY ISLAND PROTEIN E | CERTAIN | 2E-06 | 1E9R |
| HP0545 | CAG PATHOGENICITY ISLAND PROTEIN D | CERTAIN | 2E-11 | 3CWX |
| HP0552 | PREDICTED METHYLTRANSFERASE | CERTAIN | 3E-12 | 3KWP |
| HP0562 | 30S RIBOSOMAL PROTEIN S21 | HIGH | 3E-04 | 3IZV |
| HP0564 | GENE REGULATION | HIGH | 1E-04 | 2K1O |
| HP0567 | |  | TRANSPORTIN-1 | | --- | --- | | CERTAIN | 1E-06 | 4FDD |
| HP0568 | MOLYBDENUM COFACTOR BIOSYNTHESIS PROTEIN | CERTAIN | 2E-05 | 1TV8 |
| HP0572 | PREDICTED ADENINE PHOSPHORIBOSYLTRANSFERASE | CERTAIN | 5E-12 | 2DY0 |
| HP0575 | PUTATIVE ZINC METALLOPROTEASE MJ0392 | HIGH | 6E-04 | 3B4R |
| HP0576 | PREDICTED SIGNAL PEPTIDASE I | CERTAIN | 5E-08 | 1B12 |
| HP0578 | PROCESSED GLYCEROL PHOSPHATE LIPOTEICHOIC ACID SYNTHASE | CERTAIN | 4E-12 | 2W5Q |
| HP0579 | CELL DIVISION PROTEIN KINASE | HIGH | 3E-04 | 1W98 |
| HP0580 | HYDROLASE | CERTAIN | 2E-08 | 1W8O |
| HP0581 | PREDICTED DIHYDROOROTASE | CERTAIN | 2E-14 | 2Z26 |
| HP0584 | PREDICTED FLAGELLAR MOTOR SWITCH PROTEIN | CERTAIN | 3E-05 | 1O6A |
| HP0586 | DNA BINDING PROTEIN | CERTAIN | 9E-07 | 3K4X |
| HP0587 | PREDICTED AMINODEOXYCHORISMATE LYASE | CERTAIN | 6E-13 | 2R1F |
| HP0589 | OORA SUBUNIT OF THE 2-OXOGLUTARATE OXIDOREDUCTASE INVOLVED IN THE TCA CYCLE | CERTAIN | 2E-14 | 2C42 |
| HP0590 | ACETOLACTATE SYNTHASE, MITOCHONDRIAL. | CERTAIN | 6E-07 | 1T9B |
| HP0591 | OORC SUBUNIT OF THE 2-OXOGLUTARATE OXIDOREDUCTASE INVOLVED IN THE TCA CYCLE | CERTAIN | 1E-09 | 3G2E |
| HP0593 | ADENINE-SPECIFIC METHYLTRANSFERASE MBOIIA | CERTAIN | 1E-07 | 1G60 |
| HP0595 | PREDICTED DSBB-LIKE PROTEIN | CERTAIN | 8E-05 | 1L0Q |
| HP0596 | TNF-ALPHA INDUCER PROTEIN/IMMUNE SYSTEM | CERTAIN | 9E-12 | 2WCR |
| HP0598 | PREDICTED 8-AMINO-7-OXONONANOATE SYNTHASE | CERTAIN | 6E-13 | 3A2B |
| HP0599 | |  | METHYL-ACCEPTING CHEMOTAXIS PROTEIN | | --- | --- | | CERTAIN | 5E-08 | 3G67 |
| HP0600 | |  | MULTIDRUG RESISTANCE PROTEIN PGP-1 | | --- | --- | | CERTAIN | 2E-24 | 3B60 |
| HP0601 | FLAGELLIN A | CERTAIN | 9E-09 | 3K8W |
| HP0604 | PREDICTED UROPORPHYRINOGEN DECARBOXYLASE | CERTAIN | 1E-20 | 3CYV |
| HP0605 | |  | CATION EFFLUX SYSTEM PROTEIN CUSC | | --- | --- | | CERTAIN | 2E-12 | 3PIK |
| HP0606 | MULTIDRUG RESISTANCE PROTEIN | CERTAIN | 7E-07 | 4F4C |
| HP0621 | DNA MISMATCH REPAIR PROTEIN MUTS | CERTAIN | 8E-17 | 1EWQ |
| HP0629 | DNA BINDING PROTEIN/FANCONI ANEMIA GROUP I PROTEIN HOMOLOG | CERTAIN | 1E-05 | 3S51 |
| HP0631 | STRUCTURAL GENE FOR THE SMALL SUBUNIT OF HYDROGEN UPTAKE-LIKE HYDROGENASE | CERTAIN | 6E-17 | 3AYX |
| HP0633 | FORMATE DEHYDROGENASE, NITRATE-INDUCIBLE, MAJOR SUBUNIT | HIGH | 6E-04 | 1KQF |
| HP0634 | HYDROGENASE 2 MATURATION PROTEASE | CERTAIN | 7E-10 | 1CFZ |
| HP0645 | PROTEIN (SOLUBLE LYTIC TRANSGLYCOSYLASE SLT70) | CERTAIN | 4E-12 | 1QSA |
| HP0646 | PREDICTED UDP-GLUCOSE PYROPHOSPHORYLASE | CERTAIN | 1E-14 | 3JUK |
| HP0650 | URACIL-DNA GLYCOSYLASE | CERTAIN | 4E-08 | 1UI0 |
| HP0654 | BIOTIN SYNTHETASE/ADOMET BINDING PROTEIN | CERTAIN | 4E-11 | 3IIX |
| HP0656 | BIOTIN SYNTHASE/TRANSFERASE | CERTAIN | 4E-12 | 1R30 |
| HP0659 | POSSIBLE PERIPLASMIC PROTEIN/CHAPERONE | CERTAIN | 1E-10 | 3RGC |
| HP0660 | CHAPERONE | CERTAIN | 1E-05 | 2Y4T |
| HP0666 | UNCHARACTERIZED PROTEIN | CERTAIN | 6E-07 | 3KWL |
| HP0668 | MOTOR SUBUNIT OF TYPE I RESTRICTION-MODIFICATION COMPLEX | CERTAIN | 4E-08 | 2W00 |
| HP0669 | TYPE I RESTRICTION ENZYME STYSJI M PROTEIN | CERTAIN | 3E-09 | 2OKC |
| HP0673 | PROTEIN DISULFIDE-ISOMERASE/CHAPERONE | HIGH | 6E-04 | 3UEM |
| HP0675 | PREDICTED INTEGRASE/RECOMBINASE | CERTAIN | 1E-07 | 2A3V |
| HP0677 | MEMBRANE DOMAIN OF RESPIRATORY COMPLEX I | HIGH | 2E-04 | 3RKO |
| HP0680 | PREDICTED RIBONUCLEOSIDE-DIPHOSPHATE REDUCTASE 1 ALPHA SUBUNIT | CERTAIN | 7E-33 | 2XAP |
| HP0686 | |  | IRON(III) DICITRATE TRANSPORT PROTEIN FECA | | --- | --- | | CERTAIN | 6E-14 | 1KMO |
| HP0687 | |  | FERROUS IRON TRANSPORT PROTEIN B HOMOLOG | | --- | --- | | CERTAIN | 4E-09 | 2WJI |
| HP0688 | DNA POLYMERASE | HIGH | 2E-04 | 1QHT |
| HP0693 | |  | TRANSPORTER | | --- | --- | | CERTAIN | 6E-07 | 4F35 |
| HP0694 | OUTER MEMBRANE PROTEIN (LPXR) | CERTAIN | 2E-07 | 3FID |
| HP0695 | PANTHEONATE KINASE-LIKE PROTEIN | CERTAIN | 1E-04 | 3CET |
| HP0699 | SIGNALING PROTEIN/INTERFERON-INDUCED GUANYLATE-BINDING PROTEIN 1 | HIGH | 7E-04 | 1F5N |
| HP0700 | DIACYLGLYCEROL KINASE | CERTAIN | 3E-06 | 2KDC |
| HP0710 | SUBTILISIN-LIKE SERINE PROTEASE | HIGH | .0002 | 1R6V |
| HP0711 | 5'-DEOXYNUCLEOTIDASE YFBR/HYDROLASE | CERTAIN | 2E-07 | 2PAQ |
| HP0714 | PREDICTED ALTERNATIVE TRANSCRIPTION INITIATION FACTOR SIGMA-54 | CERTAIN | 2E-06 | 2K9M |
| HP0716 | ATPASE INVOLVED IN CELL WALL SYNTHESIS | CERTAIN | 4E-07 | 1HTW |
| HP0717 | LAMP LOADER GAMMA (GAMMA) COMPLEX OF E. COLI DNA POLYMERASE III. | CERTAIN | 9E-14 | 1JR3 |
| HP0718 | PEROXISOMAL ACYL-COA OXIDASE | HIGH | 7E-04 | 1W07 |
| HP0719 | DNA POLYMERASE III SUBUNIT GAMMA | CERTAIN | 9E-14 | 1JR3 |
| HP0724 | SODIUM-DEPENDENT DICARBOXYLATE TRANSPORTER. | CERTAIN | 9E-07 | 4F35 |
| HP0725 | DELTA-ENDOTOXIN | HIGH | 8E-04 | 2C9K |
| HP0726 | GALACTOCEREBROSIDASE | HIGH | 7E-04 | 3ZR5 |
| HP0728 | ISOLUECYL-TRNA LYSIDINE SYNTHETASE | CERTAIN | 2E-09 | 1WY5 |
| HP0731 | BACTERIAL DYNAMIN-LIKE PROTEIN | CERTAIN | 7E-11 | 2J69 |
| HP0733 | |  | BACTERIAL DYNAMIN-LIKE PROTEIN | | --- | --- | | CERTAIN | 2E-07 | 2J69 |
| HP0734 | OXYGEN-INDEPENDENT COPROPORPHYRINOGEN III OXIDASE | CERTAIN | 6E-13 | 2QGQ |
| HP0737 | PHOSPHATIDYLGLYCEROPHOSPHATASE (PGPASE), A PUTATIVE MEMBRANE-BOUND LIPID PHOSPHATASE, | HIGH | 3E-04 | 1Y9I |
| HP0740 | UDP-N-ACETYLMURAMOYLALANINE-D-GLUTAMYL-LYSINE-D-ALANYL-D-ALANINE LIGASE | CERTAIN | 7E-14 | 2AM1 |
| HP0743 | PREDICTED ROD SHAPE-DETERMINING PROTEIN | CERTAIN | 1E-06 | 1OTS |
| HP0745 | PREDICTED PSEUDOURIDINE SYNTHASE D INVOLVED IN 23S RRNA BASE MODIFICATIONS | CERTAIN | 5E-11 | 2IST |
| HP0746 | CELL ADHESION PROTEIN/FIBRONECTIN | CERTAIN | 5E-07 | 1FNF |
| HP0747 | PREDICTED S-ADENOSYLMETHIONINE-DEPENDENT METHYLTRANSFERASE | CERTAIN | 2E-09 | 1YZH |
| HP0750 | PUTATIVE PEPTIDASE M23 | CERTAIN | 1E-04 | 2HSI |
| HP0751 | PREDICTED POLAR FLAGELLIN | CERTAIN | 7E-05 | 2HC5 |
| HP0755 | MOLYBDOPTERIN BIOSYNTHESIS MOEB PROTEIN | CERTAIN | 6E-07 | 1JW9 |
| HP0758 | VESICULAR TRANSPORT FACTOR | CERTAIN | 8E-06 | 3GRL |
| HP0759 | MULTI ANTIMICROBIAL EXTRUSION PROTEIN (NA(+)/DRUG ANTIPORTER) MATE-LIKE MDR EFFLUX PUMP | CERTAIN | 1E-10 | 3MKT |
| HP0760 | PUTATIVE PHOSPHOHYDROLASE | CERTAIN | 9E-06 | 3M1T |
| HP0761 | 5-FORMYLTETRAHYDROFOLATE CYCLO-LIGASE FAMILY PROTEIN | CERTAIN | 2E-06 | 2 JCB |
| HP0762 | OXIDOREDUCTASE | HIGH | 7E-04 | 2B5X |
| HP0770 | |  | FLAGELLAR BIOSYNTHETIC PROTEIN | | --- | --- | | CERTAIN | 2E-07 | 3B0Z |
| HP0771 | CULLIN HOMOLOG 1/LIGASE | CERTAIN | 6E-05 | 1U6G |
| HP0772 | N-ACETYLMURAMOYL-L-ALANINE AMIDASE | CERTAIN | 3E-10 | 3NE8 |
| HP0773 | 2-NITROPROPANE DIOXYGENASE | CERTAIN | 4E-12 | 2GJL |
| HP0775 | PREDICTED BIFUNCTIONAL PPGPP-3'-PYROPHOSPHOHYDROLASE/PPGPP SYNTHETASE II | CERTAIN | 7E-16 | 1VJ7 |
| HP0781 | LEU/ILE/VAL-BINDING PROTEIN/TRANSPORT PROTEIN | CERTAIN | 2E-05 | 1Z15 |
| HP0782 | GLYCINE-GLUTAMATE DIPEPTIDE PORIN | CERTAIN | 9E-06 | 3SYB |
| HP0785 | OUTER-MEMBRANE LIPOPROTEIN CARRIER PROTEIN | CERTAIN | 2E-08 | 2W7Q |
| HP0788 | PREDICTED OUTER MEMBRANE PROTEIN HOFF | CERTAIN | 8E-05 | 3HRZ |
| HP0790 | TYPE I RESTRICTION-MODIFICATION ENZYME, S SUBUNIT | CERTAIN | 7E-13 | 1YF2 |
| HP0791 | CADMIUM-TRANSPORTING ATPASE/P-TYPE TRANSPORTING ATPASE INVOLVED IN TRANSITION METAL RESISTANCE | CERTAIN | 4E-26 | 3RFU |
| HP0792 | PREDICTED DNA TRANSFORMATION COMPETENCE PROTEIN PROBABLY INVOLVED IN RECOMBINATION | CERTAIN | 5E-07 | 1G8P |
| HP0795 | TRIGGER FACTOR | CERTAIN | 5E-20 | 1W26 |
| HP0797 | NEURAMINYLLACTOSE-BINDING HEMAGGLUTININ HOMOLOG | CERTAIN | 2E-10 | 3BGH |
| HP0807 | IRON(III) DICITRATE TRANSPORT PROTEIN FECA. | CERTAIN | 9E-13 | 1KMO |
| HP0812 | STRUCTURAL GENOMICS, UNKNOWN FUNCTION | CERTAIN | 1E-13 | 1ZKD |
| HP0813 | HYDROXYACYLGLUTATHIONE HYDROLASE | CERTAIN | 2E-07 | 2XF4 |
| HP0814 | MOLYBDOPTERIN BIOSYNTHESIS MOEB PROTEIN( PREDICTED ACTIVATOR OF MOAD/THIS SULFUR DONOR PROTEINS INVOLVED IN MOLYBDOPTERIN/THIAMINE BIOSYNTHETIC PATHWAYS) | CERTAIN | 3E-11 | 1JW9 |
| HP0816 | CHEMOTAXIS PROTEIN MOTB | CERTAIN | 3E-08 | 3CYP |
| HP0818 | GLYCINE BETAINE/CARNITINE/CHOLINE ABC TRANSPORTER CHAIN | CERTAIN | 7E-14 | 3O66 |
| HP0819 | UVRABC SYSTEM PROTEIN/ PREDICTED OSMOPROTECTION ABC TRANSPORTER/ATP-BINDING PROTEIN INVOLVED IN GLYCINE BETAINE/L-PROLINE TRANSPORT | CERTAIN | 3E-17 | 2D62 |
| HP0821 | UVRABC SYSTEM PROTEIN(PREDICTED EXCINUCLEASE ABC SUBUNIT C) | CERTAIN | 7E-11 | 2NRT |
| HP0823 | CRYSTAL STRUCTURE OF PROTEIN RPA0323 OF UNKNOWN FUNCTION | CERTAIN | 2E-05 | 3FOV |
| HP0828 | NADH-QUINONE OXIDOREDUCTASE SUBUNIT/ PREDICTED ATP SYNTHASE F0, SUBUNIT A | HIGH | 2E-04 | 3RKO |
| HP0829 | INOSINE MONOPHOSPHATE DEHYDROGENASE | CERTAIN | 1E-20 | 1ZFJ |
| HP0830 | GLUTAMYL-TRNA(GLN) AMIDOTRANSFERASE SUBUNIT. | CERTAIN | 8E-18 | 3IP4 |
| HP0831 | DEPHOSPHO-COA KINASE INVOLVED IN COENZYME A BIOSYNTHESIS | CERTAIN | 7E-11 | 1VHT |
| HP0834 | PROBABLE GTP-BINDING PROTEIN | CERTAIN | 1E-17 | 1MKY |
| HP0835 | HISTONE-LIKE DNA-BINDING PROTEIN HU PROTEIN(DNA BINDING ) | CERTAIN | 5E-06 | 1HUE |
| HP0839 | PROBABLE OUTER MEMBRANE PROTEIN | CERTAIN | 6E-10 | 3DWO |
| HP0843 | PROTEIN THIAMIN PHOSPHATE SYNTHASE | CERTAIN | 8E-12 | 2TPS |
| HP0845 | HYDROXYETHYLTHIAZOLE KINASE | CERTAIN | 3E-12 | 1ESJ |
| HP0846 | HYDROLASE/ PREDICTED TYPE I RESTRICTION ENZYME R PROTEIN | CERTAIN | 2E-31 | 2W00 |
| HP0848 | TYPE I RESTRICTION-MODIFICATION ENZYME(HYDROLASE REGULATOR) | CERTAIN | 3E-08 | 1YF2 |
| HP0851 | ALGINATE, A MAJOR COMPONENT OF THE CELL WALL MATRIX I | CERTAIN | .0001 | 3A0O |
| HP0855 | GTP-BINDING NUCLEAR PROTEIN(PROTEIN TRANSPORT)/ PREDICTED ALGINATE O-ACETYLATION PROTEIN | CERTAIN | 4E-07 | 3M1I |
| HP0858 | CARBOHYDRATE KINASE/ PREDICTED ADP-HEPTOSE SYNTHASE | CERTAIN | 6E-11 | 1RKD |
| HP0860 | D,D-HEPTOSE 1,7-BISPHOSPHATE PHOSPHATASE | CERTAIN | 4E-10 | 2GMW |
| HP0862 | TRANSCRIPTIONAL ACTIVATOR | CERTAIN | 6E-11 | 2NRH |
| HP0863 | LIPOPROTEIN(PROTEIN BINDING) | CERTAIN | 3E-06 | 3Q7O |
| HP0865 | DUTP PYROPHOSPHATASE/ PREDICTED DEOXYURIDINE 5'-TRIPHOSPHATE NUCLEOTIDOHYDROLASE | CERTAIN | 4E-09 | 3EHW |
| HP0867 | HYDROLASE/DNA/ PREDICTED LIPID A DISACCHARIDE SYNTHETASE | CERTAIN | 4E-07 | 1FOK |
| HP0869 | HYDROGENASE,UREASE NICKEL INCORPORATION PROTEIN | CERTAIN | 1E-08 | 2KDX |
| HP0872 | UNKNOWN FUNCTION/ PREDICTED ALKYLPHOSPHONATE UPTAKE PROTEIN | CERTAIN | 7E-09 | 2AKL |
| HP0874 | TRANSGLUTAMINASE-LIKE ENZYMES) | CERTAIN | 4E-05 | 3ISR |
| HP0883 | RUVA PROTEIN(HELICASE) | CERTAIN | 5E-11 | 1CUK |
| HP0885 | IMPORTIN BETA-1 SUBUNIT(NUCLEAR TRANSPORT)/ PREDICTED VIRULENCE FACTOR MVIN PROTEIN | CERTAIN | 8E-08 | 2BPT |
| HP0886 | HELICASE/ PREDICTED CYSTEINYL-TRNA SYNTHETASE | CERTAIN | 5E-18 | 1U0B |
| HP0888 | HYPOTHETICAL MALTOSE/MALTODEXTRIN TRANSPORT ATP-BINDING PROTEIN./ IRON(III) DICITRATE ABC TRANSPORTER, ATP-BINDING PROTEIN (FECE) | CERTAIN | 3E-14 | 2IT1 |
| HP0890 | 17 BETA-HYDROXYSTEROID DEHYDROGENASE | CERTAIN | 5E-11 | 1JTV |
| HP0891 | CYTOSOLIC ACYL COENZYME A THIOESTER HYDROLASE | CERTAIN | 4E-09 | 2QQ2 |
| HP0896 | GLYCOSYL HYDROLASE/ PREDICTED OUTER MEMBRANE PROTEIN HOMOLOGOUS TO AN ADHESIN BINDING FUCOSYLATED LEWIS B (LEB) HISTO-BLOOD GROUP ANTIGEN | HIGH | 2E-04 | 1TF4 |
| HP0899 | CHAPERONE, METAL BINDING PROTEIN | CERTAIN | 7E-06 | 2Z1C |
| HP0900 | HYDROLASE, METAL BINDING PROTEIN(HYPB PROTEIN INVOLVED IN THE MATURATION AND INCORPORATION OF NICKEL IONS IN THE HYDROGENASE AND UREASE CATALYTIC SITE) | CERTAIN | 1E-10 | 2HF9 |
| HP0902 | HYPOTHETICAL PROTEIN | CERTAIN | 6E-05 | 1YHF |
| HP0905 | DETHIOBIOTIN SYNTHASE/ PREDICTED PHOSPHOTRANSACETYLASE | HIGH | 2E-04 | 1BYI |
| HP0906 | PROTEIN TRANSPORT | CERTAIN | 3E-05 | 2RRL |
| HP0907 | |  | FLAGELLAR BASAL-BODY ROD MODIFICATION PROTEIN | | --- | --- | | CERTAIN | 8E-05 | 3OSV |
| HP0908 | MAJOR FRAGMENT OF THE HOOK PROTEIN | CERTAIN | 1E-07 | 1WLG |
| HP0910 | TRANSFERASE/DNA/ GTNNAC SITE-SPECIFIC TYPE II M6A METHYLASE | CERTAIN | 2E-09 | 2IH2 |
| HP0911 | DNA HELICASE | CERTAIN | 3E-21 | 3LFU |
| HP0912 | PESTICIDAL CRYSTAL PROTEIN(TOXIN)/ OUTER MEMBRANE PROTEIN HOPC/ALPA WITH PORIN AND ADHESIN PROPERTIES | HIGH | 5E-04 | 2C9K |
| HP0913 | PESTICIDAL CRYSTAL PROTEIN(TOXIN)/ OUTER MEMBRANE PROTEIN HOPB/ALPB WITH PORIN AND ADHESIN PROPERTIES | HIGH | 0.0003 | 2C9K |
| HP0914 | INTEGRIN ALPHA-V(PROTEIN BINDING )/ PREDICTED OUTER MEMBRANE PROTEIN HOPG | CERTAIN | 1E-05 | 3IJE |
| HP0915 | FERRIC ENTEROBACTIN RECEPTOR(MEMBRANE PROTEIN) | CERTAIN | 5E-09 | 1FEP |
| HP0916 | MEMBRANE PROTEIN,HEME BINDING PROTEIN | CERTAIN | 7E-06 | 3CSL |
| HP0918 | RNA BINDING PROTEIN | HIGH | 2E-04 | 3AJE |
| HP0920 | CELL ADHESION | HIGH | 4E-04 | 3L6X |
| HP0927 | HEAT SHOCK PROTEIN | CERTAIN | 5E-05 | 3CQB |
| HP0930 | HYDROLASE/ PREDICTED STATIONARY-PHASE SURVIVAL PROTEIN | CERTAIN | 4E-15 | 2WQK |
| HP0933 | LYASE | CERTAIN | 2E-06 | 2OBA |
| HP0934 | PYRUVATE FORMATE-LYASE 1-ACTIVATING ENZYME | CERTAIN | 9E-06 | 3C8F |
| HP0935 | PROBABLE ACETYLTRANSFERAS | CERTAIN | 7E-07 | 2GE3 |
| HP0936 | GLYCEROL-3-PHOSPHATE TRANSPORTE | CERTAIN | 7E-08 | 1PW4 |
| HP0939 | METHIONINE UPTAKE ATP BINDING CASSETTE (ABC) TRANSPORTER | CERTAIN | 1E-07 | 3TUI |
| HP0940 | ABC TRANSPORTER/ PREDICTED AMINO ACID ABC TRANSPORTER, PERIPLASMIC BINDING PROTEIN | CERTAIN | 1E-08 | 2YLN |
| HP0944 | PUTATIVE TRANSLATION INITIATION INHIBITOR(U)/ PREDICTED REGULATOR OF PURINE BIOSYNTHESIS | CERTAIN | 2E-09 | 1XRG |
| HP0949 | UNKNOWN FUNCTION/ PREDICTED SECRETED PROTEIN | CERTAIN | 4E-08 | 1VH0 |
| HP0950 | ACETYL-COENZYME A CARBOXYLASE CARBOXYL TRANSFERASE | CERTAIN | 6E-15 | 2F9I |
| HP0954 | OXYGEN-INSENSITIVE NADPH / NAD(P)H-DEPENDENT NITROREDUCTASE INVOLVED METRONIDAZOLE RESISTANCE  NITROREDUCTAS(OXIDOREDUCTASE) | CERTAIN | 4E-09 | 3QDL |
| HP0956 | RIBOSOMAL LARGE SUBUNIT PSEUDOURIDINE SYNTHASE(ISOMERASE) | CERTAIN | 6E-09 | 2IST |
| HP0957 | 3-DEOXY-D-MANNO-2-OCTULOSONIC ACID TRANSFERASE | CERTAIN | 2E-1 | 2XCI |
| HP0958 | GENE REGULATION, CHAPERONE | CERTAIN | 6E-14 | 3NA7 |
| HP0959 | UNKNOWN FUNCTION | CERTAIN | 3E-11 | 3LNL |
| HP0961 | OXIDOREDUCTASE/ PREDICTED GLYCEROL-3-PHOSPHATE DEHYDROGENASE INVOLVED IN MEMBRANE LIPIDS BIOSYNTHESIS | CERTAIN | 2E-15 | 3K96 |
| HP0963 | HYDROLASE | CERTAIN | 8E-10 | 2J69 |
| HP0964 | BACTERIAL DYNAMIN-LIKE PROTEIN (MECHANO-CHEMICAL GTPASES )/ | CERTAIN | 2E-15 | 2J69 |
| HP0965 | BACTERIAL DYNAMIN-LIKE PROTEIN (MECHANO-CHEMICAL GTPASES )/ | CERTAIN | 5E-09 | 2J69 |
| HP0966 | BACTERIAL DYNAMIN-LIKE PROTEIN | CERTAIN | 1E-13 | 2J69 |
| HP0970 | |  | MEMBRANE FUSION PROTEIN (MFP) HEAVY METAL CATION EFFLUX ZNEB (CZCB-LIKE) | | --- | --- | | CERTAIN | 3E-09 | 3LNN |
| HP0971 | OUTER MEMBRANE PROTEIN | CERTAIN | 2E-10 | 1EK9 |
| HP0972 | SCAFFOLD PROTEIN/ PREDICTED GLYCYL-TRNA SYNTHETASE, BETA SUBUNIT | HIGH | 0.0007 | 1B3U |
| HP0975 | GLUTAMYL-TRNA(GLN) AMIDOTRANSFERASE(LIGASE) | CERTAIN | 5E-05 | 3IP4 |
| HP0977 | SURVIVAL PROTEIN SURA (PEPTIDYL-PROLYL ISOMERASES) | CERTAIN | 4E-09 | 1M5Y |
| HP0982 | PROTEIN TRANSPORT | HIGH | 0.0003 | 2VGL |
| HP0983 | MEMBRANE PROTEIN | CERTAIN | 4E-12 | 2VV5 |
| HP0986 | STRUCTURAL GENOMICS, UNKNOWN FUNCTION | CERTAIN | 3E-07 | 1XMX |
| HP0996 | CONJUGATIVE MOBILIZATION PROTEIN | CERTAIN | 9E-05 | 2NS6 |
| HP0998 | HYPOTHETICAL TRANSPOSASE(GENE REGULATION) | CERTAIN | 3E-06 | 2EC2 |
| HP1000 | PARA FAMILY CHROMOSOME PARTITIONING PROTEIN(STRUCTURAL GENOMICS, UNKNOWN FUNCTION) | CERTAIN | 5E-07 | 3CWQ |
| HP1003 | CELL ADHESION | HIGH | 0.0005 | 1ST6 |
| HP1004 | MOBILIZATION PROTEIN | HIGH | 0.0003 | 2NS6 |
| HP1006 | BACTERIAL CONJUGATION PROTEIN TRWB RESEMBLES RING HELICASES AND F1-ATPASE | CERT | 5E-06 | 1E9R |
| HP1010 | POLYPHOSPHATE KINASE | CERTAIN | 2E-37 | 1XDP |
| HP1021 | DNA BINDING RESPONSE REGULATOR(DNA BINDING PROTEIN) | CERTAIN | 6E-09 | 1KGS |
| HP1022 | 5'-EXONUCLEASE(NUCLEASE) EXONUCLEASE | CERTAIN | 5E-06 | 1EXN |
| HP1023 | CELL ADHESION | CERTAIN | 9E-06 | 1DAB |
| HP1024 | HUMAN HSP40(MOLECULAR CHAPERONE) | CERTAIN | 4E-05 | 1HDJ |
| HP1025 | MERR-LIKE TRANSCRIPTIONAL REGULATOR/ TRANSCRIPTIONAL ACTIVATOR OF HEAT-SHOCK PROTEINS | CERTAIN | 7E-05 | 3QAO |
| HP1026 | REPLICATION-ASSOCIATED RECOMBINATION PROTEIN | CERTAIN | 5E-17 | 3PVS |
| HP1029 | STRUCTURAL GENOMICS, UNKNOWN FUNCTION | CERTAIN | 3E-07 | 1S4C |
| HP1030 | PUTATIVE FLAGELLAR MOTOR SWITCH PROTEIN | CERTAIN | 2E-06 | 1O6A |
| HP1031 | FLAGELLAR MOTOR SWITCH PROTEIN FLIM(SIGNALING PROTEIN) | CERTAIN | 4E-11 | 2HP7 |
| HP1032 | ALTERNATIVE TRANSCRIPTION INITIATION FACTOR SIGMA-28 | CERTAIN | 2E-08 | 1RP3 |
| HP1034 | CELL DIVISION INHIBITOR(CELL CYCLE, HYDROLASE)/ PROTEIN PREDICTED ATP-BINDING | CERTAIN | 2E-09 | 1G3Q |
| HP1035 | CELL DIVISION PROTEIN/ PREDICTED FLAGELLAR BIOSYNTHESIS PROTEIN WITH A GTP-BINDING DOMAIN | CERTAIN | 6E-14 | 2YHS |
| HP1036 | TRANSFERASE/ PREDICTED 7, 8-DIHYDRO-6-HYDROXYMETHYLPTERIN-PYROPHOSPHOKINASE | CERTAIN | 3E-08 | 3IP0 |
| HP1037 | AMINOPEPTIDASE | CERTAIN | 1E-12 | 2ZSG |
| HP1039 | NUCLEAR TRANSPORT | CERTAIN | 8E-08 | 2BPT |
| HP1040 | RIBOSOMAL PROTEIN S15 | CERTAIN | 1E-07 | 1A32 |
| HP1042 | EXOPOLYPHOSPHATASE-RELATED PROTEIN(STRUCTURAL GENOMICS, UNKNOWN FUNCTION) | CERTAIN | 8E-07 | 3DMA |
| HP1047 | RNA BINDING PROTEIN | CERTAIN | 5E-06 | 1JOS |
| HP1048 | TRANSLATION INITIATION FACTOR IF2/EIF5B | CERTAIN | 5E-15 | 1G7S |
| HP1050 | HOMOSERINE KINASE(TRANSFERASE) | CERTAIN | 1E-12 | 3HUL |
| HP1053 | SEPTUM SITE-DETERMINING PROTEIN MINC | CERTAIN | 3E-06 | 1HF2 |
| HP1054 | UNKNOWN FUNCTION | CERTAIN | 2E-06 | 2HSI |
| HP1062 | S-ADENOSYLMETHIONINE:TRNA RIBOSYLTRANSFERASE- ISOMERASE | CERTAIN | 7E-19 | 1YY3 |
| HP1063 | GLUCOSE-INHIBITED DIVISION PROTEIN(UNKNOWN FUNCTION) | CERTAIN | 3E-08 | 1JSX |
| HP1068 | RIBOSOMAL PROTEIN L11 METHYLTRANSFERASE | CERTAIN | 9E-11 | 2NXC |
| HP1071 | NADH-QUINONE OXIDOREDUCTASE SUBUNIT/ PREDICTED PHOSPHATIDYLSERINE SYNTHASE | CERTAIN | 4E-05 | 3RKO |
| HP1072 | HYDROLASE, MEMBRANE PROTEIN/ COPPER-TRANSPORTING ATPASE/P-TYPE TRANSPORTING ATPASE INVOLVED IN TRANSITION METAL RESISTANCE | CERTAIN | 8E-27 | 3RFU |
| HP1075 | SUCCINYLGLUTAMATE DESUCCINYLASE/ASPARTOACYLASE)/ PREDICTED SECRETED PROTEIN | CERTAIN | 5E-06 | 3NA6 |
| HP1077 | LIGASE/NUCLEAR PROTEIN/ HIGH-AFFINITY NICKEL TRANSPORTER REQUIRED FOR SCAVENGING OF NICKEL IONS UNDER NORMAL GASTRIC CONDITIONS | CERTAIN | 9E-05 | 2XWU |
| HP1078 | DE NOVO PROTEIN | HIGH | 0.0003 | 2LCI |
| HP1080 | STRUCTURAL GENOMICS, UNKNOWN FUNCTION/ PREDICTED INTEGRAL MEMBRANE PROTEIN | CERTAIN | 8E-05 | 1Y88 |
| HP1081 | RIBOSOME | CERTAIN | 2E-30 | 2Y14 |
| HP1082 | TRANSPORT PROTEIN/ PREDICTED LIPID A AND GLYCEROPHOSPHOLIPID TRANSPORTER INVOLVED IN THE BIOGENESIS OF LPS AND OF THE OUTER MEMBRANE | CERTAIN | 2E-05 | 3JTY |
| HP1084 | ASPARTATE TRANSCARBAMOYLASE | CERTAIN | 5E-17 |  |
| HP1086 | STRUCTURAL GENOMICS, UNKNOWN FUNCTION/ PORE-FORMING CYTOLYSIN | CERTAIN | 1E-10 | 3HP7 |
| HP1087 | RIBOFLAVIN BIOSYNTHESIS PROTEIN | CERTAIN | 3E-12 | 2X0K |
| HP1089 | RECOMBINATION | CERTAIN | 1E-07 | 1W36 |
| HP1091 | GLYCEROL-3-PHOSPHATE TRANSPORTER(MEMBRANE PROTEIN)/ PREDICTED ALPHA-KETOGLUTARATE PERMEASE | CERTAIN | 4E-10 | 1PW4 |
| HP1096 | 136AA LONG HYPOTHETICAL TRANSPOSASE (GENE REGULATION) | CERTAIN | 3E-06 | 2EC2 |
| HP1100 | 6-PHOSPHOGLUCONATE DEHYDRATASE | CERTAIN | 4E-26 | 2GP4 |
| HP1101 | GLUCOSE-6-PHOSPHATE 1-DEHYDROGENASE(OXIDOREDUCTASE) | CERTAIN | 1E-22 | 2BH9 |
| HP1105 | GALACTOSYL TRANSFERASE/ PREDICTED LPS BIOSYNTHESIS PROTEIN | CERTAIN | 2E-09 | 1GA8 |
| HP1110 | PYRUVATE-FERREDOXIN OXIDOREDUCTASE | CERTAIN | 1E-18 | 2C42 |
| HP1111 | ACETOLACTATE SYNTHASE(TRANSFERASE)/ PYRUVATE FERREDOXIN OXIDOREDUCTASE, BETA SUBUNIT | CERTAIN | 5E-05 | 1YBH |
| HP1112 | ADENYLOSUCCINATE LYASE | CERTAIN | 7E-21 | 2PFM |
| HP1114 | DNA EXCISION REPAIR | CERTAIN | 1E-31 | 1T5L |
| HP1116 | PROBABLE ATP-DEPENDENT RNA HELICASE | HIGH | 0.0008 | 2YKG |
| HP1119 | FLAGELLAR HOOK-ASSOCIATED PROTEIN(STRUCTURAL PROTEIN) | CERTAIN | 5E-14 | 2D4Y |
| HP1121 | TRANSFERASE(DNA) | CERTAIN | 1E-10 | 1DCT |
| HP1124 | METAL BINDING | CERTAIN | 2E-06 | 2XEV |
| HP1125 | OUTER MEMBRANE PROTEIN/ PREDICTED PEPTIDOGLYCAN-ASSOCIATED LIPOPROTEIN PRECURSOR | CERTAIN | 9E-06 | 2AIZ |
| HP1126 | TRANSPORT PROTEIN(LIPOPROTEIN) | CERTAIN | 9E-15 | 2HQS |
| HP1129 | BIOPOLYMER TRANSPORT EXBD PROTEIN | HIGH | 0.0001 | 2PFU |
| HP1135 | PREDICTED ATP SYNTHASE F1 DELTA CHAIN | HIGH | 0.0009 | 2WSS |
| HP1138 | PREDICTED PLASMID REPLICATION-PARTITION RELATED PROTEIN | CERTAIN | 1E-11 | 1V20 |
| HP1142 | ALPHA-ACTININ(TRIPLE-HELIX COILED COIL) | CERTAIN | 8E-06 | 1HCI |
| HP1143 | PHOSPHOLIPASE | CERTAIN | 3E-05 | 1JAD |
| HP1147 | 23S RRNA( PREDICTED RIBOSOMAL PROTEIN L19) | CERTAIN | 4E-09 | 3R8S |
| HP1148 | TRANSFERASE, RNA BINDING PROTEIN | CERTAIN | 2E-14 | 3IEF |
| HP1149 | RIBOSOME MATURATION FACTOR(PREDICTED 16S RRNA PROCESSING PROTEIN) | CERTAIN | 5E-08 | 3H9N |
| HP1152 | PREDICTED SIGNAL RECOGNITION PARTICLE PROTEIN | CERTAIN | 5E-24 | 2IY3 |
| HP1154 | CONTRACTILE PROTEIN | CERTAIN | 5E-07 | 2AJ7 |
| HP1155 | UDP-N-ACETYLGLUCOSAMINE-N-ACETYLMURAMYL- (PENTAPEPTIDE) PYROPHOSPHORYL-UNDECAPRENOL N- ACETYLGLUCOSAMINE TRANSFERASE | CERTAIN | 9E-16 | 1F0K |
| HP1156 | BOTULINUM NEUROTOXIN(HYDROLASE)/ PREDICTED OUTER MEMBRANE PROTEIN HOPI | HIGH | 0.0004 | 3FFZ |
| HP1158 | PUTATIVE PYRROLINE CARBOXYLATE REDUCTASE(OXIDOREDUCTASE) | CERTAIN | 2E-10 | 2AHR |
| HP1165 | GLYCEROL-3-PHOSPHATE TRANSPORTER(MEMBRANE PROTEIN) | CERTAIN | 1E-08 | 1PW4 |
| HP1167 | INTEGRIN ALPHA CHAIN, | CERTAIN | 6E-05 | 3IJE |
| HP1168 | CULLIN HOMOLOG(LIGASE)/ PREDICTED CARBON STARVATION PROTEIN | CERTAIN | 4E-07 | 1U6G |
| HP1169 | D-METHIONINE TRANSPORT SYSTEM PERMEASE PROTEIN METI | CERTAIN | 1E-06 | 3TUI |
| HP1170 | HYDROLASE/TRANSPORT PROTEIN(PREDICTED GLUTAMINE ABC TRANSPORTER, PERMEASE PROTEIN) | CERTAIN | 9E-08 | 3TUI |
| HP1174 | L-FUCOSE-PROTON SYMPORTER(TRANSPORT PROTEIN)/ PREDICTED GLUCOSE/GALACTOSE TRANSPORTER | CERTAIN | 4E-23 | 3O7Q |
| HP1175 | NADH-QUINONE OXIDOREDUCTASE | CERTAIN | 2E-05 | 3RKO |
| HP1177 | HYDROLASE/ PREDICTED OUTER MEMBRANE PROTEIN HOPQ | HIGH | 0.0004 | 1G87 |
| HP1180 | CONCENTRATIVE NUCLEOSIDE TRANSPORTER | CERTAIN | 2E-08 | 3M1I |
| HP1182 | TRANSLATION | CERTAIN | 5E-08 | 1WY5 |
| HP1183 | NADH-QUINONE OXIDOREDUCTASE/ PREDICTED NA+/H+ ANTIPORTER | CERTAIN | 1E-06 | 3RKO |
| HP1184 | MULTI ANTIMICROBIAL EXTRUSION PROTEIN(TRANSPORT PROTEIN) | CERTAIN | 5E-11 | 3MKT |
| HP1189 | OXIDOREDUCTASE/OXIDOREDUCTASE INHIBITOR/ PREDICTED ASPARTATE-SEMIALDEHYDE DEHYDROGENASE | CERTAIN | 9E-16 | 3PWK |
| HP1190 | HISTIDYL-TRNA SYNTHETASE | CERTAIN | 8E-17 | 1WU7 |
| HP1191 | GLYCOSYL TRANSFERASE FAMILY | CERTAIN | 4E-14 | 3TOV |
| HP1195 | ELONGATION FACTOR(TRANSLATION) | CERTAIN | 2E-35 | 2XEX |
| HP1196 | 16S RRNA/ PREDICTED RIBOSOMAL PROTEIN S7 | CERTAIN | 7E-12 | 2VQE |
| HP1197 | 16S RRNA/ PREDICTED RIBOSOMAL PROTEIN S12 | CERTAIN | 1E-10 | 3R8N |
| HP1199 | 50S RIBOSOMAL PROTEIN(L7/L12) | CERTAIN | 2E-08 | 1RQU |
| HP1200 | 50S RIBOSOMAL PROTEIN(L10) | CERTAIN | 1E-08 | 1ZAV |
| HP1201 | 50S RIBOSOMAL PROTEIN(L1) | CERTAIN | 2E-16 | 3QOY |
| HP1203 | TRANSCRIPTION ANTITERMINATION PROTEIN | CERTAIN | 7E-10 | 1NPP |
| HP1204 | 23S RRNA.(L33) | HIGH | 0.0002 | 3R8S |
| HP1206 | 23S RRNA(RIBOSOMES)/ PREDICTED ABC-TRANSPORTER, ATP-BINDING DOMAIN | CERTAIN | 4E-26 | 2Y14 |
| HP1208 | (ADENINE-SPECIFIC METHYLTRANSFERASE/ UBIQUITOUS CATG SITE-SPECIFIC TYPE II M6A METHYLASE | CERTAIN | 5E-05 | 2DPM |
| HP1210 | SERINE ACETYLTRANSFERASE | CERTAIN | 1E-08 | 1SSQ |
| HP1212 | ATP SYNTHASE SUBUNIT C | CERTAIN | 8E-05 | 2WGM |
| HP1213 | POLYRIBONUCLEOTIDE NUCLEOTIDYLTRANSFERASE | CERTAIN | 5E-28 | 3U1K |
| HP1216 | INTEGRIN(PROTEIN BINDING)/ PREDICTED ORGANIC SOLVENT TOLERANCE PROTEIN | CERTAIN | 1E-07 | 3IJE |
| HP1227 | ELECTRON TRANSPORT/ PREDICTED CYTOCHROME C553 | HIGH | 0.0002 | 1CNO |
| HP1244 | 16S RIBOSOMAL RNA( S18) | CERTAIN | 3E-05 | 3IZV |
| HP1246 | 30S RIBOSOMAL PROTEIN( S6) | CERTAIN | 3E-07 | 2J5A |
| HP1250 | PROBABLE ENTEROTOXIN(STRUCTURAL GENOMICS, UNKNOWN FUNCTION) | HIGH | 0.0007 | 2KRS |
| HP1259 | GENE REGULATION | CERTAIN | 4E-09 | 1M2K |
| HP1260 | NADH-QUINONE OXIDOREDUCTASE(CHAIN A) | HIGH | 0.0006 | 3RKO |
| HP1261 | NADH-QUINONE OXIDOREDUCTASE(CHAIN B) | CERTAIN | 4E-09 | 3IAM |
| HP1262 | NADH-QUINONE OXIDOREDUCTASE(CHAIN C) | CERTAIN | 1E-07 | 3IAM |
| HP1266 | NADH-QUINONE OXIDOREDUCTASE(CHAIN G) | CERTAIN | 1E-13 | 3IAM |
| HP1267 | NADH-QUINONE OXIDOREDUCTASE(CHAIN H) | HIGH | 0.0003 | 3RKO |
| HP1269 | NADH-QUINONE OXIDOREDUCTASE(CHAIN J) | CERTAIN | 4E-05 | 3RKO |
| HP1270 | NADH-QUINONE OXIDOREDUCTASE(CHAIN K) | CERTAIN | 5E-08 | 3RKO |
| HP1271 | NADH-QUINONE OXIDOREDUCTASE(CHAIN L) | CERTAIN | 1E-26 | 3RKO |
| HP1272 | NADH-QUINONE OXIDOREDUCTASE(CHAIN M) | CERTAIN | 2E-24 | 3RKO |
| HP1274 | ANAPHASE-PROMOTING COMPLEX SUBUNIT/ PREDICTED PARALYSED FLAGELLA PROTEIN | CERTAIN | 4E-11 | 2XPI |
| HP1275 | PHOSPHOMANNOMUTASE(ISOMERASE) | CERTAIN | 1E-19 | 1P5D |
| HP1285 | LIPOPROTEIN | CERTAIN | 5E-11 | 3OCU |
| HP1290 | IMPORTIN(NUCLEAR TRANSPORT) | HIGH | 0.0007 | 2BPT |
| HP1291 | HYPOTHETICAL PROTEIN(TRANSFERASE) | CERTAIN | 2E-10 | 2OMK |
| HP1293 | DNA-DIRECTED RNA POLYMERASE(TRANSFERASE) | CERTAIN | 3E-12 | 2A6H |
| HP1295 | 16S RRNA PROTEIN S11 (RPS11) | CERTAIN | 8E-10 | 3R8N |
| HP1296 | 16S RRNA PROTEIN S13 (RPS13) | CERTAIN | 8E-09 | 2VQE |
| HP1297 | RIBOSOMAL 23S RNA L36 (RPL36) | HIGH | 0.0002 | 2ZJR |
| HP1298 | NITIATION FACTOR(RIBOSOME BINDING) | CERTAIN | 1E-06 | 1AH9 |
| HP1300 | PROTEIN TRANSPORT/IMMUNE SYSTEM/ PREDICTED PREPROTEIN TRANSLOCASE SUBUNIT | CERTAIN | 1E-23 | 2ZJS |
| HP1301 | 23S RRNA PROTEIN L15. | CERTAIN | 3E-06 | 3R8S |
| HP1302 | RIBOSOMAL PROTEIN S5 | CERTAIN | 1E-09 | 1PKP |
| HP1303 | 50S RIBOSOMAL PROTEIN L18 | CERTAIN | 2E-07 | 1OVY |
| HP1306 | 16S RRNA PROTEIN S14 | CERTAIN | 3E-05 | 2VQE |
| HP1308 | 23S RRNA PROTEIN L24 | CERTAIN | 2E-05 | 3R8S |
| HP1310 | 16S RRNA PROTEIN S17 | CERTAIN | 3E-06 | 3R8N |
| HP1311 | 50S RIBOSOMAL PROTEIN L29 | HIGH | 0.0002 | 1R73 |
| HP1315 | 16S RIBOSOMAL RNA PROTEIN S19 | CERTAIN | 4E-07 | 3IZV |
| HP1316 | 23S RRNA PROTEIN L2/PEPTIDYL-TRANSFERASE | CERTAIN | 5E-17 | 3R8S |
| HP1317 | 23S RRNA PROTEIN L23 | CERTAIN | 5E-17 | 3R8S |
| HP1318 | 23S RRNA PROTEIN L4 | CERTAIN | 4E-10 | 3R8S |
| HP1319 | 23S RRNA PROTEIN L3 | CERTAIN | 4E-10 | 3R8S |
| HP1320 | 16S RRNA PROTEIN S10 | CERTAIN | 2E-06 | 3R8N |
| HP1323 | HYDROLASE/DNA-RNA HYBRID/ PREDICTED RIBONUCLEASE HII | CERTAIN | 2E-09 | 3O3F |
| HP1327 | TRANSPORT PROTEIN | CERTAIN | 3E-09 | 3PIK |
| HP1328 | METAL TRANSPORT | CERTAIN | 2E-11 | 3NE5 |
| HP1331 | NUCLEAR TRANSPORT/ PREDICTED CO-CHAPERONE PROTEIN | HIGH | 0.0002 | 2BPT |
| HP1332 | ACONITASE ,LYASE(CARBON-OXYGEN)/ PREDICTED CO-CHAPERONE PROTEIN | CERTAIN | 6E-07 | 1NIT |
| HP1333 | SPECTRIN ALPHA CHAIN | CERTAIN | 8E-05 | 1U4Q |
| HP1334 | FORMYLTETRAHYDROFOLATE DEFORMYLASE.( HYDROLASE) | CERTAIN | 3E-14 | 3O1L |
| HP1337 | NAMN ADENYLYLTRANSFERASE/ PREDICTED NICOTINATE-NUCLEOTIDE ADENYLTRANSFERASE INVOLVED IN NAD BIOSYNTHESIS | CERTAIN | 2E-08 | 1K4M |
| HP1341 | PEPTIDYL-PROLYL CIS-TRANS ISOMERASE/ PREDICTED SIDEROPHORE-MEDIATED IRON TRANSPORT PROTEIN | CERTAIN | 9E-08 | 1W74 |
| HP1342 | ENDOCYTOSIS,EXOCYTOSIS. | HIGH | 0.0009 | 2B1E |
| HP1343 | DNA BINDING PROTEIN | CERTAIN | 9E-05 | 3S4W |
| HP1344 | DIVALENT CATION TRANSPORT-RELATED PROTEIN(MEMBRANE PROTEIN) | CERTAIN | 1E-11 | 2IUB |
| HP1346 | GLYCERALDEHYDE-3-PHOSPHATE DEHYDROGENASE | CERTAIN | 4E-15 | 3K2B |
| HP1348 | GLYCEROL-3-PHOSPHATE ACYLTRANSFERASE | CERTAIN | 2E-06 | 1IUQ |
| HP1350 | PHOTOSYSTEM II D1 PROTEASE(HYDROLASES)/ PREDICTED CARBOXYL-TERMINAL PROTEASE | CERTAIN | 7E-15 | 1FC6 |
| HP1352 | RESTRICTION ENDONUCLEASE(HYDROLASE, TRANSFERASE)/ PREDICTED ADENINE SPECIFIC DNA METHYLTRANSFERASE | CERTAIN | 8E-09 | 3S1S |
| HP1354 | ADENINE-SPECIFIC METHYLTRANSFERASE | CERTAIN | 1E-08 | 1G60 |
| HP1360 | DEOXYRIBOSE-PHOSPHATE ALDOLASE(LYASES)/ PREDICTED 4-HYDROXYBENZOATE OCTAPRENYLTRANSFERASE | HIGH | 0.0001 | 3NDO |
| HP1361 | CHLORINE TRANSPORT PROTEIN(MEMBRANE PROTEIN)/ PREDICTED DNA COMPETENCE PROTEIN | CERTAIN | 2E-05 | 1KPL |
| HP1364 | SENSOR HISTIDINE KINASE(TRANSFERASE) | CERTAIN | 2E-09 | 2C2A |
| HP1368 | PUTATIVE MODIFICATION METHYLASE(TRANSFERASE) | CERTAIN | 2E-08 | 2ZIG |
| HP1371 | HYDROLASE | CERTAIN | 5E-06 | 2W00 |
| HP1372 | CELL SHAPE REGULATION/ PREDICTED ROD SHAPE-DETERMINING PROTEIN | CERTAIN | 4E-08 | 2J5U |
| HP1375 | |  | ACYL-[ACYL-CARRIER-PROTEIN]--UDP-N-ACETYLGLUCOSAMINE O-ACYLTRANSFERASE | | --- | --- | | CERTAIN | 1E-13 | 1J2Z |
| HP1376 | (3R)-HYDROXYMYRISTOYL-ACYL CARRIER PROTEIN DEHYDRATASE | CERTAIN | 2E-11 | 2GLL |
| HP1377 | HYDROLASE ACTIVATOR,PROTEIN BINDING | CERTAIN | 1E-07 | 2AIJ |
| HP1378 | MEMBRANE PROTEIN/ PREDICTED COMPETENCE LIPOPROTEIN | CERTAIN | 2E-08 | 2YHC |
| HP1379 | ATP-DEPENDENT PROTEASE | CERTAIN | 2E-21 | 3M6A |
| HP1380 | PREPHENATE DEHYDROGENASE | CERTAIN | 1E-13 | 3GGG |
| HP1383 | TYPE I RESTRICTION-MODIFICATION ENZYME, S SUBUNIT | CERTAIN | 2E-12 | 3TOV |
| HP1385 | FRUCTOSE-1,6-BISPHOSPHATASE | CERTAIN | 8E-13 | 1NUY |
| HP1387 | DNA POLYMERASE(TRANSFERASE) | CERTAIN | 1E-07 | 2P1J |
| HP1392 | FIBRINOGEN BINDING PROTEIN | HIGH | 0.0001 | 3DOA |
| HP1393 | DNA DOUBLE-STRAND BREAK REPAIR RAD50 ATPASE | CERTAIN | 4E-05 | 3QKT |
| HP1399 | HYDROLASE/ ARGINASE INVOLVED IN NITROGEN METABOLISM | CERTAIN | 4E-13 | 1D3V |
| HP1400 | IRON(III) DICITRATE TRANSPORT PROTEIN(MEMBRANE PROTEIN) | CERTAIN | 1E-12 | 1KMO |
| HP1402 | MOTOR SUBUNIT OF TYPE I RESTRICTION-MODIFICATION COMPLEX | CERTAIN | 2E-27 | 2W00 |
| HP1403 | TYPE I RESTRICTION-MODIFICATION SYSTEM METHYLTRANSFERASE | CERTAIN | 4E-17 | 3LKD |
| HP1409 | DNA BINDING PROTEIN | CERTAIN | 9E-05 | 3S4W |
| HP1410 | EXONUCLEASE RECJ.(HYDROLASE) | CERTAIN | 2E-11 | 1IR6 |
| HP1411 | CHAPERONE | HIGH | 0.0004 | 1QVR |
| HP1413 | NADPH-DEPENDENT 7-CYANO-7-DEAZAGUANINE REDUCTASE(OXIDOREDUCTASE/OXIDOREDUCTASE SUBSTRATE) | CERTAIN | 6E-07 | 3UXJ |
| HP1414 | STRUCTURAL GENOMICS, UNKNOWN FUNCTION | CERTAIN | 9E-07 | 2ID1 |
| HP1415 | TRNA DELTA(2)-ISOPENTENYLPYROPHOSPHATE TRANSFERAS | CERTAIN | 2E-12 | 3FOZ |
| HP1416 | GALACTOSYL TRANSFERASE | CERTAIN | 1E-10 | 1GA8 |
| HP1418 | UDP-N-ACETYLENOLPYRUVOYLGLUCOSAMINE REDUCTASE | CERTAIN | 7E-13 | 1HSK |
| HP1421 | TYPE IV SECRETION SYSTEM PROTEIN | CERTAIN | 1E-11 | 2GZA |
| HP1428 | RIBOSOMAL RNA LARGE SUBUNIT METHYLTRANSFERASE(OXIDOREDUCTASE) | CERTAIN | 9E-17 | 3RFA |
| HP1429 | PUTATIVE PHOSPHOSUGAR ISOMERASE INVOLVED IN CAPSULE FORMATION | CERTAIN | 2E-08 | 3ETN |
| HP1431 | PROBABLE DIMETHYLADENOSINE TRANSFERASE(TRANSFERASE) | CERTAIN | 4E-10 | 1ZQ9 |
| HP1433 | RESTRICTION ENDONUCLEASE (HYDROLASE, TRANSFERASE) | CERTAIN | 3E-05 | 3S1S |
| HP1434 | FORMYLTETRAHYDROFOLATE DEFORMYLASE | CERTAIN | 9E-15 | 3O1L |
| HP1435 | PROTEASE(HYDROLASE)/ PREDICTED SIGNAL PEPTIDE PROTEASE IV | CERTAIN | 3E-08 | 3BF0 |
| HP1442 | CARBON STORAGE REGULATOR HOMOLOG | CERTAIN | .0001 | 2BTI |
| HP1443 | 4-DIPHOSPHOCYTIDYL-2C-METHYL-D-ERYTHRITOL KINASE(TRANSFERASE) | CERTAIN | 1E-09 | 2WW4 |
| HP1447 | 23S RRNA PROTEIN L34 | CERTAIN | 0.0001 | 3R8S |
| HP1450 | CHAPERONE,PROTEIN TRANSPORT/ PREDICTED INNER MEMBRANE PROTEIN | CERTAIN | 9E-06 | 3BLC |
| HP1452 | TRNA MODIFICATION GTPASE(HYDROLASE)/ PREDICTED THIOPHENE/FURAN OXIDATION PROTEIN | CERTAIN | 3E-20 | 1XZP |
| HP1453 | TRIPEPTIDYL-PEPTIDASE(HYDROLASE)/ PREDICTED OUTER MEMBRANE PROTEIN HOMD | HIGH | 0.0003 | 3LXU |
| HP1459 | RIBOSOMAL SMALL SUBUNIT PSEUDOURIDINE SYNTHASE(LYASE) | CERTAIN | 7E-07 | 1KSK |
| HP1465 | D-METHIONINE TRANSPORT SYSTEM PERMEASE PROTEIN(HYDROLASE/TRANSPORT PROTEIN)/ PREDICTED ABC TRANSPORTER ATP-BINDING PROTEIN | CERTAIN | 9E-13 | 3TUI |
| HP1466 | DNA BINDING PROTEIN/ PREDICTED ABC TRANSPORT SYSTEM PERMEASE | HIGH | 0.0008 | 3S51 |
| HP1470 | TAQ DNA POLYMERASE(POLYMERASE/INHIBITOR) | CERTAIN | 2E-23 | 1BGX |
| HP1471 | DNA BINDING PROTEIN/ NON-FUNCTIONAL TYPE IIS SEQUENCE SPECIFIC BCGI-LIKE S-SUBUNIT IN A SILENT STATE | CERTAIN | 6E-06 | 1YDX |
| HP1472 | TYPE I RESTRICTION ENZYME STYSJI M PROTEIN | CERTAIN | 1E-10 | 2OKC |
| HP1474 | THYMIDYLATE KINASE TRANSFERASE | CERTAIN | 2E-09 | 3LV8 |
| HP1476 | PROBABLE AROMATIC ACID DECARBOXYLASE | CERTAIN | 3E-08 | 1SBZ |
| HP1477 | STRUCTURAL GENOMICS, UNKNOWN FUNCTION | HIGH | 0.0006 | 3FRN |
| HP1478 | DNA HELICASE | CERTAIN | 7E-24 | 1PJR |
| HP1479 | UDP-N-ACETYLGLUCOSAMINE--PEPTIDE N-ACETYLGLUCOSAMINYLTRANSFERASE | CERTAIN | 3E-07 | 1W3B |
| HP1483 | METHYLTRANSFERASE | CERTAIN | 4E-07 | 1IM8 |
| HP1484 | TRANSFERASE/ PREDICTED INTEGRAL MEMBRANE PROTEIN | CERTAIN | 4E-07 | 1IM8 |
| HP1486 | PUTATIVE ABC TYPE-2 TRANSPORTER | CERTAIN | 5E-05 | 3CNI |
| HP1487 | PUTATIVE ABC TYPE-2 TRANSPORTER | HIGH | 0.0002 | 3CNI |
| HP1488 | MEMBRANE PROTEIN/ PREDICTED SECRETED PROTEIN | CERTAIN | 7E-07 | 3FPP |
| HP1489 | OUTER MEMBRANE PROTEIN | CERTAIN | 2E-12 | 1EK9 |
| HP1490 | STRUCTURAL GENOMICS, UNKNOWN FUNCTION/ PREDICTED INTEGRAL MEMBRANE PROTEIN WITH A TLYC-LIKE HEMOLYSIN DOMAIN | CERTAIN | 4E-07 | 3OI8 |
| HP1491 | GTP-BINDING NUCLEAR PROTEIN/ PREDICTED PHOSPHATEPERMEASE | CERTAIN | 3E-08 | 3M1I |
| HP1492 | METAL TRANSPORT/ PREDICTED NIFU-LIKE PROTEIN | HIGH | 0.0005 | 2Z51 |
| HP1494 | UDP-N-ACETYLMURAMOYLALANYL-D-GLUTAMATE--2,6- DIAMINOPIMELATE LIGASE | CERTAIN | 4E-12 | 1E8C |
| HP1505 | DIAMINOHYDROXYPHOSPHORIBOSYLAMINOPYRIMIDINE DEAMI AMINO-6-(5-PHOSPHORIBOSYLAMINO)URACIL REDUCTASE(BIOSYNTHETIC PROTEIN) | CERTAIN | 2E-05 | 2HXV |
| HP1507 | OXIDOREDUCTASE | CERTAIN | 4E-11 | 1FF9 |
| HP1508 | STRUCTURAL GENOMICS, UNKNOWN FUNCTION/ PREDICTED FERREDOXIN-LIKE PROTEIN | HIGH | 0.0002 | 2R39 |
| HP1510 | |  | DIHYDRONEOPTERIN ALDOLASE | | --- | --- | | CERTAIN | 4E-06 | 2O90 |
| HP1513 | UNKNOWN FUNCTION/ PREDICTED SELENOCYSTEINE SYNTHASE | CERTAIN | 2E-05 | 2AEU |
| HP1514 | TRANSCRIPTION ELONGATION/ANTI-TERMINATION FACTOR NUSA | CERTAIN | 1E-10 | 1K0R |
| HP1520 | DISHEVELED-ASSOCIATED ACTIVATOR OF MORPHOGENESIS 1 | CERTAIN | .0005 | 2J1D |
| HP1521 | MOTOR SUBUNIT OF TYPE I RESTRICTION-MODIFICATION COMPLEX/PREDICTED TYPE III R-M SYSTEM RESTRICTION ENZYME | CERTAIN | 3E-06 | 2W00 |
| HP1523 | HELICASE | CERTAIN | 2E-09 | 3GM5 |
| HP1530 | S-ADENOSYLHOMOCYSTEINE NUCLEOSIDASE | HIGH | 0.0003 | 3O4V |
| HP1535 | UNKNOWN FUNCTION/ PREDICTED IS605 TRANSPOSASE A | CERTAIN | 1E-05 | 2FYX |
| HP1538 | CYTOCHROME B (OXIDOREDUCTASE/METAL TRANSPORT)/ PREDICTED UBIQUINOL CYTOCHROME C OXIDOREDUCTASE, CYTOCHROME C SUBUNIT | CERTAIN | 4E-05 | 1ZRT |
| HP1539 | CYTOCHROME B(OXIDOREDUCTASE) /PREDICTED UBIQUINOL CYTOCHROME C OXIDOREDUCTASE, CYTOCHROME B SUBUNIT | CERTAIN | 1E-12 | 2QJY |
| HP1540 | CYTOCHROME B(OXIDOREDUCTASE)/ PREDICTED UBIQUINOL CYTOCHROME C OXIDOREDUCTASE, 2FE-2S SUBUNIT | CERTAIN | 6E-05 | 2YIU |
| HP1543 | ZINC PEPTIDASE | CERTAIN | 1E-05 | 3SLU |
| HP1544 | ZINC PEPTIDASE PREDICTED TOXR-ACTIVATED GENE | HIGH | 0.0002 | 3GU1 |
| HP1547 | AMINOACYL-TRNA SYNTHETASE | CERTAIN | 7E-27 | 2V0C |
| HP1549 | PROBABLE SECDF PROTEIN-EXPORT MEMBRANE PROTEIN. | CERTAIN | 1E-12 | 3AQP |
| HP1550 | PROBABLE SECDF PROTEIN-EXPORT MEMBRANE PROTEIN. | CERTAIN | 5E-15 | 3AQP |
| HP1552 | NA(+)/H(+) ANTIPORTER(MEMBRANE PROTEIN) | CERTAIN | 6E-23 | 1ZCD |
| HP1553 | EXODEOXYRIBONUCLEASE(RECOMBIONATION)/ PREDICTED ATP-DEPENDENT NUCLEASE | CERTAIN | 3E-12 | 1W36 |
| HP1555 | TRANSLATION, TRANSFERASE/RNA | CERTAIN | 1E-18 | 3AVX |
| HP1556 | PREDICTED PENICILLIN-BINDING PROTEIN 3 OR FTSI INVOLVED IN PEPTIDOGLYCAN SYNTHESIS AND CELL DIVISION | CERTAIN | 1E-18 | 3PBT |
| HP1560 | ENVELOPE GLYCOPROTEIN(VIRAL PPROTEIN)/ PREDICTED CELL DIVISION PROTEIN | HIGH | 0.0006 | 3M1C |
| HP1561 | IRON(III) ABC TRANSPORTER, PERIPLASMIC IRON-BINDING PROTEIN | CERTAIN | 1E-07 | 3PSH |
| HP1568 | TRANSPORT PROTEIN | HIGH | 0.0003 | 2R19 |
| HP1570 | 3-DEOXY-D-MANNO-OCTULOSONATE 8-PHOSPHATE PHOSPHATASE.(HYDROLASE)/ PREDICTED ABC TRANSPORTER SYSTEM INNER MEMBRANE PROTEIN | CERTAIN | 4E-07 | 2R8E |
| HP1572 | ENDO-TYPE MEMBRANE-BOUND LYTIC MUREIN TRANSGLYCOS(LYASE)/ PREDICTED REGULATORY PROTEIN | CERTAIN | 5E-05 | 2Y8P |
| HP1575 | |  | FLAGELLAR BIOSYNTHETIC PROTEIN | | --- | --- | | HIGH | 1E-.05 | 3B1S |
| HP1576 | HYDROLASE/TRANSPORT PROTEIN ABC TRANSPORTER SYSTEM PERMEASE PROTEIN | CERTAIN | 6E-21 | 3TUI |
| HP1577 | METHIONINE ABC TRANSPORTER | CERTAIN | 1E-09 | 3TUI |
| HP1581 | LIGASE,NUCLEAR PROTEIN/ PREDICTED UNDECAPRENYL PHOSPHATE N-ACETYLGLUCOSAMINYLTRANSFERASE | CERTAIN | 3E-05 | 2XWU |
| HP1583 | 4-HYDROXYTHREONINE-4-PHOSPHATE DEHYDROGENASE(OXIDOREDUCTASE)/ PREDICTED PYRIDOXAL PHOSPHATE BIOSYNTHETIC PROTEIN A  PREDICTED PYRSPHATE BIOSYNTHETIC PROTEIN A | CERTAIN | 1E-14 | 3TSN |
| HP1584 | O-SIALOGLYCOPROTEIN ENDOPEPTIDASE(HYDROLASE) | CERTAIN | 6E-11 | 2IVN |
| HP1585 | FLAGELLAR HOOK PROTEIN(STRUCTURAL PROTEIN) | CERTAIN | 4E-06 | 1WLG |
