# Supplementary material for: Structure Based Annotation of Helicobacter pylori Strain 26695 Proteome
Source: PLoS One. 2014 Dec 30;9(12):e115020. doi: 10.1371/journal.pone.0115020 (PMC4280198; doi:10.1371/journal.pone.0115020)
Supplement: S3 Table — FUGUE results for the failed protein models mentioning template PDB_ID, confidence and Z-score. (DOC) [file pone.0115020.s003.doc]

| **Supplementary Table III. List of *H. pylori* 26695 strain proteins with fold predicted using FUGUE** | | | | |
| --- | --- | --- | --- | --- |
| **GENE NAME** | **DESCRIPTION** | **PDB ID** | **Z-SCORE** | **CONFIDENCE** |
| HP0200 | 23S RIBOSOMAL RNA | 1VOR | 12.35 | CERTAIN |
| HP0206 | CHROMOSOME PARTITION PROTEIN MUKB, LINKER | 3EUJ | 11.35 | CERTAIN |
| HP0217 | CHONDROITIN SYNTHASE | 2Z86 | 12.86 | CERTAIN |
| HP0236 | CYTOCHROME C OXIDASE, CBB3-TYPE, SUBUNIT N | 3MK7 | 12.6 | CERTAIN |
| HP0258 | REGULATOR OF SIGMA E PROTEASE | 2ZPL | 18.43 | CERTAIN |
| HP0287 | HALOALKANE DEHALOGENASE | 1B6G | 14.87 | CERTAIN |
| HP0418 | UDP-N-ACETYLMURAMOYLALANYL-D-GLUTAMYL-2,6 | 1GG4 | 15.17 | CERTAIN |
| HP0444 | DNA PRIMASE/HELICASE | INUI | 8.44 | CERTAIN |
| HP0482 | ENDONUCLEASE II | 2WSH | 8.24 | CERTAIN |
| HP0504 | RESTRICTION ENDONUCLEASE PABI | 2DVY | 12.67 | CERTAIN |
| HP0505 | RESTRICTION ENDONUCLEASE PABI | 2DVY | 29.91 | CERTAIN |
| HP0518 | PUTATIVE KETOSTEROID ISOMERASE | 3HX8 | 8.51 | CERTAIN |
| HP0554 | PUTATIVE DNA-BINDING MEMBRANE PROTEIN | 3FYM | 19.79 | CERTAIN |
| HP0564 | HYPOTHETICAL PROTEIN HP0222 | 1X93 | 10.98 | CERTAIN |
| HP0575 | PUTATIVE ZINC METALLOPROTEASE | 3B4R | 7.97 | CERTAIN |
| HP0582 | TOLA PROTEIN | 1LR0 | 9.74 | CERTAIN |
| HP0588 | FERREDOXIN | 1H98 | 13.35 | CERTAIN |
| HP0696 | TRANSCRIPTION FACTOR P53 | 2RP4 | 8.83 | CERTAIN |
| HP0752 | TRANSLOCATOR PROTEIN BIPD | 3NFT | 7.78 | CERTAIN |
| HP0828 | A1C12 SUBCOMPLEX OF F1FO ATP SYNTHASE(MEMBRANE PROTEIN) | 1C17 | 14.09 | CERTAIN |
| HP0849 | TYPE I RESTRICTION-MODIFICATION ENZYMES | 1YF2 | 10.22 | CERTAIN |
| HP0856 | PROTEIN OF UNKNOWN FUNCTION VPA0982 | 2QIP | 8.90 | CERTAIN |
| HP0879 | PROTEIN OF UNKNOWN FUNCTION VPA0982 | 2QIP | 8.90 | CERTAIN |
| HP0905 | NITROGENASE IRON PROTEIN-LIKE | 1BYI | 10.45 | CERTAIN |
| HP0918 | RNA BINDING | 1HRU | 21.09 | CERTAIN |
| HP0939 | D-METHIONINE TRANSPORT SYSTEM PERMEASE PROTEIN | 3DHW | 8.21 | CERTAIN |
| HP0973 | SOLUBLE LYTIC MUREIN TRANSGLYCOSYLASE | 1QSA | 12.23 | CERTAIN |
| HP0980 | PUTATIVE ZINC METALLOPROTEASE MJ0392 | 3B4R | 9.34 | CERTAIN |
| HP0995 | CRE RECOMBINASE | 1DRG | 25.01 | CERTAIN |
| HP1004 | MOBILIZATION PROTEIN A | 2NS6 | 8.67 | CERTAIN |
| HP1049 | HYPOTHETICAL CYTOSOLIC PROTEIN | 1G2R | 8.35 | CERTAIN |
| HP1051 | O-SIALOGLYCOPROTEIN ENDOPEPTIDASE | 2IVN | 10.48 | CERTAIN |
| HP1092 | FLAGELLAR HOOK PROTEIN FLGE | 1WLG | 6.38 | CERTAIN |
| HP1109 | FERREDOXIN | 1WTF | 9.58 | CERTAIN |
| HP1115 | E.COLI HEMOLYSIN E | 1QOY | 7.38 | CERTAIN |
| HP1127 | PSEUDOMONAS AERUGINOSA TOLA DOMAIN III, SELENO-METHIONINE DERIVATIVE | 1LR0 | 9.62 | CERTAIN |
| HP1129 | BIOPOLYMER TRANSPORT EXBD PROTEIN | 2PFU | 20.56 | CERTAIN |
| HP1131 | EPSILON SUBUNIT OF F1F0-ATP SYNTHASE N-TERMINAL DOMAIN | 1AQT | 21.73 | CERTAIN |
| HP1135 | ATP SYNTHASE SUBUNIT ALPHA, MITOCHONDRIAL | 2WSS | 17.97 | CERTAIN |
| HP1136 | ATP SYNTHASE B CHAIN | 1L2P | 13.60 | CERTAIN |
| HP1137 | ATP SYNTHASE B CHAIN | 1L2P | 13.84 | CERTAIN |
| HP1163 | POTASSIUM LARGE CONDUCTANCE CALCIUM-ACTIVATED | 1JO6 | 24.95 | CERTAIN |
| HP1204 | 23S RRNA | 3BBO | 15.81 | CERTAIN |
| HP1227 | CYTOCHROME C4 | 1ETP | 17.23 | CERTAIN |
| HP1250 | CELL WALL HYDROLASE | 2KQ8 | 15.43 | CERTAIN |
| HP1255 | PROTEIN TRANSLOCASE SUBUNIT SECA | 3DL8 | 14.66 | CERTAIN |
| HP1260 | NADH-QUINONE OXIDOREDUCTASE SUBUNIT L | 3RKO | 20.67 | CERTAIN |
| HP1265 | FORMATE DEHYDROGENASE, NITRATE-INDUCIBLE, MAJOR | 1KQF | 65.04 | CERTAIN |
| HP1279 | PHOSPHORIBOSYL-ANTHRANILATE ISOMERASE | 1PII | 26.34 | CERTAIN |
| HP1280 | ANTHRANILATE PHOSPHORIBOSYLTRANSFERASE | 1KHD | 61.16 | CERTAIN |
| HP1297 | RIBOSOMAL PROTEIN L36 FROM THERMUS THERMOPHILUS | 1DFE | 18.99 | CERTAIN |
| HP1311 | 50S RIBOSOMAL PROTEIN L29 | 1R73 | 19.97 | CERTAIN |
| HP1331 | SERUM PARAOXONASE/ARYLESTERASE 1 | 1V04 | 19.61 | CERTAIN |
| HP1336 | DNAJ MOLECULAR CHAPERONE HOMOLOGY DOMAIN | 1XBL | 15.97 | CERTAIN |
| HP1340 | BIOPOLYMER TRANSPORT EXBD PROTEIN | 2PFU | 15.65 | CERTAIN |
| HP1353 | CYTOCHROME P450 | 1N97 | 7.04 | CERTAIN |
| HP1382 | ENDONUCLEASE | 1QAE | 9.08 | CERTAIN |
| HP1392 | FIBRINOGEN BINDING PROTEIN | 3DOA | 18.87 | CERTAIN |
| HP1394 | PUTATIVE KINASE | 2AN1 | 43.33 | CERTAIN |
| HP1404 | RESTRICTION ENDONUCLEASE S SUBUNITS | 3OKG | 16.89 | CERTAIN |
| HP1440 | SERINE PROTEASE | 1ZYO | 6.57 | CERTAIN |
| HP1446 | BIOPOLYMER TRANSPORT EXBD PROTEIN | 2PFU | 15.88 | CERTAIN |
| HP1458 | PUTATIVE 42-9-9 PROTEIN | 1V9W | 23.21 | CERTAIN |
| HP1462 | UNCHARACTERIZED PROTEIN | 2LA7 | 14.24 | CERTAIN |
| HP1463 | HYPOTHETICAL PROTEIN XCC0632 | 2IQI | 22.12 | CERTAIN |
| HP1464 | CYTOPLASMIC DISTAL C-TERMINAL DOMAIN OF OCCLUDIN | 1XAW | 6.45 | CERTAIN |
| HP1473 | GLUTAMINE AMIDOTRANSFERASES CLASS-II | 1AO0 | 10.94 | CERTAIN |
| HP1477 | FLAGELLAR PROTEIN FLGA | 3FRN | 21.74 | CERTAIN |
| HP1482 | EXODEOXYRIBONUCLEASE VII SMALL SUBUNIT | 1VP7 | 7.13 | CERTAIN |
| HP1487 | PUTATIVE ABC TYPE-2 TRANSPORTER | 3CNI | 15.54 | CERTAIN |
| HP1492 | NIFU-LIKE PROTEIN HIRIP5 | 1VEH | 14.40 | CERTAIN |
| HP1493 | PEROXISOMAL TARGETING SIGNAL 1 RECEPTOR | 1FCH | 6.30 | CERTAIN |
| HP1499 | RESTRICTION ENDONUCLEASE | 2C1L | 24.22 | CERTAIN |
| HP1508 | FIXG-RELATED PROTEIN | 2R39 | 17.90 | CERTAIN |
| HP1510 | 7,8-DIHYDRONEOPTERIN TRIPHOSPHATE EPIMERASE | 1B9L | 20.16 | CERTAIN |
| HP1512 | TRANSFERRIN-BINDING PROTEIN A | 3V89 | 43.90 | CERTAIN |
| HP1540 | UBIQUINOL-CYTOCHROME C REDUCTASE IRON-SULFUR SUBUNIT, MITOCHONDRIAL | [1RIE](http://tardis.nibio.go.jp/cgi-bin/homstrad/getdata.cgi?id=1rie&pdbid=1rie&family=ISP) | 17.16 | CERTAIN |
| HP1544 | PUTATIVE PEPTIDASE M23 | 2HSI | 23.85 | CERTAIN |
| HP1546 | PUTATIVE LIPOPROTEIN B | 2JXP | 7.66 | CERTAIN |
| HP1551 | ACRIFLAVINE RESISTANCE PROTEIN B | 2RDD | 12.41 | CERTAIN |
| HP1568 | PROTEIN YHBN | 2R19 | 25.46 | CERTAIN |
| HP1575 | ESCU C-TERMINAL DOMAIN | 3BZY | 16.50 | CERTAIN |
| HP1580 | ACID PHOSPHATASE | 1D2T | 12.28 | CERTAIN |
