# Supplementary material for: Structure Based Annotation of Helicobacter pylori Strain 26695 Proteome
Source: PLoS One. 2014 Dec 30;9(12):e115020. doi: 10.1371/journal.pone.0115020 (PMC4280198; doi:10.1371/journal.pone.0115020)
Supplement: S4 Table — DALI results for the passed protein models mentioning Z-score, template PDB_ID and sequence identity. (DOCX) [file pone.0115020.s004.docx]

| **Supplementary Table IV. DALI results for *H. pylori* 26695 strain proteins with known PDB and qualified models** | | | | | |
| --- | --- | --- | --- | --- | --- |
| **Gene name** | **Protein length** | **Z-score** | **PDB_ID** | **Sequence identity (%)** | **Description** |
| HP0001 | 138 | 24.9 | [2JR0](http://ekhidna.biocenter.helsinki.fi/dali/daliquery?pdbid=2jr0&chainid=A) | 34 | N UTILIZATION SUBSTANCE PROTEIN B HOMOLOG |
| HP0002 | 156 | 28.3 | 1RVV | 49 | RIBOFLAVIN SYNTHASE |
| HP0003 | 276 | 46.3 | [2NWR](http://ekhidna.biocenter.helsinki.fi/dali/daliquery?pdbid=2nwr&chainid=A) | 52 | 2-DEHYDRO-3-DEOXYPHOSPHOOCTONATE ALDOLASE |
| HP0005 | 227 | 39.4 | [3LDV](http://ekhidna.biocenter.helsinki.fi/dali/daliquery?pdbid=3ldv&chainid=A) | 34 | OROTIDINE 5'-PHOSPHATE DECARBOXYLASE |
| HP0006 | 276 | 39.7 | 1IHO | 44 | PANTOATE--BETA-ALANINE LIGASE |
| HP0010 | 546 | 5.1 | [1WE3](http://ekhidna.biocenter.helsinki.fi/dali/daliquery?pdbid=1we3&chainid=F) | 61 | CPN60(GROEL) |
| HP0012 | 559 | 58.7 | 2AU3 | 32 | DNA PRIMASE |
| HP0014 | 275 | 19.2 | [2Q1A](http://ekhidna.biocenter.helsinki.fi/dali/daliquery?pdbid=2q1a&chainid=X) | 16 | 2-KETO-3-DEOXY-D-ARABINONATE DEHYDRATASE |
| HP0020 | 405 | 56 | [3N29](http://ekhidna.biocenter.helsinki.fi/dali/daliquery?pdbid=3n29&chainid=A) | 50 | CARBOXYNORSPERMIDINE DECARBOXYLASE |
| HP0026 | 426 | 62.7 | [2H12](http://ekhidna.biocenter.helsinki.fi/dali/daliquery?pdbid=2h12&chainid=D) | 46 | CITRATE SYNTHASE |
| HP0027 | 425 | 64.9 | [3DMS](http://ekhidna.biocenter.helsinki.fi/dali/daliquery?pdbid=3dms&chainid=A) | 71 | ISOCITRATE DEHYDROGENASE [NADP] |
| HP0029 | 218 | 42.9 | [3QXC](http://ekhidna.biocenter.helsinki.fi/dali/daliquery?pdbid=3qxc&chainid=A) | 100 | DETHIOBIOTIN SYNTHETASE |
| HP0031 | 137 | 14.6 | 1MJH | 18 | PROTEIN (ATP-BINDING DOMAIN OF PROTEIN MJ0577) |
| HP0032 | 91 | 18.8 | [3O2H](http://ekhidna.biocenter.helsinki.fi/dali/daliquery?pdbid=3o2h&chainid=A) | 34 | ATP-DEPENDENT CLP PROTEASE ADAPTOR PROTEIN CLPS |
| HP0033 | 741 | 43.5 | 1QVR | 35 | CLPB PROTEIN |
| HP0035 | 97 | 15.5 | [3F42](http://ekhidna.biocenter.helsinki.fi/dali/daliquery?pdbid=3f42&chainid=A) | 93 | PROTEIN HP0035 |
| HP0042 | 233 | 30.1 | [2BHV](http://ekhidna.biocenter.helsinki.fi/dali/daliquery?pdbid=2bhv&chainid=A) | 100 | COMB10 |
| HP0043 | 470 | 47.3 | [2QH5](http://ekhidna.biocenter.helsinki.fi/dali/daliquery?pdbid=2qh5&chainid=A) | 100 | MANNOSE-6-PHOSPHATE ISOMERASE |
| HP0044 | 381 | 54.2 | [1DB3](http://ekhidna.biocenter.helsinki.fi/dali/daliquery?pdbid=1db3&chainid=A) | 61 | GDP-MANNOSE 4,6-DEHYDRATASE |
| HP0045 | 310 | 49.3 | [1E6U](http://ekhidna.biocenter.helsinki.fi/dali/daliquery?pdbid=1e6u&chainid=A) | 37 | GDP-FUCOSE SYNTHETASE |
| HP0047 | 332 | 55.3 | [2Z1U](http://ekhidna.biocenter.helsinki.fi/dali/daliquery?pdbid=2z1u&chainid=A) | 32 | HYDROGENASE EXPRESSION/FORMATION PROTEIN |
| HP0049 | 330 | 61.1 | [3HVM](http://ekhidna.biocenter.helsinki.fi/dali/daliquery?pdbid=3hvm&chainid=A) | 96 | AGMATINE DEIMINASE |
| HP0051 | 355 | 50.1 | [2C7P](http://ekhidna.biocenter.helsinki.fi/dali/daliquery?pdbid=2c7p&chainid=A) | 28 | HHAI DNA METHYLTRANSFERASE |
| HP0056 |  | 36.1 | 4F9I | 31 | PROLINE DEHYDROGENASE/DELTA-1-PYRROLINE-5-CARBOXYLATE DEHYDROGENASE |
| HP0062 | 86 | 14.3 | 3FX7 | 100 | PUTATIVE UNCHARACTERIZED PROTEIN |
| HP0064 | 139 | 11.6 | 3D5P | 18 | PUTATIVE GLUCAN SYNTHESIS REGULATOR OF SMI1/KNR4 |
| HP0067 | 265 | 45.3 | 3SF5 | 100 | UREASE ACCESSORY PROTEIN UREF |
| HP0068 | 199 | 34.4 | [2HF9](http://ekhidna.biocenter.helsinki.fi/dali/daliquery?pdbid=2hf9&chainid=A) | 35 | PROBABLE HYDROGENASE NICKEL INCORPORATION |
| HP0069 | 254 | 40 | [2WGL](http://ekhidna.biocenter.helsinki.fi/dali/daliquery?pdbid=2wgl&chainid=A) | 100 | UREASE ACCESSORY PROTEIN URE |
| HP0070 | 170 | 27.9 | 3TJ8 | 100 | UREASE ACCESSORY PROTEIN UREE |
| HP0072 | 569 | 22.5 | 3QGA | 54 | FUSION OF UREASE BETA AND GAMMA SUBUNITS |
| HP0073 | 238 | 28 | 1E9Y | 100 | UREASE SUBUNIT ALPHA |
| HP0075 | 445 | 65.3 | [3I3W](http://ekhidna.biocenter.helsinki.fi/dali/daliquery?pdbid=3i3w&chainid=A) | 34 | PHOSPHOGLUCOSAMINE MUTASE |
| HP0084 | 141 | 24 | [3FIN](http://ekhidna.biocenter.helsinki.fi/dali/daliquery?pdbid=3fin&chainid=N) | 52 | 50S RIBOSOMAL PROTEIN L27 |
| HP0087 | 457 | 61.5 | [3M1U](http://ekhidna.biocenter.helsinki.fi/dali/daliquery?pdbid=3m1u&chainid=A) | 29 | PUTATIVE GAMMA-D-GLUTAMYL-L-DIAMINO ACID ENDOPEPTIDASE |
| HP0089 | 231 | 44.9 | [3BL6](http://ekhidna.biocenter.helsinki.fi/dali/daliquery?pdbid=3bl6&chainid=A) | 37 | 5'-METHYLTHIOADENOSINE NUCLEOSIDASE/S-ADENOSYLHOMOCYSTEINE NUCLEOSIDASE |
| HP0090 | 309 | 56.4 | [2H1Y](http://ekhidna.biocenter.helsinki.fi/dali/daliquery?pdbid=2h1y&chainid=A) | 98 | MALONYL COENZYME A-ACYL CARRIER PROTEIN TRANSACYL |
| HP0096 | 314 | 42.7 | [1WWK](http://ekhidna.biocenter.helsinki.fi/dali/daliquery?pdbid=1wwk&chainid=A) | 34 | PHOSPHOGLYCERATE DEHYDROGENASE |
| HP0098 | 486 | 56.8 | 1KL7 | 30 | THREONINE SYNTHASE |
| HP0104 | 581 | 43.6 | [2Z1A](http://ekhidna.biocenter.helsinki.fi/dali/daliquery?pdbid=2z1a&chainid=A) | 23 | 5'-NUCLEOTIDASE |
| HP0105 | 155 | 29.9 | [1J6X](http://ekhidna.biocenter.helsinki.fi/dali/daliquery?pdbid=1j6x&chainid=A) | 93 | AUTOINDUCER-2 PRODUCTION PROTEIN LUXS |
| HP0106 | 380 | 62.9 | [3E6G](http://ekhidna.biocenter.helsinki.fi/dali/daliquery?pdbid=3e6g&chainid=A) | 56 | CYSTATHIONINE GAMMA-LYASE-LIKE PROTEIN |
| HP0107 | 306 | 52 | 2Q3B | 43 | CYSTEINE SYNTHASE A |
| HP0109 | 620 | 52.8 | [1DKG](http://ekhidna.biocenter.helsinki.fi/dali/daliquery?pdbid=1dkg&chainid=D) | 57 | NUCLEOTIDE EXCHANGE FACTOR GRPE |
| HP0116 | 736 | 36.3 | [2GAJ](http://ekhidna.biocenter.helsinki.fi/dali/daliquery?pdbid=2gaj&chainid=A) | 37 | DNA TOPOISOMERASE I |
| HP0121 | 812 | 53.7 | [2OLS](http://ekhidna.biocenter.helsinki.fi/dali/daliquery?pdbid=2ols&chainid=A) | 52 | PHOSPHOENOLPYRUVATE SYNTHASE |
| HP0123 | 612 | 61.5 | [1QF6](http://ekhidna.biocenter.helsinki.fi/dali/daliquery?pdbid=1qf6&chainid=A) | 41 | THREONINE TRNA |
| HP0124 | 203 | 16.6 | 1TIF | 50 | TRANSLATION INITIATION FACTOR 3 |
| HP0133 | 413 | 34.4 | 3L1L | 13 | ARGININE/AGMATINE ANTIPORTER |
| HP0134 | 449 | 66.5 | [2B7O](http://ekhidna.biocenter.helsinki.fi/dali/daliquery?pdbid=2b7o&chainid=A) | 44 | 3-DEOXY-D-ARABINO-HEPTULOSONATE  7-PHOSPHATE SYNTHETASE |
| HP0136 | 152 | 29.1 | [3DRN](http://ekhidna.biocenter.helsinki.fi/dali/daliquery?pdbid=3drn&chainid=A) | 37 | PEROXIREDOXIN,BACTERIOFERRITIN COMIGRATORY PROTEIN |
| HP0142 | 328 | 38.7 | 1MUN | 38 | ADENINE GLYCOSYLASE |
| HP0144 | 488 | 65.2 | 3MK7 | 44 | CYTOCHROME C OXIDASE |
| HP0152 | 287 | 45.7 | 3A3U | 26 | MENAQUINONE BIOSYNTHETIC ENZYME |
| HP0153 | 347 | 50.5 | [2G88](http://ekhidna.biocenter.helsinki.fi/dali/daliquery?pdbid=2g88&chainid=A) | 60 | PROTEIN RECA |
| HP0154 | 426 | 69.5 | [2PA6](http://ekhidna.biocenter.helsinki.fi/dali/daliquery?pdbid=2pa6&chainid=A) | 54 | ENOLASE |
| HP0157 | 162 | 33.6 | [3N2E](http://ekhidna.biocenter.helsinki.fi/dali/daliquery?pdbid=3n2e&chainid=A) | 100 | SHIKIMATE KINASE |
| HP0160 | 306 | 15.9 | 1KLX | 29 | CYSTEINE RICH PROTEIN B |
| HP0162 | 240 | 18.9 | 4F3Q | 39 | TRANSCRIPTIONAL REGULATORY PROTEIN CBU_1566 |
| HP0163 | 323 | 47.5 | 1W1Z | 43 | DELTA-AMINOLEVULINIC ACID DEHYDRATASE |
| HP0166 | 225 | 31.5 | [1KGS](http://ekhidna.biocenter.helsinki.fi/dali/daliquery?pdbid=1kgs&chainid=A) | 33 | DNA BINDING RESPONSE REGULATOR D |
| HP0171 | 363 | 50.4 | [1GQE](http://ekhidna.biocenter.helsinki.fi/dali/daliquery?pdbid=1gqe&chainid=A) | 49 | RELEASE FACTOR 2 |
| HP0172 | 391 | 48 | [1G8L](http://ekhidna.biocenter.helsinki.fi/dali/daliquery?pdbid=1g8l&chainid=A) | 31 | MOLYBDOPTERIN BIOSYNTHESIS MOEA PROTEIN |
| HP0176 | 307 | 52.6 | [3C4U](http://ekhidna.biocenter.helsinki.fi/dali/daliquery?pdbid=3c4u&chainid=A) | 99 | FRUCTOSE-BISPHOSPHATE ALDOLASE |
| HP0178 | 340 | 45.5 | [2WQP](http://ekhidna.biocenter.helsinki.fi/dali/daliquery?pdbid=2wqp&chainid=A) | 30 | POLYSIALIC ACID CAPSULE BIOSYNTHESIS PROTEIN SIAC |
| HP0179 | 213 | 36.9 | [2PCJ](http://ekhidna.biocenter.helsinki.fi/dali/daliquery?pdbid=2pcj&chainid=A) | 36 | LIPOPROTEIN-RELEASING SYSTEM ATP-BINDING PROTEIN |
| HP0182 | 501 | 54.7 | [1BBU](http://ekhidna.biocenter.helsinki.fi/dali/daliquery?pdbid=1bbu&chainid=A) | 50 | PROTEIN (LYSYL-TRNA SYNTHETASE) |
| HP0183 | 416 | 65.6 | [3N0L](http://ekhidna.biocenter.helsinki.fi/dali/daliquery?pdbid=3n0l&chainid=A) | 66 | SERINE HYDROXYMETHYLTRANSFERASE |
| HP0184 | 180 | 9.2 | 2IRY | 16 | DNA LIGASE-LIKE PROTEIN RV0938/MT0965 |
| HP0191 | 245 | 37.3 | [2BS2](http://ekhidna.biocenter.helsinki.fi/dali/daliquery?pdbid=2bs2&chainid=B) | 70 | QUINOL-FUMARATE REDUCTASE FLAVOPROTEIN SUBUNIT A |
| HP0192 | 714 | 62.9 | [2BS2](http://ekhidna.biocenter.helsinki.fi/dali/daliquery?pdbid=2bs2&chainid=D) | 71 | QUINOL-FUMARATE REDUCTASE FLAVOPROTEIN SUBUNIT A |
| HP0194 | 234 | 45 | [2JGQ](http://ekhidna.biocenter.helsinki.fi/dali/daliquery?pdbid=2jgq&chainid=A) | 100 | TRIOSEPHOSPHATE ISOMERASE |
| HP0195 | 275 | 48.3 | [2PD4](http://ekhidna.biocenter.helsinki.fi/dali/daliquery?pdbid=2pd4&chainid=A) | 100 | ENOYL-[ACYL-CARRIER-PROTEIN] REDUCTASE [NADH] |
| HP0196 | 336 | 38.1 | [2IU9](http://ekhidna.biocenter.helsinki.fi/dali/daliquery?pdbid=2iu9&chainid=A) | 32 | UDP-3-O-[3-HYDROXYMYRISTOYL] GLUCOSAMINE N-ACYLTRANSFERASE |
| HP0197 | 385 | 65.1 | [1P7L](http://ekhidna.biocenter.helsinki.fi/dali/daliquery?pdbid=1p7l&chainid=A) | 61 | S-ADENOSYLMETHIONINE SYNTHETASE |
| HP0198 | 137 | 28.8 | [1NHK](http://ekhidna.biocenter.helsinki.fi/dali/daliquery?pdbid=1nhk&chainid=R) | 65 | NUCLEOSIDE DIPHOSPHATE KINASE |
| HP0201 | 338 | 50.4 | 1VI1 | 38 | 41FATTY ACID/PHOSPOLIPID SYNTHESIS PROTIEN |
| HP0202 | 331 | 58.2 | [1HNJ](http://ekhidna.biocenter.helsinki.fi/dali/daliquery?pdbid=1hnj&chainid=A) | 45 | BETA-KETOACYL-ACYL CARRIER PROTEIN SYNTHASE III |
| HP0207 | 412 | 31 | 2PH1 | 34 | NUCLEOTIDE-BINDING PROTEIN |
| HP0210 | 621 | 43 | [2IOP](http://ekhidna.biocenter.helsinki.fi/dali/daliquery?pdbid=2iop&chainid=C) | 45 | CHAPERONE PROTEIN HTPG |
| HP0211 | 250 | 16.1 | 1KLX | 41 | CYSTEINE RICH PROTEIN B |
| HP0212 | 383 | 54.9 | [1VGY](http://ekhidna.biocenter.helsinki.fi/dali/daliquery?pdbid=1vgy&chainid=A) | 39 | SUCCINYL-DIAMINOPIMELATE DESUCCINYLASE |
| HP0213 | 621 | 25.9 | 2CUL | 25 | GLUCOSE-INHIBITED DIVISION PROTEIN A-RELATED PROTEIN,  PROBABLE OXIDOREDUCTASE |
| HP0216 | 368 | 60.9 | [2C82](http://ekhidna.biocenter.helsinki.fi/dali/daliquery?pdbid=2c82&chainid=A) | 33 | 1-DEOXY-D-XYLULOSE 5-PHOSPHATE REDUCTOISOMERASE |
| HP0218 | 183 | 19 | 3N08 | 26 | PUTATIVE PHOSPHATIDYLETHANOLAMINE-BINDING PROTEIN |
| HP0220 | 387 | 63.9 | [3LVM](http://ekhidna.biocenter.helsinki.fi/dali/daliquery?pdbid=3lvm&chainid=A) | 43 | CYSTEINE DESULFURASE |
| HP0222 | 73 | 6.4 | 2AY0 | 19 | BIFUNCTIONAL PUTA PROTEIN |
| HP0224 | 359 | 46 | [3E0M](http://ekhidna.biocenter.helsinki.fi/dali/daliquery?pdbid=3e0m&chainid=A) | 59 | PEPTIDE METHIONINE SULFOXIDE REDUCTASE MSRA/MSRB |
| HP0230 | 243 | 37.9 | [3JTJ](http://ekhidna.biocenter.helsinki.fi/dali/daliquery?pdbid=3jtj&chainid=A) | 37 | 3-DEOXY-MANNO-OCTULOSONATE CYTIDYLYLTRANSFERASE |
| HP0231 | 265 | 11.5 | 1JZO | 14 | THIOL:DISULFIDE INTERCHANGE PROTEIN DSBC |
| HP0233 | 390 | 50.5 | [2IOB](http://ekhidna.biocenter.helsinki.fi/dali/daliquery?pdbid=2iob&chainid=B) | 19 | BIFUNCTIONAL GLUTATHIONYLSPERMIDINE |
| HP0235 | 355 | 13.7 | 1KLX | 25 | CYSTEINE RICH PROTEIN B |
| HP0237 | 306 | 42.8 | [3ECR](http://ekhidna.biocenter.helsinki.fi/dali/daliquery?pdbid=3ecr&chainid=A) | 37 | PORPHOBILINOGEN DEAMINASE |
| HP0238 | 577 | 49.9 | 2J31 | 50 | PROLYL-TRNA SYNTHETASE |
| HP0239 | 449 | 44.7 | [1GPJ](http://ekhidna.biocenter.helsinki.fi/dali/daliquery?pdbid=1gpj&chainid=A) | 23 | GLUTAMYL-TRNA REDUCTASE |
| HP0240 | 307 | 46.9 | [1WMW](http://ekhidna.biocenter.helsinki.fi/dali/daliquery?pdbid=1wmw&chainid=A) | 23 | GERANYLGERANYL DIPHOSPHATE SYNTHETASE |
| HP0243 | 144 | 29 | [1JI4](http://ekhidna.biocenter.helsinki.fi/dali/daliquery?pdbid=1ji4&chainid=D) | 100 | NEUTROPHIL-ACTIVATING PROTEIN A |
| HP0247 | 492 | 58.6 | [1S2M](http://ekhidna.biocenter.helsinki.fi/dali/daliquery?pdbid=1s2m&chainid=A) | 34 | PUTATIVE ATP-DEPENDENT RNA HELICASE DHH1 |
| HP0255 | 411 | 62.4 | [1ADE](http://ekhidna.biocenter.helsinki.fi/dali/daliquery?pdbid=1ade&chainid=A) | 47 | ADENYLOSUCCINATE SYNTHETASE |
| HP0263 | 252 | 31.4 | 1NW6 | 22 | N6-ADENINE DNA METHYLTRANSFERASE RSRI |
| HP0264 | 856 | 45.8 | [1QVR](http://ekhidna.biocenter.helsinki.fi/dali/daliquery?pdbid=1qvr&chainid=A) | 49 | CLPB PROTEIN |
| HP0266 | 378 | 34 | 1XRF | 22 | DIHYDROOROTASE |
| HP0267 | 409 | 53.2 | [4F0S](http://ekhidna.biocenter.helsinki.fi/dali/daliquery?pdbid=4f0s&chainid=A) | 22 | 5-METHYLTHIOADENOSINE/S-ADENOSYLHOMOCYSTEINE  DEAMINASE |
| HP0269 | 437 | 39.6 | 2QGQ | 29 | A RADICAL S-ADENOSYLMETHIONINE METHYLTHIOTRANSFERASE |
| HP0277 | 84 | 19 | [2FGO](http://ekhidna.biocenter.helsinki.fi/dali/daliquery?pdbid=2fgo&chainid=A) | 81 | FERREDOXIN |
| HP0278 | 484 | 54.5 | [3HI0](http://ekhidna.biocenter.helsinki.fi/dali/daliquery?pdbid=3hi0&chainid=A) | 23 | PUTATIVE EXOPOLYPHOSPHATASE |
| HP0279 | 340 | 49.5 | [2GT1](http://ekhidna.biocenter.helsinki.fi/dali/daliquery?pdbid=2gt1&chainid=A) | 27 | LIPOPOLYSACCHARIDE HEPTOSYLTRANSFERASE-1 |
| HP0281 | 371 | 64 | [3GC5](http://ekhidna.biocenter.helsinki.fi/dali/daliquery?pdbid=3gc5&chainid=A) | 43 | QUEUINE TRNA-RIBOSYLTRANSFERASE |
| HP0290 | 405 | 66.6 | [2QGH](http://ekhidna.biocenter.helsinki.fi/dali/daliquery?pdbid=2qgh&chainid=A) | 98 | DIAMINOPIMELATE DECARBOXYLASE |
| HP0293 | 559 | 52.7 | 1QDL | 27 | ANTHRANILATE SYNTHASE |
| HP0294 | 339 | 56.9 | [2PLQ](http://ekhidna.biocenter.helsinki.fi/dali/daliquery?pdbid=2plq&chainid=A) | 77 | ALIPHATIC AMIDASE |
| HP0297 | 88 | 13.9 | [2ZJR](http://ekhidna.biocenter.helsinki.fi/dali/daliquery?pdbid=2zjr&chainid=T) | 57 | RIBOSOMAL 23S RNA |
| HP0298 | 549 | 57 | [1DPE](http://ekhidna.biocenter.helsinki.fi/dali/daliquery?pdbid=1dpe&chainid=A) | 39 | DIPEPTIDE-BINDING PROTEIN |
| HP0301 | 287 | 40 | 3DHW | 38 | D-METHIONINE TRANSPORT SYSTEM PERMEASE PROTEIN ME |
| HP0302 | 268 | 33.1 | [2YYZ](http://ekhidna.biocenter.helsinki.fi/dali/daliquery?pdbid=2yyz&chainid=A) | 38 | SUGAR ABC TRANSPORTER, ATP-BINDING PROTEIN |
| HP0303 | 360 | 40.5 | [1LNZ](http://ekhidna.biocenter.helsinki.fi/dali/daliquery?pdbid=1lnz&chainid=A) | 47 | SPO0B-ASSOCIATED GTP-BINDING PROTEIN |
| HP0304 | 329 | 18.6 | 3NNB | 19 | ALGINATE LYASE |
| HP0305 | 184 | 19.2 | 2X32 | 15 | CELLULOSE-BINDING PROTEIN |
| HP0306 | 430 | 69.2 | 3BS8 | 49 | GLUTAMATE-1-SEMIALDEHYDE 2,1-AMINOMUTASE |
| HP0309 | 292 | 32.9 | 1UF8 | 25 | N-CARBAMYL-D-AMINO ACID AMIDOHYDROLASE |
| HP0310 | 293 | 37.8 | 1Z7A | 24 | POLYSACCHARIDE DEACETYLASE FAMILY PROTEIN |
| HP0312 | 321 | 10.2 | 2WSM | 18 | GTP-DEPENDENT HYDROGENASE EXPRESSION/FORMATION PROTEIN (HYPB) |
| HP0315 | 94 | 22.4 | 3UI3 | 100 | IMMUNOGLOBULIN G-BINDING PROTEIN G, VIRULENCE-ASSOCIATED PROTEIN |
| HP0318 | 251 | 35.5 | 3GAS | 96 | HEME OXYGENASE |
| HP0319 | 541 | 47.2 | 1F7U | 20 | ARGINYL-TRNA SYNTHETASE |
| HP0321 | 206 | 31.6 | 1S96 | 41 | GUANYLATE KINASE |
| HP0329 | 260 | 43.3 | 1XNG | 100 | NH(3)-DEPENDENT NAD(+) SYNTHETASE |
| HP0330 | 330 | 46.2 | 1NP3 | 49 | KETOL-ACID REDUCTOISOMERASE |
| HP0331 | 268 | 44.7 | 1ION | 35 | PROBABLE CELL DIVISION INHIBITOR MIND |
| HP0332 | 77 | 13.1 | 3KU7 | 100 | CELL DIVISION TOPOLOGICAL SPECIFICITY FACTOR |
| HP0333 | 270 | 37.9 | 3MAJ | 25 | PUTATIVE DNA PROCESSING PROTEIN |
| HP0334 | 134 | 13.5 | 1VHX | 25 | PUTATIVE HOLLIDAY JUNCTION RESOLVASE |
| HP0335 | 62 | 6.5 | 1KLX | 22 | CYSTEINE RICH PROTEIN B |
| HP0336 | 138 | 26.5 | 1KLX | 100 | CYSTEINE RICH PROTEIN B |
| HP0347 | 298 | 37.8 | 1V9F | 24 | RIBOSOMAL LARGE SUBUNIT PSEUDOURIDINE SYNTHASE D |
| HP0348 | 516 | 46.6 | 2ZXP | 29 | SINGLE-STRANDED DNA SPECIFIC EXONUCLEASE RECJ |
| HP0349 | 538 | 58.1 | 1VCO | 48 | CTP SYNTHETASE |
| HP0352 | 343 | 28.4 | 3USW | 100 | FLAGELLAR MOTOR SWITCH PROTEIN |
| HP0354 | 618 | 52.7 | 2O1S | 40 | 1-DEOXY-D-XYLULOSE-5-PHOSPHATE SYNTHASE |
| HP0355 | 602 | 51.6 | 3CB4 | 54 | GTP-BINDING PROTEIN LEPA |
| HP0357 |  | 43.8 | 3SVT | 18 | SHORT-CHAIN TYPE DEHYDROGENASE/REDUCTASE |
| HP0360 | 344 | 53.7 | 2C20 | 37 | UDP-GLUCOSE 4-EPIMERASE |
| HP0361 | 242 | 34.7 | 1VS3 | 32 | TRNA PSEUDOURIDINE SYNTHASE A |
| HP0363 | 209 | 41.9 | 3LBF | 39 | PROTEIN-L-ISOASPARTATE O-METHYLTRANSFERASE |
| HP0364 | 341 | 48.7 | 2RCC | 30 | RIBONUCLEOSIDE-DIPHOSPHATE REDUCTASE SUBUNIT BETA |
| HP0366 | 375 | 66.1 | [2FN6](http://ekhidna.biocenter.helsinki.fi/dali/daliquery?pdbid=2fn6&chainid=A) | 100 | AMINOTRANSFERASE |
| HP0370 | 458 | 67.8 | 2VQD | 52 | BIOTIN CARBOXYLASE |
| HP0372 | 190 | 27.8 | 2QXX | 28 | DEOXYCYTIDINE TRIPHOSPHATE DEAMINASE |
| HP0374 | 226 | 29 | 2EGV | 21 | RRNA METHYLTRANSFERASE |
| HP0376 | 334 | 47.2 | 3HCN | 32 | FERROCHELATASE, MITOCHONDRIAL |
| HP0379 | 425 | 54.2 | 2NZW | 92 | ALPHA1,3-FUCOSYLTRANSFERASE |
| HP0380 | 448 | 64.8 | 2BMA | 57 | GLUTAMATE DEHYDROGENASE |
| HP0381 | 276 | 38.7 | 1NV8 | 34 | HEMK PROTEIN |
| HP0384 | 250 | 16.7 | 1UTA | 21 | CELL DIVISION PROTEIN FTSN |
| HP0388 | 243 | 37.8 | 1IM8 | 37 | METHYLTRANSFERASE |
| HP0389 | 213 | 33.3 | 1BSM | 29 | SUPEROXIDE DISMUTASE |
| HP0390 | 166 | 35.3 | 2JSY | 42 | THIOL PEROXIDASE |
| HP0391 | 165 | 27.1 | 2QDL | 28 | CHEMOTAXIS SIGNAL TRANSDUCTION PROTEIN |
| HP0393 | 311 | 21.8 | 3G19 | 33 | RESPONSE REGULATOR |
| HP0395 | 222 | 17.5 | 4A3Q | 12 | ALANINE RACEMASE 1 |
| HP0397 | 524 | 55.6 | 1YGY | 30 | D-3-PHOSPHOGLYCERATE DEHYDROGENASE |
| HP0400 | 274 | 39.9 | 3DNF | 43 | 4-HYDROXY-3-METHYLBUT-2-ENYL DIPHOSPHATE REDUCTASE |
| HP0401 | 429 | 65.8 | 1RF6 | 35 | 5-ENOLPYRUVYLSHIKIMATE-3-PHOSPHATE SYNTHASE |
| HP0402 | 764 | 21.3 | 3ICA | 19 | PHENYLALANYL-TRNA SYNTHETASE BETA CHAIN |
| HP0403 | 328 | 37 | 2RHS | 52 | PHENYLALANYL-TRNA SYNTHETASE ALPHA CHAIN |
| HP0404 | 104 | 5.6 | [1KPC](http://ekhidna.biocenter.helsinki.fi/dali/daliquery?pdbid=1kpc&chainid=B) | 29 | HUMAN PROTEIN KINASE C INTERACTING PROTEIN 1 |
| HP0405 | 440 | 53.8 | 1T3I | 24 | PROBABLE CYSTEINE DESULFURASE |
| HP0407 | 796 | 58.5 | 1E5V | 42 | DMSO REDUCTASE |
| HP0409 | 508 | 52.7 | 2YWB | 51 | GMP SYNTHASE |
| HP0410 | 249 | 32.6 | [3BGH](http://ekhidna.biocenter.helsinki.fi/dali/daliquery?pdbid=3bgh&chainid=A) | 100 | PUTATIVE NEURAMINYLLACTOSE-BINDING HEMAGGLUTININ |
| HP0414 | 138 | 22.7 | 2EC2 | 33 | TRANSPOSASE |
| HP0416 | 389 | 43.2 | 1L1E | 36 | MYCOLIC ACID SYNTHASE |
| HP0417 | 650 | 49.2 | 1RQG | 25 | METHIONYL-TRNA SYNTHETASE |
| HP0421 | 389 | 36.4 | [3QHP](http://ekhidna.biocenter.helsinki.fi/dali/daliquery?pdbid=3qhp&chainid=A) | 100 | CATALYTIC DOMAIN OF CHOLESTRAL ALPHA GLUCOSYL TRANSFERASE |
| HP0422 | 615 | 47.6 | 3N2O | 32 | BIOSYNTHETIC ARGININE DECARBOXYLASE |
| HP0437 | 142 | 23.9 | 2FYX | 27 | TRANSPOSASE |
| HP0453 | 1021 | 10 | 1WFQ | 24 | COLD-SHOCK DOMAIN |
| HP0468 | 495 | 11.8 | 2BS2 | 14 | QUINOL-FUMARATE REDUCTASE FLAVOPROTEIN SUBUNIT A |
| HP0470 | 578 | 46.6 | 2QR4 | 24 | PEPTIDASE M3B, OLIGOENDOPEPTIDASE F |
| HP0471 | 416 | 48.2 | 3CE2 | 24 | PUTATIVE PEPTIDASE |
| HP0473 | 246 | 39.2 | 1ATG | 37 | PERIPLASMIC MOLYBDATE-BINDING PROTEIN |
| HP0475 | 265 | 34.1 | 2ONK | 43 | MOLYBDATE/TUNGSTATE ABC TRANSPORTER, ATP-BINDING |
| HP0476 | 463 | 47.7 | 2CFO | 41 | GLUTAMYL-TRNA SYNTHETASE |
| HP0480 | 599 | 37.5 | 2YWH | 29 | GTP-BINDING PROTEIN LEPA |
| HP0485 | 314 | 42.7 | 3RE8 | 20 | CATALASE |
| HP0492 | 278 | 33.5 | 2I9I | 100 | HYPOTHETICAL PROTEIN |
| HP0494 | 422 | 55.2 | 3UAG | 26 | UDP-N-ACETYLMURAMOYL-L-ALANINE:D-GLUTAMATE LIGASE |
| HP0495 | 86 | 7.6 | 3PHT | 11 | PUTATIVE NICKEL-RESPONSIVE REGULATOR |
| HP0496 | 133 | 26.5 | [2PZH](http://ekhidna.biocenter.helsinki.fi/dali/daliquery?pdbid=2pzh&chainid=A) | 100 | YBGC THIOESTERASE |
| HP0497 |  | 61.3 | 2A65 | 24 | NA(+):NEUROTRANSMITTER SYMPORTER (SNF FAMILY) |
| HP0498 | 442 | 59 | 3MPQ | 25 | TRANSPORTER |
| HP0500 | 374 | 45.7 | 1UNN | 20 | DNA POLYMERASE III BETA SUBUNIT |
| HP0501 | 773 | 53.5 | [1EI1-B](http://ekhidna.biocenter.helsinki.fi/dali/daliquery?pdbid=1ei1&chainid=B) | 46 | DNA GYRASE B |
| HP0507 | 212 | 23.2 | 1G0S | 19 | ADP-RIBOSE PYROPHOSPHATASE |
| HP0509 | 459 | 49.9 | 1WVE | 19 | 4-CRESOL DEHYDROGENASE |
| HP0510 | 254 | 38.6 | 3IJP | 38 | DIHYDRODIPICOLINATE REDUCTASE |
| HP0512 | 481 | 64 | 1F52 | 48 | GLUTAMINE SYNTHETASE |
| HP0514 | 150 | 19.6 | 1DIV | 41 | RIBOSOMAL PROTEIN L9 |
| HP0515 | 180 | 35.4 | 1G3K | 56 | ATP-DEPENDENT PROTEASE HSLV |
| HP0516 | 443 | 46.4 | 1E94 | 48 | HEAT SHOCK PROTEIN HSLV |
| HP0517 | 302 | 40.2 | 1WF3 | 28 | GTP-BINDING PROTEIN |
| HP0525 | 330 | 49.6 | 1G6O | 100 | CAG-ALPHA |
| HP0526 | 199 | 28.5 | 1S2X | 95 | CAG-Z |
| HP0534 | 196 | 28.2 | 2G3V | 94 | CAG PATHOGENICITY ISLAND PROTEIN 13 |
| HP0549 | 255 | 46.7 | 2JFZ | 94 | GLUTAMATE RACEMASE |
| HP0550 | 438 | 64.3 | 310O | 53 | TRANSCRIPTION TERMINATION FACTOR RHO |
| HP0553 | 227 | 33.4 | 1GZ0 | 24 | RRNA METHYLTRANSFERASE YJFH |
| HP0557 | 312 | 49.7 | 2F9Y | 50 | ACETYL-COA CARBOXYLASE |
| HP0558 | 412 | 77.7 | 1J3N | 50 | 3-OXOACYL-(ACYL-CARRIER PROTEIN) SYNTHASE II |
| HP0559 | 78 | 18.3 | 2EHS | 64 | ACYL CARRIER PROTEIN |
| HP0561 | 247 | 39.4 | 3F9I | 49 | 3-OXOACYL-[ACYL-CARRIER-PROTEIN] REDUCTASE |
| HP0566 | 273 | 38.3 | 3EKM | 31 | DIAMINOPIMELATE EPIMERASE |
| HP0569 | 366 | 50.1 | 1JAL | 56 | PROBABLE GTP-BINDING PROTEIN |
| HP0570 | 496 | 58.7 | 1GYT | 31 | CYTOSOL AMINOPEPTIDASE |
| HP0572 | 179 | 33.5 | 2DY0 | 48 | ADENINE PHOSPHORIBOSYLTRANSFERASE |
| HP0574 | 151 | 30.4 | 1O1X | 45 | RIBOSE-5-PHOSPHATE ISOMERASE |
| HP0577 | 292 | 47.3 | 1B0A | 47 | BIFUNCTIONAL 5,10, METHYLENE-TETRAHYDROPHOLATE DEHYDROGENASE |
| HP0581 | 339 | 56 | 2Z26 | 30 | DIHYDROOROTASE |
| HP0585 | 218 | 35 | 1ORN | 41 | ENDONUCLEASE III |
| HP0590 | 273 | 37.8 | [2C3Y](http://ekhidna.biocenter.helsinki.fi/dali/daliquery?pdbid=2c3y&chainid=A) | 21 | PYRUVATE-FERREDOXIN OXIDOREDUCTASE |
| HP0596 | 192 | 6.8 | 3IEE | 3 | PUTATIVE EXPORTED PROTEIN |
| HP0597 | 659 | 50.8 | 2XD5 | 21 | PENICILLIN-BINDING PROTEIN 1B |
| HP0598 | 373 | 56.1 | 1BS0 | 28 | PROTEIN (8-AMINO-7-OXONANOATE SYNTHASE) |
| HP0602 | 218 | 38.6 | 1PU6 | 96 | 3-METHYLADENINE DNA GLYCOSYLASE |
| HP0604 | 340 | 59.3 | 1R3S | 39 | UROPORPHYRINOGEN DECARBOXYLASE |
| HP0607 | 1028 | 43 | 4DNT | 21 | CATION EFFLUX SYSTEM PROTEIN |
| HP0613 | 233 | 30.2 | 2YYZ | 23 | SUGAR ABC TRANSPORTER, ATP-BINDING PROTEIN |
| HP0615 | 656 | 37.9 | 1V9P | 38 | DNA LIGASE |
| HP0616 | 313 | 21.4 | 3G19 | 24 | RESPONSE REGULATOR |
| HP0617 | 577 | 56.3 | 1IL2 | 50 | ASPARTYL-TRNA SYNTHETASE |
| HP0618 | 191 | 29.3 | 2BWJ | 33 | ADENYLATE KINASE 5 |
| HP0620 | 173 | 34.5 | 2BQX | 100 | INORGANIC PYROPHOSPHATASE |
| HP0623 | 449 | 56.2 | 1P3D | 36 | UDP-N-ACETYLMURAMATE--ALANINE LIGASE |
| HP0624 | 376 | 66.8 | [3EZS](http://ekhidna.biocenter.helsinki.fi/dali/daliquery?pdbid=3ezs&chainid=A) | 100 | AMINOTRANSFERASE ASPB |
| HP0625 | 359 | 54 | 3NOY | 45 | 4-HYDROXY-3-METHYLBUT-2-EN-1-YL DIPHOSPHATE SYNTHASE |
| HP0626 | 401 | 54.2 | 2RIJ | 50 | PUTATIVE 2,3,4,5-TETRAHYDROPYRIDINE-2-CARBOXYLATE |
| HP0628 | 225 | 11.6 | 1KLX | 26 | CYSTEINE RICH PROTEIN B |
| HP0630 | 194 | 33 | 2AMJ | 59 | MODULATOR OF DRUG ACTIVITY B |
| HP0631 | 384 | 42.7 | 1YQW | 50 | PERIPLASMIC [NIFE] HYDROGENASE SMALL SUBUNIT |
| HP0632 | 578 | 65.8 | 1UBO | 49 | PERIPLASMIC [NIFE] HYDROGENASE SMALL SUBUNIT |
| HP0639 | 226 | 32.6 | 3BL5 | 43 | QUEUOSINE BIOSYNTHESIS PROTEIN QUEC |
| HP0640 | 402 | 28.8 | 1VFG | 27 | POLY A POLYMERASE |
| HP0642 | 217 | 29.1 | 2H0U | 95 | NADPH-FLAVIN OXIDOREDUCTASE |
| HP0643 | 439 | 46.3 | 2O5R | 36 | GLUTAMYL-TRNA SYNTHETASE |
| HP0646 | 375 | 50.2 | [3JUK](http://ekhidna.biocenter.helsinki.fi/dali/daliquery?pdbid=3juk&chainid=A) | 100 | UDP-GLUCOSE PYROPHOSPHORYLASE (GALU) |
| HP0648 | 422 | 69 | 1EJD | 48 | UDP-N-ACETYLGLUCOSAMINE ENOLPYRUVYLTRANSFERASE |
| HP0649 | 468 | 63.5 | 1JSW | 56 | L-ASPARTATE AMMONIA-LYASE |
| HP0651 | 476 | 55.6 | 2NZW | 92 | ALPHA1,3-FUCOSYLTRANSFERASE |
| HP0652 | 207 | 38.6 | 3M1Y | 97 | PHOSPHOSERINE PHOSPHATASE |
| HP0653 | 167 | 30.3 | 3BVF | 98 | FERRITIN |
| HP0655 | 916 | 34.7 | 3EFC | 20 | OUTER MEMBRANE PROTEIN ASSEMBLY FACTOR YAET |
| HP0657 | 432 | 50.6 | 3EOQ | 16 | PUTATIVE ZINC PROTEASE |
| HP0658 | 475 | 52.9 | 3H0M | 51 | GLUTAMYL-TRNA(GLN) AMIDOTRANSFERASE SUBUNIT A |
| HP0661 | 143 | 26.2 | 1KVC | 52 | RIBONUCLEASE H |
| HP0662 | 240 | 34.2 | 1O0W | 34 | RIBONUCLEASE III |
| HP0663 | 365 | 59.1 | 1UM0 | 100 | CHORISMATE SYNTHASE |
| HP0665 | 457 | 63.5 | 1O1T | 44 | OXYGEN-INDEPENDENT COPROPORPHYRINOGEN III OXIDASE |
| HP0672 | 390 | 67.9 | 1J32 | 45 | ASPARTATE AMINOTRANSFERASE |
| HP0676 | 168 | 28.4 | 1WRJ | 32 | METHYLATED-DNA--PROTEIN-CYSTEINE |
| HP0679 | 289 | 44.4 | 3OA0 | 41 | LIPOPOLYSACCARIDE BIOSYNTHESIS PROTEIN WBPB |
| HP0680 | 788 | 61.9 | 3HNF | 36 | RIBONUCLEOSIDE-DIPHOSPHATE REDUCTASE LARGE SUBUNI |
| HP0683 | 433 | 44.9 | 3FWW | 43 | GLUCOSAMINE-1-PHOSPHATE ACETYLTRANSFERASE |
| HP0690 | 391 | 74.7 | 1WL4 | 46 | ACETYL-COENZYME A ACETYLTRANSFERASE 2 |
| HP0691 | 232 | 41.5 | 3CDK | 66 | SUCCINYL-COA:3-KETOACID-COENZYMEA TRANSFERASE |
| HP0692 | 207 | 35.3 | 3CDK | 72 | SUCCINYL-COA:3-KETOACID-COENZYME A TRANSFERASE |
| HP0701 | 827 | 45.1 | [1ZVU](http://ekhidna.biocenter.helsinki.fi/dali/daliquery?pdbid=1zvu&chainid=A) | 36 | TOPOISOMERASE IV SUBUNIT A |
| HP0703 | 381 | 46.2 | 1NY5 | 40 | TRANSCRIPTIONAL REGULATOR (NTRC FAMILY) |
| HP0705 | 935 | 60.5 | 2R6F | 51 | EXCINUCLEASE ABC SUBUNIT A |
| HP0707 | 308 | 46.4 | 1M6Y | 43 | S-ADENOSYL-METHYLTRANSFERASE MRAW |
| HP0709 | 300 | 30.7 | 2C5H | 27 | 5'-FLUORO-5'-DEOXYADENOSINE SYNTHASE |
| HP0715 | 240 | 27.7 | 1JI0 | 30 | ABC TRANSPORTER |
| HP0721 | 152 | 4.3 | 3QS8 | 5 | ANTHRANILATE PHOSPHORIBOSYLTRANSFERASE |
| HP0723 | 330 | 56.4 | 2W1T | 94 | L-ASPARAGINASE |
| HP0727 | 328 | 28.4 | 3B0U | 32 | TRNA-DIHYDROURIDINE SYNTHASE |
| HP0736 | 369 | 50.1 | 1IUG | 36 | ASPARTATE AMINOTRANSFERASE |
| HP0738 | 347 | 50.4 | 2PVP | 97 | D-ALANINE-D-ALANINE LIGASE |
| HP0739 | 241 | 36.8 | 2XUA | 11 | 3-OXOADIPATE ENOL-LACTONASE |
| HP0741 | 161 | 18.9 | 3LB5 | 23 | HIT-LIKE PROTEIN INVOLVED IN CELL-CYCLE REGULATION |
| HP0742 | 318 | 45 | 3DAH | 55 | RIBOSE-PHOSPHATE PYROPHOSPHOKINASE |
| HP0745 | 327 | 38.7 | 1V9F | 29 | RIBOSOMAL LARGE SUBUNIT PSEUDOURIDINE SYNTHASE D |
| HP0748 | 223 | 36.8 | 1L2T | 39 | HYPOTHETICAL ABC TRANSPORTER ATP-BINDING PROTEIN |
| HP0753 | 126 | 25.9 | [3IQC](http://ekhidna.biocenter.helsinki.fi/dali/daliquery?pdbid=3iqc&chainid=A) | 100 | FLAGELLAR PROTEIN |
| HP0757 | 292 | 35.8 | 3P8K | 23 | HYDROLASE, CARBON-NITROGEN FAMILY |
| HP0763 | 293 | 32.4 | 1OKK | 42 | SIGNAL RECOGNITION PARTICLE PROTEIN |
| HP0768 | 321 | 48.5 | 1TV8 | 38 | MOLYBDENUM COFACTOR BIOSYNTHESIS PROTEIN A |
| HP0769 | 201 | 23.7 | 1FRW | 20 | MOLYBDOPTERIN-GUANINE DINUCLEOTIDE BIOSYNTHESIS PROTEIN |
| HP0773 | 363 | 48.9 | 3BO9 | 31 | PUTATIVE NITROALKAN DIOXYGENASE |
| HP0774 | 402 | 52.8 | 1H3E | 42 | TYROSYL-TRNA SYNTHETASE |
| HP0777 | 240 | 42.7 | 3EK6 | 52 | URIDYLATE KINASE |
| HP0778 | 227 | 18.4 | 3A3U | 15 | MENAQUINONE BIOSYNTHETIC ENZYME |
| HP0779 | 853 | 66.2 | 1L5J | 68 | ACONITATE HYDRATASE 2 |
| HP0786 | 865 | 53.4 | 3JV2 | 49 | PROTEIN TRANSLOCASE SUBUNIT SECA |
| HP0789 | 48 | 24.8 | 3JQJ | 44 | MOLYBDENUM COFACTOR BIOSYNTHESIS PROTEIN C |
| HP0793 | 174 | 33 | 2EW5 | 98 | PEPTIDE DEFORMYLASE |
| HP0794 | 196 | 33.9 | 2ZL2 | 100 | ATP-DEPENDENT CLP PROTEASE PROTEOLYTIC SUBUNIT |
| HP0798 | 158 | 27.2 | 2EEY | 49 | MOLYBDOPTERIN BIOSYNTHESIS |
| HP0799 | 176 | 36.2 | 3K6A | 63 | MOLYBDENUM COFACTOR BIOSYNTHESIS PROTEIN MOG |
| HP0800 | 145 | 28.7 | [3RPF](http://ekhidna.biocenter.helsinki.fi/dali/daliquery?pdbid=3rpf&chainid=A) | 100 | MOLYBDOPTERIN SYNTHASE CATALYTIC SUBUNIT |
| HP0801 | 74 | 14.4 | 1NVI | 17 | MOLYBDOPTERIN CONVERTING FACTOR SUBUNIT 1 |
| HP0802 | 192 | 30.1 | [2BZ1](http://ekhidna.biocenter.helsinki.fi/dali/daliquery?pdbid=2bz1&chainid=A) | 47 | GTP CYCLOHYDROLASE II |
| HP0804 | 344 | 37.5 | [1G57](http://ekhidna.biocenter.helsinki.fi/dali/daliquery?pdbid=1g57&chainid=B) | 44 | 3,4-DIHYDROXY-2-BUTANONE 4-PHOSPHATE SYNTHASE |
| HP0806 | 206 | 57.2 | [1BY5](http://ekhidna.biocenter.helsinki.fi/dali/daliquery?pdbid=1by5&chainid=A) | 16 | FERRIC HYDROXAMATE UPTAKE PROTEIN |
| HP0808 | 119 | 23.2 | [3OTA](http://ekhidna.biocenter.helsinki.fi/dali/daliquery?pdbid=3ota&chainid=A) | 35 | HOLO-[ACYL-CARRIER-PROTEIN] SYNTHASE |
| HP0810 | 200 | 26.8 | [2ESR](http://ekhidna.biocenter.helsinki.fi/dali/daliquery?pdbid=2esr&chainid=A) | 31 | METHYLTRANSFERASE |
| HP0813 | 205 | 36.3 | [2ZWR](http://ekhidna.biocenter.helsinki.fi/dali/daliquery?pdbid=2zwr&chainid=A) | 32 | METALLO-BETA-LACTAMASE SUPERFAMILY PROTEIN |
| HP0814 | 255 | 41.2 | [1ZUD](http://ekhidna.biocenter.helsinki.fi/dali/daliquery?pdbid=1zud&chainid=1) | 35 | ADENYLYLTRANSFERASE THIF |
| HP0816 | 257 | 27.6 | 3CYP | 100 | CHEMOTAXIS PROTEIN MOTB |
| HP0817 | 148 | 7.8 | [1JL5](http://ekhidna.biocenter.helsinki.fi/dali/daliquery?pdbid=1jl5&chainid=A) | 17 | OUTER PROTEIN YOPM |
| HP0818 | 553 | 34.2 | 3O66 | 34 | GLYCINE BETAINE/CARNITINE/CHOLINE ABC TRANSPORTER |
| HP0819 | 216 | 38.4 | [2AWN](http://ekhidna.biocenter.helsinki.fi/dali/daliquery?pdbid=2awn&chainid=B) | 36 | MALTOSE/MALTODEXTRIN IMPORT ATP-BINDING PROTEIN M |
| HP0821 | 594 | 30.4 | [2NRZ](http://ekhidna.biocenter.helsinki.fi/dali/daliquery?pdbid=2nrz&chainid=B) | 34 | UVRABC SYSTEM PROTEIN C |
| HP0822 | 421 | 61.9 | [3MTJ](http://ekhidna.biocenter.helsinki.fi/dali/daliquery?pdbid=3mtj&chainid=A) | 45 | HOMOSERINE DEHYDROGENASE |
| HP0824 | 106 | 23.0 | [2PPT](http://ekhidna.biocenter.helsinki.fi/dali/daliquery?pdbid=2ppt&chainid=B) | 24 | THIOREDOXIN-2 |
| HP0825 | 311 | 52.2 | [2Q0L](http://ekhidna.biocenter.helsinki.fi/dali/daliquery?pdbid=2q0l&chainid=A) | 100 | THIOREDOXIN REDUCTASE |
| HP0827 | 82 | 14 | [3HI9](http://ekhidna.biocenter.helsinki.fi/dali/daliquery?pdbid=3hi9&chainid=D) | 32 | ELAV-LIKE PROTEIN 1 |
| HP0829 | 481 | 59.6 | [1VRD](http://ekhidna.biocenter.helsinki.fi/dali/daliquery?pdbid=1vrd&chainid=A) | 51 | INOSINE-5'-MONOPHOSPHATE DEHYDROGENASE |
| HP0830 | 453 | 70.1 | [3H0L](http://ekhidna.biocenter.helsinki.fi/dali/daliquery?pdbid=3h0l&chainid=A) | 50 | GLUTAMYL-TRNA(GLN) AMIDOTRANSFERASE SUBUNIT A |
| HP0831 | 196 | 26.1 | [2GRJ](http://ekhidna.biocenter.helsinki.fi/dali/daliquery?pdbid=2grj&chainid=A) | 40 | DEPHOSPHO-COA KINASE |
| HP0832 | 262 | 47.7 | [2CMG](http://ekhidna.biocenter.helsinki.fi/dali/daliquery?pdbid=2cmg&chainid=A) | 100 | SPERMIDINE SYNTHASE |
| HP0834 | 458 | 50.5 | [1MKY](http://ekhidna.biocenter.helsinki.fi/dali/daliquery?pdbid=1mky&chainid=A) | 33 | PROBABLE GTP-BINDING PROTEIN ENGA |
| HP0840 | 333 | 58.5 | [2GN4](http://ekhidna.biocenter.helsinki.fi/dali/daliquery?pdbid=2gn4&chainid=A) | 100 | UDP-GLCNAC C6 DEHYDRATASE |
| HP0843 | 219 | 39.0 | [1G4T](http://ekhidna.biocenter.helsinki.fi/dali/daliquery?pdbid=1g4t&chainid=A) | 38 | THIAMIN PHOSPHATE SYNTHASE |
| HP0844 | 270 | 46.1 | [1UB0](http://ekhidna.biocenter.helsinki.fi/dali/daliquery?pdbid=1ub0&chainid=A) | 47 | PHOSPHOMETHYLPYRIMIDINE KINASE |
| HP0845 | 273 | 42.4 | [1V8A](http://ekhidna.biocenter.helsinki.fi/dali/daliquery?pdbid=1v8a&chainid=A) | 37 | HYDROXYETHYLTHIAZOLE KINASE |
| HP0846 | 866 | 46.3 | [2Y3T](http://ekhidna.biocenter.helsinki.fi/dali/daliquery?pdbid=2y3t&chainid=C) | 50 | TYPE I RESTRICTION ENZYME ECOR124II R PROTEIN |
| HP0850 | 527 | 59.0 | [3LKD](http://ekhidna.biocenter.helsinki.fi/dali/daliquery?pdbid=3lkd&chainid=A) | 38 | TYPE I RESTRICTION-MODIFICATION SYSTEM |
| HP0853 | 533 | 14.6 | 2YZ2 | 21 | PUTATIVE ABC TRANSPORTER ATP-BINDING PROTEIN TM_0 |
| HP0854 | 527 | 49.3 | 1YPF | 79 | GMP REDUCTASE |
| HP0857 | 192 | 36.4 | [1TK9](http://ekhidna.biocenter.helsinki.fi/dali/daliquery?pdbid=1tk9&chainid=A) | 59 | PHOSPHOHEPTOSE ISOMERASE 1 |
| HP0858 | 461 | 52.2 | 3CQD | 20 | 6-PHOSPHOFRUCTOKINASE ISOZYME 2 |
| HP0859 | 330 | 55.3 | [3SXP](http://ekhidna.biocenter.helsinki.fi/dali/daliquery?pdbid=3sxp&chainid=D) | 94 | ADP-L-GLYCERO-D-MANNOHEPTOSE-6-EPIMERASE |
| HP0860 | 173 | 32.2 | [2GMW](http://ekhidna.biocenter.helsinki.fi/dali/daliquery?pdbid=2gmw&chainid=A) | 39 | D,D-HEPTOSE 1,7-BISPHOSPHATE PHOSPHATASE |
| HP0862 | 223 | 28.1 | [3DJC](http://ekhidna.biocenter.helsinki.fi/dali/daliquery?pdbid=3djc&chainid=A) | 25 | TYPE III PANTOTHENATE KINASE |
| HP0865 | 145 | 21.6 | [3MDX](http://ekhidna.biocenter.helsinki.fi/dali/daliquery?pdbid=3mdx&chainid=A) | 42 | DEOXYURIDINE 5'-TRIPHOSPHATE NUCLEOTIDOHYDROLASE |
| HP0866 | 164 | 23.2 | [2P4V](http://ekhidna.biocenter.helsinki.fi/dali/daliquery?pdbid=2p4v&chainid=A) | 32 | TRANSCRIPTION ELONGATION FACTOR GREB |
| HP0867 | 360 | 47.2 | [1F0K](http://ekhidna.biocenter.helsinki.fi/dali/daliquery?pdbid=1f0k&chainid=A) | 17 | UDP-N-ACETYLGLUCOSAMINE-N-ACETYLMURAMYL- |
| HP0871 | 244 | 42.5 | 2POF | 75 | CDP-DIACYLGLYCEROL PYROPHOSPHATASE |
| HP0872 | 109 | 16.3 | [2AKK](http://ekhidna.biocenter.helsinki.fi/dali/daliquery?pdbid=2akk&chainid=A) | 47 | PHNA-LIKE PROTEIN |
| HP0875 | 505 | 66.7 | [1QWL](http://ekhidna.biocenter.helsinki.fi/dali/daliquery?pdbid=1qwl&chainid=A) | 100 | KATA CATALASE |
| HP0877 | 157 | 30.8 | [1HJR](http://ekhidna.biocenter.helsinki.fi/dali/daliquery?pdbid=1hjr&chainid=A) | 33 | HOLLIDAY JUNCTION RESOLVASE (RUVC) |
| HP0883 | 183 | 23.6 | [1CUK](http://ekhidna.biocenter.helsinki.fi/dali/daliquery?pdbid=1cuk&chainid=A) | 38 | RUVA PROTEIN |
| HP0886 | 465 | 63.3 | [1U0B](http://ekhidna.biocenter.helsinki.fi/dali/daliquery?pdbid=1u0b&chainid=B) | 38 | CYSTEINYL-TRNA SYNTHETASE |
| HP0887 | 1290 | 58.3 | [2QV3](http://ekhidna.biocenter.helsinki.fi/dali/daliquery?pdbid=2qv3&chainid=A) | 96 | VACUOLATING CYTOTOXIN |
| HP0888 | 255 | 31.8 | 1OXV | 36 | ABC TRANSPORTER, ATP BINDING PROTEIN |
| HP0890 | 256 | 42.9 | [1ZEM](http://ekhidna.biocenter.helsinki.fi/dali/daliquery?pdbid=1zem&chainid=A) | 23 | XYLITOL DEHYDROGENASE |
| HP0891 | 174 | 29.6 | [1VPM](http://ekhidna.biocenter.helsinki.fi/dali/daliquery?pdbid=1vpm&chainid=A) | 35 | ACYL-COA HYDROLASE |
| HP0898 | 370 | 60.6 | 2Z1D | 36 | HYDROGENASE EXPRESSION/FORMATION PROTEIN HYPD |
| HP0900 | 242 | 39.5 | [2HF9](http://ekhidna.biocenter.helsinki.fi/dali/daliquery?pdbid=2hf9&chainid=A) | 33 | PROBABLE HYDROGENASE NICKEL INCORPORATION |
| HP0910 | 379 | 48.5 | [1AQJ](http://ekhidna.biocenter.helsinki.fi/dali/daliquery?pdbid=1aqj&chainid=B) | 20 | ADENINE-N6-DNA-METHYLTRANSFERASE TAQI |
| HP0911 | 675 | 52.4 | 2IS6 | 25 | DNA HELICASE II |
| HP0919 | 1085 | 60.2 | [1JDB](http://ekhidna.biocenter.helsinki.fi/dali/daliquery?pdbid=1jdb&chainid=B) | 53 | CARBAMOYL PHOSPHATE SYNTHETASE |
| HP0921 | 332 | 52.5 | [1VC2](http://ekhidna.biocenter.helsinki.fi/dali/daliquery?pdbid=1vc2&chainid=A) | 44 | GLYCERALDEHYDE 3-PHOSPHATE DEHYDROGENASE |
| HP0924 | 68 | 13.0 | [3M21](http://ekhidna.biocenter.helsinki.fi/dali/daliquery?pdbid=3m21&chainid=A) | 100 | PROBABLE TAUTOMERASE HP_0924 |
| HP0926 | 381 | 48.5 | [1AQJ](http://ekhidna.biocenter.helsinki.fi/dali/daliquery?pdbid=1aqj&chainid=B) | 20 | ADENINE-N6-DNA-METHYLTRANSFERASE TAQI |
| HP0928 | 180 | 29.7 | [1WUR](http://ekhidna.biocenter.helsinki.fi/dali/daliquery?pdbid=1wur&chainid=A) | 46 | GTP CYCLOHYDROLASE I |
| HP0929 | 303 | 50.4 | [3LLW](http://ekhidna.biocenter.helsinki.fi/dali/daliquery?pdbid=3llw&chainid=A) | 100 | GERANYLTRANSTRANSFERASE (ISPA) |
| HP0930 | 267 | 29.6 | 1ILV | 37 | STATIONARY-PHASE SURVIVAL PROTEIN SURE HOMOLOG |
| HP0933 | 200 | 20.9 | [2G64](http://ekhidna.biocenter.helsinki.fi/dali/daliquery?pdbid=2g64&chainid=A) | 21 | PUTATIVE 6-PYRUVOYL TETRAHYDROBIOPTERIN SYNTHASE |
| HP0935 | 161 | 19.8 | 3FIX | 20 | N-ACETYLTRANSFERASE |
| HP0940 | 256 | 37.3 | [2IEE](http://ekhidna.biocenter.helsinki.fi/dali/daliquery?pdbid=2iee&chainid=A) | 33 | PROBABLE ABC TRANSPORTER EXTRACELLULAR-BINDING |
| HP0941 | 377 | 60.1 | [1VFT](http://ekhidna.biocenter.helsinki.fi/dali/daliquery?pdbid=1vft&chainid=A) | 27 | ALANINE RACEMASE |
| HP0943 | 410 | 58.4 | [1NG3](http://ekhidna.biocenter.helsinki.fi/dali/daliquery?pdbid=1ng3&chainid=B) | 18 | GLYCINE OXIDASE |
| HP0944 | 125 | 23.9 | 1QD9 | 48 | PURINE REGULATORY PROTEIN YABJ |
| HP0950 | 289 | 44.7 | [2F9Y](http://ekhidna.biocenter.helsinki.fi/dali/daliquery?pdbid=2f9y&chainid=B) | 49 | ACETYL-COA CARBOXYLASE, CARBOXYLTRANSFERASE ALPHA |
| HP0952 | 218 | 34.6 | [2A9S](http://ekhidna.biocenter.helsinki.fi/dali/daliquery?pdbid=2a9s&chainid=A) | 42 | COMPETENCE/DAMAGE-INDUCIBLE PROTEIN CINA |
| HP0954 | 210 | 30.0 | [3BEM](http://ekhidna.biocenter.helsinki.fi/dali/daliquery?pdbid=3bem&chainid=A) | 20 | PUTATIVE NAD(P)H NITROREDUCTASE YDFN |
| HP0956 | 242 | 23.2 | 1V9K | 29 | LARGE SUBUNIT PSEUDOURIDINE SYNTHASE C |
| HP0959 | 243 | 29.3 | 2GX8 | 25 | NIF3-RELATED PROTEIN |
| HP0960 | 303 | 44.9 | [1J5W](http://ekhidna.biocenter.helsinki.fi/dali/daliquery?pdbid=1j5w&chainid=A) | 59 | GLYCYL-TRNA SYNTHETASE ALPHA CHAIN |
| HP0961 | 312 | 49.2 | [1EVY](http://ekhidna.biocenter.helsinki.fi/dali/daliquery?pdbid=1evy&chainid=A) | 30 | GLYCEROL-3-PHOSPHATE DEHYDROGENASE |
| HP0962 | 153 | 18.3 | [2QNW](http://ekhidna.biocenter.helsinki.fi/dali/daliquery?pdbid=2qnw&chainid=A) | 53 | ACYL CARRIER PROTEIN |
| HP0969 | 1020 | 45.2 | 2V50 | 20 | MULTIDRUG RESISTANCE PROTEIN MEXB |
| HP0974 | 491 | 65.5 | [1EQJ](http://ekhidna.biocenter.helsinki.fi/dali/daliquery?pdbid=1eqj&chainid=A) | 43 | PHOSPHOGLYCERATE MUTASE |
| HP0976 | 436 | 65.1 | [3DU4](http://ekhidna.biocenter.helsinki.fi/dali/daliquery?pdbid=3du4&chainid=A) | 35 | ADENOSYLMETHIONINE-8-AMINO-7-OXONONANOATE AMINOTR |
| HP0978 | 492 | 56.9 | [1E4F](http://ekhidna.biocenter.helsinki.fi/dali/daliquery?pdbid=1e4f&chainid=T) | 20 | CELL DIVISION PROTEIN FTSA |
| HP0979 | 385 | 56.8 | [2VXY](http://ekhidna.biocenter.helsinki.fi/dali/daliquery?pdbid=2vxy&chainid=A) | 46 | CELL DIVISION PROTEIN FTSZ |
| HP0995 | 355 | 37.4 | [1A0P](http://ekhidna.biocenter.helsinki.fi/dali/daliquery?pdbid=1a0p&chainid=A) | 21 | SITE-SPECIFIC RECOMBINASE XERD |
| HP1008 | 138 | 22.7 | [2EC2](http://ekhidna.biocenter.helsinki.fi/dali/daliquery?pdbid=2ec2&chainid=A) | 33 | 136AA LONG HYPOTHETICAL TRANSPOSASE |
| HP1010 | 675 | 54.4 | [1XDP](http://ekhidna.biocenter.helsinki.fi/dali/daliquery?pdbid=1xdp&chainid=A) | 35 | POLYPHOSPHATE KINASE |
| HP1011 | 351 | 61.8 | [1D3G](http://ekhidna.biocenter.helsinki.fi/dali/daliquery?pdbid=1d3g&chainid=A) | 34 | DIHYDROOROTATE DEHYDROGENASE |
| HP1012 | 444 | 62.4 | 3AMI | 31 | ZINC PEPTIDASE |
| HP1013 | 300 | 53.2 | [3M5V](http://ekhidna.biocenter.helsinki.fi/dali/daliquery?pdbid=3m5v&chainid=A) | 56 | DIHYDRODIPICOLINATE SYNTHASE |
| HP1014 | 262 | 35.6 | 1FMC | 32 | 7 ALPHA-HYDROXYSTEROID DEHYDROGENASE |
| HP1019 | 443 | 55.1 | [1KY9](http://ekhidna.biocenter.helsinki.fi/dali/daliquery?pdbid=1ky9&chainid=B) | 40 | PROTEASE DOMAIN |
| HP1020 | 406 | 52.2 | [1W55](http://ekhidna.biocenter.helsinki.fi/dali/daliquery?pdbid=1w55&chainid=A) | 40 | ISPD/ISPF BIFUNCTIONAL ENZYME |
| HP1024 | 288 | 21.7 | [1C3G](http://ekhidna.biocenter.helsinki.fi/dali/daliquery?pdbid=1c3g&chainid=A) | 24 | HEAT SHOCK PROTEIN 40 |
| HP1026 | 391 | 37.3 | [1SXJ](http://ekhidna.biocenter.helsinki.fi/dali/daliquery?pdbid=1sxj&chainid=C) | 21 | ACTIVATOR 1 95 KDA SUBUNIT |
| HP1027 | 150 | 25.8 | [2XIG](http://ekhidna.biocenter.helsinki.fi/dali/daliquery?pdbid=2xig&chainid=A) | 100 | FERRIC UPTAKE REGULATION PROTEIN |
| HP1030 | 287 | 31.3 | [2HP7](http://ekhidna.biocenter.helsinki.fi/dali/daliquery?pdbid=2hp7&chainid=A) | 12 | FLAGELLAR MOTOR SWITCH PROTEIN FLIM |
| HP1031 | 354 | 30.9 | [2HP7](http://ekhidna.biocenter.helsinki.fi/dali/daliquery?pdbid=2hp7&chainid=A) | 30 | FLAGELLAR MOTOR SWITCH PROTEIN FLIM |
| HP1032 | 255 | 34.6 | 1RP3 | 31 | RNA POLYMERASE SIGMA FACTOR SIGMA-28 (FLIA) |
| HP1034 | 294 | 41.6 | [1HYQ](http://ekhidna.biocenter.helsinki.fi/dali/daliquery?pdbid=1hyq&chainid=A) | 30 | CELL DIVISION INHIBITOR (MIND-1) FAMILY OF ATPASES |
| HP1035 | 459 | 43.6 | 2PX0 | 33 | FLAGELLAR BIOSYNTHESIS PROTEIN FLHF |
| HP1036 | 163 | 25.7 | [2QX0](http://ekhidna.biocenter.helsinki.fi/dali/daliquery?pdbid=2qx0&chainid=A) | 33 | 7,8-DIHYDRO-6-HYDROXYMETHYLPTERIN |
| HP1037 | 357 | 38.3 | 3Q6D | 32 | PROLINE DIPEPTIDASE |
| HP1038 | 167 | 32.9 | [2C4W](http://ekhidna.biocenter.helsinki.fi/dali/daliquery?pdbid=2c4w&chainid=A) | 100 | 3-DEHYDROQUINATE DEHYDRATASE |
| HP1040 | 90 | 18.0 | [2VQE](http://ekhidna.biocenter.helsinki.fi/dali/daliquery?pdbid=2vqe&chainid=O) | 55 | 16S RRNA |
| HP1041 | 733 | 53.5 | [3MYD](http://ekhidna.biocenter.helsinki.fi/dali/daliquery?pdbid=3myd&chainid=A) | 100 | FLAGELLAR BIOSYNTHESIS PROTEIN FLHA |
| HP1043 | 223 | 20.9 | 2HQO | 96 | TRANSCRIPTIONAL REGULATOR |
| HP1044 | 370 | 27.8 | 3IB8 | 16 | CYCLIC AMP PHOSPHODIESTERASE THAT MOONLIGHTS AS A MODIFIER OF CELL WALL PERMEABILITY |
| HP1045 | 662 | 68.7 | [2P2F](http://ekhidna.biocenter.helsinki.fi/dali/daliquery?pdbid=2p2f&chainid=B) | 46 | ACETYL-COENZYME A SYNTHETASE |
| HP1046 | 146 | 7 | 1KQ1 | 9 | HOST FACTOR FOR Q BETA |
| HP1050 | 293 | 47.8 | 1FWL | 26 | HOMOSERINE KINASE |
| HP1052 | 295 | 51.5 | [2VES](http://ekhidna.biocenter.helsinki.fi/dali/daliquery?pdbid=2ves&chainid=A) | 44 | UDP-3-O-[3-HYDROXYMYRISTOYL] N-ACETYLGLUCOSAMINE |
| HP1058 | 270 | 49.7 | [1M3U](http://ekhidna.biocenter.helsinki.fi/dali/daliquery?pdbid=1m3u&chainid=A) | 40 | 3-METHYL-2-OXOBUTANOATE HYDROXYMETHYLTRANSFERASE |
| HP1059 | 336 | 43.5 | [1IN4](http://ekhidna.biocenter.helsinki.fi/dali/daliquery?pdbid=1in4&chainid=A) | 51 | HOLLIDAY JUNCTION DNA HELICASE RUVB |
| HP1062 | 345 | 41.6 | [1VKY](http://ekhidna.biocenter.helsinki.fi/dali/daliquery?pdbid=1vky&chainid=A) | 36 | S-ADENOSYLMETHIONINE:TRNA RIBOSYLTRANSFERASE-ISOM |
| HP1063 | 178 | 29.1 | [1JSX](http://ekhidna.biocenter.helsinki.fi/dali/daliquery?pdbid=1jsx&chainid=A) | 31 | GLUCOSE-INHIBITED DIVISION PROTEIN B |
| HP1067 | 124 | 29.1 | [3H1G](http://ekhidna.biocenter.helsinki.fi/dali/daliquery?pdbid=3h1g&chainid=A) | 100 | CHEMOTAXIS PROTEIN CHEY HOMOLOG |
| HP1069 | 632 | 39.3 | [2R62](http://ekhidna.biocenter.helsinki.fi/dali/daliquery?pdbid=2r62&chainid=A) | 100 | CELL DIVISION PROTEASE FTSH HOMOLOG |
| HP1073 | 66 | 17.5 | [1YG0](http://ekhidna.biocenter.helsinki.fi/dali/daliquery?pdbid=1yg0&chainid=A) | 100 | COPPER ION BINDING PROTEIN |
| HP1076 | 171 | 24.5 | [3K1H](http://ekhidna.biocenter.helsinki.fi/dali/daliquery?pdbid=3k1h&chainid=A) | 100 | PUTATIVE UNCHARACTERIZED PROTEIN |
| HP1084 | 307 | 47.0 | [1ML4](http://ekhidna.biocenter.helsinki.fi/dali/daliquery?pdbid=1ml4&chainid=A) | 36 | ASPARTATE TRANSCARBAMOYLASE |
| HP1086 | 235 | 36 | 3HP7 | 35 | HEMOLYSIN, PUTATIVE |
| HP1088 | 641 | 60.5 | [3L84](http://ekhidna.biocenter.helsinki.fi/dali/daliquery?pdbid=3l84&chainid=A) | 57 | TRANSKETOLASE |
| HP1090 | 858 | 67.6 | [2IUT](http://ekhidna.biocenter.helsinki.fi/dali/daliquery?pdbid=2iut&chainid=B) | 49 | DNA TRANSLOCASE FTSK |
| HP1092 | 269 | 17.9 | [1WLG](http://ekhidna.biocenter.helsinki.fi/dali/daliquery?pdbid=1wlg&chainid=A) | 21 | FLAGELLAR HOOK PROTEIN FLGE |
| HP1098 | 290 | 15.8 | 1KLX | 36 | CYSTEINE RICH PROTEIN B |
| HP1099 | 208 | 41.4 | [1WBH](http://ekhidna.biocenter.helsinki.fi/dali/daliquery?pdbid=1wbh&chainid=A) | 48 | KHG/KDPG ALDOLASE |
| HP1100 | 608 | 54.3 | [2GP4](http://ekhidna.biocenter.helsinki.fi/dali/daliquery?pdbid=2gp4&chainid=A) | 53 | 6-PHOSPHOGLUCONATE DEHYDRATASE |
| HP1101 | 425 | 54.2 | [1DPG](http://ekhidna.biocenter.helsinki.fi/dali/daliquery?pdbid=1dpg&chainid=A) | 31 | GLUCOSE 6-PHOSPHATE DEHYDROGENASE |
| HP1102 | 227 | 41.2 | [3LHI](http://ekhidna.biocenter.helsinki.fi/dali/daliquery?pdbid=3lhi&chainid=A) | 37 | PUTATIVE 6-PHOSPHOGLUCONOLACTONASE |
| HP1103 | 336 | 52.0 | [1SZ2](http://ekhidna.biocenter.helsinki.fi/dali/daliquery?pdbid=1sz2&chainid=A) | 41 | GLUCOKINASE |
| HP1104 | 348 | 46.8 | 3TWO | 94 | MANNITOL DEHYDROGENASE |
| HP1108 | 186 | 20.5 | 3ON3 | 25 | KETO/OXOACID FERREDOXIN OXIDOREDUCTASE, GAMMA SUBUNIT |
| HP1110 | 407 | 65 | 2C3P | 28 | PYRUVATE-FERREDOXIN OXIDOREDUCTASE |
| HP1111 | 314 | 45.6 | 2C3Y | 26 | PYRUVATE-FERREDOXIN OXIDOREDUCTASE |
| HP1112 | 440 | 52.2 | [2PFM](http://ekhidna.biocenter.helsinki.fi/dali/daliquery?pdbid=2pfm&chainid=A) | 50 | ADENYLOSUCCINATE LYASE |
| HP1114 | 658 | 61.3 | [1D9X](http://ekhidna.biocenter.helsinki.fi/dali/daliquery?pdbid=1d9x&chainid=A) | 57 | EXCINUCLEASE UVRABC COMPONENT UVRB |
| HP1117 | 256 | 15.5 | 1KLX | 29 | CYSTEINE RICH PROTEIN B |
| HP1118 | 567 | 50.2 | [3FNM](http://ekhidna.biocenter.helsinki.fi/dali/daliquery?pdbid=3fnm&chainid=A) | 100 | GAMMA-GLUTAMYLTRANSPEPTIDASE (GGT) LARGE SUBUNIT |
| HP1121 | 312 | 48.6 | [2C7Q](http://ekhidna.biocenter.helsinki.fi/dali/daliquery?pdbid=2c7q&chainid=A) | 32 | DNA METHYLTRANSFERASE |
| HP1123 | 185 | 14.4 | [2K8I](http://ekhidna.biocenter.helsinki.fi/dali/daliquery?pdbid=2k8i&chainid=A) | 30 | PEPTIDYL-PROLYL CIS-TRANS ISOMERASE |
| HP1124 | 331 | 35.4 | 2FI7 | 21 | TYPE 4 FIMBRIAL BIOGENESIS PROTEIN PILF |
| HP1125 | 179 | 21.6 | 1OAP | 36 | PEPTIDOGLYCAN-ASSOCIATED LIPOPROTEIN |
| HP1126 | 417 | 61.9 | [2IVZ](http://ekhidna.biocenter.helsinki.fi/dali/daliquery?pdbid=2ivz&chainid=D) | 16 | PROTEIN TOLB |
| HP1127 | 193 | 16.1 | [1LR0](http://ekhidna.biocenter.helsinki.fi/dali/daliquery?pdbid=1lr0&chainid=A) | 16 | TOLA PROTEIN |
| HP1131 | 124 | 16.9 | 1FS0 | 28 | ATP SYNTHASE EPSILON SUBUNIT |
| HP1132 | 469 | 65.9 | [2HLD](http://ekhidna.biocenter.helsinki.fi/dali/daliquery?pdbid=2hld&chainid=D) | 69 | ATP SYNTHASE ALPHA CHAIN, MITOCHONDRIAL |
| HP1133 | 301 | 34.7 | [1FS0](http://ekhidna.biocenter.helsinki.fi/dali/daliquery?pdbid=1fs0&chainid=G) | 31 | ATP SYNTHASE EPSILON SUBUNIT |
| HP1134 | 503 | 69.9 | [2QE7](http://ekhidna.biocenter.helsinki.fi/dali/daliquery?pdbid=2qe7&chainid=A) | 66 | ATP SYNTHASE SUBUNIT ALPHA |
| HP1138 | 290 | 23.8 | [1VZ0](http://ekhidna.biocenter.helsinki.fi/dali/daliquery?pdbid=1vz0&chainid=A) | 48 | CHROMOSOME PARTITIONING PROTEIN PARB |
| HP1139 | 264 | 44.2 | [1WCV](http://ekhidna.biocenter.helsinki.fi/dali/daliquery?pdbid=1wcv&chainid=1) | 41 | SEGREGATION PROTEIN |
| HP1140 | 212 | 28.0 | [1HXD](http://ekhidna.biocenter.helsinki.fi/dali/daliquery?pdbid=1hxd&chainid=A) | 33 | BIRA BIFUNCTIONAL PROTEIN |
| HP1141 | 303 | 42.9 | [1FMT](http://ekhidna.biocenter.helsinki.fi/dali/daliquery?pdbid=1fmt&chainid=A) | 37 | METHIONYL-TRNA FMET FORMYLTRANSFERASE |
| HP1148 | 229 | 31.5 | [1P9P](http://ekhidna.biocenter.helsinki.fi/dali/daliquery?pdbid=1p9p&chainid=A) | 34 | TRNA (GUANINE-N(1)-)-METHYLTRANSFERASE |
| HP1151 | 76 | 16.8 | [2VQE](http://ekhidna.biocenter.helsinki.fi/dali/daliquery?pdbid=2vqe&chainid=P) | 49 | 16S RRNA |
| HP1152 | 448 | 38.3 | 2J45 | 41 | SIGNAL RECOGNITION PARTICLE PROTEIN |
| HP1153 | 874 | 52.8 | [1GAX](http://ekhidna.biocenter.helsinki.fi/dali/daliquery?pdbid=1gax&chainid=B) | 42 | VALYL-TRNA SYNTHETASE |
| HP1155 | 353 | 55.4 | [1F0K](http://ekhidna.biocenter.helsinki.fi/dali/daliquery?pdbid=1f0k&chainid=A) | 25 | UDP-N-ACETYLGLUCOSAMINE-N-ACETYLMURAMYL- |
| HP1159 | 177 | 37.6 | [2F6S](http://ekhidna.biocenter.helsinki.fi/dali/daliquery?pdbid=2f6s&chainid=A) | 100 | CELL FILAMENTATION PROTEIN, PUTATIVE |
| HP1160 | 140 | 6.8 | 3MDJ | 12 | ENDOPLASMIC RETICULUM AMINOPEPTIDASE 1 |
| HP1161 | 164 | 37.3 | [2BMV](http://ekhidna.biocenter.helsinki.fi/dali/daliquery?pdbid=2bmv&chainid=A) | 100 | FLAVODOXIN |
| HP1164 | 324 | 46.9 | [2ZBW](http://ekhidna.biocenter.helsinki.fi/dali/daliquery?pdbid=2zbw&chainid=A) | 21 | THIOREDOXIN REDUCTASE |
| HP1166 | 545 | 63.9 | [1HM5](http://ekhidna.biocenter.helsinki.fi/dali/daliquery?pdbid=1hm5&chainid=A) | 52 | PHOSPHOGLUCOSE ISOMERASE |
| HP1171 | 248 | 42.2 | [2OLJ](http://ekhidna.biocenter.helsinki.fi/dali/daliquery?pdbid=2olj&chainid=A) | 52 | AMINO ACID ABC TRANSPORTER |
| HP1172 | 277 | 43.8 | [1XT8](http://ekhidna.biocenter.helsinki.fi/dali/daliquery?pdbid=1xt8&chainid=A) | 60 | PUTATIVE AMINO-ACID TRANSPORTER PERIPLASMIC SOLUT |
| HP1178 | 233 | 43.6 | [1PK9](http://ekhidna.biocenter.helsinki.fi/dali/daliquery?pdbid=1pk9&chainid=A) | 55 | PURINE NUCLEOSIDE PHOSPHORYLASE |
| HP1179 | 413 | 65.2 | [3M7V](http://ekhidna.biocenter.helsinki.fi/dali/daliquery?pdbid=3m7v&chainid=A) | 37 | PHOSPHOPENTOMUTASE |
| HP1182 | 253 | 36.2 | [2E89](http://ekhidna.biocenter.helsinki.fi/dali/daliquery?pdbid=2e89&chainid=A) | 18 | TRNA(ILE)-LYSIDINE SYNTHASE |
| HP1186 | 202 | 25.9 | [1KOP](http://ekhidna.biocenter.helsinki.fi/dali/daliquery?pdbid=1kop&chainid=A) | 32 | CARBONIC ANHYDRASE |
| HP1189 | 346 | 55.9 | [2GZ1](http://ekhidna.biocenter.helsinki.fi/dali/daliquery?pdbid=2gz1&chainid=A) | 47 | ASPARTATE BETA-SEMIALDEHYDE DEHYDROGENASE |
| HP1190 | 442 | 53.4 | [1WU7](http://ekhidna.biocenter.helsinki.fi/dali/daliquery?pdbid=1wu7&chainid=B) | 26 | HISTIDYL-TRNA SYNTHETASE |
| HP1193 | 329 | 35.3 | 3N2T | 30 | PUTATIVE OXIDOREDUCTASE |
| HP1196 | 155 | 28.4 | [2QBB](http://ekhidna.biocenter.helsinki.fi/dali/daliquery?pdbid=2qbb&chainid=G) | 60 | 16S RRNA |
| HP1199 | 125 | 19.8 | [1DD3](http://ekhidna.biocenter.helsinki.fi/dali/daliquery?pdbid=1dd3&chainid=A) | 63 | 50S RIBOSOMAL PROTEIN L7/L12 |
| HP1202 | 141 | 4.2 | [2KLM](http://ekhidna.biocenter.helsinki.fi/dali/daliquery?pdbid=2klm&chainid=A) | 34 | 50S RIBOSOMAL PROTEIN L11 |
| HP1203 | 176 | 20.4 | [1M1G](http://ekhidna.biocenter.helsinki.fi/dali/daliquery?pdbid=1m1g&chainid=A) | 40 | TRANSCRIPTION ANTITERMINATION PROTEIN |
| HP1205 | 399 | 48.1 | [1EFU](http://ekhidna.biocenter.helsinki.fi/dali/daliquery?pdbid=1efu&chainid=A) | 75 | ELONGATION FACTOR TU |
| HP1207 | 222 | 36.6 | [2HSZ](http://ekhidna.biocenter.helsinki.fi/dali/daliquery?pdbid=2hsz&chainid=A) | 14 | NOVEL PREDICTED PHOSPHATASE |
| HP1209 | 172 | 5.6 | [2QGP](http://ekhidna.biocenter.helsinki.fi/dali/daliquery?pdbid=2qgp&chainid=C) | 20 | HNH ENDONUCLEASE |
| HP1210 | 171 | 32.1 | [1SSQ](http://ekhidna.biocenter.helsinki.fi/dali/daliquery?pdbid=1ssq&chainid=A) | 43 | SERINE ACETYLTRANSFERASE |
| HP1213 | 688 | 63.4 | [3GCM](http://ekhidna.biocenter.helsinki.fi/dali/daliquery?pdbid=3gcm&chainid=C) | 37 | POLYRIBONUCLEOTIDE NUCLEOTIDYLTRANSFERASE |
| HP1214 | 240 | 13.3 | 3G6W | 17 | URACIL PHOSPHORIBOSYLTRANSFERASE |
| HP1218 | 424 | 67.2 | [2YW2](http://ekhidna.biocenter.helsinki.fi/dali/daliquery?pdbid=2yw2&chainid=A) | 34 | PHOSPHORIBOSYLAMINE--GLYCINE LIGASE |
| HP1221 | 234 | 38.8 | [2D2R](http://ekhidna.biocenter.helsinki.fi/dali/daliquery?pdbid=2d2r&chainid=A) | 100 | UNDECAPRENYL PYROPHOSPHATE SYNTHASE |
| HP1222 | 948 | 24.6 | 1F0X | 11 | D-LACTATE DEHYDROGENASE |
| HP1224 | 226 | 35.2 | [3P9Z](http://ekhidna.biocenter.helsinki.fi/dali/daliquery?pdbid=3p9z&chainid=A) | 100 | UROPORPHYRINOGEN III COSYNTHASE (HEMD) |
| HP1226 | 352 | 52.7 | [1OLT](http://ekhidna.biocenter.helsinki.fi/dali/daliquery?pdbid=1olt&chainid=A) | 21 | OXYGEN-INDEPENDENT COPROPORPHYRINOGEN III OXIDASE |
| HP1227 | 96 | 16.3 | [1DVH](http://ekhidna.biocenter.helsinki.fi/dali/daliquery?pdbid=1dvh&chainid=A) | 36 | CYTOCHROME C553 |
| HP1228 | 155 | 26.7 | [1F3Y](http://ekhidna.biocenter.helsinki.fi/dali/daliquery?pdbid=1f3y&chainid=A) | 40 | DIADENOSINE 5',5'''-P1,P4-TETRAPHOSPHATE |
| HP1229 | 405 | 42.7 | [3L76](http://ekhidna.biocenter.helsinki.fi/dali/daliquery?pdbid=3l76&chainid=A) | 46 | ASPARTOKINASE |
| HP1230 | 180 | 32.3 | [2UVP](http://ekhidna.biocenter.helsinki.fi/dali/daliquery?pdbid=2uvp&chainid=A) | 100 | HOBA |
| HP1232 | 380 | 46.3 | [1AJ0](http://ekhidna.biocenter.helsinki.fi/dali/daliquery?pdbid=1aj0&chainid=A) | 31 | DIHYDROPTEROATE SYNTHASE |
| HP1237 | 375 | 65.3 | [1A9X](http://ekhidna.biocenter.helsinki.fi/dali/daliquery?pdbid=1a9x&chainid=B) | 38 | CARBAMOYL PHOSPHATE SYNTHETASE (LARGE CHAIN) |
| HP1238 | 334 | 57.1 | [2DYU](http://ekhidna.biocenter.helsinki.fi/dali/daliquery?pdbid=2dyu&chainid=A) | 100 | FORMAMIDASE |
| HP1240 | 190 | 27.8 | [1EX2](http://ekhidna.biocenter.helsinki.fi/dali/daliquery?pdbid=1ex2&chainid=A) | 21 | PROTEIN MAF |
| HP1241 | 847 | 60.4 | [1YFS](http://ekhidna.biocenter.helsinki.fi/dali/daliquery?pdbid=1yfs&chainid=B) | 49 | ALANYL-TRNA SYNTHETASE |
| HP1245 | 179 | 21.2 | [2VW9](http://ekhidna.biocenter.helsinki.fi/dali/daliquery?pdbid=2vw9&chainid=A) | 100 | SINGLE-STRANDED DNA BINDING PROTEIN |
| HP1248 | 644 | 54.0 | [2WP8](http://ekhidna.biocenter.helsinki.fi/dali/daliquery?pdbid=2wp8&chainid=J) | 25 | EXOSOME COMPLEX COMPONENT RRP45 |
| HP1249 | 263 | 41.1 | [2HK9](http://ekhidna.biocenter.helsinki.fi/dali/daliquery?pdbid=2hk9&chainid=A) | 33 | SHIKIMATE DEHYDROGENASE |
| HP1252 | 594 | 59.4 | [1XOC](http://ekhidna.biocenter.helsinki.fi/dali/daliquery?pdbid=1xoc&chainid=A) | 21 | OLIGOPEPTIDE-BINDING PROTEIN APPA |
| HP1253 | 339 | 53.4 | [1I6K](http://ekhidna.biocenter.helsinki.fi/dali/daliquery?pdbid=1i6k&chainid=A) | 52 | TRYPTOPHANYL-TRNA SYNTHETASE |
| HP1256 | 185 | 27.3 | [1IS1](http://ekhidna.biocenter.helsinki.fi/dali/daliquery?pdbid=1is1&chainid=A) | 45 | RIBOSOME RECYCLING FACTOR |
| HP1257 | 201 | 30.6 | [2YZK](http://ekhidna.biocenter.helsinki.fi/dali/daliquery?pdbid=2yzk&chainid=D) | 21 | OROTATE PHOSPHORIBOSYLTRANSFERASE |
| HP1259 | 205 | 29.0 | [1M2K](http://ekhidna.biocenter.helsinki.fi/dali/daliquery?pdbid=1m2k&chainid=A) | 35 | SILENT INFORMATION REGULATOR 2 |
| HP1261 | 159 | 23.4 | [3I9V](http://ekhidna.biocenter.helsinki.fi/dali/daliquery?pdbid=3i9v&chainid=6) | 61 | NADH-QUINONE OXIDOREDUCTASE SUBUNIT 1 |
| HP1263 | 409 | 63.5 | [3I9V](http://ekhidna.biocenter.helsinki.fi/dali/daliquery?pdbid=3i9v&chainid=4) | 42 | NADH-QUINONE OXIDOREDUCTASE SUBUNIT 1 |
| HP1268 | 220 | 18.9 | [2FUG](http://ekhidna.biocenter.helsinki.fi/dali/daliquery?pdbid=2fug&chainid=G) | 37 | NADH-QUINONE OXIDOREDUCTASE CHAIN 1 |
| HP1275 | 459 | 65.4 | [1P5D](http://ekhidna.biocenter.helsinki.fi/dali/daliquery?pdbid=1p5d&chainid=X) | 40 | PHOSPHOMANNOMUTASE |
| HP1277 | 262 | 44.6 | [2CLE](http://ekhidna.biocenter.helsinki.fi/dali/daliquery?pdbid=2cle&chainid=A) | 49 | TRYPTOPHAN SYNTHASE ALPHA CHAIN |
| HP1278 | 393 | 61.8 | 1V8Z | 60 | TRYPTOPHAN SYNTHASE BETA CHAIN 1 |
| HP1279 | 452 | 62.0 | [1PII](http://ekhidna.biocenter.helsinki.fi/dali/daliquery?pdbid=1pii&chainid=A) | 47 | N-(5'PHOSPHORIBOSYL)ANTHRANILATE ISOMERASE |
| HP1280 | 335 | 62.3 | [1V8G](http://ekhidna.biocenter.helsinki.fi/dali/daliquery?pdbid=1v8g&chainid=B) | 34 | ANTHRANILATE PHOSPHORIBOSYLTRANSFERASE |
| HP1281 | 194 | 38.4 | 1I7Q | 40 | ANTHRANILATE SYNTHASE |
| HP1282 | 500 | 62.3 | [1I1Q](http://ekhidna.biocenter.helsinki.fi/dali/daliquery?pdbid=1i1q&chainid=A) | 47 | ANTHRANILATE SYNTHASE COMPONENT I |
| HP1284 | 346 | 44.6 | [1PSW](http://ekhidna.biocenter.helsinki.fi/dali/daliquery?pdbid=1psw&chainid=A) | 18 | ADP-HEPTOSE LPS HEPTOSYLTRANSFERASE II |
| HP1285 | 230 | 36.7 | [3ET4](http://ekhidna.biocenter.helsinki.fi/dali/daliquery?pdbid=3et4&chainid=A) | 37 | OUTER MEMBRANE PROTEIN P4, NADP PHOSPHATASE |
| HP1286 | 182 | 31.0 | [3HPE](http://ekhidna.biocenter.helsinki.fi/dali/daliquery?pdbid=3hpe&chainid=A) | 90 | CONSERVED HYPOTHETICAL SECRETED PROTEIN |
| HP1287 | 217 | 41.6 | [2RD3](http://ekhidna.biocenter.helsinki.fi/dali/daliquery?pdbid=2rd3&chainid=A) | 100 | TRANSCRIPTIONAL REGULATOR |
| HP1291 | 204 | 19.4 | 3LM8 | 24 | THIAMINE PYROPHOSPHOKINASE |
| HP1292 | 116 | 22.1 | [3FIN](http://ekhidna.biocenter.helsinki.fi/dali/daliquery?pdbid=3fin&chainid=R) | 47 | 50S RIBOSOMAL PROTEIN L27 |
| HP1293 | 344 | 33.1 | [1ZYR](http://ekhidna.biocenter.helsinki.fi/dali/daliquery?pdbid=1zyr&chainid=B) | 31 | DNA-DIRECTED RNA POLYMERASE ALPHA CHAIN |
| HP1294 | 208 | 35.2 | [3I1M](http://ekhidna.biocenter.helsinki.fi/dali/daliquery?pdbid=3i1m&chainid=D) | 50 | 30S RIBOSOMAL PROTEIN S2 |
| HP1295 | 131 | 22.8 | [3I1M](http://ekhidna.biocenter.helsinki.fi/dali/daliquery?pdbid=3i1m&chainid=K) | 53 | 30S RIBOSOMAL PROTEIN S2 |
| HP1298 | 72 | 17.9 | [1AH9](http://ekhidna.biocenter.helsinki.fi/dali/daliquery?pdbid=1ah9&chainid=A) | 61 | INITIATION FACTOR 1 |
| HP1299 | 253 | 45.5 | [2GG2](http://ekhidna.biocenter.helsinki.fi/dali/daliquery?pdbid=2gg2&chainid=A) | 39 | METHIONINE AMINOPEPTIDASE |
| HP1303 | 119 | 23.0 | [3I1N](http://ekhidna.biocenter.helsinki.fi/dali/daliquery?pdbid=3i1n&chainid=O) | 31 | 50S RIBOSOMAL PROTEIN L2 |
| HP1304 | 178 | 25.5 | [1RL6](http://ekhidna.biocenter.helsinki.fi/dali/daliquery?pdbid=1rl6&chainid=A) | 41 | PROTEIN (RIBOSOMAL PROTEIN L6 |
| HP1305 | 131 | 27.4 | [1I94](http://ekhidna.biocenter.helsinki.fi/dali/daliquery?pdbid=1i94&chainid=H) | 39 | 16S RRNA |
| HP1307 | 181 | 31.6 | [1IQ4](http://ekhidna.biocenter.helsinki.fi/dali/daliquery?pdbid=1iq4&chainid=A) | 53 | 50S RIBOSOMAL PROTEIN L5 |
| HP1309 | 122 | 23 | 3I1N | 31 | 50S RIBOSOMAL PROTEIN L2 |
| HP1312 | 141 | 25.0 | [3I1N](http://ekhidna.biocenter.helsinki.fi/dali/daliquery?pdbid=3i1n&chainid=M) | 62 | 50S RIBOSOMAL PROTEIN L2 |
| HP1313 | 234 | 33.9 | [3I1M](http://ekhidna.biocenter.helsinki.fi/dali/daliquery?pdbid=3i1m&chainid=C) | 60 | 30S RIBOSOMAL PROTEIN S2 |
| HP1314 | 122 | 19.9 | [3I1N](http://ekhidna.biocenter.helsinki.fi/dali/daliquery?pdbid=3i1n&chainid=S) | 40 | 50S RIBOSOMAL PROTEIN L2 |
| HP1316 | 276 | 37.2 | [2AW4](http://ekhidna.biocenter.helsinki.fi/dali/daliquery?pdbid=2aw4&chainid=C) | 50 | 5S RIBOSOMAL RNA |
| HP1323 | 209 | 28.5 | [2ETJ](http://ekhidna.biocenter.helsinki.fi/dali/daliquery?pdbid=2etj&chainid=A) | 34 | RIBONUCLEASE HII |
| HP1325 | 463 | 57 | 1FUR | 63 | FUMARASE C |
| HP1332 | 369 | 15.7 | 3AGZ | 23 | DNAJ HOMOLOG SUBFAMILY |
| HP1335 | 360 | 48.8 | [2HMA](http://ekhidna.biocenter.helsinki.fi/dali/daliquery?pdbid=2hma&chainid=A) | 37 | PROBABLE TRNA (5-METHYLAMINOMETHYL-2-THIOURIDYLAT |
| HP1337 | 174 | 26.9 | [1KAM](http://ekhidna.biocenter.helsinki.fi/dali/daliquery?pdbid=1kam&chainid=A) | 24 | NICOTINATE-NUCLEOTIDE ADENYLYLTRANSFERASE |
| HP1338 | 148 | 22.3 | [2CA9](http://ekhidna.biocenter.helsinki.fi/dali/daliquery?pdbid=2ca9&chainid=A) | 100 | PUTATIVE NICKEL-RESPONSIVE REGULATOR |
| HP1345 | 402 | 63.9 | [1PHP](http://ekhidna.biocenter.helsinki.fi/dali/daliquery?pdbid=1php&chainid=A) | 47 | 3-PHOSPHOGLYCERATE KINASE |
| HP1346 | 330 | 56.9 | [3CMC](http://ekhidna.biocenter.helsinki.fi/dali/daliquery?pdbid=3cmc&chainid=O) | 46 | GLYCERALDEHYDE-3-PHOSPHATE DEHYDROGENASE |
| HP1347 | 233 | 38.7 | [1OKB](http://ekhidna.biocenter.helsinki.fi/dali/daliquery?pdbid=1okb&chainid=A) | 39 | URACIL-DNA GLYCOSYLASE |
| HP1350 | 459 | 53.1 | [1FC6](http://ekhidna.biocenter.helsinki.fi/dali/daliquery?pdbid=1fc6&chainid=A) | 27 | PHOTOSYSTEM II D1 C-TERMINAL PROCESSING PROTEASE |
| HP1353 | 315 | 31.7 | [1M6Z](http://ekhidna.biocenter.helsinki.fi/dali/daliquery?pdbid=1m6z&chainid=D) | 19 | CYTOCHROME C4 |
| HP1355 | 273 | 31.1 | 1QPR | 31 | QUINOLINIC ACID PHOSPHORIBOSYLTRANSFERASE |
| HP1356 | 336 | 41.3 | [1WZU](http://ekhidna.biocenter.helsinki.fi/dali/daliquery?pdbid=1wzu&chainid=A) | 34 | QUINOLINATE SYNTHETASE A |
| HP1357 | 267 | 26.9 | [2GPR](http://ekhidna.biocenter.helsinki.fi/dali/daliquery?pdbid=2gpr&chainid=A) | 19 | GLUCOSE-PERMEASE IIA COMPONENT |
| HP1362 | 488 | 25.4 | [3GXV](http://ekhidna.biocenter.helsinki.fi/dali/daliquery?pdbid=3gxv&chainid=A) | 100 | REPLICATIVE DNA HELICASE |
| HP1363 | 466 | 64.8 | [3K5W](http://ekhidna.biocenter.helsinki.fi/dali/daliquery?pdbid=3k5w&chainid=A) | 100 | CARBOHYDRATE KINASE |
| HP1365 | 213 | 27.1 | [3F6P](http://ekhidna.biocenter.helsinki.fi/dali/daliquery?pdbid=3f6p&chainid=A) | 32 | TRANSCRIPTIONAL REGULATORY PROTEIN YYCF |
| HP1367 | 260 | 36.8 | [1G60](http://ekhidna.biocenter.helsinki.fi/dali/daliquery?pdbid=1g60&chainid=A) | 59 | ADENINE-SPECIFIC METHYLTRANSFERASE MBOIIA |
| HP1373 | 347 | 48.3 | [1JCE](http://ekhidna.biocenter.helsinki.fi/dali/daliquery?pdbid=1pw4&chainid=A) | 17 | ROD SHAPE-DETERMINING PROTEIN MREB |
| HP1374 | 446 | 48.6 | [2GW1](http://ekhidna.biocenter.helsinki.fi/dali/daliquery?pdbid=2gw1&chainid=B) | 13 | MITOCHONDRIAL PRECURSOR PROTEINS IMPORT RECEPTOR |
| HP1375 | 270 | 65.4 | [1P5D](http://ekhidna.biocenter.helsinki.fi/dali/daliquery?pdbid=1p5d&chainid=X) | 40 | PHOSPHOMANNOMUTASE |
| HP1376 | 159 | 33.1 | 2GLL | 100 | (3R)-HYDROXYMYRISTOYL-(ACYL CARRIER PROTEIN) DEHYDRATASE |
| HP1377 | 146 | 3.4 | 2QMI | 9 | PBP RELATED BETA-LACTAMASE |
| HP1386 | 217 | 41.2 | [3INP](http://ekhidna.biocenter.helsinki.fi/dali/daliquery?pdbid=3inp&chainid=A) | 42 | D-RIBULOSE-PHOSPHATE 3-EPIMERASE |
| HP1393 | 524 | 36.8 | [1W1W](http://ekhidna.biocenter.helsinki.fi/dali/daliquery?pdbid=1w1w&chainid=A) | 15 | STRUCTURAL MAINTENANCE OF CHROMOSOME 1 |
| HP1394 | 284 | 28.1 | 1U0R | 27 | INORGANIC POLYPHOSPHATE/ATP-NAD KINASE |
| HP1398 | 380 | 51.7 | [2EEZ](http://ekhidna.biocenter.helsinki.fi/dali/daliquery?pdbid=2eez&chainid=A) | 43 | ALANINE DEHYDROGENASE |
| HP1399 | 322 | 50.1 | 3E9B | 22 | ARGINASE-1 |
| HP1400 | 842 | 63.6 | [2GRX](http://ekhidna.biocenter.helsinki.fi/dali/daliquery?pdbid=2grx&chainid=B) | 16 | FERRICHROME-IRON RECEPTOR |
| HP1406 | 282 | 46.0 | [1R30](http://ekhidna.biocenter.helsinki.fi/dali/daliquery?pdbid=1r30&chainid=A) | 30 | BIOTIN SYNTHASE |
| HP1418 | 259 | 38.6 | [1HSK](http://ekhidna.biocenter.helsinki.fi/dali/daliquery?pdbid=1hsk&chainid=A) | 26 | UDP-N-ACETYLENOLPYRUVOYLGLUCOSAMINE REDUCTASE |
| HP1420 | 434 | 62.5 | [2DPY](http://ekhidna.biocenter.helsinki.fi/dali/daliquery?pdbid=2dpy&chainid=A) | 44 | FLAGELLUM-SPECIFIC ATP SYNTHASE |
| HP1421 | 304 | 43.9 | [2GZA](http://ekhidna.biocenter.helsinki.fi/dali/daliquery?pdbid=2gza&chainid=B) | 25 | TYPE IV SECRETION SYSTEM PROTEIN VIRB11 |
| HP1422 | 920 | 51.2 | [1QU3](http://ekhidna.biocenter.helsinki.fi/dali/daliquery?pdbid=1qu3&chainid=A) | 39 | ISOLEUCYL-TRNA SYNTHETASE |
| HP1426 | 180 | 4.1 | [1J1T](http://ekhidna.biocenter.helsinki.fi/dali/daliquery?pdbid=1j1t&chainid=A) | 17 | ALGINATE LYASE |
| HP1429 | 329 | 32.5 | 3ETN | 36 | PHOSPHOSUGAR ISOMERASE INVOLVED IN CAPSULE FORMATION |
| HP1430 | 689 | 69.4 | [3BK2](http://ekhidna.biocenter.helsinki.fi/dali/daliquery?pdbid=3bk2&chainid=A) | 39 | METAL DEPENDENT HYDROLASE |
| HP1431 | 271 | 35.7 | [3FUT](http://ekhidna.biocenter.helsinki.fi/dali/daliquery?pdbid=3fut&chainid=A) | 34 | DIMETHYLADENOSINE TRANSFERASE |
| HP1433 | 856 | 30.3 | [2ADM](http://ekhidna.biocenter.helsinki.fi/dali/daliquery?pdbid=2adm&chainid=B) | 13 | ADENINE-N6-DNA-METHYLTRANSFERASE TAQI |
| HP1439 | 81 | 5.9 | 1NYH | 25 | REGULATORY PROTEIN SIR4 |
| HP1441 | 163 | 28.3 | [1W74](http://ekhidna.biocenter.helsinki.fi/dali/daliquery?pdbid=1w74&chainid=A) | 45 | PEPTIDYL-PROLYL CIS-TRANS ISOMERASE A |
| HP1444 | 152 | 9.3 | 1WJX | 43 | SSRA-BINDING PROTEIN |
| HP1448 | 161 | 27.1 | [1D6T](http://ekhidna.biocenter.helsinki.fi/dali/daliquery?pdbid=1d6t&chainid=A) | 26 | RIBONUCLEASE P |
| HP1450 | 547 | 34.6 | [3BLC](http://ekhidna.biocenter.helsinki.fi/dali/daliquery?pdbid=3blc&chainid=B) | 15 | INNER MEMBRANE PROTEIN OXAA |
| HP1457 | 210 | 15.5 | [2HQS](http://ekhidna.biocenter.helsinki.fi/dali/daliquery?pdbid=2hqs&chainid=A) | 14 | PROTEIN TOLB |
| HP1458 | 104 | 23.0 | [3HHV](http://ekhidna.biocenter.helsinki.fi/dali/daliquery?pdbid=3hhv&chainid=A) | 38 | THIOREDOXIN |
| HP1459 | 262 | 12.8 | 2APO | 17 | PROBABLE TRNA PSEUDOURIDINE SYNTHASE B |
| HP1460 | 1211 | 44.9 | 2HPI | 45 | DNA POLYMERASE III ALPHA SUBUNIT |
| HP1461 | 350 | 47.5 | [3HQ6](http://ekhidna.biocenter.helsinki.fi/dali/daliquery?pdbid=3hq6&chainid=A) | 50 | CYTOCHROME C551 PEROXIDASE |
| HP1465 | 261 | 39.3 | [2OLJ](http://ekhidna.biocenter.helsinki.fi/dali/daliquery?pdbid=2olj&chainid=A) | 33 | AMINO ACID ABC TRANSPORTER |
| HP1468 | 340 | 54.1 | [3DTG](http://ekhidna.biocenter.helsinki.fi/dali/daliquery?pdbid=3dtg&chainid=A) | 40 | BRANCHED-CHAIN AMINO ACID AMINOTRANSFERASE |
| HP1470 | 892 | 59.4 | [1TAQ](http://ekhidna.biocenter.helsinki.fi/dali/daliquery?pdbid=1taq&chainid=A) | 35 | TAQ DNA POLYMERASE |
| HP1476 | 187 | 35.0 | [1SBZ](http://ekhidna.biocenter.helsinki.fi/dali/daliquery?pdbid=1sbz&chainid=D) | 33 | PROBABLE AROMATIC ACID DECARBOXYLASE |
| HP1477 | 218 | 12.1 | [1XUU](http://ekhidna.biocenter.helsinki.fi/dali/daliquery?pdbid=1xuu&chainid=A) | 14 | POLYSIALIC ACID CAPSULE BIOSYNTHESIS PROTEIN |
| HP1478 | 682 | 54.7 | [1PJR](http://ekhidna.biocenter.helsinki.fi/dali/daliquery?pdbid=1pjr&chainid=A) | 38 | DNA HELICASE |
| HP1479 | 844 | 47.8 | [1W3B](http://ekhidna.biocenter.helsinki.fi/dali/daliquery?pdbid=1w3b&chainid=A) | 12 | UDP-N-ACETYLGLUCOSAMINE-PEPTIDE N-ACETYLGLUCOSAMINYLTRANSFERASE |
| HP1480 | 415 | 58.4 | [2DQ3](http://ekhidna.biocenter.helsinki.fi/dali/daliquery?pdbid=2dq3&chainid=A) | 48 | SERYL-TRNA SYNTHETASE |
| HP1481 | 265 | 41.9 | [2E11](http://ekhidna.biocenter.helsinki.fi/dali/daliquery?pdbid=2e11&chainid=C) | 16 | HYDROLASE |
| HP1485 | 190 | 8.9 | 3CB4 | 5 | GTP-BINDING PROTEIN LEPA |
| HP1494 | 447 | 48.6 | [2XJA](http://ekhidna.biocenter.helsinki.fi/dali/daliquery?pdbid=2xja&chainid=C) | 34 | UDP-N-ACETYLMURAMOYLALANYL-D-GLUTAMATE--2,6-DIAMINOPIMELATE LIGASE |
| HP1495 | 316 | 53.5 | [3CLM](http://ekhidna.biocenter.helsinki.fi/dali/daliquery?pdbid=3clm&chainid=A) | 32 | TRANSALDOLASE |
| HP1496 | 178 | 24.5 | [1FEU](http://ekhidna.biocenter.helsinki.fi/dali/daliquery?pdbid=1feu&chainid=A) | 22 | 50S RIBOSOMAL PROTEIN L25 |
| HP1497 | 186 | 35.0 | [2PTH](http://ekhidna.biocenter.helsinki.fi/dali/daliquery?pdbid=2pth&chainid=A) | 38 | PEPTIDYL-TRNA HYDROLASE |
| HP1499 | 272 | 29.1 | [1BYR](http://ekhidna.biocenter.helsinki.fi/dali/daliquery?pdbid=1byr&chainid=A) | 15 | PROTEIN (ENDONUCLEASE) |
| HP1503 | 788 | 28.9 | [1MHS](http://ekhidna.biocenter.helsinki.fi/dali/daliquery?pdbid=1mhs&chainid=A) | 17 | PLASMA MEMBRANE ATPASE |
| HP1504 | 238 | 22.2 | 2B3T | 25 | PUTATIVE METHYLTRANSFERASE |
| HP1508 | 458 | 17.8 | [2R39](http://ekhidna.biocenter.helsinki.fi/dali/daliquery?pdbid=2r39&chainid=A) | 21 | FIXG-RELATED PROTEIN |
| HP1513 | 386 | 55.1 | [1T3I](http://ekhidna.biocenter.helsinki.fi/dali/daliquery?pdbid=1t3i&chainid=A) | 14 | PROBABLE CYSTEINE DESULFURASE |
| HP1526 | 250 | 45.3 | [2O3C](http://ekhidna.biocenter.helsinki.fi/dali/daliquery?pdbid=2o3c&chainid=A) | 52 | APEX NUCLEASE 1 |
| HP1529 | 457 | 32.3 | [2WP0](http://ekhidna.biocenter.helsinki.fi/dali/daliquery?pdbid=2wp0&chainid=A) | 100 | HOBA(DNAA BINDING PROTEIN, RECENTLY IDENTIFIED AS AN ESSENTIAL REGULATOR OF DNA REPLICATION) |
| HP1531 | 79 | 7.9 | 1LRZ | 10 | FACTOR ESSENTIAL FOR EXPRESSION OF METHICILLIN |
| HP1532 | 597 | 61.4 | [1JXA](http://ekhidna.biocenter.helsinki.fi/dali/daliquery?pdbid=1jxa&chainid=A) | 38 | GLUCOSAMINE 6-PHOSPHATE SYNTHASE |
| HP1533 | 231 | 34.3 | [3AH5](http://ekhidna.biocenter.helsinki.fi/dali/daliquery?pdbid=3ah5&chainid=A) | 100 | THYMIDYLATE SYNTHASE THYX |
| HP1538 | 285 | 31.7 | [1M6Z](http://ekhidna.biocenter.helsinki.fi/dali/daliquery?pdbid=1m6z&chainid=D) | 19 | CYTOCHROME C4 |
| HP1541 | 999 | 43.3 | [2EYQ](http://ekhidna.biocenter.helsinki.fi/dali/daliquery?pdbid=2eyq&chainid=A) | 30 | TRANSCRIPTION-REPAIR COUPLING FACTOR |
| HP1547 | 806 | 53.4 | [2BTE](http://ekhidna.biocenter.helsinki.fi/dali/daliquery?pdbid=2bte&chainid=A) | 46 | AMINOACYL-TRNA SYNTHETASE |
| HP1554 | 264 | 36.9 | [3I1M](http://ekhidna.biocenter.helsinki.fi/dali/daliquery?pdbid=3i1m&chainid=B) | 49 | 30S RIBOSOMAL PROTEIN S2 |
| HP1555 | 355 | 30.8 | 3MMP | 42 | ELONGATION FACTOR TU 2, ELONGATION FACTOR TS |
| HP1556 | 615 | 40.6 | 3OCN | 26 | PENICILLIN-BINDING PROTEIN 3 |
| HP1562 | 333 | 36.2 | [2ETV](http://ekhidna.biocenter.helsinki.fi/dali/daliquery?pdbid=2etv&chainid=A) | 17 | IRON(III) ABC TRANSPORTER, PERIPLASMIC IRON-BINDING |
| HP1563 | 198 | 34.8 | [1ZOF](http://ekhidna.biocenter.helsinki.fi/dali/daliquery?pdbid=1zof&chainid=G) | 98 | ALKYL HYDROPEROXIDE-REDUCTASE |
| HP1564 | 271 | 40.6 | [1XS5](http://ekhidna.biocenter.helsinki.fi/dali/daliquery?pdbid=1xs5&chainid=A) | 37 | MEMBRANE LIPOPROTEIN TPN32 |
| HP1565 | 588 | 51.2 | 3EQU | 23 | PENICILLIN-BINDING PROTEIN 2 |
| HP1567 | 208 | 34.1 | 1SVI | 26 | GTP-BINDING PROTEIN YSXC |
| HP1570 | 164 | 28.2 | 2R8Z | 43 | 3-DEOXY-D-MANNO-OCTULOSONATE 8-PHOSPHATE PHOSPHATASE |
| HP1571 | 315 | 15.1 | [1X60](http://ekhidna.biocenter.helsinki.fi/dali/daliquery?pdbid=1x60&chainid=A) | 30 | SPORULATION-SPECIFIC N-ACETYLMURAMOYL-L-ALANINE |
| HP1573 | 254 | 45.5 | [2GZX](http://ekhidna.biocenter.helsinki.fi/dali/daliquery?pdbid=2gzx&chainid=A) | 38 | PUTATIVE TATD RELATED DNASE |
| HP1574 | 206 | 13.5 | 1PKV | 32 | RIBOFLAVIN SYNTHASE ALPHA CHAIN |
| HP1576 | 327 | 45.0 | [3DHW](http://ekhidna.biocenter.helsinki.fi/dali/daliquery?pdbid=3dhw&chainid=C) | 47 | D-METHIONINE TRANSPORT SYSTEM PERMEASE PROTEIN |
| HP1582 | 262 | 41.8 | [1M5W](http://ekhidna.biocenter.helsinki.fi/dali/daliquery?pdbid=1m5w&chainid=A) | 40 | PYRIDOXAL PHOSPHATE BIOSYNTHETIC PROTEIN PDXJ |
| HP1583 | 307 | 36.6 | [2HI1](http://ekhidna.biocenter.helsinki.fi/dali/daliquery?pdbid=2hi1&chainid=A) | 32 | 4-HYDROXYTHREONINE-4-PHOSPHATE DEHYDROGENASE 2 |
| HP1584 | 340 | 47.7 | [3EN9](http://ekhidna.biocenter.helsinki.fi/dali/daliquery?pdbid=3en9&chainid=B) | 26 | O-SIALOGLYCOPROTEIN ENDOPEPTIDASE/PROTEIN KINASE |
