# Supplementary material for: Structure Based Annotation of Helicobacter pylori Strain 26695 Proteome
Source: PLoS One. 2014 Dec 30;9(12):e115020. doi: 10.1371/journal.pone.0115020 (PMC4280198; doi:10.1371/journal.pone.0115020)
Supplement: S5 Table — Distribution of various SCOP-ID, class, fold, superfamily and family across whole proteome. (DOC) [file pone.0115020.s005.doc]

| **Supplementary Table V. Stuctural folds of *H. pylori* 26695 strain proteins that have confident structure assignment.** | | | | | | |
| --- | --- | --- | --- | --- | --- | --- |
| **Gene name** | **SCOP-ID** | **Fold** | **SuperFamily** | **Family** | **Class** | **Function analysis** |
| HP0001 | a.79.1.1 | NusB-like[48012](1 superfamily) 6 helices | NusB-like | antitermination factor NusB | All alpha proteins | DNA-dependent transcription, termination |
| HP0002 | c.16.1.1 | Lumazine synthase | Lumazine synthase | Lumazine synthase | Alpha and beta proteins (a/b) | [GO:0000906  "6,7-dimethyl-8-ribityllumazine synthase activity" evidence=ISS]  [GO:0009231 "riboflavin biosynthetic process" evidence=ISS] |
| HP0003 | c.1.10.4 | Tim beta/alpha barrel | aldolase | Class1 DAHP synthetase | Alpha and beta proteins (a/b) | [GO:0008676  "3-deoxy-8-phosphooctulonate synthase activity" evidence=ISS]  [GO:0019294 "keto-3-deoxy-D-manno-octulosonic acid biosynthetic  process" evidence=ISS] |
| HP0004 | c.53.2.1 | Resolvase like | Beta-carbonic anyhydrase.cab | Beta-carbonic anyhydrase.cab | Alpha and beta proteins (a/b) | [GO:0004089 "carbonate dehydratase  activity" evidence=ISS] [GO:0008152 "metabolic process"  evidence=ISS] |
| HP0005 | c.1.2.3 | Tim beta/alpha barrel | Ribulose phosphate binding barrel | Decarboxylase | Alpha and beta proteins (a/b) | [GO:0004590  "orotidine-5'-phosphate decarboxylase activity" evidence=ISS]  [GO:0009220 "pyrimidine ribonucleotide biosynthetic process"  evidence=ISS] |
| HP0006 | c.26.1.4 | Adenine nucleotide alpha hydrolase | Nucleotidylyl transferase | Pantothenate synthetase | Alpha and beta proteins (a/b) | [GO:0004592  "pantoate-beta-alanine ligase activity" evidence=ISS] [GO:0015940  "pantothenate biosynthetic process" evidence=ISS] |
| HP0009 | f.4.1.1 | TRANSMEMBRANE BETA BAREELS | OMPA LIKE | OUTERMEMBRANE PROTEINS | Membrane and cell surface proteins and peptides |  |
| HP0010 | c.25.1.1 | Ferrodoxin reductase like cterminal NADP linked domain | Ferrodoxin reductase like cterminal NADP linked domain | reductases | Alpha and beta proteins (a/b) | [GO:0006457 "protein folding"  evidence=ISS] [GO:0016887 "ATPase activity" evidence=ISS] |
| HP0011 | b.35.1.1 | GroES-like | GroES like | GroES | All beta proteins | [GO:0006457 "protein folding"  evidence=ISS] [GO:0051087 "chaperone binding" evidence=ISS] |
| HP0012 | e.13.1.1 | DNA primase core | DNA primase core | DNA primase DnaG catalytic core | Multi-domain proteins (alpha and beta) | [GO:0003896 "DNA primase activity"  evidence=ISS] [GO:0006269 "DNA replication, synthesis of RNA  primer" evidence=ISS] |
| HP0013 | c.26.2.5 | Adenine nucleotide alpha hydrolase like | Adenine nucleotide alpha hydrolase like | PP-loop ATPase | Alpha and beta proteins (a/b) | [GO:0003674  "molecular_function" evidence=ND] [GO:0005575 "cellular_component"  evidence=ND] [GO:0008150 "biological_process" evidence=ND] |
| HP0014 | d.177.1.1 | FAH | FAH | FAH | Alpha and beta proteins (a+b) |  |
| HP0015 | d.316.1.1 | MK0786-like | MK0786-like | MK0786-like | Alpha and beta proteins (a+b) | [GO:0004190 "aspartic-type  endopeptidase activity" evidence=IEA] |
| HP0016 | j.80.1.1 | Syndecan-4 cytoplasmic domain | Syndecan-4 cytoplasmic domain | Syndecan-4 cytoplasmic domain | peptides |  |
| HP0017 | c.37.1.1 | P-loop containing nucleoside triphosphate hydrolases | P-loop containing nucleoside triphosphate hydrolases | RecA protein like | Alpha and beta proteins (a/b) | [GO:0003674  "molecular_function" evidence=ND] |
| HP0018 | a.118.8.1 | alpha-alpha superhelix | TPR-like | Tetratricopeptide repeat (TPR) | All alpha proteins | [GO:0003674 "molecular_function"  evidence=ND] [GO:0008150 "biological_process" evidence=ND] |
| HP0019 | c.23.1.1 | Flavodoxin-like | CheY-like | CheY-related | Alpha and beta proteins (a/b) | [GO:0004871 "signal transducer activity" evidence=ISS] [GO:0006935  "chemotaxis" evidence=ISS] |
| HP0020 | b.49.2.3 | Domain of alpha and beta subunits of F1 ATP synthase-like | Alanine racemase C-terminal domain-like | Eukaryotic ODC-like | All beta proteins | [GO:0008295 "spermidine biosynthetic process"  evidence=IMP;IDA] [GO:0016831 "carboxy-lyase activity"  evidence=IDA] |
| HP0021 | a.111.1.1 | Acid phosphatase/Vanadium-dependent haloperoxidase | Acid phosphatase/Vanadium-dependent haloperoxidase | Type 2 phosphatidic acid phosphatase, PAP2 | All alpha proteins | [GO:0003674  "molecular_function" evidence=ND] [GO:0008150 "biological_process"  evidence=ND] |
| HP0022 | c.76.1.2 | Alkaline phosphatase-like | Alkaline phosphatase-like | Arylsulfatase | Alpha and beta proteins (a/b) | [GO:0003824 "catalytic activity"  evidence=ISS] [GO:0008152 "metabolic process" evidence=ISS] |
| HP0023 | g.4.1.1 | PMP inhibitors | PMP inhibitors | PMP inhibitors | All small proteins |  |
| HP0024 | a.208.1.1 | DhaL-like | DhaL-like | DhaL-like | All alpha proteins |  |
| HP0025 | f.4.1.1 | TRANSMEMBRANE BETA BAREELS | OMPA LIKE | OUTERMEMBRANE PROTEINS | Membrane and cell surface proteins and peptides |  |
| HP0026 | a.103.1.1 | Citrate synthase | Citrate synthase | Citrate synthase | All alpha proteins | [GO:0004108 "citrate (Si)-synthase  activity" evidence=ISS] [GO:0006099 "tricarboxylic acid cycle"  evidence=ISS] |
| HP0027 | c.77.1.1 | Isocitrate/Isopropylmalate dehydrogenase-like | Isocitrate/Isopropylmalate dehydrogenase-like | Dimeric isocitrate & isopropylmalate dehydrogenases | Alpha and beta proteins (a/b) | [GO:0022900 "electron transport chain" evidence=IMP] [GO:0004450  "isocitrate dehydrogenase (NADP+) activity" evidence=IEA;IDA] |
| HP0028 | b.80.6.1 | Single-stranded right-handed beta-helix | Stabilizer of iron transporter SufD | Stabilizer of iron transporter SufD | All beta proteins | [GO:0003674  "molecular_function" evidence=ND] [GO:0005575 "cellular_component"  evidence=ND] [GO:0008150 "biological_process" evidence=ND] |
| HP0029 | c.37.1.10 | P-loop containing nucleoside triphosphate hydrolases | P-loop containing nucleoside triphosphate hydrolases | Nitrogenase iron protein-like | Alpha and beta proteins (a/b) | [GO:0004141 "dethiobiotin synthase  activity" evidence=ISS] [GO:0009102 "biotin biosynthetic process"  evidence=ISS] |
| HP0030 | d.115.1.1 | YrdC/RibB | YrdC/RibB | YrdC-like | Alpha and beta proteins (a+b) |  |
| HP0031 | c.26.2.4 | Adenine nucleotide alpha hydrolase-like | Adenine nucleotide alpha hydrolase-like | Universal stress protein-like | Alpha and beta proteins (a/b) |  |
| HP0032 | f.4.3.3 | Transmembrane beta-barrels | Porins | Ligand-gated protein channel | Membrane and cell surface proteins and peptides | [GO:0003674  "molecular_function" evidence=ND] [GO:0005575 "cellular_component"  evidence=ND] [GO:0008150 "biological_process" evidence=ND] |
| HP0033 | c.37.1.20 | P-loop containing nucleoside triphosphate hydrolases | P-loop containing nucleoside triphosphate hydrolases | Extended AAA-ATPase domain | Alpha and beta proteins (a/b) | [GO:0006508 "proteolysis" evidence=ISS] [GO:0008233  "peptidase activity" evidence=ISS] |
| HP0034 | d.163.1.1 | DNA breaking-rejoining enzymes | DNA breaking-rejoining enzymes | Lambda integrase-like, catalytic core | Alpha and beta proteins (a+b) | [GO:0004068 "aspartate  1-decarboxylase activity" evidence=ISS] [GO:0015940 "pantothenate  biosynthetic process" evidence=ISS] |
| HP0035 | d.222.1.1 | YbaB-like | YbaB-like | YbaB-like | Alpha and beta proteins (a+b) | [GO:0003674  "molecular_function" evidence=ND] [GO:0005575 "cellular_component"  evidence=ND] [GO:0008150 "biological_process" evidence=ND] |
| HP0036 |  |  |  |  |  | [GO:0003824 "catalytic activity"  evidence=ISS] [GO:0008152 "metabolic process" evidence=ISS] |
| HP0037 | a.231.1.1 | EspA/CesA-like | EspA/CesA-like | EspA-like | All alpha proteins |  |
| HP0038 | d.17.4.6 | Cystatin-like | NTF2-like | VirB8-like | Alpha and beta proteins (a+b) | [GO:0003674  "molecular_function" evidence=ND] |
| HP0039 | b.68.1.1 | -bladed beta-propeller | Sialidases | Sialidases | All beta proteins | [GO:0005509 "calcium ion binding" evidence=IEA;ISS] [GO:0005634  "nucleus" evidence=ISM] [GO:0008150 "biological_process"  evidence=ND] [GO:0005829 "cytosol" evidence=IDA] |
| HP0041 | a.47.2.1 | STAT-like | t-snare proteins | t-snare proteins | All alpha proteins |  |
| HP0042 | c.55.3.10 | Ribonuclease H-like motif | Ribonuclease H-like | PIWI domain | Alpha and beta proteins (a/b) | [GO:0003674  "molecular_function" evidence=ND] [GO:0030255 "protein secretion by  the type IV secretion system" evidence=ISS] |
| HP0043 | c.68.1.20 | Nucleotide-diphospho-sugar transferases | Nucleotide-diphospho-sugar transferases | mannose-1-phosphate guanylyl transferase | Alpha and beta proteins (a/b) | [GO:0004476 "mannose-6-phosphate  isomerase activity" evidence=ISS] [GO:0008905 "mannose-phosphate  guanylyltransferase activity" evidence=ISS] [GO:0009103  "lipopolysaccharide biosynthetic process" evidence=ISS] |
| HP0044 | c.2.1.2 | NAD(P)-binding Rossmann-fold domains | NAD(P)-binding Rossmann-fold domains | Tyrosine-dependent oxidoreductases | Alpha and beta proteins (a/b) | [GO:0000271  "polysaccharide biosynthetic process" evidence=ISS] [GO:0008446  "GDP-mannose 4,6-dehydratase activity" evidence=ISS] |
| HP0045 | c.2.1.2 | NAD(P)-binding Rossmann-fold domains | NAD(P)-binding Rossmann-fold domains | Tyrosine-dependent oxidoreductases | Alpha and beta proteins (a/b) | [GO:0003824  "catalytic activity" evidence=ISS] [GO:0009225 "nucleotide-sugar  metabolic process" evidence=ISS] |
| HP0046 | a.30.1.1 | ROP-like | ROP protein | ROP protein | All alpha proteins |  |
| HP0047 | d.139.1.1 | PurM C-terminal domain-like | PurM C-terminal domain-like | PurM C-terminal domain-like | Alpha and beta proteins (a+b) | [GO:0003674  "molecular_function" evidence=ND] |
| HP0048 | d.115.1.1 | YrdC/RibB | YrdC/RibB | YrdC/RibB | Alpha and beta proteins (a+b) | [GO:0003998 "acylphosphatase activity" evidence=ISS] [GO:0006464  "cellular protein modification process" evidence=ISS] |
| HP0049 | d.126.1.6 | Pentein, beta/alpha-propeller | Pentein | Porphyromonas-type peptidylarginine deiminase | Alpha and beta proteins (a+b) | [GO:0004668  "protein-arginine deiminase activity" evidence=ISS] [GO:0019546  "arginine deiminase pathway" evidence=ISS] |
| HP0050 | d.288.1.1 | GTF2I-like repeat | GTF2I-like repeat | GTF2I-like repeat | Alpha and beta proteins (a+b) | [GO:0006306 "DNA methylation"  evidence=ISS] [GO:0009008 "DNA-methyltransferase activity"  evidence=ISS] |
| HP0051 | c.68.1.13 | Nucleotide-diphospho-sugar transferases | Nucleotide-diphospho-sugar transferases | Cytidylytransferase | Alpha and beta proteins (a/b) | [GO:0003886 "DNA  (cytosine-5-)-methyltransferase activity" evidence=ISS] [GO:0006304  "DNA modification" evidence=ISS] |
| HP0052 | g.17.1.1 | Cystine-knot cytokines | Cystine-knot cytokines | Platelet-derived growth factor-like | All small proteins |  |
| HP0053 | b.36.1.1 | PDZ domain-like | PDZ domain-like | PDZ domain-like | All beta proteins |  |
| HP0054 | c.66.1.26 | S-adenosyl-L-methionine-dependent methyltransferases | S-adenosyl-L-methionine-dependent methyltransferases | C5 cytosine-specific DNA methylase, DCM | Alpha and beta proteins (a/b) | [GO:0006306 "DNA methylation" evidence=ISS] |
| HP0055 | a.118.1.1 | alpha-alpha superhelix | ARM repeat | Armadillo repeat | All alpha proteins | [GO:0015824 "proline transport"  evidence=ISS] |
| HP0056 | c.1.23.2 | TIM beta/alpha-barrel | FAD-linked oxidoreductase | Proline dehydrohenase domain of bifunctional PutA protein | Alpha and beta proteins (a/b) | [GO:0003842  "1-pyrroline-5-carboxylate dehydrogenase activity" evidence=ISS]  [GO:0004657 "proline dehydrogenase activity" evidence=ISS]  [GO:0006562 "proline catabolic process" evidence=ISS] |
| HP0057 | c.3.1.3 | FAD/NAD(P)-binding domain | FAD/NAD(P)-binding domain | GDI-like N domain | Alpha and beta proteins (a/b) |  |
| HP0059 |  | Tropomyosin | Tropomyosin | Tropomyosin | Coiled coil proteins | [GO:0005545  "1-phosphatidylinositol binding" evidence=IEA] |
| HP0060 | c.55.1.6 | Ribonuclease H-like motif | Actin-like ATPase domain | ATPase domain of dehydratase reactivase alpha subunit | Alpha and beta proteins (a/b) |  |
| HP0061 | b.40.4.3 | OB-fold | Nucleic acid-binding proteins | Single strand DNA-binding domain, SSB | All beta proteins | [GO:0005484 "SNAP receptor activity"  evidence=IEA;TAS] [GO:0005634 "nucleus" evidence=ISM] [GO:0006886  "intracellular protein transport" evidence=IEA;TAS] |
| HP0062 | a.25.5.1 | Ferritin-like | HP0062-like | HP0062-like | All alpha proteins | [GO:0005198 "structural molecule activity" evidence=IEA] |
| HP0063 | f.42.1.1 | Mitochondrial carrier | Mitochondrial carrier | Mitochondrial carrier | Membrane and cell surface proteins and peptides | [GO:0005515 "protein binding"  evidence=IPI] |
| HP0064 | d.369.1.1 | SMI1/KNR4-like | SMI1/KNR4-like | SMI1/KNR4-like | Alpha and beta proteins (a+b) |  |
| HP0065 | a.238.1.3 | BAR/IMD domain-like | BAR/IMD domain-like | IMD domain | All alpha proteins | GO:0000166 "nucleotide binding" evidence=IEA]  [GO:0005525 "GTP binding" evidence=IEA] |
| HP0066 | c.37.1.19 | P-loop containing nucleoside triphosphate hydrolases | P-loop containing nucleoside triphosphate hydrolases | Tandem AAA-ATPase domain | Alpha and beta proteins (a/b) | [GO:0003674 "molecular_function" evidence=ND] |
| HP0067 | b.80.6.1 | Single-stranded right-handed beta-helix | Stabilizer of iron transporter SufD | Stabilizer of iron transporter SufD | All beta proteins | [GO:0005515 "protein binding"  evidence=IPI] |
| HP0068 | c.37.1.8 | P-loop containing nucleoside triphosphate hydrolases | P-loop containing nucleoside triphosphate hydrolases | G proteins | Alpha and beta proteins (a/b) | [GO:0016530  "metallochaperone activity" evidence=ISS] |
| HP0069 | a.238.1.3 | BAR/IMD domain-like | BAR/IMD domain-like | IMD domain | All alpha proteins | [GO:0016530  "metallochaperone activity" evidence=ISS] |
| HP0070 | b.107.1.1 | Urease metallochaperone UreE, N-terminal domain | Urease metallochaperone UreE, N-terminal domain | Urease metallochaperone UreE, N-terminal domain | All beta proteins | [GO:0006457 "protein folding"  evidence=ISS] |
| HP0071 | f.35.1.1 | Multidrug efflux transporter AcrB transmembrane domain | Multidrug efflux transporter AcrB transmembrane domain | Multidrug efflux transporter AcrB transmembrane domain | Membrane and cell surface proteins and peptides |  |
| HP0072 | c.1.9.2 | TIM beta/alpha-barrel | Metallo-dependent hydrolases | alpha-subunit of urease, catalytic domain | Alpha and beta proteins (a/b) | [GO:0009039 "urease activity"  evidence=ISS] [GO:0019627 "urea metabolic process" evidence=ISS] |
| HP0073 | b.85.3.1 | beta-clip | Urease, beta-subunit | Urease, beta-subunit | All beta proteins | [GO:0006807 "nitrogen  compound metabolic process" evidence=IEA;IDA] [GO:0009039 "urease  activity" evidence=IEA;ISS;IDA] |
| HP0074 | f.16.1.1 | Gated mechanosensitive channel | Gated mechanosensitive channel | Gated mechanosensitive channel | Membrane and cell surface proteins and peptides | [GO:0005887 "integral to plasma  membrane" evidence=ISS] [GO:0006508 "proteolysis" evidence=ISS]  [GO:0009306 "protein secretion" evidence=ISS] |
| HP0075 | c.84.1.1 | Phosphoglucomutase, first 3 domains | Phosphoglucomutase, first 3 domains | Phosphoglucomutase, first 3 domains | Alpha and beta proteins (a/b) |  |
| HP0076 | a.7.6.1 | Spectrin repeat-like | Ribosomal protein S20 | Ribosomal protein S20 | All alpha proteins | [GO:0003735 "structural constituent  of ribosome" evidence=ISS] [GO:0042254 "ribosome biogenesis"  evidence=ISS] |
| HP0077 | c.23.15.1 | Flavodoxin-like | Ribosomal protein S2 | Ribosomal protein S2 | Alpha and beta proteins (a/b) |  |
| HP0078 | b.159.1.1 | AOC barrel-like | Allene oxide cyclase-like | Allene oxide cyclase-like | All beta proteins | [GO:0008270 "zinc ion  binding" evidence=IEA] [GO:0005622 "intracellular" evidence=IEA]  [GO:0005575 "cellular_component" evidence=ND] [GO:0003674  "molecular_function" evidence=ND] |
| HP0079 | f.1.1.1 | Toxins' membrane translocation domains | Colicin | Colicin | Membrane and cell surface proteins and peptides | [GO:0005576 "extracellular region" evidence=IEA] |
| HP0080 | d.301.1.1 | L35p-like | L35p-like | Ribosomal protein L35p | Alpha and beta proteins (a+b) |  |
| HP0081 | d.58.60.1 | Ferredoxin-like | Bacterial polysaccharide co-polymerase-like | FepE-like | Alpha and beta proteins (a+b) |  |
| HP0082 | d.110.6.2 | Profilin-like | Sensory domain-like | YkuI C-terminal domain-like | Alpha and beta proteins (a+b) | [GO:0004871 "signal  transducer activity" evidence=ISS] [GO:0004872 "receptor activity"  evidence=ISS] [GO:0006935 "chemotaxis" evidence=ISS] |
| HP0083 | d.14.1.1 | Ribosomal protein S5 domain 2-like | Ribosomal protein S5 domain 2-like | Translational machinery components | Alpha and beta proteins (a+b) | [GO:0003735 "structural constituent  of ribosome" evidence=ISS] [GO:0006412 "translation" evidence=ISS]  [GO:0042254 "ribosome biogenesis" evidence=ISS] |
| HP0084 |  | Ribosome and ribosomal fragments | Ribosome and ribosomal fragments | Ribosome complexes | Low resolution protein structures | [GO:0003735 "structural constituent  of ribosome" evidence=ISS] [GO:0042254 "ribosome biogenesis"  evidence=ISS] |
| HP0085 | f.23.33.1 | Single transmembrane helix | Photosystem II 10 kDa phosphoprotein PsbH | PsbH-like | Membrane and cell surface proteins and peptides |  |
| HP0086 | c.3.1.2 | FAD/NAD(P)-binding domain | FAD/NAD(P)-binding domain | FAD-linked reductases, N-terminal domain | Alpha and beta proteins (a/b) | [GO:0006099  "tricarboxylic acid cycle" evidence=ISS] [GO:0008924 "malate  dehydrogenase (quinone) activity" evidence=ISS] |
| HP0087 | d.3.1.16 | Cysteine proteinases | Cysteine proteinases | NlpC/P60 | Alpha and beta proteins (a+b) | [GO:0003674 "molecular_function"  evidence=ND] [GO:0008150 "biological_process" evidence=ND] |
| HP0088 | a.177.1.1 | Sigma2 domain of RNA polymerase sigma factors | Sigma2 domain of RNA polymerase sigma factors | Sigma2 domain of RNA polymerase sigma factors | All alpha proteins | [GO:0006352  "DNA-dependent transcription, initiation" evidence=ISS] [GO:0016987  "sigma factor activity" evidence=ISS] |
| HP0089 | c.56.2.1 | Phosphorylase/hydrolase-like | Purine and uridine phosphorylases | Purine and uridine phosphorylases | Alpha and beta proteins (a/b) | [GO:0008152 "metabolic process"  evidence=ISS] [GO:0008782 "adenosylhomocysteine nucleosidase  activity" evidence=ISS] [GO:0008930 "methylthioadenosine  nucleosidase activity" evidence=ISS] |
| HP0090 | c.19.1.1 | FabD/lysophospholipase-like | FabD/lysophospholipase-like | FabD/lysophospholipase-like | Alpha and beta proteins (a/b) | [GO:0004314 "[acyl-carrier-protein] S-malonyltransferase activity"  evidence=ISS] [GO:0006633 "fatty acid biosynthetic process"  evidence=ISS] |
| HP0091 | c.52.1.18 | Restriction endonuclease-like | Restriction endonuclease-like | Hjc-like | Alpha and beta proteins (a/b) | [GO:0009307 "DNA restriction-modification system" evidence=ISS]  [GO:0015666 "restriction endodeoxyribonuclease activity"  evidence=ISS] |
| HP0092 | c.66.1.11 | S-adenosyl-L-methionine-dependent methyltransferases | S-adenosyl-L-methionine-dependent methyltransferases | Type II DNA methylase | Alpha and beta proteins (a/b) | [GO:0006306 "DNA methylation"  evidence=ISS] [GO:0009008 "DNA-methyltransferase activity"  evidence=ISS] |
| HP0093 | c.2.1.2 | NAD(P)-binding Rossmann-fold domains | NAD(P)-binding Rossmann-fold domains | Tyrosine-dependent oxidoreductases | Alpha and beta proteins (a/b) | [GO:0008417  "fucosyltransferase activity" evidence=ISS] [GO:0009243 "O antigen  biosynthetic process" evidence=ISS] [GO:0036065 "fucosylation"  evidence=ISS |
| HP0094 | c.2.1.2 | NAD(P)-binding Rossmann-fold domains | NAD(P)-binding Rossmann-fold domains | Tyrosine-dependent oxidoreductases | Alpha and beta proteins (a/b) | [GO:0008417  "fucosyltransferase activity" evidence=ISS] [GO:0009243 "O antigen  biosynthetic process" evidence=ISS] [GO:0036065 "fucosylation"  evidence=ISS] |
| HP0095 | a.123.1.1 | Nuclear receptor ligand-binding domain | Nuclear receptor ligand-binding domain | Nuclear receptor ligand-binding domain | All alpha proteins | [GO:0006508 "proteolysis"  evidence=ISS] |
| HP0096 | c.2.1.4 | NAD(P)-binding Rossmann-fold domains | NAD(P)-binding Rossmann-fold domains | Formate/glycerate dehydrogenases, NAD-domain | Alpha and beta proteins (a/b) | [GO:0003824 "catalytic activity" evidence=ISS] [GO:0008152  "metabolic process" evidence=ISS] |
| HP0097 | b.1.11.1 | Immunoglobulin-like beta-sandwich | PapD-like | Pilus chaperone | All beta proteins | [GO:0003674  "molecular_function" evidence=ND] [GO:0008150 "biological_process"  evidence=ND] [GO:0009279 "cell outer membrane" evidence=ISS] |
| HP0098 | c.79.1.1 | Tryptophan synthase beta subunit-like PLP-dependent enzymes | Tryptophan synthase beta subunit-like PLP-dependent enzymes | Tryptophan synthase beta subunit-like PLP-dependent enzymes | Alpha and beta proteins (a/b) | [GO:0004795 "threonine synthase  activity" evidence=ISS] [GO:0009088 "threonine biosynthetic  process" evidence=ISS] |
| HP0099 | d.110.6.2 | Profilin-like | Sensory domain-like | YkuI C-terminal domain-like | Alpha and beta proteins (a+b) | [GO:0004871 "signal  transducer activity" evidence=ISS] [GO:0004872 "receptor activity"  evidence=ISS] [GO:0006935 "chemotaxis" evidence=ISS] |
| HP0100 | a.138.1.3 | Multiheme cytochromes | Multiheme cytochromes | Di-heme elbow motif | All alpha proteins | [GO:0003674  "molecular_function" evidence=ND] [GO:0005575 "cellular_component"  evidence=ND] [GO:0008150 "biological_process" evidence=ND] |
| HP0101 | f.4.3 .2 | Transmembrane beta-barrels | Porins | Maltoporin-like | Membrane and cell surface proteins and peptides |  |
| HP0102 | c.68.1.1 | Nucleotide-diphospho-sugar transferases | Nucleotide-diphospho-sugar transferases | Spore coat polysaccharide biosynthesis protein SpsA | Alpha and beta proteins (a/b) | [GO:0000271  "polysaccharide biosynthetic process" evidence=ISS] [GO:0016757  "transferase activity, transferring glycosyl groups" evidence=ISS] |
| HP0103 | d.110.6.2 | Profilin-like | Sensory domain-like | YkuI C-terminal domain-like | Alpha and beta proteins (a+b) | [GO:0004871 "signal transducer activity"  evidence=ISS] [GO:0004872 "receptor activity" evidence=ISS]  [GO:0006935 "chemotaxis" evidence=ISS] |
| HP0104 | d.159.1.2 | Metallo-dependent phosphatases | Metallo-dependent phosphatases | nucleotidase (syn. UDP-sugar hydrolase), N-terminal domain | Alpha and beta proteins (a+b) | [GO:0006139 "nucleobase-containing  compound metabolic process" evidence=ISS] [GO:0008663  "2',3'-cyclic-nucleotide 2'-phosphodiesterase activity"  evidence=ISS] |
| HP0105 | d.185.1.2 | LuxS/MPP-like metallohydrolase | LuxS/MPP-like metallohydrolase | Autoinducer-2 production protein LuxS | Alpha and beta proteins (a+b) | [GO:0009372 "quorum  sensing" evidence=ISS] |
| HP0106 | c.67.1.3 | PLP-dependent transferase-like | PLP-dependent transferase-like | Cystathionine synthase-like | Alpha and beta proteins (a/b) | [GO:0004121 "cystathionine  beta-lyase activity" evidence=ISS] [GO:0009086 "methionine  biosynthetic process" evidence=ISS] |
| HP0107 | c.79.1.1 | Tryptophan synthase beta subunit-like PLP-dependent enzymes | Tryptophan synthase beta subunit-like PLP-dependent enzymes | Tryptophan synthase beta subunit-like PLP-dependent enzymes | Alpha and beta proteins (a/b) | [GO:0004124 "cysteine synthase activity" evidence=ISS]  [GO:0006535 "cysteine biosynthetic process from serine"  evidence=ISS] |
| HP0108 | c.52.1.9 | Restriction endonuclease-like | Restriction endonuclease-like | Restriction endonuclease NaeI | Alpha and beta proteins (a/b) |  |
| HP0109 | c.55.1.1 | Ribonuclease H-like motif | Actin-like ATPase domain | Actin/HSP70 | Alpha and beta proteins (a/b) | [GO:0009408 "response to heat"  evidence=IDA] |
| HP0110 |  |  |  |  |  | [GO:0000774 "adenyl-nucleotide  exchange factor activity" evidence=ISS] [GO:0006950 "response to  stress" evidence=ISS] |
| HP0111 | d.110.2.3 | Profilin-like | GAF domain-like | HrcA C-terminal domain-like | Alpha and beta proteins (a+b) | [GO:0009408 "response  to heat" evidence=ISS] |
| HP0112 | c.74.1.1 | AraD/HMP-PK domain-like | AraD/HMP-PK domain-like | AraD-like aldolase/epimerase | Alpha and beta proteins (a/b | [GO:0008150 "biological_process"  evidence=ND] |
| HP0113 | c.1.2.5 | TIM beta/alpha-barrel | Ribulose-phoshate binding barrel | NanE-like | Alpha and beta proteins (a/b) | [GO:0046982 "protein  heterodimerization activity" evidence=IEA] |
| HP0114 | c.130.1.1 | Alpha-2,3/8-sialyltransferase CstII | Alpha-2,3/8-sialyltransferase CstII | Alpha-2,3/8-sialyltransferase CstII | Alpha and beta proteins (a/b) | [GO:0001539 "ciliary or flagellar  motility" evidence=IGI] [GO:0003674 "molecular_function"  evidence=ND] |
| HP0115 | e.32.1.1 | Phase 1 flagellin | Phase 1 flagellin | Phase 1 flagellin | Multi-domain proteins (alpha and beta) | [GO:0001539 "ciliary  or flagellar motility" evidence=ISS] [GO:0005198 "structural  molecule activity" evidence=ISS] |
| HP0116 | e.10.1.1 | Prokaryotic type I DNA topoisomerase | Prokaryotic type I DNA topoisomerase | Prokaryotic type I DNA topoisomerase | Multi-domain proteins (alpha and beta) | [GO:0003677 "DNA binding"  evidence=ISS] [GO:0003917 "DNA topoisomerase type I activity"  evidence=ISS] [GO:0006260 "DNA replication" evidence=ISS]  [GO:0006265 "DNA topological change" evidence=ISS] |
| HP0117 | c.1.28.3 | TIM beta/alpha-barrel | Radical SAM enzymes | MoCo biosynthesis proteins | Alpha and beta proteins (a/b) | [GO:0003824 "catalytic  activity" evidence=ISS] [GO:0008152 "metabolic process"  evidence=ISS] |
| HP0118 | b.110.1.1 | Cloacin translocation domain | Cloacin translocation domain | Cloacin translocation domain | All beta proteins | [GO:0003676 "nucleic acid binding" evidence=IEA] [GO:0008150  "biological_process" evidence=ND] [GO:0003677 "DNA binding"  evidence=IEA] |
| HP0119 | b.110.1.1 | Cloacin translocation domain | Cloacin translocation domain | Cloacin translocation domain | All beta proteins |  |
| HP0120 | f.5.1.1 | Outer membrane efflux proteins (OEP) | Outer membrane efflux proteins (OEP) | Outer membrane efflux proteins (OEP) | Membrane and cell surface proteins and peptides | [GO:0005575 "cellular_component"  evidence=ND] |
| HP0121 | c.1.12.2 | TIM beta/alpha-barrel | Phosphoenolpyruvate/pyruvate domain | Pyruvate phosphate dikinase, C-terminal domain | Alpha and beta proteins (a/b) | [GO:0006094  "gluconeogenesis" evidence=ISS] [GO:0008986 "pyruvate, water  dikinase activity" evidence=ISS] |
| HP0122 | f.23.1.1 | Single transmembrane helix | Mitochondrial cytochrome c oxidase subunit IV | Mitochondrial cytochrome c oxidase subunit IV | Membrane and cell surface proteins and peptides |  |
| HP0123 | d.104.1.1 | Class II aaRS and biotin synthetases | Class II aaRS and biotin synthetases | Class II aminoacyl-tRNA synthetase (aaRS)-like, catalytic domain | Alpha and beta proteins (a+b) | [GO:0004829 "threonine-tRNA ligase  activity" evidence=ISS] [GO:0006435 "threonyl-tRNA aminoacylation"  evidence=ISS] |
| HP0124 | d.15.8.1 | beta-Grasp (ubiquitin-like) | Translation initiation factor IF3, N-terminal domain | Translation initiation factor IF3, N-terminal domain | Alpha and beta proteins (a+b) | [GO:0003743  "translation initiation factor activity" evidence=ISS] [GO:0006413  "translational initiation" evidence=ISS] |
| HP0125 | b.81.1.5 | Single-stranded left-handed beta-helix | Trimeric LpxA-like enzymes | gamma-carbonic anhydrase-like | All beta proteins | [GO:0003735 "structural constituent  of ribosome" evidence=ISS] [GO:0042254 "ribosome biogenesis"  evidence=ISS] |
| HP0126 | c.82.1.1 | ALDH-like | ALDH-like | ALDH-like | Alpha and beta proteins (a/b) | [GO:0003735 "structural constituent  of ribosome" evidence=ISS] |
| HP0127 | f.4.5.1 | Transmembrane beta-barrels | Autotransporter | Autotransporter | Membrane and cell surface proteins and peptides |  |
| HP0128 | a.2.5.1 | Long alpha-hairpin | Prefoldin | Prefoldin | All alpha proteins |  |
| HP0129 | b.34.15.1 | SH3-like barrel | Hypothetical protein YfhH | Hypothetical protein YfhH | All beta proteins | [GO:0008270 "zinc ion binding"  evidence=IEA] [GO:0003676 "nucleic acid binding" evidence=IEA] |
| HP0130 | a.25.1.1 | Ferritin-like | Ferritin-like | Ferritin-like | All alpha proteins | [GO:0003677 "DNA binding" evidence=IEA]  Programmed cell death protein 5 |
| HP0131 | a.24.8.1 | Four-helical up-and-down bundle | Proteasome activator | Proteasome activator | All alpha proteins |  |
| HP0132 | d.81.2.1 | FwdE/GAPDH domain-like | Serine metabolism enzymes domain | Serine dehydratase beta chain-like | Alpha and beta proteins (a+b) | [GO:0003941 "L-serine ammonia-lyase  activity" evidence=ISS] [GO:0006094 "gluconeogenesis" evidence=ISS] |
| HP0133 | f.54.1.1 | SNF-like | SNF-like | SNF-like | Membrane and cell surface proteins and peptides | [GO:0015194 "L-serine  transmembrane transporter activity" evidence=ISS] [GO:0015825  "L-serine transport" evidence=ISS] |
| HP0134 | c.1.10.8 | TIM beta/alpha-barrel | Aldolase | Class-II DAHP synthetase | Alpha and beta proteins (a/b) | [GO:0003849  "3-deoxy-7-phosphoheptulonate synthase activity" evidence=ISS]  [GO:0009423 "chorismate biosynthetic process" evidence=ISS] |
| HP0135 | c.94.1.1; | Periplasmic binding protein-like II | Periplasmic binding protein-like II | Phosphate binding protein-like | Alpha and beta proteins (a/b) |  |
| HP0136 | c.47.1.10 | Thioredoxin fold | Thioredoxin fold | Glutathione peroxidase-like | Alpha and beta proteins (a/b) | [GO:0016209  "antioxidant activity" evidence=ISS] |
| HP0137 | c.124.1.7 | NagB/RpiA/CoA transferase-like | NagB/RpiA/CoA transferase-like | YkgG-like | Alpha and beta proteins (a/b) | [GO:0003674 "molecular_function"  evidence=ND] [GO:0005575 "cellular_component" evidence=ND]  [GO:0008150 "biological_process" evidence=ND] |
| HP0138 | e.26.1.2 | Prismane protein-like | Prismane protein-like | Carbon monoxide dehydrogenase | Multi-domain proteins (alpha and beta) | [GO:0009055 "electron  carrier activity" evidence=ISS] |
|  |  |  |  |  |  |  |
| HP0139 | e.26.1.2 | Prismane protein-like | Prismane protein-like | Carbon monoxide dehydrogenase | Multi-domain proteins (alpha and beta | [GO:0003674  "molecular_function" evidence=ND] [GO:0008150 "biological_process"  evidence=ND] |
| HP0140 |  |  |  |  |  | [GO:0015129 "lactate transmembrane  transporter activity" evidence=ISS] [GO:0015727 "lactate transport"  evidence=ISS] |
| HP0141 |  |  |  |  |  | [GO:0015129 "lactate transmembrane  transporter activity" evidence=ISS] [GO:0015727 "lactate transport"  evidence=ISS] |
| HP0142 | a.96.1.2 | DNA-glycosylase | DNA-glycosylase | Mismatch glycosylase | All alpha proteins | [GO:0004844 "uracil  DNA N-glycosylase activity" evidence=ISS] [GO:0006260 "DNA  replication" evidence=ISS] |
| HP0144 | f.24.1.1 | Cytochrome c oxidase subunit I-like | Cytochrome c oxidase subunit I-like | Cytochrome c oxidase subunit I-like | Membrane and cell surface proteins and peptides | [GO:0004129  "cytochrome-c oxidase activity" evidence=ISS] |
| HP0145 | c.81.1.1 | Formate dehydrogenase/DMSO reductase, domains 1-3 | Formate dehydrogenase/DMSO reductase, domains 1-3 | Formate dehydrogenase/DMSO reductase, domains 1-3 | Alpha and beta proteins (a/b) | [GO:0004129  "cytochrome-c oxidase activity" evidence=ISS] |
| HP0146 |  |  |  |  |  | [GO:0004129  "cytochrome-c oxidase activity" evidence=ISS] |
| HP0147 | e.8.1.4 | DNA/RNA polymerases | DNA/RNA polymerases | RNA-dependent RNA-polymerase | Multi-domain proteins (alpha and beta) | [GO:0004129  "cytochrome-c oxidase activity" evidence=ISS] |
| HP0148 | d.106.1.4 | SCP-like | SCP-like | EF1021 C-terminal domain-like | Alpha and beta proteins (a+b) |  |
| HP0149 | d.92.1.15 | Zincin-like | Metalloproteases ("zincins"), catalytic domain | Predicted metal-dependent hydrolase | Alpha and beta proteins (a+b) |  |
| HP0150 | b.42.1.2 | beta-Trefoil | Cytokine | Interleukin-1 (IL-1) | All beta proteins |  |
| HP0151 | d.2.1.3 | Lysozyme-like | Lysozyme-like | Phage lysozyme | Alpha and beta proteins (a+b) |  |
| HP0152 | c.94.1.1 | Periplasmic binding protein-like II | Periplasmic binding protein-like II | Phosphate binding protein-like | Alpha and beta proteins (a/b) | [GO:0003674  "molecular_function" evidence=ND] [GO:0005575 "cellular_component"  evidence=ND] [GO:0008150 "biological_process" evidence=ND] |
| HP0153 | c.37.1.11 | P-loop containing nucleoside triphosphate hydrolases | P-loop containing nucleoside triphosphate hydrolases | RecA protein-like (ATPase-domain) | Alpha and beta proteins (a/b) | [GO:0003677 "DNA binding"  evidence=ISS] [GO:0006281 "DNA repair" evidence=ISS] |
| HP0154 | c.1.11.1 | TIM beta/alpha-barrel | Enolase C-terminal domain-like | Enolase | Alpha and beta proteins (a/b) | [GO:0004634 "phosphopyruvate hydratase activity"  evidence=ISS] [GO:0006096 "glycolysis" evidence=ISS] |
| HP0155 | a.2.7.2 | Long alpha-hairpin | tRNA-binding arm | Phenylalanyl-tRNA synthetase (PheRS) | All alpha proteins | [GO:0003674  "molecular_function" evidence=ND] [GO:0005575 "cellular_component"  evidence=ND] [GO:0008150 "biological_process" evidence=ND] |
| HP0156 |  |  |  |  |  | [GO:0003674  "molecular_function" evidence=ND] [GO:0005575 "cellular_component"  evidence=ND] [GO:0008150 "biological_process" evidence=ND] |
| HP0157 | c.37.1.2 | P-loop containing nucleoside triphosphate hydrolases | P-loop containing nucleoside triphosphate hydrolases | Shikimate kinase (AroK) | Alpha and beta proteins (a/b) | [GO:0004765 "shikimate kinase  activity" evidence=ISS] [GO:0009423 "chorismate biosynthetic  process" evidence=ISS] |
| HP0158 | d.110.6.2 | Profilin-like | Sensory domain-like | YkuI C-terminal domain-like | Alpha and beta proteins (a+b) | [GO:0006487 "protein  N-linked glycosylation" evidence=IMP] [GO:0016757 "transferase  activity, transferring glycosyl groups" evidence=IMP] |
| HP0159 | c.68.1.4 | Nucleotide-diphospho-sugar transferases | Nucleotide-diphospho-sugar transferases | Galactosyltransferase LgtC | Alpha and beta proteins (a/b) | [GO:0008918 "lipopolysaccharide 3-alpha-galactosyltransferase  activity" evidence=IEA] [GO:0009244 "lipopolysaccharide core region  biosynthetic process" evidence=IEA] |
| HP0160 | a.118.18.1 | alpha-alpha superhelix | HCP-like | HCP-like | All alpha proteins | [GO:0003674 "molecular_function"  evidence=ND] [GO:0008150 "biological_process" evidence=ND] |
| HP0161 | d.144.1.7 | Protein kinase-like (PK-like) | Protein kinase-like (PK-like) | Protein kinases, catalytic subunit | Alpha and beta proteins (a+b) |  |
| HP0162 | e.39.1.1 | YebC-like | YebC-like | YebC-like | Multi-domain proteins (alpha and beta) | [GO:0003674  "molecular_function" evidence=ND] [GO:0005575 "cellular_component"  evidence=ND] [GO:0008150 "biological_process" evidence=ND] |
| HP0163 | c.1.10.3 | TIM beta/alpha-barrel | Aldolase | 5-aminolaevulinate dehydratase, ALAD (porphobilinogen synthase) | Alpha and beta proteins (a/b) | [GO:0004655 "porphobilinogen synthase  activity" evidence=ISS] [GO:0006779 "porphyrin-containing compound  biosynthetic process" evidence=ISS] |
| HP0164 | d.122.1.3 | ATPase domain of HSP90 chaperone/DNA topoisomerase II/histidine kinase | ATPase domain of HSP90 chaperone/DNA topoisomerase II/histidine kinase | Histidine kinase | Alpha and beta proteins (a+b) | [GO:0000155 "phosphorelay sensor  kinase activity" evidence=ISS] [GO:0000160 "phosphorelay signal  transduction system" evidence=ISS] |
| HP0165 | f.17.4.1 | Transmembrane helix hairpin | Htr2 transmembrane domain-like | Htr2 transmembrane domain-like | Membrane and cell surface proteins and peptides |  |
| HP0166 | a.4.6.1 | DNA/RNA-binding 3-helical bundle | C-terminal effector domain of the bipartite response regulators | PhoB-like | All alpha proteins | [GO:0000156  "phosphorelay response regulator activity" evidence=ISS]  [GO:0000160 "phosphorelay signal transduction system" evidence=ISS]  [GO:0003677 "DNA binding" evidence=ISS] [GO:0006355 "regulation of  transcription, DNA-dependent" evidence=ISS] |
| HP0167 |  |  |  |  |  | [GO:0003674  "molecular_function" evidence=ND] [GO:0005575 "cellular_component"  evidence=ND] [GO:0008150 "biological_process" evidence=ND] |
| HP0168 | a.118.8.1 | alpha-alpha superhelix | TPR-like | Tetratricopeptide repeat (TPR) | All alpha proteins | [GO:0003674  "molecular_function" evidence=ND] [GO:0005575 "cellular_component"  evidence=ND] [GO:0008150 "biological_process" evidence=ND] |
| HP0169 | c.1.12.5 | TIM beta/alpha-barrel | Phosphoenolpyruvate/pyruvate domain | HpcH/HpaI aldolase | Alpha and beta proteins (a/b) | [GO:0006508 "proteolysis"  evidence=ISS] [GO:0008233 "peptidase activity" evidence=ISS] |
| HP0170 | a.7.12.1 | Spectrin repeat-like | PhoU-like | PhoU-like | All alpha proteins | [GO:0003674  "molecular_function" evidence=ND] [GO:0005575 "cellular_component"  evidence=ND] [GO:0008150 "biological_process" evidence=ND] |
| HP0171 | e.38.1.1 | Release factor | Release factor | Release factor | Multi-domain proteins (alpha and beta) | [GO:0003747  "translation release factor  activity" evidence=ISS] [GO:0006415  "translational termination" evidence=ISS] |
| HP0172 | b.103.1.1 | MoeA N-terminal region -like | MoeA N-terminal region -like | MoeA N-terminal region -like | All beta proteins | [GO:0003824  "catalytic activity" evidence=ISS] [GO:0006777 "Mo-molybdopterin  cofactor biosynthetic process" evidence=ISS] |
| HP0173 | e.8.1.4 | DNA/RNA polymerases | DNA/RNA polymerases | RNA-dependent RNA-polymerase | Multi-domain proteins (alpha and beta) | [GO:0008565 "protein  transporter activity" evidence=ISS] [GO:0009296 "flagellum  assembly" evidence=ISS] [GO:0016020 "membrane" evidence=ISS] |
| HP0174 | a.25.1.2 | Ferritin-like | Ferritin-like | Ribonucleotide reductase-like | All alpha proteins | [GO:0003674 "molecular_function"  evidence=ND] [GO:0008150 "biological_process" evidence=ND] |
| HP0175 | f.21.1.2 | Heme-binding four-helical bundle | Transmembrane di-heme cytochromes | Cytochrome b of cytochrome bc1 complex (Ubiquinol-cytochrome c reductase) | Membrane and cell surface proteins and peptides | [GO:0003674  "molecular_function" evidence=ND] |
|  |  |  |  |  |  |  |
| HP0176 | c.1.10.2 | TIM beta/alpha-barrel | Aldolase | Class II FBP aldolase | Alpha and beta proteins (a/b) | [GO:0004332 "fructose-bisphosphate aldolase activity" evidence=ISS]  [GO:0006096 "glycolysis" evidence=ISS] |
| HP0177 | b.40.4.5 | OB-fold | Nucleic acid-binding proteins | Cold shock DNA-binding domain-like | All beta proteins | [GO:0003746  "translation elongation factor activity" evidence=ISS] [GO:0006414  "translational elongation" evidence=ISS] |
| HP0178 | c.1.10.6 | TIM beta/alpha-barrel | Aldolase | NeuB-like | Alpha and beta proteins (a/b) | [GO:0009103  "lipopolysaccharide biosynthetic process" evidence=ISS] |
| HP0179 | c.37.1.12 | P-loop containing nucleoside triphosphate hydrolases | P-loop containing nucleoside triphosphate hydrolases | ABC transporter ATPase domain-like | Alpha and beta proteins (a/b) | [GO:0006810  "transport" evidence=ISS] [GO:0042626 "ATPase activity, coupled to  transmembrane movement of substances" evidence=ISS] |
| HP0180 | d.160.1.2 | Carbon-nitrogen hydrolase | Carbon-nitrogen hydrolase | Carbamilase | Alpha and beta proteins (a+b) | [GO:0006464 "cellular  protein modification process" evidence=ISS] [GO:0016020 "membrane"  evidence=ISS] [GO:0016410 "N-acyltransferase activity"  evidence=ISS] |
| HP0181 | f.51.1.1 | Rhomboid-like | Rhomboid-like | Rhomboid-like | Membrane and cell surface proteins and peptides | [GO:0003674 "molecular_function"  evidence=ND] [GO:0008150 "biological_process" evidence=ND] |
| HP0182 | d.104.1.1 | Class II aaRS and biotin synthetases | Class II aaRS and biotin synthetases | Class II aminoacyl-tRNA synthetase (aaRS)-like, catalytic domain | Alpha and beta proteins (a+b) | [GO:0004824 "lysine-tRNA ligase  activity" evidence=ISS] [GO:0006430 "lysyl-tRNA aminoacylation"  evidence=ISS] |
| HP0183 | c.67.1.4 | PLP-dependent transferase-like | PLP-dependent transferase-like | GABA-aminotransferase-like | Alpha and beta proteins (a/b) | [GO:0004372 "glycine  hydroxymethyltransferase activity" evidence=ISS] [GO:0006545  "glycine biosynthetic process" evidence=ISS] |
| HP0184 | d.264.1.3 | Prim-pol domain | Prim-pol domain | HP0184-like | Alpha and beta proteins (a+b) | [GO:0003674  "molecular_function" evidence=ND] [GO:0005575 "cellular_component"  evidence=ND] [GO:0008150 "biological_process" evidence=ND] |
| HP0185 | b.68.1.1 | 6-bladed beta-propeller | Sialidases | Sialidases (neuraminidases) | All beta proteins |  |
| HP0186 | d.4.1.6 | His-Me finger endonucleases | His-Me finger endonucleases | Endonuclease I | Alpha and beta proteins (a+b) | [GO:0003674  "molecular_function" evidence=ND] [GO:0008150 "biological_process"  evidence=ND] |
| HP0187 | d.268.1.2 | ParB/Sulfiredoxin | ParB/Sulfiredoxin | Hypothetical protein PF0380 | Alpha and beta proteins (a+b) |  |
| HP0188 | d.15.1.1 | beta-Grasp (ubiquitin-like) | Ubiquitin-like | Ubiquitin-related | Alpha and beta proteins (a+b) |  |
| HP0189 | f.32.1.1 | a domain/subunit of cytochrome bc1 complex (Ubiquinol-cytochrome c reductase) | a domain/subunit of cytochrome bc1 complex (Ubiquinol-cytochrome c reductase) | a domain/subunit of cytochrome bc1 complex (Ubiquinol-cytochrome c reductase) | Membrane and cell surface proteins and peptides | [GO:0003674  "molecular_function" evidence=ND] [GO:0005575 "cellular_component"  evidence=ND] [GO:0008150 "biological_process" evidence=ND] |
| HP0190 | d.136.1.2 | Phospholipase D/nuclease | Phospholipase D/nuclease | Phospholipase D | Alpha and beta proteins (a+b) | [GO:0090483  "phosphatidylglycerol-phosphatidylethanolamine  phosphatidyltransferase activity" evidence=IDA] [GO:0032049  "cardiolipin biosynthetic process" evidence=IMP] [GO:0016740  "transferase activity" evidence=IEA] |
| HP0191 | a.1.2.1 | Globin-like | alpha-helical ferredoxin | Fumarate reductase/Succinate dehydogenase iron-sulfur protein, C-terminal domain | All alpha proteins | [GO:0000104  "succinate dehydrogenase activity" evidence=ISS] [GO:0009055  "electron carrier activity" evidence=ISS] |
| HP0192 | c.3.1.4 | FAD/NAD(P)-binding domain | FAD/NAD(P)-binding domain | Succinate dehydrogenase/fumarate reductase flavoprotein N-terminal domain | Alpha and beta proteins (a/b) | [GO:0000104  "succinate dehydrogenase activity" evidence=ISS] [GO:0006099  "tricarboxylic acid cycle" evidence=ISS] |
| HP0193 | f.21.2.1 | Heme-binding four-helical bundle | Fumarate reductase respiratory complex transmembrane subunits | Fumarate reductase respiratory complex transmembrane subunits | Membrane and cell surface proteins and peptides | [GO:0009055 "electron  carrier activity" evidence=ISS] [GO:0009061 "anaerobic respiration"  evidence=ISS] [GO:0045284 "plasma membrane fumarate reductase  complex" evidence=ISS] |
| HP0194 | c.1.1.1 | TIM beta/alpha-barrel | Triosephosphate isomerase (TIM) | Triosephosphate isomerase (TIM) | Alpha and beta proteins (a/b) | [GO:0004807 "triose-phosphate  isomerase activity" evidence=ISS] [GO:0006096 "glycolysis"  evidence=ISS] |
| HP0195 | c.2.1.2 | NAD(P)-binding Rossmann-fold domains | NAD(P)-binding Rossmann-fold domains | Tyrosine-dependent oxidoreductases | Alpha and beta proteins (a/b) | [GO:0004318  "enoyl-[acyl-carrier-protein] reductase (NADH) activity"  evidence=ISS] [GO:0030497 "fatty acid elongation" evidence=ISS]  [GO:0051289 "protein homotetramerization" evidence=ISS] |
| HP0196 | b.81.1.1 | Single-stranded left-handed beta-helix | Trimeric LpxA-like enzymes | UDP N-acetylglucosamine acyltransferase | All beta proteins | [GO:0009245 "lipid A  biosynthetic process" evidence=ISS] [GO:0016410 "N-acyltransferase  activity" evidence=ISS] |
| HP0197 | d.130.1.1 | S-adenosylmethionine synthetase | S-adenosylmethionine synthetase | S-adenosylmethionine synthetase | Alpha and beta proteins (a+b) | [GO:0004478  "methionine adenosyltransferase activity" evidence=ISS] [GO:0006555  "methionine metabolic process" evidence=ISS] [GO:0006556  "S-adenosylmethionine biosynthetic process" evidence=ISS] |
| HP0198 | d.58.6.1 | Ferredoxin-like | Nucleoside diphosphate kinase, NDK | Nucleoside diphosphate kinase, NDK | Alpha and beta proteins (a+b) | [GO:0004550  "nucleoside diphosphate kinase activity" evidence=ISS] [GO:0015949  "nucleobase-containing small molecule interconversion"  evidence=ISS] |
| HP0199 | e.3.1.1 | beta-lactamase/transpeptidase-like | beta-lactamase/transpeptidase-like | beta-Lactamase/D-ala carboxypeptidase | Multi-domain proteins (alpha and beta) | [GO:0003674  "molecular_function" evidence=ND] [GO:0005575 "cellular_component"  evidence=ND] [GO:0008150 "biological_process" evidence=ND] |
| HP0200 | g.41.8.5 | Rubredoxin-like | Zn-binding ribosomal proteins | Ribosomal protein L32p | Small proteins | [GO:0003735 "structural constituent  of ribosome" evidence=ISS] [GO:0006412 "translation" evidence=ISS] |
| HP0201 | c.77.1.4 | Isocitrate/Isopropylmalate dehydrogenase-like | Isocitrate/Isopropylmalate dehydrogenase-like | PlsX-like | Alpha and beta proteins (a/b) | [GO:0003824  "catalytic activity" evidence=ISS] [GO:0008610 "lipid biosynthetic  process" evidence=ISS] |
| HP0202 | c.95.1.2 | Thiolase-like | Thiolase-like | Chalcone synthase-like | Alpha and beta proteins (a/b) | [GO:0004315  "3-oxoacyl-[acyl-carrier-protein] synthase activity" evidence=ISS]  [GO:0006633 "fatty acid biosynthetic process" evidence=ISS] |
| HP0203 | d.92.1.9 | Zincin-like | Metalloproteases ("zincins"), catalytic domain | Reprolysin-like | Alpha and beta proteins (a+b) | [GO:0000398 "mRNA  splicing, via spliceosome" evidence=IC] [GO:0071011 "precatalytic  spliceosome" evidence=IDA] [GO:0071013 "catalytic step 2  spliceosome" evidence=IDA] |
| HP0204 | b.7.1.2 | C2 domain-like | C2 domain (Calcium/lipid-binding domain, CaLB) | Synaptotagmin-like (S variant) | All beta proteins |  |
| HP0205 | a.118.1.2 | alpha-alpha superhelix | ARM repeat | HEAT repeat | All alpha proteins | [GO:0003674  "molecular_function" evidence=ND] [GO:0008150 "biological_process"  evidence=ND] |
| HP0206 | c.37.1.12 | P-loop containing nucleoside triphosphate hydrolases | P-loop containing nucleoside triphosphate hydrolases | ABC transporter ATPase domain-like | Alpha and beta proteins (a/b) | [GO:0003697 "single-stranded DNA binding" evidence=ISS] [GO:0005524  "ATP binding" evidence=ISS] [GO:0006260 "DNA replication"  evidence=ISS] [GO:0006281 "DNA repair" evidence=ISS] |
| HP0207 | c.37.1.10 | P-loop containing nucleoside triphosphate hydrolases | P-loop containing nucleoside triphosphate hydrolases | Nitrogenase iron protein-like | Alpha and beta proteins (a/b) | [GO:0003674 "molecular_function"  evidence=ND] [GO:0008150 "biological_process" evidence=ND] |
| HP0209 | f.4.3.3 | Transmembrane beta-barrels | Porins | Ligand-gated protein channel | Membrane and cell surface proteins and peptides |  |
| HP0210 | d.14.1.8 | Ribosomal protein S5 domain 2-like | Ribosomal protein S5 domain 2-like | Hsp90 middle domain | Alpha and beta proteins (a+b) | [GO:0006457 "protein folding"  evidence=ISS] [GO:0009408 "response to heat" evidence=ISS]  [GO:0016887 "ATPase activity" evidence=ISS] |
| HP0211 | a.118.18.1 | alpha-alpha superhelix | HCP-like | HCP-like | All alpha proteins | [GO:0003674  "molecular_function" evidence=ND] [GO:0005575 "cellular_component"  evidence=ND] [GO:0008150 "biological_process" evidence=ND] |
| HP0212 | c.56.5.4 | Phosphorylase/hydrolase-like | Zn-dependent exopeptidases | Bacterial dinuclear zinc exopeptidases | Alpha and beta proteins (a/b) | [GO:0009014  "succinyl-diaminopimelate desuccinylase activity" evidence=ISS]  [GO:0009089 "lysine biosynthetic process via diaminopimelate"  evidence=ISS] |
| HP0213 | c.3.1.7 | FAD/NAD(P)-binding domain | FAD/NAD(P)-binding domain | GidA-like | Alpha and beta proteins (a/b) | [GO:0003674 "molecular_function" evidence=ND] [GO:0008150  "biological_process" evidence=ND] |
| HP0214 |  |  |  |  |  | [GO:0006820 "anion transport"  evidence=ISS] [GO:0008509 "anion transmembrane transporter  activity" evidence=ISS] |
| HP0215 | f.44.1.1 | Ammonium transporter | Ammonium transporter | Ammonium transporter | Membrane and cell surface proteins and peptides | [GO:0004605  "phosphatidate cytidylyltransferase activity" evidence=ISS]  [GO:0008654 "phospholipid biosynthetic process" evidence=ISS] |
| HP0216 | c.2.1.3 | NAD(P)-binding Rossmann-fold domains | NAD(P)-binding Rossmann-fold domains | Glyceraldehyde-3-phosphate dehydrogenase-like, N-terminal domain | Alpha and beta proteins (a/b) | [GO:0019288 "isopentenyl diphosphate biosynthetic process,  mevalonate-independent pathway" evidence=ISS] [GO:0030604  "1-deoxy-D-xylulose-5-phosphate reductoisomerase activity"  evidence=ISS] |
| HP0217 | c.68.1.17 | Nucleotide-diphospho-sugar transferases | Nucleotide-diphospho-sugar transferases | Polypeptide N-acetylgalactosaminyltransferase 1, N-terminal domain | Alpha and beta proteins (a/b) |  |
| HP0218 | b.17.1.2; | PEBP-like | PEBP-like | Prokaryotic PEBP-like proteins | All beta proteins | [GO:0005575 "cellular_component"  evidence=ND] [GO:0008150 "biological_process" evidence=ND] |
| HP0219 | a.4.1.19 | DNA/RNA-binding 3-helical bundle | Homeodomain-like | Cgl2762-like | All alpha proteins |  |
| HP0220 | c.67.1.3 | PLP-dependent transferase-like | PLP-dependent transferases | Cystathionine synthase-like | Alpha and beta proteins (a/b) | [GO:0009093 "cysteine  catabolic process" evidence=ISS] [GO:0016846 "carbon-sulfur lyase  activity" evidence=ISS] |
| HP0221 | d.224.1.2 | SufE/NifU | SufE/NifU | NifU/IscU domain | Alpha and beta proteins (a+b) | [GO:0003674  "molecular_function" evidence=ND] [GO:0008150 "biological_process"  evidence=ND] |
| HP0222 | a.93.1.1 | Heme-dependent peroxidases | Heme-dependent peroxidases | CCP-like | All alpha proteins | [GO:0005634 "nucleus" evidence=ISM;IDA]  [GO:0003700 "sequence-specific DNA binding transcription factor  activity" evidence=ISS] |
| HP0223 | c.37.1.11 | P-loop containing nucleoside triphosphate hydrolases | P-loop containing nucleoside triphosphate hydrolases | RecA protein-like (ATPase-domain) | Alpha and beta proteins (a/b) | [GO:0005524 "ATP binding"  evidence=ISS] [GO:0006281 "DNA repair" evidence=ISS] |
| HP0224 | b.88.1.3 | Mss4-like | Mss4-like | SelR domain | All beta proteins | [GO:0008113 "peptide-methionine (S)-S-oxide reductase  activity" evidence=ISS] [GO:0009405 "pathogenesis" evidence=ISS] |
| HP0225 | b.55.1.1 | PH domain-like barrel | PH domain-like | Pleckstrin-homology domain (PH domain) | All beta proteins |  |
| HP0226 | d.278.1.2 | Ligand-binding domain in the NO signalling and Golgi transport | Ligand-binding domain in the NO signalling and Golgi transport | TRAPP components | Alpha and beta proteins (a+b) | [GO:0003674 "molecular_function"  evidence=ND] [GO:0008150 "biological_process" evidence=ND] |
| HP0227 | f.4.1.1 | TRANSMEMBRANE BETA BAREELS | OMPA LIKE | OUTERMEMBRANE PROTEINS | Membrane and cell surface proteins and peptides | [GO:0000932 "cytoplasmic mRNA  processing body" evidence=IEA;IDA] [GO:0016787 "hydrolase activity"  evidence=IEA;IDA] |
| HP0228 | f.38.1.2 | MFS general substrate transporter | MFS general substrate transporter | LacY-like proton/sugar symporter | Membrane and cell surface proteins and peptides | [GO:0008272 "sulfate  transport" evidence=ISS] [GO:0015116 "sulfate transmembrane  transporter activity" evidence=ISS] |
| HP0229 | f.4.1.1 | TRANSMEMBRANE BETA BAREELS | OMPA LIKE | OUTERMEMBRANE PROTEINS | Membrane and cell surface proteins and peptides | [GO:0003674  "molecular_function" evidence=ND] [GO:0008150 "biological_process"  evidence=ND] |
| HP0230 | c.68.1.13 | Nucleotide-diphospho-sugar transferases | Nucleotide-diphospho-sugar transferases | Cytidylytransferase | Alpha and beta proteins (a/b) | [GO:0008690 "3-deoxy-manno-octulosonate cytidylyltransferase  activity" evidence=ISS] [GO:0009244 "lipopolysaccharide core region  biosynthetic process" evidence=ISS] |
| HP0231 | c.55.3.14 | Ribonuclease H-like motif | Ribonuclease H-like motif | Prp8 beta-finger domain-like | Alpha and beta proteins (a/b) | [GO:0005575 "cellular_component"  evidence=ND] [GO:0008150 "biological_process" evidence=ND] |
|  |  |  |  |  |  |  |
| HP0232 | a.118.1.1 | alpha-alpha superhelix | ARM repeat | Armadillo repeat | All alpha proteins | [GO:0003674 "molecular_function"  evidence=ND] [GO:0008150 "biological_process" evidence=ND] |
| HP0233 | d.3.1.15 | Cysteine proteinases | Cysteine proteinases | CHAP domain | Alpha and beta proteins (a+b) | [GO:0003674  "molecular_function" evidence=ND] [GO:0008150 "biological_process"  evidence=ND] |
| HP0234 | f.21.1.1 | Heme-binding four-helical bundle | Transmembrane di-heme cytochromes | Formate dehydrogenase N, cytochrome (gamma) subunit | Membrane and cell surface proteins and peptides | [GO:0003674  "molecular_function" evidence=ND] [GO:0005575 "cellular_component"  evidence=ND] [GO:0008150 "biological_process" evidence=ND] |
| HP0235 | a.118.18.1 | alpha-alpha superhelix | HCP-like | HCP-like | All alpha proteins | [GO:0003674  "molecular_function" evidence=ND] [GO:0005575 "cellular_component"  evidence=ND] [GO:0008150 "biological_process" evidence=ND] |
| HP0236 | a.3.1.1 | Cytochrome c | Cytochrome c | monodomain cytochrome c | All alpha proteins | [GO:0003674  "molecular_function" evidence=ND] [GO:0005575 "cellular_component"  evidence=ND] [GO:0008150 "biological_process" evidence=ND] |
| HP0237 | c.94.1.1 | Periplasmic binding protein-like II | Periplasmic binding protein-like II | Phosphate binding protein-like | Alpha and beta proteins (a/b) | [GO:0004418 "hydroxymethylbilane  synthase activity" evidence=ISS] [GO:0006779 "porphyrin-containing  compound biosynthetic process" evidence=ISS] |
| HP0238 | d.104.1.1 | Class II aaRS and biotin synthetases | Class II aaRS and biotin synthetases | Class II aminoacyl-tRNA synthetase (aaRS)-like, catalytic domain | Alpha and beta proteins (a+b) | [GO:0004827 "proline-tRNA ligase  activity" evidence=ISS] [GO:0006433 "prolyl-tRNA aminoacylation"  evidence=ISS] |
| HP0239 | c.2.1.7 | NAD(P)-binding Rossmann-fold domains | NAD(P)-binding Rossmann-fold domains | Aminoacid dehydrogenase-like, C-terminal domain | Alpha and beta proteins (a/b) | [GO:0006779 "porphyrin-containing  compound biosynthetic process" evidence=ISS] [GO:0008883  "glutamyl-tRNA reductase activity" evidence=ISS] |
| HP0240 | a.128.1.1 | Terpenoid synthases | Terpenoid synthases | Isoprenyl diphosphate synthases | All alpha proteins | [GO:0003824 "catalytic  activity" evidence=ISS] [GO:0008152 "metabolic process"  evidence=ISS] |
| HP0241 | b.1.11.1 | Immunoglobulin-like beta-sandwich | PapD-like | Pilus chaperone | All beta proteins | [GO:0003674 "molecular_function"  evidence=ND] [GO:0005575 "cellular_component" evidence=ND]  [GO:0008150 "biological_process" evidence=ND] |
| HP0242 | a.292.1.1 | HP0242-like | HP0242-like | HP0242-like | All alpha proteins |  |
| HP0243 | a.25.1.1 | Ferritin-like | Ferritin-like | Ferritin | All alpha proteins | [GO:0006880  "intracellular sequestering of iron ion" evidence=ISS] [GO:0008199  "ferric iron binding" evidence=ISS] |
| HP0244 | e.8.1.4 | DNA/RNA polymerases | DNA/RNA polymerases | RNA-dependent RNA-polymerase | Multi-domain proteins (alpha and beta) | [GO:0000155 "phosphorelay sensor  kinase activity" evidence=ISS] [GO:0000160 "phosphorelay signal  transduction system" evidence=ISS] |
| HP0245 | d.150.1.2 | phosphopantetheinyl transferase | phosphopantetheinyl transferase | Holo-(acyl carrier protein) synthase ACPS | Alpha and beta proteins (a+b) | [GO:0003674  "molecular_function" evidence=ND] [GO:0005575 "cellular_component"  evidence=ND] [GO:0008150 "biological_process" evidence=ND] |
|  |  |  |  |  |  |  |
| HP0246 | b.1.1.4 | immunoglobulin-like beta-sandwich | Immunoglobulin | set domains | All beta proteins | [GO:0001539 "ciliary  or flagellar motility" evidence=ISS] [GO:0005198 "structural  molecule activity" evidence=ISS] |
| HP0247 | c.37.1.19 | P-loop containing nucleoside triphosphate hydrolases | P-loop containing nucleoside triphosphate hydrolases | Tandem AAA-ATPase domain | Alpha and beta proteins (a/b) | [GO:0003723 "RNA  binding" evidence=ISS] [GO:0004004 "ATP-dependent RNA helicase  activity" evidence=ISS] [GO:0005524 "ATP binding" evidence=ISS]  [GO:0043590 "bacterial nucleoid" evidence=ISS] |
| HP0248 | f.5.1.1 | Outer membrane efflux proteins (OEP) | Outer membrane efflux proteins (OEP) | Outer membrane efflux proteins (OEP) | Membrane and cell surface proteins and peptides | [GO:0003674  "molecular_function" evidence=ND] [GO:0008150 "biological_process"  evidence=ND] |
| HP0249 | b.1.5.1 | Immunoglobulin-like beta-sandwich | Transglutaminase, two C-terminal domains | Transglutaminase, two C-terminal domains | All beta proteins |  |
| HP0250 |  |  |  |  |  | [GO:0006810 "transport" evidence=ISS] [GO:0042626 "ATPase activity,  coupled to transmembrane movement of substances" evidence=ISS] |
| HP0251 | e.70.1.1 | MalF N-terminal region-like | MalF N-terminal region-like | MalF N-terminal region-like | Multi-domain proteins (alpha and beta) | [GO:0015197 "peptide transporter activity" evidence=ISS]  [GO:0015833 "peptide transport" evidence=ISS] [GO:0042626 "ATPase  activity, coupled to transmembrane movement of substances"  evidence=ISS] |
| HP0252 | f.4.3.2 | Transmembrane beta-barrels | Porins | Maltoporin-like | Membrane and cell surface proteins and peptides |  |
| HP0253 | a.137.5.1 | Non-globular all-alpha subunits of globular proteins | Moesin tail domain | Moesin tail domain | All alpha proteins |  |
| HP0254 | f.4.1.1 | TRANSMEMBRANE BETA BAREELS | OMPA LIKE | OUTERMEMBRANE PROTEINS | Membrane and cell surface proteins and peptides |  |
| HP0255 | c.37.1.10 | P-loop containing nucleoside triphosphate hydrolases | P-loop containing nucleoside triphosphate hydrolases | Nitrogenase iron protein-like | Alpha and beta proteins (a/b) | [GO:0004019  "adenylosuccinate synthase activity" evidence=ISS] [GO:0009152  "purine ribonucleotide biosynthetic process" evidence=ISS] |
| HP0256 | b.110.1.1 | Cloacin translocation domain | Cloacin translocation domain | Cloacin translocation domain | All beta proteins | [GO:0000042 "protein targeting to Golgi"  evidence=IEA] [GO:0005634 "nucleus" evidence=IEA] [GO:0005794  "Golgi apparatus" evidence=IEA] |
| HP0257 | b.110.1.1 | Cloacin translocation domain | Cloacin translocation domain | Cloacin translocation domain | All beta proteins | [GO:0003674  "molecular_function" evidence=ND] [GO:0005575 "cellular_component"  evidence=ND] [GO:0008150 "biological_process" evidence=ND] |
| HP0258 | f.20.1.1 | Clc chloride channel | Clc chloride channel | Clc chloride channel | Membrane and cell surface proteins and peptides | [GO:0004222  "metalloendopeptidase activity" evidence=ISS] [GO:0006508  "proteolysis" evidence=ISS] |
|  |  |  |  |  |  |  |
| HP0259 | b.40.4.3; | OB-fold | Nucleic acid-binding proteins | Single strand DNA-binding domain, SSB | All beta proteins | [GO:0006308 "DNA  catabolic process" evidence=ISS] [GO:0008855 "exodeoxyribonuclease  VII activity" evidence=ISS] [GO:0009318 "exodeoxyribonuclease VII  complex" evidence=ISS] |
| HP0260 | c.66.1.11 | S-adenosyl-L-methionine-dependent methyltransferases | S-adenosyl-L-methionine-dependent methyltransferases | Type II DNA methylase | Alpha and beta proteins (a/b) | [GO:0006306 "DNA methylation" evidence=ISS] [GO:0009007  "site-specific DNA-methyltransferase (adenine-specific) activity"  evidence=ISS] [GO:0009307 "DNA restriction-modification system"  evidence=ISS] |
| HP0261 | d.17.4.20 | Cystatin-like | NTF2-like | Rpa4348-like | Alpha and beta proteins (a and b) | [GO:0005634  "nucleus" evidence=IEA] [GO:0005829 "cytosol" evidence=IEA]  [GO:0006457 "protein folding" evidence=IEA] |
| HP0262 | c.52.1.22 | Restriction endonuclease-like | Restriction endonuclease-like | Type II restriction endonuclease catalytic domain | Alpha and beta proteins (a/b) | [GO:0003700 "sequence-specific DNA binding transcription factor  activity" evidence=ISS] [GO:0006355 "regulation of transcription,  DNA-dependent" evidence=ISS] |
| HP0263 | c.66.1.11 | S-adenosyl-L-methionine-dependent methyltransferases | S-adenosyl-L-methionine-dependent methyltransferases | Type II DNA methylase | Alpha and beta proteins (a/b) | [GO:0008170 "N-methyltransferase activity"  evidence=IEA] [GO:0003677 "DNA binding" evidence=IEA] |
| HP0264 | a.174.1.1 | Double Clp-N motif | Double Clp-N motif | Double Clp-N motif | All alpha proteins | [GO:0016887 "ATPase  activity" evidence=ISS] [GO:0042026 "protein refolding"  evidence=ISS] |
| HP0265 | d.42.1.2 | POZ domain | POZ domain | Tetramerization domain of potassium channels | Alpha and beta proteins (a+b) | [GO:0003674  "molecular_function" evidence=ND] [GO:0017004 "cytochrome complex  assembly" evidence=ISS] |
| HP0266 | c.1.9.6 | TIM beta/alpha-barrel | Metallo-dependent hydrolases | Hydantoinase (dihydropyrimidinase), catalytic domain | Alpha and beta proteins (a/b) | [GO:0004151 "dihydroorotase activity"  evidence=ISS] [GO:0009220 "pyrimidine ribonucleotide biosynthetic  process" evidence=ISS] |
| HP0267 | c.1.9.16 | TIM beta/alpha-barrel | Metallo-dependent hydrolases | DR0824-like | Alpha and beta proteins (a/b) | [GO:0008152 "metabolic  process" evidence=ISS] [GO:0016810 "hydrolase activity, acting on  carbon-nitrogen (but not peptide) bonds" evidence=ISS] |
| HP0268 | c.1.11.2 | TIM beta/alpha-barrel | Enolase C-terminal domain-like | D-glucarate dehydratase-like | Alpha and beta proteins (a/b) | [GO:0003674  "molecular_function" evidence=ND] [GO:0005575 "cellular_component"  evidence=ND] [GO:0008150 "biological_process" evidence=ND] |
| HP0269 | c.1.28.2 | TIM beta/alpha-barrel | Radical SAM enzymes | Oxygen-independent coproporphyrinogen III oxidase HemN | Alpha and beta proteins (a/b) | [GO:0006400 "tRNA  modification" evidence=ISS] [GO:0016782 "transferase activity,  transferring sulfur-containing groups" evidence=ISS] |
| HP0270 | c.112.1.1 | Glycerol-3-phosphate (1)-acyltransferase | Glycerol-3-phosphate (1)-acyltransferase | Glycerol-3-phosphate (1)-acyltransferase | Alpha and beta proteins (a/b) | [GO:0003674  "molecular_function" evidence=ND] [GO:0005575 "cellular_component"  evidence=ND] [GO:0008150 "biological_process" evidence=ND] |
| HP0271 | c.55.1.1 | Ribonuclease H-like motif | Actin-like ATPase domain | Actin/HSP70 | Alpha and beta proteins (a/b) | [GO:0005737 "cytoplasm"  evidence=ISM] [GO:0005874 "microtubule" evidence=ISS] [GO:0007010  "cytoskeleton organization" evidence=TAS] |
|  |  |  |  |  |  |  |
| HP0272 | d.58.14.1 | Ferredoxin-like | Ribosomal protein S6 | Ribosomal protein S6 | Alpha and beta proteins (a+b) | [GO:0003674  "molecular_function" evidence=ND] [GO:0005575 "cellular_component"  evidence=ND] [GO:0008150 "biological_process" evidence=ND] |
| HP0273 | d.58.14.1 | Ferredoxin-like | Ribosomal protein S6 | Ribosomal protein S6 | Alpha and beta proteins (a+b) | [GO:0055114  "oxidation-reduction process" evidence=IEA] [GO:0016491  "oxidoreductase activity" evidence=IEA] |
| HP0274 | b.60.1.1 | Lipocalins | Lipocalins | Retinol binding protein-like | All beta proteins | [GO:0003674  "molecular_function" evidence=ND] [GO:0005575 "cellular_component"  evidence=ND] [GO:0008150 "biological_process" evidence=ND] |
| HP0275 | a.118.8.1 | alpha-alpha superhelix | TPR-like | Tetratricopeptide repeat (TPR) | All alpha proteins | [GO:0003674 "molecular_function"  evidence=ND] [GO:0008150 "biological_process" evidence=ND] |
| HP0276 | c.1.2.4 | TIM beta/alpha-barrel | Ribulose-phoshate binding barrel | Tryptophan biosynthesis enzymes | Alpha and beta proteins (a/b) | [GO:0000162  "tryptophan biosynthetic process" evidence=ISS] [GO:0004425  "indole-3-glycerol-phosphate synthase activity" evidence=ISS] |
| HP0277 | d.58.1.1 | Ferredoxin-like | 4Fe-4S ferredoxins | Short-chain ferredoxins | Alpha and beta proteins (a+b) | [GO:0009055 "electron carrier  activity" evidence=ISS] |
| HP0278 | a.211.1.5 | HD-domain/PDEase-like | HD-domain/PDEase-like | Ppx associated domain | All alpha proteins | [GO:0008152 "metabolic  process" evidence=ISS] [GO:0016791 "phosphatase activity"  evidence=ISS] |
| HP0279 | c.87.1.7 | UDP-Glycosyltransferase/glycogen phosphorylase | UDP-Glycosyltransferase/glycogen phosphorylase | ADP-heptose LPS heptosyltransferase II | Alpha and beta proteins (a/b) | [GO:0008920  "lipopolysaccharide heptosyltransferase activity" evidence=ISS]  [GO:0009244 "lipopolysaccharide core region biosynthetic process"  evidence=ISS] |
| HP280 | c.112.1.1 | Glycerol-3-phosphate (1)-acyltransferase | Glycerol-3-phosphate (1)-acyltransferase | Glycerol-3-phosphate (1)-acyltransferase | Alpha and beta proteins (a/b) | [GO:0009245 "lipid A biosynthetic process" evidence=ISS] |
| HP0281 | c.1.20.1 | TIM beta/alpha-barrel | tRNA-guanine transglycosylase | tRNA-guanine transglycosylase | Alpha and beta proteins (a/b) | [GO:0006400 "tRNA  modification" evidence=ISS] [GO:0008479 "queuine  tRNA-ribosyltransferase activity" evidence=ISS] |
| HP0282 | c.2.1.9 | NAD(P)-binding Rossmann-fold domains | NAD(P)-binding Rossmann-fold domains | Potassium channel NAD-binding domain | Alpha and beta proteins (a/b) | [GO:0003674 "molecular_function"  evidence=ND] [GO:0008150 "biological_process" evidence=ND] |
| HP0283 | e.22.1.1 | Dehydroquinate synthase-like | Dehydroquinate synthase-like | Dehydroquinate synthase, DHQS | Multi-domain proteins (alpha and beta) | [GO:0003856 "3-dehydroquinate  synthase activity" evidence=ISS] [GO:0009423 "chorismate  biosynthetic process" evidence=ISS] |
| HP0284 | a.238.1.1 | BAR/IMD domain-like | BAR/IMD domain-like | BAR domain | All alpha proteins | [GO:0005216  "ion channel activity" evidence=ISS] [GO:0006811 "ion transport"  evidence=ISS] [GO:0016020 "membrane" evidence=ISS] |
| HP0285 | c.1.28.1 | TIM beta/alpha-barrel | Radical SAM enzymes | Biotin synthase | Alpha and beta proteins (a/b) | [GO:0003824 "catalytic  activity" evidence=ISS] [GO:0006400 "tRNA modification"  evidence=ISS] |
|  |  |  |  |  |  |  |
| HP0286 |  |  |  |  |  | [GO:0000910  "cytokinesis" evidence=ISS] [GO:0004222 "metalloendopeptidase  activity" evidence=ISS] [GO:0016887 "ATPase activity" evidence=ISS] |
| HP0287 | c.69.1.10 | alpha/beta-Hydrolases | alpha/beta-Hydrolases | Carbon-carbon bond hydrolase | Alpha and beta proteins (a/b) | [GO:0000166  "nucleotide binding" evidence=IEA] [GO:0005525 "GTP binding"  evidence=IEA] [GO:0005785 "signal recognition particle receptor  complex" evidence=IEA] |
| HP0288 | d.2.1.3 | Lysozyme-like | Lysozyme-like | Phage lysozyme | Alpha and beta proteins (a+b) | [GO:0003674 "molecular_function"  evidence=ND] [GO:0008150 "biological_process" evidence=ND] |
| HP0289 |  |  |  |  |  |  |
| HP0289 | b.18.1.10 | Galactose-binding domain-like | Galactose-binding domain-like | Family 6 carbohydrate binding module, CBM6 | All beta proteins | [GO:0003676 "nucleic acid binding" evidence=IEA]  [GO:0000166 "nucleotide binding" evidence=IEA] |
| HP0290 | c.1.6.1 | TIM beta/alpha-barrel | PLP-binding barrel | Alanine racemase-like, N-terminal domain | Alpha and beta proteins (a/b) | [GO:0008836  "diaminopimelate decarboxylase activity" evidence=ISS] [GO:0009089  "lysine biosynthetic process via diaminopimelate" evidence=ISS] |
| HP0291 | a.130.1.1 | Chorismate mutase II | Chorismate mutase II | Dimeric chorismate mutase | All alpha proteins | [GO:0004106  "chorismate mutase activity" evidence=ISS] [GO:0004664 "prephenate  dehydratase activity" evidence=ISS] [GO:0009095 "aromatic amino  acid family biosynthetic process, prephenate pathway" evidence=ISS] |
| HP0292 | d.108.1.4 | Acyl-CoA N-acyltransferases (Nat  ) | Acyl-CoA N-acyltransferases (Nat) | FemXAB nonribosomal peptidyltransferases | Alpha and beta proteins (a+b) | [GO:0003674 "molecular_function"  evidence=ND] [GO:0005575 "cellular_component" evidence=ND]  [GO:0008150 "biological_process" evidence=ND] |
| HP0293 | d.161.1.1 | ADC synthase | ADC synthase | ADC synthase | Alpha and beta proteins (a+b) | [GO:0006760 "folic acid-containing compound metabolic  process" evidence=ISS] [GO:0046820 "4-amino-4-deoxychorismate  synthase activity" evidence=ISS] |
| HP0294 | d.160.1.1 | Carbon-nitrogen hydrolase | Carbon-nitrogen hydrolase | Nitrilase | Alpha and beta proteins (a+b) | [GO:0008152  "metabolic process" evidence=ISS] [GO:0016810 "hydrolase activity,  acting on carbon-nitrogen (but not peptide) bonds" evidence=ISS] |
| HP0295 | e.32.1.1 | Phase 1 flagellin | Phase 1 flagellin | Phase 1 flagellin | Multi-domain proteins (alpha and beta) | [GO:0005515 "protein binding" evidence=IPI] |
| HP0296 | b.30.5.2 | Supersandwich | Galactose mutarotase-like | Hyaluronate lyase-like, central domain | All beta proteins | [GO:0003735 "structural  constituent of ribosome" evidence=ISS] [GO:0006412 "translation"  evidence=ISS] |
| HP0297 |  | Ribosome and ribosomal fragments | Ribosome and ribosomal fragments | Large subunit | Low resolution protein structures | [GO:0003735 "structural constituent  of ribosome" evidence=ISS] [GO:0042254 "ribosome biogenesis"  evidence=ISS] |
| HP0298 | c.94.1.1 | Periplasmic binding protein-like II | Periplasmic binding protein-like II | Phosphate binding protein-like | Alpha and beta proteins (a/b) | [GO:0015440 "peptide-transporting ATPase  activity" evidence=ISS] [GO:0015833 "peptide transport"  evidence=ISS] [GO:0043190 "ATP-binding cassette (ABC) transporter  complex" evidence=ISS] |
| HP0299 | f.58.1.1 | MetI-like | MetI-like | MetI-like | Membrane and cell surface proteins and peptides | [GO:0005887 "integral to plasma membrane" evidence=ISS]  [GO:0015440 "peptide-transporting ATPase activity" evidence=ISS]  [GO:0015833 "peptide transport" evidence=ISS] |
| HP0300 | f.58.1.1 | MetI-like | MetI-like | MetI-like | Membrane and cell surface proteins and peptides | [GO:0015031 "protein  transport" evidence=IEA] [GO:0016020 "membrane" evidence=IEA]  [GO:0005886 "plasma membrane" evidence=IEA] [GO:0015421  "oligopeptide-transporting ATPase activity" evidence=ISS] |
| HP0301 | c.37.1.12 | P-loop containing nucleoside triphosphate hydrolases | P-loop containing nucleoside triphosphate hydrolases | ABC transporter ATPase domain-like | Alpha and beta proteins (a/b) | [GO:0005524 "ATP binding" evidence=ISS]  [GO:0009898 "internal side of plasma membrane" evidence=ISS]  [GO:0015440 "peptide-transporting ATPase activity" evidence=ISS] |
| HP0302 | c.37.1.12 | P-loop containing nucleoside triphosphate hydrolases | P-loop containing nucleoside triphosphate hydrolases | ABC transporter ATPase domain-like | Alpha and beta proteins (a/b) | [GO:0017111  "nucleoside-triphosphatase activity" evidence=IEA] [GO:0016887  "ATPase activity" evidence=IEA] [GO:0015886 "heme transport"  evidence=IMP] |
| HP0303 | b.117.1.1 | Obg-fold | Obg GTP-binding protein N-terminal domain | Obg GTP-binding protein N-terminal domain | All beta proteins | [GO:0005525 "GTP  binding" evidence=ISS] [GO:0008152 "metabolic process"  evidence=ISS] |
| HP0304 | a.102.3.1 | alpha/alpha toroid | Chondroitin AC/alginate lyase | Alginate lyase A1-III | All alpha proteins |  |
| HP0305 | b.61.6.1 | Streptavidin-like | YceI-lik | YceI-like | All beta proteins | [GO:0003674  "molecular_function" evidence=ND] [GO:0005575 "cellular_component"  evidence=ND] [GO:0008150 "biological_process" evidence=ND] |
| HP0306 | c.67.1.4 | Alpha and beta proteins (a/b) | PLP-dependent transferase-like | PLP-dependent transferase | GABA-aminotransferase-like | [GO:0006779  "porphyrin-containing compound biosynthetic process" evidence=ISS]  [GO:0042286 "glutamate-1-semialdehyde 2,1-aminomutase activity"  evidence=ISS] |
| HP0307 | f.51.1.1 | Rhomboid-like | Rhomboid-like | Rhomboid-like | Membrane and cell surface proteins and peptides | [GO:0003674 "molecular_function"  evidence=ND] [GO:0008150 "biological_process" evidence=ND] |
| HP0308 | b.55.1.2 | PH domain-like barrel | PH domain-like | Phosphotyrosine-binding domain (PTB) | All beta proteins |  |
| HP0309 | d.160.1.2 | Carbon-nitrogen hydrolase | Carbon-nitrogen hydrolase | Carbamilase | Alpha and beta proteins (a+b) | [GO:0016810 "hydrolase  activity, acting on carbon-nitrogen (but not peptide) bonds"  evidence=IEA] |
| HP0310 | c.6.2.6 | stranded beta/alpha barrel | Glycoside hydrolase/deacetylase | PA1517-like | Alpha and beta proteins (a/b) | [GO:0005575  "cellular_component" evidence=ND] |
| HP0311 | d.113.1.1 | Nudix | Nudix | MutT-like | Alpha and beta proteins (a+b) | [GO:0003674  "molecular_function" evidence=ND] [GO:0005575 "cellular_component"  evidence=ND] [GO:0008150 "biological_process" evidence=ND] |
| HP0312 | c.37.1.10 | P-loop containing nucleoside triphosphate hydrolases | P-loop containing nucleoside triphosphate hydrolases | Nitrogenase iron protein-like | Alpha and beta proteins (a/b) | [GO:0009507  "chloroplast" evidence=IDA] |
| HP0313 | f.38.1.1 | MFS general substrate transporter | MFS general substrate transporter | Glycerol-3-phosphate transporter | Membrane and cell surface proteins and peptides | [GO:0005215  "transporter activity" evidence=ISS] [GO:0006810 "transport"  evidence=ISS] |
| HP0314 | d.20.1.1 | UBC-like | UBC-like | UBC-related | Alpha and beta proteins (a+b) |  |
| HP0315 | d.79.4.1 | Bacillus chorismate mutase-like | PurM N-terminal domain-like | PurM N-terminal domain-like | Alpha and beta proteins (a+b) | [GO:0003674 "molecular_function" evidence=ND] [GO:0005575  "cellular_component" evidence=ND] [GO:0008150 "biological_process"  evidence=ND] |
| HP0316 | a.6.1.3 | Putative DNA-binding domain | Putative DNA-binding domain | DNA-binding N-terminal domain of transcription activators | All alpha proteins | [GO:0005828 "kinetochore microtubule" evidence=IDA]  [GO:0005634 "nucleus" evidence=IDA] |
| HP0317 | f.4.1.1 | TRANSMEMBRANE BETA BAREELS | OMPA LIKE | OUTERMEMBRANE PROTEINS | Membrane and cell surface proteins and peptides | GO:0005618 "cell wall"  evidence=IDA] [GO:0005829 "cytosol" evidence=IDA] [GO:0005886  "plasma membrane" evidence=IDA] |
| HP0318 | b.45.1.1 | Split barrel-like | FMN-binding split barrel | PNP-oxidase like | All beta proteins | [GO:0003674  "molecular_function" evidence=ND] [GO:0005575 "cellular_component"  evidence=ND] [GO:0008150 "biological_process" evidence=ND] |
| HP0319 | c.26.1.1 | Adenine nucleotide alpha hydrolase-like | Nucleotidylyl transferase | Class I aminoacyl-tRNA synthetases (RS), catalytic domain | Alpha and beta proteins (a/b) | [GO:0004814 "arginine-tRNA ligase  activity" evidence=ISS] [GO:0006420 "arginyl-tRNA aminoacylation"  evidence=ISS] |
| HP0320 | a.26.1.1 | 4-helical cytokines | 4-helical cytokines | Long-chain cytokines | All alpha proteins | [GO:0006886 "intracellular protein transport" evidence=ISS]  [GO:0008565 "protein transporter activity" evidence=ISS] |
| HP0321 | c.37.1.1 | P-loop containing nucleoside triphosphate hydrolases | P-loop containing nucleoside triphosphate hydrolases | Nucleotide and nucleoside kinases | Alpha and beta proteins (a/b) | [GO:0004385 "guanylate  kinase activity" evidence=ISS] [GO:0015949 "nucleobase-containing  small molecule interconversion" evidence=ISS] |
| HP0322 | b.68.1.1 | 6-bladed beta-propeller | Sialidases | Sialidases (neuraminidases) | All beta proteins | [GO:0016021  "integral to membrane" evidence=IEA] [GO:0005102 "receptor binding"  evidence=IEA] [GO:0016020 "membrane" evidence=IEA] [GO:0008150  "biological_process" evidence=ND] |
| HP0323 | d.136.1.1 | Phospholipase D/nuclease | Phospholipase D/nuclease | Nuclease | Alpha and beta proteins (a+b) | [GO:0003824 "catalytic activity" evidence=IEA]  [GO:0005739 "mitochondrion" evidence=IEA] [GO:0005741  "mitochondrial outer membrane" evidence=ISO;IDA] [GO:0006629 "lipid  metabolic process" evidence=IEA] |
| HP0324 | f.4.3.2 | Transmembrane beta-barrels | Porins | Maltoporin-like | Membrane and cell surface proteins and peptides |  |
| HP0325 | c.37.1.1 | P-loop containing nucleoside triphosphate hydrolases | P-loop containing nucleoside triphosphate hydrolases | Nucleotide and nucleoside kinases | Alpha and beta proteins (a/b) | [GO:0001539 "ciliary  or flagellar motility" evidence=ISS] [GO:0005198 "structural  molecule activity" evidence=ISS] |
| HP0326 | c.68.1.13 | Nucleotide-diphospho-sugar transferases | Nucleotide-diphospho-sugar transferases | Cytidylytransferase | Alpha and beta proteins (a/b) | [GO:0008781 "N-acylneuraminate cytidylyltransferase activity"  evidence=ISS] [GO:0045227 "capsule polysaccharide biosynthetic  process" evidence=ISS] |
| HP0327 | d.108.1.1 | Acyl-CoA N-acyltransferases (Nat) | Acyl-CoA N-acyltransferases (Nat) | N-acetyl transferase, NAT | Alpha and beta proteins (a+b) | [GO:0008080  "N-acetyltransferase activity" evidence=ISS] [GO:0008152 "metabolic  process" evidence=ISS] |
| HP0328 | c.56.5.5 | Phosphorylase/hydrolase-like | Zn-dependent exopeptidases | FolH catalytic domain-like | Alpha and beta proteins (a/b) | [GO:0009029  "tetraacyldisaccharide 4'-kinase activity" evidence=ISS]  [GO:0009245 "lipid A biosynthetic process" evidence=ISS] |
| HP0329 | c.26.2.1 | Adenine nucleotide alpha hydrolase-like | Adenine nucleotide alpha hydrolase-like | N-type ATP pyrophosphatases | Alpha and beta proteins (a/b) | [GO:0003952 "NAD+ synthase  (glutamine-hydrolyzing) activity" evidence=ISS] [GO:0009435 "NAD  biosynthetic process" evidence=ISS] |
| HP0330 | c.2.1.6 | NAD(P)-binding Rossmann-fold domains | NAD(P)-binding Rossmann-fold domains | phosphogluconate dehydrogenase-like, N-terminal domain | Alpha and beta proteins (a/b) | [GO:0004455  "ketol-acid reductoisomerase activity" evidence=ISS] [GO:0009082  "branched-chain amino acid biosynthetic process" evidence=ISS] |
| HP0331 | c.37.1.10 | P-loop containing nucleoside triphosphate hydrolases | P-loop containing nucleoside triphosphate hydrolases | Nitrogenase iron protein-like | Alpha and beta proteins (a/b) | [GO:0000910  "cytokinesis" evidence=ISS] [GO:0004857 "enzyme inhibitor activity"  evidence=ISS] [GO:0043086 "negative regulation of catalytic  activity" evidence=ISS] |
| HP0332 | d.79.4.1 | Bacillus chorismate mutase-like | PurM N-terminal domain-like | PurM N-terminal domain-like | Alpha and beta proteins (a+b) | [GO:0003674  "molecular_function" evidence=ND] |
| HP0333 | c.67.1.9 | PLP-dependent transferase-like | PLP-dependent transferase-like | SepSecS-like | Alpha and beta proteins (a/b) | [GO:0003674 "molecular_function"  evidence=ND] |
| HP0334 | c.55.3.8 | Ribonuclease H-like motif | Ribonuclease H-like | Putative Holliday junction resolvase RuvX | Alpha and beta proteins (a/b) | [GO:0003674  "molecular_function" evidence=ND] [GO:0005575 "cellular_component"  evidence=ND] [GO:0008150 "biological_process" evidence=ND] |
| HP0335 | a.118.18.1 | alpha-alpha superhelix | HCP-like | HCP-like | All alpha proteins |  |
| HP0336 | a.118.18.1 | alpha-alpha superhelix | HCP-like | HCP-like | All alpha proteins | [GO:0003674  "molecular_function" evidence=ND] [GO:0005575 "cellular_component"  evidence=ND] [GO:0008150 "biological_process" evidence=ND] |
| HP0337 | a.118.8.1 | alpha-alpha superhelix | TPR-like | Tetratricopeptide repeat (TPR) | All alpha proteins | [GO:0003779 "actin binding"  evidence=IEA] [GO:0005737 "cytoplasm" evidence=IEA] [GO:0005856  "cytoskeleton" evidence=IEA] |
| HP0338 | c.3.1.4 | FAD/NAD(P)-binding domain | FAD/NAD(P)-binding domain | Succinate dehydrogenase/fumarate reductase flavoprotein N-terminal domain | Alpha and beta proteins (a/b) |  |
| HP0339 | d.2.1.3 | Lysozyme-like | Lysozyme-like | Phage lysozyme | Alpha and beta proteins (a+b) | [GO:0003796 "lysozyme activity"  evidence=ISS] [GO:0009253 "peptidoglycan catabolic process"  evidence=ISS] [GO:0019835 "cytolysis" evidence=ISS] |
| HP0340 | c.23.16.2 | Flavodoxin-like | Class I glutamine amidotransferase-like | DJ-1/PfpI | Alpha and beta proteins (a/b) | [GO:0016568 "chromatin modification"  evidence=IEA] [GO:0004402 "histone acetyltransferase activity"  evidence=IEA] [GO:0016573 "histone acetylation" evidence=IEA] |
| HP0341 | c.67.1.3 | PLP-dependent transferase-like | PLP-dependent transferase-like | Cystathionine synthase-like | Alpha and beta proteins (a/b) |  |
| HP0342 |  |  |  |  |  | [GO:0003674 "molecular_function" evidence=ND]  [GO:0008150 "biological_process" evidence=ND] |
| HP0343 | g.53.1.1 | TAZ domain | TAZ domain | TAZ domain | Small proteins |  |
| HP0344 | a.4.5.14 | DNA/RNA-binding 3-helical bundle | Winged helix" DNA-binding domain | Forkhead DNA-binding domain | All alpha proteins | [GO:0003674 "molecular_function" evidence=ND] [GO:0005575  "cellular_component" evidence=ND] [GO:0008150 "biological_process"  evidence=ND] |
| HP0345 | f.54.1.1 | SNF-like | SNF-like | SNF-like | Membrane and cell surface proteins and peptides |  |
| HP0346 | a.24.1.1 | Four-helical up-and-down bundle | Apolipoprotein | Apolipoprotein | All alpha proteins | [GO:0003674 "molecular_function" evidence=ND] [GO:0005575  "cellular_component" evidence=ND] [GO:0008150 "biological_process"  evidence=ND] |
| HP0347 | d.265.1.3 | Pseudouridine synthase | Pseudouridine synthase | Pseudouridine synthase RsuA/RluD | Alpha and beta proteins (a+b) | [GO:0009451  "RNA modification" evidence=ISS] [GO:0009982 "pseudouridine  synthase activity" evidence=ISS] |
| HP0348 | d.265.1.3 | Pseudouridine synthase | Pseudouridine synthase | Pseudouridine synthase RsuA/RluD | Alpha and beta proteins (a+b) | [GO:0006310 "DNA  recombination" evidence=ISS] [GO:0008297 "single-stranded DNA  specific exodeoxyribonuclease activity" evidence=ISS] |
| HP0349 | c.107.1.2 | DHH phosphoesterases | DHH phosphoesterases | Exonuclease RecJ | Alpha and beta proteins (a/b) | [GO:0003883 "CTP synthase activity"  evidence=ISS] [GO:0009220 "pyrimidine ribonucleotide biosynthetic  process" evidence=ISS] |
| HP0350 | c.37.1.10 | P-loop containing nucleoside triphosphate hydrolases | P-loop containing nucleoside triphosphate hydrolases | Nitrogenase iron protein-like | Alpha and beta proteins (a/b) | [GO:0003674  "molecular_function" evidence=ND] [GO:0008150 "biological_process"  evidence=ND] |
|  | a.111.1.1 | Acid phosphatase/Vanadium-dependent haloperoxidase | Acid phosphatase/Vanadium-dependent haloperoxidase | Type 2 phosphatidic acid phosphatase, PAP2 | All alpha proteins |  |
| HP0351 | d.266.1.1 | Hypothetical protein MTH677 | Hypothetical protein MTH677 | Hypothetical protein MTH677 | Alpha and beta proteins (a+b) | [GO:0001539 "ciliary  or flagellar motility" evidence=ISS] [GO:0005198 "structural  molecule activity" evidence=ISS] [GO:0009431 "bacterial-type  flagellum basal body, MS ring" evidence=ISS] |
| HP0352 | a.118.8.2 | alpha-alpha superhelix | TPR-like | Transcription factor MalT domain III | All alpha proteins | [GO:0009288  "bacterial-type flagellum" evidence=IMP] [GO:0071978  "bacterial-type flagellar swarming motility" evidence=IMP] |
| HP0353 | a.29.16.1 | Bromodomain-like | IVS-encoded protein-like | IVS-encoded protein-like | All alpha proteins | [GO:0005515 "protein binding"  evidence=IPI] |
| HP0354 | c.36.1.10 | Thiamin diphosphate-binding fold (THDP-binding) | Thiamin diphosphate-binding fold (THDP-binding) | TK-like PP module | Alpha and beta proteins (a/b) | [GO:0008299  "isoprenoid biosynthetic process" evidence=ISS] [GO:0008615  "pyridoxine biosynthetic process" evidence=ISS] [GO:0008661  "1-deoxy-D-xylulose-5-phosphate synthase activity" evidence=ISS] |
| HP0355 | c.37.1.8 | P-loop containing nucleoside triphosphate hydrolases | P-loop containing nucleoside triphosphate hydrolases | G proteins | Alpha and beta proteins (a/b) | [GO:0005525 "GTP binding"  evidence=ISS] [GO:0008152 "metabolic process" evidence=ISS] |
| HP0356 | d.82.5.1 | N domain of copper amine oxidase-like | GK1464-like | GK1464-like | Alpha and beta proteins (a+b) |  |
| HP0357 | c.2.1.2 | NAD(P)-binding Rossmann-fold domains | NAD(P)-binding Rossmann-fold domains | Tyrosine-dependent oxidoreductases | Alpha and beta proteins (a/b) | [GO:0000166 "nucleotide  binding" evidence=IEA] [GO:0035527 "3-hydroxypropionate  dehydrogenase (NADP+) activity" evidence=IEA;IDA] [GO:0006212  "uracil catabolic process" evidence=IMP] [GO:0055114  "oxidation-reduction process" evidence=IEA] |
| HP0358 | d.83.1.1 | Aha1/BPI domain-like | Bactericidal permeability-increasing protein, BPI | Bactericidal permeability-increasing protein, BPI | Alpha and beta proteins (a+b) | [GO:0003674  "molecular_function" evidence=ND] [GO:0005575 "cellular_component"  evidence=ND] [GO:0008150 "biological_process" evidence=ND] |
| HP0359 | b.40.4.3 | OB-fold | Nucleic acid-binding proteins | Single strand DNA-binding domain, SSB | All beta proteins |  |
| HP0360 | c.2.1.2 | NAD(P)-binding Rossmann-fold domains | NAD(P)-binding Rossmann-fold domains | Tyrosine-dependent oxidoreductases | Alpha and beta proteins (a/b) | [GO:0003978 "UDP-glucose 4-epimerase  activity" evidence=ISS] [GO:0006012 "galactose metabolic process"  evidence=ISS] |
| HP0361 | d.265.1.1 | Pseudouridine synthase | Pseudouridine synthase | Pseudouridine synthase | Alpha and beta proteins (a+b) | [GO:0006400 "tRNA  modification" evidence=ISS] [GO:0009982 "pseudouridine synthase  activity" evidence=ISS] |
| HP0362 |  |  |  |  |  | [GO:0003674 "molecular_function"  evidence=ND] [GO:0008150 "biological_process" evidence=ND] |
| HP0363 | c.66.1.7 | S-adenosyl-L-methionine-dependent methyltransferases | S-adenosyl-L-methionine-dependent methyltransferases | Protein-L-isoaspartyl O-methyltransferase | Alpha and beta proteins (a/b) | [GO:0004719 "protein-L-isoaspartate (D-aspartate)  O-methyltransferase activity" evidence=ISS] [GO:0030091 "protein  repair" evidence=ISS] |
| HP0364 | a.25.1.2 | All alpha proteins | Ferritin-like | Ferritin-like | Ribonucleotide reductase-like | [GO:0004748 "ribonucleoside-diphosphate reductase activity,  thioredoxin disulfide as acceptor" evidence=ISS] [GO:0009265  "2'-deoxyribonucleotide biosynthetic process" evidence=ISS] |
| HP0365 | a.116.1.2 | GTPase activation domain, GAP | GTPase activation domain, GAP | p120GAP domain-like | All alpha proteins |  |
| HP0366 | c.67.1.4 | PLP-dependent transferase-like | PLP-dependent transferases | GABA-aminotransferase-like | Alpha and beta proteins (a/b) | [GO:0008152  "metabolic process" evidence=ISS] [GO:0008483 "transaminase  activity" evidence=ISS] |
| HP0367 | a.25.1.2 | Ferritin-like | Ferritin-like | Ribonucleotide reductase-like | All alpha proteins | [GO:0000075 "cell cycle  checkpoint" evidence=ISS] [GO:0000776 "kinetochore" evidence=ISS]  [GO:0003682 "chromatin binding" evidence=ISS] [GO:0005634 "nucleus"  evidence=ISS] [GO:0007126 "meiosis" evidence=ISS] |
| HP0368 | b.7.1.2 | C2 domain-like | C2 domain (Calcium/lipid-binding domain, CaLB) | Synaptotagmin-like (S variant) | All beta proteins |  |
| HP0369 | c.66.1.27 | S-adenosyl-L-methionine-dependent methyltransferases | S-adenosyl-L-methionine-dependent methyltransferases | DNA methylase TaqI, N-terminal domain | Alpha and beta proteins (a/b) |  |
| HP0370 | d.142.1.2 | ATP-grasp | Glutathione synthetase ATP-binding domain-like | BC ATP-binding domain-like | Alpha and beta proteins (a+b) | [GO:0003989 "acetyl-CoA carboxylase activity" evidence=ISS]  [GO:0006633 "fatty acid biosynthetic process" evidence=ISS] |
| HP0371 | d.142.1.2 | ATP-grasp | Glutathione synthetase ATP-binding domain-like | BC ATP-binding domain-like | Alpha and beta proteins (a+b) | [GO:0003989 "acetyl-CoA carboxylase activity" evidence=ISS]  [GO:0006633 "fatty acid biosynthetic process" evidence=ISS]  [GO:0009317 "acetyl-CoA carboxylase complex" evidence=ISS] |
| HP0372 | b.85.4.1 | beta-clip | dUTPase-like | dUTPase-like | All beta proteins | [GO:0008829  "dCTP deaminase activity" evidence=ISS] [GO:0009394  "2'-deoxyribonucleotide metabolic process" evidence=ISS] |
| HP0373 | f.4.3.2 | Transmembrane beta-barrels | Porins | Maltoporin-like | Membrane and cell surface proteins and peptides |  |
| HP0374 | c.116.1.5 | alpha/beta knot | alpha/beta knot | YggJ C-terminal domain-like | Alpha and beta proteins (a/b) | [GO:0003674 "molecular_function" evidence=ND] [GO:0005575  "cellular_component" evidence=ND] [GO:0008150 "biological_process"  evidence=ND] |
| HP0375 | d.2.1.3 | Lysozyme-like | Lysozyme-like | Phage lysozyme | Alpha and beta proteins (a+b) |  |
| HP0376 | c.92.1.1 | Chelatase-like | Chelatase-like | Ferrochelatase | Alpha and beta proteins (a/b) | [GO:0004325 "ferrochelatase activity"  evidence=ISS] [GO:0006779 "porphyrin-containing compound  biosynthetic process" evidence=ISS] |
| HP0377 | c.47.1.1 | Thioredoxin fold | Thioredoxin like | Thioltransferase | Alpha and beta proteins (a/b) | [GO:0019417 "sulfur oxidation" evidence=ISS]  [GO:0030288 "outer membrane-bounded periplasmic space"  evidence=ISS] |
| HP0378 | d.15.6.1 | beta-Grasp (ubiquitin-like) | Superantigen toxins, C-terminal domain | Superantigen toxins, C-terminal domain | Alpha and beta proteins (a+b) | [GO:0003674 "molecular_function"  evidence=ND] [GO:0016021 "integral to membrane" evidence=NAS]  [GO:0017004 "cytochrome complex assembly" evidence=NAS] |
| HP0379 | c.87.1.11 | UDP-Glycosyltransferase/glycogen phosphorylase | UDP-Glycosyltransferase/glycogen phosphorylase | FucT-like | Alpha and beta proteins (a/b) | [GO:0036065 "fucosylation"  evidence=IEA] [GO:0016020 "membrane" evidence=IEA] [GO:0008417  "fucosyltransferase activity" evidence=IEA] [GO:0006486 "protein  glycosylation" evidence=IEA] [GO:0016757 "transferase activity,  transferring glycosyl groups" evidence=IEA] |
| HP0380 | c.58.1.1 | Aminoacid dehydrogenase-like, N-terminal domain | Aminoacid dehydrogenase-like, N-terminal domain | Aminoacid dehydrogenase | Alpha and beta proteins (a/b) | [GO:0004354 "glutamate  dehydrogenase (NADP+) activity" evidence=ISS] [GO:0005737  "cytoplasm" evidence=ISS] [GO:0006537 "glutamate biosynthetic  process" evidence=ISS] |
| HP0381 | c.66.1.30 | S-adenosyl-L-methionine-dependent methyltransferases | S-adenosyl-L-methionine-dependent methyltransferases | N5-glutamine methyltransferase, HemK | Alpha and beta proteins (a/b) | [GO:0008152 "metabolic  process" evidence=ISS] [GO:0008170 "N-methyltransferase activity"  evidence=ISS] |
| HP0382 |  |  |  |  |  | [GO:0004222 "metalloendopeptidase  activity" evidence=ISS] [GO:0006508 "proteolysis" evidence=ISS]  [GO:0016020 "membrane" evidence=ISS] |
| HP0383 | a.24.8.1 | Four-helical up-and-down bundle | Proteasome activator | Proteasome activator | All alpha proteins |  |
| HP0384 | d.58.52.1 | Ferredoxin-like | Sporulation related repeat | Sporulation related repeat | Alpha and beta proteins (a+b) |  |
| HP0385 | a.215.1.1 | A middle domain of Talin 1 | A middle domain of Talin 1 | A middle domain of Talin 1 | All alpha proteins | [GO:0003674  "molecular_function" evidence=ND] [GO:0005575 "cellular_component"  evidence=ND] [GO:0008150 "biological_process" evidence=ND] |
| HP0386 | d.18.1.2 | ssDNA-binding transcriptional regulator domain | ssDNA-binding transcriptional regulator domain | Plant transcriptional regulator PBF-2 | Alpha and beta proteins (a+b) | [GO:0005575 "cellular_component" evidence=ND] [GO:0008150  "biological_process" evidence=ND] [GO:0003674 "molecular_function"  evidence=ND] |
| HP0387 | b.44.1.1 | Elongation factor/aminomethyltransferase common domain | EF-Tu/eEF-1alpha/eIF2-gamma C-terminal domain | EF-Tu/eEF-1alpha/eIF2-gamma C-terminal domain | All beta proteins | [GO:0003678 "DNA helicase activity"  evidence=ISS] [GO:0003688 "DNA replication origin binding"  evidence=ISS] [GO:0005658 "alpha DNA polymerase:primase complex"  evidence=ISS] [GO:0006260 "DNA replication" evidence=ISS] |
| HP0388 | c.66.1.14 | S-adenosyl-L-methionine-dependent methyltransferases | S-adenosyl-L-methionine-dependent methyltransferases | Hypothetical protein HI0319 (YecO) | Alpha and beta proteins (a/b) | [GO:0008152 "metabolic  process" evidence=ISS] [GO:0008168 "methyltransferase activity"  evidence=ISS] |
| HP0389 | d.44.1.1 | Fe,Mn superoxide dismutase (SOD), C-terminal domain | Fe,Mn superoxide dismutase (SOD), C-terminal domain | Fe,Mn superoxide dismutase (SOD), C-terminal domain | Alpha and beta proteins (a+b) | [GO:0019430 "removal of superoxide  radicals" evidence=ISS] |
| HP0390 | c.47.1.10 | Thioredoxin fold | Thioredoxin fold | Glutathione peroxidase-like | Alpha and beta proteins (a/b) | [GO:0006979 "response to oxidative  stress" evidence=ISS] [GO:0008379 "thioredoxin peroxidase activity"  evidence=ISS] |
| HP0391 | b.40.7.1 | OB-fold | CheW-like | CheW-like | All beta proteins | [GO:0005515 "protein binding" evidence=IPI] |
| HP0393 | c.23.1.1 | Flavodoxin-like | CheY-like | CheY-related | All alpha and beta proteins(a/b) | [GO:0005515 "protein binding" evidence=IPI] |
| HP0392 |  |  |  |  |  | [GO:0005515 "protein binding"  evidence=IPI] |
| HP0394 | d.159.1.7 | Metallo-dependent phosphatases | Metallo-dependent phosphatases | YfcE-like | Alpha and beta proteins (a+b) | [GO:0003674  "molecular_function" evidence=ND] [GO:0005575 "cellular_component"  evidence=ND] [GO:0008150 "biological_process" evidence=ND] |
| HP0395 | c.1.6.2 | TIM beta/alpha-barrel | PLP-binding barrel | Hypothetical" protein ybl036c | Alpha and beta proteins (a/b) | [GO:0003674  "molecular_function" evidence=ND] [GO:0005575 "cellular_component"  evidence=ND] [GO:0008150 "biological_process" evidence=ND] |
| HP0396 | b.45.1.3 | Split barrel-like | FMN-binding split barrel | UbiD middle domain-like | All beta proteins | [GO:0006744 "ubiquinone biosynthetic process" evidence=ISS]  [GO:0008694 "3-octaprenyl-4-hydroxybenzoate carboxy-lyase activity"  evidence=ISS] |
| HP0397 | c.2.1.4 | NAD(P)-binding Rossmann-fold domains | NAD(P)-binding Rossmann-fold domains | Formate/glycerate dehydrogenases, NAD-domain | Alpha and beta proteins (a/b) | [GO:0004617  "phosphoglycerate dehydrogenase activity" evidence=ISS] [GO:0006564  "L-serine biosynthetic process" evidence=ISS] |
| HP0398 | d.58.3.1 | Ferredoxin-like | Protease propeptides/inhibitors | Pancreatic carboxypeptidase, activation domain | Alpha and beta proteins (a+b) | [GO:0044351  "macropinocytosis" evidence=RCA] |
| HP0399 |  |  |  |  |  | [GO:0003735 "structural constituent  of ribosome" evidence=ISS] [GO:0006412 "translation" evidence=ISS] |
| HP0400 | c.93.1.1 | Periplasmic binding protein-like I | Periplasmic binding protein-like I | L-arabinose binding protein-like | Alpha and beta proteins (a/b) | [GO:0015968  "stringent response" evidence=ISS] [GO:0042380  "hydroxymethylbutenyl pyrophosphate reductase activity"  evidence=ISS] [GO:0046677 "response to antibiotic" evidence=ISS] |
| HP0401 |  |  |  |  |  | [GO:0003866 "3-phosphoshikimate 1-carboxyvinyltransferase  activity" evidence=ISS] [GO:0009423 "chorismate biosynthetic  process" evidence=ISS] |
| HP0402 | b.153.1.1 | PheT/TilS domain | PheT/TilS domain | B3/B4 domain of PheRS, PheT | All beta proteins | [GO:0004826  "phenylalanine-tRNA ligase activity" evidence=ISS] [GO:0006432  "phenylalanyl-tRNA aminoacylation" evidence=ISS] |
| HP0403 | d.104.1.1 | Class II aaRS and biotin synthetases | Class II aaRS and biotin synthetases | Class II aminoacyl-tRNA synthetase (aaRS)-like, catalytic domain | Alpha and beta proteins (a+b) | [GO:0004826  "phenylalanine-tRNA ligase activity" evidence=ISS] [GO:0006432  "phenylalanyl-tRNA aminoacylation" evidence=ISS] |
| HP0404 | d.13.1.1 | HIT-like | HIT-like | HIT (HINT, histidine triad) family of protein kinase-interacting proteins | Alpha and beta proteins (a+b) | [GO:0003674 "molecular_function"  evidence=ND] [GO:0008150 "biological_process" evidence=ND] |
| HP0405 | c.67.1.3 | PLP-dependent transferase-like | PLP-dependent transferase-like | Cystathionine synthase-like | Alpha and beta proteins (a/b) | [GO:0008152 "metabolic  process" evidence=ISS] [GO:0008483 "transaminase activity"  evidence=ISS] |
| HP0406 | a.114.1.1 | Interferon-induced guanylate-binding protein 1 (GBP1), C-terminal domain | Interferon-induced guanylate-binding protein 1 (GBP1), C-terminal domain | Interferon-induced guanylate-binding protein 1 (GBP1), C-terminal domain | All alpha proteins | [GO:0003674 "molecular_function" evidence=ND] [GO:0005575  "cellular_component" evidence=ND] [GO:0008150 "biological_process"  evidence=ND] |
| HP0407 | c.81.1.1 | Formate dehydrogenase/DMSO reductase, domains 1-3 | Formate dehydrogenase/DMSO reductase, domains 1-3 | Formate dehydrogenase/DMSO reductase, domains 1-3 | Alpha and beta proteins (a/b) | [GO:0050626 "trimethylamine-N-oxide reductase (cytochrome c)  activity" evidence=IEA;IMP] [GO:0005515 "protein binding"  evidence=IPI] [GO:0016020 "membrane" evidence=IBA] |
| HP0408 | d.38.1.1 | Thioesterase/thiol ester dehydrase-isomerase | Thioesterase/thiol ester dehydrase-isomerase | 4HBT-like | Alpha and beta proteins (a+b) | [GO:0048015  "phosphatidylinositol-mediated signaling" evidence=IEA] [GO:0046872  "metal ion binding" evidence=IEA] |
| HP0409 | c.23.16.1 | Flavodoxin-like | Class I glutamine amidotransferase-like | Class I glutamine amidotransferase-(GAT) | Alpha and beta proteins (a/b) | [GO:0003922 "GMP synthase  (glutamine-hydrolyzing) activity" evidence=ISS] [GO:0009152 "purine  ribonucleotide biosynthetic process" evidence=ISS] |
| HP0410 | c.51.6.1 | Anticodon-binding domain-like | XCC0632-like | NLBH-like | Alpha and beta proteins (a/b) | [GO:0005634  "nucleus" evidence=IEA] [GO:0019898 "extrinsic to membrane"  evidence=IEA] [GO:0005737 "cytoplasm" evidence=IEA] |
| HP0411 | b.62.1.1 | Cyclophilin-like | Cyclophilin-like | Cyclophilin (peptidylprolyl isomerase) | All beta proteins | [GO:0016020 "membrane" evidence=IEA] [GO:0016628  "oxidoreductase activity, acting on the CH-CH group of donors, NAD  or NADP as acceptor" evidence=IEA] |
| HP0412 | a.119.1.1 | Lipoxigenase | Lipoxigenase | Plant lipoxigenases | All alpha proteins |  |
| HP0413 | d.166.1.6 | ADP-ribosylation | ADP-ribosylation | BC2332-like | Alpha and beta proteins (a+b) | [GO:0004803 "transposase activity"  evidence=ISS] [GO:0006313 "transposition, DNA-mediated"  evidence=ISS] |
| HP0414 | d.58.57.1 | Ferredoxin-like | Transposase IS200-like | Transposase IS200-like | Alpha and beta proteins (a+b) | [GO:0004803 "transposase  activity" evidence=ISS] [GO:0006313 "transposition, DNA-mediated"  evidence=ISS] |
| HP0415 | d.58.43.1 | Ferredoxin-like | Mechanosensitive channel protein MscS (YggB), C-terminal domain | Mechanosensitive channel protein MscS (YggB), C-terminal domain | Alpha and beta proteins (a+b) | [GO:0005216  "ion channel activity" evidence=ISS] [GO:0006811 "ion transport"  evidence=ISS] [GO:0016020 "membrane" evidence=ISS] |
| HP0416 | c.66.1.18 | S-adenosyl-L-methionine-dependent methyltransferases | S-adenosyl-L-methionine-dependent methyltransferases | Mycolic acid cyclopropane synthase | Alpha and beta proteins (a/b) | [GO:0008610 "lipid biosynthetic process" evidence=ISS] [GO:0008825  "cyclopropane-fatty-acyl-phospholipid synthase activity"  evidence=ISS] |
| HP0417 | c.26.1.1 | Adenine nucleotide alpha hydrolase-like | Nucleotidylyl transferase | Class I aminoacyl-tRNA synthetases (RS), catalytic domain | Alpha and beta proteins (a/b) | [GO:0004825 "methionine-tRNA ligase  activity" evidence=ISS] [GO:0006431 "methionyl-tRNA aminoacylation"  evidence=ISS] |
| HP0418 | c.72.2.1 | Ribokinase-like | MurD-like peptide ligases, catalytic domain | MurCDEF | Alpha and beta proteins (a/b) | [GO:0003674  "molecular_function" evidence=ND] [GO:0005575 "cellular_component"  evidence=ND] [GO:0008150 "biological_process" evidence=ND] |
| HP0419 | c.66.1.34 | S-adenosyl-L-methionine-dependent methyltransferases | S-adenosyl-L-methionine-dependent methyltransferases | mRNA cap (Guanine N-7) methyltransferase | Alpha and beta proteins (a/b) | [GO:0008152 "metabolic  process" evidence=ISS] [GO:0008168 "methyltransferase activity"  evidence=ISS] |
| HP0420 | d.38.1.5 | Thioesterase/thiol ester dehydrase-isomerase | Thioesterase/thiol ester dehydrase-isomerase | PaaI/YdiI-like | Alpha and beta proteins (a+b) | [GO:0003674  "molecular_function" evidence=ND] [GO:0005575 "cellular_component"  evidence=ND] [GO:0008150 "biological_process" evidence=ND] |
| HP0421 | c.87.1.8 | UDP-Glycosyltransferase/glycogen phosphorylase | UDP-Glycosyltransferase/glycogen phosphorylase | Glycosyl transferases group 1 | Alpha and beta proteins (a/b) | [GO:0000271 "polysaccharide biosynthetic process" evidence=ISS]  [GO:0016757 "transferase activity, transferring glycosyl groups"  evidence=ISS] |
| HP0422 | c.1.6.1 | TIM beta/alpha-barrel | PLP-binding barrel | Alanine racemase-like, N-terminal domain | Alpha and beta proteins (a/b) | [GO:0006596 "polyamine biosynthetic  process" evidence=ISS] [GO:0008792 "arginine decarboxylase  activity" evidence=ISS] |
| HP0423 | b.47.1.1 | Trypsin-like serine proteases | Trypsin-like serine proteases | Prokaryotic proteases | All beta proteins |  |
| HP0424 | a.118.8.1 | alpha-alpha superhelix | TPR-like | Tetratricopeptide repeat (TPR) | All alpha proteins | [GO:0005824 "outer plaque of spindle pole body" evidence=IDA]  [GO:0000070 "mitotic sister chromatid segregation" evidence=IMP] |
| HP0425 | c.107.1.2 | DHH phosphoesterases | DHH phosphoesterases | Exonuclease RecJ | Alpha and beta proteins (a/b) | [GO:0006310  "DNA recombination" evidence=ISS] [GO:0008297 "single-stranded DNA  specific exodeoxyribonuclease activity" evidence=ISS] |
| HP0426 | d.268.1.2 | ParB/Sulfiredoxin | ParB/Sulfiredoxin | Hypothetical protein PF0380 | Alpha and beta proteins (a+b) | [GO:0003674  "molecular_function" evidence=ND] [GO:0005575 "cellular_component"  evidence=ND] [GO:0008150 "biological_process" evidence=ND] |
| HP0427 | b.1.5.1 | Immunoglobulin-like beta-sandwich | Transglutaminase, two C-terminal domains | Transglutaminase, two C-terminal domains | All beta proteins | [GO:0003674  "molecular_function" evidence=ND] [GO:0008150 "biological_process"  evidence=ND] |
| HP0428 | c.62.1.1 | vWA-like | vWA-like | Integrin A (or I) domain | Alpha and beta proteins (a/b) | [GO:0003674 "molecular_function" evidence=ND] [GO:0008150  "biological_process" evidence=ND] |
| HP0430 | c.62.1.1 | vWA-like | vWA-like | Integrin A (or I) domain | Alpha and beta proteins (a/b) |  |
| HP0431 | d.219.1.1 | PP2C-like | PP2C-like | PP2C-like | Alpha and beta proteins (a+b) |  |
| HP0432 | d.144.1.7 | Protein kinase-like (PK-like) | Protein kinase-like (PK-like) | Protein kinases, catalytic subunit | Alpha and beta proteins (a+b) | [GO:0005524 "ATP binding" evidence=IEA] [GO:0007049  "cell cycle" evidence=IEA] [GO:0046872 "metal ion binding"  evidence=IEA] |
| HP0433 | b.40.4.15 | OB-fold | Nucleic acid-binding proteins | SSO2064-like | All beta proteins |  |
| HP0434 | a.25.1.2 | Ferritin-like | Ferritin-like | Ribonucleotide reductase-like | All alpha proteins | [GO:0003674 "molecular_function" evidence=ND] [GO:0005575  "cellular_component" evidence=ND] |
| HP0435 | c.37.1.19 | P-loop containing nucleoside triphosphate hydrolases | P-loop containing nucleoside triphosphate hydrolases | Tandem AAA-ATPase domain | Alpha and beta proteins (a/b) | [GO:0003677 "DNA binding" evidence=IEA]  [GO:0003724 "RNA helicase activity" evidence=ISS] [GO:0004386  "helicase activity" evidence=IEA] |
| HP0436 | a.24.10.4 | Four-helical up-and-down bundle | Histidine-containing phosphotransfer domain, HPT domain | Sensor-like histidine kinase YojN, C-terminal domain | All alpha proteins | [GO:0005730 "nucleolus" evidence=IEA]  [GO:0004526 "ribonuclease P activity" evidence=IEA] [GO:0008033  "tRNA processing" evidence=IEA] |
| HP0437 | d.58.57.1 | Ferredoxin-like | Transposase IS200-like | Transposase IS200-like | Alpha and beta proteins (a+b) | [GO:0004803 "transposase  activity" evidence=ISS] [GO:0006313 "transposition, DNA-mediated"  evidence=ISS] |
| HP0438 | b.113.1.1 | N-terminal domain of MutM-like DNA repair proteins | N-terminal domain of MutM-like DNA repair proteins | N-terminal domain of MutM-like DNA repair proteins | All beta proteins | [GO:0004803 "transposase activity"  evidence=ISS] [GO:0006313 "transposition, DNA-mediated"  evidence=ISS] |
| HP0439 | a.238.1.4 | BAR/IMD domain-like | BAR/IMD domain-like | FCH domain | All alpha proteins | [GO:0003674  "molecular_function" evidence=ND] [GO:0030255 "protein secretion by  the type IV secretion system" evidence=ISS] |
| HP0440 | e.10.1.1 | Prokaryotic type I DNA topoisomerase | Prokaryotic type I DNA topoisomerase | Prokaryotic type I DNA topoisomerase | Multi-domain proteins (alpha and beta) | [GO:0003677 "DNA binding"  evidence=ISS] [GO:0003917 "DNA topoisomerase type I activity"  evidence=ISS] [GO:0006260 "DNA replication" evidence=ISS] |
| HP0441 | c.37.1.11 | P-loop containing nucleoside triphosphate hydrolases | P-loop containing nucleoside triphosphate hydrolases | RecA protein-like (ATPase-domain) | Alpha and beta proteins (a/b) | [GO:0003674  "molecular_function" evidence=ND] [GO:0030255 "protein secretion by  the type IV secretion system" evidence=ISS] |
| HP0442 |  |  |  |  |  | [GO:0003674 "molecular_function"  evidence=ND] [GO:0008150 "biological_process" evidence=ND] |
| HP0443 | d.320.1.1 | YojJ-like | YojJ-like | YojJ-like | Alpha and beta proteins (a+b) |  |
| HP0444 | e.13.1.1 | DNA primase core | DNA primase core | DNA primase DnaG catalytic core | Multi-domain proteins (alpha and beta) | [GO:0005085 "guanyl-nucleotide exchange  factor activity" evidence=IBA] [GO:0005737 "cytoplasm"  evidence=IBA] |
| HP0445 | c.56.4.1 | Phosphorylase/hydrolase-like | Pyrrolidone carboxyl peptidase (pyroglutamate aminopeptidase) | Pyrrolidone carboxyl peptidase (pyroglutamate aminopeptidase) | Alpha and beta proteins (a/b) |  |
| HP0446 | b.82.3.2 | Double-stranded beta-helix | cAMP-binding domain-like | cAMP-binding domain-like | All beta proteins |  |
| HP0447 | c.26.2.1 | Adenine nucleotide alpha hydrolase-like | Adenine nucleotide alpha hydrolase-like | N-type ATP pyrophosphatases | Alpha and beta proteins (a/b) | [GO:0008150  "biological_process" evidence=ND] [GO:0005575 "cellular_component"  evidence=ND] [GO:0003674 "molecular_function" evidence=ND] |
| HP0448 | b.1.2.1 | Immunoglobulin-like beta-sandwich | Fibronectin type III | Fibronectin type III | All beta proteins |  |
| HP0449 | d.93.1.1 | SH2-like | SH2 domain | SH2 domain | Alpha and beta proteins (a+b) | [GO:0005881 "cytoplasmic microtubule"  evidence=IEA] [GO:0030473 "nuclear migration along microtubule"  evidence=IEA] |
| HP0450 | a.238.1.4 | BAR/IMD domain-like | BAR/IMD domain-like | FCH domain | All alpha proteins |  |
| HP0451 | b.30.6.1 | Supersandwich | V-region of surface antigen I/II (SA I/II, PAC) | V-region of surface antigen I/II (SA I/II, PAC) | All beta proteins | [GO:0046872 "metal ion binding"  evidence=IEA] |
| HP0452 | d.14.1.10 | Ribosomal protein S5 domain 2-like | Ribosomal protein S5 domain 2-like | ATP-dependent protease Lon (La), catalytic domain | Alpha and beta proteins (a+b) | [GO:0003674 "molecular_function" evidence=ND]  [GO:0005575 "cellular_component" evidence=ND] [GO:0008150  "biological_process" evidence=ND] |
| HP0453 | b.34.5.6 | SH3-like barrel | Translation proteins SH3-like domain | Ribosomal protein L19 | All beta proteins | [GO:0060041 "retina development in camera-type eye"  evidence=IEA] [GO:0005932 "microtubule basal body" evidence=IEA]  [GO:0005737 "cytoplasm" evidence=IDA] |
| HP0454 | c.60.1.1 | Phosphoglycerate mutase-like | Phosphoglycerate mutase-like | Cofactor-dependent phosphoglycerate mutase | Alpha and beta proteins (a/b) | [GO:0008150  "biological_process" evidence=ND] [GO:0003674 "molecular_function"  evidence=ND] [GO:0005575 "cellular_component" evidence=ND] |
| HP0455 | c.46.1.1 | Rhodanese/Cell cycle control phosphatase | Rhodanese/Cell cycle control phosphatase | Cell cycle control phosphatase, catalytic domain | Alpha and beta proteins (a/b) |  |
| HP0456 |  |  |  |  |  |  |
| HP0457 |  |  |  |  |  | [GO:0003674  "molecular_function" evidence=ND] [GO:0008150 "biological_process"  evidence=ND] |
| HP0458 |  |  |  |  |  | [GO:0005575 "cellular_component" evidence=ND] [GO:0003674  "molecular_function" evidence=ND] [GO:0008150 "biological_process"  evidence=ND] |
| HP0459 | c.37.1.11 | P-loop containing nucleoside triphosphate hydrolases | P-loop containing nucleoside triphosphate hydrolases | RecA protein-like (ATPase-domain) | Alpha and beta proteins (a/b) | [GO:0003674 "molecular_function" evidence=ND] |
| HP0460 | b.110.1.1 | Cloacin translocation domain | Cloacin translocation domain | Cloacin translocation domain | All beta proteins | [GO:0003674  "molecular_function" evidence=ND] [GO:0005575 "cellular_component"  evidence=ND] |
| HP0461 | c.37.1.12 | P-loop containing nucleoside triphosphate hydrolases | P-loop containing nucleoside triphosphate hydrolases | ABC transporter ATPase domain-like | Alpha and beta proteins (a/b) |  |
| HP0462 | d.287.1.2 | DNA methylase specificity domain | DNA methylase specificity domain | Type I restriction modification DNA specificity domain | Alpha and beta proteins (a+b) | [GO:0003677 "DNA binding" evidence=ISS]  [GO:0006304 "DNA modification" evidence=ISS] [GO:0009307 "DNA  restriction-modification system" evidence=ISS] |
| HP0463 | c.66.1.45 | S-adenosyl-L-methionine-dependent methyltransferases | S-adenosyl-L-methionine-dependent methyltransferases | N-6 DNA Methylase-like | Alpha and beta proteins (a/b) | [GO:0006304 "DNA modification" evidence=ISS] [GO:0009007  "site-specific DNA-methyltransferase (adenine-specific) activity"  evidence=ISS] |
| HP0464 | a.118.1.1 | alpha-alpha superhelix | ARM repeat | Armadillo repeat | All alpha proteins | [GO:0009035  "Type I site-specific deoxyribonuclease activity" evidence=ISS]  [GO:0009307 "DNA restriction-modification system" evidence=ISS] |
| HP0465 | c.130.1.1 | Alpha-2,3/8-sialyltransferase CstII | Alpha-2,3/8-sialyltransferase CstII | Alpha-2,3/8-sialyltransferase CstII | Alpha and beta proteins (a/b) | [GO:0001539 "ciliary or flagellar  motility" evidence=IGI] [GO:0003674 "molecular_function"  evidence=ND] |
| HP0466 | a.118.1.1 | alpha-alpha superhelix | ARM repeat | Armadillo repeat | All alpha proteins | [GO:0033674  "positive regulation of kinase activity" evidence=IEA] [GO:0031954  "positive regulation of protein autophosphorylation" evidence=IEA] |
| HP0467 |  |  |  |  |  | [GO:0006897  "endocytosis" evidence=IEA] [GO:0032947 "protein complex scaffold"  evidence=IEA] |
| HP0468 |  | FAD/NAD(P)-binding domain | FAD/NAD(P)-binding domain | FAD/NAD-linked reductases, N-terminal and central domains | Alpha and beta proteins (a/b) | [GO:0003674  "molecular_function" evidence=ND] [GO:0005575 "cellular_component"  evidence=ND] [GO:0008150 "biological_process" evidence=ND] |
| HP0469 | b.136.1.2 | SspB-like | SspB-like | AGR_C_3712p-like | All beta proteins | [GO:0003674  "molecular_function" evidence=ND] [GO:0005575 "cellular_component"  evidence=ND] [GO:0008150 "biological_process" evidence=ND] |
| HP0470 | a.118.1.1 | alpha-alpha superhelix | ARM repeat | Armadillo repeat | All alpha proteins | [GO:0004222 "metalloendopeptidase  activity" evidence=ISS] [GO:0006508 "proteolysis" evidence=ISS] |
| HP0471 | c.1.16.1 | TIM beta/alpha-barrel | Bacterial luciferase-like | Bacterial luciferase (alkanal monooxygenase) | Alpha and beta proteins (a/b) | [GO:0006814 "sodium  ion transport" evidence=ISS] [GO:0006818 "hydrogen transport"  evidence=ISS] |
| HP0472 | f.4.1.1 | Transmembrane beta-barrels | OMPA-like | Outer membrane protein | Membrane and cell surface proteins and hp0peptides | [GO:0008340  "determination of adult lifespan" evidence=IMP] [GO:0045087 "innate  immune response" evidence=IEP;IMP] |
| HP0473 | c.94.1.1 | Periplasmic binding protein-like II | Periplasmic binding protein-like II | Phosphate binding protein-like | Alpha and beta proteins (a/b) | [GO:0015412 "molybdate transmembrane-transporting ATPase  activity" evidence=ISS] [GO:0015689 "molybdate ion transport"  evidence=ISS] |
| HP0474 | f.58.1.1 | MetI-like | MetI-like | MetI-like | Membrane and cell surface proteins and peptides | [GO:0015412  "molybdate transmembrane-transporting ATPase activity"  evidence=ISS] [GO:0015689 "molybdate ion transport" evidence=ISS]  [GO:0016020 "membrane" evidence=ISS] |
| HP0475 | c.37.1.12 | P-loop containing nucleoside triphosphate hydrolases | P-loop containing nucleoside triphosphate hydrolases | ABC transporter ATPase domain-like | Alpha and beta proteins (a/b) | [GO:0015412  "molybdate transmembrane-transporting ATPase activity"  evidence=ISS] [GO:0015689 "molybdate ion transport" evidence=ISS] |
| HP0476 | c.26.1.1 | Adenine nucleotide alpha hydrolase-like | Nucleotidylyl transferase | Class I aminoacyl-tRNA synthetases (RS), catalytic domain | Alpha and beta proteins (a/b) | [GO:0004818 "glutamate-tRNA ligase  activity" evidence=ISS] [GO:0006424 "glutamyl-tRNA aminoacylation"  evidence=ISS] [GO:0009332 "glutamate-tRNA ligase complex"  evidence=ISS] |
| HP0477 | f.4.4.2 | Transmembrane beta-barrels | OMPT-like | Outer membrane adhesin/invasin OpcA | Membrane and cell surface proteins and peptides |  |
| HP0478 | c.66.1.45 | S-adenosyl-L-methionine-dependent methyltransferases | S-adenosyl-L-methionine-dependent methyltransferases | N-6 DNA Methylase-like | Alpha and beta proteins (a/b) | [GO:0006304 "DNA modification" evidence=ISS]  [GO:0009008 "DNA-methyltransferase activity" evidence=ISS] |
| HP0479 | c.87.1.7 | UDP-Glycosyltransferase/glycogen phosphorylase | UDP-Glycosyltransferase/glycogen phosphorylase | ADP-heptose LPS heptosyltransferase II | Alpha and beta proteins (a/b) |  |
| HP0480 | c.37.1.8 | P-loop containing nucleoside triphosphate hydrolases | P-loop containing nucleoside triphosphate hydrolases | G proteins | Alpha and beta proteins (a/b) | [GO:0005525 "GTP binding"  evidence=ISS] [GO:0008152 "metabolic process" evidence=ISS] |
| HP0481 | c.66.1.28 | S-adenosyl-L-methionine-dependent methyltransferases | S-adenosyl-L-methionine-dependent methyltransferases | N6 adenine-specific DNA methylase, DAM | Alpha and beta proteins (a/b) | [GO:0006281 "DNA repair"  evidence=ISS] [GO:0009007 "site-specific DNA-methyltransferase  (adenine-specific) activity" evidence=ISS] |
| HP0482 | d.50.1.1 | dsRBD-like | dsRNA-binding domain-like | Double-stranded RNA-binding domain (dsRBD) | Alpha and beta proteins (a+b) |  |
| HP0484 | c.68.1.22 | Nucleotide-diphospho-sugar transferases | Nucleotide-diphospho-sugar transferases | Glycosylating toxin catalytic domain-like | Alpha and beta proteins (a/b) |  |
| HP0485 | e.5.1.1 | Heme-dependent catalase-like | Heme-dependent catalase-like | Heme-dependent catalase | Multi-domain proteins (alpha and beta) | [GO:0004096 "catalase activity"  evidence=ISS] [GO:0006979 "response to oxidative stress"  evidence=ISS] |
| HP0486 | f.4.3.3 | Transmembrane beta-barrels | Porins | Ligand-gated protein channel | Membrane and cell surface proteins and peptides |  |
| HP0487 | f.4.3.3 | Transmembrane beta-barrels | Porins | Ligand-gated protein channel | Membrane and cell surface proteins and peptides |  |
| HP0488 | f.42.1.1 | Mitochondrial carrier | Mitochondrial carrier | Mitochondrial carrier | Membrane and cell surface proteins and peptides | [GO:0003674  "molecular_function" evidence=ND] [GO:0005575 "cellular_component"  evidence=ND] [GO:0008150 "biological_process" evidence=ND] |
| HP0489 | a.238.1.4 | BAR/IMD domain-like | BAR/IMD domain-like | FCH domain | All alpha proteins | [GO:0003674 "molecular_function"  evidence=ND] [GO:0016020 "membrane" evidence=IDA] |
| HP0490 | c.2.1.9 | NAD(P)-binding Rossmann-fold domains | NAD(P)-binding Rossmann-fold domains | Potassium channel NAD-binding domain | Alpha and beta proteins (a/b) | [GO:0042802 "identical protein binding" evidence=IPI] |
| HP0491 | d.325.1.1 | L28p-like | L28p-like | Ribosomal protein L28 | Alpha and beta proteins (a+b) | [GO:0003735 "structural constituent  of ribosome" evidence=ISS] [GO:0042254 "ribosome biogenesis"  evidence=ISS] |
| HP0492 | c.51.6.1 | Anticodon-binding domain-like | XCC0632-like | NLBH-like | Alpha and beta proteins (a/b) |  |
| HP0493 |  |  |  |  |  | [GO:0008963  "phospho-N-acetylmuramoyl-pentapeptide-transferase activity"  evidence=ISS] [GO:0009252 "peptidoglycan biosynthetic process"  evidence=ISS] [GO:0016020 "membrane" evidence=ISS] |
| HP0494 | c.72.2.1 | Ribokinase-like | MurD-like peptide ligases, catalytic domain | MurCDEF | Alpha and beta proteins (a/b) | [GO:0008764  "UDP-N-acetylmuramoylalanine-D-glutamate ligase activity"  evidence=ISS] [GO:0009252 "peptidoglycan biosynthetic process"  evidence=ISS] |
| HP0495 | d.58.54.2 | Ferredoxin-like | YbeD/HP0495-like | HP0495-like | Alpha and beta proteins (a+b) | [GO:0003674  "molecular_function" evidence=ND] [GO:0005575 "cellular_component"  evidence=ND] [GO:0008150 "biological_process" evidence=ND] |
| HP0496 | d.38.1.1 | Thioesterase/thiol ester dehydrase-isomerase | Thioesterase/thiol ester dehydrase-isomerase | 4HBT-like | Alpha and beta proteins (a+b) | [GO:0003824 "catalytic  activity" evidence=ISS] [GO:0008152 "metabolic process"  evidence=ISS] |
| HP0497 | f.54.1.1 | SNF-like | SNF-like | SNF-like | Membrane and cell surface proteins and peptides | [GO:0006814 "sodium  ion transport" evidence=ISS] [GO:0015081 "sodium ion transmembrane  transporter activity" evidence=ISS] |
| HP0498 | f.54.1.1 | SNF-like | SNF-like | SNF-like | Membrane and cell surface proteins and peptides | [GO:0006814 "sodium  ion transport" evidence=ISS] [GO:0015081 "sodium ion transmembrane  transporter activity" evidence=ISS] |
| HP0499 | f.4.2.1 | Transmembrane beta-barrels | Outer membrane phospholipase A (OMPLA) | Outer membrane phospholipase A (OMPLA) | Membrane and cell surface proteins and peptides | [GO:0031230  "intrinsic to cell outer membrane" evidence=IDA] [GO:0008970  "phosphatidylcholine 1-acylhydrolase activity" evidence=IEA;TAS] |
| HP0500 | d.131.1.1 | DNA clamp | DNA clamp | DNA polymerase III, beta subunit | Alpha and beta proteins (a+b) | [GO:0003887  "DNA-directed DNA polymerase activity" evidence=ISS] [GO:0006260  "DNA replication" evidence=ISS] [GO:0009360 "DNA polymerase III  complex" evidence=ISS] |
| HP0501 | d.122.1.2 | ATPase domain of HSP90 chaperone/DNA topoisomerase II/histidine kinase | ATPase domain of HSP90 chaperone/DNA topoisomerase II/histidine kinase | DNA gyrase/MutL, N-terminal domain | Alpha and beta proteins (a+b) | [GO:0003918 "DNA topoisomerase type  II (ATP-hydrolyzing) activity" evidence=ISS] [GO:0006265 "DNA  topological change" evidence=ISS] [GO:0009330 "DNA topoisomerase  complex (ATP-hydrolyzing)" evidence=ISS] |
| HP0502 | c.66.1.45 | S-adenosyl-L-methionine-dependent methyltransferases | S-adenosyl-L-methionine-dependent methyltransferases | N-6 DNA Methylase-like | Alpha and beta proteins (a/b) |  |
| HP0503 | c.66.1.27 | S-adenosyl-L-methionine-dependent methyltransferases | S-adenosyl-L-methionine-dependent methyltransferases | DNA methylase TaqI, N-terminal domain | Alpha and beta proteins (a/b) |  |
| HP0504 | d.129.3.5 | TBP-like | Bet v1-like | AHSA1 domain | Alpha and beta proteins (a+b) | [GO:0004149  "dihydrolipoyllysine-residue succinyltransferase activity"  evidence=ISS] [GO:0006099 "tricarboxylic acid cycle" evidence=ISS] |
| HP0505 | d.129.3.5 | TBP-like | Bet v1-like | AHSA1 domain | Alpha and beta proteins (a+b) |  |
| HP0506 | b.84.3.2 | Barrel-sandwich hybrid | Duplicated hybrid motif | Peptidoglycan hydrolase LytM | All beta proteins | [GO:0004222 "metalloendopeptidase  activity" evidence=ISS] [GO:0006508 "proteolysis" evidence=ISS] |
| HP0507 | d.113.1.1 | Nudix | Nudix | MutT-like | Alpha and beta proteins (a+b) | [GO:0016787  "hydrolase activity" evidence=IEA] [GO:0016818 "hydrolase activity,  acting on acid anhydrides, in phosphorus-containing anhydrides"  evidence=IEA] |
| HP0508 | f.5.1.1 | Outer membrane efflux proteins (OEP) | Outer membrane efflux proteins (OEP) | Outer membrane efflux proteins (OEP) | Membrane and cell surface proteins and peptides | [GO:0005575 "cellular_component"  evidence=ND] [GO:0003674 "molecular_function" evidence=ND] |
| HP0509 | d.145.1.1 | FAD-binding/transporter-associated domain-like | FAD-binding/transporter-associated domain-like | FAD-linked oxidases, N-terminal domain | Alpha and beta proteins (a+b) | [GO:0008891 "glycolate  oxidase activity" evidence=ISS] [GO:0015976 "carbon utilization"  evidence=ISS] |
| HP0510 | d.81.1.3 | FwdE/GAPDH domain-like | Glyceraldehyde-3-phosphate dehydrogenase-like, C-terminal domain | Dihydrodipicolinate reductase-like | Alpha and beta proteins (a+b) | [GO:0008839  "4-hydroxy-tetrahydrodipicolinate reductase" evidence=ISS]  [GO:0009089 "lysine biosynthetic process via diaminopimelate"  evidence=ISS] |
| HP0511 | c.45.1.2 | (Phosphotyrosine protein) phosphatases II | (Phosphotyrosine protein) phosphatases II | Higher-molecular-weight phosphotyrosine protein phosphatases | Alpha and beta proteins (a/b) |  |
| HP0512 | d.128.1.1 | Glutamine synthetase/guanido kinase | Glutamine synthetase/guanido kinase | automated matches | Alpha and beta proteins (a+b) | [GO:0004356  "glutamate-ammonia ligase activity" evidence=ISS] [GO:0006542  "glutamine biosynthetic process" evidence=ISS] |
| HP0513 | d.198.4.1 | Secretion chaperone-like | YdhG-like | YdhG-like | Alpha and beta proteins (a+b) | [GO:0003674  "molecular_function" evidence=ND] [GO:0005575 "cellular_component"  evidence=ND] [GO:0008150 "biological_process" evidence=ND] |
| HP0514 | d.99.1.1 | Ribosomal protein L9 C-domain | Ribosomal protein L9 C-domain | Ribosomal protein L9 C-domain | Alpha and beta proteins (a+b) | [GO:0003735 "structural constituent  of ribosome" evidence=ISS] [GO:0042254 "ribosome biogenesis"  evidence=ISS] |
| HP0515 | d.153.1.4 | Ntn hydrolase-like | N-terminal nucleophile aminohydrolases (Ntn hydrolases) | Proteasome subunits | Alpha and beta proteins (a+b) | [GO:0005515 "protein  binding" evidence=IPI] |
| HP0516 | c.37.1.20 | P-loop containing nucleoside triphosphate hydrolases | P-loop containing nucleoside triphosphate hydrolases | Extended AAA-ATPase domain | Alpha and beta proteins (a/b) | [GO:0016887  "ATPase activity" evidence=ISS] |
| HP0517 | d.52.3.1 | Alpha-lytic protease prodomain-like | Prokaryotic type KH domain (KH-domain type II) | Prokaryotic type KH domain (KH-domain type II) | Alpha and beta proteins (a+b) | [GO:0005525 "GTP binding"  evidence=ISS] [GO:0016049 "cell growth" evidence=ISS] |
| HP0518 | b.160.1.1 | L,D-transpeptidase catalytic domain-like | L,D-transpeptidase catalytic domain-like | L,D-transpeptidase catalytic domain-like | All beta proteins | [GO:0003674  "molecular_function" evidence=ND] [GO:0005575 "cellular_component"  evidence=ND] [GO:0008150 "biological_process" evidence=ND] |
| HP0519 | a.118.18.1 | alpha-alpha superhelix | HCP-like | HCP-like | All alpha proteins | [GO:0003674 "molecular_function"  evidence=ND] [GO:0008150 "biological_process" evidence=ND] |
| HP0520 | a.40.1.1 | CH domain-like | Calponin-homology domain, CH-domain | Calponin-homology domain, CH-domain | All alpha proteins |  |
| HP0522 | c.23.1.1 | Flavodoxin-like | CheY-like | CheY-like | Alpha and beta proteins (a/b) | [GO:0005515 "protein  binding" evidence=IPI] |
| HP0523 | d.2.1.2 | Lysozyme-like | Lysozyme-like | C-type lysozyme | Alpha and beta proteins (a+b) | [GO:0005622  "intracellular" evidence=IEA] [GO:0005543 "phospholipid binding"  evidence=IEA] |
| HP0524 | c.37.1.11 | P-loop containing nucleoside triphosphate hydrolases | P-loop containing nucleoside triphosphate hydrolases | RecA protein-like (ATPase-domain) | Alpha and beta proteins (a/b) | [GO:0003674  "molecular_function" evidence=ND] [GO:0030255 "protein secretion by  the type IV secretion system" evidence=TAS] |
| HP0525 | c.37.1.11 | P-loop containing nucleoside triphosphate hydrolases | P-loop containing nucleoside triphosphate hydrolases | RecA protein-like (ATPase-domain) | Alpha and beta proteins (a/b) | [GO:0005515 "protein binding" evidence=IPI] |
| HP0526 | a.47.3.1 | STAT-like | Cag-Z | Cag-Z | All alpha proteins |  |
| HP0527 | f.42.1.1 | Mitochondrial carrier | Mitochondrial carrier | Mitochondrial carrier | Membrane and cell surface proteins and peptides | [GO:0005515 "protein binding" evidence=IPI] |
| HP0528 | f.5.1.1 | Outer membrane efflux proteins (OEP) | Outer membrane efflux proteins (OEP) | Outer membrane efflux proteins (OEP) | Membrane and cell surface proteins and peptides | [GO:0005515 "protein binding" evidence=IPI] |
| HP0529 | a.4.1.3 | DNA/RNA-binding 3-helical bundle | Homeodomain-like | Myb/SANT domain | All alpha proteins |  |
| HP0530 | d.17.4.26 | Cystatin-like | NTF2-like | VirB8-like | Alpha and beta proteins (a+b) |  |
| HP0531 | d.90.1.1 | FMN-dependent nitroreductase-like | FMN-dependent nitroreductase-like | NADH oxidase/flavin reductase | Alpha and beta proteins (a+b) |  |
| HP0532 | d.19.1.1 | MHC antigen-recognition domain | MHC antigen-recognition domain | MHC antigen-recognition domain | Alpha and beta proteins (a+b) | [GO:0005515 "protein  binding" evidence=IPI] |
| HP0534 | a.25.1.2 | Ferritin-like | Ferritin-like | Ribonucleotide reductase-like | All alpha proteins | [GO:0005634 "nucleus" evidence=IEA;ISO]  [GO:0005794 "Golgi apparatus" evidence=IEA;ISO] [GO:0006915  "apoptotic process" evidence=IEA] |
| HP0535 | d.230.5.1 | Dodecin subunit-like | YbjQ-like | YbjQ-like | Alpha and beta proteins (a+b) |  |
| HP0536 | c.36.1.12 | Thiamin diphosphate-binding fold (THDP-binding) | Thiamin diphosphate-binding fold (THDP-binding) | PFOR PP module | Alpha and beta proteins (a/b) | [GO:0003674 "molecular_function" evidence=ND] [GO:0008150  "biological_process" evidence=ND] |
| HP0537 | a.25.1.2 | Ferritin-like | Ferritin-like | Ribonucleotide reductase-like | All alpha proteins | [GO:0005515 "protein binding" evidence=IPI] |
| HP0538 | a.25.1.4 | Ferritin-like | Ferritin-like | YciF-like | All alpha proteins | [GO:0005543 "phospholipid binding" evidence=IEA] |
| HP0539 | d.79.1.1 | Bacillus chorismate mutase-like | YjgF-like | YjgF/L-PSP | Alpha and beta proteins (a+b) | [GO:0000042 "protein targeting to  Golgi" evidence=IEA] |
| HP0540 | a.74.1.3 | Cyclin-like | Cyclin-like | Retinoblastoma tumor suppressor domains | All alpha proteins | [GO:0005874 "microtubule" evidence=IEA]  [GO:0005524 "ATP binding" evidence=IEA] [GO:0007018  "microtubule-based movement" evidence=IEA] |
| HP0541 | f.40.1.1 | V-type ATP synthase subunit C | V-type ATP synthase subunit C | V-type ATP synthase subunit C | Membrane and cell surface proteins and peptides |  |
| HP0542 | d.79.7.1 | Bacillus chorismate mutase-like | OmpA-like | OmpA-like | Alpha and beta proteins (a+b) |  |
| HP0543 | a.132.1.3 | Heme oxygenase-like | Heme oxygenase-like | TENA/THI-4 | All alpha proteins | [GO:0003674  "molecular_function" evidence=ND] [GO:0005575 "cellular_component"  evidence=ND] [GO:0008150 "biological_process" evidence=ND] |
| HP0544 | c.37.1.11 | P-loop containing nucleoside triphosphate hydrolases | P-loop containing nucleoside triphosphate hydrolases | RecA protein-like (ATPase-domain) | Alpha and beta proteins (a/b) | [GO:0003674  "molecular_function" evidence=ND] |
| HP0545 | c.55.1.1 | Ribonuclease H-like motif | Actin-like ATPase domain | Actin/HSP70 | Alpha and beta proteins (a/b) | [GO:0003779 "actin binding"  evidence=IEA] [GO:0008150 "biological_process" evidence=ND]  [GO:0005575 "cellular_component" evidence=ND] |
| HP0546 | a.39.1.8 | EF Hand-like | EF-hand | Penta-EF-hand proteins | All alpha proteins |  |
| HP0547 | a.238.1.4 | BAR/IMD domain-like | BAR/IMD domain-like | FCH domain | All alpha proteins | [GO:0005543 "phospholipid binding" evidence=IEA] |
| HP0549 | c.78.2.1 | ATC-like | Aspartate/glutamate racemase | Aspartate/glutamate racemase | Alpha and beta proteins (a/b) | [GO:0008881 "glutamate racemase  activity" evidence=ISS] [GO:0009252 "peptidoglycan biosynthetic  process" evidence=ISS] |
| HP0550 | c.37.1.11 | P-loop containing nucleoside triphosphate hydrolases | P-loop containing nucleoside triphosphate hydrolases | RecA protein-like (ATPase-domain) | Alpha and beta proteins (a/b) | [GO:0006353  "DNA-dependent transcription, termination" evidence=ISS] |
| HP0551 |  | Ribosome and ribosomal fragments | Ribosome and ribosomal fragments | Large subunit | Low resolution protein structures | [GO:0003735 "structural constituent  of ribosome" evidence=ISS] [GO:0042254 "ribosome biogenesis"  evidence=ISS] |
| HP0552 | c.90.1.1 | Tetrapyrrole methylase | Tetrapyrrole methylase | Tetrapyrrole methylase | Alpha and beta proteins (a/b) | [GO:0003674  "molecular_function" evidence=ND] [GO:0005575 "cellular_component"  evidence=ND] [GO:0008150 "biological_process" evidence=ND] |
| HP0553 | c.116.1.1 | alpha/beta knot | alpha/beta knot | SpoU-like RNA 2'-O ribose methyltransferase | Alpha and beta proteins (a/b) | [GO:0001510 "RNA  methylation" evidence=ISS] [GO:0008173 "RNA methyltransferase  activity" evidence=ISS] |
| HP0554 | a.35.1.3 | lambda repressor-like DNA-binding domains | lambda repressor-like DNA-binding domains | SinR domain-like | All alpha proteins | [GO:0003674  "molecular_function" evidence=ND] [GO:0005575 "cellular_component"  evidence=ND] [GO:0008150 "biological_process" evidence=ND] |
| HP0555 | d.129.6.2 | TBP-like | KA1-like | Ssp2 C-terminal domain-like | Alpha and beta proteins (a+b) | [GO:0003674  "molecular_function" evidence=ND] [GO:0005575 "cellular_component"  evidence=ND] [GO:0008150 "biological_process" evidence=ND] |
| HP0556 | f.38.1.1 | MFS general substrate transporter | MFS general substrate transporter | Glycerol-3-phosphate transporter | Membrane and cell surface proteins and peptides |  |
| HP0557 | c.14.1.4 | ClpP/crotonase | ClpP/crotonase | Biotin dependent carboxylase carboxyltransferase domain | Alpha and beta proteins (a/b) | [GO:0003989 "acetyl-CoA carboxylase activity" evidence=ISS]  [GO:0006633 "fatty acid biosynthetic process" evidence=ISS]  [GO:0009317 "acetyl-CoA carboxylase complex" evidence=ISS] |
| HP0558 | c.95.1.1 | Thiolase-like | Thiolase-like | Thiolase-related | Alpha and beta proteins (a/b) | [GO:0004315  "3-oxoacyl-[acyl-carrier-protein] synthase activity" evidence=ISS]  [GO:0006633 "fatty acid biosynthetic process" evidence=ISS] |
| HP0559 | a.28.1.1 | Acyl carrier protein-like | ACP-like | Acyl-carrier protein (ACP) | All alpha proteins | [GO:0000036 "ACP phosphopantetheine  attachment site binding involved in fatty acid biosynthetic  process" evidence=ISS] [GO:0006633 "fatty acid biosynthetic  process" evidence=ISS] |
| HP0561 | c.2.1.2 | NAD(P)-binding Rossmann-fold domains | NAD(P)-binding Rossmann-fold domains | Tyrosine-dependent oxidoreductases | Alpha and beta proteins (a/b) | [GO:0004316  "3-oxoacyl-[acyl-carrier-protein] reductase (NADPH) activity"  evidence=ISS] [GO:0006633 "fatty acid biosynthetic process"  evidence=ISS] |
| HP0562 | c.123.1.1 | CoA-transferase family III (CaiB/BaiF) | CoA-transferase family III (CaiB/BaiF) | CoA-transferase family III (CaiB/BaiF) | Alpha and beta proteins (a/b) | [GO:0003735 "structural constituent  of ribosome" evidence=ISS] [GO:0042254 "ribosome biogenesis"  evidence=ISS] |
| HP0563 | d.38.1.4 | Thioesterase/thiol ester dehydrase-isomerase | Thioesterase/thiol ester dehydrase-isomerase | MaoC-like | Alpha and beta proteins (a+b) |  |
| HP0564 | a.93.1.1 | Heme-dependent peroxidases | Heme-dependent peroxidases | CCP-like | All alpha proteins |  |
| HP0565 | g.32.1.1 | GLA-domain | GLA-domain | GLA-domain | Small proteins | [GO:0005886 "plasma membrane"  evidence=IEA] [GO:0016021 "integral to membrane" evidence=IEA]  [GO:0016020 "membrane" evidence=IEA] |
| HPO566 | d.21.1.1 | Diaminopimelate epimerase-like | Diaminopimelate epimerase-like | Diaminopimelate epimerase | Alpha and beta proteins (a+b) | [GO:0008837 "diaminopimelate  epimerase activity" evidence=ISS] [GO:0009089 "lysine biosynthetic  process via diaminopimelate" evidence=ISS] |
| HP0567 | f.1.1.1 | Toxins' membrane translocation domains | Colicin | Colicin | Membrane and cell surface proteins and peptides | [GO:0003674 "molecular_function"  evidence=ND] [GO:0008150 "biological_process" evidence=ND] |
| HP0568 | c.1.28.3 | TIM beta/alpha-barrel | Radical SAM enzymes | MoCo biosynthesis proteins | Alpha and beta proteins (a/b) | [GO:0003674 "molecular_function" evidence=ND]  [GO:0005575 "cellular_component" evidence=ND] [GO:0008150  "biological_process" evidence=ND] |
| HP0569 | c.37.1.8 | P-loop containing nucleoside triphosphate hydrolases | P-loop containing nucleoside triphosphate hydrolases | G proteins | Alpha and beta proteins (a/b) | [GO:0005525 "GTP binding"  evidence=ISS] [GO:0008152 "metabolic process" evidence=ISS] |
| HP0570 | c.56.5.3 | Phosphorylase/hydrolase-like | Zn-dependent exopeptidases | Leucine aminopeptidase, C-terminal domain | Alpha and beta proteins (a/b) | [GO:0004177  "aminopeptidase activity" evidence=ISS] [GO:0006508 "proteolysis"  evidence=ISS] |
| HP0571 | d.6.1.1 | Prion-like | Prion-like | Prion-like | Alpha and beta proteins (a+b) | [GO:0003674 "molecular_function"  evidence=ND] [GO:0008150 "biological_process" evidence=ND] |
| HP0572 | c.61.1.1 | PRTase-like | PRTase-like | Phosphoribosyltransferases (PRTases) | Alpha and beta proteins (a/b) | [GO:0003999 "adenine  phosphoribosyltransferase activity" evidence=ISS] [GO:0006166  "purine ribonucleoside salvage" evidence=ISS] |
| HP0573 | a.238.1.3 | BAR/IMD domain-like | BAR/IMD domain-like | IMD domain | All alpha proteins | [GO:0005515 "protein binding" evidence=IPI] |
| HP0574 | c.121.1.1 | Ribose/Galactose isomerase RpiB/AlsB | Ribose/Galactose isomerase RpiB/AlsB | Ribose/Galactose isomerase RpiB/AlsB | Alpha and beta proteins (a/b) | [GO:0004751  "ribose-5-phosphate isomerase activity" evidence=ISS] [GO:0009052  "pentose-phosphate shunt, non-oxidative branch" evidence=ISS] |
| HP0575 | f.20.1.1 | Clc chloride channel | Clc chloride channel | Clc chloride channel | Membrane and cell surface proteins and peptides | [GO:0003674  "molecular_function" evidence=ND] [GO:0005575 "cellular_component"  evidence=ND] [GO:0008150 "biological_process" evidence=ND] |
| HP0576 | b.87.1.2 | LexA/Signal peptidase | LexA/Signal peptidase | Type 1 signal peptidase | All beta proteins | [GO:0009306 "protein secretion"  evidence=ISS] |
| HP0577 | c.58.1.2 | Aminoacid dehydrogenase-like, N-terminal domain | Aminoacid dehydrogenase-like, N-terminal domain | Tetrahydrofolate dehydrogenase/cyclohydrolase | Alpha and beta proteins (a/b) | [GO:0000105 "histidine biosynthetic  process" evidence=ISS] [GO:0004477 "methenyltetrahydrofolate  cyclohydrolase activity" evidence=ISS] [GO:0004486  "methylenetetrahydrofolate dehydrogenase [NAD(P)+] activity"  evidence=ISS] [GO:0009086 "methionine biosynthetic process"  evidence=ISS] |
| HP0578 | c.76.1.2 | Alkaline phosphatase-like | Alkaline phosphatase-like | Arylsulfatase | Alpha and beta proteins (a/b) | [GO:0003674 "molecular_function" evidence=ND] [GO:0008150  "biological_process" evidence=ND] |
| HP0579 | g.3.11.1 | Knottins (small inhibitors, toxins, lectins) | EGF/Laminin | EGF-type module | Small proteins |  |
| HP0580 |  |  |  |  |  | [GO:0005575 "cellular_component" evidence=ND] [GO:0008150  "biological_process" evidence=ND] |
| HP0581 | c.1.9.4 | TIM beta/alpha-barrel | Metallo-dependent hydrolases | Dihydroorotase | Alpha and beta proteins (a/b) | [GO:0004151  "dihydroorotase activity" evidence=ISS] [GO:0009220 "pyrimidine  ribonucleotide biosynthetic process" evidence=ISS] |
| HP0582 | b.68.1.1 | 6-bladed beta-propeller | Sialidases | Sialidases (neuraminidases) | All beta proteins | [GO:0005215 "transporter activity"  evidence=ISS] [GO:0006810 "transport" evidence=ISS] |
| HP0583 |  |  |  |  |  |  |
| HP0584 | b.139.1.1 | Surface presentation of antigens (SPOA) | Surface presentation of antigens (SPOA) | Surface presentation of antigens (SPOA) | All beta proteins | [GO:0001539 "ciliary  or flagellar motility" evidence=ISS] [GO:0003774 "motor activity"  evidence=ISS] |
| HP0585 | a.96.1.1 | DNA-glycosylase | DNA-glycosylase | Endonuclease III | All alpha proteins | [GO:0003906 "DNA-(apurinic or  apyrimidinic site) lyase activity" evidence=ISS] [GO:0006281 "DNA  repair" evidence=ISS] |
| HP0586 | d.22.1.1 | GFP-like | GFP-like | Fluorescent proteins | Alpha and beta proteins (a+b) | [GO:0003674  "molecular_function" evidence=ND] [GO:0005575 "cellular_component"  evidence=ND] [GO:0008150 "biological_process" evidence=ND] |
| HP0587 | a.39.1.5 | EF Hand-like | EF Hand-like | Calmodulin-like | All alpha proteins | [GO:0003674  "molecular_function" evidence=ND] [GO:0005575 "cellular_component"  evidence=ND] [GO:0008150 "biological_process" evidence=ND] |
| HP0588 | d.58.1.2 | Ferredoxin-like | 4Fe-4S ferredoxins | 7-Fe ferredoxin | Alpha and beta proteins (a+b) | [GO:0047553  "2-oxoglutarate synthase activity" evidence=ISS] |
| HP0589 | c.36.1.8 | Thiamin diphosphate-binding fold (THDP-binding) | Thiamin diphosphate-binding fold (THDP-binding) | PFOR Pyr module | Alpha and beta proteins (a/b) | [GO:0047553  "2-oxoglutarate synthase activity" evidence=ISS] |
| HP0590 | c.36.1.12 | Thiamin diphosphate-binding fold (THDP-binding) | Thiamin diphosphate-binding fold (THDP-binding) | PFOR PP module | Alpha and beta proteins (a/b) | [GO:0047553  "2-oxoglutarate synthase activity" evidence=ISS] |
| HP0591 | c.64.1.1 | Pyruvate-ferredoxin oxidoreductase, PFOR, domain III | Pyruvate-ferredoxin oxidoreductase, PFOR, domain III | Pyruvate-ferredoxin oxidoreductase, PFOR, domain III | Alpha and beta proteins (a/b) | [GO:0047553  "2-oxoglutarate synthase activity" evidence=ISS] |
| HP0592 | c.37.1.19 | P-loop containing nucleoside triphosphate hydrolases | P-loop containing nucleoside triphosphate hydrolases | Tandem AAA-ATPase domain | Alpha and beta proteins (a/b) | [GO:0009307 "DNA restriction-modification system"  evidence=ISS] [GO:0015666 "restriction endodeoxyribonuclease  activity" evidence=ISS] |
| HP0593 | c.66.1.11 | S-adenosyl-L-methionine-dependent methyltransferases | S-adenosyl-L-methionine-dependent methyltransferases | Type II DNA methylase | Alpha and beta proteins (a/b) | [GO:0006306 "DNA methylation" evidence=ISS] [GO:0009008  "DNA-methyltransferase activity" evidence=ISS] |
| HP0594 | a.7.14.1 | Spectrin repeat-like | MIT domain | MIT domain | All alpha proteins | [GO:0003674  "molecular_function" evidence=ND] [GO:0005575 "cellular_component"  evidence=ND] [GO:0008150 "biological_process" evidence=ND] |
| HP0595 | e.61.1.1 | ImpE-like | ImpE-like | ImpE-like | Multi-domain proteins (alpha and beta) | [GO:0006457  "protein folding" evidence=ISS] [GO:0015036 "disulfide  oxidoreductase activity" evidence=ISS] [GO:0016021 "integral to  membrane" evidence=ISS] |
| HP0596 | c.82.1.1 | ALDH-like | ALDH-like | ALDH-like | Alpha and beta proteins (a/b) |  |
| HP0597 | e.3.1.1 | beta-lactamase/transpeptidase-like | beta-lactamase/transpeptidase-like | beta-Lactamase/D-ala carboxypeptidase | Multi-domain proteins (alpha and beta) | [GO:0008658  "penicillin binding" evidence=ISS] [GO:0009252 "peptidoglycan  biosynthetic process" evidence=ISS] |
| HP0598 | c.67.1.4 | PLP-dependent transferase-like | PLP-dependent transferase | GABA-aminotransferase-like | Alpha and beta proteins (a/b) | [GO:0008710 "8-amino-7-oxononanoate synthase activity"  evidence=ISS] [GO:0009102 "biotin biosynthetic process"  evidence=ISS] |
| HP0599 | a.238.1.4 | BAR/IMD domain-like | BAR/IMD domain-like | FCH domain | All alpha proteins | [GO:0005515 "protein binding" evidence=IPI] |
| HP0600 | f.37.1.1 | ABC transporter transmembrane region | ABC transporter transmembrane region | ABC transporter transmembrane region | Membrane and cell surface proteins and peptides | [GO:0016021 "integral to membrane" evidence=IBA] [GO:0042626  "ATPase activity, coupled to transmembrane movement of substances"  evidence=IBA] [GO:0055085 "transmembrane transport" evidence=IBA] |
| HP0601 | e.32.1.1 | Phase 1 flagellin | Phase 1 flagellin | Phase 1 flagellin | Multi-domain proteins (alpha and beta) | [GO:0001539 "ciliary  or flagellar motility" evidence=ISS] [GO:0005198 "structural  molecule activity" evidence=ISS] [GO:0007155 "cell adhesion"  evidence=ISS] |
| HP0602 | a.96.1.5 | DNA-glycosylase | DNA-glycosylase | Methyladenine DNA glycosylase III (MagIII) | All alpha proteins | [GO:0003906 "DNA-(apurinic or  apyrimidinic site) lyase activity" evidence=ISS] [GO:0006281 "DNA  repair" evidence=ISS] |
| HP0603 | f.4.1.1 | TRANSMEMBRANE BETA BAREELS | OMPA LIKE | OUTERMEMBRANE PROTEINS | Membrane and cell surface proteins and peptides |  |
| HP0604 | c.1.22.1 | TIM beta/alpha-barrel | UROD/MetE-like | Uroporphyrinogen decarboxylase, UROD | Alpha and beta proteins (a/b) | [GO:0004853  "uroporphyrinogen decarboxylase activity" evidence=ISS] [GO:0006779  "porphyrin-containing compound biosynthetic process" evidence=ISS] |
| HP0605 | f.5.1.1 | Outer membrane efflux proteins (OEP) | Outer membrane efflux proteins (OEP) | Outer membrane efflux proteins (OEP) | Membrane and cell surface proteins and peptides | [GO:0042930 "enterobactin transport" evidence=IGI] [GO:0014070  "response to organic cyclic compound" evidence=IMP] [GO:0009279  "cell outer membrane" evidence=IEA;IDA] |
| HP0606 | f.46.1.1 | HlyD-like secretion proteins | HlyD-like secretion proteins | HlyD-like secretion proteins | Membrane and cell surface proteins and peptides | [GO:0003674  "molecular_function" evidence=ND] [GO:0005575 "cellular_component"  evidence=ND] [GO:0008150 "biological_process" evidence=ND] |
| HP0607 | f.35.1.1 | Multidrug efflux transporter AcrB transmembrane domain | Multidrug efflux transporter AcrB transmembrane domain | Multidrug efflux transporter AcrB transmembrane domain | Membrane and cell surface proteins and peptides | [GO:0005215  "transporter activity" evidence=ISS] [GO:0006810 "transport"  evidence=ISS] |
| HP0608 | f.4.1.1 | TRANSMEMBRANE BETA BAREELS | OMPA LIKE | OUTERMEMBRANE PROTEINS | Membrane and cell surface proteins and peptides |  |
| HP0609 | a.204.1.2 | all-alpha NTP pyrophosphatases | all-alpha NTP pyrophosphatases | MazG-like | All alpha proteins | [GO:0016772 "transferase  activity, transferring phosphorus-containing groups" evidence=IEA]  [GO:0006468 "protein phosphorylation" evidence=IEA] [GO:0005524  "ATP binding" evidence=IEA] [GO:0004674 "protein serine/threonine  kinase activity" evidence=IEA] |
| HP0610 | b.40.4.5 | OB-fold | Nucleic acid-binding proteins | Cold shock DNA-binding domain-like | All beta proteins | [GO:0005575  "cellular_component" evidence=ND] [GO:0003674 "molecular_function"  evidence=ND] [GO:0008150 "biological_process" evidence=ND] |
| HP0611 | d.42.1.2 | POZ domain | POZ domain | Tetramerization domain of potassium channels | Alpha and beta proteins (a+b) | [GO:0006810  "transport" evidence=ISS] [GO:0042626 "ATPase activity, coupled to  transmembrane movement of substances" evidence=ISS] |
| HP0612 | a.47.1.1 | STAT-like | STAT | STAT | All alpha proteins |  |
| HP0613 | c.37.1.12 | P-loop containing nucleoside triphosphate hydrolases | P-loop containing nucleoside triphosphate hydrolases | ABC transporter ATPase domain-like | Alpha and beta proteins (a/b) | [GO:0006814 "sodium ion transport" evidence=ISS] [GO:0042626  "ATPase activity, coupled to transmembrane movement of substances"  evidence=ISS] |
| HP0614 | d.58.18.4 | Ferredoxin-like | ACT-like | Nickel responsive regulator NikR, C-terminal domain | Alpha and beta proteins (a+b) |  |
| HP0615 | d.142.2.2 | ATP-grasp | DNA ligase/mRNA capping enzyme, catalytic domain | Adenylation domain of NAD+-dependent DNA ligase | Alpha and beta proteins (a+b) | GO:0003911 "DNA ligase (NAD+)  activity" evidence=ISS] [GO:0006260 "DNA replication" evidence=ISS]  [GO:0006281 "DNA repair" evidence=ISS] |
| HP0616 | c.23.1.1 | Flavodoxin-like | CheY-like | CheY-related | Alpha and beta proteins (a/b) | [GO:0004871 "signal  transducer activity" evidence=ISS] [GO:0006935 "chemotaxis"  evidence=ISS] |
| HP0617 | d.104.1.1 | Class II aaRS and biotin synthetases | Class II aaRS and biotin synthetases | Class II aminoacyl-tRNA synthetase (aaRS)-like, catalytic domain | Alpha and beta proteins (a+b) | [GO:0004815 "aspartate-tRNA ligase  activity" evidence=ISS] [GO:0006422 "aspartyl-tRNA aminoacylation"  evidence=ISS] |
| HP0618 | c.37.1.1 | P-loop containing nucleoside triphosphate hydrolases | P-loop containing nucleoside triphosphate hydrolases | Nucleotide and nucleoside kinases | Alpha and beta proteins (a/b) | [GO:0004017 "adenylate kinase  activity" evidence=ISS] [GO:0015949 "nucleobase-containing small  molecule interconversion" evidence=ISS] |
| HP0620 | b.40.5.1 | OB-fold | Inorganic pyrophosphatase | Inorganic pyrophosphatase | All beta proteins | [GO:0004427 "inorganic diphosphatase  activity" evidence=ISS] [GO:0006793 "phosphorus metabolic process"  evidence=ISS] |
| HP0621 | c.37.1.12 | P-loop containing nucleoside triphosphate hydrolases | P-loop containing nucleoside triphosphate hydrolases | ABC transporter ATPase domain-like | Alpha and beta proteins (a/b) | [GO:0003677 "DNA  binding" evidence=ISS] [GO:0006259 "DNA metabolic process"  evidence=ISS] |
| HP0622 | f.23.2.1 | Single transmembrane helix | Mitochondrial cytochrome c oxidase subunit VIa | Mitochondrial cytochrome c oxidase subunit VIa | Membrane and cell surface proteins and peptides | [GO:0003674  "molecular_function" evidence=ND] [GO:0005575 "cellular_component"  evidence=ND] [GO:0008150 "biological_process" evidence=ND] |
| HP0623 | c.59.1.1 | MurD-like peptide ligases, peptide-binding domain | MurD-like peptide ligases, peptide-binding domain | MurCDEF C-terminal domain | Alpha and beta proteins (a/b) | [GO:0008763  "UDP-N-acetylmuramate-L-alanine ligase activity" evidence=ISS]  [GO:0009252 "peptidoglycan biosynthetic process" evidence=ISS] |
| HP0624 | c.67.1.1 | PLP-dependent transferase-like | PLP-dependent transferase-like | AAT-like | Alpha and beta proteins (a/b) | [GO:0009016 "succinyldiaminopimelate transaminase activity"  evidence=ISS] [GO:0009089 "lysine biosynthetic process via  diaminopimelate" evidence=ISS] |
| HP0625 | c.1.21.2 | TIM beta/alpha-barrel | Dihydropteroate synthetase-like | Methyltetrahydrofolate-utiluzing methyltransferases | Alpha and beta proteins (a/b) | [GO:0005737 "cytoplasm" evidence=ISS] [GO:0019288  "isopentenyl diphosphate biosynthetic process,  mevalonate-independent pathway" evidence=ISS] |
| HP0626 | b.81.1.2 | Single-stranded left-handed beta-helix | Trimeric LpxA-like enzymes | Tetrahydrodipicolinate-N-succinlytransferase, THDP-succinlytransferase, DapD | All beta proteins | [GO:0008666  "2,3,4,5-tetrahydropyridine-2,6-dicarboxylate N-succinyltransferase  activity" evidence=ISS] [GO:0009089 "lysine biosynthetic process  via diaminopimelate" evidence=ISS] |
| HP0627 | a.118.18.1 | alpha-alpha superhelix | HCP-like | HCP-like | All alpha proteins | [GO:0001890 "placenta development" evidence=ISO;ISS]  [GO:0005576 "extracellular region" evidence=IEA] [GO:0090214  "spongiotrophoblast layer developmental growth" evidence=ISO] |
| HP0628 | a.118.18.1 | alpha-alpha superhelix | HCP-like | HCP-like | All alpha proteins | [GO:0003674 "molecular_function"  evidence=ND] [GO:0008150 "biological_process" evidence=ND] |
| HP0629 | d.228.1.1 | Replication modulator SeqA, C-terminal DNA-binding domain | Replication modulator SeqA, C-terminal DNA-binding domain | Replication modulator SeqA, C-terminal DNA-binding domain | Alpha and beta proteins (a+b) | [GO:0003674  "molecular_function" evidence=ND] [GO:0005575 "cellular_component"  evidence=ND] [GO:0008150 "biological_process" evidence=ND] |
| HP0630 | c.23.5.3 | Flavodoxin-like | Flavodoxin-like | Quinone reductase | Alpha and beta proteins (a/b) | [GO:0003674  "molecular_function" evidence=ND] [GO:0005575 "cellular_component"  evidence=ND] [GO:0008150 "biological_process" evidence=ND] |
| HP0631 | e.19.1.1 | HydA/Nqo6-like | HydA/Nqo6-like | Nickel-iron hydrogenase, small subunit | Multi-domain proteins (alpha and beta) | [GO:0006091  "generation of precursor metabolites and energy" evidence=ISS]  [GO:0047067 "hydrogen:quinone oxidoreductase activity"  evidence=ISS] |
| HP0632 |  |  |  |  |  | [GO:0006091  "generation of precursor metabolites and energy" evidence=ISS]  [GO:0047067 " |
| HP0633 |  |  |  |  |  | [GO:0009055 "electron carrier activity" evidence=ISS] [GO:0016021  "integral to membrane" evidence=ISS] |
| HP0634 | c.56.1.1 | Phosphorylase/hydrolase-like | HybD-like | Hydrogenase maturating endopeptidase HybD | Alpha and beta proteins (a/b) | [GO:0008233 "peptidase  activity" evidence=ISS] [GO:0016485 "protein processing"  evidence=ISS] |
| HP0635 | d.115.1.1 | YrdC/RibB | YrdC/RibB | YrdC-like | Alpha and beta proteins (a+b) | [GO:0003674  "molecular_function" evidence=ND] [GO:0005575 "cellular_component"  evidence=ND] [GO:0008150 "biological_process" evidence=ND] |
| HP0636 | b.34.2.1 | SH3-like barrel | SH3-domain | SH3-domain | All beta proteins |  |
| HP0637 | e.25.1.1 | Sec1/munc18-like (SM) proteins | Sec1/munc18-like (SM) proteins | Sec1/munc18-like (SM) proteins | Multi-domain proteins (alpha and beta) | [GO:0016021  "integral to membrane" evidence=IEA] [GO:0004930 "G-protein coupled  receptor activity" evidence=IEA] [GO:0004984 "olfactory receptor  activity" evidence=IEA] |
| HP0638 | f.4.3.2 | Transmembrane beta-barrels | Porins | Maltoporin-like | Membrane and cell surface proteins and peptides |  |
| HP0639 | c.26.2.1 | Adenine nucleotide alpha hydrolase-like | Adenine nucleotide alpha hydrolase-like | N-type ATP pyrophosphatases | Alpha and beta proteins (a/b) | [GO:0008150 "biological_process" evidence=ND] |
| HP0640 | a.173.1.1 | Poly A polymerase C-terminal region-like | Poly A polymerase C-terminal region-like | Poly A polymerase C-terminal region-like | All alpha proteins | [GO:0004652 "polynucleotide  adenylyltransferase activity" evidence=ISS] [GO:0006396 "RNA  processing" evidence=ISS] |
| HP0641 | d.129.3.6 | TBP-like | Bet v1-like | oligoketide cyclase/dehydrase-like | Alpha and beta proteins (a+b) | [GO:0008679  "2-hydroxy-3-oxopropionate reductase activity" evidence=ISS]  [GO:0019582 "D-galactarate catabolic process" evidence=ISS] |
| HP0642 | d.90.1.1 | FMN-dependent nitroreductase-like | FMN-dependent nitroreductase-like | NADH oxidase/flavin reductase | Alpha and beta proteins (a+b) | [GO:0009055 "electron carrier activity"  evidence=ISS] |
| HP0643 | c.26.1.1 | Adenine nucleotide alpha hydrolase-like | Nucleotidylyl transferase | Class I aminoacyl-tRNA synthetases (RS), catalytic domain | Alpha and beta proteins (a/b) | [GO:0004818 "glutamate-tRNA ligase  activity" evidence=ISS] [GO:0006424 "glutamyl-tRNA aminoacylation"  evidence=ISS] |
| HP0644 | d.140.1.1 | Ribosomal protein S8 | Ribosomal protein S8 | Ribosomal protein S8 | Alpha and beta proteins (a+b) | [GO:0003674 "molecular_function"  evidence=ND] [GO:0005575 "cellular_component" evidence=ND]  [GO:0008150 "biological_process" evidence=ND] |
| HP0645 | a.118.5.1 | alpha-alpha superhelix | Bacterial muramidases | Bacterial muramidases | All alpha proteins | [GO:0009253  "peptidoglycan catabolic process" evidence=ISS] [GO:0016798  "hydrolase activity, acting on glycosyl bonds" evidence=ISS] |
| HP0646 | c.68.1.6 | Nucleotide-diphospho-sugar transferases | Nucleotide-diphospho-sugar transferases | glucose-1-phosphate thymidylyltransferase | Alpha and beta proteins (a/b) | [GO:0003983 "UTP:glucose-1-phosphate  uridylyltransferase activity" evidence=ISS] [GO:0009225  "nucleotide-sugar metabolic process" evidence=ISS] |
| HP0647 | a.238.1.3 | BAR/IMD domain-like | BAR/IMD domain-like | IMD domain | All alpha proteins | [GO:0005575  "cellular_component" evidence=ND] [GO:0003674 "molecular_function"  evidence=ND] [GO:0008150 "biological_process" evidence=ND] |
| HP0648 | d.68.2.2 | IF3-like | EPT/RTPC-like | Enolpyruvate transferase, EPT | Alpha and beta proteins (a+b) | [GO:0008760 "UDP-N-acetylglucosamine  1-carboxyvinyltransferase activity" evidence=ISS] [GO:0009252  "peptidoglycan biosynthetic process" evidence=ISS] |
| HP0649 | a.127.1.1 | L-aspartase-like | L-aspartase-like | L-aspartase/fumarase | All alpha proteins | [GO:0006520 "cellular amino acid  metabolic process" evidence=ISS] [GO:0008797 "aspartate  ammonia-lyase activity" evidence=ISS] |
| HP0650 | c.18.1.2 | Uracil-DNA glycosylase-like | Uracil-DNA glycosylase-like | Mug-like | Alpha and beta proteins (a/b) |  |
| HP0651 | c.87.1.11 | UDP-Glycosyltransferase/glycogen phosphorylase | UDP-Glycosyltransferase/glycogen phosphorylase | FucT-like | Alpha and beta proteins (a/b) | [GO:0000502 "proteasome complex" evidence=ISS] |
| HP0652 | c.108.1.4; | HAD-like | HAD-like | Phosphoserine phosphatase | Alpha and beta proteins (a/b) | [GO:0004647  "phosphoserine phosphatase activity" evidence=ISS] [GO:0006545  "glycine biosynthetic process" evidence=ISS] [GO:0006564 "L-serine  biosynthetic process" evidence=ISS] |
| HP0653 | a.25.1.1 | Ferritin-like | Ferritin-like | Ferritin | All alpha proteins | [GO:0006880  "intracellular sequestering of iron ion" evidence=ISS] [GO:0008199  "ferric iron binding" evidence=ISS] |
| HP0654 | c.1.28.1 | TIM beta/alpha-barrel | Radical SAM enzymes | Biotin synthase | Alpha and beta proteins (a/b) | [GO:0003824 "catalytic  activity" evidence=ISS] [GO:0008152 "metabolic process"  evidence=ISS] |
| HP0655 | d.153.1.4 | Ntn hydrolase-like | N-terminal nucleophile aminohydrolases (Ntn hydrolases) | Proteasome subunits | Alpha and beta proteins (a+b) | [GO:0003674  "molecular_function" evidence=ND] [GO:0008150 "biological_process"  evidence=ND] |
| HP0656 | c.1.28.1 | TIM beta/alpha-barrel | Radical SAM enzymes | Biotin synthase | Alpha and beta proteins (a/b) | [GO:0003824 "catalytic  activity" evidence=ISS] [GO:0008152 "metabolic process"  evidence=ISS] |
| HP0657 | d.185.1.1 | LuxS/MPP-like metallohydrolase | LuxS/MPP-like metallohydrolase | MPP-like | Alpha and beta proteins (a+b) | [GO:0003674 "molecular_function"  evidence=ND] [GO:0008150 "biological_process" evidence=ND] |
| HP0658 | d.128.1.5 | Glutamine synthetase/guanido kinase | Glutamine synthetase/guanido kinase | GatB/GatE catalytic domain-like | Alpha and beta proteins (a+b) | [GO:0006422 "aspartyl-tRNA aminoacylation" evidence=ISS]  [GO:0006424 "glutamyl-tRNA aminoacylation" evidence=ISS] |
| HP0659 | a.223.1.2 | Triger factor/SurA peptide-binding domain-like | Triger factor/SurA peptide-binding domain-like | Porin chaperone SurA, peptide-binding domain | All alpha proteins | [GO:0003674  "molecular_function" evidence=ND] [GO:0005575 "cellular_component"  evidence=ND] [GO:0008150 "biological_process" evidence=ND] |
| HP0660 | a.118.8.1 | alpha-alpha superhelix | TPR-like | Tetratricopeptide repeat (TPR) | All alpha proteins | [GO:0003674  "molecular_function" evidence=ND] [GO:0005575 "cellular_component"  evidence=ND] [GO:0008150 "biological_process" evidence=ND] |
| HP0661 | c.55.3.1 | Ribonuclease H-like motif | Ribonuclease H-like | Ribonuclease H | Alpha and beta proteins (a/b) | [GO:0004523 "ribonuclease H activity" evidence=ISS]  [GO:0006401 "RNA catabolic process" evidence=ISS] HAMAP:MF_00042 |
| HP0662 | a.149.1.1 | RNase III domain-like | RNase III domain-like | RNase III catalytic domain-like | All alpha proteins | [GO:0004525 "ribonuclease III  activity" evidence=ISS] [GO:0006396 "RNA processing" evidence=ISS] |
| HP0663 | d.258.1.1 | Chorismate synthase, AroC | Chorismate synthase, AroC | Chorismate synthase, AroC | Alpha and beta proteins (a+b) | [GO:0004107 "chorismate synthase  activity" evidence=ISS] [GO:0009423 "chorismate biosynthetic  process" evidence=ISS] |
| HP0664 | c.124.1.2 | NagB/RpiA/CoA transferase-like | NagB/RpiA/CoA transferase-like | CoA transferase alpha subunit-like | Alpha and beta proteins (a/b) | [GO:0003674 "molecular_function"  evidence=ND] [GO:0005575 "cellular_component" evidence=ND]  [GO:0008150 "biological_process" evidence=ND] |
| HP0665 | c.1.28.2 | TIM beta/alpha-barrel | Radical SAM enzymes | Oxygen-independent coproporphyrinogen III oxidase HemN | Alpha and beta proteins (a/b) | [GO:0004109  "coproporphyrinogen oxidase activity" evidence=ISS] [GO:0006779  "porphyrin-containing compound biosynthetic process" evidence=ISS] |
| HP0666 | e.26.1.2 | Prismane protein-like | Prismane protein-like | Carbon monoxide dehydrogenase | Multi-domain proteins (alpha and beta) | [GO:0008150  "biological_process" evidence=ND] |
| HP0667 | c.52.1.18 | Restriction endonuclease-like | Restriction endonuclease-like | Hjc-like | Alpha and beta proteins (a/b) |  |
| HP668 | c.37.1.19 | P-loop containing nucleoside triphosphate hydrolases | P-loop containing nucleoside triphosphate hydrolases | Tandem AAA-ATPase domain | Alpha and beta proteins (a/b) | [GO:0005739  "mitochondrion" evidence=IEA] [GO:0008150 "biological_process"  evidence=ND] |
| HP0669 | c.66.1.45 | S-adenosyl-L-methionine-dependent methyltransferases | S-adenosyl-L-methionine-dependent methyltransferases | N-6 DNA Methylase-like | Alpha and beta proteins (a/b) | [GO:0006306 "DNA methylation" evidence=ISS] [GO:0009007  "site-specific DNA-methyltransferase (adenine-specific) activity"  evidence=ISS] |
| HP0670 | d.17.1.3 | Cystatin-like | Cystatin/monellin | Cathelicidin motif | Alpha and beta proteins (a+b) | [GO:0003674 "molecular_function"  evidence=ND] |
| HP0671 | f.4.3.2 | Transmembrane beta-barrels | Porins | Maltoporin-like | Membrane and cell surface proteins and peptides |  |
| HP0672 | c.67.1.1 | PLP-dependent transferase-like | PLP-dependent transferase-like | AAT-like | Alpha and beta proteins (a/b) | [GO:0004069  "L-aspartate:2-oxoglutarate aminotransferase activity"  evidence=ISS] [GO:0006532 "aspartate biosynthetic process"  evidence=ISS] |
| HP0673 | a.149.1.1 | RNase III domain-like | RNase III domain-like | RNase III catalytic domain-like | All alpha proteins | [GO:0006355 "regulation of transcription,  DNA-dependent" evidence=ISS] |
| HP0674 | d.2.1.3 | Lysozyme-like | Lysozyme-like | Phage lysozyme | Alpha and beta proteins (a+b) |  |
| HP0675 | d.163.1.1 | DNA breaking-rejoining enzymes | DNA breaking-rejoining enzymes | Lambda integrase-like, catalytic core | Alpha and beta proteins (a+b) | [GO:0006310 "DNA recombination" evidence=ISS] [GO:0009009  "site-specific recombinase activity" evidence=ISS] [GO:0015074 "DNA  integration" evidence=ISS] |
| HP0676 | a.4.2.1 | DNA/RNA-binding 3-helical bundle | Methylated DNA-protein cysteine methyltransferase, C-terminal domain | Methylated DNA-protein cysteine methyltransferase, C-terminal domain | All alpha proteins | [GO:0005886 "plasma membrane" evidence=IDA] [GO:0006307 "DNA  dealkylation involved in DNA repair" evidence=IDA] [GO:0051409  "response to nitrosative stress" evidence=IDA] |
| HP0677 | c.66.1.53 | S-adenosyl-L-methionine-dependent methyltransferases | S-adenosyl-L-methionine-dependent methyltransferases | TrmB-like | Alpha and beta proteins (a/b) | [GO:0003674 "molecular_function"  evidence=ND] [GO:0008150 "biological_process" evidence=ND] |
| HP0678 | j.30.1.3 | Conotoxins | Conotoxins | Alpha-a-conotoxin | Peptides |  |
| HP0679 | c.2.1.3 | NAD(P)-binding Rossmann-fold domains | NAD(P)-binding Rossmann-fold domains | Glyceraldehyde-3-phosphate dehydrogenase-like, N-terminal domain | Alpha and beta proteins (a/b) | [GO:0000166 "nucleotide  binding" evidence=IEA] [GO:0055114 "oxidation-reduction process"  evidence=IEA] [GO:0016491 "oxidoreductase activity" evidence=IEA] |
| HP0680 | c.7.1.2 | PFL-like glycyl radical enzymes | PFL-like glycyl radical enzymes | R1 subunit of ribonucleotide reductase, C-terminal domain | Alpha and beta proteins (a/b) | [GO:0004748  "ribonucleoside-diphosphate reductase activity, thioredoxin  disulfide as acceptor" evidence=ISS] [GO:0009265  "2'-deoxyribonucleotide biosynthetic process" evidence=ISS] |
| HP0681 | b.40.4.5 | OB-fold | Nucleic acid-binding proteins | Cold shock DNA-binding domain-like | All beta proteins |  |
| HP0682 | b.40.4.11 | OB-fold | Nucleic acid-binding proteins | DNA replication initiator (cdc21/cdc54) N-terminal domain | All beta proteins |  |
| HP0683 | b.81.1.4 | Single-stranded left-handed beta-helix | Trimeric LpxA-like enzymes | GlmU C-terminal domain-like | All beta proteins | [GO:0003977 "UDP-N-acetylglucosamine diphosphorylase activity"  evidence=ISS] [GO:0009103 "lipopolysaccharide biosynthetic process"  evidence=ISS] [GO:0009252 "peptidoglycan biosynthetic process"  evidence=ISS] |
| HP0684 | e.8.1.4 | DNA/RNA polymerases | DNA/RNA polymerases | RNA-dependent RNA-polymerase | Multi-domain proteins (alpha and beta) | [GO:0001539 "ciliary  or flagellar motility" evidence=ISS] [GO:0008565 "protein  transporter activity" evidence=ISS] [GO:0009296 "flagellum  assembly" evidence=ISS] |
| HP0685 | d.29.1.1 | Ribosomal protein L31e | Ribosomal protein L31e | Ribosomal protein L31e | Alpha and beta proteins (a+b) | [GO:0001539 "ciliary  or flagellar motility" evidence=ISS] [GO:0008565 "protein  transporter activity" evidence=ISS] |
| HP0686 | f.4.3.3 | Transmembrane beta-barrels | Porins | Ligand-gated protein channel | Membrane and cell surface proteins and peptides | [GO:0044718 "siderophore  transmembrane transport" evidence=IEA] [GO:0015343 "siderophore  transmembrane transporter activity" evidence=IEA] |
| HP0687 | c.37.1.8 | P-loop containing nucleoside triphosphate hydrolases | P-loop containing nucleoside triphosphate hydrolases | G proteins | Alpha and beta proteins (a/b) | [GO:0015639  "ferrous iron uptake transmembrane transporter activity"  evidence=ISS] [GO:0015684 "ferrous iron transport" evidence=ISS] |
| HP0688 | c.55.3.5 | Ribonuclease H-like motif | Ribonuclease H-like motif | DnaQ-like 3'-5' exonuclease | Alpha and beta proteins (a/b) | [GO:0000271  "polysaccharide biosynthetic process" evidence=ISS] [GO:0003824  "catalytic activity" evidence=ISS] |
| HP0689 | c.1.8.10 | TIM beta/alpha-barrel | (Trans)glycosidases | alpha-D-glucuronidase/Hyaluronidase catalytic domain | Alpha and beta proteins (a/b) | [GO:0000271  "polysaccharide biosynthetic process" evidence=ISS] [GO:0003824  "catalytic activity" evidence=ISS] |
| HP0690 | c.95.1.1 | Thiolase-like | Thiolase-like | Thiolase-related | Alpha and beta proteins (a/b) | [GO:0003985 "acetyl-CoA C-acetyltransferase activity" evidence=ISS]  [GO:0006631 "fatty acid metabolic process" evidence=ISS] |
| HP0691 | c.124.1.2 | NagB/RpiA/CoA transferase-like | NagB/RpiA/CoA transferase-like | CoA transferase alpha subunit-like | Alpha and beta proteins (a/b) | [GO:0008152  "metabolic process" evidence=ISS] [GO:0008260 "3-oxoacid  CoA-transferase activity" evidence=ISS] |
| HP0692 | c.124.1.3 | NagB/RpiA/CoA transferase-like | NagB/RpiA/CoA transferase-like | CoA transferase beta subunit-like | Alpha and beta proteins (a/b) | [GO:0008260 "3-oxoacid CoA-transferase activity" evidence=ISS]  [GO:0018894 "dibenzo-p-dioxin metabolic process" evidence=ISS] |
| HP0693 |  |  |  |  |  | [GO:0003674 "molecular_function" evidence=ND]  [GO:0008150 "biological_process" evidence=ND] |
| HP0694 | f.4.3.3 | Transmembrane beta-barrels | Porins | Ligand-gated protein channel | Membrane and cell surface proteins and peptides | [GO:0003674  "molecular_function" evidence=ND] [GO:0005575 "cellular_component"  evidence=ND] [GO:0008150 "biological_process" evidence=ND] |
| HP0695 | c.55.1.10 | Ribonuclease H-like motif | Actin-like ATPase domain | ROK | Alpha and beta proteins (a/b) | [GO:0018710 "acetone  carboxylase activity" evidence=IDA] [GO:0043443 "acetone metabolic  process" evidence=IDA] |
| HP0696 | c.23.5.8 | Flavodoxin-like | Flavoproteins | WrbA-like | Alpha and beta proteins (a/b) | [GO:0018710 "acetone  carboxylase activity" evidence=IDA] [GO:0043443 "acetone metabolic  process" evidence=IDA] |
| HP0697 | a.180.1.1 | N-terminal, cytoplasmic domain of anti-sigmaE factor RseA | N-terminal, cytoplasmic domain of anti-sigmaE factor RseA | N-terminal, cytoplasmic domain of anti-sigmaE factor RseA | All alpha proteins | [GO:0018710 "acetone  carboxylase activity" evidence=IDA] [GO:0043443 "acetone metabolic  process" evidence=IDA] |
| HP0698 | a.224.1.1 | Glycolipid transfer protein, GLTP | Glycolipid transfer protein, GLTP | Glycolipid transfer protein, GLTP | All alpha proteins |  |
| HP0699 |  |  |  |  |  | [GO:0005515 "protein binding"  evidence=IPI] |
| HP0700 | a.63.1.1 | Apolipophorin-III | Apolipophorin-III | Apolipophorin-III | All alpha proteins | [GO:0004143 "diacylglycerol kinase  activity" evidence=ISS] [GO:0016021 "integral to membrane"  evidence=ISS] |
| HP0701 | e.11.1.1 | Type II DNA topoisomerase | Type II DNA topoisomerase | Type II DNA topoisomerase | Multi-domain proteins (alpha and beta) | [GO:0003916 "DNA topoisomerase  activity" evidence=ISS] [GO:0003918 "DNA topoisomerase type II  (ATP-hydrolyzing) activity" evidence=ISS] |
| HP0702 | b.1.1.2 | Immunoglobulin-like beta-sandwich | Immunoglobulin | C1 set domains (antibody constant domain-like) | All beta proteins |  |
| HP0703 | c.37.1.20 | P-loop containing nucleoside triphosphate hydrolases | P-loop containing nucleoside triphosphate hydrolases | Extended AAA-ATPase domain | Alpha and beta proteins (a/b) | [GO:0000156  "phosphorelay response regulator activity" evidence=ISS]  [GO:0000160 "phosphorelay signal transduction system" evidence=ISS]  [GO:0003700 "sequence-specific DNA binding transcription factor  activity" evidence=ISS] |
| HP0704 | b.34.7.1 | SH3-like barrel | DNA-binding domain of retroviral integrase | DNA-binding domain of retroviral integrase | All beta proteins |  |
| HP0705 | c.37.1.12 | P-loop containing nucleoside triphosphate hydrolases | P-loop containing nucleoside triphosphate hydrolases | ABC transporter ATPase domain-like | Alpha and beta proteins (a/b) | [GO:0006281 "DNA  repair" evidence=ISS] [GO:0009380 "excinuclease repair complex"  evidence=ISS] [GO:0009381 "excinuclease ABC activity" evidence=ISS] |
| HP0706 | f.4.3.2 | Transmembrane beta-barrels | Porins | Maltoporin-like | Membrane and cell surface proteins and peptides |  |
| HP0707 | c.66.1.23 | S-adenosyl-L-methionine-dependent methyltransferases | S-adenosyl-L-methionine-dependent methyltransferases | MraW-like putative methyltransferases | Alpha and beta proteins (a/b) | [GO:0008152 "metabolic  process" evidence=ISS] [GO:0008168 "methyltransferase activity"  evidence=ISS] |
| HP0708 | a.118.7.1 | alpha-alpha superhelix | 14-3-3 protein | 14-3-3 protein | All alpha proteins | [GO:0003674  "molecular_function" evidence=ND] [GO:0005575 "cellular_component"  evidence=ND] [GO:0008150 "biological_process" evidence=ND] |
| HP0709 | c.132.1.1 | Bacterial fluorinating enzyme, N-terminal domain | Bacterial fluorinating enzyme, N-terminal domain | Bacterial fluorinating enzyme, N-terminal domain | Alpha and beta proteins (a/b) | [GO:0005575  "cellular_component" evidence=ND] [GO:0008150 "biological_process"  evidence=ND] |
| HP0710 | f.4.1.1 | TRANSMEMBRANE BETA BAREELS | OMPA LIKE | OUTERMEMBRANE PROTEINS | Membrane and cell surface proteins and peptides | [GO:0003674  "molecular_function" evidence=ND] [GO:0005575 "cellular_component"  evidence=ND] [GO:0008150 "biological_process" evidence=ND] |
| HP0711 | a.211.1.1 | HD-domain/PDEase-like | HD-domain/PDEase-like | HD domain | All alpha proteins | [GO:0003674  "molecular_function" evidence=ND] [GO:0005575 "cellular_component"  evidence=ND] [GO:0008150 "biological_process" evidence=ND] |
| HP0712 | f.54.1.1 | SNF-like | SNF-like | SNF-like | Membrane and cell surface proteins and peptides | [GO:0000090 "mitotic anaphase"  evidence=IEA] [GO:0000940 "condensed chromosome outer kinetochore"  evidence=IEA] [GO:0005876 "spindle microtubule" evidence=IEA] |
| HP0713 | f.54.1.1 | SNF-like | SNF-like | SNF-like | Membrane and cell surface proteins and peptides | [GO:0070733 "protein adenylyltransferase activity"  evidence=ISS] [GO:0018117 "protein adenylylation" evidence=ISS]  [GO:0000166 "nucleotide binding" evidence=IEA] |
| HP0714 | a.208.1.1 | DhaL-like | DhaL-like | DhaL-like | All alpha proteins | [GO:0005515 "protein binding" evidence=IPI] |
| HP0715 | c.37.1.12 | P-loop containing nucleoside triphosphate hydrolases | P-loop containing nucleoside triphosphate hydrolases | ABC transporter ATPase domain-like | Alpha and beta proteins (a/b) | [GO:0005524 "ATP  binding" evidence=ISS] [GO:0006810 "transport" evidence=ISS]  [GO:0042626 "ATPase activity, coupled to transmembrane movement of  substances" evidence=ISS] |
| HP0716 | c.37.1.18 | P-loop containing nucleoside triphosphate hydrolases | P-loop containing nucleoside triphosphate hydrolases | YjeE-like | Alpha and beta proteins (a/b) | [GO:0003674  "molecular_function" evidence=ND] [GO:0005575 "cellular_component"  evidence=ND] [GO:0008150 "biological_process" evidence=ND] |
| HP0717 | c.37.1.20 | P-loop containing nucleoside triphosphate hydrolases | P-loop containing nucleoside triphosphate hydrolases | Extended AAA-ATPase domain | Alpha and beta proteins (a/b) | [GO:0003887  "DNA-directed DNA polymerase activity" evidence=ISS] [GO:0006260  "DNA replication" evidence=ISS] [GO:0009360 "DNA polymerase III  complex" evidence=ISS] |
| HP0718 | f.36.1.1 | Neurotransmitter-gated ion-channel transmembrane pore | Neurotransmitter-gated ion-channel transmembrane pore | Neurotransmitter-gated ion-channel transmembrane pore | Membrane and cell surface proteins and peptides | [GO:0005215 "transporter activity"  evidence=ISS] [GO:0006810 "transport" evidence=ISS] |
| HP0719 | b.34.15.1 | SH3-like barrel | Hypothetical protein YfhH | Hypothetical protein YfhH | Hypothetical protein YfhH | [GO:0003674  "molecular_function" evidence=ND] [GO:0008150 "biological_process"  evidence=ND] |
| HP0720 | b.38.1.2 | Sm-like fold | Sm-like ribonucleoproteins | Pleiotropic translational regulator Hfq | All beta proteins |  |
| HP0721 | b.34.15.1 | SH3-like barrel | Hypothetical protein YfhH | Hypothetical protein YfhH | Hypothetical protein YfhH | [GO:0007507 "heart development"  evidence=IMP] |
| HP0722 | f.4.1.1 | TRANSMEMBRANE BETA BAREELS | OMPA LIKE | OUTERMEMBRANE PROTEINS | Membrane and cell surface proteins and peptides | [GO:0003674  "molecular_function" evidence=ND] [GO:0008150 "biological_process"  evidence=ND] |
| HP0723 | c.88.1.1 | Glutaminase/Asparaginase | Glutaminase/Asparaginase | Glutaminase/Asparaginase | Alpha and beta proteins (a/b) | [GO:0042597 "periplasmic space" evidence=ISM] [GO:0006528  "asparagine metabolic process" evidence=IEA] [GO:0006520 "cellular  amino acid metabolic process" evidence=IEA] |
| HP0724 |  |  |  |  |  | [GO:0015556 "C4-dicarboxylate transmembrane transporter activity"  evidence=ISS] [GO:0015740 "C4-dicarboxylate transport"  evidence=ISS] |
| HP0725 | f.4.1.1 | TRANSMEMBRANE BETA BAREELS | OMPA LIKE | OUTERMEMBRANE PROTEINS | Membrane and cell surface proteins and peptides | [GO:0003674  "molecular_function" evidence=ND] [GO:0005739 "mitochondrion"  evidence=NAS] [GO:0016020 "membrane" evidence=NAS] |
| HP0726 | f.4.5.1 | Transmembrane beta-barrels | Autotransporter | Autotransporter | Membrane and cell surface proteins and peptides |  |
| HP0727 | c.1.4.1 | TIM beta/alpha-barrel | FMN-linked oxidoreductases | FMN-linked oxidoreductases | Alpha and beta proteins (a/b) | [GO:0006400 "tRNA  modification" evidence=ISS] [GO:0017150 "tRNA dihydrouridine  synthase activity" evidence=ISS] |
| HP0728 | c.26.2.5 | Adenine nucleotide alpha hydrolase-like | Adenine nucleotide alpha hydrolase-like | PP-loop ATPase | Alpha and beta proteins (a/b) | [GO:0006400 "tRNA  modification" evidence=ISS] [GO:0016879 "ligase activity, forming  carbon-nitrogen bonds" evidence=ISS] |
| HP0729 | b.140.1.1 | Replicase NSP9 | Replicase NSP9 | Replicase NSP9 | All beta proteins | [GO:0004820  "glycine-tRNA ligase activity" evidence=ISS] [GO:0006426  "glycyl-tRNA aminoacylation" evidence=ISS] |
| HP0730 |  |  |  |  |  | [GO:0003674  "molecular_function" evidence=ND] [GO:0005575 "cellular_component"  evidence=ND] [GO:0008150 "biological_process" evidence=ND] |
| HP0731 | a.118.1.1 | alpha-alpha superhelix | ARM repeat | Armadillo repeat | All alpha proteins | [GO:0030260 "entry into host cell" evidence=TAS] |
| HP0732 | c.1.19.2 | TIM beta/alpha-barrel | Cobalamin (vitamin B12)-dependent enzymes | Glutamate mutase, large subunit | Alpha and beta proteins (a/b) | [GO:0042157 "lipoprotein metabolic process" evidence=IEA]  [GO:0008289 "lipid binding" evidence=IEA] [GO:0005576  "extracellular region" evidence=IEA] [GO:0006869 "lipid transport"  evidence=IEA] |
| HP0733 | a.118.1.1 | alpha-alpha superhelix | ARM repeat | Armadillo repeat | All alpha proteins | [GO:0043021  "ribonucleoprotein complex binding" evidence=IGI;IPI] [GO:0051028  "mRNA transport" evidence=IEA] |
| HP0734 | c.1.28.1 | TIM beta/alpha-barrel | Radical SAM enzymes | Biotin synthase | Alpha and beta proteins (a/b) | [GO:0003824  "catalytic activity" evidence=ISS] [GO:0006400 "tRNA modification"  evidence=ISS] |
| HP0735 | c.61.1.1 | PRTase-like | PRTase-like | Phosphoribosyltransferases (PRTases) | Alpha and beta proteins (a/b) | [GO:0009117  "nucleotide metabolic process" evidence=ISS] [GO:0016763  "transferase activity, transferring pentosyl groups" evidence=ISS] |
| HP0736 | c.67.1.3 | PLP-dependent transferase-like | PLP-dependent transferase-like | Cystathionine synthase-like | Alpha and beta proteins (a/b) | [GO:0016695 "oxidoreductase activity, acting on hydrogen as donor"  evidence=ISS] |
| HP0737 | a.195.1.1 | YutG-like | YutG-like | YutG-like | All alpha proteins | [GO:0008962  "phosphatidylglycerophosphatase activity" evidence=ISS] [GO:0009395  "phospholipid catabolic process" evidence=ISS] |
| HP0738 | d.142.1.1 | ATP-grasp | Glutathione synthetase ATP-binding domain-like | ATP-binding domain of peptide synthetases | Alpha and beta proteins (a+b) | [GO:0008716  "D-alanine-D-alanine ligase activity" evidence=ISS] [GO:0009252  "peptidoglycan biosynthetic process" evidence=ISS] |
| HP0739 | c.69.1.10 | alpha/beta-Hydrolases | alpha/beta-Hydrolases | Carbon-carbon bond hydrolase | Alpha and beta proteins (a/b) | [GO:0003674  "molecular_function" evidence=ND] [GO:0005575 "cellular_component"  evidence=ND] [GO:0008150 "biological_process" evidence=ND] |
| HP0740 | c.72.2.1 | Ribokinase-like | MurD-like peptide ligases, catalytic domain | MurCDEF | Alpha and beta proteins (a/b) | [GO:0003824 "catalytic activity"  evidence=ISS] [GO:0008152 "metabolic process" evidence=ISS] |
| HP0741 | d.13.1.1 | HIT-like | HIT-like | HIT (HINT, histidine triad) family of protein kinase-interacting proteins | Alpha and beta proteins (a+b) | [GO:0003674 "molecular_function"  evidence=ND] [GO:0008150 "biological_process" evidence=ND] |
| HP0742 | c.61.1.2 | PRTase-like | PRTase-like | Phosphoribosylpyrophosphate synthetase-like | Alpha and beta proteins (a/b) | [GO:0004749 "ribose  phosphate diphosphokinase activity" evidence=ISS] [GO:0009152  "purine ribonucleotide biosynthetic process" evidence=ISS] |
| HP0743 | g.23.1.1 | TB module/8-cys domain | TB module/8-cys domain | TB module/8-cys domain | Small proteins | [GO:0003824  "catalytic activity" evidence=ISS] [GO:0008152 "metabolic process"  evidence=ISS] [GO:0008360 "regulation of cell shape" evidence=ISS] |
| HP0745 | d.265.1.3 | Pseudouridine synthase | Pseudouridine synthase | Pseudouridine synthase RsuA/RluD | Alpha and beta proteins (a+b) | [GO:0009451 "RNA modification" evidence=ISS] [GO:0009982  "pseudouridine synthase activity" evidence=ISS] |
| HP0746 | b.1.2.1 | Immunoglobulin-like beta-sandwich | Fibronectin type III | Fibronectin type III | All beta proteins | [GO:0003674  "molecular_function" evidence=ND] [GO:0005575 "cellular_component"  evidence=ND] [GO:0008150 "biological_process" evidence=ND] |
| HP0747 | c.66.1.16 | S-adenosyl-L-methionine-dependent methyltransferases | S-adenosyl-L-methionine-dependent methyltransferases | Guanidinoacetate methyltransferase | Alpha and beta proteins (a/b) | [GO:0006400 "tRNA  modification" evidence=ISS] [GO:0008176 "tRNA  (guanine-N7-)-methyltransferase activity" evidence=ISS] |
| HP0748 | c.37.1.12 | P-loop containing nucleoside triphosphate hydrolases | P-loop containing nucleoside triphosphate hydrolases | ABC transporter ATPase domain-like | Alpha and beta proteins (a/b) | [GO:0006810 "transport" evidence=ISS] [GO:0042626 "ATPase activity,  coupled to transmembrane movement of substances" evidence=ISS] |
| HP0749 | a.114.1.1 | Interferon-induced guanylate-binding protein 1 (GBP1), C-terminal domain | Interferon-induced guanylate-binding protein 1 (GBP1), C-terminal domain | Interferon-induced guanylate-binding protein 1 (GBP1), C-terminal domain | All alpha proteins | [GO:0003674  "molecular_function" evidence=ND] |
| HP0750 | a.238.1.4 | BAR/IMD domain-like | BAR/IMD domain-like | FCH domain | All alpha proteins | [GO:0004222 "metalloendopeptidase  activity" evidence=ISS] [GO:0006508 "proteolysis" evidence=ISS] |
| HP0751 | d.352.1.1 | FlaG-like | FlaG-like | FlaG-like | Alpha and beta proteins (a+b) | [GO:0003674 "molecular_function"  evidence=ND] |
| HP0752 | a.238.1.4 | BAR/IMD domain-like | BAR/IMD domain-like | FCH domain | All alpha proteins | [GO:0001539 "ciliary  or flagellar motility" evidence=ISS] [GO:0005198 "structural  molecule activity" evidence=ISS] [GO:0009424 "bacterial-type  flagellum hook" evidence=ISS] |
| HP0753 | a.24.18.1 | Four-helical up-and-down bundle | Oxygen-evolving enhancer protein 3, | Oxygen-evolving enhancer protein 3, | All alpha proteins | GO:0003674 "molecular_function"  evidence=ND] |
| HP0754 | a.238.1.4 | BAR/IMD domain-like | BAR/IMD domain-like | FCH domain | All alpha proteins | [GO:0003674  "molecular_function" evidence=ND] [GO:0005575 "cellular_component"  evidence=ND] [GO:0008150 "biological_process" evidence=ND] |
| HP0755 | c.111.1.1 | Activating enzymes of the ubiquitin-like proteins | Activating enzymes of the ubiquitin-like proteins | Molybdenum cofactor biosynthesis protein MoeB | Alpha and beta proteins (a/b) | [GO:0003674 "molecular_function"  evidence=ND] [GO:0008150 "biological_process" evidence=ND] |
| HP0756 | d.230.5.1 | Dodecin subunit-like | YbjQ-like | YbjQ-like | Alpha and beta proteins (a+b) |  |
| HP0757 | d.160.1.2 | Carbon-nitrogen hydrolase | Carbon-nitrogen hydrolase | Carbamilase | Alpha and beta proteins (a+b) | [GO:0008152 "metabolic  process" evidence=ISS] [GO:0016810 "hydrolase activity, acting on  carbon-nitrogen (but not peptide) bonds" evidence=ISS] |
| HP0758 |  |  |  |  |  | [GO:0006814 "sodium  ion transport" evidence=ISS] [GO:0006818 "hydrogen transport"  evidence=ISS] [GO:0015385 "sodium:hydrogen antiporter activity"  evidence=ISS] |
| HP0759 | f.24.1.1 | Cytochrome c oxidase subunit I-like | Cytochrome c oxidase subunit I-like | Cytochrome c oxidase subunit I-like | Membrane and cell surface proteins and peptides | [GO:0005215 "transporter activity" evidence=ISS] [GO:0006810  "transport" evidence=ISS] |
| HP0760 |  |  |  |  |  | [GO:0003674 "molecular_function" evidence=ND]  [GO:0008150 "biological_process" evidence=ND] |
| HP0761 | c.124.1.6 | NagB/RpiA/CoA transferase-like | NagB/RpiA/CoA transferase-like | Methenyltetrahydrofolate synthetase [ | Alpha and beta proteins (a/b) | [GO:0003674  "molecular_function" evidence=ND] [GO:0005575 "cellular_component"  evidence=ND] [GO:0008150 "biological_process" evidence=ND] |
| HP0762 | c.47.1.10 | Thioredoxin fold | Thioredoxin fold | Glutathione peroxidase-like | Alpha and beta proteins (a/b) | [GO:0003674 "molecular_function"  evidence=ND] [GO:0008150 "biological_process" evidence=ND] |
| HP0763 | c.37.1.10 | P-loop containing nucleoside triphosphate hydrolases | P-loop containing nucleoside triphosphate hydrolases | Nitrogenase iron protein-like | Alpha and beta proteins (a/b) | [GO:0005515 "protein binding"  evidence=IPI] |
| HP0764 | d.134.1.1 | Nitrite and sulphite reductase 4Fe-4S domain-like | Nitrite and sulphite reductase 4Fe-4S domain-like | Nitrite and sulphite reductase 4Fe-4S domain-like | Alpha and beta proteins (a+b) | [GO:0003674  "molecular_function" evidence=ND] [GO:0005575 "cellular_component"  evidence=ND] [GO:0008150 "biological_process" evidence=ND] |
| HP0765 | d.201.1.1 | SRP19 | SRP19 | SRP19 | Alpha and beta proteins (a+b) |  |
| HP0766 | f.1.1.1 | Toxins' membrane translocation domains | Colicin | Colicin | Membrane and cell surface proteins and peptides | [GO:0003674  "molecular_function" evidence=ND] [GO:0005575 "cellular_component"  evidence=ND] [GO:0008150 "biological_process" evidence=ND] |
| HP0768 | c.1.28.3 | TIM beta/alpha-barrel | Radical SAM enzymes | MoCo biosynthesis proteins | Alpha and beta proteins (a/b) | [GO:0003824 "catalytic  activity" evidence=ISS] [GO:0006777 "Mo-molybdopterin cofactor  biosynthetic process" evidence=ISS] |
| HP0769 | c.68.1.8 | Nucleotide-diphospho-sugar transferases | Nucleotide-diphospho-sugar transferases | Molybdenum cofactor biosynthesis protein MobA | Alpha and beta proteins (a/b) | [GO:0003824 "catalytic activity" evidence=ISS]  [GO:0006777 "Mo-molybdopterin cofactor biosynthetic process"  evidence=ISS] |
| HP0770 | d.367.1.1 | EscU C-terminal domain-like | EscU C-terminal domain-like | EscU C-terminal domain-like | Alpha and beta proteins (a+b) | [GO:0001539 "ciliary or flagellar motility" evidence=ISS]  [GO:0005198 "structural molecule activity" evidence=ISS]  [GO:0009296 "flagellum assembly" evidence=ISS] |
| HP0772 | c.56.5.6 | Phosphorylase/hydrolase-like | Zn-dependent exopeptidases | N-acetylmuramoyl-L-alanine amidase-like | Alpha and beta proteins (a/b) | [GO:0008745  "N-acetylmuramoyl-L-alanine amidase activity" evidence=ISS]  [GO:0009253 "peptidoglycan catabolic process" evidence=ISS] |
| HP0773 | c.1.5.1 | TIM beta/alpha-barrel | Inosine monophosphate dehydrogenase (IMPDH) | Inosine monophosphate dehydrogenase (IMPDH) | Alpha and beta proteins (a/b) | [GO:0008152 "metabolic process" evidence=ISS] [GO:0016491  "oxidoreductase activity" evidence=ISS] |
| HP0774 | c.26.1.1 | Adenine nucleotide alpha hydrolase-like | Nucleotidylyl transferase | Class I aminoacyl-tRNA synthetases (RS), catalytic domain | Alpha and beta proteins (a/b) | [GO:0004831 "tyrosine-tRNA ligase  activity" evidence=ISS] [GO:0006437 "tyrosyl-tRNA aminoacylation"  evidence=ISS] |
| HP0775 | a.211.1.1 | HD-domain/PDEase-like | HD-domain/PDEase-like | HD domain | All alpha proteins | [GO:0003824 "catalytic activity"  evidence=ISS] [GO:0015968 "stringent response" evidence=ISS] |
| HP0776 | a.143.1.1 | RPB6/omega subunit-like | RPB6/omega subunit-like | RNA polymerase omega subunit | All alpha proteins | [GO:0003899  "DNA-directed RNA polymerase activity" evidence=ISS] |
| HP0777 | c.73.1.3 | Carbamate kinase-like | Carbamate kinase-like | PyrH-like | Alpha and beta proteins (a/b) | [GO:0009041 "uridylate kinase  activity" evidence=ISS] [GO:0015949 "nucleobase-containing small  molecule interconversion" evidence=ISS] |
| HP0778 | c.94.1.1 | Periplasmic binding protein-like II | Periplasmic binding protein-like II | Phosphate binding protein-like | Alpha and beta proteins (a/b) | [GO:0003674  "molecular_function" evidence=ND] [GO:0005575 "cellular_component"  evidence=ND] [GO:0008150 "biological_process" evidence=ND] |
| HP0779 | c.83.1.1 | Aconitase iron-sulfur domain | Aconitase iron-sulfur domain | Aconitase iron-sulfur domain | Alpha and beta proteins (a/b) | [GO:0003994 "aconitate hydratase  activity" evidence=ISS] [GO:0006099 "tricarboxylic acid cycle"  evidence=ISS] |
| HP0780 | f.5.1.1; | Outer membrane efflux proteins (OEP) | Outer membrane efflux proteins (OEP) | Outer membrane efflux proteins (OEP) | Membrane and cell surface proteins and peptides | [GO:0005813 "centrosome" evidence=IEA] |
| HP0781 | c.93.1.1 | Periplasmic binding protein-like I | Periplasmic binding protein-like I | L-arabinose binding protein-like | Alpha and beta proteins (a/b) | [GO:0003674  "molecular_function" evidence=ND] [GO:0005575 "cellular_component"  evidence=ND] [GO:0008150 "biological_process" evidence=ND] |
| HP0782 | f.4.3.3 | Transmembrane beta-barrels | Porins | Ligand-gated protein channel | Membrane and cell surface proteins and peptides |  |
| HP0783 | a.238.1.4 | BAR/IMD domain-like | BAR/IMD domain-like | FCH domain | All alpha proteins | [GO:0005100 "Rho GTPase  activator activity" evidence=ISS] [GO:0005622 "intracellular"  evidence=IEA] [GO:0007165 "signal transduction" evidence=IEA] |
| HP0784 | d.9.1.1 | IL8-like | Interleukin 8-like chemokines | Interleukin 8-like chemokines | Alpha and beta proteins (a+b) |  |
| HP0785 | b.125.1.1 | LolA-like prokaryotic lipoproteins and lipoprotein localization factors | Prokaryotic lipoproteins and lipoprotein localization factors | Outer-membrane lipoproteins carrier protein LolA | All beta proteins | [GO:0003674  "molecular_function" evidence=ND] [GO:0005575 "cellular_component"  evidence=ND] [GO:0008150 "biological_process" evidence=ND] |
| HP0786 | c.37.1.19 | P-loop containing nucleoside triphosphate hydrolases | P-loop containing nucleoside triphosphate hydrolases | Tandem AAA-ATPase domain | Alpha and beta proteins (a/b) | [GO:0009306 "protein  secretion" evidence=ISS] [GO:0015450 "P-P-bond-hydrolysis-driven  protein transmembrane transporter activity" evidence=ISS] |
| HP0787 | c.83.1.1 | Aconitase iron-sulfur domain | Aconitase iron-sulfur domain | Aconitase iron-sulfur domain | Alpha and beta proteins (a/b) | [GO:0006810 "transport" evidence=ISS] |
| HP0788 | f.4.3.3 | Transmembrane beta-barrels | Porins | Ligand-gated protein channel | Membrane and cell surface proteins and peptides [ |  |
| HP0789 | c.2.1.2 | NAD(P)-binding Rossmann-fold domains | NAD(P)-binding Rossmann-fold domains | Tyrosine-dependent oxidoreductases | Alpha and beta proteins (a/b) |  |
| HP0790 | d.287.1.2 | DNA methylase specificity domain [ | DNA methylase specificity domain [ | Type I restriction modification DNA specificity domain | Alpha and beta proteins (a+b) | [GO:0009307 "DNA  restriction-modification system" evidence=ISS] [GO:0015666  "restriction endodeoxyribonuclease activity" evidence=ISS] |
| HP0791 | d.220.1.1; | Metal cation-transporting ATPase, ATP-binding domain N | Metal cation-transporting ATPase, ATP-binding domain N | Metal cation-transporting ATPase, ATP-binding domain N | Alpha and beta proteins (a+b) | [GO:0006812 "cation  transport" evidence=ISS] [GO:0015662 "ATPase activity, coupled to  transmembrane movement of ions, phosphorylative mechanism"  evidence=ISS] |
| HP0792 | d.14.1.10 | Ribosomal protein S5 domain 2-like | Ribosomal protein S5 domain 2-like | ATP-dependent protease Lon (La), catalytic domain | Alpha and beta proteins (a+b) | [GO:0003674  "molecular_function" evidence=ND] [GO:0008150 "biological_process"  evidence=ND] |
| HP0793 | d.167.1.1 | Peptide deformylase | Peptide deformylase | Peptide deformylase | Alpha and beta proteins (a+b) | [GO:0006464 "cellular protein  modification process" evidence=ISS] [GO:0042586 "peptide  deformylase activity" evidence=ISS] |
| HP0794 | c.14.1.1 | ClpP/crotonase | ClpP/crotonase | Clp protease, ClpP subunit | Alpha and beta proteins (a/b) | [GO:0009368 "endopeptidase Clp complex" evidence=ISS] |
| HP0795 | d.26.1.1 | FKBP-like | FKBP-like | FKBP immunophilin/proline isomerase | Alpha and beta proteins (a+b) | [GO:0003755 "peptidyl-prolyl  cis-trans isomerase activity" evidence=ISS] [GO:0006457 "protein  folding" evidence=ISS] |
| HP0796 | f.4.3.2 | Transmembrane beta-barrels | Porins | Maltoporin-like | Membrane and cell surface proteins and peptides |  |
| HP0797 | c.51.6.1 | Anticodon-binding domain-like | XCC0632-like | NLBH-like | Alpha and beta proteins (a/b) |  |
| HP0798 | d.58.21.1 | Ferredoxin-like | Molybdenum cofactor biosynthesis protein C, MoaC | Molybdenum cofactor biosynthesis protein C, MoaC | Alpha and beta proteins (a+b) | [GO:0003824  "catalytic activity" evidence=ISS] [GO:0006777 "Mo-molybdopterin  cofactor biosynthetic process" evidence=ISS] |
| HP0799 | c.57.1.1 | Molybdenum cofactor biosynthesis proteins | Molybdenum cofactor biosynthesis proteins | MogA-like | Alpha and beta proteins (a/b) | [GO:0003824 "catalytic  activity" evidence=ISS] [GO:0006777 "Mo-molybdopterin cofactor  biosynthetic process" evidence=ISS] |
| HP0800 | d.41.5.1 | alpha/beta-Hammerhead | Molybdopterin synthase subunit MoaE | Molybdopterin synthase subunit MoaE | Alpha and beta proteins (a+b) | [GO:0003824  "catalytic activity" evidence=ISS] [GO:0006777 "Mo-molybdopterin  cofactor biosynthetic process" evidence=ISS] |
| HP0801 | d.15.3.1 | beta-Grasp (ubiquitin-like) | MoaD/ThiS | MoaD | Alpha and beta proteins (a+b) | [GO:0003824 "catalytic activity"  evidence=ISS] [GO:0009228 "thiamine biosynthetic process"  evidence=ISS] |
| HP0802 | c.144.1.1 | RibA-like | RibA-like | RibA-like | Alpha and beta proteins (a/b) | [GO:0003935 "GTP cyclohydrolase II  activity" evidence=ISS] [GO:0009231 "riboflavin biosynthetic  process" evidence=ISS] |
| HP0803 | f.33.1.1 | Calcium ATPase, transmembrane domain M | Calcium ATPase, transmembrane domain M | Calcium ATPase, transmembrane domain M | Membrane and cell surface proteins and peptides | [GO:0003674 "molecular_function" evidence=ND] [GO:0005575  "cellular_component" evidence=ND] [GO:0008150 "biological_process"  evidence=ND] |
| HP0804 | d.115.1.2 | YrdC/RibB | YrdC/RibB | 3,4-dihydroxy-2-butanone 4-phosphate synthase, DHBP synthase, RibB | Alpha and beta proteins (a+b) | [GO:0008686  "3,4-dihydroxy-2-butanone-4-phosphate synthase activity"  evidence=ISS] [GO:0009231 "riboflavin biosynthetic process"  evidence=ISS] |
| HP0805 | d.52.10.1 | Alpha-lytic protease prodomain-like | EspE N-terminal domain-like | GSPII protein E N-terminal domain-like | Alpha and beta proteins (a+b) | [GO:0009103 "lipopolysaccharide biosynthetic process"  evidence=ISS] [GO:0016757 "transferase activity, transferring  glycosyl groups" evidence=ISS] |
| HP0806 | c.60.1.2 | Phosphoglycerate mutase-like | Phosphoglycerate mutase-like | Histidine acid phosphatase | Alpha and beta proteins (a/b) | [GO:0003674  "molecular_function" evidence=ND] [GO:0005575 "cellular_component"  evidence=ND] [GO:0008150 "biological_process" evidence=ND] |
| HP0807 | f.4.3.3 | Transmembrane beta-barrels | Porins | Ligand-gated protein channel | Membrane and cell surface proteins and peptides | [GO:0044718 "siderophore  transmembrane transport" evidence=IEA] [GO:0015343 "siderophore  transmembrane transporter activity" evidence=IEA] [GO:0005506 "iron  ion binding" evidence=IEA] |
| HP0808 | d.150.1.2 | 4'-phosphopantetheinyl transferase | 4'-phosphopantetheinyl transferase | Holo-(acyl carrier protein) synthase ACPS | Alpha and beta proteins (a+b) | [GO:0008610 "lipid  biosynthetic process" evidence=ISS] [GO:0008897  "holo-[acyl-carrier-protein] synthase activity" evidence=ISS] |
| HP0809 | a.118.9.4 | alpha-alpha superhelix | ENTH/VHS domain | RPR domain (SMART 00582 ) | All alpha proteins | [GO:0001539 "ciliary or flagellar  motility" evidence=ISS] [GO:0005198 "structural molecule activity"  evidence=ISS] [GO:0006935 "chemotaxis" evidence=ISS] [GO:0009288  "bacterial-type flagellum" evidence=ISS] |
| HP0810 | c.66.1.46 | S-adenosyl-L-methionine-dependent methyltransferases | S-adenosyl-L-methionine-dependent methyltransferases | YhhF-like | Alpha and beta proteins (a/b) | [GO:0006306  "DNA methylation" evidence=ISS] [GO:0009008 "DNA-methyltransferase  activity" evidence=ISS] |
| HP0811 |  |  |  |  |  | [GO:0003674  "molecular_function" evidence=ND] [GO:0005575 "cellular_component"  evidence=ND] [GO:0008150 "biological_process" evidence=ND] |
| HP0812 | c.66.1.52 | S-adenosyl-L-methionine-dependent methyltransferases | S-adenosyl-L-methionine-dependent methyltransferases | RPA4359-like | Alpha and beta proteins (a/b) | [GO:0003674  "molecular_function" evidence=ND] [GO:0005575 "cellular_component"  evidence=ND] [GO:0008150 "biological_process" evidence=ND] |
| HP0813 | d.157.1.2 | Metallo-hydrolase/oxidoreductase | Metallo-hydrolase/oxidoreductase | Glyoxalase II (hydroxyacylglutathione hydrolase) | Alpha and beta proteins (a+b) | [GO:0003824 "catalytic  activity" evidence=ISS] [GO:0008152 "metabolic process"  evidence=ISS] |
| HP0814 | c.111.1.1 | Activating enzymes of the ubiquitin-like proteins | Activating enzymes of the ubiquitin-like proteins | Molybdenum cofactor biosynthesis protein MoeB | Alpha and beta proteins (a/b) | [GO:0003674  "molecular_function" evidence=ND] [GO:0008150 "biological_process"  evidence=ND] |
| HP0815 | c.116.1.1 | alpha/beta knot | alpha/beta knot | SpoU-like RNA 2'-O ribose methyltransferase | Alpha and beta proteins (a/b) | [GO:0001539 "ciliary  or flagellar motility" evidence=ISS] [GO:0003774 "motor activity"  evidence=ISS] |
| HP0816 | d.79.7.1 | Bacillus chorismate mutase-like | OmpA-like | OmpA-like | Alpha and beta proteins (a+b) | [GO:0005515 "protein binding" evidence=IPI] |
| HP0818 | f.58.1.1 | MetI-like | MetI-like | MetI-like | Membrane and cell surface proteins and peptides | [GO:0006810 "transport"  evidence=ISS] [GO:0042626 "ATPase activity, coupled to  transmembrane movement of substances" evidence=ISS] |
| HP0819 | c.37.1.12 | P-loop containing nucleoside triphosphate hydrolases | P-loop containing nucleoside triphosphate hydrolases | ABC transporter ATPase domain-like | Alpha and beta proteins (a/b) | [GO:0017111 "nucleoside-triphosphatase activity"  evidence=IEA] [GO:0016887 "ATPase activity" evidence=IEA]  [GO:0016820 "hydrolase activity, acting on acid anhydrides,  catalyzing transmembrane movement of substances" evidence=IEA] |
| HP0820 | f.13.1.2; | Family A G protein-coupled receptor-like | Family A G protein-coupled receptor-like | Rhodopsin-like | Membrane and cell surface proteins and peptides |  |
| HP0821 | c.55.5.2 | Ribonuclease H-like motif | Nitrogenase accessory factor-like | Nitrogenase accessory factor-like | Alpha and beta proteins (a/b) | [GO:0006281 "DNA  repair" evidence=ISS] [GO:0009380 "excinuclease repair complex"  evidence=ISS] [GO:0009381 "excinuclease ABC activity" evidence=ISS] |
| HP0822 | d.81.1.2 | FwdE/GAPDH domain-like | Glyceraldehyde-3-phosphate dehydrogenase-like, C-terminal domain | Homoserine dehydrogenase-like | Alpha and beta proteins (a+b) | [GO:0004412 "homoserine dehydrogenase  activity" evidence=ISS] [GO:0009067 "aspartate family amino acid  biosynthetic process" evidence=ISS] |
| HP0823 | c.52.1.30 | Restriction endonuclease-like | Restriction endonuclease-like | MRR-like | Alpha and beta proteins (a/b) | [GO:0003674 "molecular_function"  evidence=ND] [GO:0005575 "cellular_component" evidence=ND]  [GO:0008150 "biological_process" evidence=ND] |
| HP0824 | c.47.1.1 | Thioredoxin fold | Thioredoxin fold | Thioltransferase | Alpha and beta proteins (a/b) | [GO:0009055 "electron carrier  activity" evidence=ISS] |
| HP0825 | c.3.1.5 | FAD/NAD(P)-binding domain | FAD/NAD(P)-binding domain | FAD/NAD-linked reductases, N-terminal and central domains | Alpha and beta proteins (a/b) | [GO:0004791 "thioredoxin-disulfide reductase activity"  evidence=ISS] |
| HP0826 | d.52.10.1 | Alpha-lytic protease prodomain-like | EspE N-terminal domain-like | GSPII protein E N-terminal domain-like | Alpha and beta proteins (a+b) | [GO:0009103 "lipopolysaccharide biosynthetic process"  evidence=ISS] [GO:0016757 "transferase activity, transferring  glycosyl groups" evidence=ISS] |
| HP0827 | d.58.7.1 | Ferredoxin-like | RNA-binding domain, RBD | Canonical RBD | Alpha and beta proteins (a+b) | [GO:0005575  "cellular_component" evidence=ND] [GO:0008150 "biological_process"  evidence=ND] |
| HP0828 | f.18.1.1 | F1F0 ATP synthase subunit A | F1F0 ATP synthase subunit A | F1F0 ATP synthase subunit A | Membrane and cell surface proteins and peptides | [GO:0015986 "ATP  synthesis coupled proton transport" evidence=ISS] [GO:0045263  "proton-transporting ATP synthase complex, coupling factor F(o)"  evidence=ISS] [GO:0046933 "proton-transporting ATP synthase  activity, rotational mechanism" evidence=ISS] |
| HP0829 | c.1.5.1 | TIM beta/alpha-barrel | Inosine monophosphate dehydrogenase (IMPDH) | Inosine monophosphate dehydrogenase (IMPDH) | Alpha and beta proteins (a/b) | [GO:0003938 "IMP  dehydrogenase activity" evidence=ISS] [GO:0009152 "purine  ribonucleotide biosynthetic process" evidence=ISS] |
| HP0830 | c.117.1.1 | Amidase signature (AS) enzymes | Amidase signature (AS) enzymes | Amidase signature (AS) enzymes | Alpha and beta proteins (a/b) | [GO:0006422 "aspartyl-tRNA aminoacylation" evidence=ISS]  [GO:0006424 "glutamyl-tRNA aminoacylation" evidence=ISS] |
| HP0831 | c.37.1.1 | P-loop containing nucleoside triphosphate hydrolases | P-loop containing nucleoside triphosphate hydrolases | Nucleotide and nucleoside kinases | Alpha and beta proteins (a/b) | [GO:0004140 "dephospho-CoA kinase  activity" evidence=ISS] [GO:0015937 "coenzyme A biosynthetic  process" evidence=ISS] |
| HP0832 | c.66.1.17 | S-adenosyl-L-methionine-dependent methyltransferases | S-adenosyl-L-methionine-dependent methyltransferases | Spermidine synthase | Alpha and beta proteins (a/b) | [GO:0004766 "spermidine synthase  activity" evidence=ISS] [GO:0008295 "spermidine biosynthetic  process" evidence=ISS] |
| HP0833 | b.96.1.1 | Nicotinic receptor ligand binding domain-like | Nicotinic receptor ligand binding domain-like | Nicotinic receptor ligand binding domain-like | All beta proteins |  |
| HP0834 | c.37.1.8 | P-loop containing nucleoside triphosphate hydrolases | P-loop containing nucleoside triphosphate hydrolases | G proteins | Alpha and beta proteins (a/b) | [GO:0003924 "GTPase activity"  evidence=ISS] [GO:0005525 "GTP binding" evidence=ISS] [GO:0008152  "metabolic process" evidence=ISS] |
| HP0835 | a.55.1.1 | IHF-like DNA-binding proteins | IHF-like DNA-binding proteins | Prokaryotic DNA-bending protein | All alpha proteins | [GO:0003677 "DNA  binding" evidence=ISS] [GO:0005694 "chromosome" evidence=ISS]  [GO:0006323 "DNA packaging" evidence=ISS] |
| HP0836 | c.66.1.35 | S-adenosyl-L-methionine-dependent methyltransferases | S-adenosyl-L-methionine-dependent methyltransferases | Salicylic acid carboxyl methyltransferase (SAMT) | Alpha and beta proteins (a/b) | [GO:0003674 "molecular_function" evidence=ND] [GO:0005739  "mitochondrion" evidence=ISM] [GO:0008150 "biological_process"  evidence=ND] |
| HP0837 | d.50.2.1 | dsRBD-like | Porphobilinogen deaminase (hydroxymethylbilane synthase), C-terminal domain | Porphobilinogen deaminase (hydroxymethylbilane synthase), C-terminal domain | Alpha and beta proteins (a+b) | [GO:0003674 "molecular_function"  evidence=ND] [GO:0008150 "biological_process" evidence=ND] |
| HP0838 | g.24.1.1 | TNF receptor-like | TNF receptor-like | TNF receptor-like | Small proteins | [GO:0003674 "molecular_function"  evidence=ND] [GO:0008150 "biological_process" evidence=ND] |
| HP0839 | f.4.3.4 | Transmembrane beta-barrels | Porins | Outer membrane protein transport protein | Membrane and cell surface proteins and peptides | [GO:0005215 "transporter activity" evidence=ISS] [GO:0006810  "transport" evidence=ISS] [GO:0019867 "outer membrane"  evidence=ISS] |
| HP0840 | c.2.1.2 | NAD(P)-binding Rossmann-fold domains | NAD(P)-binding Rossmann-fold domains | Tyrosine-dependent oxidoreductases | Alpha and beta proteins (a/b) | [GO:0003674  "molecular_function" evidence=ND] |
| HP0841 | c.34.1.1 | Homo-oligomeric flavin-containing Cys decarboxylases, HFCD | Homo-oligomeric flavin-containing Cys decarboxylases, HFCD | Homo-oligomeric flavin-containing Cys decarboxylases, HFCD | Alpha and beta proteins (a/b) | [GO:0004632  "phosphopantothenate--cysteine ligase activity" evidence=ISS]  [GO:0004633 "phosphopantothenoylcysteine decarboxylase activity"  evidence=ISS] [GO:0009055 "electron carrier activity" evidence=ISS] |
| HP0842 | b.68.9.1 | 6-bladed beta-propeller | NHL repeat | NHL repeat | All beta proteins |  |
| HP0843 | c.1.3.1 | TIM beta/alpha-barrel | Thiamin phosphate synthase | Thiamin phosphate synthase | Alpha and beta proteins (a/b) | [GO:0004789  "thiamine-phosphate diphosphorylase activity" evidence=ISS]  [GO:0009228 "thiamine biosynthetic process" evidence=ISS] |
| HP0844 | c.72.1.2 | Ribokinase-like | Ribokinase-like | Thiamin biosynthesis kinases | Alpha and beta proteins (a/b) | [GO:0008972  "phosphomethylpyrimidine kinase activity" evidence=ISS] [GO:0009228  "thiamine biosynthetic process" evidence=ISS] |
| HP0845 | c.72.1.2 | Ribokinase-like | Ribokinase-like | Thiamin biosynthesis kinases | Alpha and beta proteins (a/b) | GO:0004417 "hydroxyethylthiazole kinase activity" evidence=ISS]  [GO:0009228 "thiamine biosynthetic process" evidence=ISS] |
| HP0846 |  |  |  |  |  | [GO:0009035 "Type I site-specific deoxyribonuclease activity"  evidence=ISS] [GO:0009307 "DNA restriction-modification system"  evidence=ISS] [GO:0019812 "Type I site-specific deoxyribonuclease  complex" evidence=ISS] |
| HP0847 | b.29.1.18 | Concanavalin A-like lectins/glucanases | Concanavalin A-like lectins/glucanases | Alginate lyase | All beta proteins |  |
| HP0848 | d.287.1.2 | DNA methylase specificity domain | DNA methylase specificity domain | Type I restriction modification DNA specificity domain | Alpha and beta proteins (a+b) |  |
| HP0849 | d.287.1.2 | DNA methylase specificity domain | DNA methylase specificity domain | Type I restriction modification DNA specificity domain | Alpha and beta proteins (a+b) |  |
| HP0850 | c.66.1.45 | S-adenosyl-L-methionine-dependent methyltransferases | S-adenosyl-L-methionine-dependent methyltransferases | N-6 DNA Methylase-like | Alpha and beta proteins (a/b) | [GO:0006304 "DNA modification" evidence=ISS] [GO:0009007  "site-specific DNA-methyltransferase (adenine-specific) activity"  evidence=ISS] |
| HP0851 | a.111.1.1 | Acid phosphatase/Vanadium-dependent haloperoxidase | Acid phosphatase/Vanadium-dependent haloperoxidase | Type 2 phosphatidic acid phosphatase, PAP2 | All alpha proteins | [GO:0003674  "molecular_function" evidence=ND] [GO:0008150 "biological_process"  evidence=ND] |
| HP0852 | d.198.1.1 | Secretion chaperone-like | Type III secretory system chaperone-like | Type III secretory system chaperone-like | Alpha and beta proteins (a+b) | [GO:0003674 "molecular_function" evidence=ND]  [GO:0008150 "biological_process" evidence=ND] |
| HP0853 | a.118.1.2 | alpha-alpha superhelix | ARM repeat | HEAT repeat | All alpha proteins | [GO:0006810  "transport" evidence=ISS] [GO:0042626 "ATPase activity, coupled to  transmembrane movement of substances" evidence=ISS] |
| HP0854 | c.1.5.1 | TIM beta/alpha-barrel | Inosine monophosphate dehydrogenase (IMPDH) | Inosine monophosphate dehydrogenase (IMPDH) | Alpha and beta proteins (a/b) | [GO:0003920 "GMP  reductase activity" evidence=ISS] [GO:0015949  "nucleobase-containing small molecule interconversion"  evidence=ISS] |
| HP0855 | d.44.1.1 | Fe,Mn superoxide dismutase (SOD), C-terminal domain | Fe,Mn superoxide dismutase (SOD), C-terminal domain | Fe,Mn superoxide dismutase (SOD), C-terminal domain | Alpha and beta proteins (a+b) | [GO:0000271 "polysaccharide  biosynthetic process" evidence=ISS] [GO:0016413  "O-acetyltransferase activity" evidence=ISS] |
| HP0856 | a.65.1.1 | Annexin | Annexin | Annexin | All alpha proteins | [GO:0020011  "apicoplast" evidence=RCA] |
| HP0857 | c.80.1.3 | SIS domain | SIS domain | mono-SIS domain | Alpha and beta proteins (a/b) | [GO:0008968 "D-sedoheptulose  7-phosphate isomerase activity" evidence=ISS] [GO:0009244  "lipopolysaccharide core region biosynthetic process" evidence=ISS] |
| HP0858 | c.72.1.1 | Ribokinase-like | Ribokinase-like | Ribokinase-like | Alpha and beta proteins (a/b) | [GO:0008713  "ADP-heptose-lipopolysaccharide heptosyltransferase activity"  evidence=ISS] [GO:0009244 "lipopolysaccharide core region  biosynthetic process" evidence=ISS] |
| HP0859 | c.2.1.2 | NAD(P)-binding Rossmann-fold domains | NAD(P)-binding Rossmann-fold domains | Tyrosine-dependent oxidoreductases | Alpha and beta proteins (a/b) | [GO:0008712  "ADP-glyceromanno-heptose 6-epimerase activity" evidence=ISS]  [GO:0009244 "lipopolysaccharide core region biosynthetic process"  evidence=ISS] |
| HP0860 | c.108.1.19 | HAD-like | HAD-like | Histidinol phosphatase-like | Alpha and beta proteins (a/b) | [GO:0000287 "magnesium  ion binding" evidence=ISS] [GO:0008270 "zinc ion binding"  evidence=ISS] [GO:0034200 "D,D-heptose 1,7-bisphosphate phosphatase  activity" evidence=ISS] |
| HP0861 | d.42.1.2 | POZ domain | POZ domain | Tetramerization domain of potassium channels | Alpha and beta proteins (a+b) | [GO:0003674 "molecular_function"  evidence=ND] [GO:0005575 "cellular_component" evidence=ND] |
| HP0862 | c.55.1.13 | Ribonuclease H-like motif | Actin-like ATPase domain | CoaX-like | Alpha and beta proteins (a/b) | [GO:0006355  "regulation of transcription, DNA-dependent" evidence=ISS] |
| HP0863 | b.69.4.1 | bladed beta-propeller | WD40 repeat-like | WD40 repeat | All beta proteins | [GO:0003674 "molecular_function"  evidence=ND] [GO:0008150 "biological_process" evidence=ND] |
| HP0864 | a.118.8.2 | alpha-alpha superhelix | TPR-like | Transcription factor MalT domain III | All alpha proteins | [GO:0003674  "molecular_function" evidence=ND] [GO:0005575 "cellular_component"  evidence=ND] [GO:0008150 "biological_process" evidence=ND] |
| HP0865 | b.85.4.1 | beta-clip | dUTPase-like | dUTPase-like | All beta proteins | [GO:0004170 "dUTP diphosphatase activity" evidence=ISS]  [GO:0009394 "2'-deoxyribonucleotide metabolic process"  evidence=ISS] |
| HP0866 | a.2.1.1 | Long alpha-hairpin | GreA transcript cleavage protein, N-terminal domain | GreA transcript cleavage protein, N-terminal domain | All alpha proteins | [GO:0006354  "DNA-dependent transcription, elongation" evidence=ISS] |
| HP0867 | c.87.1.2 | UDP-Glycosyltransferase/glycogen phosphorylase | UDP-Glycosyltransferase/glycogen phosphorylase | Peptidoglycan biosynthesis glycosyltransferase MurG | Alpha and beta proteins (a/b) | [GO:0008915  "lipid-A-disaccharide synthase activity" evidence=ISS] [GO:0009245  "lipid A biosynthetic process" evidence=ISS] |
| HP0868 | b.110.1.1 | Cloacin translocation domain | Cloacin translocation domain | Cloacin translocation domain | All beta proteins | [GO:0003674  "molecular_function" evidence=ND] [GO:0005575 "cellular_component"  evidence=ND] [GO:0005737 "cytoplasm" evidence=NAS] |
| HP0869 | c.1.15.5 | TIM beta/alpha-barrel | Xylose isomerase-like | Hypothetical protein YgbM (EC1530) | Alpha and beta proteins (a/b) | [GO:0003674 "molecular_function" evidence=ND] |
| HP0870 | b.152.1.1 | Flagellar hook protein flgE | Flagellar hook protein flgE | Flagellar hook protein flgE | All beta proteins | [GO:0001539 "ciliary or flagellar  motility" evidence=ISS] [GO:0005198 "structural molecule activity"  evidence=ISS] [GO:0009424 "bacterial-type flagellum hook"  evidence=ISS] |
| HP0871 | d.13.1.4 | HIT-like | HIT-like | CDH-like | Alpha and beta proteins (a+b) | [GO:0006629  "lipid metabolic process" evidence=IEA] [GO:0046342  "CDP-diacylglycerol catabolic process" evidence=IEA] [GO:0005886  "plasma membrane" evidence=IEA] [GO:0016787 "hydrolase activity"  evidence=IEA] |
| HP0872 | g.41.3.5 | Rubredoxin-like | Zinc beta-ribbon | PhnA zinc-binding domain | Small proteins | [GO:0003674  "molecular_function" evidence=ND] [GO:0008150 "biological_process"  evidence=ND] |
| HP0873 | d.131.1.2 | DNA clamp | DNA clamp | DNA polymerase processivity factor | Alpha and beta proteins (a+b) | [GO:0016021 "integral to membrane" evidence=IEA] [GO:0005934  "cellular bud tip" evidence=IEA] [GO:0032153 "cell division site"  evidence=IEA] [GO:0003674 "molecular_function" evidence=ND] |
| HP0874 | b.80.6.1 | Single-stranded right-handed beta-helix | Stabilizer of iron transporter SufD | Stabilizer of iron transporter SufD | All beta proteins |  |
| HP0875 | e.5.1.1 | Heme-dependent catalase-like | Heme-dependent catalase-like | Heme-dependent catalase | Multi-domain proteins (alpha and beta) | [GO:0004096 "catalase activity" evidence=ISS]  [GO:0006979 "response to oxidative stress" evidence=ISS] |
| HP0876 | f.4.3.3 | Transmembrane beta-barrels | Porins | Ligand-gated protein channel | Membrane and cell surface proteins and peptides | [GO:0015232 "heme transporter activity" evidence=ISS] [GO:0015886  "heme transport" evidence=ISS] |
| HP0877 | c.55.3.6 | Ribonuclease H-like motif | Ribonuclease H-like | RuvC resolvase | Alpha and beta proteins (a/b) | [GO:0006310 "DNA  recombination" evidence=ISS] [GO:0008821 "crossover junction  endodeoxyribonuclease activity" evidence=ISS] |
| HP0879 | c.1.12.7 | TIM beta/alpha-barrel | Phosphoenolpyruvate/pyruvate domain | Phosphoenolpyruvate mutase/Isocitrate lyase-like | Alpha and beta proteins (a/b) | [GO:0003674  "molecular_function" evidence=ND] [GO:0005575 "cellular_component"  evidence=ND] [GO:0008150 "biological_process" evidence=ND] |
| HP0880 | c.127.1.1 | F420-dependent methylenetetrahydromethanopterin dehydrogenase (MTD) | F420-dependent methylenetetrahydromethanopterin dehydrogenase (MTD) | F420-dependent methylenetetrahydromethanopterin dehydrogenase (MTD) | Alpha and beta proteins (a/b) |  |
| HP0883 |  | RuvA C-terminal domain-lik | DNA helicase RuvA subunit, C-terminal domain | DNA helicase RuvA subunit, C-terminal domain | All alpha proteins | [GO:0032508 "DNA  duplex unwinding" evidence=IEA] [GO:0005737 "cytoplasm"  evidence=IEA] [GO:0000725 "recombinational repair" evidence=IMP]  [GO:0048476 "Holliday junction resolvase complex" evidence=IDA] |
| HP0884 | a.238.1.3 | BAR/IMD domain-like | BAR/IMD domain-like | IMD domain | All alpha proteins | [GO:0003674  "molecular_function" evidence=ND] [GO:0005575 "cellular_component"  evidence=ND] [GO:0008150 "biological_process" evidence=ND] |
| HP0885 | f.24.1.1 | Cytochrome c oxidase subunit I-like | Cytochrome c oxidase subunit I-like | Cytochrome c oxidase subunit I-like | Membrane and cell surface proteins and peptides | [GO:0003674 "molecular_function"  evidence=ND] [GO:0008150 "biological_process" evidence=ND] |
| HP0886 | c.26.1.1 | Adenine nucleotide alpha hydrolase-like | Nucleotidylyl transferase | Class I aminoacyl-tRNA synthetases (RS), catalytic domain | Alpha and beta proteins (a/b) | [GO:0004817 "cysteine-tRNA ligase  activity" evidence=ISS] [GO:0006423 "cysteinyl-tRNA aminoacylation"  evidence=ISS] |
| HP0887 | b.69.4.1 | 7-bladed beta-propeller | WD40 repeat-like | WD40 repeat | All beta proteins | [GO:0040007 "growth"  evidence=IMP] |
| HP0888 | c.37.1.12 | P-loop containing nucleoside triphosphate hydrolases | P-loop containing nucleoside triphosphate hydrolases | ABC transporter ATPase domain-like | Alpha and beta proteins (a/b) | [GO:0005381 "iron ion  transmembrane transporter activity" evidence=ISS] [GO:0006826 "iron  ion transport" evidence=ISS] [GO:0042626 "ATPase activity, coupled  to transmembrane movement of substances" evidence=ISS] |
| HP0889 | f.22.1.1 | ABC transporter involved in vitamin B12 uptake, BtuC | ABC transporter involved in vitamin B12 uptake, BtuC | ABC transporter involved in vitamin B12 uptake, BtuC | Membrane and cell surface proteins and peptides | [GO:0005381 "iron ion transmembrane transporter activity"  evidence=ISS] [GO:0006826 "iron ion transport" evidence=ISS] |
| HP0890 | c.2.1.2 | NAD(P)-binding Rossmann-fold domains | NAD(P)-binding Rossmann-fold domains | Tyrosine-dependent oxidoreductases | Alpha and beta proteins (a/b) | [GO:0008152 "metabolic process" evidence=ISS]  [GO:0016491 "oxidoreductase activity" evidence=ISS] |
| HP0891 | d.38.1.1 | Thioesterase/thiol ester dehydrase-isomerase | Thioesterase/thiol ester dehydrase-isomerase | 4HBT-like | Alpha and beta proteins (a+b) | [GO:0006631 "fatty acid metabolic process" evidence=ISS]  [GO:0016289 "CoA hydrolase activity" evidence=ISS] |
| HP0892 | d.298.1.2 | RelE-like | RelE-like | RelE-like | Alpha and beta proteins (a+b) | [GO:0003674 "molecular_function" evidence=ND] |
| HP0893 | d.129.3.6 | TBP-like | Bet v1-like | oligoketide cyclase/dehydrase-like | Alpha and beta proteins (a+b) | [GO:0004713 "protein tyrosine kinase activity"  evidence=IEA] [GO:0005524 "ATP binding" evidence=IEA] |
| HP0894 | d.298.1.2 | RelE-like | RelE-like | RelE-like | Alpha and beta proteins (a+b) | [GO:0003674 "molecular_function" evidence=ND] [GO:0005575  "cellular_component" evidence=ND] [GO:0008150 "biological_process"  evidence=ND] |
| HP0895 | a.6.1.3 | Putative DNA-binding domain | Putative DNA-binding domain | DNA-binding N-terminal domain of transcription activators | All alpha proteins | [GO:0005634  "nucleus" evidence=ISM] [GO:0003700 "sequence-specific DNA binding  transcription factor activity" evidence=ISS] [GO:0006355  "regulation of transcription, DNA-dependent" evidence=TAS] |
| HP0896 | f.4.1.1 | TRANSMEMBRANE BETA BAREELS | OMPA LIKE | OUTERMEMBRANE PROTEINS | Membrane and cell surface proteins and peptides |  |
| HP0897 | d.135.1.1 | The spindle assembly checkpoint protein mad2 | The spindle assembly checkpoint protein mad2 | The spindle assembly checkpoint protein mad2 | Alpha and beta proteins (a+b) |  |
| HP0898 | c.1.23.1 | TIM beta/alpha-barrel | FAD-linked oxidoreductase | Methylenetetrahydrofolate reductase | Alpha and beta proteins (a/b) | [GO:0003674  "molecular_function" evidence=ND] |
| HP0899 | b.40.14.1 | OB-fold | HupF/HypC-like | HupF/HypC-like | All beta proteins | [GO:0006461 "protein  complex assembly" evidence=ISS] [GO:0016530 "metallochaperone  activity" evidence=ISS] [GO:0051082 "unfolded protein binding"  evidence=ISS] |
| HP0900 | c.37.1.8 | P-loop containing nucleoside triphosphate hydrolases | P-loop containing nucleoside triphosphate hydrolases | G proteins | Alpha and beta proteins (a/b) | [GO:0000166 "nucleotide  binding" evidence=ISS] [GO:0006457 "protein folding" evidence=ISS]  [GO:0016530 "metallochaperone activity" evidence=ISS] |
| HP0901 | j.35.1.1 | Transmembrane helical fragments | Transmembrane helical fragments | Transmembrane helical fragments | Peptides |  |
| HP0902 | b.82.1.9 | Double-stranded beta-helix | RmlC-like cupins | TM1287-like | All beta proteins | [GO:0003674  "molecular_function" evidence=ND] [GO:0005575 "cellular_component"  evidence=ND] [GO:0008150 "biological_process" evidence=ND] |
| HP0903 | c.55.1.2 | Ribonuclease H-like motif | Actin-like ATPase domain | Acetokinase-like | Alpha and beta proteins (a/b) |  |
| HP0905 | c.37.1.10 | P-loop containing nucleoside triphosphate hydrolases | P-loop containing nucleoside triphosphate hydrolases | Nitrogenase iron protein-like | Alpha and beta proteins (a/b) | [GO:0006086  "acetyl-CoA biosynthetic process from pyruvate" evidence=ISS]  [GO:0008959 "phosphate acetyltransferase activity" evidence=ISS] |
| HP0906 |  |  |  |  |  | [GO:0003674  "molecular_function" evidence=ND] [GO:0005575 "cellular_component"  evidence=ND] [GO:0008150 "biological_process" evidence=ND] |
| HP0907 | b.1.4.1 | Immunoglobulin-like beta-sandwich | beta-Galactosidase/glucuronidase domain | beta-Galactosidase/glucuronidase domain | All beta proteins | [GO:0001539 "ciliary  or flagellar motility" evidence=ISS] [GO:0005198 "structural  molecule activity" evidence=ISS] [GO:0009424 "bacterial-type  flagellum hook" evidence=ISS] |
| HP0908 | b.152.1.1 | Flagellar hook protein flgE | Flagellar hook protein flgE | Flagellar hook protein flgE | All beta proteins | [GO:0001539 "ciliary or flagellar  motility" evidence=ISS] [GO:0005198 "structural molecule activity"  evidence=ISS] [GO:0009424 "bacterial-type flagellum hook"  evidence=ISS] |
| HP0909 | d.82.5.1 | N domain of copper amine oxidase-like | GK1464-like | GK1464-like | Alpha and beta proteins (a+b) |  |
| HP0910 | c.66.1.27 | S-adenosyl-L-methionine-dependent methyltransferases | S-adenosyl-L-methionine-dependent methyltransferases | DNA methylase TaqI, N-terminal domain | Alpha and beta proteins (a/b) | [GO:0009036 "Type II  site-specific deoxyribonuclease activity" evidence=ISS] [GO:0009307  "DNA restriction-modification system" evidence=ISS] |
| HP0911 | c.37.1.19 | P-loop containing nucleoside triphosphate hydrolases | P-loop containing nucleoside triphosphate hydrolases | Tandem AAA-ATPase domain | Alpha and beta proteins (a/b) | [GO:0004003  "ATP-dependent DNA helicase activity" evidence=ISS] [GO:0006281  "DNA repair" evidence=ISS] |
| HP0912 | f.4.1.1 | TRANSMEMBRANE BETA BAREELS | OMPA LIKE | OUTERMEMBRANE PROTEINS | Membrane and cell surface proteins and peptides |  |
| HP0913 | f.4.1.1 | TRANSMEMBRANE BETA BAREELS | OMPA LIKE | OUTERMEMBRANE PROTEINS | Membrane and cell surface proteins and peptides |  |
| HP0914 | f.4.3.3 | Transmembrane beta-barrels | Porins | Ligand-gated protein channel | Membrane and cell surface proteins and peptides |  |
| HP0915 | f.4.3.3 | Transmembrane beta-barrels | Porins | Ligand-gated protein channel | Membrane and cell surface proteins and peptides |  |
| HP0916 | f.4.3.3 | Transmembrane beta-barrels | Porins | Ligand-gated protein channel | Membrane and cell surface proteins and peptides | [GO:0015232 "heme  transporter activity" evidence=ISS] [GO:0015886 "heme transport"  evidence=ISS] |
| HP0917 | c.1.8.5 | TIM beta/alpha-barrel | (Trans)glycosidases | Type II chitinase | Alpha and beta proteins (a/b) |  |
| HP0918 | d.115.1.1 | YrdC/RibB | YrdC/RibB | YrdC-like | Alpha and beta proteins (a+b) | [GO:0003674  "molecular_function" evidence=ND] [GO:0005575 "cellular_component"  evidence=ND] [GO:0008150 "biological_process" evidence=ND] |
| HP0919 | d.142.1.2 | ATP-grasp | Glutathione synthetase ATP-binding domain-like | BC ATP-binding domain-like | Alpha and beta proteins (a+b) | [GO:0004088  "carbamoyl-phosphate synthase (glutamine-hydrolyzing) activity"  evidence=ISS] [GO:0009220 "pyrimidine ribonucleotide biosynthetic  process" evidence=ISS] |
| HP0919 | d.142.1.2 | ATP-grasp | Glutathione synthetase ATP-binding domain-like | BC ATP-binding domain-like | Alpha and beta proteins (a+b) | [GO:0004088  "carbamoyl-phosphate synthase (glutamine-hydrolyzing) activity"  evidence=ISS] [GO:0009220 "pyrimidine ribonucleotide biosynthetic  process" evidence=ISS] |
| HP0920 | a.127.1.1 | L-aspartase-like | L-aspartase-like | L-aspartase/fumarase | All alpha proteins | [GO:0003674 "molecular_function"  evidence=ND] [GO:0008150 "biological_process" evidence=ND] |
| HP0921 | d.81.1.1 | FwdE/GAPDH domain-like | Glyceraldehyde-3-phosphate dehydrogenase-like, C-terminal domain | GAPDH-like | Alpha and beta proteins (a+b) | [GO:0006094  "gluconeogenesis" evidence=ISS] [GO:0006096 "glycolysis"  evidence=ISS] [GO:0019682 "glyceraldehyde-3-phosphate metabolic  process" evidence=ISS] |
| HP0922 | b.81.1.7 | Single-stranded left-handed beta-helix | Trimeric LpxA-like enzymes | YdcK-like | All beta proteins | [GO:0008150 "biological_process"  evidence=ND] [GO:0005575 "cellular_component" evidence=ND]  [GO:0003674 "molecular_function" evidence=ND] |
| HP0923 | f.4.4.2 | Transmembrane beta-barrels | OMPT-like | Outer membrane adhesin/invasin OpcA | Membrane and cell surface proteins and peptides |  |
| HP0924 | d.80.1.1 | Tautomerase/MIF | Tautomerase/MIF | 4-oxalocrotonate tautomerase-like | Alpha and beta proteins (a+b) | [GO:0006725  "cellular aromatic compound metabolic process" evidence=ISS]  [GO:0016862 "intramolecular oxidoreductase activity,  interconverting keto- and enol-groups" evidence=ISS] |
| HP0925 | e.49.1.1 | Recombination protein RecR | Recombination protein RecR | Recombination protein RecR | Multi-domain proteins (alpha and beta) | [GO:0003824 "catalytic  activity" evidence=ISS] [GO:0006281 "DNA repair" evidence=ISS]  [GO:0006310 "DNA recombination" evidence=ISS] |
| HP0926 | d.265.1.4 | Pseudouridine synthase | Pseudouridine synthase | tRNA pseudouridine synthase TruD | Alpha and beta proteins (a+b) | [GO:0006400 "tRNA  modification" evidence=ISS] [GO:0009982 "pseudouridine synthase  activity" evidence=ISS] |
| HP0928 | d.96.1.1 | T-fold | Tetrahydrobiopterin biosynthesis enzymes-like | GTP cyclohydrolase I | Alpha and beta proteins (a+b) | [GO:0003934 "GTP cyclohydrolase I  activity" evidence=ISS] [GO:0006760 "folic acid-containing compound  metabolic process" evidence=ISS] |
| HP0929 | a.128.1.1 | Terpenoid synthases | Terpenoid synthases | Isoprenyl diphosphate synthases | All alpha proteins | [GO:0004337 "geranyltranstransferase  activity" evidence=ISS] [GO:0008299 "isoprenoid biosynthetic  process" evidence=ISS] |
| HP0930 | c.106.1.1 | SurE-like | SurE-like | SurE-like | Alpha and beta proteins (a/b) | [GO:0003993 "acid phosphatase  activity" evidence=ISS] [GO:0006950 "response to stress"  evidence=ISS] |
| HP0931 | d.58.7.1 | Ferredoxin-like | RNA-binding domain, RBD | Canonical RBD | Alpha and beta proteins (a+b) |  |
| HP0932 | a.28.1.1 | Acyl carrier protein-like | ACP-like | Acyl carrier protein(ACP) | All alpha proteins | [GO:0045892 "negative regulation of transcription,  DNA-dependent" evidence=IEA] [GO:0005634 "nucleus" evidence=IEA] |
| HP0933 | d.96.1.2 | T-fold | Tetrahydrobiopterin biosynthesis enzymes-like | 6-pyruvoyl tetrahydropterin synthase | Alpha and beta proteins (a+b) | [GO:0003824  "catalytic activity" evidence=ISS] [GO:0008152 "metabolic process"  evidence=ISS] |
| HP0934 | c.1.28.3 | TIM beta/alpha-barrel | Radical SAM enzymes | MoCo biosynthesis proteins | Alpha and beta proteins (a/b) | [GO:0003824 "catalytic  activity" evidence=ISS] [GO:0008152 "metabolic process"  evidence=ISS] |
| HP0935 | d.108.1.1 | Acyl-CoA N-acyltransferases (Nat) | Acyl-CoA N-acyltransferases (Nat) | N-acetyl transferase, NAT | Alpha and beta proteins (a+b) | [GO:0008080 "N-acetyltransferase activity" evidence=IEA]  [GO:0016740 "transferase activity" evidence=IEA] |
| HP0936 | f.54.1.1 | SNF-like | SNF-like | SNF-like | Membrane and cell surface proteins and peptides | [GO:0005215  "transporter activity" evidence=ISS] [GO:0006810 "transport"  evidence=ISS] |
| HP0937 | d.4.1.1 | His-Me finger endonucleases | His-Me finger endonucleases | HNH-motif | Alpha and beta proteins (a+b) |  |
| HP0938 | b.55.1.3 | PH domain-like barrel | PH domain-like | Ran-binding domain | All beta proteins |  |
| HP0939 | f.58.1.1 | MetI-like | MetI-like | MetI-like | Membrane and cell surface proteins and peptides | [GO:0006865  "amino acid transport" evidence=ISS] [GO:0042626 "ATPase activity,  coupled to transmembrane movement of substances" evidence=ISS] |
| HP0940 | c.94.1.1 | Periplasmic binding protein-like II | Periplasmic binding protein-like II | Phosphate binding protein-like | Alpha and beta proteins (a/b) | [GO:0006865 "amino acid transport" evidence=ISS] [GO:0016597 "amino  acid binding" evidence=ISS] [GO:0042626 "ATPase activity, coupled  to transmembrane movement of substances" evidence=ISS] |
| HP0941 | c.1.6.1 | TIM beta/alpha-barrel | PLP-binding barrel | Alanine racemase-like, N-terminal domain | Alpha and beta proteins (a/b) | [GO:0008784 "alanine  racemase activity" evidence=ISS] [GO:0009252 "peptidoglycan  biosynthetic process" evidence=ISS] |
| HP0942 | f.54.1.1 | SNF-like | SNF-like | SNF-like | Membrane and cell surface proteins and peptides | [GO:0015655  "alanine:sodium symporter activity" evidence=ISS] [GO:0015808  "L-alanine transport" evidence=ISS] |
| HP0943 | c.3.1.2 | FAD/NAD(P)-binding domain | FAD/NAD(P)-binding domain | FAD-linked reductases, N-terminal domain | Alpha and beta proteins (a/b) | [GO:0008152 "metabolic  process" evidence=ISS] [GO:0016491 "oxidoreductase activity"  evidence=ISS] |
| HP0944 | d.79.1.1 | Bacillus chorismate mutase-like | YjgF-like | YjgF/L-PSP | Alpha and beta proteins (a+b) | [GO:0004521  "endoribonuclease activity" evidence=ISS] [GO:0006402 "mRNA  catabolic process" evidence=ISS] |
| HP0945 | c.45.1.1 | (Phosphotyrosine protein) phosphatases II | (Phosphotyrosine protein) phosphatases II | Dual specificity phosphatase-like | Dual specificity phosphatase-like | [GO:0003674  "molecular_function" evidence=ND] [GO:0005575 "cellular_component"  evidence=ND] [GO:0008150 "biological_process" evidence=ND] |
| HP0947 | d.79.9.1 | Bacillus chorismate mutase-like | BB2672-like | BB2672-like | Alpha and beta proteins (a+b) |  |
| HP0948 | a.118.8.2 | alpha-alpha superhelix | TPR-like | Transcription factor MalT domain III | All alpha proteins | [GO:0003674  "molecular_function" evidence=ND] [GO:0005575 "cellular_component"  evidence=ND] [GO:0008150 "biological_process" evidence=ND] |
| HP0949 | c.116.1.3 | alpha/beta knot | alpha/beta knot | alpha/beta knot | Alpha and beta proteins (a/b) | [GO:0003674  "molecular_function" evidence=ND] [GO:0005575 "cellular_component"  evidence=ND] [GO:0008150 "biological_process" evidence=ND] |
| HP0950 | c.14.1.4 | ClpP/crotonase | ClpP/crotonase | Biotin dependent carboxylase carboxyltransferase domain | Alpha and beta proteins (a/b) | [GO:0005515 "protein  binding" evidence=IPI] |
| HP0951 | g.45.1.2 | ArfGap/RecO-like zinc finger | ArfGap/RecO-like zinc finger | RecO C-terminal domain-like | Small proteins | [GO:0003674  "molecular_function" evidence=ND] [GO:0005575 "cellular_component"  evidence=ND] [GO:0008150 "biological_process" evidence=ND] |
| HP0952 | c.51.5.1 | Anticodon-binding domain-like | CinA-like | CinA-like | Alpha and beta proteins (a/b) | [GO:0008150  "biological_process" evidence=ND] |
| HP0953 | c.123.1.1 | CoA-transferase family III (CaiB/BaiF) | CoA-transferase family III (CaiB/BaiF) | CoA-transferase family III (CaiB/BaiF) | Alpha and beta proteins (a/b) |  |
| HP0954 | d.90.1.1 | FMN-dependent nitroreductase-like | FMN-dependent nitroreductase-like | NADH oxidase/flavin reductase | Alpha and beta proteins (a+b) | [GO:0009055 "electron carrier activity"  evidence=ISS] |
| HP0955 |  |  |  |  |  | [GO:0008961 "phosphatidylglycerol-prolipoprotein diacylglyceryl  transferase activity" evidence=ISS] [GO:0009249 "protein  lipoylation" evidence=ISS] [GO:0016020 "membrane" evidence=ISS] |
| HP0956 | d.265.1.3 | Pseudouridine synthase | Pseudouridine synthase | Pseudouridine synthase RsuA/RluD | Alpha and beta proteins (a+b) | [GO:0009451  "RNA modification" evidence=ISS] [GO:0009982 "pseudouridine  synthase activity" evidence=ISS] |
| HP0957 | c.87.1.3 | UDP-Glycosyltransferase/glycogen phosphorylase | UDP-Glycosyltransferase/glycogen phosphorylase | UDP-N-acetylglucosamine 2-epimerase | Alpha and beta proteins (a/b) | [GO:0009244 "lipopolysaccharide core region biosynthetic process"  evidence=ISS] [GO:0016740 "transferase activity" evidence=ISS] |
| HP0958 | a.238.1.4 | BAR/IMD domain-like | BAR/IMD domain-like | FCH domain | All alpha proteins | [GO:0005515 "protein binding" evidence=IPI] |
| HP0959 | c.135.1.1 | NIF3 (NGG1p interacting factor 3)-like | NIF3 (NGG1p interacting factor 3)-like | NIF3 (NGG1p interacting factor 3)-like | Alpha and beta proteins (a/b) | [GO:0003674  "molecular_function" evidence=ND] [GO:0005575 "cellular_component"  evidence=ND] [GO:0008150 "biological_process" evidence=ND] |
| HP0960 | d.104.1.1 | Class II aaRS and biotin synthetases | Class II aaRS and biotin synthetases | Class II aminoacyl-tRNA synthetase (aaRS)-like, catalytic domain | Alpha and beta proteins (a+b) | [GO:0004820  "glycine-tRNA ligase activity" evidence=ISS] [GO:0006426  "glycyl-tRNA aminoacylation" evidence=ISS] [GO:0009345  "glycine-tRNA ligase complex" evidence=ISS] |
| HP0961 | c.2.1.6 | NAD(P)-binding Rossmann-fold domains | NAD(P)-binding Rossmann-fold domains | 6-phosphogluconate dehydrogenase-like, N-terminal domain | Alpha and beta proteins (a/b) | [GO:0006072 "glycerol-3-phosphate metabolic process" evidence=ISS]  [GO:0008654 "phospholipid biosynthetic process" evidence=ISS]  [GO:0047952 "glycerol-3-phosphate dehydrogenase [NAD(P)+] activity"  evidence=ISS] |
| HP0962 | a.28.1.1 | Acyl carrier protein-like | ACP-like | Acyl-carrier protein (ACP) | All alpha proteins | [GO:0000036 "ACP  phosphopantetheine attachment site binding involved in fatty acid  biosynthetic process" evidence=ISS] [GO:0006633 "fatty acid  biosynthetic process" evidence=ISS] |
| HP0963 | a.238.1.4 | BAR/IMD domain-like | BAR/IMD domain-like | FCH domain | All alpha proteins | [GO:0008150 "biological_process"  evidence=ND] |
| HP0964 |  |  |  |  |  | [GO:0016319 "mushroom body development"  evidence=IMP;TAS] [GO:0007058 "spindle assembly involved in female  meiosis II" evidence=IMP] [GO:0051233 "spindle midzone"  evidence=IDA] [GO:0005515 "protein binding" evidence=IPI] |
| HP0965 | a.118.1.1 | alpha-alpha superhelix | ARM repeat | Armadillo repeat | All alpha proteins | [GO:0008150 "biological_process"  evidence=ND] |
| HP0966 | a.118.1.1 | alpha-alpha superhelix | ARM repeat | Armadillo repeat | All alpha proteins | [GO:0003674 "molecular_function" evidence=ND] [GO:0005575  "cellular_component" evidence=ND] [GO:0008150 "biological_process"  evidence=ND] |
| HP0967 | d.79.4.1 | Bacillus chorismate mutase-like | PurM N-terminal domain-like | PurM N-terminal domain-like | Alpha and beta proteins (a+b) |  |
| HP0969 | f.35.1.1 | Multidrug efflux transporter AcrB transmembrane domain | Multidrug efflux transporter AcrB transmembrane domain | Multidrug efflux transporter AcrB transmembrane domain | Membrane and cell surface proteins and peptides | [GO:0010038  "response to metal ion" evidence=ISS] [GO:0030001 "metal ion  transport" evidence=ISS] [GO:0046873 "metal ion transmembrane  transporter activity" evidence=ISS] |
| HP0970 | f.46.1.1 | HlyD-like secretion proteins | HlyD-like secretion proteins | HlyD-like secretion proteins | Membrane and cell surface proteins and peptides | [GO:0008565  "protein transporter activity" evidence=ISS] [GO:0009306 "protein  secretion" evidence=ISS] |
| HP0971 | f.5.1.1 | Outer membrane efflux proteins (OEP) | Outer membrane efflux proteins (OEP) | Outer membrane efflux proteins (OEP) | Membrane and cell surface proteins and peptides | [GO:0006810 "transport"  evidence=ISS] [GO:0015562 "efflux transmembrane transporter  activity" evidence=ISS] |
| HP0972 | d.104.1.1 | Class II aaRS and biotin synthetases | Class II aaRS and biotin synthetases | Class II aminoacyl-tRNA synthetase (aaRS)-like, catalytic domain | Alpha and beta proteins (a+b) | [GO:0004820  "glycine-tRNA ligase activity" evidence=ISS] [GO:0006426  "glycyl-tRNA aminoacylation" evidence=ISS] |
| HP0973 | d.2.1.6 | Lysozyme-like | Lysozyme-like | Bacterial muramidase, catalytic domain | Alpha and beta proteins (a+b) | [GO:0008270  "zinc ion binding" evidence=IEA] [GO:0019787 "small conjugating  protein ligase activity" evidence=ISM] [GO:0030998 "linear element"  evidence=IDA] |
| HP0974 | c.105.1.1 | Bisphosphoglycerate-independent phosphoglycerate mutase, substrate-binding domain | Bisphosphoglycerate-independent phosphoglycerate mutase, substrate-binding domain | Bisphosphoglycerate-independent phosphoglycerate mutase, substrate-binding domain | Alpha and beta proteins (a/b) | [GO:0004619 "phosphoglycerate mutase activity"  evidence=ISS] [GO:0006096 "glycolysis" evidence=ISS] |
| HP0975 | a.137.12.1 | Non-globular all-alpha subunits of globular proteins | Glu-tRNAGln amidotransferase C subunit | Glu-tRNAGln amidotransferase C subunit | All alpha proteins | [GO:0006422 "aspartyl-tRNA aminoacylation" evidence=ISS]  [GO:0006424 "glutamyl-tRNA aminoacylation" evidence=ISS] |
| HP0976 | c.67.1.4 | PLP-dependent transferase-like | PLP-dependent transferase-like | GABA-aminotransferase-like | Alpha and beta proteins (a/b) | [GO:0004015  "adenosylmethionine-8-amino-7-oxononanoate transaminase activity"  evidence=ISS] [GO:0009102 "biotin biosynthetic process"  evidence=ISS] |
| HP0977 | a.223.1.2 | Triger factor/SurA peptide-binding domain-like | Triger factor/SurA peptide-binding domain-like | Porin chaperone SurA, peptide-binding domain | All alpha proteins | [GO:0003824  "catalytic activity" evidence=ISS] [GO:0008152 "metabolic process"  evidence=ISS] |
| HP0978 | c.55.1.1 | Ribonuclease H-like motif | Actin-like ATPase domain | Actin/HSP70 | Alpha and beta proteins (a/b) | [GO:0000910  "cytokinesis" evidence=ISS] [GO:0005524 "ATP binding" evidence=ISS] |
| HP0979 | c.32.1.1 | Tubulin nucleotide-binding domain-like | Tubulin nucleotide-binding domain-like | Tubulin, GTPase domain | Alpha and beta proteins (a/b) | [GO:0000910  "cytokinesis" evidence=ISS] [GO:0003924 "GTPase activity"  evidence=ISS] |
| HP0980 | f.20.1.1 | Clc chloride channel | Clc chloride channel | Clc chloride channel | Membrane and cell surface proteins and peptides | [GO:0004222  "metalloendopeptidase activity" evidence=ISS] [GO:0006508  "proteolysis" evidence=ISS] |
| HP0981 |  | RNA polymerase | RNA polymerase | RNA polymerase | Low resolution protein structures | [GO:0006308 "DNA  catabolic process" evidence=ISS] [GO:0008855 "exodeoxyribonuclease  VII activity" evidence=ISS] [GO:0009318 "exodeoxyribonuclease VII  complex" evidence=ISS] |
| HP0982 | d.15.7.1 | beta-Grasp (ubiquitin-like) | Immunoglobulin-binding domains | Immunoglobulin-binding domains | Alpha and beta proteins (a+b) |  |
| HP0983 | d.58.43.1 | Ferredoxin-like | Mechanosensitive channel protein MscS (YggB), C-terminal domain | Mechanosensitive channel protein MscS (YggB), C-terminal domain | Alpha and beta proteins (a+b) | [GO:0003674 "molecular_function" evidence=ND] [GO:0005575  "cellular_component" evidence=ND] [GO:0008150 "biological_process"  evidence=ND] |
| HP0984 | a.118.1.1 | alpha-alpha superhelix | ARM repeat | Armadillo repeat | All alpha proteins |  |
| HP0985 | b.1.18.8 | Immunoglobulin-like beta-sandwich | E set domains | RhoGDI-like | All beta proteins |  |
| HP0986 | c.52.1.26 | Restriction endonuclease-like | Restriction endonuclease-like | Hypothetical protein VC1899 | Alpha and beta proteins (a/b) |  |
| HP0987 | a.118.8.2 | alpha-alpha superhelix | TPR-like | Transcription factor MalT domain III | All alpha proteins |  |
| HP0988 | d.58.57.1 | Ferredoxin-like | Transposase IS200-like | Transposase IS200-like | Alpha and beta proteins (a+b) | [GO:0004803 "transposase  activity" evidence=ISS] [GO:0006313 "transposition, DNA-mediated"  evidence=ISS] |
| HP0989 | b.113.1.1 | N-terminal domain of MutM-like DNA repair proteins | N-terminal domain of MutM-like DNA repair proteins | N-terminal domain of MutM-like DNA repair proteins | All beta proteins | [GO:0004803 "transposase activity"  evidence=ISS] [GO:0006313 "transposition, DNA-mediated"  evidence=ISS] |
| HP0990 | f.1.3.1 | Toxins' membrane translocation domains | delta-Endotoxin (insectocide), N-terminal domain | delta-Endotoxin (insectocide), N-terminal domain | Membrane and cell surface proteins and peptides | [GO:0001525 "angiogenesis" evidence=ISO] [GO:0001933  "negative regulation of protein phosphorylation" evidence=ISO]  [GO:0002902 "regulation of B cell apoptotic process" evidence=ISO]  [GO:0004438 "phosphatidylinositol-3-phosphatase activity"  evidence=ISO] |
| HP0991 | b.1.1.4 | Immunoglobulin-like beta-sandwich | Immunoglobulin | I set domains | All beta proteins |  |
| HP0992 | c.37.1.9 | P-loop containing nucleoside triphosphate hydrolases | P-loop containing nucleoside triphosphate hydrolases | Motor proteins | Alpha and beta proteins (a/b) | [GO:0003674 "molecular_function" evidence=ND] [GO:0005634  "nucleus" evidence=ISM] [GO:0008150 "biological_process"  evidence=ND] |
| HP0993 | d.108.1.1 | Acyl-CoA N-acyltransferases (Nat) | Acyl-CoA N-acyltransferases (Nat) | N-acetyl transferase, NAT | Alpha and beta proteins (a+b) | [GO:0005522  "profilin binding" evidence=ISS] [GO:0030036 "actin cytoskeleton  organization" evidence=IEA] [GO:0016043 "cellular component  organization" evidence=IEA] [GO:0003779 "actin binding"  evidence=IEA] |
| HP0994 | a.160.1.5 | PAP/OAS1 substrate-binding domain [ | PAP/OAS1 substrate-binding domain [ | AadK C-terminal domain-like | All alpha proteins | [GO:0008150  "biological_process" evidence=ND] |
| HP0995 | d.92.1.5 | Zincin-like | Metalloproteases ("zincins"), catalytic domain | Neurolysin-like | Alpha and beta proteins (a+b) | [GO:0006310 "DNA recombination" evidence=ISS] [GO:0009009  "site-specific recombinase activity" evidence=ISS] [GO:0015074 "DNA  integration" evidence=ISS] |
| HP0996 | c.72.2.2 | Ribokinase-like | MurD-like peptide ligases, catalytic domain | Folylpolyglutamate synthetase | Alpha and beta proteins (a/b) | [GO:0000226 "microtubule cytoskeleton organization"  evidence=IEA] [GO:0003779 "actin binding" evidence=IEA] [GO:0005783  "endoplasmic reticulum" evidence=IEA] |
| HP0997 | b.113.1.1 | N-terminal domain of MutM-like DNA repair proteins | N-terminal domain of MutM-like DNA repair proteins | N-terminal domain of MutM-like DNA repair proteins | All beta proteins | [GO:0004803 "transposase activity"  evidence=ISS] [GO:0006313 "transposition, DNA-mediated"  evidence=ISS] |
| HP0998 | d.58.57.1 | Ferredoxin-like | Transposase IS200-like | Transposase IS200-like | Alpha and beta proteins (a+b) | [GO:0004803 "transposase  activity" evidence=ISS] [GO:0006313 "transposition, DNA-mediated"  evidence=ISS] |
| HP0999 | d.211.2.1 | beta-hairpin-alpha-hairpin repeat | Plakin repeat | Plakin repeat | Alpha and beta proteins (a+b) | [GO:0003713 "transcription coactivator  activity" evidence=IEA] [GO:0003677 "DNA binding" evidence=IEA]  [GO:0006355 "regulation of transcription, DNA-dependent"  evidence=IEA] |
| HP1000 | c.37.1.10 | P-loop containing nucleoside triphosphate hydrolases | P-loop containing nucleoside triphosphate hydrolases | Nitrogenase iron protein-like | Alpha and beta proteins (a/b) | [GO:0003674 "molecular_function" evidence=ND] [GO:0005575  "cellular_component" evidence=ND] [GO:0008150 "biological_process"  evidence=ND] |
| HP1001 | d.58.18.4 | Ferredoxin-like | ACT-like | Nickel responsive regulator NikR, C-terminal domain | Alpha and beta proteins (a+b) | [GO:0006396 "RNA processing" evidence=IEA] [GO:0003723 "RNA  binding" evidence=IEA] [GO:0003676 "nucleic acid binding"  evidence=IEA] [GO:0000166 "nucleotide binding" evidence=IEA]  [GO:0005575 "cellular_component" evidence=ND] |
| HP1002 | d.17.1.2 | Cystatin-like | Cystatin/monellin | Cystatins | Alpha and beta proteins (a+b) | [GO:0003674  "molecular_function" evidence=ND] [GO:0008150 "biological_process"  evidence=ND] [GO:0020011 "apicoplast" evidence=RCA] |
| HP1003 | f.24.1.1 | Cytochrome c oxidase subunit I-like | Cytochrome c oxidase subunit I-like | Cytochrome c oxidase subunit I-like | Membrane and cell surface proteins and peptides | [GO:0009908 "flower  development" evidence=IMP] [GO:0007020 "microtubule nucleation"  evidence=RCA] |
| HP1004 | d.89.1.5 | Origin of replication-binding domain, RBD-like | Origin of replication-binding domain, RBD-like | Relaxase domain | Alpha and beta proteins (a+b) | [GO:0003674  "molecular_function" evidence=ND] [GO:0008150 "biological_process"  evidence=ND] [GO:0020011 "apicoplast" evidence=RCA] |
| HP1005 | a.134.1.1 | Fungal elicitin | Fungal elicitin | Fungal elicitin | All alpha proteins | [GO:0005575 "cellular_component" evidence=ND]  [GO:0003674 "molecular_function" evidence=ND] [GO:0008150  "biological_process" evidence=ND] |
| HP1006 | c.37.1.11 | P-loop containing nucleoside triphosphate hydrolases | P-loop containing nucleoside triphosphate hydrolases | RecA protein-like (ATPase-domain) | Alpha and beta proteins (a/b) | [GO:0000746  "conjugation" evidence=ISS] |
| HP1008 | d.58.57.1 | Ferredoxin-like | Transposase IS200-like | Transposase IS200-like | Alpha and beta proteins (a+b) | [GO:0004803 "transposase  activity" evidence=ISS] [GO:0006313 "transposition, DNA-mediated"  evidence=ISS] |
| HP1009 | d.163.1.1 | DNA breaking-rejoining enzymes | DNA breaking-rejoining enzymes | Lambda integrase-like, catalytic core | Alpha and beta proteins (a+b) | [GO:0009009  "site-specific recombinase activity" evidence=ISS] [GO:0015074 "DNA  integration" evidence=ISS] |
| HP1010 | d.322.1.2 | PHP14-like | PHP14-like | PPK middle domain-like | Alpha and beta proteins (a+b) | [GO:0006797 "polyphosphate metabolic  process" evidence=ISS] [GO:0008976 "polyphosphate kinase activity"  evidence=ISS] [GO:0009358 "polyphosphate kinase complex"  evidence=ISS] |
| HP1011 | c.1.4.1 | TIM beta/alpha-barrel | FMN-linked oxidoreductases | FMN-linked oxidoreductases | Alpha and beta proteins (a/b) | [GO:0004158  "dihydroorotate oxidase activity" evidence=ISS] [GO:0009220  "pyrimidine ribonucleotide biosynthetic process" evidence=ISS] |
| HP1012 | d.185.1.1 | LuxS/MPP-like metallohydrolase | LuxS/MPP-like metallohydrolase | MPP-like | Alpha and beta proteins (a+b) | [GO:0004222 "metalloendopeptidase  activity" evidence=ISS] [GO:0006508 "proteolysis" evidence=ISS] |
| HP1013 | c.1.10.1 | TIM beta/alpha-barrel | Aldolase | Class I aldolase | Alpha and beta proteins (a/b) | [GO:0008840  "4-hydroxy-tetrahydrodipicolinate synthase" evidence=ISS]  [GO:0009089 "lysine biosynthetic process via diaminopimelate"  evidence=ISS] |
| HP1014 | c.2.1.2 | NAD(P)-binding Rossmann-fold domains | NAD(P)-binding Rossmann-fold domains | Tyrosine-dependent oxidoreductases | Alpha and beta proteins (a/b) | [GO:0008152 "metabolic process" evidence=ISS]  [GO:0016491 "oxidoreductase activity" evidence=ISS] |
| HP1015 | b.100.1.1 | Sortase | Sortase | Sortase | All beta proteins |  |
| HP1016 | d.42.1.2 | POZ domain | POZ domain | Tetramerization domain of potassium channels | Alpha and beta proteins (a+b) | [GO:0008444 "CDP-diacylglycerol-glycerol-3-phosphate  3-phosphatidyltransferase activity" evidence=ISS] [GO:0008654  "phospholipid biosynthetic process" evidence=ISS] |
| HP1017 | f.54.1.1 | SNF-like | SNF-like | SNF-like | Membrane and cell surface proteins and peptides | [GO:0006525 "arginine  metabolic process" evidence=IDA] |
| HP1018 | c.37.1.21 | P-loop containing nucleoside triphosphate hydrolases | P-loop containing nucleoside triphosphate hydrolases | Plasmid maintenance system epsilon/zeta, toxin zeta subunit | Alpha and beta proteins (a/b) |  |
| HP1019 | b.47.1.1 | Trypsin-like serine proteases | Trypsin-like serine proteases | Prokaryotic proteases | All beta proteins | [GO:0004252 "serine-type  endopeptidase activity" evidence=ISS] [GO:0006508 "proteolysis"  evidence=ISS] [GO:0006950 "response to stress" evidence=ISS]  [GO:0030288 "outer membrane-bounded periplasmic space"  evidence=ISS] |
| HP1020 | c.68.1.13 | Nucleotide-diphospho-sugar transferases | Nucleotide-diphospho-sugar transferases | Cytidylytransferase | Alpha and beta proteins (a/b) | [GO:0008685  "2-C-methyl-D-erythritol 2,4-cyclodiphosphate synthase activity"  evidence=ISS] [GO:0019288 "isopentenyl diphosphate biosynthetic  process, mevalonate-independent pathway" evidence=ISS] |
| HP1021 | a.4.6.1 | DNA/RNA-binding 3-helical bundle | C-terminal effector domain of the bipartite response regulators | PhoB-like | All alpha proteins | [GO:0000156  "phosphorelay response regulator activity" evidence=ISS]  [GO:0000160 "phosphorelay signal transduction system" evidence=ISS]  [GO:0003677 "DNA binding" evidence=ISS] |
| HP1022 | c.120.1.2 | PIN domain-like | PIN domain-like | 5' to 3' exonuclease catalytic domain | Alpha and beta proteins (a/b) | [GO:0003887 "DNA-directed DNA  polymerase activity" evidence=ISS] [GO:0006260 "DNA replication"  evidence=ISS] [GO:0009360 "DNA polymerase III complex"  evidence=ISS] |
| HP1023 | f.4.3.3 | Transmembrane beta-barrels | Porins | Ligand-gated protein channel | Membrane and cell surface proteins and peptides |  |
| HP1024 | b.4.1.1 | HSP40/DnaJ peptide-binding domain | HSP40/DnaJ peptide-binding domain | HSP40/DnaJ peptide-binding domain | All beta proteins | [GO:0005515  "protein binding" evidence=IPI] |
| HP1025 | a.6.1.3 | Putative DNA-binding domain | Putative DNA-binding domain | DNA-binding N-terminal domain of transcription activators | All alpha proteins | [GO:0005515 "protein  binding" evidence=IPI] |
| HP1026 | a.80.1.2 | post-AAA+ oligomerization domain-like | post-AAA+ oligomerization domain-like | MgsA/YrvN C-terminal domain-like | All alpha proteins | [GO:0016887 "ATPase  activity" evidence=ISS] [GO:0019538 "protein metabolic process"  evidence=ISS] |
| HP1027 | a.4.5.42 | DNA/RNA-binding 3-helical bundle | "Winged helix" DNA-binding domain | FUR-like | All alpha proteins | [GO:0042802 "identical  protein binding" evidence=IPI] |
| HP1028 | b.61.1.1 | Streptavidin-like | Avidin/streptavidin | Avidin/streptavidin | All beta proteins | [GO:0003674  "molecular_function" evidence=ND] [GO:0005575 "cellular_component"  evidence=ND] [GO:0008150 "biological_process" evidence=ND] |
| HP1029 | b.82.2.7 | Double-stranded beta-helix | Clavaminate synthase-like | YhcH-like | All beta proteins | [GO:0003674 "molecular_function"  evidence=ND] [GO:0005575 "cellular_component" evidence=ND]  [GO:0008150 "biological_process" evidence=ND] |
| HP1030 | b.139.1.1 | Surface presentation of antigens (SPOA) | Surface presentation of antigens (SPOA) | Surface presentation of antigens (SPOA) | All beta proteins | [GO:0001539 "ciliary  or flagellar motility" evidence=ISS] [GO:0003774 "motor activity"  evidence=ISS] [GO:0005198 "structural molecule activity"  evidence=ISS] |
| HP1031 | d.252.1.1 | CheC-like | CheC-like | CheC-like | Alpha and beta proteins (a+b) | [GO:0001539 "ciliary  or flagellar motility" evidence=ISS] [GO:0003774 "motor activity"  evidence=ISS] [GO:0005198 "structural molecule activity"  evidence=ISS] |
|  | b.139.1.1 | Surface presentation of antigens (SPOA) | Surface presentation of antigens (SPOA) | Surface presentation of antigens (SPOA) | All beta proteins | [GO:0001539 "ciliary  or flagellar motility" evidence=ISS] [GO:0003774 "motor activity"  evidence=ISS] [GO:0005198 "structural molecule activity"  evidence=ISS] |
| HP1032 | a.177.1.1 | Sigma2 domain of RNA polymerase sigma factors | Sigma2 domain of RNA polymerase sigma factors | Sigma2 domain of RNA polymerase sigma factors | All alpha proteins | [GO:0006352  "DNA-dependent transcription, initiation" evidence=ISS] [GO:0016987  "sigma factor activity" evidence=ISS] |
| HP1033 | a.127.1.2 | L-aspartase-like | L-aspartase-like | HAL/PAL-like | All alpha proteins |  |
| HP1034 | c.37.1.10 | P-loop containing nucleoside triphosphate hydrolases | P-loop containing nucleoside triphosphate hydrolases | Nitrogenase iron protein-like | Alpha and beta proteins (a/b) | [GO:0000910 "cytokinesis"  evidence=ISS] [GO:0016887 "ATPase activity" evidence=ISS] |
| HP1035 | c.37.1.10 | P-loop containing nucleoside triphosphate  hydrolases | P-loop containing nucleoside triphosphate  hydrolases | Nitrogenase iron protein-like | Alpha and beta proteins (a/b) | [GO:0001539 "ciliary  or flagellar motility" evidence=ISS] [GO:0005525 "GTP binding"  evidence=ISS] [GO:0009296 "flagellum assembly" evidence=ISS] |
| HP1036 | d.58.30.1 | Ferredoxin-like | 6-hydroxymethyl-7,8-dihydropterin pyrophosphokinase, HPPK | 6-hydroxymethyl-7,8-dihydropterin pyrophosphokinase, HPPK | Alpha and beta proteins (a+b) | [GO:0003848 "2-amino-4-hydroxy-6-hydroxymethyldihydropteridine  diphosphokinase activity" evidence=ISS] [GO:0009396 "folic  acid-containing compound biosynthetic process" evidence=ISS] |
| HP1037 | d.127.1.1 | Creatinase/aminopeptidase | Creatinase/aminopeptidase | Creatinase/aminopeptidase | Alpha and beta proteins (a+b) | [GO:0006508  "proteolysis" evidence=ISS] [GO:0008235 "metalloexopeptidase  activity" evidence=ISS] |
| HP1038 | c.23.13.1 | Flavodoxin-like | Type II 3-dehydroquinate dehydratase | Type II 3-dehydroquinate dehydratase | Alpha and beta proteins (a/b) | [GO:0003855  "3-dehydroquinate dehydratase activity" evidence=ISS] [GO:0009423  "chorismate biosynthetic process" evidence=ISS] |
| HP1039 | d.272.1.1 | Dystroglycan, domain 2 | Dystroglycan, domain 2 | Dystroglycan, domain 2 | Alpha and beta proteins (a+b) | [GO:0003674 "molecular_function" evidence=ND]  [GO:0008150 "biological_process" evidence=ND] |
| HP1040 | a.29.3.1; | Bromodomain-like | Acyl-CoA dehydrogenase C-terminal domain-like | Medium chain acyl-CoA dehydrogenase-like, C-terminal domain | All alpha proteins | [GO:0003735 "structural  constituent of ribosome" evidence=ISS] [GO:0006412 "translation"  evidence=ISS] |
| HP1041 | c.82.1.1 | ALDH-like | ALDH-like | ALDH-like | Alpha and beta proteins (a/b) | [GO:0005515 "protein  binding" evidence=IPI] |
| HP1042 | c.107.1.1 | DHH phosphoesterases | DHH phosphoesterases | Manganese-dependent inorganic pyrophosphatase (family II) | Alpha and beta proteins (a/b) | [GO:0003674  "molecular_function" evidence=ND] [GO:0005575 "cellular_component"  evidence=ND] [GO:0008150 "biological_process" evidence=ND] |
| HP1043 | c.23.1.1 | Flavodoxin-like | CheY-like | CheY-related | Alpha and beta proteins (a/b) | [GO:0000156  "phosphorelay response regulator activity" evidence=ISS]  [GO:0000160 "phosphorelay signal transduction system" evidence=ISS]  [GO:0003677 "DNA binding" evidence=ISS] |
| HP1044 | d.159.1.11 | Metallo-dependent phosphatases | Metallo-dependent phosphatases | GpdQ-like | Alpha and beta proteins (a+b) | [GO:0004722  "protein serine/threonine phosphatase activity" evidence=ISS]  [GO:0006470 "protein dephosphorylation" evidence=ISS] |
|  | d.159.1.6 | Metallo-dependent phosphatases | Metallo-dependent phosphatases | TT1561-like | Alpha and beta proteins (a+b) | [GO:0004722  "protein serine/threonine phosphatase activity" evidence=ISS]  [GO:0006470 "protein dephosphorylation" evidence=ISS] |
| HP1045 | e.23.1.1 | Acetyl-CoA synthetase-like | Acetyl-CoA synthetase-like | Acetyl-CoA synthetase-like | Multi-domain proteins (alpha and beta) | [GO:0003987  "acetate-CoA ligase activity" evidence=ISS] [GO:0006090 "pyruvate  metabolic process" evidence=ISS] [GO:0006091 "generation of  precursor metabolites and energy" evidence=ISS] [GO:0006094  "gluconeogenesis" evidence=ISS] |
| HP1046 | d.52.4.1 | Alpha-lytic protease prodomain-like | YhbC-like, N-terminal domain | YhbC-like, N-terminal domain | Alpha and beta proteins (a+b) | [GO:0003674  "molecular_function" evidence=ND] [GO:0005575 "cellular_component"  evidence=ND] [GO:0008150 "biological_process" evidence=ND] |
| HP1047 | d.52.7.1 | Alpha-lytic protease prodomain-like | Ribosome-binding factor A, RbfA | Ribosome-binding factor A, RbfA | Alpha and beta proteins (a+b) | [GO:0006396 "RNA processing"  evidence=ISS] [GO:0019843 "rRNA binding" evidence=ISS] |
| HP1048 | c.37.1.8 | P-loop containing nucleoside triphosphate hydrolases | P-loop containing nucleoside triphosphate hydrolases | G proteins | Alpha and beta proteins (a/b) | [GO:0003743  "translation initiation factor activity" evidence=ISS] [GO:0006413  "translational initiation" evidence=ISS] |
| HP1049 | d.192.1.1 | YlxR-like | YlxR-like | YlxR-like | Alpha and beta proteins (a+b) | [GO:0003674  "molecular_function" evidence=ND] [GO:0005575 "cellular_component"  evidence=ND] [GO:0008150 "biological_process" evidence=ND] |
| HP1050 | d.14.1.5 | Ribosomal protein S5 domain 2-like | Ribosomal protein S5 domain 2-like | GHMP Kinase, N-terminal domain | Alpha and beta proteins (a+b) | [GO:0004413 "homoserine kinase  activity" evidence=ISS] [GO:0009088 "threonine biosynthetic  process" evidence=ISS] |
| HP1051 | c.55.1.9 | Ribonuclease H-like motif | Actin-like ATPase domain | YeaZ-like | Alpha and beta proteins (a/b) | [GO:0003674  "molecular_function" evidence=ND] [GO:0005575 "cellular_component"  evidence=ND] [GO:0008150 "biological_process" evidence=ND] |
| HP1052 | d.14.1.7 | Ribosomal protein S5 domain 2-like | Ribosomal protein S5 domain 2-like | UDP-3-O-[3-hydroxymyristoyl] N-acetylglucosamine deacetylase LpxC | Alpha and beta proteins (a+b) | [GO:0008759 "UDP-3-O-[3-hydroxymyristoyl] N-acetylglucosamine  deacetylase activity" evidence=ISS] [GO:0009245 "lipid A  biosynthetic process" evidence=ISS] |
| HP1053 | b.80.3.1 | Single-stranded right-handed beta-helix | Cell-division inhibitor MinC, C-terminal domain | Cell-division inhibitor MinC, C-terminal domain | All beta proteins | [GO:0004857 "enzyme  inhibitor activity" evidence=ISS] [GO:0007105 "cytokinesis, site  selection" evidence=ISS] |
| HP1054 | b.84.3.2 | Barrel-sandwich hybrid | Duplicated hybrid motif | Peptidoglycan hydrolase LytM | All beta proteins | [GO:0004222 "metalloendopeptidase  activity" evidence=ISS] [GO:0006508 "proteolysis" evidence=ISS] |
|  | b.84.3.2 | Barrel-sandwich hybrid | Duplicated hybrid motif | Peptidoglycan hydrolase LytM | All beta proteins | [GO:0004222 "metalloendopeptidase  activity" evidence=ISS] [GO:0006508 "proteolysis" evidence=ISS] |
| HP1055 | f.4.3.2 | Transmembrane beta-barrels | Porins | Maltoporin-like | Membrane and cell surface proteins and peptides |  |
| HP1056 | f.4.1.1 | TRANSMEMBRANE BETA BAREELS | OMPA LIKE | OUTERMEMBRANE PROTEINS | Membrane and cell surface proteins and peptides |  |
| HP1057 | f.4.3.2 | Transmembrane beta-barrels | Porins | Maltoporin-like | Membrane and cell surface proteins and peptides |  |
| HP1058 | c.1.12.8 | TIM beta/alpha-barrel | Phosphoenolpyruvate/pyruvate domain | Ketopantoate hydroxymethyltransferase PanB | Alpha and beta proteins (a/b) | [GO:0003864 "3-methyl-2-oxobutanoate  hydroxymethyltransferase activity" evidence=ISS] [GO:0015940  "pantothenate biosynthetic process" evidence=ISS] |
| HP1059 | c.37.1.20 | P-loop containing nucleoside triphosphate hydrolases | P-loop containing nucleoside triphosphate hydrolases | Extended AAA-ATPase domain | Alpha and beta proteins (a/b) | [GO:0006310 "DNA  recombination" evidence=ISS] [GO:0009378 "four-way junction  helicase activity" evidence=ISS] |
| HP1060 | a.26.1.1 | 4-helical cytokines | 4-helical cytokines | Long-chain cytokines | All alpha proteins | [GO:0015031 "protein  transport" evidence=ISS] |
| HP1061 | a.238.1.1 | BAR/IMD domain-like | BAR/IMD domain-like | BAR domain | All alpha proteins | [GO:0006886  "intracellular protein transport" evidence=ISS] [GO:0008565  "protein transporter activity" evidence=ISS] |
| HP1062 | e.53.1.1 | QueA-like | QueA-like | QueA-like | Multi-domain proteins (alpha and beta) | [GO:0006400 "tRNA modification" evidence=ISS] [GO:0008616  "queuosine biosynthetic process" evidence=ISS] [GO:0016853  "isomerase activity" evidence=ISS] |
| HP1063 | c.66.1.20 | S-adenosyl-L-methionine-dependent methyltransferases | S-adenosyl-L-methionine-dependent methyltransferases | Glucose-inhibited division protein B | Alpha and beta proteins (a/b) | [GO:0008152 "metabolic process"  evidence=ISS] [GO:0008168 "methyltransferase activity"  evidence=ISS] |
| HP1064 | g.46.1.1 | Metallothionein | Metallothionein | Metallothionein | Small proteins |  |
| HP1065 | c.1.10.1 | TIM beta/alpha-barrel | Aldolase | Class I aldolase | Alpha and beta proteins (a/b) | [GO:0003674  "molecular_function" evidence=ND] [GO:0005575 "cellular_component"  evidence=ND] [GO:0008150 "biological_process" evidence=ND] |
| HP1066 | f.4.3.2 | Transmembrane beta-barrels | Porins | Maltoporin-like | Membrane and cell surface proteins and peptides |  |
| HP1067 | c.23.1.1 | Flavodoxin-like | CheY-like | CheY-related | Alpha and beta proteins (a/b) | [GO:0005515 "protein binding"  evidence=IPI] |
| HP1068 | c.66.1.39 | S-adenosyl-L-methionine-dependent methyltransferases | S-adenosyl-L-methionine-dependent methyltransferases | Ribosomal protein | Alpha and beta proteins (a/b) | [GO:0006464  "cellular protein modification process" evidence=ISS] [GO:0008757  "S-adenosylmethionine-dependent methyltransferase activity"  evidence=ISS] |
| HP1069 |  |  |  |  |  | [GO:0000910  "cytokinesis" evidence=ISS] [GO:0004222 "metalloendopeptidase  activity" evidence=ISS] [GO:0016887 "ATPase activity" evidence=ISS] |
|  |  |  |  |  |  | [GO:0000910  "cytokinesis" evidence=ISS] [GO:0004222 "metalloendopeptidase  activity" evidence=ISS] [GO:0016887 "ATPase activity" evidence=ISS] |
| HP1070 | b.1.2.1; | Immunoglobulin-like beta-sandwich | Fibronectin type III | Fibronectin type III | All beta proteins |  |
| HP1071 | d.2.1.3; | Lysozyme-like | Lysozyme-like | Phage lysozyme | Alpha and beta proteins (a+b) | [GO:0003882 "CDP-diacylglycerol-serine  O-phosphatidyltransferase activity" evidence=ISS] [GO:0008654  "phospholipid biosynthetic process" evidence=ISS] |
| HP1072 | d.220.1.1 | Metal cation-transporting ATPase, ATP-binding domain N | Metal cation-transporting ATPase, ATP-binding domain N | Metal cation-transporting ATPase, ATP-binding domain N | Alpha and beta proteins (a+b) | [GO:0006812 "cation  transport" evidence=ISS] [GO:0015662 "ATPase activity, coupled to  transmembrane movement of ions, phosphorylative mechanism"  evidence=ISS] |
| HP1073 | d.58.17.1 | Ferredoxin-like | HMA, heavy metal-associated domain | HMA, heavy metal-associated domain | Alpha and beta proteins (a+b) | [GO:0003824 "catalytic activity" evidence=ISS] [GO:0008152  "metabolic process" evidence=ISS] |
| HP1074 | b.36.1.1 | PDZ domain-like | PDZ domain-like | PDZ domain | All beta proteins |  |
| HP1075 | c.56.5.1 | Phosphorylase/hydrolase-like | Zn-dependent exopeptidases | Pancreatic carboxypeptidases | Alpha and beta proteins (a/b) | [GO:0003674  "molecular_function" evidence=ND] [GO:0005575 "cellular_component"  evidence=ND] [GO:0008150 "biological_process" evidence=ND] |
|  | c.56.5.7 | Phosphorylase/hydrolase-like | Zn-dependent exopeptidases | AstE/AspA-like | Alpha and beta proteins (a/b) | [GO:0003674  "molecular_function" evidence=ND] [GO:0005575 "cellular_component"  evidence=ND] [GO:0008150 "biological_process" evidence=ND] |
| HP1076 | e.32.1.1 | Phase 1 flagellin | Phase 1 flagellin | Phase 1 flagellin | Multi-domain proteins (alpha and beta) | [GO:0003674  "molecular_function" evidence=ND] [GO:0005575 "cellular_component"  evidence=ND] [GO:0008150 "biological_process" evidence=ND] |
| HP1077 | g.40.1.1 | Retrovirus zinc finger-like domains | Retrovirus zinc finger-like domains | Retrovirus zinc finger-like domains | Small proteins | [GO:0005886  "plasma membrane" evidence=IDA] [GO:0040007 "growth" evidence=IMP] |
| HP1078 | e.67.1.1 | PH0156-like | PH0156-like | PH0156-like | Multi-domain proteins (alpha and beta) |  |
| HP1079 | c.37.1.12 | P-loop containing nucleoside triphosphate hydrolases | P-loop containing nucleoside triphosphate hydrolases | ABC transporter ATPase domain-like | Alpha and beta proteins (a/b) | [GO:0000070  "mitotic sister chromatid segregation" evidence=ISS] |
| HP1080 | c.52.1.30 | Restriction endonuclease-like | Restriction endonuclease-like | MRR-like | Alpha and beta proteins (a/b) | [GO:0009307 "DNA restriction-modification system"  evidence=ISS] [GO:0015666 "restriction endodeoxyribonuclease  activity" evidence=ISS] |
| HP1081 | c.51.6.1 | Anticodon-binding domain-like | XCC0632-like | NLBH-like | Alpha and beta proteins (a/b) |  |
| HP1082 |  |  |  |  |  | [GO:0005319 "lipid transporter activity"  evidence=ISS] [GO:0006869 "lipid transport" evidence=ISS]  [GO:0042626 "ATPase activity, coupled to transmembrane movement of  substances" evidence=ISS] |
| HP1083 | f.4.3.3 | Transmembrane beta-barrels | Porins | Ligand-gated protein channel | Membrane and cell surface proteins and peptides | [GO:0045203 "integral to cell outer membrane" evidence=IDA]  [GO:0015288 "porin activity" evidence=IMP] [GO:0015478  "oligosaccharide transporting porin activity" evidence=IMP] |
| HP1084 | c.78.1.1 | ATC-like | Aspartate/ornithine carbamoyltransferase | Aspartate/ornithine carbamoyltransferase | Alpha and beta proteins (a/b) | [GO:0004070 "aspartate  carbamoyltransferase activity" evidence=ISS] [GO:0009220  "pyrimidine ribonucleotide biosynthetic process" evidence=ISS] |
| HP1085 | b.61.6.1 | Streptavidin-like | YceI-like | YceI-like | All beta proteins | [GO:0004672 "protein kinase activity"  evidence=IEA] [GO:0004674 "protein serine/threonine kinase  activity" evidence=IEA] [GO:0005524 "ATP binding" evidence=IEA]  [GO:0005618 "cell wall" evidence=ISS] [GO:0006468 "protein  phosphorylation" evidence=IEA;ISS] |
| HP1086 | c.66.1.3 | S-adenosyl-L-methionine-dependent methyltransferases | S-adenosyl-L-methionine-dependent methyltransferases | Fibrillarin homologue | Alpha and beta proteins (a/b) | [GO:0009405  "pathogenesis" evidence=ISS] [GO:0019836 "hemolysis by symbiont of  host erythrocytes" evidence=ISS] |
| HP1087 | c.26.1.3 | Adenine nucleotide alpha hydrolase-like | Nucleotidylyl transferase | Adenylyltransferase | Alpha and beta proteins (a/b) | [GO:0003919  "FMN adenylyltransferase activity" evidence=ISS] [GO:0006747 "FAD  biosynthetic process" evidence=ISS] [GO:0008531 "riboflavin kinase  activity" evidence=ISS] [GO:0009231 "riboflavin biosynthetic  process" evidence=ISS] |
| HP1088 | c.36.1.10 | Thiamin diphosphate-binding fold (THDP-binding) | Thiamin diphosphate-binding fold (THDP-binding) | TK-like PP module | Alpha and beta proteins (a/b) | [GO:0004802 "transketolase activity"  evidence=ISS] [GO:0006098 "pentose-phosphate shunt" evidence=ISS] |
| HP1089 | c.37.1.19 | P-loop containing nucleoside triphosphate hydrolases | P-loop containing nucleoside triphosphate hydrolases | Tandem AAA-ATPase domain | Alpha and beta proteins (a/b) | [GO:0003674  "molecular_function" evidence=ND] [GO:0005575 "cellular_component"  evidence=ND] [GO:0008150 "biological_process" evidence=ND] |
| HP1090 | c.37.1.19 | P-loop containing nucleoside triphosphate hydrolases | P-loop containing nucleoside triphosphate hydrolases | Tandem AAA-ATPase domain | Alpha and beta proteins (a/b) | [GO:0000910  "cytokinesis" evidence=ISS] [GO:0005199 "structural constituent of  cell wall" evidence=ISS] |
|  | c.37.1.19 | P-loop containing nucleoside triphosphate hydrolases | P-loop containing nucleoside triphosphate hydrolases | Tandem AAA-ATPase domain | Alpha and beta proteins (a/b) | [GO:0000910  "cytokinesis" evidence=ISS] [GO:0005199 "structural constituent of  cell wall" evidence=ISS] |
| HP1091 | f.54.1.1 | SNF-like | SNF-like | SNF-like | Membrane and cell surface proteins and peptides | [GO:0015532  "alpha-ketoglutarate:hydrogen symporter activity" evidence=ISS]  [GO:0015742 "alpha-ketoglutarate transport" evidence=ISS] |
| HP1092 | b.152.1.1 | Flagellar hook protein flgE | Flagellar hook protein flgE | Flagellar hook protein flgE | All beta proteins | [GO:0001539 "ciliary  or flagellar motility" evidence=ISS] [GO:0005198 "structural  molecule activity" evidence=ISS] [GO:0009425 "bacterial-type  flagellum basal body" evidence=ISS] |
| HP1093 |  |  |  |  |  |  |
| HP1094 | g.7.1.3 | Snake toxin-like | Snake toxin-like | Extracellular domain of cell surface receptors | Small proteins |  |
| HP1095 | b.113.1.1 | N-terminal domain of MutM-like DNA repair proteins | N-terminal domain of MutM-like DNA repair proteins | N-terminal domain of MutM-like DNA repair proteins | All beta proteins | [GO:0004803 "transposase activity"  evidence=ISS] [GO:0006313 "transposition, DNA-mediated"  evidence=ISS] |
| HP1096 | d.58.57.1 | Ferredoxin-like | Transposase IS200-like | Transposase IS200-like | Alpha and beta proteins (a+b) | [GO:0004803 "transposase  activity" evidence=ISS] [GO:0006313 "transposition, DNA-mediated"  evidence=ISS] |
| HP1097 | d.241.1.1 | Ribosome binding domain-like | Translation initiation factor 2 beta, aIF2beta, N-terminal domain | Translation initiation factor 2 beta, aIF2beta, N-terminal domain | Alpha and beta proteins (a+b) |  |
| HP1098 | a.118.18.1 | alpha-alpha superhelix | HCP-like | HCP-like | All alpha proteins | [GO:0003674 "molecular_function"  evidence=ND] [GO:0008150 "biological_process" evidence=ND] |
| HP1099 | c.1.10.1 | TIM beta/alpha-barrel | Aldolase | Class I aldolase | Alpha and beta proteins (a/b) | [GO:0008675  "2-dehydro-3-deoxy-phosphogluconate aldolase activity"  evidence=ISS] [GO:0008700 "4-hydroxy-2-oxoglutarate aldolase  activity" evidence=ISS] |
| HP1100 | d.334.1.1 | IlvD/EDD N-terminal domain-like | IlvD/EDD N-terminal domain-like | IlvD/EDD N-terminal domain-like | Alpha and beta proteins (a+b) | [GO:0004456  "phosphogluconate dehydratase activity" evidence=ISS] [GO:0009255  "Entner-Doudoroff pathway" evidence=ISS] |
| HP1101 | d.81.1.5 | FwdE/GAPDH domain-like | Glyceraldehyde-3-phosphate dehydrogenase-like, C-terminal domain | Glucose 6-phosphate dehydrogenase-like | Alpha and beta proteins (a+b) | [GO:0004345  "glucose-6-phosphate dehydrogenase activity" evidence=ISS]  [GO:0006098 "pentose-phosphate shunt" evidence=ISS] |
| HP1102 | c.124.1.1 | NagB/RpiA/CoA transferase-like | NagB/RpiA/CoA transferase-like | NagB-like | Alpha and beta proteins (a/b) | [GO:0006098 "pentose-phosphate shunt"  evidence=ISS] [GO:0017057 "6-phosphogluconolactonase activity"  evidence=ISS] |
| HP1103 | c.55.1.7 | Ribonuclease H-like motif | Actin-like ATPase domain | Glucokinase | Alpha and beta proteins (a/b) | [GO:0004340 "glucokinase activity"  evidence=ISS] [GO:0005975 "carbohydrate metabolic process"  evidence=ISS] |
| HP1104 | b.35.1.2 | GroES-like | GroES-like | Alcohol dehydrogenase-like, N-terminal domain | All beta proteins | [GO:0008152 "metabolic process" evidence=ISS] [GO:0008270 "zinc ion  binding" evidence=ISS] [GO:0016491 "oxidoreductase activity"  evidence=ISS] |
| HP1105 | c.68.1.4 | Nucleotide-diphospho-sugar transferases | Nucleotide-diphospho-sugar transferases | Galactosyltransferase LgtC | Alpha and beta proteins (a/b) | [GO:0009244 "lipopolysaccharide core region biosynthetic  process" evidence=IEA] [GO:0008918 "lipopolysaccharide  3-alpha-galactosyltransferase activity" evidence=IEA;IMP]  [GO:0009103 "lipopolysaccharide biosynthetic process" evidence=IEA] |
| HP1106 | c.47.1.1 | Thioredoxin fold | Thioredoxin-like | Thioltransferase | Alpha and beta proteins (a/b) |  |
| HP1107 | f.4.3.2 | Transmembrane beta-barrels | Porins | Maltoporin-like | Membrane and cell surface proteins and peptides |  |
| HP1108 | c.64.1.1 | Pyruvate-ferredoxin oxidoreductase, PFOR, domain III | Pyruvate-ferredoxin oxidoreductase, PFOR, domain III | Pyruvate-ferredoxin oxidoreductase, PFOR, domain III | Alpha and beta proteins (a/b) | [GO:0006086 "acetyl-CoA biosynthetic process from pyruvate"  evidence=ISS] [GO:0019164 "pyruvate synthase activity"  evidence=ISS] |
| HP1109 | d.58.1.5 | Ferredoxin-like | 4Fe-4S ferredoxins | Ferredoxin domains from multidomain proteins | Alpha and beta proteins (a+b) | [GO:0006086 "acetyl-CoA biosynthetic process from pyruvate"  evidence=ISS] [GO:0009055 "electron carrier activity" evidence=ISS]  [GO:0019164 "pyruvate synthase activity" evidence=ISS] |
|  | d.58.1.5 | Ferredoxin-like | 4Fe-4S ferredoxins | Ferredoxin domains from multidomain proteins | Alpha and beta proteins (a+b) | [GO:0006086 "acetyl-CoA biosynthetic process from pyruvate"  evidence=ISS] [GO:0009055 "electron carrier activity" evidence=ISS]  [GO:0019164 "pyruvate synthase activity" evidence=ISS] |
| HP1110 | c.36.1.12 | Thiamin diphosphate-binding fold (THDP-binding) | Thiamin diphosphate-binding fold (THDP-binding) | PFOR PP module | Alpha and beta proteins (a/b) | [GO:0006086 "acetyl-CoA biosynthetic process from pyruvate"  evidence=ISS] [GO:0019164 "pyruvate synthase activity"  evidence=ISS] |
| HP1111 | c.36.1.12 | Thiamin diphosphate-binding fold (THDP-binding) | Thiamin diphosphate-binding fold (THDP-binding) | PFOR PP module | Alpha and beta proteins (a/b) | [GO:0006086 "acetyl-CoA biosynthetic process from pyruvate"  evidence=ISS] [GO:0019164 "pyruvate synthase activity"  evidence=ISS] |
| HP1112 | a.127.1.1 | L-aspartase-like | L-aspartase-like | L-aspartase/fumarase | All alpha proteins | [GO:0009152 "purine ribonucleotide biosynthetic  process" evidence=ISS] |
| HP1113 | f.4.3.2 | Transmembrane beta-barrels | Porins | Maltoporin-like | Membrane and cell surface proteins and peptides |  |
| HP1114 | c.37.1.19 | P-loop containing nucleoside triphosphate hydrolases | P-loop containing nucleoside triphosphate hydrolases | Tandem AAA-ATPase domain | Alpha and beta proteins (a/b) | [GO:0006281 "DNA  repair" evidence=ISS] [GO:0009380 "excinuclease repair complex"  evidence=ISS] [GO:0009381 "excinuclease ABC activity" evidence=ISS] |
| HP1115 |  |  |  |  |  | [GO:0005829  "cytosol" evidence=IEA] [GO:0051177 "meiotic sister chromatid  cohesion" evidence=IEA] [GO:0007130 "synaptonemal complex assembly"  evidence=IEA] |
| HP1116 | f.42.1.1 | Mitochondrial carrier | Mitochondrial carrier | Mitochondrial carrier | Membrane and cell surface proteins and peptides | [GO:0003674  "molecular_function" evidence=ND] [GO:0005575 "cellular_component"  evidence=ND] [GO:0008150 "biological_process" evidence=ND] |
| HP1117 | a.118.18.1 | alpha-alpha superhelix | HCP-like | HCP-like | All alpha proteins | [GO:0003674 "molecular_function"  evidence=ND] [GO:0008150 "biological_process" evidence=ND] |
| HP1118 | d.153.1.6 | Ntn hydrolase-like | N-terminal nucleophile aminohydrolases (Ntn hydrolases) | Gamma-glutamyltranspeptidase-like | Alpha and beta proteins (a+b) | [GO:0003840  "gamma-glutamyltransferase activity" evidence=IEA;IDA] [GO:0016787  "hydrolase activity" evidence=IEA] [GO:0006749 "glutathione  metabolic process" evidence=IEA] |
| HP1119 | b.152.1.1 | Flagellar hook protein flgE | Flagellar hook protein flgE | Flagellar hook protein flgE | All beta proteins | [GO:0001539  "ciliary or flagellar motility" evidence=ISS] [GO:0005198  "structural molecule activity" evidence=ISS] [GO:0009424  "bacterial-type flagellum hook" evidence=ISS] |
|  | a.238.1.4 | BAR/IMD domain-like | BAR/IMD domain-like | FCH domain | All alpha proteins | [GO:0001539  "ciliary or flagellar motility" evidence=ISS] [GO:0005198  "structural molecule activity" evidence=ISS] [GO:0009424  "bacterial-type flagellum hook" evidence=ISS] |
| HP1120 | a.47.5.1 | STAT-like | FlgN-like | FlgN-like | All alpha proteins | [GO:0005515 "protein binding" evidence=IPI] |
| HP1121 | c.66.1.26 | S-adenosyl-L-methionine-dependent methyltransferases | S-adenosyl-L-methionine-dependent methyltransferases | C5 cytosine-specific DNA methylase, DCM | Alpha and beta proteins (a/b) | [GO:0003677 "DNA binding" evidence=IEA]  [GO:0090116 "C-5 methylation of cytosine" evidence=IEA;IDA]  [GO:0005515 "protein binding" evidence=IPI] |
| HP1122 | a.137.11.1 | Non-globular all-alpha subunits of globular proteins | Anti-sigma factor FlgM | Anti-sigma factor FlgM | All alpha proteins | [GO:0005515 "protein  binding" evidence=IPI] |
| HP1123 | d.26.1.1 | FKBP-like | FKBP-like | FKBP immunophilin/proline isomerase | Alpha and beta proteins (a+b) | [GO:0003755 "peptidyl-prolyl cis-trans isomerase activity"  evidence=ISS] [GO:0006457 "protein folding" evidence=ISS] |
|  | d.26.1.1 | FKBP-like | FKBP-like | FKBP immunophilin/proline isomerase | Alpha and beta proteins (a+b) | [GO:0003755 "peptidyl-prolyl cis-trans isomerase activity"  evidence=ISS] [GO:0006457 "protein folding" evidence=ISS] |
| HP1124 | a.118.8.1 | alpha-alpha superhelix | TPR-like | Tetratricopeptide repeat | All alpha proteins | [GO:0003674  "molecular_function" evidence=ND] [GO:0005575 "cellular_component"  evidence=ND] [GO:0008150 "biological_process" evidence=ND] |
|  | a.118.8.1 | alpha-alpha superhelix | TPR-like | Tetratricopeptide repeat | All alpha proteins | [GO:0003674  "molecular_function" evidence=ND] [GO:0005575 "cellular_component"  evidence=ND] [GO:0008150 "biological_process" evidence=ND] |
|  | a.118.8.1 | alpha-alpha superhelix | TPR-like | Tetratricopeptide repeat | All alpha proteins | [GO:0003674  "molecular_function" evidence=ND] [GO:0005575 "cellular_component"  evidence=ND] [GO:0008150 "biological_process" evidence=ND] |
| HP1125 | d.79.7.1 | Bacillus chorismate mutase-like | OmpA-like | OmpA-like | Alpha and beta proteins (a+b) | [GO:0003674  "molecular_function" evidence=ND] [GO:0008150 "biological_process"  evidence=ND] |
| HP1126 | b.68.4.1 | bladed beta-propeller | TolB, C-terminal domain | TolB, C-terminal domain | All beta proteins | [GO:0005215 "transporter activity"  evidence=ISS] [GO:0006810 "transport" evidence=ISS] |
| HP1127 | d.212.1.1 | TolA/TonB C-terminal domain | TolA/TonB C-terminal domain | TolA | Alpha and beta proteins (a+b) |  |
| HP1128 | f.16.1.1 | Gated mechanosensitive channel | Gated mechanosensitive channel | Gated mechanosensitive channel | Membrane and cell surface proteins and peptides |  |
| HP1129 | c.81.1.1 | Formate dehydrogenase/DMSO reductase, domains 1-3 | Formate dehydrogenase/DMSO reductase, domains 1-3 | Formate dehydrogenase/DMSO reductase, domains 1-3 | Alpha and beta proteins (a/b) | [GO:0005215  "transporter activity" evidence=ISS] [GO:0006810 "transport"  evidence=ISS] |
| HP1130 | f.51.1.1 | Rhomboid-like | Rhomboid-like | Rhomboid-like | Membrane and cell surface proteins and peptides | [GO:0015078  "hydrogen ion transmembrane transporter activity" evidence=ISS]  [GO:0015992 "proton transport" evidence=ISS] [GO:0016020 "membrane"  evidence=ISS] |
| HP1131 | b.93.1.1 | Epsilon subunit of F1F0-ATP synthase N-terminal domain | Epsilon subunit of F1F0-ATP synthase N-terminal domain | Epsilon subunit of F1F0-ATP synthase N-terminal domain | All beta proteins | [GO:0015986 "ATP  synthesis coupled proton transport" evidence=ISS] [GO:0045261  "proton-transporting ATP synthase complex, catalytic core F(1)"  evidence=ISS] |
| HP1132 | c.37.1.11 | P-loop containing nucleoside triphosphate hydrolases | P-loop containing nucleoside triphosphate hydrolases | RecA protein-like | Alpha and beta proteins (a/b) | [GO:0015986 "ATP  synthesis coupled proton transport" evidence=ISS] [GO:0045261  "proton-transporting ATP synthase complex, catalytic core F(1)"  evidence=ISS] [GO:0046933 "proton-transporting ATP synthase  activity, rotational mechanism" evidence=ISS] |
| HP1133 | c.49.2.1 | Pyruvate kinase C-terminal domain-like | ATP synthase (F1-ATPase), gamma subunit | ATP synthase (F1-ATPase), gamma subunit | Alpha and beta proteins (a/b) | GO:0015986 "ATP  synthesis coupled proton transport" evidence=ISS] [GO:0045261  "proton-transporting ATP synthase complex, catalytic core F(1)"  evidence=ISS] |
| HP1134 | c.37.1.11 | P-loop containing nucleoside triphosphate hydrolases | P-loop containing nucleoside triphosphate hydrolases | RecA protein-like | Alpha and beta proteins (a/b) | [GO:0015986 "ATP  synthesis coupled proton transport" evidence=ISS] [GO:0045261  "proton-transporting ATP synthase complex, catalytic core F(1)"  evidence=ISS] |
| HP1135 | f.38.1.1 | MFS general substrate transporter | MFS general substrate transporter | Glycerol-3-phosphate transporter | Membrane and cell surface proteins and peptides | [GO:0015986 "ATP  synthesis coupled proton transport" evidence=ISS] [GO:0045261  "proton-transporting ATP synthase complex, catalytic core F(1)"  evidence=ISS] |
| HP1136 | f.52.1.1 | ATP synthase B chain-like | ATP synthase B chain-like | ATP synthase B chain-like | Membrane and cell surface proteins and peptides | [GO:0015986 "ATP  synthesis coupled proton transport" evidence=ISS] [GO:0045264  "plasma membrane proton-transporting ATP synthase complex, coupling  factor F(o)" evidence=ISS] |
| HP1137 | f.52.1.1 | ATP synthase B chain-like | ATP synthase B chain-like | ATP synthase B chain-like | Membrane and cell surface proteins and peptides | [GO:0015986 "ATP  synthesis coupled proton transport" evidence=ISS] [GO:0045264  "plasma membrane proton-transporting ATP synthase complex, coupling  factor F(o)" evidence=ISS] |
| HP1138 | d.268.1.1 | ParB/Sulfiredoxin | ParB/Sulfiredoxin | ParB-like nuclease domain | Alpha and beta proteins (a+b) | [GO:0000910  "cytokinesis" evidence=ISS] [GO:0003677 "DNA binding" evidence=ISS] |
|  | a.4.14.1 | DNA/RNA-binding 3-helical bundle | KorB DNA-binding domain-like | KorB DNA-binding domain-like | All alpha proteins | [GO:0000910  "cytokinesis" evidence=ISS] [GO:0003677 "DNA binding" evidence=ISS] |
| HP1139 | c.37.1.10 | P-loop containing nucleoside triphosphate hydrolases | P-loop containing nucleoside triphosphate hydrolases | Nitrogenase iron protein-like | Alpha and beta proteins (a/b) | [GO:0000910  "cytokinesis" evidence=ISS] [GO:0016887 "ATPase activity"  evidence=ISS] |
| HP1140 | d.104.1.2 | Class II aaRS and biotin synthetases | Class II aaRS and biotin synthetases | Biotin holoenzyme synthetase | Alpha and beta proteins (a+b) | [GO:0004077  "biotin-[acetyl-CoA-carboxylase] ligase activity" evidence=ISS]  [GO:0009102 "biotin biosynthetic process" evidence=ISS] |
| HP1141 | c.65.1.1 | Formyltransferase | Formyltransferase | Formyltransferase | Alpha and beta proteins (a/b) | [GO:0004479  "methionyl-tRNA formyltransferase activity" evidence=ISS]  [GO:0006431 "methionyl-tRNA aminoacylation" evidence=ISS] |
| HP1142 | c.37.1.12 | P-loop containing nucleoside triphosphate hydrolases | P-loop containing nucleoside triphosphate hydrolases | ABC transporter ATPase domain-like | Alpha and beta proteins (a/b) | [GO:0003674 "molecular_function" evidence=ND] [GO:0005575  "cellular_component" evidence=ND] [GO:0008150 "biological_process"  evidence=ND] |
|  | a.118.1.1 | alpha-alpha superhelix | ARM repeat | Armadillo repeat | All alpha proteins | [GO:0003674 "molecular_function" evidence=ND] [GO:0005575  "cellular_component" evidence=ND] [GO:0008150 "biological_process"  evidence=ND] |
| HP1143 | b.2.5.5 | Common fold of diphtheria toxin/transcription factors/cytochrome f | p53-like transcription factors | STAT DNA-binding domain | All beta proteins | [GO:0003674  "molecular_function" evidence=ND] [GO:0005575 "cellular_component"  evidence=ND] [GO:0008150 "biological_process" evidence=ND] |
|  | b.110.1.1 | Cloacin translocation domain | Cloacin translocation domain | Cloacin translocation domain | All beta proteins | [GO:0003674  "molecular_function" evidence=ND] [GO:0005575 "cellular_component"  evidence=ND] [GO:0008150 "biological_process" evidence=ND] |
| HP1144 | b.40.4.3 | OB-fold | Nucleic acid-binding proteins | Single strand DNA-binding domain, SSB | All beta proteins |  |
| HP1145 | c.1.12.7 | TIM beta/alpha-barrel | Phosphoenolpyruvate/pyruvate domain | Phosphoenolpyruvate mutase/Isocitrate lyase-like | Alpha and beta proteins (a/b) |  |
| HP1146 | b.123.1.1 | Hypothetical protein TM1070 | Hypothetical protein TM1070 | Hypothetical protein TM1070 | All beta proteins |  |
| HP1147 | d.166.1.1 | ADP-ribosylation | ADP-ribosylation | ADP-ribosylating toxins | Alpha and beta proteins (a+b) | [GO:0003735 "structural constituent  of ribosome" evidence=ISS] [GO:0006412 "translation" evidence=ISS]  [GO:0042254 "ribosome biogenesis" evidence=ISS] |
| HP1148 | c.116.1.4 | alpha/beta knot | alpha/beta knot | tRNA(m1G37)-methyltransferase TrmD | Alpha and beta proteins (a/b) | [GO:0006400 "tRNA  modification" evidence=ISS] [GO:0009019 "tRNA  (guanine-N1-)-methyltransferase activity" evidence=ISS] |
| HP1149 | b.43.3.4 | Reductase/isomerase/elongation factor common domain | Translation proteins | RimM N-terminal domain-like | All beta proteins | [GO:0003674  "molecular_function" evidence=ND] |
| HP1150 | c.79.1.1 | Tryptophan synthase beta subunit-like PLP-dependent enzymes | Tryptophan synthase beta subunit-like PLP-dependent enzymes | Tryptophan synthase beta subunit-like PLP-dependent enzymes | Alpha and beta proteins (a/b) | [GO:0003674  "molecular_function" evidence=ND] [GO:0005575 "cellular_component"  evidence=ND] [GO:0008150 "biological_process" evidence=ND] |
| HP1151 |  | Ribosome and ribosomal fragments | Ribosome and ribosomal fragments | Ribosome complexes | Low resolution protein structures | [GO:0003735 "structural constituent  of ribosome" evidence=ISS] [GO:0004519 "endonuclease activity"  evidence=ISS] [GO:0042254 "ribosome biogenesis" evidence=IMP] |
| HP1152 |  | Four-helical up-and-down bundle | Domain of the SRP/SRP receptor G-proteins | Domain of the SRP/SRP receptor G-proteins | All alpha proteins | [GO:0003924 "GTPase  activity" evidence=ISS] [GO:0005048 "signal sequence binding"  evidence=ISS] [GO:0009306 "protein secretion" evidence=ISS] |
| HP1153 | c.26.1.1 | Adenine nucleotide alpha hydrolase-like | Nucleotidylyl transferase | Class I aminoacyl-tRNA synthetases (RS), catalytic domain | Alpha and beta proteins (a/b) | [GO:0004832 "valine-tRNA ligase  activity" evidence=ISS] [GO:0006438 "valyl-tRNA aminoacylation"  evidence=ISS] |
| HP1154 | b.158.1.1 | BH3618-like | BH3618-like | BH3618-like | All beta proteins | [GO:0003674 "molecular_function"  evidence=ND] [GO:0005575 "cellular_component" evidence=ND]  [GO:0008150 "biological_process" evidence=ND] |
| HP1155 | c.87.1.2 | UDP-Glycosyltransferase/glycogen phosphorylase | UDP-Glycosyltransferase/glycogen phosphorylase | Peptidoglycan biosynthesis glycosyltransferase MurG | Alpha and beta proteins (a/b) | [GO:0009252  "peptidoglycan biosynthetic process" evidence=ISS] [GO:0016758  "transferase activity, transferring hexosyl groups" evidence=ISS] |
| HP1156 | f.4.1.1 | TRANSMEMBRANE BETA BAREELS | OMPA LIKE | OUTERMEMBRANE PROTEINS | Membrane and cell surface proteins and peptides |  |
| HP1157 | g.7.1.3 | Snake toxin-like | Snake toxin-like | Extracellular domain of cell surface receptors | Small proteins |  |
| HP1157 | g.7.1.3 | Snake toxin-like | Snake toxin-like | Extracellular domain of cell surface receptors | Small proteins |  |
|  | g.7.1.3 | Snake toxin-like | Snake toxin-like | Extracellular domain of cell surface receptors | Small proteins |  |
| HP1157 | g.7.1.3 | Snake toxin-like | Snake toxin-like | Extracellular domain of cell surface receptors | Small proteins |  |
| HP1157 | f.4.1.1 | TRANSMEMBRANE BETA BAREELS | OMPA LIKE | OUTERMEMBRANE PROTEINS | Membrane and cell surface proteins and peptides |  |
| HP1158 | c.2.1.6 | NAD(P)-binding Rossmann-fold domains | NAD(P)-binding Rossmann-fold domains | 6-phosphogluconate dehydrogenase-like, N-terminal domain | Alpha and beta proteins (a/b) | [GO:0004735  "pyrroline-5-carboxylate reductase activity" evidence=ISS]  [GO:0006561 "proline biosynthetic process" evidence=ISS] |
| HP1159 | a.265.1.1 | Fic-like | Fic-like | Fic-like | All alpha proteins | [GO:0018117  "protein adenylylation" evidence=IDA] [GO:0070733 "protein  adenylyltransferase activity" evidence=IDA] |
| HP1160 | d.92.1.15 | Zincin-like | Metalloproteases ("zincins"), catalytic domain | Predicted metal-dependent hydrolase | Alpha and beta proteins (a+b) | [GO:0003674 "molecular_function"  evidence=ND] [GO:0005575 "cellular_component" evidence=ND]  [GO:0008150 "biological_process" evidence=ND] |
| HP1161 | c.23.5.1 | Flavodoxin-like | Flavoproteins | Flavodoxin-related | Alpha and beta proteins (a/b) | [GO:0009055 "electron carrier  activity" evidence=ISS] |
| HP1162 | d.2.1.3 | Lysozyme-like | Lysozyme-like | Phage lysozyme | Alpha and beta proteins (a+b) | [GO:0003674 "molecular_function"  evidence=ND] [GO:0008150 "biological_process" evidence=ND] |
| HP1163 | f.24.1.1 | Cytochrome c oxidase subunit I-like | Cytochrome c oxidase subunit I-like | Cytochrome c oxidase subunit I-like | Membrane and cell surface proteins and peptides | [GO:0003674 "molecular_function" evidence=ND] |
| HP1164 | c.3.1.5 | FAD/NAD(P)-binding domain | FAD/NAD(P)-binding domain | FAD/NAD-linked reductases, N-terminal and central domains | Alpha and beta proteins (a/b) | [GO:0008152 "metabolic process" evidence=ISS] [GO:0015036  "disulfide oxidoreductase activity" evidence=ISS] |
| HP1165 | f.38.1.2 | MFS general substrate transporter | MFS general substrate transporter | LacY-like proton/sugar symporter | Membrane and cell surface proteins and peptides | [GO:0003674 "molecular_function"  evidence=ND] [GO:0008150 "biological_process" evidence=ND] |
| HP1166 | c.80.1.2 | SIS domain | SIS domain | Phosphoglucose isomerase, PGI | Alpha and beta proteins (a/b) | [GO:0006096 "glycolysis" evidence=IEA]  [GO:0006094 "gluconeogenesis" evidence=IEA] [GO:0004347  "glucose-6-phosphate isomerase activity" evidence=IEA] |
| HP1167 | f.4.3.3 | Transmembrane beta-barrels | Porins | Ligand-gated protein channel | Membrane and cell surface proteins and peptides |  |
| HP1168 | d.272.1.1 | Dystroglycan, domain 2 | Dystroglycan, domain 2 | Dystroglycan, domain 2 | Alpha and beta proteins (a+b) | [GO:0003674 "molecular_function"  evidence=ND] |
| HP1169 | f.58.1.1 | MetI-like | MetI-like | MetI-like | Membrane and cell surface proteins and peptides | [GO:0006865 "amino acid transport"  evidence=ISS] [GO:0015424 "amino acid-transporting ATPase activity"  evidence=ISS] |
| HP1170 | f.58.1.1 | MetI-like | MetI-like | MetI-like | Membrane and cell surface proteins and peptides | [GO:0006865 "amino acid transport"  evidence=ISS] [GO:0015424 "amino acid-transporting ATPase activity"  evidence=ISS] |
| HP1171 | c.37.1.12 | P-loop containing nucleoside triphosphate hydrolases | P-loop containing nucleoside triphosphate hydrolases | ABC transporter ATPase domain-like | Alpha and beta proteins (a/b) | [GO:0006865  "amino acid transport" evidence=ISS] [GO:0042626 "ATPase activity,  coupled to transmembrane movement of substances" evidence=ISS] |
| HP1172 | c.94.1.1 | Periplasmic binding protein-like II | Periplasmic binding protein-like II | Phosphate binding protein-like | Alpha and beta proteins (a/b) | [GO:0003674 "molecular_function"  evidence=ND] [GO:0009405 "pathogenesis" evidence=ISS] |
| HP1173 | b.50.1.1 | Acid proteases | Acid proteases | Retroviral protease (retropepsin) | All beta proteins |  |
| HP1174 | f.38.1.2 | MFS general substrate transporter | MFS general substrate transporter | LacY-like proton/sugar symporter | Membrane and cell surface proteins and peptides | [GO:0008645 "hexose  transport" evidence=ISS] [GO:0015149 "hexose transmembrane  transporter activity" evidence=ISS] |
| HP1175 | f.38.1.2 | MFS general substrate transporter | MFS general substrate transporter | LacY-like proton/sugar symporter | Membrane and cell surface proteins and peptides | [GO:0005337  "nucleoside transmembrane transporter activity" evidence=ISS]  [GO:0015858 "nucleoside transport" evidence=ISS] |
| HP1177 | f.4.1.1 | TRANSMEMBRANE BETA BAREELS | OMPA LIKE | OUTERMEMBRANE PROTEINS | Membrane and cell surface proteins and peptides | [GO:0005886  "plasma membrane" evidence=IEA] [GO:0005796 "Golgi lumen"  evidence=TAS] [GO:0016266 "O-glycan processing" evidence=TAS]  [GO:0043687 "post-translational protein modification" evidence=TAS] |
| HP1178 | c.56.2.1 | Phosphorylase/hydrolase-like | Phosphorylase/hydrolase-like | Phosphorylase/hydrolase-like | Alpha and beta proteins (a/b) | [GO:0042802 "identical protein binding" evidence=IPI] [GO:0016020  "membrane" evidence=IDA] [GO:0005829 "cytosol" evidence=IDA]  [GO:0019686 "purine nucleoside interconversion" evidence=IDA]  [GO:0006152 "purine nucleoside catabolic process" evidence=IMP] |
| HP1179 | c.76.1.5 | Alkaline phosphatase-like | Alkaline phosphatase-like | DeoB catalytic domain-like | Alpha and beta proteins (a/b) | [GO:0006139  "nucleobase-containing compound metabolic process" evidence=ISS]  [GO:0008973 "phosphopentomutase activity" evidence=ISS] |
| HP1180 | a.118.1.1 | alpha-alpha superhelix | ARM repeat | Armadillo repeat | All alpha proteins | [GO:0005337 "nucleoside  transmembrane transporter activity" evidence=ISS] [GO:0015858  "nucleoside transport" evidence=ISS] |
| HP1181 | f.38.1.2 | MFS general substrate transporter | MFS general substrate transporter | LacY-like proton/sugar symporter | Membrane and cell surface proteins and peptides | [GO:0006855  "drug transmembrane transport" evidence=ISS] [GO:0016020 "membrane"  evidence=ISS] [GO:0042493 "response to drug" evidence=ISS] |
| HP1182 | c.26.2.5 | Adenine nucleotide alpha hydrolase-like | Adenine nucleotide alpha hydrolase-like | PP-loop ATPase | Alpha and beta proteins (a/b) | [GO:0003674 "molecular_function"  evidence=ND] [GO:0008150 "biological_process" evidence=ND] |
| HP1183 | a.25.1.2 | Ferritin-like | Ferritin-like | Ribonucleotide reductase-like | All alpha proteins | [GO:0006814 "sodium  ion transport" evidence=ISS] [GO:0006818 "hydrogen transport"  evidence=ISS] [GO:0015385 "sodium:hydrogen antiporter activity"  evidence=ISS] |
| HP1184 | f.24.1.1 | Cytochrome c oxidase subunit I-like | Cytochrome c oxidase subunit I-like | Cytochrome c oxidase subunit I-like | Membrane and cell surface proteins and peptides | [GO:0006855 "drug  transmembrane transport" evidence=ISS] [GO:0042493 "response to  drug" evidence=ISS] |
| HP1185 | f.38.1.1 | MFS general substrate transporter | MFS general substrate transporter | Glycerol-3-phosphate transporter | Membrane and cell surface proteins and peptides | [GO:0055085 "transmembrane transport"  evidence=IEA] [GO:0005886 "plasma membrane" evidence=IEA]  [GO:0016021 "integral to membrane" evidence=IEA] [GO:0016020  "membrane" evidence=IEA] [GO:0008643 "carbohydrate transport"  evidence=IEA] [GO:0006810 "transport" evidence=IEA] |
| HP1186 | b.74.1.1 | Carbonic anhydrase | Carbonic anhydrase | Carbonic anhydrase | All beta proteins | [GO:0004089 "carbonate  dehydratase activity" evidence=ISS] [GO:0008152 "metabolic process"  evidence=ISS] |
| HP1187 | a.212.1.1 | KRAB domain (Kruppel-associated box) | KRAB domain (Kruppel-associated box) | KRAB domain (Kruppel-associated box) | All alpha proteins | [GO:0003674 "molecular_function"  evidence=ND] [GO:0005575 "cellular_component" evidence=ND]  [GO:0008150 "biological_process" evidence=ND] |
| HP1188 | b.68.8.1 | 6-bladed beta-propeller | Fucose-specific lectin | Fucose-specific lectin | All beta proteins | [GO:0003674 "molecular_function" evidence=ND]  [GO:0003924 "GTPase activity" evidence=IEA;ISO] [GO:0004871 "signal  transducer activity" evidence=IEA] [GO:0005575 "cellular_component"  evidence=ND] [GO:0005834 "heterotrimeric G-protein complex"  evidence=IEA;ISO] |
| HP1189 | d.81.1.1 | FwdE/GAPDH domain-like | Glyceraldehyde-3-phosphate dehydrogenase-like, C-terminal domain | GAPDH-like | Alpha and beta proteins (a+b) | [GO:0004073  "aspartate-semialdehyde dehydrogenase activity" evidence=ISS]  [GO:0009086 "methionine biosynthetic process" evidence=ISS]  [GO:0009088 "threonine biosynthetic process" evidence=ISS] |
| HP1190 | d.104.1.1 | Class II aaRS and biotin synthetases | Class II aaRS and biotin synthetases | Class II aminoacyl-tRNA synthetase (aaRS)-like, catalytic domain | Alpha and beta proteins (a+b) | [GO:0004821 "histidine-tRNA ligase  activity" evidence=ISS] [GO:0006427 "histidyl-tRNA aminoacylation"  evidence=ISS] |
| HP1191 | c.87.1.7 | UDP-Glycosyltransferase/glycogen phosphorylase | UDP-Glycosyltransferase/glycogen phosphorylase | ADP-heptose LPS heptosyltransferase II | Alpha and beta proteins (a/b) | [GO:0008920  "lipopolysaccharide heptosyltransferase activity" evidence=ISS]  [GO:0009244 "lipopolysaccharide core region biosynthetic process"  evidence=ISS] |
| HP1192 | a.118.1.1 | alpha-alpha superhelix | ARM repeat | Armadillo repeat | All alpha proteins | [GO:0035392  "maintenance of chromatin silencing at telomere" evidence=IMP]  [GO:0005739 "mitochondrion" evidence=IDA] [GO:0006611 "protein  export from nucleus" evidence=IGI;IMP;IPI] [GO:0000061 "protein  import into nucleus, substrate release" evidence=IDA] |
| HP1193 | c.1.7.1 | TIM beta/alpha-barrel | NAD(P)-linked oxidoreductase | Aldo-keto reductases (NADP) | Alpha and beta proteins (a/b) | [GO:0008152  "metabolic process" evidence=ISS] [GO:0016491 "oxidoreductase  activity" evidence=ISS] |
| HP1194 | d.20.1.1 | UBC-like | UBC-like | UBC-related | Alpha and beta proteins (a+b) |  |
| HP1195 | c.37.1.8 | P-loop containing nucleoside triphosphate hydrolases | P-loop containing nucleoside triphosphate hydrolases | G proteins | Alpha and beta proteins (a/b) | [GO:0003746  "translation elongation factor activity" evidence=ISS] [GO:0006414  "translational elongation" evidence=ISS] |
| HP1196 |  |  |  |  |  | [GO:0003735 "structural constituent  of ribosome" evidence=ISS] [GO:0006412 "translation" evidence=ISS]  [GO:0042254 "ribosome biogenesis" evidence=ISS] |
| HP1197 | b.40.4.5 | OB-fold | Nucleic acid-binding proteins | Cold shock DNA-binding domain-like | All beta proteins | [GO:0003735 "structural constituent  of ribosome" evidence=ISS] [GO:0006412 "translation" evidence=ISS]  [GO:0006450 "regulation of translational fidelity" evidence=ISS]  [GO:0042254 "ribosome biogenesis" evidence=ISS] |
| HP1198 | e.29.1.1 | beta and beta-prime subunits of DNA dependent RNA-polymerase | beta and beta-prime subunits of DNA dependent RNA-polymerase | RNA-polymerase beta | Multi-domain proteins (alpha and beta) | [GO:0003899  "DNA-directed RNA polymerase activity" evidence=ISS] |
| HP1199 | d.45.1.1 | ClpS-like | ClpS-like | Ribosomal protein L7/12, C-terminal domain | Alpha and beta proteins (a+b) | [GO:0003735 "structural constituent  of ribosome" evidence=ISS] [GO:0006412 "translation" evidence=ISS] |
| HP1200 |  | Ribosome and ribosomal fragments | Ribosome and ribosomal fragments | Ribosome complexes |  | [GO:0003735 "structural constituent  of ribosome" evidence=ISS] [GO:0006412 "translation" evidence=ISS]  [GO:0042254 "ribosome biogenesis" evidence=ISS] |
| HP1201 | e.24.1.1 | Ribosomal protein L1 | Ribosomal protein L1 | Ribosomal protein L1 | Multi-domain proteins (alpha and beta) | [GO:0003735 "structural constituent  of ribosome" evidence=ISS] [GO:0006412 "translation" evidence=ISS]  [GO:0042254 "ribosome biogenesis" evidence=ISS] |
| HP1202 |  | Ribosome and ribosomal fragments | Ribosome and ribosomal fragments | Ribosome complexes | Low resolution protein structures | [GO:0003735 "structural constituent  of ribosome" evidence=ISS] [GO:0042254 "ribosome biogenesis"  evidence=ISS] |
| HP1203 | b.34.5.4 | SH3-like barrel | Translation proteins SH3-like domain | N-utilization substance G protein NusG, C-terminal domain | All beta proteins | [GO:0006353  "DNA-dependent transcription, termination" evidence=ISS] |
| HP1204 | b.68.1.1 | 6-bladed beta-propeller | Sialidases | Sialidases | All beta proteins | [GO:0003735 "structural constituent  of ribosome" evidence=ISS] [GO:0042254 "ribosome biogenesis"  evidence=ISS] |
| HP1205 | b.43.3.1 | Reductase/isomerase/elongation factor common domain | Translation proteins | Elongation factors | All beta proteins | [GO:0003746  "translation elongation factor activity" evidence=ISS] [GO:0006414  "translational elongation" evidence=ISS] |
| HP1206 |  |  |  |  |  | [GO:0006810  "transport" evidence=ISS] [GO:0016020 "membrane" evidence=ISS]  [GO:0042626 "ATPase activity, coupled to transmembrane movement of  substances" evidence=ISS] |
| HP1207 | c.108.1.6 | HAD-like | HAD-like | beta-Phosphoglucomutase-like | Alpha and beta proteins (a/b) |  |
| HP1208 | c.66.1.28 | S-adenosyl-L-methionine-dependent methyltransferases | S-adenosyl-L-methionine-dependent methyltransferases | N6 adenine-specific DNA methylase, DAM | Alpha and beta proteins (a/b) | [GO:0006306 "DNA methylation" evidence=ISS] [GO:0009007  "site-specific DNA-methyltransferase (adenine-specific) activity"  evidence=ISS] |
| HP1209 | d.81.3.1 | FwdE/GAPDH domain-like | FwdE-like | FwdE-like | Alpha and beta proteins (a+b) | [GO:0004252  "serine-type endopeptidase activity" evidence=ISS] |
| HP1210 | b.81.1.6 | Single-stranded left-handed beta-helix | Trimeric LpxA-like enzymes | Serine acetyltransferase | All beta proteins | [GO:0006535 "cysteine  biosynthetic process from serine" evidence=ISS] [GO:0009001 "serine  O-acetyltransferase activity" evidence=ISS] |
| HP1211 | b.29.1.18 | Concanavalin A-like lectins/glucanases | Concanavalin A-like lectins/glucanases | Alginate lyase | All beta proteins | [GO:0003674  "molecular_function" evidence=ND] [GO:0005575 "cellular_component"  evidence=ND] [GO:0008150 "biological_process" evidence=ND] |
| HP1212 | f.52.1.1 | ATP synthase B chain-like | ATP synthase B chain-like | ATP synthase B chain-like | Membrane and cell surface proteins and peptides | [GO:0015986 "ATP  synthesis coupled proton transport" evidence=ISS] [GO:0045263  "proton-transporting ATP synthase complex, coupling factor F(o)"  evidence=ISS] [GO:0046933 "proton-transporting ATP synthase  activity, rotational mechanism" evidence=ISS] |
| HP1213 | d.14.1.4 | Ribosomal protein S5 domain 2-like | Ribosomal protein S5 domain 2-like | Ribonuclease PH domain 1-like | Alpha and beta proteins (a+b) | [GO:0004654 "polyribonucleotide nucleotidyltransferase  activity" evidence=ISS] [GO:0006402 "mRNA catabolic process"  evidence=ISS] |
| HP1214 | c.61.1.1 | PRTase-like | PRTase-like | Phosphoribosyltransferases (PRTases) | Alpha and beta proteins (a/b) | [GO:0003674  "molecular_function" evidence=ND] [GO:0005575 "cellular_component"  evidence=ND] [GO:0008150 "biological_process" evidence=ND] |
| HP1216 | b.108.1.2 | Triple-stranded beta-helix | Phage fibre proteins | Tail-associated lysozyme gp5, C-terminal domain | All beta proteins | [GO:0003674  "molecular_function" evidence=ND] |
| HP1217 | f.19.1.1 | Aquaporin-like | Aquaporin-like | Aquaporin-like | Membrane and cell surface proteins and peptides | [GO:0003674  "molecular_function" evidence=ND] [GO:0005575 "cellular_component"  evidence=ND] [GO:0008150 "biological_process" evidence=ND] |
| HP1218 | d.142.1.2 | ATP-grasp | Glutathione synthetase ATP-binding domain-like | BC ATP-binding domain-like | Alpha and beta proteins (a+b) | [GO:0004637  "phosphoribosylamine-glycine ligase activity" evidence=ISS]  [GO:0006189 "'de novo' IMP biosynthetic process" evidence=ISS] |
| HP1219 | d.58.5.2 | Ferredoxin-like | GlnB-like | Divalent ion tolerance proteins CutA (CutA1) | Alpha and beta proteins (a+b) | [GO:0005622 "intracellular"  evidence=IEA] [GO:0008270 "zinc ion binding" evidence=IEA] |
| HP1220 | c.37.1.12 | P-loop containing nucleoside triphosphate hydrolases | P-loop containing nucleoside triphosphate hydrolases | ABC transporter ATPase domain-like | Alpha and beta proteins (a/b) | [GO:0005524 "ATP  binding" evidence=ISS] [GO:0006810 "transport" evidence=ISS]  [GO:0042626 "ATPase activity, coupled to transmembrane movement of  substances" evidence=ISS] |
| HP1221 | c.101.1.1 | Undecaprenyl diphosphate synthase | Undecaprenyl diphosphate synthase | Undecaprenyl diphosphate synthase | Alpha and beta proteins (a/b) | [GO:0000287 "magnesium ion  binding" evidence=IDA] [GO:0051301 "cell division" evidence=IEA]  [GO:0009252 "peptidoglycan biosynthetic process" evidence=IEA;IMP] |
| HP1222 | d.145.1.1 | FAD-binding/transporter-associated domain-like | FAD-binding/transporter-associated domain-like | FAD-linked oxidases, N-terminal domain | Alpha and beta proteins (a+b) | [GO:0008152 "metabolic process" evidence=ISS] [GO:0009055 "electron  carrier activity" evidence=ISS] [GO:0016491 "oxidoreductase  activity" evidence=ISS] [GO:0050660 "flavin adenine dinucleotide  binding" evidence=ISS] |
| HP1223 | c.46.1.2 | Rhodanese/Cell cycle control phosphatase | Rhodanese/Cell cycle control phosphatase | Multidomain sulfurtransferase (rhodanese) | Alpha and beta proteins (a/b) | [GO:0003674  "molecular_function" evidence=ND] [GO:0008150 "biological_process"  evidence=ND] |
| HP1224 | c.113.1.1 | HemD-like | HemD-like | HemD-like | Alpha and beta proteins (a/b) | [GO:0004852  "uroporphyrinogen-III synthase activity" evidence=ISS] [GO:0006779  "porphyrin-containing compound biosynthetic process" evidence=ISS] |
| HP1225 | f.36.1.1 | Neurotransmitter-gated ion-channel transmembrane pore | Neurotransmitter-gated ion-channel transmembrane pore | Neurotransmitter-gated ion-channel transmembrane pore | Membrane and cell surface proteins and peptides | [GO:0003674 "molecular_function"  evidence=ND] [GO:0008150 "biological_process" evidence=ND] |
| HP1226 | c.1.28.2 | TIM beta/alpha-barrel | Radical SAM enzymes | Oxygen-independent coproporphyrinogen III oxidase HemN | Alpha and beta proteins (a/b) | [GO:0004109 "coproporphyrinogen oxidase activity" evidence=ISS]  [GO:0006779 "porphyrin-containing compound biosynthetic process"  evidence=ISS] |
| HP1227 | a.3.1.4 | Cytochrome c | Cytochrome c | Two-domain cytochrome c | All alpha proteins | [GO:0009055 "electron carrier  activity" evidence=ISS] |
| HP1228 | d.113.1.1 | Nudix | Nudix | MutT-like | Alpha and beta proteins (a+b) | [GO:0006139  "nucleobase-containing compound metabolic process" evidence=ISS]  [GO:0016818 "hydrolase activity, acting on acid anhydrides, in  phosphorus-containing anhydrides" evidence=ISS] |
| HP1229 | c.73.1.3 | Carbamate kinase-like | Carbamate kinase-like | PyrH-like | Alpha and beta proteins (a/b) | [GO:0004072 "aspartate  kinase activity" evidence=ISS] [GO:0009089 "lysine biosynthetic  process via diaminopimelate" evidence=ISS] |
| HP1230 | c.80.1.3 | SIS domain | SIS domain | mono-SIS domain | Alpha and beta proteins (a/b) | [GO:0003674  "molecular_function" evidence=ND] [GO:0005575 "cellular_component"  evidence=ND] [GO:0008150 "biological_process" evidence=ND] |
| HP1231 | c.37.1.20 | P-loop containing nucleoside triphosphate hydrolases | P-loop containing nucleoside triphosphate hydrolases | Extended AAA-ATPase domain | Alpha and beta proteins (a/b) | [GO:0006260  "DNA replication" evidence=ISS] |
| HP1232 | c.1.21.1 | TIM beta/alpha-barrel | Dihydropteroate synthetase-like | Dihydropteroate synthetase | Alpha and beta proteins (a/b) | [GO:0004156 "dihydropteroate synthase  activity" evidence=ISS] [GO:0009396 "folic acid-containing compound  biosynthetic process" evidence=ISS] |
| HP1234 | a.114.1.1 | Interferon-induced guanylate-binding protein 1 (GBP1), C-terminal domain | Interferon-induced guanylate-binding protein 1 (GBP1), C-terminal domain | Interferon-induced guanylate-binding protein 1 (GBP1), C-terminal domain | All alpha proteins | [GO:0003674 "molecular_function"  evidence=ND] [GO:0008150 "biological_process" evidence=ND] |
| HP1235 | a.118.1.1; | alpha-alpha superhelix | ARM repeat | Armadillo repeat | All alpha proteins | [GO:0003674 "molecular_function"  evidence=ND] [GO:0008150 "biological_process" evidence=ND] |
| HP1236 | a.128.1.4 | Terpenoid synthases | Terpenoid synthases | Aristolochene/pentalenene synthase | All alpha proteins | [GO:0003674  "molecular_function" evidence=ND] [GO:0005575 "cellular_component"  evidence=ND] [GO:0008150 "biological_process" evidence=ND] |
| HP1237 | c.23.16.1 | Flavodoxin-like | Class I glutamine amidotransferase-like | Class I glutamine amidotransferases (GAT) | Alpha and beta proteins (a/b) | [GO:0004088  "carbamoyl-phosphate synthase (glutamine-hydrolyzing) activity"  evidence=ISS] [GO:0009220 "pyrimidine ribonucleotide biosynthetic  process" evidence=ISS] |
| HP1238 | d.160.1.2 | Carbon-nitrogen hydrolase | Carbon-nitrogen hydrolase | Carbamilase | Alpha and beta proteins (a+b) | [GO:0008152  "metabolic process" evidence=ISS] [GO:0016810 "hydrolase activity,  acting on carbon-nitrogen (but not peptide) bonds" evidence=ISS] |
| HP1239 | f.23.30.1 | Single transmembrane helix | Oligosaccharyltransferase subunit ost4p | Oligosaccharyltransferase subunit ost4p | Membrane and cell surface proteins and peptides |  |
| HP1240 | c.51.4.2 | Anticodon-binding domain-like | ITPase-like | Maf-like | Alpha and beta proteins (a/b) | [GO:0003674 "molecular_function"  evidence=ND] |
| HP1241 | a.203.1.1 | Putative anticodon-binding domain of alanyl-tRNA synthetase (AlaRS) | Putative anticodon-binding domain of alanyl-tRNA synthetase (AlaRS) | Putative anticodon-binding domain of alanyl-tRNA synthetase (AlaRS) | All alpha proteins | [GO:0004813 "alanine-tRNA ligase  activity" evidence=ISS] [GO:0006419 "alanyl-tRNA aminoacylation"  evidence=ISS] |
| HP1242 | f.36.1.1 | Neurotransmitter-gated ion-channel transmembrane pore | Neurotransmitter-gated ion-channel transmembrane pore | Neurotransmitter-gated ion-channel transmembrane pore | Membrane and cell surface proteins and peptides | [GO:0003674  "molecular_function" evidence=ND] [GO:0005575 "cellular_component"  evidence=ND] [GO:0008150 "biological_process" evidence=ND] |
| HP1243 | f.4.1.1 | TRANSMEMBRANE BETA BAREELS | OMPA LIKE | OUTERMEMBRANE PROTEINS | Membrane and cell surface proteins and peptides | [GO:0000920 "cytokinetic  cell separation" evidence=NAS] [GO:0005576 "extracellular region"  evidence=IEA] [GO:0005634 "nucleus" evidence=IDA] [GO:0005829  "cytosol" evidence=IDA] [GO:0008422 "beta-glucosidase activity"  evidence=ISM] |
| HP1244 | a.4.8.1 | DNA/RNA-binding 3-helical bundle | Ribosomal protein S18 | Ribosomal protein S18 | All alpha proteins | [GO:0003735 "structural constituent  of ribosome" evidence=ISS] [GO:0042254 "ribosome biogenesis"  evidence=ISS] |
| HP1245 | b.40.4.3 | OB-fold | Nucleic acid-binding proteins | Single strand DNA-binding domain, SSB | All beta proteins | [GO:0003697  "single-stranded DNA binding" evidence=ISS] [GO:0006260 "DNA  replication" evidence=ISS] [GO:0006281 "DNA repair" evidence=ISS]  [GO:0006310 "DNA recombination" evidence=ISS] |
| HP1246 |  | Ribosome and ribosomal fragments | Ribosome and ribosomal fragments | Ribosome complexes | Low resolution protein structures | [GO:0003735 "structural constituent  of ribosome" evidence=ISS] [GO:0042254 "ribosome biogenesis"  evidence=ISS] |
| HP1247 | c.37.1.20 | P-loop containing nucleoside triphosphate hydrolases | P-loop containing nucleoside triphosphate hydrolases | Extended AAA-ATPase domain | Alpha and beta proteins (a/b) | [GO:0003674  "molecular_function" evidence=ND] [GO:0005575 "cellular_component"  evidence=ND] [GO:0008150 "biological_process" evidence=ND] |
| HP1248 | b.40.4.16 | OB-fold | Nucleic acid-binding proteins | RNB domain-like | All beta proteins | [GO:0003674 "molecular_function"  evidence=ND] [GO:0005575 "cellular_component" evidence=ND]  [GO:0008150 "biological_process" evidence=ND] |
| HP1249 | c.2.1.7 | NAD(P)-binding Rossmann-fold domains | NAD(P)-binding Rossmann-fold domains | Aminoacid dehydrogenase-like, C-terminal domain | Alpha and beta proteins (a/b) | [GO:0004764 "shikimate  3-dehydrogenase (NADP+) activity" evidence=ISS] [GO:0009423  "chorismate biosynthetic process" evidence=ISS] |
| HP1250 | d.3.1.16 | Cysteine proteinases | Cysteine proteinases | NlpC/P60 | Alpha and beta proteins (a+b) |  |
| HP1251 | f.58.1.1 | MetI-like | MetI-like | MetI-like | Membrane and cell surface proteins and peptides | [GO:0015197 "peptide transporter activity" evidence=ISS]  [GO:0015833 "peptide transport" evidence=ISS] [GO:0042626 "ATPase  activity, coupled to transmembrane movement of substances"  evidence=ISS] |
| HP1252 | c.94.1.1 | Periplasmic binding protein-like II | Periplasmic binding protein-like II | Phosphate binding protein-like | Alpha and beta proteins (a/b) | [GO:0015197 "peptide transporter  activity" evidence=ISS] [GO:0015833 "peptide transport"  evidence=ISS] [GO:0042626 "ATPase activity, coupled to  transmembrane movement of substances" evidence=ISS] |
| HP1253 | c.26.1.1 | Adenine nucleotide alpha hydrolase-like | Nucleotidylyl transferase | Class I aminoacyl-tRNA synthetases (RS), catalytic domain | Alpha and beta proteins (a/b) | [GO:0004830  "tryptophan-tRNA ligase activity" evidence=ISS] [GO:0006436  "tryptophanyl-tRNA aminoacylation" evidence=ISS] |
| HP1254 | c.66.1.41 | S-adenosyl-L-methionine-dependent methyltransferases | S-adenosyl-L-methionine-dependent methyltransferases | UbiE/COQ5-like | Alpha and beta proteins (a/b) | [GO:0003824  "catalytic activity" evidence=ISS] [GO:0009102 "biotin biosynthetic  process" evidence=ISS] |
| HP1255 | a.24.3.2 | Four-helical up-and-down bundle | Cytochromes | Cytochrome c'-like | All alpha proteins | [GO:0009306 "protein  secretion" evidence=ISS] [GO:0015450 "P-P-bond-hydrolysis-driven  protein transmembrane transporter activity" evidence=ISS] |
| HP1256 | d.67.3.1 | RRF/tRNA synthetase additional domain-like | Ribosome recycling factor, RRF | Ribosome recycling factor, RRF | Alpha and beta proteins (a+b) | [GO:0006412 "translation"  evidence=ISS] [GO:0008135 "translation factor activity, nucleic  acid binding" evidence=ISS] |
| HP1257 | c.61.1.1 | PRTase-like | PRTase-like | Phosphoribosyltransferases (PRTases) | Alpha and beta proteins (a/b) | [GO:0004588 "orotate  phosphoribosyltransferase activity" evidence=ISS] [GO:0009220  "pyrimidine ribonucleotide biosynthetic process" evidence=ISS] |
| HP1258 | c.9.1.1 | Barstar-like | Barstar-related | Barstar-related | Alpha and beta proteins (a/b) | [GO:0003674  "molecular_function" evidence=ND] [GO:0005575 "cellular_component"  evidence=ND] [GO:0008150 "biological_process" evidence=ND] |
| HP1259 | c.31.1.5 | DHS-like NAD/FAD-binding domain | DHS-like NAD/FAD-binding domain | Sir2 family of transcriptional regulators | Alpha and beta proteins (a/b) | [GO:0008152 "metabolic process" evidence=ISS]  [GO:0019213 "deacetylase activity" evidence=ISS] |
| HP1260 |  |  |  |  |  | [GO:0050136 "NADH  dehydrogenase (quinone) activity" evidence=ISS] |
| HP1261 | e.19.1.2 | HydA/Nqo6-like | HydA/Nqo6-like | Nqo6-like | Multi-domain proteins (alpha and beta) | [GO:0050136 "NADH  dehydrogenase (quinone) activity" evidence=ISS] |
| HP1262 | d.307.1.1 | Nqo5-like | Nqo5-like | Nqo5-like | Alpha and beta proteins (a+b) | [GO:0050136 "NADH  dehydrogenase (quinone) activity" evidence=ISS] |
| HP1263 | e.18.1.2 | HydB/Nqo4-like | HydB/Nqo4-like | Nqo4-like | Multi-domain proteins (alpha and beta) | [GO:0050136 "NADH  dehydrogenase (quinone) activity" evidence=ISS] |
| HP1264 | d.68.3.3 | IF3-like | SirA-like | SirA-like | Alpha and beta proteins (a+b) | [GO:0003674  "molecular_function" evidence=ND] [GO:0005575 "cellular_component"  evidence=ND] [GO:0008150 "biological_process" evidence=ND] |
| HP1265 | c.81.1.1 | Formate dehydrogenase/DMSO reductase, domains 1-3 | Formate dehydrogenase/DMSO reductase, domains 1-3 | Formate dehydrogenase/DMSO reductase, domains 1-3 | Alpha and beta proteins (a/b) |  |
| HP1266 | c.81.1.1 | Formate dehydrogenase/DMSO reductase, domains 1-3 | Formate dehydrogenase/DMSO reductase, domains 1-3 | Formate dehydrogenase/DMSO reductase, domains 1-3 | Alpha and beta proteins (a/b) | [GO:0050136 "NADH  dehydrogenase (quinone) activity" evidence=ISS] |
| HP1267 | a.177.1.1 | Sigma2 domain of RNA polymerase sigma factors | Sigma2 domain of RNA polymerase sigma factors | Sigma2 domain of RNA polymerase sigma factors | All alpha proteins | [GO:0050136 "NADH  dehydrogenase (quinone) activity" evidence=ISS] |
| HP1268 | d.58.1.5 | Ferredoxin-like | 4Fe-4S ferredoxins | Ferredoxin domains from multidomain proteins | Alpha and beta proteins (a+b) | [GO:0050136 "NADH  dehydrogenase (quinone) activity" evidence=ISS] |
| HP1269 |  |  |  |  |  | [GO:0050136 "NADH  dehydrogenase (quinone) activity" evidence=ISS] |
| HP1270 |  |  |  |  |  | [GO:0006120  "mitochondrial electron transport, NADH to ubiquinone"  evidence=ISS] [GO:0008137 "NADH dehydrogenase (ubiquinone)  activity" evidence=ISS] |
| HP1271 |  |  |  |  |  | [GO:0050136 "NADH  dehydrogenase (quinone) activity" evidence=ISS] |
| HP1272 |  |  |  |  |  | [GO:0006120  "mitochondrial electron transport, NADH to ubiquinone"  evidence=ISS] [GO:0008137 "NADH dehydrogenase (ubiquinone)  activity" evidence=ISS] |
| HP1273 |  |  |  |  |  | [GO:0006120  "mitochondrial electron transport, NADH to ubiquinone"  evidence=ISS] [GO:0008137 "NADH dehydrogenase (ubiquinone)  activity" evidence=ISS] |
| HP1274 | a.118.1.2; | alpha-alpha superhelix | ARM repeat | HEAT repeat | All alpha proteins | [GO:0001539 "ciliary  or flagellar motility" evidence=ISS] [GO:0005198 "structural  molecule activity" evidence=ISS] [GO:0007155 "cell adhesion"  evidence=ISS] |
| HP1275 | c.84.1.1 | Phosphoglucomutase, first 3 domains | Phosphoglucomutase, first 3 domains | Phosphoglucomutase, first 3 domains | Alpha and beta proteins (a/b) | [GO:0004614  "phosphoglucomutase activity" evidence=ISS] [GO:0004615  "phosphomannomutase activity" evidence=ISS] [GO:0009244  "lipopolysaccharide core region biosynthetic process" evidence=ISS] |
| HP1276 | b.40.4.5 | OB-fold | Nucleic acid-binding proteins | Cold shock DNA-binding domain-like | All beta proteins | [GO:0016887  "ATPase activity" evidence=IEA] [GO:0005813 "centrosome"  evidence=IEA] [GO:0005730 "nucleolus" evidence=IEA] |
| HP1277 | c.1.2.4 | TIM beta/alpha-barrel | Ribulose-phoshate binding barrel | Tryptophan biosynthesis enzymes | Alpha and beta proteins (a/b) | [GO:0005515 "protein binding" evidence=IPI] |
| HP1278 | c.79.1.1 | Tryptophan synthase beta subunit-like PLP-dependent enzymes | Tryptophan synthase beta subunit-like PLP-dependent enzymes | Tryptophan synthase beta subunit-like PLP-dependent enzymes | Alpha and beta proteins (a/b) | [GO:0000162  "tryptophan biosynthetic process" evidence=ISS] [GO:0004834  "tryptophan synthase activity" evidence=ISS] |
| HP1279 | c.1.2.4 | TIM beta/alpha-barrel | Ribulose-phoshate binding barrel | Tryptophan biosynthesis enzymes | Alpha and beta proteins (a/b) | [GO:0000162 "tryptophan biosynthetic process" evidence=ISS]  [GO:0004425 "indole-3-glycerol-phosphate synthase activity"  evidence=ISS] [GO:0004640 "phosphoribosylanthranilate isomerase  activity" evidence=ISS] |
| HP1280 | c.27.1.1 | Nucleoside phosphorylase/phosphoribosyltransferase catalytic domain | Nucleoside phosphorylase/phosphoribosyltransferase catalytic domain | Nucleoside phosphorylase/phosphoribosyltransferase catalytic domain | Alpha and beta proteins (a/b) | [GO:0000162  "tryptophan biosynthetic process" evidence=ISS] [GO:0004048  "anthranilate phosphoribosyltransferase activity" evidence=ISS] |
| HP1281 | c.23.16.1 | Flavodoxin-like | Class I glutamine amidotransferase-like | Class I glutamine amidotransferases | Alpha and beta proteins (a/b) | [GO:0000162  "tryptophan biosynthetic process" evidence=ISS] [GO:0016884  "carbon-nitrogen ligase activity, with glutamine as amido-N-donor"  evidence=ISS] |
| HP1282 | d.161.1.1 | ADC synthase | ADC synthase | ADC synthase | Alpha and beta proteins (a+b) | [GO:0000162  "tryptophan biosynthetic process" evidence=ISS] [GO:0004049  "anthranilate synthase activity" evidence=ISS] |
| HP1283 | c.47.1.1 | Thioredoxin fold | Thioredoxin-like | Thioltransferase | Alpha and beta proteins (a/b) |  |
| HP1284 | c.87.1.7 | UDP-Glycosyltransferase/glycogen phosphorylase | UDP-Glycosyltransferase/glycogen phosphorylase | ADP-heptose LPS heptosyltransferase II | Alpha and beta proteins (a/b) | [GO:0008920 "lipopolysaccharide heptosyltransferase  activity" evidence=ISS] [GO:0009244 "lipopolysaccharide core region  biosynthetic process" evidence=ISS] |
| HP1285 | c.108.1.6 | HAD-like | HAD-like | beta-Phosphoglucomutase-like | Alpha and beta proteins (a/b) | [GO:0003993 "acid phosphatase  activity" evidence=ISS] [GO:0008152 "metabolic process"  evidence=ISS] |
| HP1286 | b.61.6.1 | Streptavidin-like | YceI-like | YceI-like | All beta proteins | [GO:0003674  "molecular_function" evidence=ND] [GO:0005575 "cellular_component"  evidence=ND] [GO:0008150 "biological_process" evidence=ND] |
| HP1287 | a.132.1.3 | Heme oxygenase-like | Heme oxygenase-like | TENA/THI-4 | All alpha proteins | [GO:0006355  "regulation of transcription, DNA-dependent" evidence=ISS]  [GO:0030436 "asexual sporulation" evidence=ISS] |
| HP1288 | b.82.2.11 | Double-stranded beta-helix | Clavaminate synthase-like | Asparaginyl hydroxylase-like | All beta proteins |  |
| HP1289 | c.60.1.2 | Phosphoglycerate mutase-like | Phosphoglycerate mutase-like | Histidine acid phosphatase | Alpha and beta proteins (a/b) |  |
| HP1290 | b.56.1.1 | Transcription factor IIA (TFIIA), beta-barrel domain | Transcription factor IIA (TFIIA), beta-barrel domain | Transcription factor IIA (TFIIA), beta-barrel domain | All beta proteins | [GO:0005337  "nucleoside transmembrane transporter activity" evidence=ISS]  [GO:0015858 "nucleoside transport" evidence=ISS] |
| HP1291 | c.100.1.1 | Thiamin pyrophosphokinase, catalytic domain | Thiamin pyrophosphokinase, catalytic domain | Thiamin pyrophosphokinase, catalytic domain | Alpha and beta proteins (a/b) | [GO:0004788 "thiamine diphosphokinase activity" evidence=ISS]  [GO:0006772 "thiamine metabolic process" evidence=ISS] |
| HP1292 |  | Ribosome and ribosomal fragments | Ribosome and ribosomal fragments | Large subunit | Low resolution protein structures | [GO:0003735 "structural constituent  of ribosome" evidence=ISS] |
| HP1293 | d.181.1.1 | Insert subdomain of RNA polymerase alpha subunit | Insert subdomain of RNA polymerase alpha subunit | Insert subdomain of RNA polymerase alpha subunit | Alpha and beta proteins (a+b) | [GO:0003899  "DNA-directed RNA polymerase activity" evidence=ISS] |
| HP1294 | d.66.1.2 | Alpha-L RNA-binding motif | Alpha-L RNA-binding motif | Ribosomal protein S4 | Alpha and beta proteins (a+b) | [GO:0003735 "structural constituent  of ribosome" evidence=ISS] [GO:0006412 "translation" evidence=ISS]  [GO:0006450 "regulation of translational fidelity" evidence=ISS]  [GO:0042254 "ribosome biogenesis" evidence=ISS] |
| HP1295 | c.55.4.1 | Ribonuclease H-like motif | Translational machinery components | Ribosomal protein L18 and S11 | Alpha and beta proteins (a/b) | [GO:0003735 "structural constituent  of ribosome" evidence=ISS] [GO:0042254 "ribosome biogenesis"  evidence=ISS] |
| HP1296 | a.156.1.1 | S13-like H2TH domain | S13-like H2TH domain | Ribosomal protein S13 | All alpha proteins | [GO:0003735 "structural constituent  of ribosome" evidence=ISS] [GO:0042254 "ribosome biogenesis"  evidence=ISS] |
| HP1297 | g.42.1.1 | Ribosomal protein L36 | Ribosomal protein L36 | Ribosomal protein L36 | Small proteins | [GO:0003735 "structural constituent  of ribosome" evidence=ISS] [GO:0042254 "ribosome biogenesis"  evidence=ISS] |
| HP1298 | b.40.4.5 | OB-fold | Nucleic acid-binding proteins | Cold shock DNA-binding domain-like | All beta proteins | [GO:0003743  "translation initiation factor activity" evidence=ISS] [GO:0006413  "translational initiation" evidence=ISS] |
| HP1299 | d.127.1.1 | Creatinase/aminopeptidase | Creatinase/aminopeptidase | Creatinase/aminopeptidase | Alpha and beta proteins (a+b) | [GO:0006464 "cellular  protein modification process" evidence=ISS] |
| HP1300 | f.24.1.1 | Cytochrome c oxidase subunit I-like | Cytochrome c oxidase subunit I-like | Cytochrome c oxidase subunit I-like | Membrane and cell surface proteins and peptides | [GO:0006886  "intracellular protein transport" evidence=ISS] [GO:0008565  "protein transporter activity" evidence=ISS] [GO:0016020 "membrane"  evidence=ISS] |
| HP1301 |  | Ribosome and ribosomal fragments | Ribosome and ribosomal fragments | Large subunit | Low resolution protein structures | [GO:0003735 "structural constituent  of ribosome" evidence=ISS] |
| HP1302 | d.50.1.2 | dsRBD-like | dsRNA-binding domain-like | Ribosomal S5 protein, N-terminal domain | Alpha and beta proteins (a+b) | [GO:0003735 "structural constituent  of ribosome" evidence=ISS] [GO:0006412 "translation" evidence=ISS]  [GO:0006450 "regulation of translational fidelity" evidence=ISS]  [GO:0042254 "ribosome biogenesis" evidence=ISS] |
| HP1303 | c.56.5.4 | Phosphorylase/hydrolase-like | Zn-dependent exopeptidases | Bacterial dinuclear zinc exopeptidases | Alpha and beta proteins (a/b) | [GO:0003735 "structural constituent  of ribosome" evidence=ISS] [GO:0042254 "ribosome biogenesis"  evidence=ISS] |
| HP1304 | b.67.2.1 | -bladed beta-propeller | Arabinanase/levansucrase/invertase | alpha-L-arabinanase-like | All beta proteins | [GO:0003735 "structural constituent  of ribosome" evidence=ISS] [GO:0006412 "translation" evidence=ISS]  [GO:0042254 "ribosome biogenesis" evidence=ISS] |
| HP1305 |  | Ribosome and ribosomal fragments | Ribosome and ribosomal fragments | Large subunit | Low resolution protein structures | [GO:0003735 "structural constituent  of ribosome" evidence=ISS] [GO:0042254 "ribosome biogenesis"  evidence=ISS] |
| HP1306 | g.39.1.7 | Glucocorticoid receptor-like (DNA-binding domain) | Glucocorticoid receptor-like (DNA-binding domain) | Ribosomal protein S14 | Small proteins | [GO:0003735 "structural constituent  of ribosome" evidence=ISS] [GO:0042254 "ribosome biogenesis"  evidence=ISS] |
| HP1307 | d.77.1.1 | RL5-like | RL5-like | Ribosomal protein L5 | Alpha and beta proteins (a+b) | [GO:0003735 "structural constituent  of ribosome" evidence=ISS] [GO:0042254 "ribosome biogenesis"  evidence=ISS] |
| HP1308 | c.81.1.1 | Formate dehydrogenase/DMSO reductase, domains 1-3 | Formate dehydrogenase/DMSO reductase, domains 1-3 | Formate dehydrogenase/DMSO reductase, domains 1-3 | Alpha and beta proteins (a/b) | [GO:0003735 "structural constituent  of ribosome" evidence=ISS] [GO:0042254 "ribosome biogenesis"  evidence=ISS] |
| HP1309 | b.39.1.1 | Ribosomal protein L14 | Ribosomal protein L14 | Ribosomal protein L14 | All beta proteins | [GO:0003735 "structural constituent  of ribosome" evidence=ISS] [GO:0042254 "ribosome biogenesis"  evidence=ISS] |
| HP1310 | b.40.4.5 | OB-fold | Nucleic acid-binding proteins | Cold shock DNA-binding domain-like | All beta proteins | [GO:0003735 "structural constituent  of ribosome" evidence=ISS] [GO:0006412 "translation" evidence=ISS]  [GO:0042254 "ribosome biogenesis" evidence=ISS] |
| HP1311 | f.21.1.2 | Heme-binding four-helical bundle | Transmembrane di-heme cytochromes | Cytochrome b of cytochrome bc1 complex (Ubiquinol-cytochrome c reductase) | Membrane and cell surface proteins and peptides | [GO:0003735 "structural constituent  of ribosome" evidence=ISS] [GO:0042254 "ribosome biogenesis"  evidence=ISS] |
| HP1312 | d.41.4.2 | alpha/beta-Hammerhead | Ribosomal protein L16p/L10e | Ribosomal protein L16p | Alpha and beta proteins (a+b) | [GO:0003735 "structural constituent  of ribosome" evidence=ISS] [GO:0006412 "translation" evidence=ISS]  [GO:0042254 "ribosome biogenesis" evidence=ISS] |
| HP1313 | d.53.1.1 | Ribosomal protein S3 C-terminal domain | Ribosomal protein S3 C-terminal domain | Ribosomal protein S3 C-terminal domain | Alpha and beta proteins (a+b) | [GO:0003735 "structural constituent  of ribosome" evidence=ISS] [GO:0006412 "translation" evidence=ISS]  [GO:0042254 "ribosome biogenesis" evidence=ISS] |
| HP1314 | d.55.1.1 | Ribosomal protein L22 | Ribosomal protein L22 | Ribosomal protein L22 | Alpha and beta proteins (a+b) | [GO:0003735 "structural constituent  of ribosome" evidence=ISS] [GO:0009292 "genetic transfer"  evidence=ISS] [GO:0042254 "ribosome biogenesis" evidence=ISS] |
| HP1315 | d.28.1.1 | Ribosomal protein S19 | Ribosomal protein S19 | Ribosomal protein S19 | Alpha and beta proteins (a+b) | [GO:0003735 "structural constituent  of ribosome" evidence=ISS] [GO:0042254 "ribosome biogenesis"  evidence=ISS] |
| HP1316 | b.40.4.5 | OB-fold | Nucleic acid-binding proteins | Cold shock DNA-binding domain-like | All beta proteins | [GO:0003735 "structural constituent  of ribosome" evidence=ISS] [GO:0006412 "translation" evidence=ISS]  [GO:0015934 "large ribosomal subunit" evidence=ISS] [GO:0042254  "ribosome biogenesis" evidence=ISS] |
| HP1317 |  | Ribosome and ribosomal fragments | Ribosome and ribosomal fragments | Large subunit | Low resolution protein structures | [GO:0003735 "structural constituent of  ribosome" evidence=ISS] [GO:0042255 "ribosome assembly"  evidence=ISS] |
| HP1318 | c.22.1.1 | Ribosomal protein L4 | Ribosomal protein L4 | Ribosomal protein L4 | Alpha and beta proteins (a/b) | [GO:0003735 "structural constituent  of ribosome" evidence=ISS] [GO:0042254 "ribosome biogenesis"  evidence=ISS] |
| HP1319 |  | Ribosome and ribosomal fragments | Ribosome and ribosomal fragments | Large subunit | Low resolution protein structures | [GO:0003735 "structural constituent  of ribosome" evidence=ISS] [GO:0042254 "ribosome biogenesis"  evidence=ISS] |
| HP1320 | d.58.15.1 | Ferredoxin-like | Ribosomal protein S10 | Ribosomal protein S10 | Alpha and beta proteins (a+b) | [GO:0003735 "structural constituent  of ribosome" evidence=ISS] [GO:0006412 "translation" evidence=ISS] |
| HP1321 | c.37.1.20 | P-loop containing nucleoside triphosphate hydrolases | P-loop containing nucleoside triphosphate hydrolases | Extended AAA-ATPase domain | Alpha and beta proteins (a/b) | [GO:0003674  "molecular_function" evidence=ND] [GO:0005575 "cellular_component"  evidence=ND] [GO:0008150 "biological_process" evidence=ND] |
| HP1322 | d.110.2.1 | Profilin-like | GAF domain-like | GAF domain | Alpha and beta proteins (a+b) |  |
| HP1323 | c.55.3.1 | Ribonuclease H-like motif | Ribonuclease H-like | Ribonuclease H | Alpha and beta proteins (a/b) | [GO:0004540 "ribonuclease activity"  evidence=ISS] [GO:0006401 "RNA catabolic process" evidence=ISS] |
| HP1324 | c.55.3.2 | Ribonuclease H-like motif | Ribonuclease H-like | Retroviral integrase, catalytic domain | Alpha and beta proteins (a/b) | [GO:0003674 "molecular_function" evidence=ND] [GO:0005634  "nucleus" evidence=ISM] [GO:0005777 "peroxisome" evidence=IEA;ISS]  [GO:0007031 "peroxisome organization" evidence=ISS;IMP] [GO:0005829  "cytosol" evidence=IDA] |
| HP1325 | a.127.1.1 | L-aspartase-like | L-aspartase-like | L-aspartase/fumarase | All alpha proteins | [GO:0004333 "fumarate  hydratase activity" evidence=ISS] [GO:0006099 "tricarboxylic acid  cycle" evidence=ISS] |
| HP1326 | b.1.18.10 | Immunoglobulin-like beta-sandwich | E set domains | Filamin repeat (rod domain) | All beta proteins |  |
| HP1327 | f.5.1.1 | Outer membrane efflux proteins (OEP) | Outer membrane efflux proteins (OEP) | Outer membrane efflux proteins (OEP) | Membrane and cell surface proteins and peptides | [GO:0005096 "GTPase  activator activity" evidence=ISS] [GO:0005737 "cytoplasm"  evidence=IEA] [GO:0005100 "Rho GTPase activator activity"  evidence=IDA] [GO:0045887 "positive regulation of synaptic growth  at neuromuscular junction" evidence=IMP] |
| HP1328 | f.46.1.1 | HlyD-like secretion proteins | HlyD-like secretion proteins | HlyD-like secretion proteins | Membrane and cell surface proteins and peptides | [GO:0005375 "copper  ion transmembrane transporter activity" evidence=ISS] [GO:0006825  "copper ion transport" evidence=ISS] [GO:0010038 "response to metal  ion" evidence=ISS] [GO:0015080 "silver ion transmembrane  transporter activity" evidence=ISS] [GO:0015673 "silver ion  transport" evidence=ISS] |
| HP1329 | f.35.1.1 | Multidrug efflux transporter AcrB transmembrane domain | Multidrug efflux transporter AcrB transmembrane domain | Multidrug efflux transporter AcrB transmembrane domain | Membrane and cell surface proteins and peptides | [GO:0030001  "metal ion transport" evidence=ISS] [GO:0046873 "metal ion  transmembrane transporter activity" evidence=ISS] |
| HP1330 | f.1.1.1 | Toxins' membrane translocation domains | Colicin | Colicin | Membrane and cell surface proteins and peptides | [GO:0005524 "ATP binding"  evidence=IEA] [GO:0042626 "ATPase activity, coupled to  transmembrane movement of substances" evidence=IEA] [GO:0016021  "integral to membrane" evidence=IEA] [GO:0005634 "nucleus"  evidence=NAS] |
| HP1331 | f.23.13.1 | Single transmembrane helix | Ubiquinone-binding protein QP-C of cytochrome bc1 complex (Ubiquinol-cytochrome c reductase) | Ubiquinone-binding protein QP-C of cytochrome bc1 complex (Ubiquinol-cytochrome c reductase) | Membrane and cell surface proteins and peptides | [GO:0015658 "branched-chain amino acid  transmembrane transporter activity" evidence=ISS] [GO:0015803  "branched-chain amino acid transport" evidence=ISS] [GO:0016020  "membrane" evidence=ISS] |
| HP1332 | b.4.1.1 | HSP40/DnaJ peptide-binding domain | HSP40/DnaJ peptide-binding domain | HSP40/DnaJ peptide-binding domain | All beta proteins | [GO:0006457 "protein folding"  evidence=ISS] [GO:0006950 "response to stress" evidence=ISS] |
| HP1333 | a.238.1.4 | BAR/IMD domain-like | BAR/IMD domain-like | FCH domain | All alpha proteins | [GO:0007130 "synaptonemal complex assembly"  evidence=IEA] [GO:0000795 "synaptonemal complex" evidence=IEA] |
| HP1334 | c.1.12.7 | TIM beta/alpha-barrel | Phosphoenolpyruvate/pyruvate domain | Phosphoenolpyruvate mutase/Isocitrate lyase-like | Alpha and beta proteins (a/b) | [GO:0003674  "molecular_function" evidence=ND] [GO:0005575 "cellular_component"  evidence=ND] [GO:0008150 "biological_process" evidence=ND] |
| HP1335 | c.26.2.5 | Adenine nucleotide alpha hydrolase-like | Adenine nucleotide alpha hydrolase-like | PP-loop ATPase | Alpha and beta proteins (a/b) | [GO:0004808 "tRNA  (5-methylaminomethyl-2-thiouridylate)-methyltransferase activity"  evidence=ISS] [GO:0006396 "RNA processing" evidence=ISS] |
| HP1336 | c.47.1.2 | Thioredoxin fold | Thioredoxin-like | PDI-like | Alpha and beta proteins (a/b) | [GO:0031314 "extrinsic to  mitochondrial inner membrane" evidence=IEA] [GO:0006458 "'de novo'  protein folding" evidence=IEA] [GO:0000002 "mitochondrial genome  maintenance" evidence=IEA] [GO:0006515 "misfolded or incompletely  synthesized protein catabolic process" evidence=IEA] |
| HP1337 | c.26.1.3 | Adenine nucleotide alpha hydrolase-like | Nucleotidylyl transferase | Adenylyltransferase | Alpha and beta proteins (a/b) | [GO:0000309 "nicotinamide-nucleotide adenylyltransferase activity"  evidence=ISS] [GO:0004515 "nicotinate-nucleotide  adenylyltransferase activity" evidence=ISS] [GO:0009435 "NAD  biosynthetic process" evidence=ISS] |
| HP1338 | d.58.18.1 | Ferredoxin-like | ACT-like | Phosphoglycerate dehydrogenase, regulatory (C-terminal) domain | Alpha and beta proteins (a+b) | [GO:0003700  "sequence-specific DNA binding transcription factor activity"  evidence=ISS] [GO:0006355 "regulation of transcription,  DNA-dependent" evidence=ISS] |
| HP1339 | d.42.1.2 | POZ domain | POZ domain | Tetramerization domain of potassium channels | Alpha and beta proteins (a+b) | [GO:0005215  "transporter activity" evidence=ISS] [GO:0006810 "transport"  evidence=ISS] |
| HP1340 | c.81.1.1 | Formate dehydrogenase/DMSO reductase, domains 1-3 | Formate dehydrogenase/DMSO reductase, domains 1-3 | Formate dehydrogenase/DMSO reductase, domains 1-3 | Alpha and beta proteins (a/b) | [GO:0005215  "transporter activity" evidence=ISS] [GO:0006810 "transport"  evidence=ISS] |
| HP1341 | d.212.1.2 | TolA/TonB C-terminal domain | TolA/TonB C-terminal domain | TonB | Alpha and beta proteins (a+b) | [GO:0005215 "transporter activity"  evidence=ISS] [GO:0006810 "transport" evidence=ISS] |
| HP1342 | f.4.1.1 | TRANSMEMBRANE BETA BAREELS | OMPA LIKE | OUTERMEMBRANE PROTEINS | Membrane and cell surface proteins and peptides | [GO:0046872  "metal ion binding" evidence=IEA] [GO:0000932 "cytoplasmic mRNA  processing body" evidence=IEA;IDA] [GO:0016787 "hydrolase activity"  evidence=IEA;IDA] [GO:0000290 "deadenylation-dependent decapping of  nuclear-transcribed mRNA" evidence=IDA;IMP] |
| HP1343 | b.150.1.1 | Putative glucosidase YicI, C-terminal domain | Putative glucosidase YicI, C-terminal domain | Putative glucosidase YicI, C-terminal domain | All beta proteins | [GO:0003674  "molecular_function" evidence=ND] [GO:0008150 "biological_process"  evidence=ND] |
| HP1344 |  |  |  |  |  | [GO:0006824  "cobalt ion transport" evidence=ISS] [GO:0015087 "cobalt ion  transmembrane transporter activity" evidence=ISS] [GO:0015095  "magnesium ion transmembrane transporter activity" evidence=ISS] |
| HP1345 | c.86.1.1 | Phosphoglycerate kinase | Phosphoglycerate kinase | Phosphoglycerate kinase | Alpha and beta proteins (a/b) | [GO:0004618 "phosphoglycerate kinase  activity" evidence=ISS] [GO:0006096 "glycolysis" evidence=ISS] |
| HP1346 | d.81.1.1 | FwdE/GAPDH domain-like | Glyceraldehyde-3-phosphate dehydrogenase-like, C-terminal domain | GAPDH-like | Alpha and beta proteins (a+b) | [GO:0006094  "gluconeogenesis" evidence=ISS] [GO:0006096 "glycolysis"  evidence=ISS] [GO:0019682 "glyceraldehyde-3-phosphate metabolic  process" evidence=ISS] |
| HP1347 | c.18.1.1 | Uracil-DNA glycosylase-like | Uracil-DNA glycosylase-like | Uracil-DNA glycosylase-like | Alpha and beta proteins (a/b) | [GO:0004844 "uracil DNA N-glycosylase  activity" evidence=ISS] [GO:0006281 "DNA repair" evidence=ISS] |
| HP1348 | c.112.1.1 | Glycerol-3-phosphate (1)-acyltransferase | Glycerol-3-phosphate (1)-acyltransferase | Glycerol-3-phosphate (1)-acyltransferase | Alpha and beta proteins (a/b) | [GO:0003841 "1-acylglycerol-3-phosphate O-acyltransferase  activity" evidence=ISS] [GO:0008654 "phospholipid biosynthetic  process" evidence=ISS] |
| HP1349 | b.34.2.1 | SH3-like barrel | SH3-domain | SH3-domain | All beta proteins | [GO:0003674 "molecular_function"  evidence=ND] [GO:0005575 "cellular_component" evidence=ND]  [GO:0008150 "biological_process" evidence=ND] |
| HP1350 | c.14.1.2 | ClpP/crotonase | ClpP/crotonase | Tail specific protease, catalytic domain | Alpha and beta proteins (a/b) | [GO:0006508  "proteolysis" evidence=ISS] |
| HP1351 | c.52.1.30 | Restriction endonuclease-like | Restriction endonuclease-like | MRR-like | Alpha and beta proteins (a/b) | [GO:0003674 "molecular_function" evidence=ND]  [GO:0008150 "biological_process" evidence=ND] |
| HP1352 | c.66.1.11 | S-adenosyl-L-methionine-dependent methyltransferases | S-adenosyl-L-methionine-dependent methyltransferases | Type II DNA methylase | Alpha and beta proteins (a/b) | [GO:0006306 "DNA methylation"  evidence=ISS] [GO:0009008 "DNA-methyltransferase activity"  evidence=ISS] |
| HP1353 | c.1.19.2 | TIM beta/alpha-barrel | Cobalamin (vitamin B12)-dependent enzymes | Glutamate mutase, large subunit | Alpha and beta proteins (a/b) | [GO:0006306 "DNA methylation" evidence=ISS] [GO:0009007  "site-specific DNA-methyltransferase (adenine-specific) activity"  evidence=ISS] |
| HP1354 | c.66.1.45 | S-adenosyl-L-methionine-dependent methyltransferases | S-adenosyl-L-methionine-dependent methyltransferases | N-6 DNA Methylase-like | Alpha and beta proteins (a/b) | [GO:0006306 "DNA methylation" evidence=ISS] [GO:0009007  "site-specific DNA-methyltransferase (adenine-specific) activity"  evidence=ISS] |
| HP1355 | c.1.17.1 | TIM beta/alpha-barrel | Nicotinate/Quinolinate PRTase C-terminal domain-like | NadC C-terminal domain-like | Alpha and beta proteins (a/b) | [GO:0004514 "nicotinate-nucleotide diphosphorylase (carboxylating)  activity" evidence=ISS] [GO:0009435 "NAD biosynthetic process"  evidence=ISS] |
| HP1356 | c.145.1.1 | NadA-like | NadA-like | NadA-like | Alpha and beta proteins (a/b) | [GO:0008987  "quinolinate synthetase A activity" evidence=ISS] [GO:0019363  "pyridine nucleotide biosynthetic process" evidence=ISS] |
| HP1357 | d.20.1.1 | UBC-like | UBC-like | UBC-related | Alpha and beta proteins (a+b) | [GO:0004609  "phosphatidylserine decarboxylase activity" evidence=ISS]  [GO:0008654 "phospholipid biosynthetic process" evidence=ISS] |
| HP1358 | b.110.1.1 | Cloacin translocation domain | Cloacin translocation domain | Cloacin translocation domain | All beta proteins | [GO:0003674  "molecular_function" evidence=ND] [GO:0005575 "cellular_component"  evidence=ND] [GO:0008150 "biological_process" evidence=ND] |
| HP1359 | g.92.1.1 | T-antigen specific domain-like | T-antigen specific domain-like | T-antigen specific domain-like | Small proteins |  |
| HP1360 | a.215.1.1 | A middle domain of Talin 1 | A middle domain of Talin 1 | A middle domain of Talin 1 | All alpha proteins | [GO:0006744  "ubiquinone biosynthetic process" evidence=ISS] [GO:0008412  "4-hydroxybenzoate octaprenyltransferase activity" evidence=ISS] |
| HP1361 | c.45.1.1 | (Phosphotyrosine protein) phosphatases II | (Phosphotyrosine protein) phosphatases II | Dual specificity phosphatase-like | Alpha and beta proteins (a/b) | [GO:0003674 "molecular_function"  evidence=ND] [GO:0008150 "biological_process" evidence=ND] |
| HP1362 | c.37.1.11 | P-loop containing nucleoside triphosphate hydrolases | P-loop containing nucleoside triphosphate hydrolases | RecA protein-like (ATPase-domain) | Alpha and beta proteins (a/b) | [GO:0004003 "ATP-dependent DNA  helicase activity" evidence=ISS] [GO:0006260 "DNA replication"  evidence=ISS] |
| HP1363 | c.104.1.1 | YjeF N-terminal domain-like | YjeF N-terminal domain-like | YjeF N-terminal domain-like | Alpha and beta proteins (a/b) | [GO:0003674  "molecular_function" evidence=ND] [GO:0005575 "cellular_component"  evidence=ND] [GO:0008150 "biological_process" evidence=ND] |
| HP1364 | d.122.1.3 | ATPase domain of HSP90 chaperone/DNA topoisomerase II/histidine kinase | ATPase domain of HSP90 chaperone/DNA topoisomerase II/histidine kinase | Histidine kinase | Alpha and beta proteins (a+b) | [GO:0000155 "phosphorelay sensor  kinase activity" evidence=ISS] [GO:0000160 "phosphorelay signal  transduction system" evidence=ISS] |
| HP1365 | a.4.6.1 | DNA/RNA-binding 3-helical bundle | C-terminal effector domain of the bipartite response regulators | PhoB-like | All alpha proteins | [GO:0000156  "phosphorelay response regulator activity" evidence=ISS]  [GO:0000160 "phosphorelay signal transduction system" evidence=ISS]  [GO:0003677 "DNA binding" evidence=ISS] [GO:0006355 "regulation of  transcription, DNA-dependent" evidence=ISS] |
| HP1366 | d.4.1.1 | His-Me finger endonucleases | His-Me finger endonucleases | HNH-motif | Alpha and beta proteins (a+b) | [GO:0003674  "molecular_function" evidence=ND] [GO:0008150 "biological_process"  evidence=ND] |
| HP1367 | c.66.1.11 | S-adenosyl-L-methionine-dependent methyltransferases | S-adenosyl-L-methionine-dependent methyltransferases | Type II DNA methylase | Alpha and beta proteins (a/b) | [GO:0008170 "N-methyltransferase activity"  evidence=IEA] [GO:0003677 "DNA binding" evidence=IEA] [GO:0003676  "nucleic acid binding" evidence=IEA] [GO:0032775 "DNA methylation  on adenine" evidence=IEA;IDA] |
| HP1368 | c.66.1.11 | S-adenosyl-L-methionine-dependent methyltransferases | S-adenosyl-L-methionine-dependent methyltransferases | Type II DNA methylase | Alpha and beta proteins (a/b) | [GO:0006306 "DNA methylation"  evidence=ISS] [GO:0009008 "DNA-methyltransferase activity"  evidence=ISS] |
| HP1369 | c.66.1.11 | S-adenosyl-L-methionine-dependent methyltransferases | S-adenosyl-L-methionine-dependent methyltransferases | Type II DNA methylase | Alpha and beta proteins (a/b) | [GO:0006306 "DNA methylation" evidence=ISS] [GO:0009007  "site-specific DNA-methyltransferase (adenine-specific) activity"  evidence=ISS] [GO:0009307 "DNA restriction-modification system"  evidence=ISS] |
| HP1371 | c.37.1.19 | P-loop containing nucleoside triphosphate hydrolases | P-loop containing nucleoside triphosphate hydrolases | Tandem AAA-ATPase domain | Alpha and beta proteins (a/b) | [GO:0009307 "DNA  restriction-modification system" evidence=ISS] [GO:0015668 "Type  III site-specific deoxyribonuclease activity" evidence=ISS] |
| HP1372 | b.69.3.1 | bladed beta-propeller | Nitrous oxide reductase, N-terminal domain | Nitrous oxide reductase, N-terminal domain | All beta proteins | [GO:0003674  "molecular_function" evidence=ND] |
| HP1373 | c.55.1.1 | Ribonuclease H-like motif | Actin-like ATPase domain | Actin/HSP70 | Alpha and beta proteins (a/b) | [GO:0003674  "molecular_function" evidence=ND] |
| HP1374 | g.39.1.11 | Glucocorticoid receptor-like (DNA-binding domain) | Glucocorticoid receptor-like (DNA-binding domain) | ClpX chaperone zinc binding domain | Small proteins | [GO:0005524 "ATP binding" evidence=ISS] [GO:0009368 "endopeptidase  Clp complex" evidence=ISS] |
| HP1375 | b.81.1.1 | Single-stranded left-handed beta-helix | Trimeric LpxA-like enzymes | UDP N-acetylglucosamine acyltransferase | All beta proteins | [GO:0008780 "acyl-[acyl-carrier-protein]-UDP-N-acetylglucosamine  O-acyltransferase activity" evidence=ISS] [GO:0009245 "lipid A  biosynthetic process" evidence=ISS] |
| HP1376 | d.38.1.6 | Thioesterase/thiol ester dehydrase-isomerase | Thioesterase/thiol ester dehydrase-isomerase | FabZ-like | Alpha and beta proteins (a+b) | [GO:0006633 "fatty acid biosynthetic process" evidence=ISS]  [GO:0016836 "hydro-lyase activity" evidence=ISS] |
| HP1377 | b.158.1.1 | BH3618-like | BH3618-like | BH3618-like | All beta proteins | [GO:0003674 "molecular_function"  evidence=ND] [GO:0005575 "cellular_component" evidence=ND]  [GO:0008150 "biological_process" evidence=ND] |
| HP1378 | a.118.8.1 | alpha-alpha superhelix | TPR-like | Tetratricopeptide repeat (TPR) | All alpha proteins | [GO:0003674 "molecular_function"  evidence=ND] [GO:0008150 "biological_process" evidence=ND] |
| HP1379 | b.122.1.10 | PUA domain-like | PUA domain-like | LON domain-like | All beta proteins | [GO:0006508 "proteolysis"  evidence=ISS] |
| HP1380 | c.2.1.6 | NAD(P)-binding Rossmann-fold domains | NAD(P)-binding Rossmann-fold domains | phosphogluconate dehydrogenase-like, N-terminal domain | Alpha and beta proteins (a/b) | [GO:0008977 "prephenate dehydrogenase  activity" evidence=ISS] |
| HP1381 | b.67.2.4 | 5-bladed beta-propeller | Arabinanase/levansucrase/invertase | TM1225-like predicted glycosylases | All beta proteins |  |
| HP1382 | d.4.1.2 | His-Me finger endonucleases | His-Me finger endonucleases | DNA/RNA non-specific endonuclease | Alpha and beta proteins (a+b) | [GO:0004519  "endonuclease activity" evidence=ISS] [GO:0006308 "DNA catabolic  process" evidence=ISS] [GO:0006401 "RNA catabolic process"  evidence=ISS] |
| HP1383 | d.287.1.2 | DNA methylase specificity domain | DNA methylase specificity domain | Type I restriction modification DNA specificity domain | Alpha and beta proteins (a+b) |  |
| HP1384 | f.38.1.1 | MFS general substrate transporter | MFS general substrate transporter | Glycerol-3-phosphate transporter | Membrane and cell surface proteins and peptides | [GO:0003674  "molecular_function" evidence=ND] [GO:0005575 "cellular_component"  evidence=ND] [GO:0008150 "biological_process" evidence=ND] |
| HP1385 | e.7.1.1 | Carbohydrate phosphatase | Carbohydrate phosphatase | Inositol monophosphatase/fructose-1,6-bisphosphatase-like | Multi-domain proteins (alpha and beta) | [GO:0006094  "gluconeogenesis" evidence=ISS] [GO:0042132 "fructose  1,6-bisphosphate 1-phosphatase activity" evidence=ISS] |
| HP1386 | c.1.2.2 | TIM beta/alpha-barrel | Ribulose-phoshate binding barrel | D-ribulose-5-phosphate 3-epimerase | Alpha and beta proteins (a/b) | [GO:0004750  "ribulose-phosphate 3-epimerase activity" evidence=ISS] [GO:0006098  "pentose-phosphate shunt" evidence=ISS] |
| HP1387 | c.55.3.5 | Ribonuclease H-like motif | Ribonuclease H-like | DnaQ-like 3'-5' exonuclease | Alpha and beta proteins (a/b) | [GO:0004527 "exonuclease activity" evidence=ISS] [GO:0006260 "DNA  replication" evidence=ISS] [GO:0009360 "DNA polymerase III complex"  evidence=ISS] |
| HP1388 | f.24.1.1 | Cytochrome c oxidase subunit I-like | Cytochrome c oxidase subunit I-like | Cytochrome c oxidase subunit I-like | Membrane and cell surface proteins and peptides | [GO:0051093 "negative  regulation of developmental process" evidence=IMP] [GO:0016772  "transferase activity, transferring phosphorus-containing groups"  evidence=IEA] [GO:0006468 "protein phosphorylation" evidence=IEA]  [GO:0005524 "ATP binding" evidence=IEA] [GO:0004674 "protein  serine/threonine kinase activity" evidence=IEA] |
| HP1389 | d.153.1.4 | Ntn hydrolase-like | N-terminal nucleophile aminohydrolases (Ntn hydrolases) | Proteasome subunits | Alpha and beta proteins (a+b) |  |
| HP1390 | b.68.1.1 | 6-bladed beta-propeller | Sialidases | Sialidases (neuraminidases) | All beta proteins | [GO:0005524 "ATP binding" evidence=IEA] [GO:0004386 "helicase  activity" evidence=IEA] [GO:0003677 "DNA binding" evidence=IEA]  [GO:0003676 "nucleic acid binding" evidence=IEA] [GO:0006357  "regulation of transcription from RNA polymerase II promoter"  evidence=ISS] |
| HP1391 | b.1.5.1 | Immunoglobulin-like beta-sandwich | Transglutaminase, two C-terminal domains | Transglutaminase, two C-terminal domains | All beta proteins | [GO:0003674  "molecular_function" evidence=ND] [GO:0005575 "cellular_component"  evidence=ND] [GO:0008150 "biological_process" evidence=ND] |
| HP1392 | d.32.1.7 | Glyoxalase/Bleomycin resistance protein/Dihydroxybiphenyl dioxygenase | Glyoxalase/Bleomycin resistance protein/Dihydroxybiphenyl dioxygenase | 3-demethylubiquinone-9 3-methyltransferase | Alpha and beta proteins (a+b) | [GO:0008152  "metabolic process" evidence=ISS] |
| HP1393 | a.238.1.2 | BAR/IMD domain-like | BAR/IMD domain-like | Arfaptin, Rac-binding fragment | All alpha proteins | [GO:0005524 "ATP binding"  evidence=ISS] [GO:0006281 "DNA repair" evidence=ISS] [GO:0006310  "DNA recombination" evidence=ISS] |
| HP1394 | e.52.1.1 | NAD kinase/diacylglycerol kinase-like | NAD kinase/diacylglycerol kinase-like | NAD kinase-like | Multi-domain proteins (alpha and beta) | [GO:0003951 "NAD+ kinase activity"  evidence=ISS] [GO:0019363 "pyridine nucleotide biosynthetic  process" evidence=ISS] |
| HP1395 | f.4.3.2 | Transmembrane beta-barrels | Porins | Maltoporin-like | Membrane and cell surface proteins and peptides |  |
| HP1396 | d.4.1.6 | His-Me finger endonucleases | His-Me finger endonucleases | Endonuclease I | Alpha and beta proteins (a+b) | [GO:0003674  "molecular_function" evidence=ND] [GO:0008150 "biological_process"  evidence=ND] |
| HP1397 | a.7.12.1 | Spectrin repeat-like | PhoU-like | PhoU-like | All alpha proteins | [GO:0003674  "molecular_function" evidence=ND] [GO:0005575 "cellular_component"  evidence=ND] [GO:0008150 "biological_process" evidence=ND] |
| HP1398 | c.23.12.2 | Flavodoxin-like | Formate/glycerate dehydrogenase catalytic domain-like | L-alanine dehydrogenase-like | Alpha and beta proteins (a/b) | [GO:0000286 "alanine dehydrogenase  activity" evidence=ISS] [GO:0006522 "alanine metabolic process"  evidence=ISS] |
| HP1399 | c.42.1.1 | Arginase/deacetylase | Arginase/deacetylase | Arginase-like amidino hydrolases | Alpha and beta proteins (a/b) | [GO:0004053 "arginase activity"  evidence=IDA] [GO:0006525 "arginine metabolic process"  evidence=IDA] |
| HP1400 | f.4.3.3 | Transmembrane beta-barrels | Porins | Ligand-gated protein channel | Membrane and cell surface proteins and peptides | [GO:0044718 "siderophore  transmembrane transport" evidence=IEA] [GO:0015343 "siderophore  transmembrane transporter activity" evidence=IEA] [GO:0005506 "iron  ion binding" evidence=IEA] [GO:0015091 "ferric iron transmembrane  transporter activity" evidence=IMP] |
| HP01401 | [d.92.1.11](http://scop.berkeley.edu/search/?key="sccs:d.92.1.11") | [Zincin-like](http://scop.mrc-lmb.cam.ac.uk/scop/data/scop.b.e.cbc.html) | [Metalloproteases ("zincins"), catalytic domain](http://scop.mrc-lmb.cam.ac.uk/scop/data/scop.b.e.cbc.b.html) | [Matrix metalloproteases, catalytic domain](http://scop.mrc-lmb.cam.ac.uk/scop/data/scop.b.e.cbc.b.be.html) | Alpha and beta proteins (a+b) | [GO:0003674  "molecular_function" evidence=ND] [GO:0005575 "cellular_component"  evidence=ND] [GO:0008150 "biological_process" evidence=ND] |
| HP01402 | [c.37.1.19](http://scop.berkeley.edu/search/?key="sccs:c.37.1.19") | [P-loop containing nucleoside triphosphate hydrolases](http://scop.mrc-lmb.cam.ac.uk/scop/data/scop.b.d.fb.A.html) | [P-loop containing nucleoside triphosphate hydrolases](http://scop.mrc-lmb.cam.ac.uk/scop/data/scop.b.d.fb.A.html) | [Tandem AAA-ATPase domain](http://scop.mrc-lmb.cam.ac.uk/scop/data/scop.b.d.fb.b.bh.html) | Alpha and beta proteins (a/b) | [GO:0009035 "Type I site-specific deoxyribonuclease activity"  evidence=ISS] [GO:0009307 "DNA restriction-modification system"  evidence=ISS] [GO:0019812 "Type I site-specific deoxyribonuclease  complex" evidence=ISS] |
| HP01403 | [c.66.1.45](http://scop.berkeley.edu/search/?key="sccs:c.66.1.45") | [S-adenosyl-L-methionine-dependent methyltransferases](http://scop.mrc-lmb.cam.ac.uk/scop/data/scop.b.d.je.A.html) | [S-adenosyl-L-methionine-dependent methyltransferases](http://scop.mrc-lmb.cam.ac.uk/scop/data/scop.b.d.je.A.html) | [N-6 DNA Methylase-like](http://scop.mrc-lmb.cam.ac.uk/scop/data/scop.b.d.je.b.ed.html) | Alpha and beta proteins (a/b) | [GO:0006304 "DNA modification" evidence=ISS] [GO:0009007  "site-specific DNA-methyltransferase (adenine-specific) activity"  evidence=ISS] |
| HP01404 | [d.287.1.2](http://scop.berkeley.edu/search/?key="sccs:d.287.1.2") | [DNA methylase specificity domain](http://scop.mrc-lmb.cam.ac.uk/scop/data/scop.b.e.dfg.A.html) | [DNA methylase specificity domain](http://scop.mrc-lmb.cam.ac.uk/scop/data/scop.b.e.dfg.A.html) | [Type I restriction modification DNA specificity domain](http://scop.mrc-lmb.cam.ac.uk/scop/data/scop.b.e.dfg.b.c.html) | Alpha and beta proteins (a+b) | [GO:0009307 "DNA  restriction-modification system" evidence=ISS] [GO:0015666  "restriction endodeoxyribonuclease activity" evidence=ISS] |
| HP01405 | [h.1.5.1](http://scop.berkeley.edu/search/?key="sccs:h.1.5.1") | [Parallel coiled-coil](http://scop.mrc-lmb.cam.ac.uk/scop/data/scop.b.i.b.html) | [Tropomyosin](http://scop.mrc-lmb.cam.ac.uk/scop/data/scop.b.i.b.f.A.html) | [Tropomyosin](http://scop.mrc-lmb.cam.ac.uk/scop/data/scop.b.i.b.f.A.html) |  |  |
| HP01406 | [c.1.28.1](http://scop.berkeley.edu/search/?key="sccs:c.1.28.1") | [TIM beta/alpha-barrel](http://scop.mrc-lmb.cam.ac.uk/scop/data/scop.b.d.b.html) | [Radical SAM enzymes](http://scop.mrc-lmb.cam.ac.uk/scop/data/scop.b.d.b.ci.html) | [Biotin synthase](http://scop.mrc-lmb.cam.ac.uk/scop/data/scop.b.d.b.ci.b.A.html) | Alpha and beta proteins(a/b) | [GO:0004076 "biotin synthase  activity" evidence=ISS] [GO:0009102 "biotin biosynthetic process"  evidence=ISS] |
| HP01407 | [d.2.1.3](http://scop.berkeley.edu/search/?key="sccs:d.2.1.3") | [Lysozyme-like](http://scop.mrc-lmb.cam.ac.uk/scop/data/scop.b.e.c.A.html) | [Lysozyme-like](http://scop.mrc-lmb.cam.ac.uk/scop/data/scop.b.e.c.A.html) | [Phage lysozyme](http://scop.mrc-lmb.cam.ac.uk/scop/data/scop.b.e.c.b.d.html) | Alpha and beta proteins(a+b) | [GO:0003674 "molecular_function" evidence=ND]  [GO:0005575 "cellular_component" evidence=ND] [GO:0008150  "biological_process" evidence=ND] |
| HP01408 | [b.1.5.1](http://scop.berkeley.edu/search/?key="sccs:b.1.5.1") | [Immunoglobulin-like beta-sandwich](http://scop.mrc-lmb.cam.ac.uk/scop/data/scop.b.c.b.html) | [Transglutaminase, two C-terminal domains](http://scop.mrc-lmb.cam.ac.uk/scop/data/scop.b.c.b.g.b.html) | [Transglutaminase, two C-terminal domains](http://scop.mrc-lmb.cam.ac.uk/scop/data/scop.b.c.b.g.b.html) | All beta proteins | [GO:0003674  "molecular_function" evidence=ND] [GO:0008150 "biological_process"  evidence=ND] |
| HP01409 | [d.268.1.2](http://scop.berkeley.edu/search/?key="sccs:d.268.1.2") | [ParB/Sulfiredoxin](http://scop.mrc-lmb.cam.ac.uk/scop/data/scop.b.e.fc.A.html) | [ParB/Sulfiredoxin](http://scop.mrc-lmb.cam.ac.uk/scop/data/scop.b.e.fc.A.html) | [Hypothetical protein PF0380](http://scop.mrc-lmb.cam.ac.uk/scop/data/scop.b.e.fc.b.c.A.html) | Alpha and beta proteins(a+b) | [GO:0003674  "molecular_function" evidence=ND] [GO:0005575 "cellular_component"  evidence=ND] [GO:0008150 "biological_process" evidence=ND] |
| HP01410 | [c.107.1.2](http://scop.berkeley.edu/search/?key="sccs:c.107.1.2") | [DHH phosphoesterases](http://scop.mrc-lmb.cam.ac.uk/scop/data/scop.b.d.bdd.A.html) | [DHH phosphoesterases](http://scop.mrc-lmb.cam.ac.uk/scop/data/scop.b.d.bdd.A.html) | [Exonuclease RecJ](http://scop.mrc-lmb.cam.ac.uk/scop/data/scop.b.d.bdd.b.c.A.html) | Alpha and beta proteins(a/b) | [GO:0006310  "DNA recombination" evidence=ISS] [GO:0008297 "single-stranded DNA  specific exodeoxyribonuclease activity" evidence=ISS] |
| HP01411 | [h.4.4.1](http://scop.berkeley.edu/search/?key="sccs:h.4.4.1") | [Antiparallel coiled-coil](http://scop.mrc-lmb.cam.ac.uk/scop/data/scop.b.i.e.html) | [Bacterial hemolysins](http://scop.mrc-lmb.cam.ac.uk/scop/data/scop.b.i.e.f.html) | [Hemolysin E (HlyE, ClyA, SheA](http://scop.mrc-lmb.cam.ac.uk/scop/data/scop.b.i.e.f.b.A.html) |  | [GO:0005824 "outer plaque of spindle pole body" evidence=IDA]  [GO:0000070 "mitotic sister chromatid segregation" evidence=IMP]  [GO:0005200 "structural constituent of cytoskeleton" evidence=IPI]  [GO:0000022 "mitotic spindle elongation" evidence=IMP] |
| HP01412 | [b.47.1.1](http://scop.berkeley.edu/search/?key="sccs:b.47.1.1") | [Trypsin-like serine proteases](http://scop.mrc-lmb.cam.ac.uk/scop/data/scop.b.c.ia.A.html) | [Trypsin-like serine proteases](http://scop.mrc-lmb.cam.ac.uk/scop/data/scop.b.c.ia.A.html) | [Prokaryotic proteases](http://scop.mrc-lmb.cam.ac.uk/scop/data/scop.b.c.ia.b.b.html) | All beta proteins |  |
| HP01413 | [d.96.1.1](http://scop.berkeley.edu/search/?key="sccs:d.96.1.1") | [T-fold](http://scop.mrc-lmb.cam.ac.uk/scop/data/scop.b.e.cbj.html) | [Tetrahydrobiopterin biosynthesis enzymes-like](http://scop.mrc-lmb.cam.ac.uk/scop/data/scop.b.e.cbj.b.html) | [GTP cyclohydrolase](http://scop.mrc-lmb.cam.ac.uk/scop/data/scop.b.e.cbj.b.b.html) | Alpha and beta proteins(a+b) | [GO:0003674  "molecular_function" evidence=ND] [GO:0005575 "cellular_component"  evidence=ND] [GO:0008150 "biological_process" evidence=ND] |
| HP01414 | [d.218.1.12](http://scop.berkeley.edu/search/?key="sccs:d.218.1.12") | [Nucleotidyltransferase](http://scop.mrc-lmb.cam.ac.uk/scop/data/scop.b.e.bbg.A.html) | [Nucleotidyltransferase](http://scop.mrc-lmb.cam.ac.uk/scop/data/scop.b.e.bbg.A.html) | [Iojap/YbeB-like](http://scop.mrc-lmb.cam.ac.uk/scop/data/scop.b.e.bbg.b.bc.html) | Alpha and beta proteins(a+b) | [GO:0003674  "molecular_function" evidence=ND] [GO:0005575 "cellular_component"  evidence=ND] [GO:0008150 "biological_process" evidence=ND] |
| HP01415 | [f.19.1.1](http://scop.berkeley.edu/search/?key="sccs:f.19.1.1") | [Aquaporin-like](http://scop.mrc-lmb.cam.ac.uk/scop/data/scop.b.g.bb.A.A.html) | [Aquaporin-like](http://scop.mrc-lmb.cam.ac.uk/scop/data/scop.b.g.bb.A.A.html) | [Aquaporin-like](http://scop.mrc-lmb.cam.ac.uk/scop/data/scop.b.g.bb.A.A.html) | Membrane and cell surface proteins and peptides | [GO:0006400 "tRNA modification" evidence=ISS] |
| HP01416 | [c.68.1.4](http://scop.berkeley.edu/search/?key="sccs:c.68.1.4") | [Nucleotide-diphospho-sugar transferases](http://scop.mrc-lmb.cam.ac.uk/scop/data/scop.b.d.jh.A.html) | [Nucleotide-diphospho-sugar transferases](http://scop.mrc-lmb.cam.ac.uk/scop/data/scop.b.d.jh.A.html) | [Galactosyltransferase LgtC](http://scop.mrc-lmb.cam.ac.uk/scop/data/scop.b.d.jh.b.d.A.html) | Alpha and beta proteins (a/b) | [GO:0016757 "transferase  activity, transferring glycosyl groups" evidence=IEA] [GO:0016020  "membrane" evidence=IEA] [GO:0016740 "transferase activity"  evidence=IEA] [GO:0016021 "integral to membrane" evidence=IEA] |
| HP01417 | [c.76.1.2](http://scop.berkeley.edu/search/?key="sccs:c.76.1.2") | [Alkaline phosphatase-like](http://scop.mrc-lmb.cam.ac.uk/scop/data/scop.b.d.bah.A.html) | [Alkaline phosphatase-like](http://scop.mrc-lmb.cam.ac.uk/scop/data/scop.b.d.bah.A.html) | [Arylsulfatase](http://scop.mrc-lmb.cam.ac.uk/scop/data/scop.b.d.bah.b.d.html) | Alpha and beta proteins (a/b) |  |
| HP01418 | [d.145.1.2](http://scop.berkeley.edu/search/?key="sccs:d.145.1.2") | [FAD-binding/transporter-associated domain-like](http://scop.mrc-lmb.cam.ac.uk/scop/data/scop.b.e.dab.A.html) | [FAD-binding/transporter-associated domain-like](http://scop.mrc-lmb.cam.ac.uk/scop/data/scop.b.e.dab.A.html) | [Uridine diphospho-N-Acetylenolpyruvylglucosamine reductase (MurB), N-terminal domain](http://scop.mrc-lmb.cam.ac.uk/scop/data/scop.b.e.dab.b.c.html) | Alpha and beta proteins (a+b) | [GO:0008762  "UDP-N-acetylmuramate dehydrogenase activity" evidence=ISS]  [GO:0009252 "peptidoglycan biosynthetic process" evidence=ISS] |
| HP01419 | [c.72.2.2](http://scop.berkeley.edu/search/?key="sccs:c.72.2.2") | [Ribokinase-like](http://scop.mrc-lmb.cam.ac.uk/scop/data/scop.b.d.bad.html) | [MurD-like peptide ligases, catalytic domain](http://scop.mrc-lmb.cam.ac.uk/scop/data/scop.b.d.bad.c.html) | [Folylpolyglutamate synthetase](http://scop.mrc-lmb.cam.ac.uk/scop/data/scop.b.d.bad.c.c.html) | Alpha and beta proteins (a/b) | [GO:0003674  "molecular_function" evidence=ND] |
| HP01420 | [i.3.1.1](http://scop.berkeley.edu/search/?key="sccs:i.3.1.1") | [ATP synthase](http://scop.mrc-lmb.cam.ac.uk/scop/data/scop.b.j.f.A.A.html) | [ATP synthase](http://scop.mrc-lmb.cam.ac.uk/scop/data/scop.b.j.f.A.A.html) | [ATP synthase](http://scop.mrc-lmb.cam.ac.uk/scop/data/scop.b.j.f.A.A.html) |  | [GO:0005515 "protein binding"  evidence=IPI] |
| HP01421 | [c.37.1.11](http://scop.berkeley.edu/search/?key="sccs:c.37.1.11") | [P-loop containing nucleoside triphosphate hydrolases](http://scop.mrc-lmb.cam.ac.uk/scop/data/scop.b.d.fb.A.html) | [P-loop containing nucleoside triphosphate hydrolases](http://scop.mrc-lmb.cam.ac.uk/scop/data/scop.b.d.fb.A.html) | [RecA protein-like (ATPase-domain](http://scop.mrc-lmb.cam.ac.uk/scop/data/scop.b.d.fb.b.be.html) | Alpha and beta proteins (a/b) | [GO:0008150  "biological_process" evidence=ND] |
| HP01422 | [a.27.1.1](http://scop.berkeley.edu/search/?key="sccs:a.27.1.1") | [Anticodon-binding domain of a subclass of class I aminoacyl-tRNA synthetases](http://scop.mrc-lmb.cam.ac.uk/scop/data/scop.b.b.ea.A.A.html) | [Anticodon-binding domain of a subclass of class I aminoacyl-tRNA synthetases](http://scop.mrc-lmb.cam.ac.uk/scop/data/scop.b.b.ea.A.A.html) | [Anticodon-binding domain of a subclass of class I aminoacyl-tRNA synthetases](http://scop.mrc-lmb.cam.ac.uk/scop/data/scop.b.b.ea.A.A.html) | All alpha proteins | [GO:0004822 "isoleucine-tRNA ligase  activity" evidence=ISS] [GO:0006428 "isoleucyl-tRNA aminoacylation"  evidence=ISS] |
| HP01423 | [d.66.1.3](http://scop.berkeley.edu/search/?key="sccs:d.66.1.3") | [Alpha-L RNA-binding motif](http://scop.mrc-lmb.cam.ac.uk/scop/data/scop.b.e.bfh.A.html) | [Alpha-L RNA-binding motif](http://scop.mrc-lmb.cam.ac.uk/scop/data/scop.b.e.bfh.A.html) | [Heat shock protein 15 kD](http://scop.mrc-lmb.cam.ac.uk/scop/data/scop.b.e.bfh.b.c.A.html) | Alpha and beta proteins (a+b) | [GO:0008150 "biological_process"  evidence=ND] |
| HP01424 | [b.51.1.1](http://scop.berkeley.edu/search/?key="sccs:b.51.1.1") | [ValRS/IleRS/LeuRS editing domain](http://scop.mrc-lmb.cam.ac.uk/scop/data/scop.b.c.ig.A.A.html) | [ValRS/IleRS/LeuRS editing domain](http://scop.mrc-lmb.cam.ac.uk/scop/data/scop.b.c.ig.A.A.html) | [ValRS/IleRS/LeuRS editing domain](http://scop.mrc-lmb.cam.ac.uk/scop/data/scop.b.c.ig.A.A.html) | All beta proteins |  |
| HP01425 | [c.2.1.1](http://scop.berkeley.edu/search/?key="sccs:c.2.1.1") | [NAD(P)-binding Rossmann-fold domains](http://scop.mrc-lmb.cam.ac.uk/scop/data/scop.b.d.c.A.html) | [NAD(P)-binding Rossmann-fold domains](http://scop.mrc-lmb.cam.ac.uk/scop/data/scop.b.d.c.A.html) | [Alcohol dehydrogenase-like, C-terminal domain](http://scop.mrc-lmb.cam.ac.uk/scop/data/scop.b.d.c.b.b.html) | Alpha and beta proteins (a/b) |  |
| HP01426 | [a.4.1.3](http://scop.berkeley.edu/search/?key="sccs:a.4.1.3") | [DNA/RNA-binding 3-helical bundle](http://scop.mrc-lmb.cam.ac.uk/scop/data/scop.b.b.j.html) | [Homeodomain-like](http://scop.mrc-lmb.cam.ac.uk/scop/data/scop.b.b.j.b.html) | [Myb/SANT domain](http://scop.mrc-lmb.cam.ac.uk/scop/data/scop.b.b.j.b.d.html) | All alpha proteins |  |
| HP01427 | [f.4.4.1](http://scop.berkeley.edu/search/?key="sccs:f.4.4.1") | [Transmembrane beta-barrels](http://scop.mrc-lmb.cam.ac.uk/scop/data/scop.b.g.eh.html) | [OMPT-like](http://scop.mrc-lmb.cam.ac.uk/scop/data/scop.b.g.eh.c.html) | [Outer membrane protease OMPT](http://scop.mrc-lmb.cam.ac.uk/scop/data/scop.b.g.eh.c.b.A.html) | Membrane and cell surface proteins and peptides |  |
| HP01428 | [c.1.28.1](http://scop.berkeley.edu/search/?key="sccs:c.1.28.1") | [TIM beta/alpha-barrel](http://scop.mrc-lmb.cam.ac.uk/scop/data/scop.b.d.b.html) | [Radical SAM enzymes](http://scop.mrc-lmb.cam.ac.uk/scop/data/scop.b.d.b.ci.html) | [Biotin synthase](http://scop.mrc-lmb.cam.ac.uk/scop/data/scop.b.d.b.ci.b.A.html) | Alpha and beta proteins (a/b) | [GO:0003824 "catalytic  activity" evidence=ISS] [GO:0008152 "metabolic process"  evidence=ISS] |
| HP01429 | [c.80.1.3](http://scop.berkeley.edu/search/?key="sccs:c.80.1.3") | [SIS domain](http://scop.mrc-lmb.cam.ac.uk/scop/data/scop.b.d.bbi.b.html) | [SIS domain](http://scop.mrc-lmb.cam.ac.uk/scop/data/scop.b.d.bbi.b.html) | [mono-SIS domain](http://scop.mrc-lmb.cam.ac.uk/scop/data/scop.b.d.bbi.b.b.html) | Alpha and beta proteins (a/b) | [GO:0003674 "molecular_function" evidence=ND] [GO:0005575  "cellular_component" evidence=ND] [GO:0008150 "biological_process"  evidence=ND] |
| HP01430 | [d.157.1.13](http://scop.berkeley.edu/search/?key="sccs:d.157.1.13") | [Metallo-hydrolase/oxidoreductase](http://scop.mrc-lmb.cam.ac.uk/scop/data/scop.b.e.dbe.A.html) | [Metallo-hydrolase/oxidoreductase](http://scop.mrc-lmb.cam.ac.uk/scop/data/scop.b.e.dbe.A.html) | [Alkylsulfatase-like](http://scop.mrc-lmb.cam.ac.uk/scop/data/scop.b.e.dbe.b.bd.A.html)[beta-CASP RNA metabolising hydrolases](http://scop.mrc-lmb.cam.ac.uk/scop/data/scop.b.e.dbe.b.ba.html) | Alpha and beta proteins (a+b) | [GO:0003824 "catalytic  activity" evidence=ISS] [GO:0008152 "metabolic process"  evidence=ISS] |
| HP01431 | c.66.1.24 | S-adenosyl-L-methionine-dependent methyltransferases | S-adenosyl-L-methionine-dependent methyltransferases | rRNA adenine dimethylase-like | Alpha and beta proteins (a/b) | [GO:0000154 "rRNA  modification" evidence=ISS] [GO:0000179 "rRNA  (adenine-N6,N6-)-dimethyltransferase activity" evidence=ISS]  [GO:0046677 "response to antibiotic" evidence=ISS] |
| HP01432 | d.290.1.2 | AF0104/ALDC/Ptd012-like | AF0104/ALDC/Ptd012-like | PTD012-like | Alpha and beta proteins (a+b) |  |
| HP01433 | c.66.1.45 | S-adenosyl-L-methionine-dependent methyltransferases | S-adenosyl-L-methionine-dependent methyltransferases | N-6 DNA Methylase-like | Alpha and beta proteins (a/b) |  |
| HP01434 | c.65.1.1 | Formyltransferase | Formyltransferase | Formyltransferase | Alpha and beta proteins (a/b) | [GO:0008864  "formyltetrahydrofolate deformylase activity" evidence=ISS]  [GO:0009152 "purine ribonucleotide biosynthetic process"  evidence=ISS] |
| HP01435 |  |  |  |  |  | [GO:0006465 "signal  peptide processing" evidence=ISS] |
| HP01436 | c.87.1.4 | UDP-Glycosyltransferase/glycogen phosphorylase | UDP-Glycosyltransferase/glycogen phosphorylase | Oligosaccharide phosphorylase | Alpha and beta proteins (a/b) | [GO:0003674 "molecular_function" evidence=ND] [GO:0005575  "cellular_component" evidence=ND] [GO:0008150 "biological_process"  evidence=ND] |
| HP01437 | c.1.10.1 | TIM beta/alpha-barrel | Aldolase | Class I aldolase | Alpha and beta proteins (a/b) | [GO:0007165  "signal transduction" evidence=IEA] [GO:0046872 "metal ion binding"  evidence=IEA] [GO:0047555 "3',5'-cyclic-GMP phosphodiesterase  activity" evidence=IEA] [GO:0006198 "cAMP catabolic process"  evidence=IEA] |
| HP01438 | b.110.1.1 | Cloacin translocation domain | Cloacin translocation domain | Cloacin translocation domain | All beta proteins | [GO:0003674  "molecular_function" evidence=ND] [GO:0060187 "cell pole"  evidence=IDA] [GO:0031941 "filamentous actin" evidence=IDA]  [GO:0071944 "cell periphery" evidence=IDA] |
| HP01439 | b.37.1.1 | N-terminal domains of the minor coat protein g3p | N-terminal domains of the minor coat protein g3p | N-terminal domains of the minor coat protein g3p | All beta proteins | [GO:0004106 "chorismate  mutase activity" evidence=ISS] [GO:0006571 "tyrosine biosynthetic  process" evidence=ISS] [GO:0008977 "prephenate dehydrogenase  activity" evidence=ISS] |
| HP01440 | b.47.1.1 | Trypsin-like serine proteases | Trypsin-like serine proteases | Prokaryotic proteases | All beta proteins |  |
| HP01441 | b.62.1.1 | Cyclophilin-like | Cyclophilin-like | Cyclophilin (peptidylprolyl isomerase) | All beta proteins | [GO:0003755  "peptidyl-prolyl cis-trans isomerase activity" evidence=ISS]  [GO:0006457 "protein folding" evidence=ISS] |
| HP01442 | b.50.1.3 | Acid proteases | Acid proteases | LPG0085-like | All beta proteins | [GO:0003723 "RNA binding"  evidence=ISS] [GO:0006109 "regulation of carbohydrate metabolic  process" evidence=ISS] |
| HP01443 | d.14.1.5 | Ribosomal protein S5 domain 2-like | Ribosomal protein S5 domain 2-like | GHMP Kinase, N-terminal domain | Alpha and beta proteins (a+b) | [GO:0019288  "isopentenyl diphosphate biosynthetic process,  mevalonate-independent pathway" evidence=ISS] [GO:0050515  "4-(cytidine 5'-diphospho)-2-C-methyl-D-erythritol kinase activity"  evidence=ISS] |
| HP01444 | b.111.1.1 | Small protein B (SmpB) | Small protein B (SmpB) | Small protein B (SmpB) | All beta proteins | [GO:0003723 "RNA binding"  evidence=ISS] [GO:0006450 "regulation of translational fidelity"  evidence=ISS] |
| HP01446 | c.81.1.1 | Formate dehydrogenase/DMSO reductase, domains 1-3 | Formate dehydrogenase/DMSO reductase, domains 1-3 | Formate dehydrogenase/DMSO reductase, domains 1-3 | Alpha and beta proteins (a/b) | [GO:0005215  "transporter activity" evidence=ISS] [GO:0006810 "transport"  evidence=ISS] |
| HP01447 | b.34.5.3 | SH3-like barrel | Translation proteins SH3-like domain | C-terminal domain of ribosomal protein L2 | All beta proteins | [GO:0003735 "structural constituent  of ribosome" evidence=ISS] [GO:0042254 "ribosome biogenesis"  evidence=ISS] |
| HP01448 | d.14.1.2 | Ribosomal protein S5 domain 2-like | Ribosomal protein S5 domain 2-like | RNase P protein | Alpha and beta proteins (a+b) | [GO:0004526  "ribonuclease P activity" evidence=ISS] [GO:0005655 "nucleolar  ribonuclease P complex" evidence=ISS] [GO:0006396 "RNA processing"  evidence=ISS] |
| HP01449 | c.142.1.1 | Nqo1 FMN-binding domain-like | Nqo1 FMN-binding domain-like | Nqo1 FMN-binding domain-like | Alpha and beta proteins (a/b) | [GO:0003674 "molecular_function" evidence=ND] [GO:0005575  "cellular_component" evidence=ND] [GO:0008150 "biological_process"  evidence=ND] |
| HP01450 | b.69.6.1 | 7-bladed beta-propeller | Clathrin heavy-chain terminal domain | Clathrin heavy-chain terminal domain | All beta proteins | [GO:0003674  "molecular_function" evidence=ND] [GO:0008150 "biological_process"  evidence=ND] |
| HP01451 | d.58.32.1 | Ferredoxin-like | FAD-linked oxidases, C-terminal domain | Vanillyl-alcohol oxidase-like | Alpha and beta proteins (a+b) | [GO:0005515 "protein binding"  evidence=IPI] |
| HP01452 | c.37.1.8 | P-loop containing nucleoside triphosphate hydrolases | P-loop containing nucleoside triphosphate hydrolases | G proteins | Alpha and beta proteins (a/b) | [GO:0003924 "GTPase  activity" evidence=ISS] [GO:0006400 "tRNA modification"  evidence=ISS] |
| HP01453 | f.4.1.1 | TRANSMEMBRANE BETA BAREELS | OMPA LIKE | OUTERMEMBRANE PROTEINS | Membrane and cell surface proteins and peptides | [GO:0016772 "transferase  activity, transferring phosphorus-containing groups" evidence=IEA]  [GO:0006468 "protein phosphorylation" evidence=IEA] [GO:0005524  "ATP binding" evidence=IEA] [GO:0004674 "protein serine/threonine  kinase activity" evidence=IEA] |
| HP01454 | c.82.1.1 | ALDH-like | ALDH-like | ALDH-like | Alpha and beta proteins (a/b) |  |
| HP01455 | a.3.1.1 | Cytochrome c | Cytochrome c | monodomain cytochrome c | All alpha proteins |  |
| HP01456 | c.82.1.1 | ALDH-like | ALDH-like | ALDH-like | Alpha and beta proteins (a/b) |  |
| HP01457 | a.118.8.1 | alpha-alpha superhelix | TPR-like | Tetratricopeptide repeat (TPR) | All alpha proteins | [GO:0003674 "molecular_function"  evidence=ND] [GO:0008150 "biological_process" evidence=ND] |
| HP01458 | c.47.1.1 | Thioredoxin fold | Thioredoxin-like | Thioltransferase | Alpha and beta proteins (a/b) | [GO:0006662 "glycerol ether  metabolic process" evidence=IEA] [GO:0009055 "electron carrier  activity" evidence=IEA] [GO:0015035 "protein disulfide  oxidoreductase activity" evidence=IEA] [GO:0045454 "cell redox  homeostasis" evidence=IEA] |
| HP01459 | d.265.1.3 | Pseudouridine synthase | Pseudouridine synthase | Pseudouridine synthase RsuA/RluD | Alpha and beta proteins (a+b) | [GO:0009451  "RNA modification" evidence=ISS] [GO:0009982 "pseudouridine  synthase activity" evidence=ISS] |
| HP01460 | c.92.2.3 | Chelatase-like | Helical backbone metal receptor | Nitrogenase iron-molybdenum protein | Alpha and beta proteins (a/b) | [GO:0003887  "DNA-directed DNA polymerase activity" evidence=ISS] [GO:0006260  "DNA replication" evidence=ISS] [GO:0009360 "DNA polymerase III  complex" evidence=ISS] |
| HP01461 | a.3.1.5 | Cytochrome c | Cytochrome c | Di-heme cytochrome c peroxidase | All alpha proteins | [GO:0004130  "cytochrome-c peroxidase activity" evidence=ISS] |
| HP01462 | d.153.1.3 | Ntn hydrolase-like | N-terminal nucleophile aminohydrolases (Ntn hydrolases) | Penicillin V acylase | Alpha and beta proteins (a+b) | [GO:0003674  "molecular_function" evidence=ND] [GO:0005575 "cellular_component"  evidence=ND] [GO:0008150 "biological_process" evidence=ND] |
| HP01463 | c.51.6.2 | Anticodon-binding domain-like | XCC0632-like | XCC0632-like | Alpha and beta proteins (a/b) |  |
| HP01464 | a.238.1.4 | BAR/IMD domain-like | BAR/IMD domain-like | FCH domain | All alpha proteins | [GO:0006810 "transport" evidence=ISS] [GO:0042626  "ATPase activity, coupled to transmembrane movement of substances"  evidence=ISS] |
| HP01465 | c.37.1.12 | P-loop containing nucleoside triphosphate hydrolases | P-loop containing nucleoside triphosphate hydrolases | ABC transporter ATPase domain-like | Alpha and beta proteins (a/b) | [GO:0005524 "ATP  binding" evidence=ISS] [GO:0006810 "transport" evidence=ISS]  [GO:0042626 "ATPase activity, coupled to transmembrane movement of  substances" evidence=ISS] |
| HP01466 | f.20.1.1 | Clc chloride channel | Clc chloride channel | Clc chloride channel | Membrane and cell surface proteins and peptides | [GO:0006810  "transport" evidence=ISS] [GO:0042626 "ATPase activity, coupled to  transmembrane movement of substances" evidence=ISS] |
| HP01467 | f.4.1.1 | Transmembrane beta-barrels | OMPA-like | Outer membrane protein | Membrane and cell surface proteins and peptides |  |
| HP01468 | e.17.1.1 | D-aminoacid aminotransferase-like PLP-dependent enzymes | D-aminoacid aminotransferase-like PLP-dependent enzymes | D-aminoacid aminotransferase-like PLP-dependent enzymes | Multi-domain proteins (alpha and beta) | [GO:0003824 "catalytic  activity" evidence=IEA;ISS] [GO:0004084 "branched-chain-amino-acid  transaminase activity" evidence=IEA;IGI] [GO:0008152 "metabolic  process" evidence=IEA] [GO:0009081 "branched-chain amino acid  metabolic process" evidence=IEA] |
| HP01469 | f.4.1.1 | TRANSMEMBRANE BETA BAREELS | OMPA LIKE | OUTERMEMBRANE PROTEINS | Membrane and cell surface proteins and peptides | [GO:0000064 "L-ornithine  transmembrane transporter activity" evidence=IEA] [GO:0002537  "nitric oxide production involved in inflammatory response"  evidence=IEA] [GO:0005289 "high affinity arginine transmembrane  transporter activity" evidence=IEA] |
| HP01470 | e.8.1.1 | DNA/RNA polymerases | DNA/RNA polymerases | DNA polymerase I | Multi-domain proteins (alpha and beta) | [GO:0003887 "DNA-directed DNA  polymerase activity" evidence=ISS] [GO:0006260 "DNA replication"  evidence=ISS] [GO:0009360 "DNA polymerase III complex"  evidence=ISS] |
| HP01471 | d.287.1.2 | DNA methylase specificity domain | DNA methylase specificity domain | Type I restriction modification DNA specificity domain | Alpha and beta proteins (a+b) | [GO:0003674  "molecular_function" evidence=ND] |
| HP01472 | c.66.1.45 | S-adenosyl-L-methionine-dependent methyltransferases | S-adenosyl-L-methionine-dependent methyltransferases | N-6 DNA Methylase-like | Alpha and beta proteins (a/b) | [GO:0006304  "DNA modification" evidence=ISS] [GO:0006306 "DNA methylation"  evidence=ISS] [GO:0009007 "site-specific DNA-methyltransferase  (adenine-specific) activity" evidence=ISS] |
| HP01473 | c.61.1.2 | PRTase-like | PRTase-like | Phosphoribosylpyrophosphate synthetase-like | Alpha and beta proteins (a/b) | [GO:0003674 "molecular_function"  evidence=ND] [GO:0009294 "DNA mediated transformation"  evidence=IGI] |
| HP01474 | c.37.1.1 | P-loop containing nucleoside triphosphate hydrolases | P-loop containing nucleoside triphosphate hydrolases | Nucleotide and nucleoside kinases | Alpha and beta proteins (a/b) | [GO:0004798 "thymidylate kinase  activity" evidence=ISS] [GO:0015949 "nucleobase-containing small  molecule interconversion" evidence=ISS] |
| HP01475 | c.26.1.3 | Adenine nucleotide alpha hydrolase-like | Nucleotidylyl transferase | Adenylyltransferase | Alpha and beta proteins (a/b) | [GO:0004595  "pantetheine-phosphate adenylyltransferase activity" evidence=ISS]  [GO:0015937 "coenzyme A biosynthetic process" evidence=ISS] |
| HP01476 | c.34.1.1 | Homo-oligomeric flavin-containing Cys decarboxylases, HFCD | Homo-oligomeric flavin-containing Cys decarboxylases, HFCD | Homo-oligomeric flavin-containing Cys decarboxylases, HFCD | Alpha and beta proteins (a/b) | [GO:0006744 "ubiquinone biosynthetic process" evidence=ISS]  [GO:0008694 "3-octaprenyl-4-hydroxybenzoate carboxy-lyase activity"  evidence=ISS] |
| HP01477 | b.85.1.1 | beta-clip | AFP III-like domain | AFP III-like domain | All beta proteins | [GO:0001539 "ciliary or flagellar motility" evidence=ISS]  [GO:0005198 "structural molecule activity" evidence=ISS]  [GO:0009428 "bacterial-type flagellum basal body, distal rod, P  ring" evidence=ISS] |
| HP01478 | c.37.1.19 | P-loop containing nucleoside triphosphate hydrolases | P-loop containing nucleoside triphosphate hydrolases | Tandem AAA-ATPase domain | Alpha and beta proteins (a/b) | [GO:0004003  "ATP-dependent DNA helicase activity" evidence=ISS] [GO:0006281  "DNA repair" evidence=ISS] |
| HP01479 | a.118.8.1 | alpha-alpha superhelix | TPR-like | Tetratricopeptide repeat (TPR) | All alpha proteins | [GO:0003674 "molecular_function"  evidence=ND] [GO:0008150 "biological_process" evidence=ND] |
| HP01480 | d.104.1.1 | Class II aaRS and biotin synthetases | Class II aaRS and biotin synthetases | Class II aminoacyl-tRNA synthetase (aaRS)-like, catalytic domain | Alpha and beta proteins (a+b) | [GO:0004828 "serine-tRNA ligase  activity" evidence=ISS] [GO:0006434 "seryl-tRNA aminoacylation"  evidence=ISS] |
| HP01481 | d.160.1.2 | Carbon-nitrogen hydrolase | Carbon-nitrogen hydrolase | Carbamilase | Alpha and beta proteins (a+b) | [GO:0008152 "metabolic  process" evidence=ISS] [GO:0016810 "hydrolase activity, acting on  carbon-nitrogen (but not peptide) bonds" evidence=ISS] |
| HP01482 | a.7.13.1 | Spectrin repeat-like | XseB-like | XseB-like | All alpha proteins | " [GO:0006308  "DNA catabolic process" evidence=ISS] [GO:0008855  "exodeoxyribonuclease VII activity" evidence=ISS] [GO:0009318  "exodeoxyribonuclease VII complex" evidence=ISS] |
| HP01483 | c.66.1.18 | S-adenosyl-L-methionine-dependent methyltransferases | S-adenosyl-L-methionine-dependent methyltransferases | Mycolic acid cyclopropane synthase | Alpha and beta proteins (a/b) | [GO:0006744 "ubiquinone biosynthetic process" evidence=ISS]  [GO:0008168 "methyltransferase activity" evidence=ISS] |
| HP01484 | b.82.2.11 | Double-stranded beta-helix | Clavaminate synthase-like | Asparaginyl hydroxylase-like | All beta proteins | [GO:0003674  "molecular_function" evidence=ND] [GO:0005575 "cellular_component"  evidence=ND] [GO:0008150 "biological_process" evidence=ND] |
| HP01485 | d.14.1.11 | Ribosomal protein S5 domain 2-like | Ribosomal protein S5 domain 2-like | YigZ N-terminal domain-like | Alpha and beta proteins (a+b) | [GO:0003674  "molecular_function" evidence=ND] [GO:0005575 "cellular_component"  evidence=ND] [GO:0008150 "biological_process" evidence=ND] |
| HP01486 | c.1.21.1 | TIM beta/alpha-barrel | Dihydropteroate synthetase-like | Dihydropteroate synthetase-like | Alpha and beta proteins (a/b) | [GO:0003674  "molecular_function" evidence=ND] [GO:0005575 "cellular_component"  evidence=ND] [GO:0008150 "biological_process" evidence=ND] |
| HP01487 | c.1.21.1 | TIM beta/alpha-barrel | Dihydropteroate synthetase-like | Dihydropteroate synthetase-like | Alpha and beta proteins (a/b) | [GO:0003674 "molecular_function" evidence=ND] [GO:0005575  "cellular_component" evidence=ND] [GO:0008150 "biological_process"  evidence=ND] |
| HP01488 | f.46.1.1 | HlyD-like secretion proteins | HlyD-like secretion proteins | HlyD-like secretion proteins | Membrane and cell surface proteins and peptides | [GO:0003674 "molecular_function" evidence=ND] [GO:0005575  "cellular_component" evidence=ND] [GO:0008150 "biological_process"  evidence=ND] |
| HP01489 | f.5.1.1 | Outer membrane efflux proteins (OEP) | Outer membrane efflux proteins (OEP) | Outer membrane efflux proteins (OEP) | Membrane and cell surface proteins and peptides | [GO:0003674 "molecular_function" evidence=ND] [GO:0005575  "cellular_component" evidence=ND] [GO:0008150 "biological_process"  evidence=ND] |
| HP01490 | d.37.1.1 | CBS-domain pair | CBS-domain pair | CBS-domain pair | Alpha and beta proteins (a+b) | [GO:0005215 "transporter activity"  evidence=ISS] [GO:0006810 "transport" evidence=ISS] |
| HP01491 |  |  |  |  |  | [GO:0006817 "phosphate ion  transport" evidence=ISS] [GO:0015114 "phosphate ion transmembrane  transporter activity" evidence=ISS] |
| HP01492 | [d.52.8.1](http://scop.berkeley.edu/search/?key="sccs:d.52.8.1") | [Alpha-lytic protease prodomain-like](http://scop.berkeley.edu/sunid=54805) | [Fe-S cluster assembly (FSCA) domain-like](http://scop.berkeley.edu/sunid=117916) | [NifU C-terminal domain-like](http://scop.berkeley.edu/sunid=117917) | Alpha and beta proteins (a+b) | [GO:0003674 "molecular_function"  evidence=ND] [GO:0008150 "biological_process" evidence=ND] |
| HP01493 | [a.118.8.1](http://scop.berkeley.edu/search/?key="sccs:a.118.8.1") | [alpha-alpha superhelix](http://scop.berkeley.edu/sunid=48370) | [TPR-like](http://scop.berkeley.edu/sunid=48452) | [Tetratricopeptide repeat (TPR)](http://scop.berkeley.edu/sunid=48453) | All alpha proteins | [GO:0003674  "molecular_function" evidence=ND] [GO:0005575 "cellular_component"  evidence=ND] [GO:0008150 "biological_process" evidence=ND] |
| HP01494 | c.72.2.1 | Ribokinase-like | MurD-like peptide ligases, catalytic domain | MurCDEF | Alpha and beta proteins (a/b) | [GO:0008765  "UDP-N-acetylmuramoylalanyl-D-glutamate-2,6-diaminopimelate ligase  activity" evidence=ISS] [GO:0009252 "peptidoglycan biosynthetic  process" evidence=ISS] |
| HP01495 | c.1.10.1 | TIM beta/alpha-barrel | Aldolase | Class I aldolase | Alpha and beta proteins (a/b) | [GO:0004801  "sedoheptulose-7-phosphate:D-glyceraldehyde-3-phosphate  glyceronetransferase activity" evidence=ISS] [GO:0006098  "pentose-phosphate shunt" evidence=ISS] |
| HP01496 | b.53.1.1 | Ribosomal protein L25-like | Ribosomal protein L25-like | Ribosomal protein L25-like | All beta proteins | [GO:0003735 "structural constituent  of ribosome" evidence=ISS] [GO:0006950 "response to stress"  evidence=ISS] [GO:0008097 "5S rRNA binding" evidence=ISS] |
| HP01497 | c.56.3.1 | Phosphorylase/hydrolase-like | Peptidyl-tRNA hydrolase-like | Peptidyl-tRNA hydrolase-like | Alpha and beta proteins (a/b) | [GO:0004045 "aminoacyl-tRNA hydrolase  activity" evidence=ISS] [GO:0006412 "translation" evidence=ISS] |
| HP01498 | c.55.3.14 | Ribonuclease H-like motif | Ribonuclease H-like | Prp8 beta-finger domain-like | Alpha and beta proteins (a/b) | [GO:0003674 "molecular_function"  evidence=ND] [GO:0008150 "biological_process" evidence=ND] |
| HP01499 | d.136.1.1 | Phospholipase D/nuclease | Phospholipase D/nuclease | Nuclease | Alpha and beta proteins (a+b) | [GO:0005524 "ATP binding" evidence=ISS]  [GO:0008026 "ATP-dependent helicase activity" evidence=ISS]  [GO:0008152 "metabolic process" evidence=ISS] |
| HP01500 | a.118.1.1 | alpha-alpha superhelix | ARM repeat | Armadillo repeat | All alpha proteins |  |
| HP01501 | f.4.1.1 | Transmembrane Beta Bareels | Ompa Like | Outermembrane Proteins | Membrane and cell surface proteins and peptides |  |
| HP01502 | [d.2.1.3](http://scop.berkeley.edu/search/?key="sccs:d.2.1.3") | [Lysozyme-like](http://scop.berkeley.edu/sunid=53954) | [Lysozyme-like](http://scop.berkeley.edu/sunid=53954) | [Phage lysozyme](http://scop.berkeley.edu/sunid=53981) | Alpha and beta proteins (a+b) | [GO:0003674  "molecular_function" evidence=ND] [GO:0005575 "cellular_component"  evidence=ND] [GO:0008150 "biological_process" evidence=ND] |
| HP01503 | d.58.17.1 | [Ferredoxin-like](http://scop.berkeley.edu/sunid=54861) | [HMA, heavy metal-associated domain](http://scop.berkeley.edu/sunid=55008) | [HMA, heavy metal-associated domain](http://scop.berkeley.edu/sunid=55008) | Alpha and beta proteins (a+b) | [GO:0030001 "metal ion  transport" evidence=ISS] [GO:0046873 "metal ion transmembrane  transporter activity" evidence=ISS] |
| HP01504 | [c.66.1.47](http://scop.berkeley.edu/search/?key="sccs:c.66.1.47") | [S-adenosyl-L-methionine-dependent methyltransferases](http://scop.berkeley.edu/sunid=53334&ver=1.75) | [S-adenosyl-L-methionine-dependent methyltransferases](http://scop.berkeley.edu/sunid=53334&ver=1.75) | [Met-10+ protein-like](http://scop.berkeley.edu/sunid=142622&ver=1.75) | Alpha and beta proteins (a/b) | [GO:0003674  "molecular_function" evidence=ND] [GO:0005575 "cellular_component"  evidence=ND] [GO:0008150 "biological_process" evidence=ND] |
| HP01505 | [c.71.1.2](http://scop.berkeley.edu/search/?key="sccs:c.71.1.2") | [Dihydrofolate reductase-like](http://scop.berkeley.edu/sunid=53596) | [Dihydrofolate reductase-like](http://scop.berkeley.edu/sunid=53596) | [RibD C-terminal domain-like](http://scop.berkeley.edu/sunid=142701) | Alpha and beta proteins (a/b) | [GO:0008703  "5-amino-6-(5-phosphoribosylamino)uracil reductase activity"  evidence=ISS] [GO:0008835  "diaminohydroxyphosphoribosylaminopyrimidine deaminase activity"  evidence=ISS] [GO:0009231 "riboflavin biosynthetic process"  evidence=ISS] |
| HP01506 | a.118.1.1 | alpha-alpha superhelix | ARM repeat | Armadillo repeat | All alpha proteins | [GO:0016020 "membrane" evidence=IEA]  [GO:0015813 "L-glutamate transport" evidence=IEA] [GO:0015501  "glutamate:sodium symporter activity" evidence=IEA] [GO:0015293  "symporter activity" evidence=IEA] |
| HP01507 | [d.81.1.2](http://scop.berkeley.edu/search/?key="sccs:d.81.1.2") | [FwdE/GAPDH domain-like](http://scop.berkeley.edu/sunid=55346) | [Glyceraldehyde-3-phosphate dehydrogenase-like, C-terminal domain](http://scop.berkeley.edu/sunid=55347) | [Homoserine dehydrogenase-like](http://scop.berkeley.edu/sunid=55363) | Alpha and beta proteins (a+b) | [GO:0004754  "saccharopine dehydrogenase (NAD+, L-lysine-forming) activity"  evidence=ISS] [GO:0009085 "lysine biosynthetic process"  evidence=ISS] |
| HP01508 | [b.1.11.1](http://scop.berkeley.edu/search/?key="sccs:b.1.11.1") | [Immunoglobulin-like beta-sandwich](http://scop.berkeley.edu/sunid=48725) | [PapD-like](http://scop.berkeley.edu/sunid=49354) | [Pilus chaperone](http://scop.berkeley.edu/sunid=49355) | All beta proteins | [GO:0008150  "biological_process" evidence=ND] |
| HP01509 | [b.1.8.1](http://scop.berkeley.edu/search/?key="sccs:b.1.8.1") | [Immunoglobulin-like beta-sandwich](http://scop.berkeley.edu/sunid=48725) | [Cu,Zn superoxide dismutase-like](http://scop.berkeley.edu/sunid=49329) | [Cu,Zn superoxide dismutase-like](http://scop.berkeley.edu/sunid=49329) | All beta proteins | [GO:0003674  "molecular_function" evidence=ND] [GO:0005575 "cellular_component"  evidence=ND] [GO:0008150 "biological_process" evidence=ND] |
| HP01510 | [d.96.1.3](http://scop.berkeley.edu/search/?key="sccs:d.96.1.3") | [T-fold](http://scop.berkeley.edu/sunid=55619) | [Tetrahydrobiopterin biosynthesis enzymes-like](http://scop.berkeley.edu/sunid=55620) | [DHN aldolase/epimerase](http://scop.berkeley.edu/sunid=55628) | Alpha and beta proteins (a+b) | [GO:0003824 "catalytic activity"  evidence=ISS] [GO:0008152 "metabolic process" evidence=ISS] |
| HP01511 | [f.4.3.3](http://scop.berkeley.edu/search/?key="sccs:f.4.3.3") | [Transmembrane beta-barrels](http://scop.berkeley.edu/sunid=56924) | [Porins](http://scop.berkeley.edu/sunid=56935) | [Ligand-gated protein channel](http://scop.berkeley.edu/sunid=56948) | Membrane and cell surface proteins and peptides |  |
| HP01512 | [f.4.3.3](http://scop.berkeley.edu/search/?key="sccs:f.4.3.3") | [Transmembrane beta-barrels](http://scop.berkeley.edu/sunid=56924) | [Porins](http://scop.berkeley.edu/sunid=56935) | [Ligand-gated protein channel](http://scop.berkeley.edu/sunid=56948) | Membrane and cell surface proteins and peptides | [GO:0015232 "heme  transporter activity" evidence=ISS] [GO:0015886 "heme transport"  evidence=ISS] |
| HP01513 | c.67.1.3 | [PLP-dependent transferase-like](http://scop.berkeley.edu/sunid=53382) | [PLP-dependent transferase-like](http://scop.berkeley.edu/sunid=53382) | [Cystathionine synthase-like](http://scop.berkeley.edu/sunid=53402) | Alpha and beta proteins (a/b) | [GO:0001514  "selenocysteine incorporation" evidence=ISS] [GO:0004125  "L-seryl-tRNASec selenium transferase activity" evidence=ISS]  [GO:0016260 "selenocysteine biosynthetic process" evidence=ISS] |
| HP01514 | [d.202.1.1](http://scop.berkeley.edu/search/?key="sccs:d.202.1.1") | [Transcription factor NusA, N-terminal domain](http://scop.berkeley.edu/sunid=69704) | [Transcription factor NusA, N-terminal domain](http://scop.berkeley.edu/sunid=69704) | [Transcription factor NusA, N-terminal domain](http://scop.berkeley.edu/sunid=69704) | Alpha and beta proteins (a+b) | [GO:0006353  "DNA-dependent transcription, termination" evidence=ISS] |
| HP01515 | [f.26.1.1](http://scop.berkeley.edu/search/?key="sccs:f.26.1.1") | [Bacterial photosystem II reaction centre, L and M subunits](http://scop.berkeley.edu/sunid=81484&ver=1.75) | [Bacterial photosystem II reaction centre, L and M subunits](http://scop.berkeley.edu/sunid=81484&ver=1.75) | [Bacterial photosystem II reaction centre, L and M subunits](http://scop.berkeley.edu/sunid=81484&ver=1.75) | Membrane and cell surface proteins and peptides |  |
| HP01516 | a.74.1.3 | [Cyclin-like](http://scop.berkeley.edu/sunid=47953) | [Cyclin-like](http://scop.berkeley.edu/sunid=47953) | [Retinoblastoma tumor suppressor domains](http://scop.berkeley.edu/sunid=47969) | All alpha proteins |  |
| HP01517 | c.66.1.27 | [S-adenosyl-L-methionine-dependent methyltransferases](http://scop.berkeley.edu/sunid=53334) | [S-adenosyl-L-methionine-dependent methyltransferases](http://scop.berkeley.edu/sunid=53334) | [DNA methylase TaqI, N-terminal domain](http://scop.berkeley.edu/sunid=88787) | Alpha and beta proteins (a/b) | [GO:0009036 "Type II  site-specific deoxyribonuclease activity" evidence=ISS] [GO:0009307  "DNA restriction-modification system" evidence=ISS] |
| HP01518 | [c.55.3.5](http://scop.berkeley.edu/search/?key="sccs:c.55.3.5") | Ribonuclease H-like motif | Ribonuclease H-like | DnaQ-like 3'-5' exonuclease | Alpha and beta proteins (a/b) | [GO:0003674  "molecular_function" evidence=ND] [GO:0008150 "biological_process"  evidence=ND] [GO:0020011 "apicoplast" evidence=RCA] |
| HP01519 | [a.22.1.1](http://scop.berkeley.edu/search/?key="sccs:a.22.1.1") | [Histone-fold](http://scop.berkeley.edu/sunid=47112&ver=1.75) | [Histone-fold](http://scop.berkeley.edu/sunid=47112&ver=1.75) | [Nucleosome core histones](http://scop.berkeley.edu/sunid=47114&ver=1.75) | All alpha proteins | [GO:0008270 "zinc ion binding" evidence=ISS] |
| HP01520 | [c.37.1.12](http://scop.berkeley.edu/search/?key="sccs:c.37.1.12") | [P-loop containing nucleoside triphosphate hydrolases](http://scop.berkeley.edu/sunid=52540) | [P-loop containing nucleoside triphosphate hydrolases](http://scop.berkeley.edu/sunid=52540) | [ABC transporter ATPase domain-like](http://scop.berkeley.edu/sunid=52686) | Alpha and beta proteins (a/b) | [GO:0003674 "molecular_function"  evidence=ND] [GO:0016020 "membrane" evidence=IDA] |
| HP01521 | [c.37.1.19](http://scop.berkeley.edu/search/?key="sccs:c.37.1.19") | [P-loop containing nucleoside triphosphate hydrolases](http://scop.berkeley.edu/sunid=52540) | [P-loop containing nucleoside triphosphate hydrolases](http://scop.berkeley.edu/sunid=52540) | [Tandem AAA-ATPase domain](http://scop.berkeley.edu/sunid=81268) | Alpha and beta proteins (a/b) | [GO:0009307 "DNA restriction-modification system"  evidence=ISS] [GO:0015666 "restriction endodeoxyribonuclease  activity" evidence=ISS] |
| HP01522 |  |  |  |  |  |  |
| HP01523 | [a.24.21.1](http://scop.berkeley.edu/sunid=69009) | [Four-helical up-and-down bundle](http://scop.berkeley.edu/sunid=47161) | [RecG, N-terminal domain](http://scop.berkeley.edu/sunid=69008) | [RecG, N-terminal domain](http://scop.berkeley.edu/sunid=69008) | All alpha proteins | [GO:0004003  "ATP-dependent DNA helicase activity" evidence=ISS] [GO:0006310  "DNA recombination" evidence=ISS] |
| HP01524 | [a.38.1.1](http://scop.berkeley.edu/search/?key="sccs:a.38.1.1") | [HLH-like](http://scop.berkeley.edu/sunid=47458) | [HLH, helix-loop-helix DNA-binding domain](http://scop.berkeley.edu/sunid=47459) | [HLH, helix-loop-helix DNA-binding domain](http://scop.berkeley.edu/sunid=47459) | All alpha proteins |  |
| HP01525 | [f.4.1.1](http://scop.berkeley.edu/search/?key="sccs:f.4.1.1") | [Transmembrane beta-barrels](http://scop.berkeley.edu/sunid=56924) | [OMPA-like](http://scop.berkeley.edu/sunid=56925) | [Outer membrane protein](http://scop.berkeley.edu/sunid=56926) | Membrane and cell surface proteins and peptides |  |
| HP01526 | [d.151.1.1](http://scop.berkeley.edu/search/?key="sccs:d.151.1.1") | [DNase I-like](http://scop.berkeley.edu/sunid=56218) | [DNase I-like](http://scop.berkeley.edu/sunid=56218) | [DNase I-like](http://scop.berkeley.edu/sunid=56218) | Alpha and beta proteins (a+b) | [GO:0006281 "DNA repair"  evidence=ISS] [GO:0008853 "exodeoxyribonuclease III activity"  evidence=ISS] |
| HP01527 | [b.29.1.11](http://scop.berkeley.edu/sunid=49978) | [Concanavalin A-like lectins/glucanases](http://scop.berkeley.edu/sunid=49898) | [Concanavalin A-like lectins/glucanases](http://scop.berkeley.edu/sunid=49898) | [Xylanase/endoglucanase 11/12](http://scop.berkeley.edu/sunid=49978) | All beta proteins | [GO:0008270 "zinc ion binding"  evidence=IEA] [GO:0005575 "cellular_component" evidence=ND]  [GO:0046872 "metal ion binding" evidence=IEA] |
| HP01528 | [a.102.4.4](http://scop.berkeley.edu/search/?key="sccs:a.102.4.4") | [alpha/alpha toroid](http://scop.berkeley.edu/sunid=48207) | [Terpenoid cyclases/Protein prenyltransferases](http://scop.berkeley.edu/sunid=48239) | [Complement components](http://scop.berkeley.edu/sunid=48251) | All alpha proteins |  |
| HP01529 | [c.37.1.20](http://scop.berkeley.edu/search/?key="sccs:c.37.1.20") | [P-loop containing nucleoside triphosphate hydrolases](http://scop.berkeley.edu/sunid=52539) | [P-loop containing nucleoside triphosphate hydrolases](http://scop.berkeley.edu/sunid=52539) | [Extended AAA-ATPase domain](http://scop.berkeley.edu/sunid=81269) | Alpha and beta proteins (a/b) | [GO:0003688 "DNA  replication origin binding" evidence=ISS] [GO:0005524 "ATP binding"  evidence=ISS] [GO:0006270 "DNA replication initiation"  evidence=ISS] [GO:0006275 "regulation of DNA replication"  evidence=ISS] |
| HP01530 | [c.56.2.1](http://scop.berkeley.edu/search/?key="sccs:c.56.2.1") | [Phosphorylase/hydrolase-like](http://scop.berkeley.edu/sunid=53162&ver=1.75) | [Purine and uridine phosphorylases](http://scop.berkeley.edu/sunid=53167&ver=1.75) | [Purine and uridine phosphorylases](http://scop.berkeley.edu/sunid=53167&ver=1.75) | Alpha and beta proteins (a/b) | [GO:0003674  "molecular_function" evidence=ND] [GO:0005575 "cellular_component"  evidence=ND] [GO:0008150 "biological_process" evidence=ND] |
| HP01531 | a.30.6.1 | ROP-like | HP1531-like | HP1531-like | All alpha proteins | [GO:0003677 "DNA  binding" evidence=ISS] |
| HP01532 | [c.80.1.1](http://scop.berkeley.edu/search/?key="sccs:c.80.1.1") | [SIS domain](http://scop.berkeley.edu/sunid=53696) | [SIS domain](http://scop.berkeley.edu/sunid=53696) | double-SIS domain | Alpha and beta proteins (a/b) | [GO:0004360 "glutamine-fructose-6-phosphate transaminase  (isomerizing) activity" evidence=ISS] [GO:0006040 "amino sugar  metabolic process" evidence=ISS] |
| HP01533 | [d.207.1.1](http://scop.berkeley.edu/search/?key="sccs:d.207.1.1") | [Thymidylate synthase-complementing protein Thy1](http://scop.berkeley.edu/sunid=69795) | [Thymidylate synthase-complementing protein Thy1](http://scop.berkeley.edu/sunid=69795) | [Thymidylate synthase-complementing protein Thy1](http://scop.berkeley.edu/sunid=69795) | Alpha and beta proteins (a+b) | [GO:0009265  "2'-deoxyribonucleotide biosynthetic process" evidence=ISS]  [GO:0050797 "thymidylate synthase (FAD) activity" evidence=ISS] |
| HP01534 | [b.113.1.1](http://scop.berkeley.edu/search/?key="sccs:b.113.1.1") | [N-terminal domain of MutM-like DNA repair proteins](http://scop.berkeley.edu/sunid=81625) | [N-terminal domain of MutM-like DNA repair proteins](http://scop.berkeley.edu/sunid=81625) | [N-terminal domain of MutM-like DNA repair proteins](http://scop.berkeley.edu/sunid=81625) | All beta proteins | [GO:0004803 "transposase activity"  evidence=ISS] [GO:0006313 "transposition, DNA-mediated"  evidence=ISS] |
| HP01535 | [d.58.57.1](http://scop.berkeley.edu/search/?key="sccs:d.58.57.1") | [Ferredoxin-like](http://scop.berkeley.edu/sunid=54861) | [Transposase IS200-like](http://scop.berkeley.edu/sunid=143422) | [Transposase IS200-like](http://scop.berkeley.edu/sunid=143422) | Alpha and beta proteins (a+b) | [GO:0004803 "transposase  activity" evidence=ISS] [GO:0006313 "transposition, DNA-mediated"  evidence=ISS] |
| HP01537 | [c.1.8.3](http://scop.berkeley.edu/search/?key="sccs:c.1.8.3") | [TIM beta/alpha-barrel](http://scop.berkeley.edu/sunid=51350) | [(Trans)glycosidases](http://scop.berkeley.edu/sunid=51445) | [beta-glycanases](http://scop.berkeley.edu/sunid=51487) | Alpha and beta proteins (a/b) |  |
| HP01538 | [a.3.1.3](http://scop.berkeley.edu/search/?key="sccs:a.3.1.3") | [Cytochrome c](http://scop.berkeley.edu/sunid=46625) | [Cytochrome c](http://scop.berkeley.edu/sunid=46625) | [Cytochrome bc1 domain](http://scop.berkeley.edu/sunid=46676) | All alpha proteins | [GO:0008121 "ubiquinol-cytochrome-c reductase activity"  evidence=ISS] [GO:0009055 "electron carrier activity" evidence=ISS]  [GO:0009060 "aerobic respiration" evidence=ISS] |
| HP01539 | [f.21.1.2](http://scop.berkeley.edu/search/?key="sccs:f.21.1.2") | [Heme-binding four-helical bundle](http://scop.berkeley.edu/sunid=81344) | [Transmembrane di-heme cytochromes](http://scop.berkeley.edu/sunid=81342) | [Cytochrome b of cytochrome bc1 complex (Ubiquinol-cytochrome c reductase)](http://scop.berkeley.edu/sunid=81642) | Membrane and cell surface proteins and peptides | [GO:0008121 "ubiquinol-cytochrome-c reductase activity"  evidence=ISS] [GO:0009055 "electron carrier activity" evidence=ISS]  [GO:0009060 "aerobic respiration" evidence=ISS] |
| HP01540 | [b.33.1.1](http://scop.berkeley.edu/sunid=50023) | [ISP domain](http://scop.berkeley.edu/sunid=50021) | [ISP domain](http://scop.berkeley.edu/sunid=50021) | [Rieske iron-sulfur protein (ISP)](http://scop.berkeley.edu/sunid=50023) | All beta proteins | [GO:0008121 "ubiquinol-cytochrome-c reductase activity"  evidence=ISS] [GO:0009055 "electron carrier activity" evidence=ISS]  [GO:0009060 "aerobic respiration" evidence=ISS] |
| HP01541 | [c.37.1.19](http://scop.berkeley.edu/search/?key="sccs:c.37.1.19") | [P-loop containing nucleoside triphosphate hydrolases](http://scop.berkeley.edu/sunid=52539) | [P-loop containing nucleoside triphosphate hydrolases](http://scop.berkeley.edu/sunid=52539) | [Tandem AAA-ATPase domain](http://scop.berkeley.edu/sunid=81268) | Alpha and beta proteins (a/b) | [GO:0003690  "double-stranded DNA binding" evidence=ISS] [GO:0006281 "DNA  repair" evidence=ISS] [GO:0016887 "ATPase activity" evidence=ISS] |
| HP01542 | [b.81.1.4](http://scop.berkeley.edu/search/?key="sccs:b.81.1.4") | [Single-stranded left-handed beta-helix](http://scop.berkeley.edu/sunid=51160) | [Trimeric LpxA-like enzymes](http://scop.berkeley.edu/sunid=51161) | [GlmU C-terminal domain-like](http://scop.berkeley.edu/sunid=51171) | All beta proteins | [GO:0003674  "molecular_function" evidence=ND] [GO:0005575 "cellular_component"  evidence=ND] [GO:0008150 "biological_process" evidence=ND] |
| HP01543 | [b.84.3.2](http://scop.berkeley.edu/search/?key="sccs:b.84.3.2") | [Barrel-sandwich hybrid](http://scop.berkeley.edu/sunid=51229) | [Duplicated hybrid motif](http://scop.berkeley.edu/sunid=51261) | [Peptidoglycan hydrolase LytM](http://scop.berkeley.edu/sunid=102006) | All beta proteins | [GO:0004222 "metalloendopeptidase  activity" evidence=ISS] [GO:0006508 "proteolysis" evidence=ISS] |
| HP01544 | [b.84.3.2](http://scop.berkeley.edu/search/?key="sccs:b.84.3.2") | [Barrel-sandwich hybrid](http://scop.berkeley.edu/sunid=51229) | [Duplicated hybrid motif](http://scop.berkeley.edu/sunid=51261) | [Peptidoglycan hydrolase LytM](http://scop.berkeley.edu/sunid=102006) | All beta proteins | [GO:0009405 "pathogenesis" evidence=ISS] |
| HP01545 | [c.72.2.2](http://scop.berkeley.edu/search/?key="sccs:c.72.2.2") | [Ribokinase-like](http://scop.berkeley.edu/sunid=53612) | [MurD-like peptide ligases, catalytic domain](http://scop.berkeley.edu/sunid=53623) | [Folylpolyglutamate synthetase](http://scop.berkeley.edu/sunid=53629) | Alpha and beta proteins (a/b) | [GO:0004326  "tetrahydrofolylpolyglutamate synthase activity" evidence=ISS]  [GO:0006760 "folic acid-containing compound metabolic process"  evidence=ISS] [GO:0008841 "dihydrofolate synthase activity"  evidence=ISS] |
| HP01546 | [c.51.6.2](http://scop.berkeley.edu/search/?key="sccs:c.51.6.2") | [Anticodon-binding domain-like](http://scop.berkeley.edu/sunid=52953&ver=1.75) | [XCC0632-like](http://scop.berkeley.edu/sunid=159594&ver=1.75) | [XCC0632-like](http://scop.berkeley.edu/sunid=159594&ver=1.75) | Alpha and beta proteins (a/b) | [GO:0003674 "molecular_function"  evidence=ND] [GO:0008150 "biological_process" evidence=ND] |
| HP01547 | c.26.1.1 | Adenine nucleotide alpha hydrolase-like | Nucleotidylyl transferase | Class I aminoacyl-tRNA synthetases (RS), catalytic domain | Alpha and beta proteins (a/b) | [GO:0004823 "leucine-tRNA ligase  activity" evidence=ISS] [GO:0006429 "leucyl-tRNA aminoacylation"  evidence=ISS] |
| HP01548 | [d.2.1.3](http://scop.berkeley.edu/search/?key="sccs:d.2.1.3") | [Lysozyme-like](http://scop.berkeley.edu/sunid=53954) | [Lysozyme-like](http://scop.berkeley.edu/sunid=53954) | [Phage lysozyme](http://scop.berkeley.edu/sunid=53981) | Alpha and beta proteins (a+b) | [GO:0008270 "zinc ion binding" evidence=IEA]  [GO:0046872 "metal ion binding" evidence=IEA] |
| HP01549 | [h.4.3.1](http://scop.berkeley.edu/search/?key="sccs:h.4.3.1") | [Antiparallel coiled-coil](http://scop.berkeley.edu/sunid=58086) | [Colicin Ia, N-terminal domain](http://scop.berkeley.edu/sunid=58096) | [Colicin Ia, N-terminal domain](http://scop.berkeley.edu/sunid=58096) |  | [GO:0006886  "intracellular protein transport" evidence=ISS] [GO:0008565  "protein transporter activity" evidence=ISS] [GO:0009306 "protein  secretion" evidence=ISS] |
| HP01550 | [h.4.3.1](http://scop.berkeley.edu/search/?key="sccs:h.4.3.1") | [Antiparallel coiled-coil](http://scop.berkeley.edu/sunid=58086) | [Colicin Ia, N-terminal domain](http://scop.berkeley.edu/sunid=58096) | [Colicin Ia, N-terminal domain](http://scop.berkeley.edu/sunid=58096) |  | [GO:0006886  "intracellular protein transport" evidence=ISS] [GO:0008565  "protein transporter activity" evidence=ISS] [GO:0009306 "protein  secretion" evidence=ISS] |
| HP01551 | [d.225.1.1](http://scop.berkeley.edu/search/?key="sccs:d.225.1.1") | [Multidrug efflux transporter AcrB TolC docking domain; DN and DC subdomains](http://scop.berkeley.edu/sunid=82713) | [Multidrug efflux transporter AcrB TolC docking domain; DN and DC subdomains](http://scop.berkeley.edu/sunid=82713) | [Multidrug efflux transporter AcrB TolC docking domain; DN and DC subdomains](http://scop.berkeley.edu/sunid=82713) | Alpha and beta proteins (a+b) | [GO:0005515 "protein binding" evidence=IPI] |
| HP01552 | [a.25.1.2](http://scop.berkeley.edu/search/?key="sccs:a.25.1.2") | [Ferritin-like](http://scop.berkeley.edu/sunid=47239) | [Ferritin-like](http://scop.berkeley.edu/sunid=47239) | [Ribonucleotide reductase-like](http://scop.berkeley.edu/sunid=47253) | All alpha proteins | [GO:0005886 "plasma  membrane" evidence=IEA] [GO:0016021 "integral to membrane"  evidence=IEA] [GO:0016020 "membrane" evidence=IEA;IDA] |
| HP01553 | [c.37.1.19](http://scop.berkeley.edu/search/?key="sccs:c.37.1.19") | [P-loop containing nucleoside triphosphate hydrolases](http://scop.berkeley.edu/sunid=52539) | [P-loop containing nucleoside triphosphate hydrolases](http://scop.berkeley.edu/sunid=52539) | [Tandem AAA-ATPase domain](http://scop.berkeley.edu/sunid=81268) | Alpha and beta proteins (a/b) | [GO:0004003  "ATP-dependent DNA helicase activity" evidence=ISS] [GO:0006281  "DNA repair" evidence=ISS] |
| HP01554 | [c.23.15.1](http://scop.berkeley.edu/search/?key="sccs:c.23.15.1") | [Flavodoxin-like](http://scop.berkeley.edu/sunid=52171) | [Ribosomal protein S2](http://scop.berkeley.edu/sunid=52313) | [Ribosomal protein S2](http://scop.berkeley.edu/sunid=52313) | Alpha and beta proteins (a/b) | [GO:0003735 "structural constituent  of ribosome" evidence=ISS] [GO:0042254 "ribosome biogenesis"  evidence=ISS] |
| HP01555 | [d.43.1.1](http://scop.berkeley.edu/search/?key="sccs:d.43.1.1") | [EF-Ts domain-like](http://scop.berkeley.edu/sunid=54712) | [Elongation factor Ts (EF-Ts), dimerisation domain](http://scop.berkeley.edu/sunid=54713) | [Elongation factor Ts (EF-Ts), dimerisation domain](http://scop.berkeley.edu/sunid=54713) | Alpha and beta proteins (a+b) | [GO:0003746  "translation elongation factor activity" evidence=ISS] [GO:0006414  "translational elongation" evidence=ISS] |
| HP01556 | [e.3.1.1](http://scop.berkeley.edu/search/?key="sccs:e.3.1.1") | [beta-lactamase/transpeptidase-like](http://scop.berkeley.edu/sunid=56600) | [beta-lactamase/transpeptidase-like](http://scop.berkeley.edu/sunid=56600) | [beta-Lactamase/D-ala carboxypeptidase](http://scop.berkeley.edu/sunid=56602) | Multi-domain proteins (alpha and beta) | [GO:0008233 "peptidase  activity" evidence=ISS] [GO:0008658 "penicillin binding"  evidence=ISS] [GO:0009252 "peptidoglycan biosynthetic process"  evidence=ISS] |
| HP01557 | [a.7.2.1](http://scop.berkeley.edu/search/?key="sccs:a.7.2.1") | [Spectrin repeat-like](http://scop.berkeley.edu/sunid=46965) | [Enzyme IIa from lactose specific PTS, IIa-lac](http://scop.berkeley.edu/sunid=46973) | [Enzyme IIa from lactose specific PTS, IIa-lac](http://scop.berkeley.edu/sunid=46973) | All alpha proteins | [GO:0001539  "ciliary or flagellar motility" evidence=ISS] [GO:0005198  "structural molecule activity" evidence=ISS] [GO:0009425  "bacterial-type flagellum basal body" evidence=ISS] |
| HP01558 | [b.152.1.1](http://scop.berkeley.edu/search/?key="sccs:b.152.1.1") | Flagellar hook protein flgE | Flagellar hook protein flgE | Flagellar hook protein flgE | All beta proteins | [GO:0001539 "ciliary  or flagellar motility" evidence=ISS] [GO:0005198 "structural  molecule activity" evidence=ISS] [GO:0009429 "bacterial-type  flagellum basal body, proximal rod" evidence=ISS] |
| HP01559 | [b.152.1.1](http://scop.berkeley.edu/search/?key="sccs:b.152.1.1") | Flagellar hook protein flgE | Flagellar hook protein flgE | Flagellar hook protein flgE | All beta proteins | [GO:0001539 "ciliary  or flagellar motility" evidence=ISS] [GO:0005198 "structural  molecule activity" evidence=ISS] [GO:0009429 "bacterial-type  flagellum basal body, proximal rod" evidence=ISS] |
| HP01560 | a.63.1.1 | Apolipophorin-III | Apolipophorin-III | Apolipophorin-III | All alpha proteins | [GO:0003674  "molecular_function" evidence=ND] |
| HP01561 | a.3.1.5 | Cytochrome c | Cytochrome c | Di-heme cytochrome c peroxidase | All alpha proteins | [GO:0006810 "transport" evidence=TAS] [GO:0042626 "ATPase  activity, coupled to transmembrane movement of substances"  evidence=TAS] |
| HP01562 | d.153.1.3 | Ntn hydrolase-like | N-terminal nucleophile aminohydrolases (Ntn hydrolases) | Penicillin V acylase | Alpha and beta proteins (a+b) | [GO:0003674  "molecular_function" evidence=ND] [GO:0005575 "cellular_component"  evidence=ND] [GO:0008150 "biological_process" evidence=ND] |
| HP01563 | c.47.1.10 | Thioredoxin fold | Thioredoxin fold | Glutathione peroxidase-like | Alpha and beta proteins (a/b) | [GO:0006979 "response  to oxidative stress" evidence=ISS] [GO:0016209 "antioxidant  activity" evidence=ISS] |
| HP01564 | c.94.1.1 | Periplasmic binding protein-like II | Periplasmic binding protein-like II | Periplasmic binding protein-like II | Alpha and beta proteins (a/b) | [GO:0003674 "molecular_function"  evidence=ND] [GO:0008150 "biological_process" evidence=ND] |
| HP01565 | e.3.1.1 | beta-lactamase/transpeptidase-like | beta-lactamase/transpeptidase-like | beta-Lactamase/D-ala carboxypeptidase | Multi-domain proteins (alpha and beta) | [GO:0008658  "penicillin binding" evidence=ISS] [GO:0009252 "peptidoglycan  biosynthetic process" evidence=ISS] |
| HP01566 | [d.20.1.1](http://scop.berkeley.edu/sunid=54496&ver=1.75) | [UBC-like](http://scop.berkeley.edu/sunid=54494&ver=1.75) | [UBC-like](http://scop.berkeley.edu/sunid=54494&ver=1.75) | [UBC-related](http://scop.berkeley.edu/sunid=54496&ver=1.75) | Alpha and beta proteins (a+b) | [GO:0003674 "molecular_function"  evidence=ND] [GO:0008150 "biological_process" evidence=ND] |
| HP01567 | [c.37.1.8](http://scop.berkeley.edu/search/?key="sccs:c.37.1.8") | [P-loop containing nucleoside triphosphate hydrolases](http://scop.berkeley.edu/sunid=52539) | [P-loop containing nucleoside triphosphate hydrolases](http://scop.berkeley.edu/sunid=52540) | [G proteins](http://scop.berkeley.edu/sunid=52592) | Alpha and beta proteins (a/b) | [GO:0005525 "GTP binding"  evidence=ISS] [GO:0008152 "metabolic process" evidence=ISS] |
| HP01568 | [b.108.1.2](http://scop.berkeley.edu/search/?key="sccs:b.108.1.2") | [Triple-stranded beta-helix](http://scop.berkeley.edu/sunid=69348) | [Phage fibre proteins](http://scop.berkeley.edu/sunid=69349) | [Tail-associated lysozyme gp5, C-terminal domain](http://scop.berkeley.edu/sunid=69353) | All beta proteins | [GO:0003674 "molecular_function"  evidence=ND] [GO:0008150 "biological_process" evidence=ND] |
| HP01569 | b.108.1.2 | Triple-stranded beta-helix | Phage fibre proteins | Tail-associated lysozyme gp5, C-terminal domain | All beta proteins |  |
| HP01570 | c.108.1.5 | HAD-like | HAD-like | Probable phosphatase YrbI | Alpha and beta proteins (a/b) | [GO:0009103  "lipopolysaccharide biosynthetic process" evidence=IEA;IMP]  [GO:0046872 "metal ion binding" evidence=IEA] [GO:0019143  "3-deoxy-manno-octulosonate-8-phosphatase activity" evidence=IEA] |
| HP01571 | b.7.3.1 | C2 domain-like | PHL pollen allergen | PHL pollen allergen | All beta proteins | [GO:0003674 "molecular_function"  evidence=ND] [GO:0008150 "biological_process" evidence=ND] |
| HP01572 | d.2.1.6 | Lysozyme-like | Lysozyme-like | Bacterial muramidase, catalytic domain | Alpha and beta proteins (a+b) | [GO:0009253 "peptidoglycan catabolic process" evidence=ISS]  [GO:0016798 "hydrolase activity, acting on glycosyl bonds"  evidence=ISS] |
| HP01573 | c.1.9.12 | TIM beta/alpha-barrel | Metallo-dependent hydrolases | TatD Mg-dependent DNase-like | Alpha and beta proteins (a/b) | [GO:0008152 "metabolic process"  evidence=ISS] [GO:0016787 "hydrolase activity" evidence=ISS] |
| HP01574 | b.43.4.3 | Reductase/isomerase/elongation factor common domain | Riboflavin synthase domain-like | Riboflavin synthase | All beta proteins | [GO:0004746  "riboflavin synthase activity" evidence=ISS] [GO:0009231  "riboflavin biosynthetic process" evidence=ISS] |
| HP01575 | d.367.1.1 | EscU C-terminal domain-like | EscU C-terminal domain-like | EscU C-terminal domain-like | Alpha and beta proteins (a+b) | [GO:0003674 "molecular_function"  evidence=ND] [GO:0008150 "biological_process" evidence=ND] |
| HP01576 | c.37.1.12 | P-loop containing nucleoside triphosphate hydrolases | P-loop containing nucleoside triphosphate hydrolases | ABC transporter ATPase domain-like | Alpha and beta proteins (a/b) | [GO:0006810 "transport"  evidence=ISS] [GO:0042626 "ATPase activity, coupled to  transmembrane movement of substances" evidence=ISS] |
| HP01577 | f.58.1.1 | MetI-like | MetI-like | MetI-like | Membrane and cell surface proteins and peptides | [GO:0006810 "transport" evidence=ISS] [GO:0042626  "ATPase activity, coupled to transmembrane movement of substances"  evidence=ISS] |
| HP01578 | c.68.1.4 | Nucleotide-diphospho-sugar transferases | Nucleotide-diphospho-sugar transferases | Galactosyltransferase LgtC | Alpha and beta proteins (a/b) |  |
| HP01579 | b.34.9.1 | SH3-like barrel | Tudor/PWWP/MBT | Tudor domain | All beta proteins |  |
| HP01580 | a.111.1.1 | Acid phosphatase/Vanadium-dependent haloperoxidase | Acid phosphatase/Vanadium-dependent haloperoxidase | Type 2 phosphatidic acid phosphatase, PAP2 | All alpha proteins | [GO:0003674 "molecular_function"  evidence=ND] [GO:0008150 "biological_process" evidence=ND] |
| HP01581 | d.2.1.3 | Lysozyme-like | Lysozyme-like | Phage lysozyme | Alpha and beta proteins (a+b) | [GO:0000287 "magnesium  ion binding" evidence=ISS] [GO:0005515 "protein binding"  evidence=IPI] [GO:0016780 "phosphotransferase activity, for other  substituted phosphate groups" evidence=ISS] |
| HP01582 | c.1.24.1 | TIM beta/alpha-barrel | Pyridoxine 5'-phosphate synthase | Pyridoxine 5'-phosphate synthase | Alpha and beta proteins (a/b) | [GO:0003824  "catalytic activity" evidence=ISS] [GO:0008615 "pyridoxine  biosynthetic process" evidence=ISS] |
| HP01583 | c.77.1.3 | Isocitrate/Isopropylmalate dehydrogenase-like | Isocitrate/Isopropylmalate dehydrogenase-like | PdxA-like | Alpha and beta proteins (a/b) | [GO:0003824 "catalytic activity" evidence=ISS] [GO:0008615  "pyridoxine biosynthetic process" evidence=ISS] |
| HP01584 | c.55.1.5 | Ribonuclease H-like motif | Actin-like ATPase domain | BadF/BadG/BcrA/BcrD-like | Alpha and beta proteins (a/b) | [GO:0006508  "proteolysis" evidence=ISS] |
| HP01585 | b.152.1.1 | Flagellar hook protein flgE | Flagellar hook protein flgE | Flagellar hook protein flgE | All beta proteins | [GO:0001539 "ciliary  or flagellar motility" evidence=ISS] [GO:0005198 "structural  molecule activity" evidence=ISS] [GO:0009425 "bacterial-type  flagellum basal body" evidence=ISS] |
| HP01586 | a.133.1.2 | Phospholipase A2, PLA2 | Phospholipase A2, PLA2 | Vertebrate phospholipase A2 | All alpha proteins |  |
| HP01587 | d.89.1.2 | Origin of replication-binding domain, RBD-like | Origin of replication-binding domain, RBD-like | Replication initiation protein E1 | Alpha and beta proteins (a+b) |  |
| HP01588 |  |  |  |  |  | [GO:0015031 "protein transport"  evidence=IEA] [GO:0031902 "late endosome membrane" evidence=IEA]  [GO:0019904 "protein domain specific binding" evidence=IPI] |
| HP01589 | f.1.1.1 | Toxins' membrane translocation domains | Colicin | Colicin | Membrane and cell surface proteins and peptides |  |
| HP01590 | a.39.1.5 | EF Hand-like | EF Hand | Penta-EF-hand proteins | All alpha proteins |  |
