# Supplementary material for: Structure Based Annotation of Helicobacter pylori Strain 26695 Proteome
Source: PLoS One. 2014 Dec 30;9(12):e115020. doi: 10.1371/journal.pone.0115020 (PMC4280198; doi:10.1371/journal.pone.0115020)
Supplement: S6 Table — Comparison of previous annotation from Pylorigene database and new annotation from our work. (DOC) [file pone.0115020.s006.doc]

| **Supplementary Table VI. Comparison of the annotation of *H. pylori* 26695 strain proteins from Pylorigene database and the results from this work** | | |
| --- | --- | --- |
| **­­ Gene name** | **Annotation in Pylorigene database** | **Annotation through our methodology** |
| HP0001 | Predicted N utilization substance protein. | N utilization substance protein b homolog |
| HP0002 | Predicted riboflavin synthase beta chain. | Riboflavin synthase |
| HP0003 | Predicted 3-deoxy-D-manno-octulosonic acid 8-phosphate synthetase | 2-dehydro-3-deoxyphosphooctonate aldolase |
| HP0004 | Predicted beta-carbonic anhydrase | Beta carbonic anhydrase |
| HP0005 | Predicted orotidine 5'-phosphate decarboxylase | Orotidine 5'-phosphate decarboxylase |
| HP0006 | Predicted pantoate-beta-alanine ligase | Pantoate--beta-alanine ligase |
| HP0007 | Predicted coding region HP0007 with no homologue in the databases | 23 aa |
| HP0008 | Predicted coding region HP0008 with no homologue in the databases | 27 aa |
| HP0009 | Outer membrane protein HopZ involved in adhesion | Endoglucanase 9g |
| HP0010 | Chaperone and heat shock protein | CPN60 (GROEL) |
| HP0011 | Cochaperone involved in proper folding of proteins during heat-shock | heat shock protein |
| HP0012 | Predicted DNA primase/synthesis of primers for Okazaki fragments elongation | Dna primase |
| HP0013 | Predicted coding region HP0013 | (5-methylaminomethyl-2-thiouridylate)-methyltransferase trmu |
| HP0014 | Predicted coding region HP0014 | 2-keto-3-deoxy-d-arabinonate dehydratase |
| HP0015 | Predicted coding region HP0015 with no homologue in the databases | |  | transcriptional regulator | | --- | --- | |
| HP0016 | Predicted coding region HP0016 with no homologue in the databases | Signaling Protein |
| HP0017 | ComB4 competence protein involved in a type IV secretion system that mediates  Natural transformation | Type iv secretory pathway virb4 components-like protein |
| HP0018 | Predicted coding region HP0018 | TPR repeat-containing protein |
| HP0019 | Chemotaxis protein/CheA-MCP interaction modulator | Chemotaxis protein |
| HP0020 | Predicted carboxynorspermidine decarboxylase | Carboxynorspermidine decarboxylase |
| HP0021 | Predicted coding region HP0021 | Acid phosphatase |
| HP0022 | Predicted integral membrane protein | Protein in the alkaline phosphatase supefamily |
| HP0023 | Predicted coding region HP0023 with no homologue in the databases | trypsin inhibitor |
| HP0024 | Predicted coding region HP0024 with no homologue in the databases | K+-channel voltage-sensor paddle domain |
| HP0025 | Outer membrane protein HopD with porin properties | Outer membrane protein H1 |
| HP0026 | Predicted citrate synthase | Citrate synthase |
| HP0027 | Predicted isocitrate dehydrogenase | Isocitrate dehydrogenase [NADP] |
| HP0028 | Predicted coding region HP0028 | 177 aa |
| HP0029 | Predicted dethiobiotin synthetase | Dethiobiotin synthetase |
| HP0030 | Predicted coding region HP0030 | probable translation factor |
| HP0031 | Predicted coding region HP0031 | ATP-binding domain of protein MJ0577 |
| HP0032 | Predicted coding region HP0032 | ATP-dependent clp protease adaptor protein clps |
| HP0033 | Predicted ATP-dependent C1p protease, ATP-binding subunit/chaperone protein | chaperone protein CLPB PROTEIN |
| HP0034 | Predicted aspartate alpha-decarboxylase precursor | Aspartate 1-decarboxylase precursor |
| HP0035 | Predicted coding region HP0035 | 97 aa |
| HP0036 | Predicted coding region HP0036 | Hydrolase/htra proteases |
| HP0037 | Predicted coding region HP0037 with no homologue in the databases | Nuclear transport |
| HP0037.1 | ComB7 putative lipoprotein involved in natural transformation efficiency | |  | Comb7 competence protein | | --- | --- | |
| HP0038 | ComB8 competence protein involved in a type IV secretion system that mediates natural transformation | Type iv secretion system |
| HP0039 | ComB9 competence protein involved in a type IV secretion system that mediates natural transformation | outer membrane complex of a type IV secretion system |
| HP0040 | ComB9 competence protein involved in a type IV secretion system that mediates natural transformation | TraF protein(outer membrane complex of a type IV secretion system) |
| HP0041 | ComB10 competence protein involved in a type IV secretion system that mediates natural transformation | ComB10 competence protein |
| HP0042 | ComB10 competence protein involved in a type IV secretion system that mediates natural transformation | comb10 of the com type iv secretion system |
| HP0043 | Predicted phosphomannose isomerase/GDP-mannose pyrophosphorylase | Mannose-6-phosphate isomerase |
| HP0044 | Predicted GDP-D-mannose dehydratase | Gdp-mannose 4,6-dehydratase |
| HP0045 | GDP-fucose synthetase | Gdp-fucose synthetase |
| HP0046 | Predicted coding region HP0046 with no homologue in the databases | Transcription regulation |
| HP0047 | Predicted hydrogenase expression/formation protein | Hydrogenase expression/formation protein hype |
| HP0048 | HypF, a predicted transcriptional regulator of hydrogenase activity, essential for hydrogenase maturation | Hypf Hydrogenase maturation factor |
| HP0049 | Non-functional type II restriction endonuclease in a silent state or degenerated enough to be considered as a pseudogene | Putative peptidyl-arginine deiminase |
| HP0050 | GAGG site-specific adenine-methyltransferase involved in type II restriction/modification system | Gagg site-specific adenine-methyltransferase involved in type ii restriction/modification system |
| HP0051 | CCTC site-specific cytosine-methyltransferase involved in type II restriction/modification system | DNA (cytosine-5) methylase |
| HP0052 | Predicted coding region HP0052 with no homologue in the databases | 330 aa |
| HP0053 | Sequence recognition type II restriction endonuclease which cleaves very frequently DNA substrates | Hin4II restriction endonuclease |
| HP0054 | Asymetric site-specific adenine-methyltransferase involved in type I restriction/modification system | Cytosine-specific methyltransferase |
| HP0055 | Predicted sodium/proline symporter | Sodium/glucose cotransporter |
| HP0056 | Predicted proline/delta 1-pyrroline-5-carboxylate dehydrogenase | Proline dehydrogenase/delta-1-pyrroline-5-carboxylate dehydrogenase |
| HP0057 | Predicted coding region HP0057 with no homologue in the databases | DNA binding motif |
| HP0058 | Predicted coding region HP0058 with no homologue in the databases | 121 aa |
| HP0059 | Predicted coding region HP0059 with no homologue in the databases | 284 aa |
| HP0060 | Predicted coding region HP0060 with no homologue in the databases | diol dehydratase-reactivating factor |
| HP0061 | Predicted coding region HP0061 with no homologue in the databases | Protection of telomeres protein 1 |
| HP0062 | Predicted coding region HP0062 with no homologue in the databases | Chain A, Crystal Structure Of Hypothetical Protein Of Hp0062 ( 3FX7) |
| HP0063 | Predicted coding region HP0063 with no homologue in the databases | Cell adhesion/ vinculin isoform |
| HP0064 | Predicted coding region HP0064 | Putative glucan synthesis regulator of SMI1/KNR4 |
| HP0065 | Predicted coding region HP0065 | Glutamic acid/alanine-rich protein |
| HP0066 | Predicted ATP-binding protein | Dna translocase ftsk |
| HP0067 | Urease accessory protein/predicted chaperone protein stabilizing the apo-urease complex | Urease accessory protein uref |
| HP0068 | Urease accessory protein/predicted GTP-binding protein | Probable hydrogenase nickel incorporation |
| HP0069 | Urease accessory protein/predicted modulator of the ured-apourease activation complex | Urease accessory protein ure |
| HP0070 | Urease accessory protein/predicted nickel-ion metallochaperone protein | Urease accessory protein uree |
| HP0071 | Urease accessory protein/ph-dependent urea-transporter | Acid-activated urea channel |
| HP0072 | Urease B subunit/Urea amidohydrolase | Fusion of urease beta and gamma subunits |
| HP0073 | Urease A subunit/Urea amidohydrolase | Urease subunit alpha |
| HP0074 | Predicted lipoprotein signal peptidase involved in the cleavage of signal peptides and anchoring of lipoproteins to outer membrane | Large-conductance mechanosensitive channel |
| HP0075 | Phosphoglucosamine mutase | Phosphoglucosamine mutase |
| HP0076 | Predicted ribosomal protein S20 involved in 30S ribosome subunit assembly | 30s ribosomal protein s20 |
| HP0077 | Predicted peptide chain release factor RF-1 involved in termination of ribosomal polypeptide synthesis | Peptide chain release factor 1 |
| HP0078 | Reminiscent outer membrane protein HorA | allene oxide cyclase |
| HP0079 | Outer membrane protein HorA | Uv excision repair protein, outer membrane protein HorA |
| HP0080 | Predicted coding region HP0080 with no homologue in databases | Ribosomal Protein L35 |
| HP0081 | Predicted coding region HP0081 with no homologue in databases | 40 aa protein |
| HP0082 | Predicted methyl-accepting chemotaxis transmembrane sensory protein (MCP-like protein) | |  | HAMP, methyl-accepting chemotaxis  protein | | --- | --- | |
| HP0083 | Predicted ribosomal protein S9 involved in 30S ribosome subunit assembly | 16s rrna ribosome |
| HP0084 | Predicted 50S ribosomal protein L13 | 50s ribosomal protein l13 |
| HP0085 | Predicted coding region HP0085 with no homologue in databases | 62 aa protein |
| HP0086 | Malate:quinone oxireductase, an unusual type of enzyme of citric acid cycle, responsible for the oxidation of malate to oxaloacetate | conserved exported protein |
| HP0087 | Predicted coding region HP0087 | Putative gamma-d-glutamyl-l-diamino acid endopeptidase |
| HP0088 | Major transcription initiation factor sigma-80 | Dna-directed rna polymerase alpha |
| HP0089 | Predicted 5'-methylthioadenosine nucleosidase/S-adenosylhomocysteine nucleosidase involved in S-adenosylmethionine recycling into methionine | 5'-methylthioadenosine nucleosidase/s-adenosylhomocysteine nucleosidase |
| HP0090 | Predicted malonyl coenzyme A-acyl carrier protein transacylase | Malonyl Coenzyme A-Acyl Carrier Protein  Transacyl |
| HP0091 | GATC site-specific type II restriction endonuclease, isoschizomer of MboI,MjaII and DpnII,that is either silent or degenerated enough to become a pseudogene | type II restriction enzyme R protein (hsdR) |
| HP0092 | GATC site-specific adenine-methyltransferase involved in type II restriction-modification system | adenine-specific Methyltransferase MboIIA |
| HP0093 | Alpha-(1,2)fucosyltransferase | Alpha-(1,6)-fucosyltransferase |
| HP0094 | Alpha-(1,2)fucosyltransferase | alpha-(1,2) fucosyltransferase |
| HP0095 | Predicted coding region HP0095 with no homologue in the databases | 176 aa |
| HP0096 | Predicted D-2-hydroxyacid dehydrogenase | Phosphoglycerate dehydrogenase |
| HP0097 | Predicted coding region HP0097 with no homologue in the databases | Chaperone protein |
| HP0098 | Predicted threonine synthase | Threonine synthase |
| HP0099 | Predicted methyl-accepting chemotaxis transmembrane sensory protein (MCP-like protein) | HAMP, methyl-accepting chemotaxis protein i |
| HP0100 | Predicted coding region HP0100 | Electron transport protein |
| HP0101 | Predicted coding region HP0101 with no homologue in the databases | outer membrane protein |
| HP0102 | Predicted glycosyl transferase | glycosyltransferase protein |
| HP0103 | Predicted methyl-accepting chemotaxis transmembrane sensory protein (MCP-like protein) | HAMP, methyl-accepting chemotaxis protein i |
| HP0104 | Predicted 2',3'-cyclic-nucleotide 2'-phosphodiesterase | 5'-nucleotidase |
| HP0105 | Autoinducer-2 production protein involved in quorum-sensing | Autoinducer-2 production protein luxs |
| HP0106 | Predicted cystathionine gamma-synthase | Cystathionine gamma-lyase-like protein |
| HP0107 | Predicted cysteine synthetase | Cysteine synthase a |
| HP0108 | Predicted coding region HP0108 with no homologue in the databases | 5-methylcytosine-specific restriction enzyme |
| HP0109 | Chaperone and heat shock protein 70/DnaK | Nucleotide exchange factor grpe bound to the ATPase domain of the molecular chaperone DnaK |
| HP0110 | Co-chaperone and heat shock protein 24/GrpE | Nucleotide exchange factor grpe |
| HP0111 | Predicted heat-inducible transcription repressor of Class I heat shock genes | Predicted heat-inducible transcription repressor of class 1heat shock genes |
| HP0112 | Predicted L-fuculose-1-phosphate aldolase involved in fucose metabolism | Predicted l-fuculose-1-phosphate aldolase involved in fucose metabolism |
| HP0113 | Predicted coding region HP0113 with no homologue in the databases | Putative N-acetylmannosamine-6-phosphate 2-epimerase |
| HP0114 | Predicted coding region HP0114 | Alpha-2,3/8-sialyltransferase |
| HP0115 | Flagellin B | Flagellin b |
| HP0116 | DNA topoisomerase I involved in tertiary DNA structure | DNA topoisomerase i |
| HP0117 | Predicted coding region HP0117 | Molybdenum cofactor biosynthesis protein A |
| HP0118 | Predicted coding region HP0118 | COLICIN E3 (Ribosome Inhibitor) |
| HP0119 | Predicted coding region HP0119 | COLICIN E3 (Ribosome Inhibitor) |
| HP0120 | Predicted coding region HP0120 | Major vault protein (cytoplasmic ribonucleoprotein) |
| HP0121 | Predicted phosphoenolpyruvate synthase | Phosphoenolpyruvate synthase |
| HP0122 | Predicted coding region HP0122 with no homologue in the databases | 43 aa protein |
| HP0123 | Predicted threonyl-tRNA synthetase | Threonyl-trna synthetase |
| HP0124 | Predicted translation initiation factor IF-3 | Translation initiation factor 3 |
| HP0125 | Predicted ribosomal protein L35 | 50s ribosomal protein L35 |
| HP0126 | Predicted ribosomal protein L20 | 50s ribosomal protein L20 |
| HP0127 | Predicted outer membrane protein HorB | outer membrane protein |
| HP0128 | Predicted coding region HP0128 with no homologue in the databases | Non-structural glycoprotein 4 |
| HP0129 | Predicted coding region HP0129 | Transcription antitermination protein nusg |
| HP0130 | Predicted coding region HP0130 with no homologue in the databases | DPS family dna-binding stress response protein |
| HP0131 | Predicted coding region HP0131 with no homologue in the databases | cortexillin I/GCN4 hybrid peptide |
| HP0132 | Predicted L-serine/L-threonine deaminase | l-serine/l-threonine deaminase |
| HP0133 | Predicted L-serine transporter | Arginine/agmatine antiporter |
| HP0134 | Predicted tyrosine-regulated 3-deoxy-D-arabino-heptulosonate 7-phosphate synthase (DAHP synthase) | 3-deoxy-d-arabino-heptulosonate  7-phosphate synthetase |
| HP0135 | Predicted coding region HP0135 with no homologue in the databases | 44 aa protein |
| HP0136 | Predicted bacterioferritin comigratory protein. | Peroxiredoxin, bacterioferritin comigratory protein |
| HP0137 | Predicted coding region HP0137. | 211 aa protein |
| HP0138 | Predicted iron-sulfur protein. | Acetyl-CoA decarboxylase/synthase alpha subunit |
| HP0139 | Predicted secreted protein | Acetyl-CoA decarboxylase/synthase alpha subunit |
| HP0140 | Predicted L-lactate permease | lactate transmembrane  transporter |
| HP0141 | Predicted L-lactate permease | lactate transmembrane transporter |
| HP0142 | Predicted A/G-specific adenine glycosylase | Adenine glycosylase |
| HP0143 | Predicted C(4)-dicarboxylates and tricarboxylates/succinate antiporter | C(4)-dicarboxylates and tricarboxylates/succinate antiporter |
| HP0144 | Predicted cytochrome c oxidase heme b and copper-binding subunit | Cytochrome c oxidase |
| HP0145 | Predicted cytochrome c oxidase monoheme subunit | Predicted cytochrome c oxidase monoheme subunit |
| HP0146 | Predicted cytochrome c oxidase subunit Q | RNA Binding Protein |
| HP0147 | Predicted cytochrome c oxidase diheme subunit | Cytochrome c oxidase, cbb3-type, subunit P |
| HP0148 | Predicted coding region HP0148 with no homologue in the databases | Phosphatase and actin regulator 1 |
| HP0149 | Predicted coding region HP0149 with no homologue in the databases | 194 aa |
| HP0150 | Predicted coding region HP0150 with no homologue in the databases | 196 aa |
| HP0151 | Predicted membrane protein | Transcription repressor |
| HP0152 | Predicted coding region HP0152 | Menaquinone biosynthetic enzyme |
| HP0153 | Recombinase | Protein reca |
| HP0154 | Predicted enolase | Enolase |
| HP0155 | Predicted coding region HP0155 with no homologue in the databases | Max protein (transcription) |
| HP0156 | Predicted coding region HP0156 | Type iv pilus biogenesis and competence protein |
| HP0157 | Predicted shikimic acid kinase I | Shikimate kinase |
| HP0158 | Predicted coding region HP0158 | Transferase/ sensor protein. |
| HP0159 | Predicted LPS 1,2-glycosyltransferase | Predicted lps 1,2-glycosyltransferase |
| HP0160 | Cystein-rich protein D | Cysteine rich protein b |
| HP0161 | Predicted coding region HP0161 | Mitogen-activated protein kinase |
| HP0162 | Predicted coding region HP0162 | Transcriptional regulatory protein |
| HP0163 | Predicted delta-aminolevulinic acid dehydratase | Delta-aminolevulinic acid dehydratase |
| HP0164 | Signal-transducing protein, histidine kinase | Signal-transducing protein, histidine kinase |
| HP0165 | Signal-transducing protein, histidine kinase | Sensory rhodopsin II transducer |
| HP0166 | Response regulator | Dna binding response regulator d |
| HP0167 | Predicted coding region HP0167 with no homologue in the databases | Nitrite transporter NirC |
| HP0168 | Predicted coding region HP0168 | Tetratricopeptide repeat domain protein |
| HP0169 | Predicted collagenase | Citrate lyase |
| HP0170 | Predicted coding region HP0170 | Signaling protein/ chemotaxis protein chey |
| HP0171 | Predicted peptide chain release factor RF-2 | Release factor 2/Polypeptide chain release factor 2 |
| HP0172 | Predicted molybdopterin biosynthesis protein involved in the activation of molybdenium | Molybdopterin biosynthesis moea protein |
| HP0173 | Predicted flagellar biosynthetic protein | Ligase/nuclear protein/ conjugating enzyme ubc9 |
| HP0174 | Predicted coding region HP0174 | sulfate permease,Uncharacterized protein involved in cysteine biosynthesis |
| HP0175 | Predicted peptidyl-prolyl cis-trans isomerase C involved in protein maturation | Cell-binding factor 2 |
| HP0176 | Predicted fructose-bisphosphate aldolase | Fructose-bisphosphate aldolase |
| HP0177 | Predicted translation elongation factor EF-P involved in peptidyltransferase reactions | Elongation factor p |
| HP0178 | Predicted sialic acid synthase | Polysialic acid capsule biosynthesis protein siac |
| HP0179 | Predicted ABC transporter/ATP-binding protein | Lipoprotein-releasing system atp-binding protein |
| HP0180 | Predicted apolipoprotein N-acyltransferase | Lipoprotein-releasing system  ATP-binding protein |
| HP0181 | Predicted coding region HP0181 | Hydrolase, carbon-nitrogen family |
| HP0182 | Predicted lysyl-tRNA synthetase | Protein (lysyl-trna synthetase) |
| HP0183 | Predicted serine hydroxymethyltransferase | Serine hydroxymethyltransferase |
| HP0184 | Predicted coding region HP0184 | H. pylori predicted coding region HP0184(2ATZ) |
| HP0185 | Predicted coding region HP0185 | Putative uncharacterized protein spr0440(glycoside hydrolase domain) |
| HP0186 | Predicted coding region HP0186 with no homologue in the databases | Endonuclease I |
| HP0187 | Predicted coding region HP0187 with no homologue in the databases | 95aa |
| HP0188 | Predicted coding region HP0188 with no homologue in the databases | Ubiquitin |
| HP0189 | Predicted coding region HP0189 | 177 aa |
| HP0190 | Predicted phospholipase D-family protein | Phospholipase d |
| HP0191 | Fumarate reductase, iron-sulfur subunit | Quinol-fumarate reductase flavoprotein subunit a |
| HP0192 | Fumarate reductase, flavoprotein subunit | Quinol-fumarate reductase flavoprotein subunit a |
| HP0193 | Fumarate reductase, cytochrome b subunit | Fumarate reductase cytochrome b subunit |
| HP0194 | Predicted triosephosphate isomerase | Triosephosphate isomerase |
| HP0195 | Predicted enoyl-(acyl-carrier-protein) reductase (NADH) | Enoyl-[acyl-carrier-protein] reductase [nadh] |
| HP0196 | Predicted UDP-3-0-(3-hydroxymyristoyl) glucosamine N-acyltransferase | UDP-3-o-[3-hydroxymyristoyl] glucosamine n-acyltransferase |
| HP0197 | Predicted S-adenosylmethionine synthetase | S-adenosylmethionine synthetase |
| HP0198 | Predicted nucleoside diphosphate kinase | Nucleoside diphosphate kinase |
| HP0199 | Predicted coding region HP0199 | Beta-lactamase |
| HP0200 | Predicted ribosomal protein L32 | 50S ribosomal protein L32 |
| HP0201 | Predicted fatty acid/phospholipid synthesis protein | Fatty acid/phospholipid synthesis protein plsX |
| HP0202 | Predicted beta-ketoacyl-acyl carrier protein synthase III | Beta-ketoacyl-acyl carrier protein synthase iii |
| HP0203 | Predicted coding region HP0203 with no homologue in the databases | BFT-3( zinc-dependentmetallopeptidase ) |
| HP0204 | Predicted coding region HP0204 with no homologue in the databases | 127 aa protein |
| HP0205 | Predicted coding region HP0205 with no homologue in the databases | Elongation factor 3a |
| HP0206 | Predicted coding region HP0205 with no homologue in the databases | chromosome partition protein mukb, linker |
| HP0207 | Predicted ATP-binding protein | Nucleotide-binding protein |
| HP0208 | Predicted LPS 1,2-glycosyltransferase | lipopolysaccharide biosynthesis protein |
| HP0209 | Predicted outer membrane protein HofA | outer membrane protein |
| HP0210 | Predicted chaperone and heat shock protein 90 | Chaperone protein htpg |
| HP0211 | Cysteine-rich protein A | Cysteine rich protein b |
| HP0212 | Succinyl-diaminopimelate desuccinylase | Succinyl-diaminopimelate desuccinylase |
| HP0213 | Predicted glucose inhibited division protein A | tRNA uridine 5-carboxymethylaminomethyl modif |
| HP0214 | Predicted sodium-dependent transporter | sodium-dependent dicarboxylate transporter. |
| HP0215 | Predicted CDP-diacylglycerol synthetase | Ammonium transporter Rh type C |
| HP0216 | Predicted 1-deoxy-D-xylulose 5-phosphate reductoisomerase involved in isoprenoid synthesis | 1-deoxy-d-xylulose 5-phosphate reductoisomerase |
| HP0217 | Predicted beta-1,4-N-acetylgalactosamyltransferase involved in LPS synthesis | UDP-galactofuranosyl transferase GlfT2 |
| HP0218 | Predicted coding region HP0218 | Putative phosphatidylethanolamine-binding protein |
| HP0219 | Predicted coding region HP0219 with no homologue in the databases | |  | helix-turn-helix domain protein | | --- | --- | |
| HP0220 | Cysteine desulfurase involved in [Fe-S] cluster synthesis | Cysteine desulfurase |
| HP0221 | NifU scaffold protein involved in [Fe-S] cluster assembly | NifU-like protein |
| HP0222 | Predicted coding region HP0222 | Putative transcriptional regulator |
| HP0223 | Predicted DNA repair protein | dna repair protein/reca protein |
| HP0224 | Predicted peptide methionine sulfoxide reductase | Peptide methionine sulfoxide reductase MSRA/MSRB |
| HP0225 | Predicted coding region HP0225 with no homologue in the databases | SET binding factor 1 |
| HP0226 | Predicted integral membrane protein | Membrane protein |
| HP0227 | Predicted outer membrane protein HopM | Outer membrane protein H1 |
| HP0228 | Predicted sulfate permease | Uracil permease |
| HP0229 | Outer membrane protein HopA with porin properties | Outer membrane protein H1 |
| HP0230 | Predicted 3-deoxy-manno-octulosonate cytidylyltransferase (CMP-Kdo synthetase) | 3-deoxy-manno-octulosonate cytidylyltransferase |
| HP0231 | Predicted coding region HP0231 with no homologue in the databases | Thiol:disulfide interchange protein dsbc |
| HP0232 | Secreted protein involved in flagellar motility | flagellar motility |
| HP0233 | Predicted glutathionespermidine synthetase involved in polyamine synthesis | Bifunctional glutathionylspermidine synthetase |
| HP0234 | Predicted integral membrane protein | Membrane protein formate dehydrogenase-N (Fdn-N) |
| HP0235 | Cysteine-rich protein E | Cysteine rich protein |
| HP0236 | Predicted coding region HP0236 | cytochrome c oxidase, cbb3-type, subunit n |
| HP0237 | Predicted porphobilinogen deaminase | Porphobilinogen deaminase |
| HP0238 | Predicted prolyl-tRNA synthetase | Prolyl-trna synthetase |
| HP0239 | Predicted glutamyl-tRNA reductase | Glutamyl-trna reductase |
| HP0240 | Predicted octaprenyl-diphosphate synthase | Geranylgeranyl diphosphate synthetase |
| HP0241 | Predicted coding region HP0241 with no homologue in the databases | PKD domain, Polycystic Kidney Disease |
| HP0242 | Predicted coding region HP0241 with no homologue in the databases | 2BO3_A  ( hypothetical protein fromHelicobacter pylori with a novel fold) |
| HP0243 | Neutrophil activating protein (bacterioferritin) | Neutrophil-activating protein a |
| HP0244 | Signal-transducing protein, histidine kinase | sensory histidine kinase |
| HP0245 | Predicted coding region HP0245 | Acyl carrier protein synthase |
| HP0246 | Predicted flagellar basal-body P-ring protein | Macrophage colony-stimulating factor 1 receptor |
| HP0247 | Predicted ATP-dependent RNA helicase | Putative atp-dependent rna helicase DHH1 |
| HP0248 | Predicted coding region HP0248 | Membrane protein |
| HP0249 | Predicted coding region HP0249 with no homologue in the databases | CARDB domain |
| HP0250 | Predicted ATPase protein of the oligopeptide transport system | |  | Translation/transport Protein | | --- | --- | |
| HP0251 | Predicted integral membrane protein of the oligopeptide transport system | D-methionine transport system permease protein |
| HP0252 | Outer membrane protein HopF | Outer membrane protein HopF |
| HP0253 | Outer membrane protein HopG | |  | Membrane Protein | | --- | --- | |
| HP0254 | Outer membrane protein HopG | Outer membrane protein H1 |
| HP0255 | Predicted adenylosuccinate synthetase involved in de novo biosynthesis of AMP | Adenylosuccinate synthetase |
| HP0256 | Predicted coding region HP0256 with no homologue in the databases | COLICIN E3 |
| HP0257 | Predicted secreted protein | COLICIN E3 |
| HP0258 | Predicted integral membrane protein | |  | Regulator of sigma E protease | | --- | --- | |
| HP0259 | Predicted large subunit of the single-stranded DNA-specific dexodeoxyribonuclease VII | DNA Binding Protein |
| HP0260 | TCGA site-specific m6A methyltransferase involved in type III restriction-modification systems | Tcga site-specific m6a methyltransferase involved in type iii restriction-modification systems/pvuii dna methyltransferase |
| HP0261 | Predicted coding region HP0261 with no homologue in the databases | uncharacterized NTF2-like protein |
| HP0262 | Non-functional type II restriction endonuclease in a silent state or degenerated enough to be considered as a pseudogene | Restriction endonuclease EcoRII |
| HP0263 | Non-functional cytosine methyltransferase belonging to type II DNA restriction-modification systems that is either silent or degenerated enough to become a pseudogene | Tcga site-specific m6a methyltransferase involved in type iii restriction-modification |
| HP0264 | Predicted ATP-dependent protease binding subunit/heat shock protein | CLPB (a molecular chaperone) PROTEIN |
| HP0265 | Predicted cytochrome c biogenesis protein | 26s proteasome regulatory subunit, Potassium voltage-gated channel subfamily A |
| HP0266 | Predicted dihydroorotase | Dihydroorotase |
| HP0267 | Predicted detoxifying protein involved in deamination/dechlorination | 5-methylthioadenosine/S-adenosylhomocysteinedeaminase /Amidohydrolase family protein |
| HP0268 | Predicted coding region HP0268 | putative Mandelate racemase/muconate lactoniz |
| HP0269 | Predicted ATP-binding protein | A radical s-adenosylmethionine methylthiotransferase / Biotin synthetase |
| HP0270 | Predicted coding region HP0270 | glycerol-3-phosphate acyltransferase |
| HP0271 | Predicted coding region HP0271 with no homologue in the databases | Exopolyphosphatase |
| HP0272 | Predicted coding region HP0272 with no homologue in the databases | PilO protein(bacterial virulence factors) |
| HP0273 | Predicted coding region HP0273 with no homologue in the databases | PilO protein(bacterial virulence factors) |
| HP0274 | Predicted coding region HP0274 | 132aa |
| HP0275 | Predicted coding region HP0275 | Udp-n-acetylglucosamine--peptide n-acetylglucosaminyltransferase |
| HP0276 | Predicted coding region HP0276 | Indole-3-glycerol phosphate synthase |
| HP0277 | Predicted ferrodoxin | ferrodoxin |
| HP0278 | Predicted guanosine pentaphosphate phosphohydrolase involved in stringent response factor | Exopolyphosphatase |
| HP0279 | Predicted lipopolysaccharide heptosyltransferase-1 involved in lipopolysaccharide core biosynthesis | Lipopolysaccharide heptosyltransferase-1 |
| HP0280 | Predicted lipid A biosynthesis acyltransferase | glycerol-3-phosphate acyltransferase |
| HP0281 | tRNA-guanine transglycosylase involved in tRNA modification and maturation | Queuine trna-ribosyltransferase |
| HP0282 | Predicted coding region HP0282 | Metal transport, membrane protein/calcium-gated potassium channel mthk |
| HP0283 | Predicted 3-dehydroquinate synthase | 3-dehydroquinate synthase |
| HP0284 | Predicted integral membrane protein of unknown function | Membrane protein/small-conductance mechanosensitive channel. |
| HP0285 | Predicted coding region HP0285 | Biotin synthetase |
| HP0286 | Predicted cell division protein/metalloprotease | 26S proteasome |
| HP0287 | Predicted coding region HP0287 with no homologue in the databases | 2-hydroxy-6-oxo-6-phenylhexa-2,4-dienoate hydrolase |
| HP0288 | Predicted coding region HP0288 | D(3) dopamine receptor, Lysozyme chimera |
| HP0289 | Predicted toxin-like outer membrane protein/vacuolating cytotoxin (VacA) paralogue | toxin-like outer membrane protein |
| HP0290 | Predicted diaminopimelate decarboxylase | Diaminopimelate decarboxylase |
| HP0291 | Predicted chorismate mutase involved in phenylalanine synthesis | chorismate mutase |
| HP0292 | Predicted coding region HP0292 | 290 aa |
| HP0293 | Predicted para-aminobenzoate (PABA)-synthetase | Anthranilate synthase |
| HP0294 | Aliphatic amidase involved in nitrogen metabolism | Aliphatic amidase |
| HP0295 | Predicted flagellar-hook associated protein 3 | |  | Flagellar hook-associated protein 3 | | --- | --- | |
| HP0296 | Predicted ribosomal protein L21 | ribosomal protein L21 |
| HP0297 | Predicted ribosomal protein L27 | Ribosomal 23S rna |
| HP0298 | Predicted periplasmic dipeptide-binding protein involved in the dipeptide transport system and chemotaxis | Dipeptide-binding protein |
| HP0299 | Predicted dipeptide permease protein involved in the dipeptide transport system | D-methionine transport system permease protein |
| HP0300 | Predicted dipeptide transport system permease protein | Sulfate/molybdate ABC transporter, permease p |
| HP0301 | Predicted dipeptide transporte system ATP-binding protein | Methionine import ATP-binding protein |
| HP0302 | Predicted dipeptide transporte system ATP-binding protein | Methionine import ATP-binding protein |
| HP0303 | Predicted GTP-binding protein of the GTP1/Obg family involved in stress response | SPO0B-associated gtp-binding protein |
| HP0304 | Predicted coding region HP0304 with no homologue in the databases | Alginate lyase |
| HP0305 | Predicted coding region HP0305 | Cellulose-binding protein |
| HP0306 | Predicted glutamate-1-semialdehyde aminotransferase involved in heme and porphyrin biosynthesis | Glutamate-1-semialdehyde 2,1-aminomutase |
| HP0307 | Predicted coding region HP0307 | Protein glpG(rhomboid protease family) |
| HP0308 | Predicted coding region HP0308 with no homologue in the databases | Amyloid beta A4 precursor protein-binding family |
| HP0309 | Predicted N-carbomoyl-D-amino acid amidohydrolase | N-carbamyl-d-amino acid amidohydrolase |
| HP0310 | Predicted coding region HP0310 | Polysaccharide deacetylase family protein |
| HP0311 | Predicted coding region HP0311 with no homologue in the databases | NUDIX hydrolase |
| HP0312 | Predicted ATP/GTP binding protein | GTP-dependent HYDROGENASE EXPRESSION/FORMATION PROTEIN (HYPB) |
| HP0313 | Predicted transporter | Glycerol-3-phosphate transporter |
| HP0314 | Predicted coding region HP0314 with no homologue in the databases | Hypothetical ubiquitin-conjugating enzyme LOC |
| HP0315 | Predicted virulence-associate protein D | Immunoglobulin g-binding protein g, virulence-associated |
| HP0316 | Predicted coding region HP0316 with no homologue in the databases | transcription activator of multidrug-efflux transporter genes mta |
| HP0317 | Predicted outer membrane protein HopU | Glycerol-3-phosphate transporter (Outer membrane protein H1) |
| HP0318 | Predicted coding region HP0318 | Heme oxygenase |
| HP0319 | Predicted arginyl-tRNA synthetase | Arginyl-trna synthetase |
| HP0320 | Sec-independent protein translocase protein involved in the twin-arginine translocation system | Sec-independent protein translocase protein t |
| HP0321 | Predicted guanylate kinase | Guanylate kinase |
| HP0322 | Predicted poly E-rich protein | structure of the dna repair protein hhr23a /UV excision repair protein RAD23 homolog A |
| HP0323 | Predicted membrane bound endonuclease | endonuclease |
| HP0324 | Predicted outer membrane protein HorC | Outer membrane protein A |
| HP0325 | Predicted flagellar basal-body L-ring protein | Thymidylate kinase |
| HP0326 | Predicted CMP-N-acetylneuraminic acid synthetase | cytidine monophospho-N-acetylneuraminic acid synthetase |
| HP0327 | Predicted flagellar biosynthesis protein G | Acetyltransferase |
| HP0328 | Predicted tetraacyldisaccharide-1-P 4'-kinase | Tetraacyldisaccharide 4'-kinase |
| HP0329 | Predicted NH(3)-dependent NAD+ synthetase | NH(3)-dependent NAD(+) synthetase |
| HP0330 | Predicted ketol-acid reductoisomerase | Ketol-acid reductoisomerase |
| HP0331 | Predicted MinD cell division inhibitor protein | Probable cell division inhibitor mind |
| HP0332 | Predicted MinE protein involved in septum localization | Cell division topological specificity factor |
| HP0333 | DNA processing chain A | Putative dna processing protein |
| HP0334 | Predicted coding region HP0334 | Putative holliday junction resolvase |
| HP0335 | Cysteine-rich protein B | Cysteine rich protein b |
| HP0336 | Cysteine-rich protein B | Cysteine rich protein b |
| HP0337 | Predicted coding region HP0337 with no homologue in the databases | Odorant Binding Protein |
| HP0338 | Predicted coding region HP0338 with no homologue in the databases | Lysozyme |
| HP0339 | Predicted coding region HP0339 | Lysozyme |
| HP0340 | Predicted coding region HP0340 with no homologue in the databases | Chaperone Hsp31 |
| HP0341 | Predicted coding region HP0341 with no homologue in the databases | 31 aa protein |
| HP0342 | Predicted coding region HP0342 | Membrane Protein |
| HP0343 | Predicted coding region HP0343 with no homologue in the databases | Histone acetyltransferase |
| HP0344 | Predicted coding region HP0344 with no homologue in the databases | Forkhead box protein (DNA-binding protein) |
| HP0345 | Predicted coding region HP0345 with no homologue in the databases | Potassium channel subfamily K member 4 |
| HP0346 | Predicted coding region HP0346 with no homologue in the databases | Apolipoprotein E |
| HP0347 | Predicted pseudouridine synthase D involved in 23S rRNA base modifications | Ribosomal large subunit pseudouridine synthase d |
| HP0348 | Predicted single-stranded-DNA-specific exonuclease | Single-stranded dna specific exonuclease recj |
| HP0349 | Predicted CTP synthetase | Ctp synthetase |
| HP0350 | Predicted coding region HP0350 with no homologue in the databases | Class A nonspecific acid phosphatase PhoN |
| HP0351 | Predicted flagellar basal-body M-ring protein | protein transport/ type iii secretion system protein |
| HP0352 | Predicted flagellar motor switch protein | Flagellar motor switch protein |
| HP0353 | Predicted flagellar export protein | V-type ATP synthase subunit E |
| HP0354 | Predicted 1-deoxyxylulose-5-phosphate synthase involved in thiamin, pyridoxol and isopentenyl diphosphate synthesis | 1-deoxy-d-xylulose-5-phosphate synthase |
| HP0355 | Predicted GTP-binding membrane protein | GTP-binding protein LEPA |
| HP0356 | Predicted coding region HP0356 with no homologue in the databases | Putative endonuclease |
| HP0357 | Predicted short chain dehydrogenase | Short-chain type dehydrogenase/reductase |
| HP0358 | Predicted outer membrane protein | amino outer membrane protein |
| HP0359 | Predicted coding region HP0359 with no holomologue in the databases | 21aa |
| HP0360 | UDP-glucose 4-epimerase | Udp-glucose 4-epimerase |
| HP0361 | Predicted pseudouridylate synthase I | Trna pseudouridine synthase a |
| HP0362 | Predicted integral membrane protein | Nadh-quinone oxidoreductase subunit a |
| HP0363 | Predicted L-isoaspartyl-protein carboxyl methyltransferase | Protein-l-isoaspartate o-methyltransferase |
| HP0364 | Predicted ribonucleoside-diphosphate reductase 1 beta subunit | Ribonucleoside-diphosphate reductase subunit beta |
| HP0365 | Predicted coding region HP0365 with no homologue in the databases | Ectatomin(toxin) |
| HP0366 | Predicted sugar nucleotide biosynthesis | Aminotransferase |
| HP0367 | Predicted coding region HP0367 with no homologue in the databases | RNA-directed RNA polymerase |
| HP0368 | Non-functional type II restriction endonuclease in a silent state or degenerated enough to be considered as a pseudogene | putative elicitor-responsive gene |
| HP0369 | Non-functional site-specific adenine methyltransferase in a silent state or degenerated enough to be considered as a pseudogene | Adenine-N6-dna-methyltransferase TAQI |
| HP0370 | Predicted biotin carboxylase involved in long-chain fatty acid synthesis | Biotin carboxylase |
| HP0371 | Predicted biotin carboxyl carrier protein | Biotinyl domain of acetyl-coenzyme  a carboxylase |
| HP0372 | Predicted deoxycytidine triphosphate deaminase | Deoxycytidine triphosphate deaminase |
| HP0373 | Predicted outer membrane protein HomC | outer membrane protein |
| HP0374 | Predicted coding region HP0374 | rRNA methyltransferase |
| HP0375 | Predicted coding region HP0375 with no homologue in the databases | Sphingosine 1-phosphate receptor 1, Lysozyme |
| HP0376 | Predicted ferrochelatase | Ferrochelatase, mitochondrial |
| HP0377 | Predicted DsbC-like protein | Thioredoxin Disulfide Isomerase |
| HP0378 | Predicted biogenesis protein involved in cytochrome c-type maturation | Predicted biogenesis protein involved in cytochrome c-type maturation |
| HP0379 | Alpha1,3-fucosyltransferase | Alpha1,3-fucosyltransferase |
| HP0380 | Predicted glutamate dehydrogenase | Glutamate dehydrogenase |
| HP0381 | Predicted S-adenosylmethionine-dependent methyltransferase | N5-glutamine methyltransferase/ Protein methyltransferase hemK |
| HP0382 | Predicted zinc metalloprotease | Predicted zinc metalloprotease |
| HP0383 | Predicted coding region HP0383 with no homologue in the databases | Sensor protein qseC |
| HP0384 | Predicted coding region HP0384 | Cell division protein FTSN |
| HP0385 | Predicted coding region HP0385 with no homologue in the databases | Monopolin complex subunit CSM1 |
| HP0386 | Predicted coding region HP0386 with no homologue in the databases | DNA Binding Protein |
| HP0387 | Predicted primosomal protein replication factor | Transcription-repair coupling factor, Primosomal protein |
| HP0388 | Predicted coding region HP0388 | tRNA (cmo5U34)-methyltransferase |
| HP0389 | Iron-dependent superoxide dismutase involved in detoxification of radicals and oxidative-stress | Superoxide dismutase |
| HP0390 | Predicted thiol peroxidase | Thiol peroxidase |
| HP0391 | Predicted purine-binding chemotaxis protein/CheA-MCP coupling protein | Chemotaxis signal transduction protein |
| HP0392 | Autophosphorylating histidine kinase | signal-transducing histidine kinase |
| HP0393 | Chemotaxis protein/CheA-MCP interaction modulator | Response regulator receiver |
| HP0394 | Predicted coding region HP0394 | Hydrolase/exonuclease |
| HP0395 | Predicted coding region HP0395 | Alanine racemase 1 |
| HP0396 | Predicted 3-octaprenyl-4-hydroxybenzoate carboxy-lyase involved in ubiquinone synthesis | 3-octaprenyl-4-hydroxybenzoate carboxy-lyase |
| HP0397 | Predicted 3-phosphoglycerate dehydrogenase | D-3-phosphoglycerate dehydrogenase |
| HP0398 | Predicted coding region HP0398 with no homologue in the databases | Procarboxypeptidase |
| HP0399 | Predicted 30S ribosomal protein S1 | RNA-binding domain proteins |
| HP0400 | Predicted isoprenyl pyrophosphate/dimethylallyl diphosphate synthetase involved in isoprenoid biosynthesis | 4-hydroxy-3-methylbut-2-enyl diphosphate  reductase |
| HP0401 | Predicted 3-phosphoshikimate 1-carboxyvinyltransferase | 5-enolpyruvylshikimate-3-phosphate synthase |
| HP0402 | Predicted phenylalanyl-tRNA synthetase beta subunit | Phenylalanyl-trna synthetase alpha chain |
| HP0403 | Predicted phenylalanyl-tRNA synthetase alpha subunit | Phenylalanyl-trna synthetase alpha chain |
| HP0404 | Predicted ADP hydrolase of the HIT protein family | Human protein kinase c interacting protein 1 |
| HP0405 | Predicted NifS-like protein | Cysteine desulfurase NIFS |
| HP0406 | Predicted coding region HP0406 | Homer protein homolog 3 |
| HP0407 | Predicted S/N-oxide reductase | Dimethyl sulfoxide reductase |
| HP0408 | Predicted coding region HP0408 with no homologue in the databases | Dynemicin thioesterase |
| HP0409 | Predicted guanosine-5'-monophosphate synthase involved in de novo synthesis of guanosine nucleotides | Gmp synthase |
| HP0410 | Predicted neuraminyllactose-binding hemagglutinin homolog/paralog of HpaA | Putative neuraminyllactose-binding hemagglutinin |
| HP0411 | Predicted coding region HP0411 with no homologue in the databases | Peptidyl-prolyl cis-trans isomerase |
| HP0412 | Predicted coding region HP0412 with no homologue in the databases | ERBB-2 receptor protein-tyrosine kinase |
| HP0413 | Predicted IS606 transposase B | transposase-like protein |
| HP0414 | Predicted IS606 transposase A | TRANSPOSASE |
| HP0415 | Predicted integral membrane protein of unknown function | Small-conductance mechanosensitive channel in membrane |
| HP0416 | Predicted cyclopropane fatty acid synthase | Mycolic acid synthase |
| HP0417 | Predicted methionyl-tRNA synthetase | Methionyl-trna synthetase |
| HP0418 | Predicted coding region HP0418 | UDP-N-acetylmuramoylalanine-D-glutamyl-lysine |
| HP0419 | Predicted S-adenosylmethionine-dependent methyltransferase | S-adenosylmethionine dependent  methyltransferase |
| HP0420 | Predicted coding region HP0420 | Phenylacetic acid degradation protein paai |
| HP0421 | Predicted polysaccharide biosynthesis protein | Catalytic domain of cholestral alpha glucosyl transferase |
| HP0422 | Predicted arginine decarboxylase | Biosynthetic arginine decarboxylase |
| HP0423 | Predicted coding region HP0423 | Transcription |
| HP0424 | Predicted coding region HP0424 | Clpb protein/chaperone |
| HP0425 | Predicted coding region HP0425 with no homologue in the databases | Exonuclease recj |
| HP0426 | Predicted coding region HP0426 | Dna binding protein |
| HP0427 | Predicted coding region HP0427 | |  | putative lipoprotein | | --- | --- | |
| HP0428 | Predicted coding region HP0428 homologous to the plasmid T478 phage/colicin/tellurite resistance cluster terY protein | Cell Adhesion |
| HP0429 | Predicted coding region HP0429 with no homologue in the databases | 12aa |
| HP0430 | Predicted coding region HP0430 with no homologue in the databases | Von willebrand factor(hemostatic) |
| HP0431 | Predicted serine/threonine phosphatase 2C homolog | Protein serine-threonine phosphatase |
| HP0432 | Predicted serine/threonine kinase C-like protein | |  | Phosphorylase kinase | | --- | --- | |
| HP0433 | Predicted coding region HP0433 with no homologue in the databases | |  | Acyl Coa Binding Protein | | --- | --- | |
| HP0434 | Predicted coding region HP0434 with no homologue in the databases | Transcription regulator |
| HP0435 | Predicted coding region HP0435 | DNA-binding protein SMUBP-2 |
| HP0436 | Predicted coding region HP0436 with no homologue in the databases | Putative sensor-like histidine kinase |
| HP0437 | Predicted IS605 transposase A | Transposase |
| HP0438 | Predicted IS605 transposase B | Endonuclease VIII |
| HP0439 | Predicted DNA transformation competence ComB8 homologue | type iv secretion system protein virb8 |
| HP0440 | Predicted DNA topoisomerase I | |  | DNA Topoisomerase I | | --- | --- | |
| HP0441 | Predicted ATPase/DNA transfer protein | Conjugal transfer protein TRWB |
| HP0442 | Predicted coding region HP0442 with no homologue in the databases | 16S rRNA |
| HP0443 | Predicted coding region HP0443 with no homologue in the databases | 100 aa |
| HP0444 | Predicted coding region HP0444 with no homologue in the databases | DNA primase |
| HP0445 | Predicted coding region HP0445 with no homologue in the databases | Pyrrolidone-carboxylate peptidase |
| HP0446 | Predicted coding region HP0446 | transcriptional regulator, Crp/Fnr family |
| HP0447 | Predicted DNA helicase | ATP-dependent helicase NAM7 |
| HP0448 | Predicted coding region HP0448 with no homologue in the databases | 128 aa |
| HP0449 | Predicted coding region HP0449 with no homologue in the databases | Rna-binding protein |
| HP0450 | Predicted coding region HP0450 with no homologue in the databases | Small conductance calcium-activated potassium |
| HP0451 | Predicted coding region HP0451 | AgI/II(Cell Adhesion) |
| HP0452 | Predicted coding region HP0452 | Atpase rava(regulatory atpase variant complex with adp), hydrolase |
| HP0453 | Predicted coding region HP0453 with no homologue in the databases | Cold-shock domain, Ribosomal binding Protein |
| HP0454 | Predicted coding region HP0454 with no homologue in the databases | 6-phosphofructo-2-kinase/fructose-2,6-biphosphatase |
| HP0455 | Predicted coding region HP0455 | Dual-specificity tyrosine phosphatase |
| HP0456 | Predicted coding region HP0456 with no homologue in the databases | Signaling Protein |
| HP0457 | Predicted coding region HP0457 with no homologue in the databases | 16S rRNA |
| HP0458 | Predicted coding region HP0458 with no homologue in the databases | 78 aa |
| HP0459 | Predicted ATPase/DNA transfer protein | bacterial conjugation protein TrwB resembling ring helicases and F1-ATPase |
| HP0460 | Predicted coding region HP0460 with no homologue in the databases | COLICIN E3 |
| HP0461 | Predicted coding region HP0461 with no homologue in the databases | ZINC finger dna binding domain |
| HP0462 | Predicted type I restriction enzyme S protein | Type I restriction-modification enzyme, S subunit |
| HP0463 | Predicted type I restriction enzyme M protein | Type I restriction-modification system methyltransferase subunit |
| HP0464 | Predicted type I restriction enzyme R protein | Type I site-specific restriction-modification system, R (restriction) subunit and related helicases  [Defense mechanisms] |
| HP0465 | Predicted coding region HP0465 | alpha-2,3/8-sialyltransferase |
| HP0466 | Predicted coding region HP0466 | tellurite resistance TerB family protein |
| HP0467 | Predicted integral membrane protein | integral membrane protein |
| HP0468 | Predicted coding region HP0468 | QUINOL-FUMARATE REDUCTASE FLAVOPROTEIN SUBUNIT A |
| HP0469 | Predicted coding region HP0469 | Type i restriction-modification enzyme |
| HP0470 | Predicted oligoendopeptidase F involved in oligopeptide degradation | peptidase M3B, oligoendopeptidase F |
| HP0471 | Predicted glutathione-regulated potassium-efflux system protein | Putativepeptidase/ NADH-quinone oxidoreductase subunit l. |
| HP0472 | Predicted outer membrane protein HorE | outer membrane protein |
| HP0473 | Predicted molybdenum ABC transporter, periplasmic molybdate-binding protein | Periplasmic molybdate-binding protein |
| HP0474 | Predicted molybdenum ABC transporter, permease protein | Sulfate/molybdate ABC transporter, ATP-binding protein |
| HP0475 | Predicted molybdenum ABC transporter, ATP-binding protein | molybdenum abc transporter, atp-binding protein |
| HP0476 | Predicted glutamyl-tRNA synthetase | Glutamyl-tRNA synthetase |
| HP0477 | Predicted outer membrane protein HopJ | Outer membrane protein W |
| HP0478 | ATTAAT site-specific adenine methyltransferase involved in type II restriction-modification system | type IIG restriction endonuclease/restriction endonuclease  adenine-specific DNA methyltransferase |
| HP0479 | Non-functional ATTAAT site-specific type II restriction endonuclease in a silent state or degenerated enough to be considered as a pseudogene | |  | ADP-heptose lps heptosyltransferase II | | --- | --- | |
| HP0480 | Predicted GTP-binding protein of the TypA subfamily | Tetracycline resistance protein |
| HP0481 | Non-functional type II adenine specific DNA methyltransferase in a silent state or degenerated enough to be considered as a pseudogene | PROTEIN (ADENINE-SPECIFIC METHYLTRANSFERASE DPNII 1) |
| HP0482 | Non-functional type II restriction endonuclease involved in DNA cleavage by sequence recognition | endonuclease ii |
| HP0483 | Non-functional type II cytosine specific DNA methyltransferase in a silent state or degenerated enough to be considered as a pseudogene | 351 aa |
| HP0484 | Non-functional ACGT site-specific type II restriction endonuclease in a silent state or degenerated enough to be considered as a pseudogene | Protein Transport |
| HP0485 | Predicted catalase involved in hydrogen peroxyde detoxification | Catalase |
| HP0486 | Predicted outer membrane protein HofC | Anaerobically-induced outer membrane porin |
| HP0487 | Predicted outer membrane protein HofD | Anaerobically-induced outer membrane porin |
| HP0488 | Predicted coding region HP0488 | Seca-signal peptide complex/protein transport |
| HP0489 | Predicted coding region HP0489dicted coding region HP | Apolipoprotein A-IV (critical mediators of lipid metabolism ) |
| HP0490 | Predicted potassium channel protein | Calcium-gated potassium channel mthK |
| HP0491 | Predicted ribosomal protein L28 | | 50S ribosomal protein L28 | | --- | |
| HP0492 | Predicted neuraminyllactose-binding hemagglutinin homolog/paralog of HpaA | neuraminyllactose-binding hemagglutinin homolog |
| HP0493 | Predicted phospho-N-acetylmuramoyl-pentapeptide transferase | phospho-N-acetylmuramoyl-pentapeptide transferase |
| HP0494 | Predicted UDP-N-acetylmuramoylalanine-D-glutamate ligase | UDP-n-acetylmuramoyl-l-alanine:d-glutamate ligase |
| HP0495 | Predicted coding region HP0495 | Putative nickel-responsive regulator |
| HP0496 | Predicted coding region HP0496 | Ybgc thioesterase |
| HP0497 | Predicted sodium- and chloride-dependent transporter | NA(+):neurotransmitter symporter (SNF FAMILY) |
| HP0498 | Predicted sodium- and chloride-dependent transporter | Transporter |
| HP0499 | Outer membrane phospholipase A1 precursor | Outer membrane phospholipase A |
| HP0500 | Predicted DNA polymerase III beta subunit | Dna polymerase iii beta subunit |
| HP0501 | Predicted DNA gyrase subunit B | DNA gyrase B |
| HP0502 | Predicted coding region HP0502 with no homologue in the databases | Type I restriction enzyme EcoKI M protein |
| HP0503 | Predicted coding region HP0503 with no homologue in the databases | Adenine-N6-DNA-methyltransferase TAQI |
| HP0504 | Predicted coding region HP0504 | Restriction endonuclease |
| HP0505 | Predicted coding region HP0505 | Restriction endonuclease pabi |
| HP0506 | Predicted outer membrane protein | Zinc peptidase |
| HP0507 | Predicted coding region HP0507 | Uridine diphosphate glucose pyrophosphatase. |
| HP0508 | Predicted coding region HP0508 | Alpha-2-macroglobulin receptor-associated protein |
| HP0509 | Predicted glycolate oxidase | oxidoreductase |
| HP0510 | Predicted dihydrodipicolinate reductase | Dihydrodipicolinate reductase |
| HP0511 | Predicted lipoprotein Lpp, an urease enhancing factor | urease-enhancing factor |
| HP0512 | Predicted glutamine synthetase | Glutamine synthetase |
| HP0513 | Predicted coding region HP0513 | Cullin-associated nedd8-dissociated protein |
| HP0514 | Predicted ribosomal protein L9 | Ribosomal protein L9 |
| HP0515 | Predicted heat shock protein, protease subunit | ATP-dependent protease HSLV |
| HP0516 | Predicted heat shock protein, ATP-binding subunit | Heat shock protein HSLV |
| HP0517 | Predicted GTP-binding protein | GTP-binding protein |
| HP0518 | Predicted coding region HP0518 | Putative transpeptidase |
| HP0519 | Predicted coding region HP0519 | Helicobacter cysteine rich protein |
| HP0520 | cag pathogenicity island protein 1 | |  | cag pathogenicity island protein 1 | | --- | --- | |
| HP0521 | cag pathogenicity island protein 2 | cag 2 |
| HP0522 | cag pathogenicity island protein 3 | cag pathogenicity island protein (cag3) |
| HP0523 | cag pathogenicity island protein 4 | cag pathogenicity island protein (cag4) |
| HP0524 | cag pathogenicity island protein 5 | Cag -ALPHA |
| HP0525 | cag pathogenicity island encoded | Cag-Z |
| HP0526 | cag pathogenicity island protein Z | Cag -Z |
| HP0527 | cag pathogenicity island protein Y/DNA transport pore protein | Cag pathogenicity island protein (cag7) |
| HP0528 | cag pathogenicity island protein X | VirB9/CagX/TrbG, a component of the type IV secretion system |
| HP0529 | cag pathogenicity island protein W | Cag pathogenicity island protein w |
| HP0530 | cag pathogenicity island protein V | Cag pathogenicity island protein v/type iv secretion system protein virb8 |
| HP0531 | cag pathogenicity island protein U | Cag pathogenicity island protein (cag11) |
| HP0532 | cag pathogenicity island protein T | Cag pathogenicity island protein (cag12) |
| HP0533 | Predicted coding region HP0533 with no homologue in the databases | 29 aa |
| HP0534 | cag pathogenicity island protein S | Cag pathogenicity island protein 13 |
| HP0535 | cag pathogenicity island protein Q | Cag pathogenicity island protein Q |
| HP0536 | cag pathogenicity island protein P | CagP protein |
| HP0537 | cag pathogenicity island protein M | Cag pathogenicity island protein (cag16) |
| HP0538 | cag pathogenicity island protein N | Cag pathogenicity island protein (cag17) |
| HP0539 | cag pathogenicity island protein L | Cag pathogenicity island protein (cag18) |
| HP0540 | cag pathogenicity island protein I | Cag pathogenicity island protein i/vinculin isoform 1 |
| HP0541 | cag pathogenicity island protein H | |  | Cag pathogenicity island protein (cag20) | | --- | --- | |
| HP0542 | cag pathogenicity island protein G | Cag pathogenicity island protein (cag21) |
| HP0543 | cag pathogenicity island protein F | Cag pathogenicity island protein (cag22) |
| HP0544 | cag pathogenicity island protein E | Cag pathogenicity island protein e |
| HP0545 | cag pathogenicity island protein D | Cag pathogenicity island protein d |
| HP0546 | cag pathogenicity island protein C | Cag pathogenicity island protein C |
| HP0547 | cag pathogenicity island protein A, immunodominant antigen | Cag pathogenicity island protein (cag26) |
| HP0548 | Predicted DNA helicase | Cag pathogenicity island protein |
| HP0549 | Predicted glutamate racemase | Glutamate racemase |
| HP0550 | Predicted transcription termination factor Rho | Transcription termination factor RHO |
| HP0551 | Predicted ribosomal protein L31 | 50S ribosomal protein L31 |
| HP0552 | Predicted coding region HP0552 | Predicted methyltransferase |
| HP0553 | Predicted 23S rRNA methyltransferase | rRNA methyltransferase YJFH |
| HP0554 | Predicted coding region HP0554 with no homologue in the databases | Clp gene regulator (Transcription Activator) |
| HP0555 | Predicted coding region HP0555 | SNF1-like protein kinase ssp2 |
| HP0556 | Predicted coding region HP0556 with no homologue in the databases | Di-or tripeptide H+ symporter |
| HP0557 | Predicted acetyl-coenzyme A carboxylase A subunit | Acetyl-coa carboxylase |
| HP0558 | Predicted 3-oxoacyl-[acyl-carrier-protein] synthase II | 3-oxoacyl-(acyl-carrier protein) synthase ii |
| HP0559 | Predicted acyl carrier protein | Acyl carrier protein |
| HP0560 | Predicted coding region HP0560 with no homologue in the databases | 20aa |
| HP0561 | Predicted 3-ketoacyl-acyl carrier protein reductase | 3-oxoacyl-[acyl-carrier-protein] reductase |
| HP0562 | Predicted ribosomal protein S21 | 30s ribosomal protein s21 |
| HP0563 | Predicted coding region HP0563 | MaoC-like dehydratase |
| HP0564 | Predicted coding region HP0564 | Gene regulation |
| HP0565 | Predicted coding region HP0565 | inner membrane protein ykgB |
| HP0566 | Predicted diaminopimelate epimerase | Diaminopimelate epimerase |
| HP0567 | Predicted outer membrane protein | |  | Transportin-1 | | --- | --- | |
| HP0568 | Predicted coding region HP0568 | Molybdenum cofactor biosynthesis protein |
| HP0569 | Predicted GTP-binding protein | GTP-binding protein |
| HP0570 | Predicted aminopeptidase A/I | Cytosol aminopeptidase |
| HP0571 | Predicted integral membrane protein | 192 aa |
| HP0572 | Predicted adenine phosphoribosyltransferase | Adenine phosphoribosyltransferase |
| HP0573 | Predicted coding region HP0573 | 110 aa |
| HP0574 | Predicted ribose 5-phosphate isomerase | RIBOSE-5-PHOSPHATE ISOMERASE |
| HP0575 | Predicted membrane protein | Putative zinc metalloprotease MJ0392 |
| HP0576 | Predicted signal peptidase I | Predicted signal peptidase i |
| HP0577 | Predicted methylene-tetrahydrofolate dehydrogenase | Bifunctional 5,10, methylene-tetrahydropholate dehydrogenase |
| HP0578 | Predicted coding region HP0578 with no homologue in the databases | Processed glycerol phosphate lipoteichoic acid synthase |
| HP0579 | Predicted coding region HP0579 with no homologue in the databases | Cell division protein kinase, Growth Factor |
| HP0580 | Predicted coding region HP0580 with no homologue in the databases | Bacterial sialidase |
| HP0581 | Predicted dihydroorotase | Dihydroorotase |
| HP0582 | Predicted siderophore-mediated iron transport protein | glycoside hydrolase domain |
| HP0583 | Predicted coding region HP0583 with no homologue in the databases | TYPE IV pilus biogenesis and competence protein |
| HP0584 | Predicted flagellar motor switch protein | putative flagellar motor switch protein |
| HP0585 | Predicted endonuclease III | Endonuclease iii |
| HP0585.1 | Predicted coding region HP0585.1 | 76 aa |
| HP0586 | Predicted coding region HP0586 with no homologue in the databases | Dna binding protein |
| HP0587 | Predicted aminodeoxychorismate lyase | Predicted aminodeoxychorismate lyase |
| HP0588 | OorD subunit of the 2-oxoglutarate oxidoreductase involved in the TCA cycle | predicted aminodeoxychorismate lyase |
| HP0589 | OorA subunit of the 2-oxoglutarate oxidoreductase involved in the TCA cycle | Pyruvate-ferredoxin oxidoreductase |
| HP0590 | OorB subunit of the 2-oxoglutarate oxidoreductase involved in the TCA cycle | Pyruvate-ferredoxin oxidoreductase |
| HP0591 | OorC subunit of the 2-oxoglutarate oxidoreductase involved in the TCA cycle | Oorc subunit of the 2-oxoglutarate oxidoreductase involved in the tca cycle |
| HP0592 | Predicted type III restriction enzyme R protein | DNA repair protein |
| HP0593 | Predicted type III adenine specific DNA methyltransferase | Adenine-specific Methyltransferase MboIIA |
| HP0594 | Predicted coding region HP0594 | Membrane Protein/transport Protein |
| HP0595 | Predicted DsbB-like protein | Disulfide bond formation protein B |
| HP0596 | Predicted coding region HP0596 with no homologue in the databases | Tnf-alpha inducer protein/immune system |
| HP0597 | Predicted penicillin-binding protein 1 | Penicillin-binding protein 1b |
| HP0598 | Predicted 8-amino-7-oxononanoate | Predicted 8-amino-7-oxononanoate synthase |
| HP0599 | Predicted methyl-accepting chemotaxis transmembrane sensory protein (MCP-like protein | |  | Methyl-accepting chemotaxis protein | | --- | --- | |
| HP0600 | Predicted multidrug resistance protein | |  | |  | Multidrug resistance protein pgp-1 | | --- | --- | | | --- | --- | --- | --- | |
| HP0601 | Flagellin A | Flagellin a |
| HP0602 | Novel 3-methyladenine DNA glycosylase involved in base excision repair | 3-methyladenine dna glycosylase |
| HP0603 | Predicted coding region HP0603 with no homologue in the databases | Outer membrane protein H1 |
| HP0604 | Predicted uroporphyrinogen decarboxylase | uroporphyrinogen decarboxylase |
| HP0605 | Outer-membrane protein of the hefABC efflux system | |  | Cation efflux system protein cusC | | --- | --- | |
| HP0606 | Membrane fusion protein of the hefABC efflux system | Multidrug resistance protein |
| HP0607 | Cytoplasmic pump protein of the hefABC efflux system | Cation efflux system protein |
| HP0608 | Predicted coding region HP0608 | outer membrane beta-barrel domain protein |
| HP0609 | Predicted toxin-like outer membrane protein/vacuolating cytotoxin (VacA) paralogue | |  | putative pyrophosphatase | | --- | --- | |
| HP0610 | Predicted toxin-like outer membrane protein/vacuolating cytotoxin (VacA) paralogue | RNA Binding Protein |
| HP0611 | Predicted ABC transporter, permease | voltage dependent potassium channel |
| HP0612 | Predicted ABC transporter, permease | Signal transducer and activator of transcription |
| HP0613 | Predicted ABC transporter, ATP-binding protein | ABC transporter, ATP-binding protein |
| HP0614 | Predicted coding region HP0614 with no homologue in the databases | Putative nickel-responsive regulator |
| HP0615 | Predicted DNA ligase | Dna ligase |
| HP0616 | Predicted chemotaxis protein/CheA-MCP interaction modulator | Response regulator |
| HP0617 | Predicted aspartyl-tRNA synthetase | Aspartyl-trna synthetase |
| HP0618 | Predicted adenylate kinase | Adenylate kinase 5 |
| HP0619 | Predicted LPS biosynthesis protein | 351 aa |
| HP0620 | Inorganic pyrophosphatase controlling the cellular levels of inorganic pyrophosphate | Inorganic pyrophosphatase |
| HP0621 | Predicted DNA mismatch repair protein | Dna mismatch repair protein muts |
| HP0622 | Predicted coding region HP0622 with no homologue in the databases | Cytochrome c oxidase polypeptide 6a2 |
| HP0623 | Predicted UDP-N-acetylmuramate-alanine ligase | Udp-n-acetylmuramate--alanine ligase |
| HP0624 | Predicted aminotransferase | Aminotransferase aspb |
| HP0625 | Predicted 1-hydroxy-2-methyl-2-(E)-butenyl 4-diphosphate synthetase involved in isoprenoid biosynthesis | 4-hydroxy-3-methylbut-2-en-1-yl diphosphate synthase |
| HP0626 | Predicted tetrahydrodipicolinate (THDP) N-succinyltransferase involved involved in diaminopimelate and lysine biosynthesis | Putative 2,3,4,5-tetrahydropyridine-2-carboxylate |
| HP0627 | Cysteine-rich protein F | |  | cysteine-rich protein F | | --- | --- | |
| HP0628 | Cysteine-rich protein F | |  | cysteine-rich protein F | | --- | --- | |
| HP0629 | Predicted coding region HP0629 | Dna binding protein/fanconi anemia group i protein homolog |
| HP0630 | Predicted modulator of drug activity | MODULATOR OF DRUG ACTIVITY B |
| HP0631 | Structural gene for the small subunit of hydrogen uptake-like hydrogenase | PERIPLASMIC [NIFE] HYDROGENASE SMALL SUBUNIT |
| HP0632 | Structural gene for the large subunit of hydrogen uptake-like hydrogenase | MEMBRANE-BOUND HYDROGENASE LARGE SUBUNIT |
| HP0633 | Structural gene for the cytochrome b subunit of hydrogen uptake-like hydrogenase | Ni/Fe-hydrogenase 1 B-type cytochrome subunit |
| HP0634 | Predicted endoprotease involved hydrogenase maturation | Hydrogenase 2 maturation protease |
| HP0635 | Predicted coding region HP0635 | Hydrogenase maturation factor |
| HP0636 | Predicted coding region HP0636 with no homologue in the databases | Growth arrest-specific protein 7 |
| HP0637 | Predicted coding region HP0637 with no homologue in the databases | Sec1 family domain containing protein 1 |
| HP0638 | Proinflamatory outer membrane protein | outer membrane protein HopH |
| HP0639 | Predicted ExsB trans-regulatory protein | Queuosine biosynthesis protein quec |
| HP0640 | Predicted poly(A) polymerase | Poly a polymerase |
| HP0641 | Predicted coding region HP0641 | Multifunctional cyclase-dehydratase-3-O-methytransferase |
| HP0642 | NAD(P)H-flavin oxidoreductase | NADPH-flavin oxidoreductase |
| HP0643 | Predicted glutamyl-tRNA synthetase | Glutamyl-trna synthetase |
| HP0644 | Predicted integral membrane protein | 30S ribosomal protein S8 |
| HP0645 | Predicted soluble lytic murein transglycosylase | Protein (soluble lytic transglycosylase slt70) |
| HP0646 | Predicted UDP-glucose pyrophosphorylase | Predicted udp-glucose pyrophosphorylase |
| HP0647 | Predicted coding region HP0647 with no homologue in the databases | endocytosis, cell motility and morphogenesis |
| HP0648 | Predicted UDP-N-acetylglucosamine enolpyruvyl transferase | UDP-N-acetylglucosamine enolpyruvyltransferase |
| HP0649 | Predicted aspartate ammonia-lyase | L-aspartate ammonia-lyase |
| HP0650 | Predicetd coding region HP0650 with no homologue in the databases | Uracil-dna glycosylase |
| HP0651 | Alpha1,3-fucosyltransferase | Alpha1,3-fucosyltransferase |
| HP0652 | Predicted phosphoserine phosphatase | Phosphoserine phosphatase |
| HP0653 | Nonheme iron-containing ferritin designed to store nonheme iron | Ferritin |
| HP0654 | Predicted coding region HP0654 | Biotin synthetase/adomet binding protein |
| HP0655 | Predicted outer membrane protein | Outer membrane protein assembly |
| HP0656 | Predicted coding region HP0656 | Biotin synthase/transferase |
| HP0657 | Predicted processing zinc-metalloprotease | Putative zinc protease |
| HP0658 | Predicted Glu-tRNA(Gln) amidotransferase subunit B involved in tRNA-dependent transformation of misacylated Glu-tRNAGln | Glutamyl-tRNA (gln) amidotransferase subunit a |
| HP0659 | Predicetd coding region HP0659 with no homologue in the databases | Possible periplasmic protein/chaperone |
| HP0660 | Predicted coding region HP0660 | Chaperone |
| HP0661 | Predicted ribonuclease H | Ribonuclease h |
| HP0662 | Predicted ribonuclease III | Ribonuclease iii |
| HP0663 | Predicted chorismate synthase | Chorismate synthase |
| HP0664 | Predicted coding region HP0664 | 171 aa |
| HP0665 | Predicted oxygen-independent coproporphyrinogen III oxidase | Oxygen-independent coproporphyrinogen iii oxidase |
| HP0666 | Predicted anaerobic glycerol-3-phosphate dehydrogenase | Acetyl-coa decarboxylase/synthase alpha subunit |
| HP0667 | Remnant of type I restriction-modification polypeptide | Holliday-junction resolvase |
| HP0668 | Remnant of type I restriction-modification polypeptide | motor subunit of type I restriction-modification complex |
| HP0669 | Remnant of type I restriction-modification polypeptide | Type I restriction enzyme StySJI M protein |
| HP0670 | Predicted coding region HP0670 with no homologue in the databases | Cathelicidin antimicrobial peptide |
| HP0671 | Predicted outer membrane protein HorF | outer membrane protein HorF |
| HP0672 | Predicted aspartate aminotransferase | Aspartate aminotransferase |
| HP0673 | Predicted coding region HP0673 | Protein disulfide-isomerase/chaperone |
| HP0674 | Predicted coding region HP0674 | Lysozyme |
| HP0675 | Predicted integrase/recombinase | Integrase |
| HP0676 | Predicted methylated-DNA-protein-cysteine methyltransferase | Methylated-DNA--protein-cysteine |
| HP0677 | Predicted integral membrane protein | TRNA (guanine-N(7)-)-methyltransferase |
| HP0678 | Predicted lipopolysaccharide biosynthesis protein | putative lipopolysaccharide biosynthesis protein |
| HP0679 | Predicted lipopolysaccharide biosynthesis protein | Lipopolysaccaride biosynthesis protein WBPB |
| HP0680 | Predicted ribonucleoside-diphosphate reductase 1 alpha subunit | ribonucleotide-diphosphate reductase subunit alpha |
| HP0681 | Predicted coding region HP0681 | 40S ribosomal protein S11 |
| HP0682 | Predicted coding region HP0682 | |  | DNA replication initiator | | --- | --- | |
| HP0683 | Predicted UDP-N-acetylglucosamine pyrophosphorylase | Glucosamine-1-phosphate acetyltransferase |
| HP0684 | Flagellar biosynthetic protein | |  | flagellar biosynthesis protein FliP | | --- | --- | |
| HP0685 | Flagellar biosynthetic protein | |  | flagellar biosynthesis protein FliP | | --- | --- | |
| HP0686 | Predicted iron(III) dicitrate transport protein | |  | Iron(III) dicitrate transport protein fecA | | --- | --- | |
| HP0687 | Predicted iron(II) (ferous iron) transport protein | |  | Ferrous iron transport protein b  Homolog | | --- | --- | |
| HP0688 | Predicted coding region HP0688 | Dna polymerase |
| HP0689 | Predicted coding region HP0689 | DNA polymerase subunit gamma-1 |
| HP0690 | Predicted acetyl coenzyme A acetyltransferase | Acetyl-coenzyme a acetyltransferase 2 |
| HP0691 | Succinyl-CoA-transferase subunit A of the succinyl-coenzyme A (CoA):acetoacetyl-CoA transferase involved in the TCA cycle | Succinyl-CoA:3-ketoacid-coenzymea transferase |
| HP0692 | Succinyl-CoA-transferase subunit B of the succinyl-coenzyme A (CoA):acetoacetyl-CoA transferase involved in the TCA cycle | Succinyl-CoA:3-ketoacid-coenzymea transferase |
| HP0693 | Predicted short-chain fatty acids transporter | |  | Transporter | | --- | --- | |
| HP0694 | Predicted outer membrane protein | outer membrane protein (LpxR) |
| HP0695 | Predicted N-methylhydantoinase | pantheonate kinase-like protein |
| HP0696 | Predicted N-methylhydantoinase | N-methylhydantoinase |
| HP0697 | Predicted coding region HP0697 | Sigma-E factor negative regulatory protein |
| HP0698 | Predicted coding region HP0698 with no homologue in the databases | Motor Protein/apoptosis |
| HP0699 | Predicted coding region HP0699 with no homologue in the databases | Signaling protein/interferon-induced guanylate-binding protein 1 |
| HP0700 | Predicted diacylglycerol kinase | diacylglycerol kinase |
| HP0701 | Predicted DNA gyrase subunit A | DNA gyrase subunit A |
| HP0702 | Predicted coding region HP0702 with no homologue in the databases | Immune system protein |
| HP0703 | Transcriptional activator of flagella proteins | TRANSCRIPTIONAL REGULATOR (ntrc FAMILY) |
| HP0704 | Predicted coding region HP0704 with no homologue in the databases | 36aa |
| HP0705 | Predicted excinuclease ABC subunit A/damage recognition protein | Excinuclease ABC subunit A |
| HP0706 | Outer membrane protein HopE with porin properties | Outer membrane protein A |
| HP0707 | Predicted coding region HP0707 | S-adenosyl-methyltransferase MRAW |
| HP0708 | Predicted coding region HP0708 | Transcription |
| HP0709 | Predicted coding region HP0709 | 5'-fluoro-5'-deoxyadenosine synthase |
| HP0710 | Predicted outer membrane protein HomA | Outer membrane protein H1 |
| HP0711 | Predicted coding region HP0711 | HD-domain phosphohydrolase |
| HP0712 | Predicted coding region HP0712 | Protein of unknown function with a Fic domain(signaling) |
| HP0713 | Predicted coding region HP0713 | Protein of unknown function with a Fic domain |
| HP0714 | Predicted alternative transcription initiation factor sigma-54 | RNA polymerase sigma factor RpoN   |  |  | | --- | --- | |
| HP0715 | Predicted ABC transporter, ATP-binding protein | ABC transporter |
| HP0716 | Predicted coding region HP0716 | Atpase involved in cell wall synthesis /   |  | | --- | |
| HP0717 | Predicted gamma and tau subunits of the DNA polymerase III holoenzyme needed for DNA sliding clamp and chromosome replication | clamp loader gamma (gamma) complex of E. coli DNA polymerase III. |
| HP0718 | Predicted integral membrane protein | Peroxisomal acyl-COA oxidase |
| HP0719 | Predicted coding region HP0719 with no homologue in the databases | Dna polymerase iii subunit gamma |
| HP0720 | Predicted coding region HP0720 with no homologue in the databases | 16s rRNA processing protein |
| HP0721 | Predicted coding region HP0721 with no homologue in the databases | Anthranilate phosphoribosyltransferase |
| HP0722 | Predicted outer membrane protein HopO | outer membrane protein family protein |
| HP0723 | Predicted L-asparaginase type II | L-asparaginase |
| HP0724 | Predicted anaerobic C4-dicarboxylate transport protein | sodium-dependent dicarboxylate transporter. |
| HP0725 | Predicted outer membrane protein HopP | delta-endotoxin |
| HP0726 | Predicted outer membrane protein | Outer membrane protein oprg |
| HP0727 | Predicted transcriptional regulator | tRNA-dihydrouridine synthase |
| HP0728 | Predicted coding region HP0728 | isoluecyl-tRNA lysidine synthetase |
| HP0729 | Predicted coding region HP0729 | Replicase polyprotein 1AB |
| HP0730 | Predicted coding region HP0730 | Tensin-like C1 domain-containing phosphatase |
| HP0731 | Predicted LeoA required for secretion of heat-labile enterotoxin in E.coli H10407 | Bacterial dynamin-like protein |
| HP0732 | Remnant of ancestral polypeptide of unknown function | hemagglutinin |
| HP0733 | Remnant of ancestral polypeptide of unknown function | |  | Bacterial dynamin-like protein | | --- | --- | |  |  | |
| HP0734 | Predicted coding region HP0734 | methylornithine synthase PylB |
| HP0735 | Predicted xanthine-guanine phosphoribosyl transferase/purine salvage and interconversion pathways | Hypoxanthine phosphoribosyltransferase |
| HP0736 | Predicted aminotransferase | Aspartate aminotransferase |
| HP0737 | Predicted phosphatidylglycerophosphatase A | phosphatidylglycerophosphatase (PGPase), a putative membrane-bound lipid phosphatase, |
| HP0738 | Predicted D-alanine:D-alanine ligase A | D-alanine-d-alanine ligase |
| HP0739 | Predicted coding region HP0739 | Probable hydrolase |
| HP0740 | Predicted UDP-MurNac-pentapeptide synthetase involved in peptidoglycan biosynthesis | UDP-N-acetylmuramoylalanine-D-glutamyl-lysine-D-alanyl-D-alanine ligase |
| HP0741 | Predicted HIT family protein | HIT-like protein involved in cell-cycle regulation |
| HP0742 | Predicted phosphoribosylpyrophosphate synthetase | Phosphoribosyl pyrophosphate synthetase |
| HP0743 | Predicted rod shape-determining protein | Predicted rod shape-determining protein |
| HP0744 | Predicted coding region HP0744 with no homologue in the databases | 50S ribosome-binding GTPase family protein |
| HP0745 | Predicted pseudouridine synthase D involved in 23S rRNA base modifications | Ribosomal large subunit pseudouridine synthase D |
| HP0746 | Predicted coding region HP0746 | Cell adhesion protein/fibronectin |
| HP0747 | Predicted S-adenosylmethionine-dependent methyltransferase | S-adenosylmethionine-dependent methyltransferase |
| HP0748 | Predicted cell division protein/ABC transporter, ATP-binding protein | MJ0796 ATP-binding cassette, macrolide-specific ABC-type efflux carrier (MacAB), and proteins involved in cell division (FtsE),and release of liporoteins from the cytoplasmic membrane(LolCDE). |
| HP0749 | Predicted cell division membrane protein | cell division membrane protein |
| HP0750 | Predicted coding region HP0750 | Putative peptidase m, Contractile Protein |
| HP0751 | Predicted polar flagellin | flagellar protein FlaG |
| HP0752 | Predicted flagellar hook-associated protein 2 | flagellar capping protein |
| HP0753 | Predicted flagellar protein | Flagellar protein |
| HP0754 | Predicted coding region HP0754 with no homologue in the databases | Suppressor protein |
| HP0755 | Predicted moeB/thiF family protein | Molybdopterin biosynthesis moeb protein |
| HP0756 | Predicted coding region HP0756 with no homologue in the databases | 48aa |
| HP0757 | Predicted carbon-nitrogen hydrolase | Hydrolase, carbon-nitrogen family |
| HP0758 | Predicted integral membrane protein | vesicular transport factor |
| HP0759 | Predicted DNA-damage induced multidrug efflux protein | Multi antimicrobial extrusion protein (Na(+)/drug antiporter) MATE-like MDR efflux pump |
| HP0760 | Predicted coding region HP0760 | Putative phosphohydrolase |
| HP0761 | Predicted coding region HP0761 | 5-formyltetrahydrofolate cyclo-ligase family protein |
| HP0762 | Predicted coding region HP0762 | Putative thioredoxin |
| HP0763 | Predicted signal recognition particule receptor | SIGNAL RECOGNITION PARTICLE PROTEIN |
| HP0764 | Remnant of ancestral polypeptide of unknown function | Fancl(Ligase) |
| HP0765 | Remnant of ancestral polypeptide of unknown function | Srp19( RNA Binding Protein) |
| HP0766 | Predicted coding region HP0766 with no homologue in the databases | COLICIN A |
| HP0767 | Predicted coding region HP0767 with no homologue in the databases | Molybdenum cofactor biosynthesis protein A |
| HP0768 | Predicted molybdenum cofactor biosynthesis protein A/precursor Z synthesis | Molybdenum cofactor biosynthesis protein a |
| HP0769 | Predicted molybdopterin-guanine dinucleotide biosynthesis protein A | Molybdopterin-guanine dinucleotide biosynthesis protein |
| HP0770 | Flagellar biosynthesis and export protein/adhesion to and colonisation of the gastric mucosa | |  | Flagellar biosynthetic protein | | --- | --- | |
| HP0771 | Predicted coding region HP0771 with no homologue in the databases | N-acetylmuramoyl-l-alanine amidase |
| HP0772 | Predicted N-acetylmuramoyl-L-alanine amidase | N-acetylmuramoyl-l-alanine amidase |
| HP0773 | Predicted coding region HP0773 | Putative nitroalkan dioxygenase |
| HP0774 | Predicted tyrosyl-tRNA synthetase | Tyrosyl-trna synthetase |
| HP0775 | Predicted bifunctional ppGpp-3'-pyrophosphohydrolase/ppGpp synthetase II | bifunctional ppgpp-3'-pyrophosphohydrolase/ppgpp synthetase ii |
| HP0776 | Predicted RNA polymerase omega chain, a transcriptional activator | DNA-directed RNA polymerase subunit omega |
| HP0777 | Predicted uridine 5'-monophosphate kinase | Uridylate kinase |
| HP0778 | Predicted coding region HP0778 | Menaquinone biosynthetic enzyme |
| HP0779 | Predicted aconitase B | Aconitate hydratase 2 |
| HP0780 | Predicted coding region HP0780 with no homologue in the databases | Signaling Protein |
| HP0781 | Predicted coding region HP0781 | Leu/ile/val-binding protein/transport protein |
| HP0782 | Predicted outermembrane protein HofE | Glycine-glutamate dipeptide porin |
| HP0783 | Predicted coding region HP0783 with no homologue in the databases | 165aa |
| HP0784 | Predicted coding region HP0784 with no homologue in the databases | 44aa |
| HP0785 | Predicted carrier protein involved in outer membrane lipoprotein translocation from the inner membrane | Outer-membrane lipoprotein carrier protein |
| HP0786 | Predicted preprotein translocase subunit | Protein translocase subunit Sec A |
| HP0787 | Predicted transmembrane protein involved in lipoprotein release from the inner membrane prior to translocation to the outer membrane | Macrolide export ATP-binding/permease protein |
| HP0788 | Predicted outer membrane protein HofF | Predicted outer membrane protein ,porin |
| HP0789 | Predicted coding region HP0789 with no homologue in the databases | Molybdenum cofactor biosynthesis protein C |
| HP0790 | Predicted specificity subunit S of type I restriction-modification system | Type I restriction-modification enzyme, S subunit |
| HP0791 | Cadmium-transporting ATPase/P-type transporting ATPase involved in transition metal resistance | Copper efflux ATPase   |  | | --- | |
| HP0792 | Predicted DNA transformation competence protein probably involved in recombination | Putative protease La homolog |
| HP0793 | Predicted polypeptide deformylase | Peptide deformylase |
| HP0794 | Predicted ATP-dependent Clp protease proteolytic subunit | Atp-dependent clp protease proteolytic subunit |
| HP0795 | Predicted trigger factor | trigger factor |
| HP0796 | Predicted outer membrane protein HorG | Outer membrane protein A |
| HP0797 | Neuraminyllactose-binding hemagglutinin/flagella sheath lipoprotein, protects the flagella | neuraminyllactose-binding hemagglutinin homolog |
| HP0798 | Predicted molybdenum cofactor biosynthesis protein C | Molybdopterin biosynthesis |
| HP0799 | Predicted molybdopterin biosynthesis protein | Molybdenum cofactor biosynthesis protein mog |
| HP0800 | Predicted molybdopterin converting factor subunit 2 | Molybdopterin synthase catalytic subunit/ |
| HP0801 | Predicted molybdopterin converting factor subunit 1 | Molybdopterin converting factor subunit 1 |
| HP0802 | GTP-cyclohydrolase II, first step of riboflavin biosynthesis | Gtp cyclohydrolase ii |
| HP0803 | Predicted coding region HP0803 | Isoprenylcysteine carboxyl methyltransferase |
| HP0804 | Bifunctional 3,4-dihydroxy-2-butanone 4-phosphate (DHBP) synthase/GTP cyclohydrolase II involved in riboflavin biosynthesis | 3,4-dihydroxy-2-butanone 4-phosphate synthase |
| HP0805 | Predicted LPS biosynthesis protein | Beta-1,3-n-acetylglucosaminyltransferase |
| HP0806 | Predicted coding region HP0806 | Putative Metal-dependent Hydrolase |
| HP0807 | Predicted iron(III) dicitrate transport protein | Iron(iii) dicitrate transport protein |
| HP0808 | Predicted holo-acyl carrier protein synthase | Holo-[acyl-carrier-protein] synthase |
| HP0809 | Predicted flagellar biosynthesis protein | flagellar basal body protein FliL |
| HP0810 | Predicted N-6 adenine methyltransferase involved in DNA modification | Methyltransferase |
| HP0811 | Predicted coding region HP0811 | Transcription Regulator |
| HP0812 | Predicted coding region HP0812 | S-adenosyl-L-methionine-dependent methyltransferase family protein |
| HP0813 | Predicted coding region HP0813 | Hydroxyacylglutathione hydrolase |
| HP0814 | Predicted activator of MoaD/ThiS sulfur donor proteins involved in molybdopterin/thiamine biosynthetic pathways | Molybdopterin biosynthesis moeb protein |
| HP0815 | Predicted flagellar motor rotation protein | flagellar motor protein MotA |
| HP0816 | Flagellar motor rotation protein | Chemotaxis protein motb |
| HP0817 | Predicted coding region HP0817 with no homologue in the databases | Outer protein YOPM (a leucine-rich effector protein) |
| HP0818 | Predicted betaine/proline/choline ABC transporter transmembrane component | Glycine betaine/carnitine/choline abc transporter |
| HP0819 | Predicted osmoprotection ABC transporter/ATP-binding protein involved in glycine betaine/L-proline transport | Molybdate/tungstate ABC transporter, ATP-bind ing |
| HP0820 | Predicted coding region HP0820 with no homologue in the databases | Receptor protein |
| HP0821 | Predicted excinuclease ABC subunit C/nicks DNA 5' to the damage site | Uvrabc system protein |
| HP0822 | Predicted homoserine dehydrogenase | Homoserine dehydrogenase |
| HP0823 | Predicted coding region HP0823 | rpa0323 protein of unknown function |
| HP0824 | Thioredoxin | Thioredoxin-2 |
| HP0825 | Thioredoxin reductase | Thioredoxin reductase |
| HP0826 | Beta-4-galactosyltransferase | Beta-1,3-n-acetylglucosaminyltransferase |
| HP0827 | Predicted RNA binding protein | Ss-DNA binding protein 12RNP2 |
| HP0828 | Predicted ATP synthase F0, subunit A | Nadh-quinone oxidoreductase subunit / A1c12 subcomplex of f1fo atp synthase(membrane protein) |
| HP0829 | Predicted inosine-5'-monophosphate dehydrogenase involved in de novo synthesis of guanosine nucleotides | Inosine-5'-monophosphate dehydrogenase |
| HP0830 | Predicted Glu-tRNA(Gln) amidotransferase subunit A involved in tRNA-dependent transformation of misacylated Glu-tRNAGln |  Glutamyl-tRNA(gln) amidotransferase subunit A |
| HP0831 | Predicted dephospho-CoA kinase involved in coenzyme A biosynthesis | Dephospho-CoA kinase involved in coenzyme A biosynthesis |
| HP0832 | Predicted spermidine synthase | Spermidine synthase |
| HP0833 | Predicted coding region HP0833 with no homologue in the databases | Glr4197 protein(proton-gated ion channel) |
| HP0834 | Predicted GTP-binding protein | Probable GTP-binding protein ENGA |
| HP0835 | Predicted histone-like DNA-binding protein HU | DNA-binding protein |
| HP0836 | Predicted coding region HP0836 | 3,7-dimethylxanthine methyltransferase |
| HP0837 | Predicted coding region HP0837 | antilipopolysaccharide factor |
| HP0838 | Predicted coding region HP0838 with no homologue in the databases | TNF receptor |
| HP0839 | Predicted outer membrane protein P1 | Probable outer membrane protein |
| HP0840 | Bifunctional UDP-GlcNAc C6 dehydratase/C4 reductase involved in the first step of UDP-QuiNAc production | UDP-glcnac C6 dehydratase |
| HP0841 | Bifunctional phosphopantothenoylcysteine decarboxylase/phosphopantothenate synthase | phosphopantothenoylcysteine decarboxylase /phosphopantothenate--cysteine ligase |
| HP0842 | Predicted coding region HP0842 with no homologue in the databases | CG4244-PB(Signaling Protein) |
| HP0843 | Predicted thiamin phosphate pyrophosphorylase | Thiamin phosphate synthase |
| HP0844 | Predicted phosphomethylpyrimidine kinase | Phosphomethylpyrimidine kinase |
| HP0845 | Predicted hyroxyethylthiazole kinase |  Hydroxyethylthiazole kinase |
| HP0846 | Predicted type I restriction enzyme R protein | Type I restriction enzyme ecor124ii r protein |
| HP0847 | Predicted coding region HP0847 with no homologue in the databases | polyguluronate lyase |
| HP0848 | Predicted type I restriction enzyme/specificity protein | Type i restriction-modification enzyme(Hydrolase regulator) |
| HP0849 | Predicted type I restriction enzyme/specificity protein | type i restriction-modification enzymes |
| HP0850 | Predicted type I restriction enzyme modification protein | Type I restriction-modification system |
| HP0851 | Predicted integral membrane protein | Class A nonspecific acid phosphatase PhoN |
| HP0852 | Predicted coding region HP0852 | uncharacterized protein SPO1766 |
| HP0853 | Predicted ABC transporter, ATP-binding protein | Putative ABC transporter atp-binding protein TM_0 |
| HP0854 | Predicted guanosine 5'-monophosphate oxidoreductase in purine nucleotides salvage | GMP reductase |
| HP0855 | Predicted alginate O-acetylation protein | iron superoxide dismutase |
| HP0856 | Predicted coding region HP0856 | D-alanyl-lipoteichoic acid synthetase |
| HP0857 | Predicted phosphoheptose isomerase | Phosphoheptose isomerase 1 |
| HP0858 | Predicted ADP-heptose synthase | D-beta-D-heptose 7-phosphate kinase |
| HP0859 | Predicted ADP-L-glycero-D-mannoheptose-6-epimerase | ADP-L-glycero-D-mannoheptose-6-epimerase |
| HP0860 | Predicted coding region HP0860 | D,D-heptose 1,7-bisphosphate phosphatase |
| HP0861 | Predicted coding region HP0861 | Paddle chimera voltage gated potassium channel |
| HP0862 | Predicted coding region HP0862 | Transcriptional activator |
| HP0863 | Predicted coding region HP0863 with no homologue in the databases | β-barrel Outer Membrane Proteins |
| HP0864 | Predicted coding region HP0864 | Response regulator aspartate phosphatase H |
| HP0865 | Predicted deoxyuridine 5'-triphosphate nucleotidohydrolase | Deoxyuridine 5'-triphosphate nucleotidohydrolase |
| HP0866 | Predicted transcription elongation factor | Transcription elongation factor GREB |
| HP0867 | Predicted lipid A disaccharide synthetase | UDP-N-acetylglucosamine-n-acetylmuramyl(membrane-associated glycosyltransferase involved in peptidoglycan biosynthesis) |
| HP0868 | Predicted coding region HP0868 with no homologue in the databases | COLICIN E3 |
| HP0869 | HypA, protein involved in the maturation and incorporation of nickel ions in the hydrogenase and urease catalytic site | Hydrogenase,urease nickel incorporation protein/ |
| HP0870 | Flagellar hook protein | Flagellar hook protein flgE |
| HP0871 | Predicted CDP-diglyceride hydrolase | CDP-diacylglycerol pyrophosphatase |
| HP0872 | Predicted alkylphosphonate uptake protein | phnA-like protein pa0128 |
| HP0873 | Predicted coding region HP0873 with no homologue in the databases | SUMO-modified proliferating cell nuclear antigen(proliferating cell nuclear antigen) |
| HP0874 | Predicted coding region HP0874 with no homologue in the databases | Transglutaminase-like enzymes, putative cysteine protease |
| HP0875 | Catalase involved in detoxification of hydrogen peroxyde | KATA catalase |
| HP0876 | Iron-regulated outer membrane protein | Outer membrane heme receptor ShuA |
| HP0877 | Predicted Holliday junction endodeoxyribonuclease | Holliday junction resolvase (RUVC) |
| HP0878 | Predicted coding region HP0878 | Effector protein HopAB2 (kinase-interacting domains) |
| HP0879 | Predicted coding region HP0879 | protein of unknown function vpa0982 |
| HP0880 | Predicted coding region HP0880 with no homologue in the databases | DNA-directed rna polymerase iii subunit RPC3 |
| HP0881 | Predicted coding region HP0881 with no homologue in the databases | 31aa |
| HP0882 | Predicted coding region HP0882 | 118aa |
| HP0883 | Predicted Holliday junction DNA | Holliday junction DNA helicase RUVA |
| HP0884 | Predicted coding region HP0884 | 614 aa |
| HP0885 | Predicted virulence factor mviN protein | |  | Multi antimicrobial extrusion protein | | --- | --- | |
| HP0886 | Predicted cysteinyl-tRNA synthetase | Cysteinyl-tRNA synthetase |
| HP0887 | VacA cytotoxin | Vacuolating cytotoxin |
| HP0888 | iron(III) dicitrate ABC transporter, ATP-binding protein (fecE) | ABC transporter, ATP binding protein |
| HP0889 | iron(III) dicitrate ABC transporter, permease protein (fecD) | Hypothetical ABC transporter permease protein |
| HP0890 | Predicted short-chain oxidoreductase | 17 beta-hydroxysteroid dehydrogenase |
| HP0891 | Predicted acyl coenzyme A thioesterase | Acyl-CoA hydrolase |
| HP0892 | Predicted coding region HP0892 | Hypothetical protein HP0892 |
| HP0893 | Predicted coding region HP0893 with no homologue in the databases | conserved hypothetical protein |
| HP0894 | Predicted coding region HP0894 | conserved hypothetical protein HP0894 |
| HP0895 | Predicted coding region HP0895 with no homologue in the databases | transcription activator of multidrug-efflux transporter genes |
| HP0896 | Predicted outer membrane protein homologous to an adhesin binding fucosylated Lewis b (Leb) histo-blood group antigen | Outer membrane protein H1 |
| HP0897 | Predicted coding region HP0897 with no homologue in the databases | Mitotic spindle assembly checkpoint protein M |
| HP0898 | HypD protein involved in hydrogenase maturation | HYDROGENASE EXPRESSION/FORMATION |
| HP0899 | Predicted chaperone-like protein involved in hydrogenase maturation |  Hydrogenase assembly chaperone hypC/hupF |
| HP0900 | HypB protein involved in the maturation and incorporation of nickel ions in the hydrogenase and urease catalytic site | Hydrogenase nickel incorporation |
| HP0901 | Predicted coding region HP0901 with no homologue in the databases | 41aa |
| HP0902 | Predicted coding region HP0902 | CUPIN 2, conserved barrel |
| HP0903 | Predicted acetate kinase | Acetate kinase |
| HP0904 | Predicted phosphotransacetylase | Nitrogenase iron protein |
| HP0905 | Predicted phosphotransacetylase | Nitrogenase iron protein-like |
| HP0906 | Predicted coding region HP0906 | Protein transport |
| HP0907 | Predicted hook assembly protein | |  | Flagellar basal-body rod modification protein | | --- | --- | |
| HP0908 | Predicted flagellar hook protein | fragment of the hook protein |
| HP0909 | Non-functional type II restriction endonuclease in a silent state or degenerated enough to be considered as a pseudogene | Adenine-N6-DNA-methyltransferase taqi |
| HP0910 | GTNNAC site-specific type II m6A methylase | Adenine-N6-DNA-methyltransferase TAQI |
| HP0911 | Predicted ATP-dependent single-stranded DNA helicase | DNA helicase II |
| HP0912 | Outer membrane protein HopC/AlpA with porin and adhesin properties | Outer membrane protein H1 |
| HP0913 | Outer membrane protein HopC/AlpA with porin and adhesin properties | outer membrane protein HopB |
| HP0914 | Predicted outer membrane protein HopG | integrin alpha-v(protein binding) |
| HP0915 | Predicted iron-regulated outer membrane protein | Ferric enterobactin receptor (membrane protein) |
| HP0916 | Predicted iron-regulated outer membrane protein | Membrane protein,heme binding protein |
| HP0917 | Predicted coding region HP0917 with no homologue in the databases | 23aa |
| HP0918 | Predicted coding region HP0918 | RNA binding protein |
| HP0919 | Predicted carbamoyl-phosphate synthase, large subunit, glutamine-hydrolysing | Carbamoyl phosphate synthetase |
| HP0920 | Predicted integral membrane protein | Cell adhesion |
| HP0921 | Predicted glyceraldehyde-3-phosphate dehydrogenase | Glyceraldehyde 3-phosphate dehydrogenase |
| HP0922 | Predicted toxin-like outer membrane protein/vacuolating cytotoxin (VacA) paralogue | Hypothetical protein YDCK |
| HP0923 | Predicted outer membrane protein HopK | Outer membrane protein W |
| HP0924 | Predicted 4-oxalocrotonate tautomerase | Probable tautomerase HP_0924 |
| HP0925 | Predicted recombinational DNA repair protein | Recombination protein recR |
| HP0926 | tRNA pseudouridine synthase D | Probable tRNA pseudouridine synthase D |
| HP0927 | Predicted heat shock protein | heat shock protein |
| HP0928 | Predicted GTP cyclohydrolase I | GTP cyclohydrolase I |
| HP0929 | Predicted geranyltranstransferase | Geranyltranstransferase (ISPA) |
| HP0930 | Predicted stationary-phase survival protein | Stationary-phase survival protein sure homolog |
| HP0931 | Predicted coding region HP0931 with no homologue in the databases | RNA-binding protein 40 |
| HP0932 | Predicted coding region HP0932 with no homologue in the databases | Fatty acid synthase |
| HP0933 | Predicted coding region HP0933 | Putative 6-pyruvoyl tetrahydrobiopterin synthase |
| HP0934 | Predicted coding region HP0934 | Pyruvate formate-lyase 1-activating enzyme |
| HP0935 | Predicted coding region HP0935 |  N-acetyltransferase |
| HP0936 | Predicted proline/betaine transporter | D-xylose-proton symporter |
| HP0937 | Remnant of ancestral polypeptide of unknown function | HNH endonuclease |
| HP0938 | Remnant of ancestral polypeptide of unknown function | Protein ADRM1(Protein Binding) |
| HP0939 | Predicted amino acid ABC transporter, permease protein | D-methionine transport system permease protein |
| HP0940 | Predicted amino acid ABC transporter, periplasmic binding protein | Probable abc transporter extracellular-binding |
| HP0941 | Predicted alanine racemase | Alanine racemase |
| HP0942 | Predicted sodium/D-alanine glycine symporter | Transport Protein |
| HP0943 | Predicted D-amino acid dehydrogenase | Glycine oxidase   |  |  | | --- | --- | |
| HP0944 | Predicted regulator of purine biosynthesis | Putative translation initiation inhibitor |
| HP0945 | Predicted coding region HP0945 with no homologue in the databases | hypothetical protein (putative phosphatase) |
| HP0946 | Predicted integral membrane protein | Transporter, NadC family |
| HP0947 | Predicted coding region HP0947 with no homologue in the databases | 120 aa |
| HP0948 | Predicted coding region HP0948 | PROTEASOME REGULATORY SUBUNIT |
| HP0949 | Predicted secreted protein | Hypothetical UPF0247 protein |
| HP0950 | Predicted acetyl-coenzyme A carboxylase carboxyl transferase subunit beta involved in membrane lipids biosynthesis | Acetyl-coenzyme a carboxylase carboxyl transferase |
| HP0951 | Predicted coding region HP0951 | Recombinational repair protein |
| HP0952 | Predicted integral membrane protein | Competence/damage-inducible protein cina |
| HP0953 | Predicted coding region HP0953 with no homologue in the databases | DNA Binding Protein |
| HP0954 | NAD(P)H-dependent nitroreductase involved metronidazole resistance | Putative NAD(P)H nitroreductase YDFN |
| HP0955 | Predicted prolipoprotein diacylglyceryl transferase | HIG1 domain family member |
| HP0956 | Predicted pseudouridine synthase C involved in 23S rRNA base modifications |  Ribosomal large subunit pseudouridine synthase C |
| HP0957 | Predicted 3-deoxy-d-manno-octulosonic-acid transferase | 3-deoxy-d-manno-2-octulosonic acid transferase |
| HP0958 | Predicted coding region HP0958 | Gene regulation, chaperone |
| HP0959 | Predicted coding region HP0959 | 243 aa |
| HP0960 | Predicted glycyl-tRNA synthetase, alpha subunit | Glycyl-trna synthetase alpha chain |
| HP0961 | Predicted glycerol-3-phosphate dehydrogenase involved in membrane lipids biosynthesis | Glycerol-3-phosphate dehydrogenase |
| HP0962 | Predicted acyl carrier protein | Acyl carrier protein |
| HP0963 | Predicted coding region HP0963 with no homologue in the databases | Bacterial dynamin-like protein |
| HP0964 | Remnant pseudogene of an ancestral ATP/GTP-binding protein | Bacterial dynamin-like protein (mechano-chemical gtpases )/ Remnant pseudogene of an ancestral ATP/GTP-binding protein |
| HP0965 | Remnant pseudogene of an ancestral ATP/GTP-binding protein | Bacterial dynamin-like protein (mechano-chemical GTPases )/ |
| HP0966 | Predicted coding region HP0966 | bacterial dynamin-like protein |
| HP0967 | Predicted virulence-associate protein D | |  | Immunoglobulin G-binding protein G,  Virulence-associated protein D | | --- | --- | |
| HP0968 | Predicted coding region HP0968 with no homologue in the databases | 21aa |
| HP0969 | Predicted cobalt-zinc-cadmium resistance protein/cation efflux system protein | Multidrug resistance protein MEXB |
| HP0970 | Predicted cobalt-zinc-cadmium resistance protein/cation efflux system protein | |  | Membrane fusion protein (MFP) heavy metal cation  efflux ZneB (CzcB-like) | | --- | --- | |
| HP0971 | Predicted coding region HP0971with no homologue in the databases | Outer membrane protein |
| HP0972 | Predicted glycyl-tRNA synthetase, beta subunit | |  |  | | --- | --- |   Alanyl-tRNA synthetase |
| HP0973 | Predicted coding region HP0973 with no homologue in the databases | soluble lytic murein transglycosylase |
| HP0974 | Predicted 2,3-bisphosphoglycerate-independent phosphoglycerate mutase | 2,3-bisphosphoglycerate-independent phosphoglycerate mutase |
| HP0975 | Predicted Glu-tRNA(Gln) amidotransferase subunit C involved in tRNA-dependent transformation of misacylated Glu-tRNAGln | Glutamyl-tRNA(gln) amidotransferase(ligase) |
| HP0976 | Predicted adenosylmethionine-8-amino-7-oxononanoate aminotransferase | Adenosylmethionine-8-amino-7-oxononanoate aminotr |
| HP0977 | Predicted pedidyl-prolyl cis-trans isomerase D | Survival protein surA (peptidyl-prolyl isomerases) |
| HP0978 | Predicted cell division protein/septum formation protein | Cell division protein ftsa |
| HP0979 | Predicted GTPase/circumferential ring formation/cell division protein | Cell division protein ftsz |
| HP0980 | Predicted integral membrane protein | putative zinc metalloprotease |
| HP0981 | Predicted exonuclease VII-like, large subunit protein | RNA polymerase, sigma factor |
| HP0982 | Predicted coding region HP0982 with no homologue in the databases | Protein transport |
| HP0983 | Predicted integral membrane protein | Membrane protein |
| HP0984 | Predicted coding region HP0984 with no homologue in the databases | DNA-binding domain of the replication initiator protein |
| HP0985 | Predicted coding region HP0985 with no homologue in the databases | Signaling Protein |
| HP0986 | Predicted coding region HP0986 with no homologue in the databases | 237 aa |
| HP0987 | Predicted coding region HP0987 with no homologue in the databases | Metal-binding protein |
| HP0988 | Predicted IS605 transposase A | transposase |
| HP0989 | Predicted IS605 transposase B | Endonuclease VIII |
| HP0990 | Predicted coding region HP0990 with no homologue in the databases | Lipid Binding Protein |
| HP0991 | Predicted coding region HP0991 with no homologue in the databases | Transferase |
| HP0992 | Predicted coding region HP0992 with no homologue in the databases | Endonuclease Inhibitor |
| HP0993 | Predicted coding region HP0993 with no homologue in the databases | Histone acetyltransferase |
| HP0994 | Predicted coding region HP0994 with no homologue in the databases | Lincosamide nucleotidyltransferase |
| HP0995 | Predicted integrase/recombinase xerCD family | SITE-SPECIFIC RECOMBINASE |
| HP0996 | Predicted coding region HP0996 with no homologue in the databases | Mobilization protein A  (conjugative mobilization between bacterial cells) |
| HP0997 | Predicted IS605 transposase B | Endonuclease VIII |
| HP0998 | Predicted IS605 transposase A | transposase |
| HP0999 | Predicted coding region HP0999 with no homologue in the databases | adenine specific DNA methyltransferase |
| HP1000 | Predicted partitioning protein A | Para family chromosome partitioning protein |
| HP1001 | Predicted coding region HP1001 with no homologue in the databases | Putative nickel-responsive regulator |
| HP1002 | Predicted coding region HP1002 with no homologue in the databases | Cystatin D |
| HP1003 | Predicted coding region HP1003 with no homologue in the databases | Cell adhesion |
| HP1004 | Predicted coding region HP1004 with no homologue in the databases | Mobilization protein |
| HP1005 | Predicted coding region HP1005 with no homologue in the databases | Beta-elicitin cinnamomin |
| HP1006 | Predicted DNA transfer protein | bacterial conjugation protein TrwB resembles ring helicases and F1-ATPase |
| HP1007 | Predicted IS200 transposase B | transposase |
| HP1008 | Predicted IS200 transposase A | Transposase |
| HP1009 | Predicted integrase/recombinase xerCD family | Site-specific recombinase xerd |
| HP1010 | Predicted polyphosphate kinase | Polyphosphate kinase |
| HP1011 | Predicted dihydroorotate dehydrogenase | Dihydroorotate dehydrogenase |
| HP1012 | Predicted zinc protease | Zinc peptidase |
| HP1013 | Predicted dihydrodipicolinate synthetase | Dihydrodipicolinate synthase |
| HP1014 | Predicted 7 alpha-hydroxysteroid dehydrogenase involved in bile-acid metabolism | Enoyl-ACP Reductases III |
| HP1015 | Predicted coding region HP1015 with no homologue in the databases | Putative sortase |
| HP1016 | Predicted phosphatidylglycerophosphate synthase | phosphatidylglycerophosphate synthase |
| HP1017 | Predicted arginine permease | glutamate/gamma-aminobutyrate antipo rter |
| HP1018 | Predicted Secreted serine protease | F-actin-capping protein subunit alpha-1 |
| HP1019 | Predicted protease DO | Protease DO |
| HP1020 | Predicted bifunctional bifunctional methylerythritol-phosphate (MEP) cytidyltransferase/ methylerythritol-cyclodiphosphate synthetase (MECPS) protein involved in isoprenoid biosynthesis | ISPD/ISPF bifunctional enzyme |
| HP1021 | Predicted transcriptional regulator | Transcriptional regulatory protein |
| HP1022 | Predicted DNA-polymerase I-like 5’-3’ exonuclease | 5’-exonuclease(nuclease) exonuclease |
| HP1023 | Predicted coding region HP1023 with no homologue in the databases | Cell adhesion |
| HP1024 | Predicted co-chaperone protein | Human hsp40(Molecular chaperone) |
| HP1025 | Transcriptional activator of heat-shock proteins | Merr-like transcriptional regulator |
| HP1026 | Predicted coding region HP1026 | Replication-associated recombination protein |
| HP1027 | Fur regulatory protein involved in iron homeostasis | Ferric uptake regulation protein |
| HP1028 | Predicted coding region HP1028 with no homologue in the databases | Xenavidin |
| HP1029 | Predicted coding region HP1029 with no homologue in the databases | bacterial YhcH protein indicates a role in sialic acid catabolism. |
| HP1030 | Predicted flagellar motor switch protein | Flagellar motor switch protein FLIM |
| HP1031 | Predicted flagellar motor switch protein | Flagellar motor switch protein FLIN |
| HP1032 | Alternative transcription initiation factor sigma-28 | Rna polymerase sigma factor SIGMA-28 (FLIA) |
| HP1033 | Predicted coding region HP1033 with no homologue in the databases | Immunoglobulin G-binding protein |
| HP1034 | Predicted ATP-binding protein | Cell division inhibitor (MIND-1) family of ATPases |
| HP1035 | Predicted flagellar biosynthesis protein with a GTP-binding domain | Cell division protein |
| HP1036 | Predicted 7, 8-dihydro-6-hydroxymethylpterin-pyrophosphokinase | 7,8-dihydro-6-hydroxymethylpterin |
| HP1037 | Predicted proline peptidase | Proline dipeptidase |
| HP1038 | Predicted 3-dehydroquinate dehydratase | 3-dehydroquinate dehydratase |
| HP1039 | Predicted coding region HP1039 with no homologue in the databases | Nuclear transport |
| HP1040 | Predicted ribosomal protein S15 | 30S ribosomal protein S15 |
| HP1041 | Flagellar biosynthesis protein | Flagellar biosynthesis protein FLHA |
| HP1042 | Predicted coding region HP1042 | Exopolyphosphatase-related protein |
| HP1043 | Predicted transcriptional regulator | Transcriptional regulator |
| HP1044 | Predicted integral membrane protein | nucleotide phosphodiesterase |
| HP1045 | Predicted acetyl-CoA synthetase | Acetyl-coenzyme a synthetase |
| HP1046 | Predicted coding region HP1046 | Host factor for Q beta |
| HP1047 | Predicted ribosome-binding factor A | Ribosome-binding factor a |
| HP1048 | Predicted translation initiation factor IF-2 | Translation initiation factor |
| HP1049 | Predicted coding region HP1049 | 89 aa |
| HP1050() | Predicted homoserine kinase | Homoserine kinase |
| HP1051 | Predicted coding region HP1051 | o-sialoglycoprotein endopeptidase |
| HP1052 | Predicted UDP-3-0-acyl N-acetylglcosamine deacetylase | UDP-3-o-[3-hydroxymyristoyl] n-acetylglucosamine |
| HP1053 | Predicted septum site-directing protein | Septum site-determining protein MINC |
| HP1054 | Predicted coding region HP1054 | zinc containing peptidase |
| HP1055 | Predicted coding region HP1055 | outer membrane protein |
| HP1056 | Predicted coding region HP1056 | outer membrane protein |
| HP1057 | Predicted coding region HP1057 | Outer membrane protein A |
| HP1058 | Predicted 3-methyl-2-oxobutanoate hydroxymethyltransferase | 3-methyl-2-oxobutanoate hydroxymethyltransferase |
| HP1059 | Predicted Holliday junction DNA helicase | Holliday junction dna helicase RUVB |
| HP1060 | Predicted Sec-independent protein translocase protein involved in the twin-arginine translocation system | sec-independent translocase |
| HP1061 | Predicted Sec-independent protein translocase protein involved in the twin-arginine translocation system | twin arginine-targeting protein translocase TatC |
| HP1062 | Predicted S-adenosylmethionine:tRNA ribosyltransferase-isomerase | S-adenosylmethionine:trna ribosyltransferase- isomerase |
| HP1063 | Predicted glucose-inhibited division protein | Glucose-inhibited division protein B |
| HP1064 | Predicted coding region HP1064 with no homologue in the databases | prokaryotic metallothionein family protein |
| HP1065 | Predicted coding region HP1065 with no homologue in the databases | Probable transaldolase |
| HP1066 | Predicted outer membrane protein HorD | outer membrane protein HorD |
| HP1067 | Modulator of the flagellar rotation direction during chemotactic response | Chemotaxis protein CHEY homolog |
| HP1068 | Predicted ribosomal protein L11 methyltransferase | Ribosomal protein l11 methyltransferase |
| HP1069 | Cell division protein/metalloprotease | ATP-dependent zinc metalloprotease |
| HP1070 | Predicted coding region HP1070 with no homologue in the databases | Putative uncharacterized protein |
| HP1071 | Predicted phosphatidylserine synthase | phosphatidylserine synthase |
| HP1072 | Copper-transporting ATPase/P-type transporting ATPase involved in transition metal resistance | copper-transporting PIB-type ATPase |
| HP1073 | Predicted copper ion binding protein | copper ion binding protein |
| HP1074 | Predicted coding region HP1074 with no homologue in the databases | Trafficking protein particle complex subunit |
| HP1075 | Predicted secreted protein | Carboxypeptidase M |
| HP1076 | Predicted coding region HP1076 | Putative uncharacterized protein |
| HP1077 | High-affinity nickel transporter required for scavenging of nickel ions under normal gastric conditions | |  | Nucleocapsid (NC) Protein | | --- | --- | |
| HP1078 | Predicted coding region HP1078 with no homologue in the databases | 167 aa |
| HP1079 | Predicted coding region HP1079 with no homologue in the databases | DNA double-strand break repair rad50 ATPase |
| HP1080 | Predicted integral membrane protein | Hypothetical protein AF1548 |
| HP1081 | Predicted coding region HP1081 with no homologue in the databases | neuraminyllactose-binding hemagglutinin homolog   |  | | --- | |
| HP1082 | Predicted lipid A and glycerophospholipid transporter involved in the biogenesis of LPS and of the outer membrane | Lipid A export ATP-binding/permease protein msbA |
| HP1083 | Predicted outer membrane protein HofB | Glycine-glutamate dipeptide porin OpdP |
| HP1084 | Aspartate transcarbamoylase | Aspartate transcarbamoylase |
| HP1085 | Predicted coding region HP1085 with no homologue in the databases | Putative uncharacterized protein |
| HP1086 | Pore-forming cytolysin |  hemolysin, putative |
| HP1087 | Bifunctional riboflavin kinase/FMN adenylyltransferase | Riboflavin biosynthesis protein |
| HP1088 | Predicted transketolase A | Transketolase |
| HP1089 | Predicted coding region HP1089 | ATP-dependent helicase/deoxyribonuclease subunit |
| HP1090 | Predicted septum formation protein | DNA translocase |
| HP1091 | Predicted alpha-ketoglutarate permease | Glucose transporters |
| HP1092 | Predicted flagellar basal-body rod protein | Flagellar hook protein FLGE |
| HP1093 | Predicted coding region HP1093 with no homologue in the databases | Caa3-type cytochrome oxidase subunit iv |
| HP1094 | Predicted coding region HP1094 with no homologue in the databases | NUDIX domain-containing protein |
| HP1095 | Predicted IS605 transposase B | |  | IS605 transposase (tnpb) | | --- | --- | |
| HP1096 | Predicted IS605 transposase A | IS605 transposase |
| HP1097 | Predicted coding region HP1097 with no homologue in the databases | Leiurotoxin |
| HP1098 | Cysteine-rich protein C | Cysteine rich protein b |
| HP1099 | Predicted 2-keto-3-deoxy-6-phosphogluconate aldolase | Khg/kdpg aldolase |
| HP1100 | Predicted 6-phosphogluconate dehydratase | 6-phosphogluconate dehydratase |
| HP1101 | Predicted glucose-6-phosphate dehydrogenase | Glucose 6-phosphate dehydrogenase |
| HP1102 | Predicted 6-phosphogluconolactonase | Putative 6-phosphogluconolactonase |
| HP1103 | Predicted glucokinase | Glucokinase |
| HP1104 | Predicted mannitol dehydrogenase | Mannitol dehydrogenase |
| HP1105 | Predicted LPS biosynthesis protein | alpha-1,4-galactosyl transferase |
| HP1106 | Predicted coding region HP1106 | Thioredoxin domain protein |
| HP1107 | Predicted outer membrane protein HorH | outer membrane protein |
| HP1108 | Pyruvate ferredoxin oxidoreductase, gamma subunit | Keto/oxoacid ferredoxin oxidoreductase, gamma subunit |
| HP1109 | Pyruvate ferredoxin oxidoreductase, delta subunit | Ferredoxin |
| HP1110 | Pyruvate ferredoxin oxidoreductase, alpha subunit | Pyruvate-ferredoxin oxidoreductase |
| HP1111 | Pyruvate ferredoxin oxidoreductase, beta subunit | Pyruvate-ferredoxin oxidoreductase |
| HP1112 | Predicted adenylosuccinate lyase |  adenylosuccinate lyase |
| HP1113 | Predicted outer membrane protein HorI | | outer membrane protein HorI | | --- |  |  | | --- | |
| HP1114 | Predicted excinuclease ABC subunit B/nicks DNA 3'’to the damaged site | EXCINUCLEASE UVRABC COMPONENT UVRB |
| HP1115 | Predicted coding region HP1115 | Apolipoprotein E |
| HP1116 | Predicted coding region HP1116 | Probable atp-dependent RNA helicase |
| HP1117 | Cysteine-rich protein X | Cysteine rich protein B |
| HP1118 | Gamma-glutamyltranspeptidase | Gamma-glutamyltranspeptidase (ggt) large subunit |
| HP1119 | Predicted flagellar hook-associated protein 1 | Flagellar hook-associated protein |
| HP1120 | Predicted coding region HP1120 | putative flagella synthesis protein 144aa |
| HP1121 | GCGC site-specific type II m5C methylase | Protein (cytosine-specific methyltransferase) |
| HP1122 | Temporal regulator of flagellar apparatus biogenesis | anti sigma factor FlgM |
| HP1123 | Predicted FKBP-type peptidyl-prolyl cis-trans isomerase | FKBP-type peptidyl-prolyl cis-trans isomerase |
| HP1124 | Predicted periplasmic protein of unknown function | TYPE 4 fimbrial biogenesis protein PILF |
| HP1125 | Predicted peptidoglycan-associated lipoprotein precursor | Outer membrane protein |
| HP1126 | Predicted TonB-independent protein-uptake protein | Protein TOLB |
| HP1127 | Predicted coding region HP1127 with no homologue in the databases | TOL A protein |
| HP1128 | Predicted coding region HP1128 with no homologue in the databases | |  | Large-conductance mechanosensitive channel | | --- | --- | |
| HP1129 | Predicted biopolymer transport accessory protein | Biopolymer transport exbd protein |
| HP1130 | Predicted biopolymer transport accessory protein | transmembrane segments of integral membrane proteins |
| HP1131 | Predicted ATP synthase F1 epsilon chain | ATP synthase epsilon subunit |
| HP1132 | Predicted ATP synthase F1 beta chain | ATP synthase alpha chain, mitochondrial |
| HP1133 | Predicted ATP synthase F1 gamma chain | ATP synthase epsilon subunit |
| HP1134 | Predicted ATP synthase F1 alpha chain | ATP synthase subunit alpha |
| HP1135 | Predicted ATP synthase F1 delta chain | Predicted ATP synthase F1 delta chain |
| HP1136 | Predicted ATP synthase F0 B chain | ATP synthase b chain, mitochondrial |
| HP1137 | Predicted ATP synthase F0 B'’chain | ATP synthase b chain, mitochondrial |
| HP1138 | Predicted plasmid replication-partition related protein | Chromosome partitioning protein PARB |
| HP1139 | Predicted SpoOJ regulator | Segregation protein SOJ |
| HP1140 | Predicted bifunctional biotin operon repressor/biotin acetyl coenzyme A carboxylase synthetase | Biotin [acetyl-CoA-carboxylase] ligase |
| HP1141 | Predicted methionyl-tRNA formyltransferase | Methionyl-trna fmet formyltransferase |
| HP1142 | Predicted coding region HP1142 with no homologue in the databases | DNA double-strand break repair rad50 ATPase |
| HP1143 | Predicted coding region HP1143 | Signal transducer and activator of transcription |
| HP1144 | Predicted coding region HP1144 | Replication protein A 70 kDa DNA-binding subunit |
| HP1145 | Predicted coding region HP1145 | Protein of unknown function VPA0982 |
| HP1146 | Predicted coding region HP1146 | Anabaena sensory rhodopsin transducer protein |
| HP1147 | Predicted ribosomal protein L19 | 50s ribosomal protein l19 |
| HP1148 | Predicted tRNA (guanine-N1)-methyltransferase | Trna (guanine-n(1)-)-methyltransferase |
| HP1149 | Predicted 16s rRNA processing protein | 16S rRNA processing protein |
| HP1150 | Predicted coding region HP1150 | Hypothetical protein |
| HP1151 | Predicted ribosomal protein S16 | 30s ribosomal protein s16 |
| HP1152 | Predicted signal recognition particle protein | Signal recognition particle protein |
| HP1153 | Predicted valyl-tRNA synthetase | Valyl-trna synthetase |
| HP1154 | Predicted coding region HP1154 | Contractile protein |
| HP1155 | Predicted UDP-N-acetylglucosamine lipidI transferase | Udp-n-acetylglucosamine-n-acetylmuramyl- (pentapeptide) pyrophosphoryl-undecaprenol n- acetylglucosamine transferase |
| HP1156 | Predicted outer membrane protein HopI | Outer membrane protein H1 |
| HP1157 | Predicted outer membrane protein HopL | TGF-beta receptor type-1 |
| HP1158 | Predicted pyrroline-5-carboxylate reductase | Putative pyrroline carboxylate reductase |
| HP1159 | Predicted cell filamentation protein | Cell filamentation protein, putative |
| HP1160 | Predicted coding region HP1160 | Aminopeptidase 1 |
| HP1161 | Predicted flavodoxin | Flavodoxin |
| HP1162 | Predicted integral membrane protein | Sphingosine 1-phosphate receptor 1, Lysozyme |
| HP1163 | Predicted cation transport subunit for cbb3-type oxidase | potassium large conductance calcium-activated |
| HP1164 | Thioredoxin reductase | Thioredoxin reductase |
| HP1165 | Predicted coding region HP1165 | Glycerol-3-phosphate transporter |
| HP1166 | Predicted glucose-6-phosphate isomerase | Phosphoglucose isomerase |
| HP1167 | Predicted outer membrane protein HofH | outer membrane porin |
| HP1168 | Predicted carbon starvation protein | Cullin homolog(ligase) |
| HP1169 | Predicted glutamine ABC transporter, permease protein | D-methionine transport system permease protein metI |
| HP1170 | Predicted glutamine ABC transporter, permease protein | |  | D-methionine transport system permease  protein metI | | --- | --- | |
| HP1171 | Predicted glutamine ABC transporter, ATP-binding protein | Amino acid abc transporter |
| HP1172 | Predicted glutamine ABC transporter, periplasmic glutamine-binding protein | Putative amino-acid transporter periplasmic |
| HP1173 | Predicted coding region HP1173 with no homologue in the databases | 183 aa |
| HP1174 | Predicted glucose/galactose transporter | L-fucose-proton symporter |
| HP1175 | Predicted integral membrane protein | Uracil permease |
| HP1176 | Predicted coding region HP1176 with no homologue in the databases | Formin-2 |
| HP1177 | Predicted outer membrane protein HopQ | | outer membrane protein | | --- | |
| HP1178 | Predicted purine-nucleoside phosphorylase | Purine nucleoside phosphorylase |
| HP1179 | Predicted phosphopentomutase | Phosphopentomutase |
| HP1180 | Predicted sodium/nucleoside cotransporter | nucleoside transporter protein |
| HP1181 | Predicted multidrug-efflux transporter | multidrug-efflux transporter |
| HP1182 | Predicted coding region HP1182 | tRNA(ile)-lysidine synthase |
| HP1183 | Predicted Na+/H+ antiporter | Na(+)/H(+) antiporter 1 |
| HP1184 | Predicted integral membrane protein | Cation-bound Multidrug and Toxin Compound Extrusion (MATE) transporter |
| HP1185 | Predicted sugar efflux transporter protein | Glycerol-3-phosphate transporter |
| HP1186 | Alpha-carbonic anhydrase | Carbonic anhydrase |
| HP1187 | Predicted coding region HP1187 | RIKEN cDNA 2610044O15 |
| HP1188 | Predicted coding region HP1188 | fucose-binding lectin protein |
| HP1189 | Predicted aspartate-semialdehyde dehydrogenase | Aspartate beta-semialdehyde dehydrogenase |
| HP1190 | Predicted histidyl-tRNA synthetase | Histidyl-tRNA synthetase |
| HP1191 | Predicted ADP-heptose-LPS heptosyltransferase II | ADP-heptose lps heptosyltransferase II |
| HP1192 | Predicted secreted protein | Nucleoporin NSP1 |
| HP1193 | Predicted aldo-keto reductase | Perakine reductase |
| HP1194 | Predicted coding region HP1194 with no homologue in the databases | 28aa |
| HP1195 | Predicted translation elongation factor G | Elongation factor G |
| HP1196 | Predicted ribosomal protein S7 | 16S rRNA |
| HP1197 | Predicted ribosomal protein S12 | 30S ribosomal protein S12 |
| HP1198 | DNA-dependent RNA polymerase beta-beta prime subunit | DNA-directed RNA polymerase subunit beta |
| HP1199 | Predicted ribosomal protein L7/L12 | 50s ribosomal protein(L7/L12) |
| HP1200 | Predicted ribosomal protein L10 | 50s ribosomal protein(L10) |
| HP1201 | Predicted ribosomal protein L1 | 50s ribosomal protein(L1) |
| HP1202 | Predicted ribosomal protein L11 | 50S ribosomal protein L11 |
| HP1203 | Predicted transcription termination factor G | Transcription antitermination protein NUSG |
| HP1203.1 | Predicted preprotein translocase subunit involved in protein secretion through or insertion into the cytoplasmic membrane | 50S ribosomal protein L33 |
| HP1204 | Predicted ribosomal protein L33 | 50S ribosomal protein L33 |
| HP1205 | Predicted translation elongation factor Tu | Elongation factor TU |
| HP1206 | Predicted ABC-transporter, ATP-binding domain | |  | ATP-binding cassette sub-family b  member 10 mitochondrial | | --- | --- | |
| HP1207 | Predicted coding region HP1207 with no homologue in the databases | novel predicted phosphatase |
| HP1208 | Ubiquitous CATG site-specific type II m6A methylase | adenine-specific methyltransferase |
| HP1209 | Non-functional CATG site-specific type II restriction endonuclease, identified as an adherence to gastric epithelial cells induced gene iceA1 | HNH endonuclease |
| HP1210 | Predicted serine acetyltransferase | Serine acetyltransferase |
| HP1211 | Predicted coding region HP1211 with no homologue in the databases | alginate lyase |
| HP1212 | Predicted ATP synthase F0 C chain | Atp synthase subunit c |
| HP1213 | Predicted polynucleotide phosphorylase | Polyribonucleotide nucleotidyltransferase |
| HP1214 | Predicted coding region HP1214 | Uracil phosphoribosyltransferase |
| HP1215 | Predicted organic solvent tolerance protein | organic solvent tolerance protein |
| HP1216 | Predicted organic solvent tolerance protein | periplasmic lipopolysaccharide transport protein |
| HP1217 | Predicted coding region HP1217 with no homologue in the databases | Aquaporin 1 |
| HP1218 | Predicted glycinamide ribonucleotide synthetase/ second step in de novo purine synthesis | Phosphoribosylamine--glycine ligase |
| HP1219 | Predicted coding region HP1219 with no homologue in the databases | periplasmic divalent cation tolerance protein |
| HP1220 | Predicted ABC transporter/ATP-binding protein | ABC transporter, ATP-binding protein |
| HP1221 | Predicted undecaprenyl pyrophosphate synthase involved in the synthesis of the C55 lipid carrier of peptidoglycan, LPS, LOS, capsule, teichoic and lipoteichoic precursors | Undecaprenyl pyrophosphate synthase |
| HP1222 | Predicted D-lactate dehydrogenase | D-lactate dehydrogenase |
| HP1223 | Predicted coding region HP1223 | Uncharacterized protein hp1203 |
| HP1224 | Predicted uroporphyrinogen III cosynthase | Uroporphyrinogen iii cosynthase (hemd) |
| HP1225 | Predicted CrcB integral membrane protein involved chromosome condensation | Acetylcholine receptor protein, delta chain |
| HP1226 | Predicted oxygen-independent coproporphyrinogen III oxidase | Oxygen-independent coproporphyrinogen iii oxidase |
| HP1227 | Predicted cytochrome c553 | Cytochrome c-553 |
| HP1228 | Predicted diadenosine polyphosphate hydrolase | Diadenosine 5',5'''-p1,p4-tetraphosphate |
| HP1229 | Predicted aspartokinase | Aspartokinase |
| HP1230 | Predicted coding region HP1230 | HOBA DnaA-binding proteins HobA |
| HP1231 | Delta prime subunit of the DNA polymerase III holoenzyme needed for DNA sliding clamp and chromosome replication | DNA polymerase III subunit gamma |
| HP1232 | Predicted dihydropteroate synthase | 2-amino-4-hydroxy-6-hydroxymethyldihydropteridine pyrophosphokinase/dihydropteroate synthase |
| HP1233 | Predicted coding region HP1233 with no homologue in the databases | Ribosomal protein L13 |
| HP1234 | Predicted coding region HP1234 | transmembrane domain of the multidrug-resistance antiporter |
| HP1235 | Predicted integral membrane protein | Oligosaccharide transferase to N-glycosylate |
| HP1236 | Predicted coding region HP1236 | 2-methylisoborneol synthase |
| HP1237 | Predicted carbamoyl-phosphate synthetase small chain | Carbamoyl phosphate synthetase (large chain) |
| HP1238 | Formamidase involved in nitrogen metabolism | Formamidase |
| HP1239 | Predicted coding region HP1239 with no homologue in the databases | Dolichyl-diphosphooligosaccharide--protein gl |
| HP1240 | Predicted Maf protein likely to be involved in septum formation | Septum formation protein Maf |
| HP1241 | Predicted alanyl-tRNA synthetase | Alanyl-trna synthetase |
| HP1242 | Predicted coding region HP1242 | hypothetical protein HP1242 |
| HP1243 | Adhesin binding fucosylated Lewis b (Leb) histo-blood group antigen | Outer membrane protein H1 |
| HP1244 | Predicted ribosomal protein S18 | 30s ribosomal protein s18 |
| HP1245 | Predicted single-strand DNA-binding protein/DNA helix-destabilizing protein | Single-stranded dna binding protein |
| HP1246 | Predicted ribosomal protein S6 | 30s ribosomal protein s6 |
| HP1247 | Delta subunit of the DNA polymerase III holoenzyme nedded for DNA sliding clamp and chromosome replication | DNA polymerase III, delta subunit |
| HP1248 | Predicted 3'-5' exoribonuclease R | Exoribonuclease 2 |
| HP1249 | Predicted shikimate 5-dehydrogenase | Shikimate dehydrogenase |
| HP1250 | Predicted coding region HP1250 with no homologue in the databases | Dipeptidyl-peptidase VI |
| HP1251 | Predicted oligopeptide ABC transporter, permease protein | D-methionine transport system permease protein |
| HP1252 | Predicted oligopeptide ABC transporter, periplasmic oligopeptide-binding protein | Oligopeptide-binding protein appa |
| HP1253 | Predicted tryptophanyl-tRNA synthetase | Tryptophanyl-trna synthetase |
| HP1254 | Predicted biotin synthesis protein | biotin synthase |
| HP1255 | Predicted protein translocation protein | Protein-export membrane protein secG |
| HP1256 | Predicted ribosome releasing factor | Ribosome recycling factor |
| HP1257 | Predicted orotate phosphoribosyltransferase | Orotate phosphoribosyltransferase |
| HP1258 | Predicted coding region HP1258 | 154 aa |
| HP1259 | Predicted coding region HP1259 | NAD-dependent deacetylase |
| HP1260 | Predicted NADH-ubiquinone oxidoreductase chain A | NADH-quinone oxidoreductase subunit A |
| HP1261 | Predicted NADH-ubiquinone oxidoreductase chain B | NADH-quinone oxidoreductase chain B |
| HP1262 | Predicted NADH-ubiquinone oxidoreductase chain C | Nadh-quinone oxidoreductase chain C |
| HP1263 | Predicted NADH-ubiquinone oxidoreductase chain D | NADH-quinone oxidoreductase chain D |
| HP1264 | Predicted NADH-ubiquinone oxidoreductase chain E | NADH dehydrogenase I subunit E |
| HP1265 | Predicted NADH-ubiquinone oxidoreductase chain F | formate dehydrogenase H |
| HP1266 | Predicted NADH-ubiquinone oxidoreductase chain G | Nadh-quinone oxidoreductase(chain G) |
| HP1267 | Predicted NADH-ubiquinone oxidoreductase chain H | Nadh-quinone oxidoreductase(chain H) |
| HP1268 | Predicted NADH-ubiquinone oxidoreductase chain I | NADH-quinone oxidoreductase chaiN 1 |
| HP1269 | Predicted NADH-ubiquinone oxidoreductase chain J | NADH-quinone oxidoreductase subunit J |
| HP1270 | Predicted NADH-ubiquinone oxidoreductase chain K | NADH-quinone oxidoreductase subunit K |
| HP1271 | Predicted NADH-ubiquinone oxidoreductase chain L | NADH-quinone oxidoreductase subunit L |
| HP1272 | Predicted NADH-ubiquinone oxidoreductase chain M | NADH-quinone oxidoreductase subunit M |
| HP1273 | Predicted NADH-ubiquinone oxidoreductase chain N | NADH-quinone oxidoreductase subunit N |
| HP1274 | Predicted paralysed flagella protein | ciliary or flagellar motility |
| HP1275 | Predicted phosphomannomutase | Phosphomannomutase |
| HP1276 | Predicted coding region HP1276 with no homologue in the databases | 94 aa |
| HP1277 | Predicted tryptophan synthase, alpha subunit | Tryptophan synthase alpha chain |
| HP1278 | Predicted tryptophan synthase, beta subunit | Tryptophan synthase beta chain 1 |
| HP1279 | Predicted bifunctional N-(5'-phospho-ribosyl)anthraate isomerase/Indole-3-glycerol phosphate synthase | N-(5'phosphoribosyl)anthranilate isomerase |
| HP1280 | Predicted anthraate phosphoribosyltransferase | Anthranilate phosphoribosyltransferase |
| HP1281 | Predicted anthraate synthase component II, glutamine amido-transferase | Anthranilate synthase |
| HP1282 | Predicted anthraate synthase component I | Anthranilate synthase component i |
| HP1283 | Predicted coding region HP1283 with no homologue in the databases | glycosyltransferase 9 family protein |
| HP1284 | Predicted coding region HP1284 | ADP-heptose lps heptosyltransferase ii |
| HP1285 | Predicted acid phosphatase lipoprotein | Lipoprotein |
| HP1286 | Predicted secreted protein | Conserved hypothetical secreted protein |
| HP1287 | Predicted transcriptional regulator | Transcriptional regulator |
| HP1288 | Predicted coding region HP1288 | |  | Protein Transport | | --- | --- | |
| HP1289 | Predicted coding region HP1289 | Histone deacetylase complex subunit |
| HP1290 | Predicted nicotinamide mononucleotide transporter | |  | nicotinamide mononucleotide transporter | | --- | --- | |
| HP1291 | Predicted coding region HP1291 | Thiamine pyrophosphokinase |
| HP1292 | Predicted ribosomal protein L17 | 50S ribosomal protein L27 |
| HP1293 | Predicted alpha subunit of the DNA-dependent RNA polymerase core enzyme | DNA-directed rna polymerase alpha chain |
| HP1294 | ribosomal protein S4 (rps4) | 30S ribosomal protein S4 |
| HP1295 | ribosomal protein S11 (rps11) | 30S ribosomal protein S11 |
| HP1296 | ribosomal protein S13 (rps13) | 30S ribosomal protein S13 |
| HP1297 | ribosomal protein L36 (rpl36) | Ribosomal Protein L36 |
| HP1298 | Predicted translation initiation factor IF-1 | Initiation factor 1 |
| HP1299 | Predicted methionine amino peptidase | Methionine aminopeptidase |
| HP1300 | Predicted preprotein translocase subunit | Preprotein translocase SecY subunit |
| HP1301 | Predicted ribosomal protein L15 | 50S ribosomal protein L15 |
| HP1302 | Predicted ribosomal protein S5 | Ribosomal protein S5 |
| HP1303 | Predicted ribosomal protein L18 | 50S Ribosomal Protein L18 |
| HP1304 | Predicted ribosomal protein L6 | 50S Ribosomal Protein L6 |
| HP1305 | Predicted ribosomal protein S8 | Protein (S8 ribosomal protein) |
| HP1306 | Predicted ribosomal protein S14 | 30S ribosomal protein S14 |
| HP1307 | Predicted ribosomal protein L5 | 50S ribosomal protein L5 |
| HP1308 | Predicted ribosomal protein L24 | 50S ribosomal protein L24 |
| HP1309 | Predicted ribosomal protein L14 | 50S ribosomal protein L14 |
| HP1310 | Predicted ribosomal protein S17 | 30S ribosomal protein S17 |
| HP1311 | Predicted ribosomal protein L29 | 50S ribosomal protein L29 |
| HP1312 | Predicted ribosomal protein L16 | 50S ribosomal protein L16 |
| HP1313 | Predicted ribosomal protein S3 | 30S ribosomal protein S3 |
| HP1314 | Predicted ribosomal protein L22 | Protein (ribosomal protein L22) |
| HP1315 | Predicted ribosomal protein S19 | 30S ribosomal protein S19 |
| HP1316 | Predicted ribosomal protein L2/peptidyl-transferase | 50S ribosomal protein L2 |
| HP1317 | Predicted ribosomal protein L23 | 50S ribosomal protein L23 |
| HP1318 | Predicted ribosomal protein L4 | 50S ribosomal protein L4 |
| HP1319 | Predicted ribosomal protein L3 | 50S ribosomal protein L3 |
| HP1320 | Predicted ribosomal protein S10 | 30S ribosomal protein S10 |
| HP1321 | Predicted coding region HP1321 | Walker-type ATPase |
| HP1322 | Predicted coding region HP1322 with no homologue in the databases | ligase |
| HP1323 | Predicted ribonuclease HII | Ribonuclease HII |
| HP1324 | Predicted coding region HP1324 with no homologue in the databases | Ion transport 2 domain protein, voltage-gated sod |
| HP1325 | Predicted fumarase | Fumarase C |
| HP1326 | Predicted coding region HP1326 with no homologue in the databases | Tripartite motif protein 45 |
| HP1327 | Predicted coding region HP1327 with no homologue in the databases | Outer membrane protein tolC |
| HP1328 | Predicted cobalt-zinc-cadmium resistance protein/cation efflux system protein | Cation efflux system protein cusB |
| HP1329 | Predicted cobalt-zinc-cadmium resistance protein/cation efflux system protein | cation efflux system protein |
| HP1330 | Predicted branched-chain amino acid transport protein | Transmembrane Protein |
| HP1331 | Predicted branched-chain amino acid transport protein | Electron Transport |
| HP1332 | Predicted co-chaperone protein | Putative chaperone DnaJ |
| HP1333 | Predicted coding region HP1333 with no homologue in the databases | Spectrin alpha chain |
| HP1334 | Predicted coding region HP1334 | Formyltetrahydrofolate deformylase |
| HP1335 | Predicted tRNA (5-methylaminomethyl-2-thiouridylate)-methyltransferase | Probable tRNA (5-methylaminomethyl-2-  Thiouridylat e methyltransferase |
| HP1336 | Predicted coding region HP1336 with no homologue in the databases | DnaJ molecular chaperone homology domain |
| HP1337 | Predicted nicotinate-nucleotide adenyltransferase involved in NAD biosynthesis | Nicotinate-nucleotide adenylyltransferase |
| HP1338 | Predicted nickel uptake regulation protein involved in nickel homeostasis | Putative nickel-responsive regulator |
| HP1339 | Predicted biopolymer transport accessory protein | |  | Potassium voltage-gated channel | | --- | --- | |
| HP1340 | Predicted biopolymer transport and binding protein | Biopolymer transport exbD protein |
| HP1341 | Predicted siderophore-mediated iron transport protein | siderophore-mediated iron transport protein (tonB) |
| HP1342 | Predicted outer membrane protein HopN | Outer membrane protein H1 |
| HP1343 | Iconserved hypothetical ntegral membrane protein | hypothetical protein BA_2335 |
| HP1344 | Predicted magnesium and cobalt transport protein | Magnesium transport protein CorA |
| HP1345 | Predicted phosphoglycerate kinase | 3-phosphoglycerate kinase |
| HP1346 | Predicted glyceraldehyde-3-phosphate dehydrogenase | Glyceraldehyde-3-phosphate dehydrogenase |
| HP1347 | Predicted uracil-DNA glycosylase | Uracil-dna glycosylase |
| HP1348 | Predicted 1-acyl-glycerol-3-phosphate acyltransferase | Glycerol-3-phosphate acyltransferase |
| HP1349 | Predicted periplasmic protein of unknown function | Probable surface protein |
| HP1350 | Predicted carboxyl-terminal protease | Photosystem ii d1 protease(hydrolases)/ carboxyl-terminal protease/ |
| HP1351 | GANTC site-specific type II restriction endonuclease | type II restriction endonuclease |
| HP1352 | GANTC site-specific type II m6A methylase involved in DNA modification | N6 adenine specific DNA methyltransferase |
| HP1353 | Predicted adenine specific DNA methyltransferase | Putative adenine specific DNA methyltransferas |
| HP1354 | Predicted adenine specific DNA methyltransferase | Adenine-specific methyltransferase |
| HP1355 | Predicted quinolinate phosphoribosyl transferase | Quinolinic acid phosphoribosyltransferase |
| HP1356 | Predicted quinolinate synthetase A | Quinolinate synthetase a |
| HP1357 | Predicted phosphatidylserine decarboxylase | Ubiquitin-conjugating enzyme |
| HP1358 | Predicted coding region HP1358 with no homologue in the databases | COLICIN E3 |
| HP1359 | Predicted coding region HP1359 with no homologue in the databases | Serine/threonine-protein phosphatase |
| HP1360 | Predicted 4-hydroxybenzoate octaprenyltransferase | Cytochrome b-large subunit |
| HP1361 | Predicted DNA competence protein | |  | competence family protein | | --- | --- | |
| HP1362 | Predicted DNA replicative helicase | Replicative dna helicase |
| HP1363 | Predicted integral membrane protein | Carbohydrate kinase |
| HP1364 | Signal-transducing protein, histidine kinase | Sensor histidine kinase |
| HP1365 | Response regulator | Sensory transduction protein |
| HP1366 | Asymmetric GAAGA site-specific type IIS restriction endonuclease, an isoschizomer of MboII | |  | type IIS restriction enzyme R protein | | --- | --- | |
| HP1367 | Asymmetric GAAGA site-specific type IIS m6A methylase involved in DNA modification | Adenine-specific methyltransferase mboiia |
| HP1368 | Asymmetric TCTTC site-specific type IIS m4C methylase involved in DNA modification | |  | Protein (N-4 cytosine-specific  methylt ransferase pvu ii) | | --- | --- | |
| HP1369 | Predicted type III restriction enzyme M protein | |  | adenine-specific DNA methylase | | --- | --- | |
| HP1370 | Predicted type III restriction enzyme M protein | DNA modification methylase [DNA replication, recombination, and repair |
| HP1371 | Predicted coding region HP1371 | Structure of the motor subunit of type I restriction-modification complex   |  |  | | --- | --- | |
| HP1372 | Predicted rod shape-determining protein | Cell shape regulation |
| HP1373 | Predicted rod shape-determining actin-like protein | Rod shape-determining protein MREB |
| HP1374 | Predicted ATP-dependent C1p protease, ATPase subunit/chaperone protein | ATP-dependent Clp protease ATP-binding subunit |
| HP1375 | Predicted UDP-N-acetylglucosamine acyltransferase | |  | Acyl-[acyl-carrier-protein]--UDP-N-  acetylglucosamine O-acyltransferase | | --- | --- | |
| HP1376 | Predicted (3R)-hydroxymyristoyl-(acyl carrier protein) dehydratase | (3R)-hydroxymyristoyl-(acyl carrier protein) dehydratase |
| HP1377 | Predicted coding region HP1377 | hypothetical protein BH3618 |
| HP1378 | Predicted competence lipoprotein | Lipoprotein (outermembrane protein biogenesis in bacteria) |
| HP1379 | Predicted ATP-dependent protease | Atp-dependent protease |
| HP1380 | Predicted prephenate dehydrogenase | Prephenate dehydrogenase |
| HP1381 | Predicted coding region HP1381 | Apical membrane antigen, putative |
| HP1382 | Predicted endonuclease G | Endonuclease |
| HP1383 | Predicted type I R-M system specificity subunit | Type I restriction-modification enzyme, S subunit |
| HP1384 | Predicted coding region HP1384 | Beta-1,3-glucanase |
| HP1385 | Predicted fructose-1,6-bisphosphatase | Fructose-1,6-bisphosphatase |
| HP1386 | Predicted D-ribulose-5-phosphate 3 epimerase | D-ribulose-phosphate 3-epimerase |
| HP1387 | Predicted DNA polymerase III epsilon subunit | |  | DNA polymerase III polC-type | | --- | --- | |
| HP1388 | Predicted coding region HP1388 with no homologue in the databases | Type VI secretion system |
| HP1389 | Predicted coding region HP1389 with no homologue in the databases | Cell-cycle regulator |
| HP1390 | Predicted coding region HP1390 with no homologue in the databases | glycoside hydrolase domain |
| HP1391 | Predicted coding region HP1391 with no homologue in the databases | putative lipoprotein |
| HP1392 | Predicted fibronectin/fibrinogen-binding protein | Fibrinogen binding protein |
| HP1393 | Predicted DNA repair protein | Dna repair protein RECN |
| HP1394 | Predicted inorganic polyphosphate/ATP-NAD kinase | putative kinase |
| HP1395 | Predicted outer membrane protein HorL | Outer membrane protein A |
| HP1396 | Predicted coding region HP1396 | Endonuclease I |
| HP1397 | Predicted coding region HP1397 | RNA Binding Protein |
| HP1398 | Predicted alanine dehydrogenase | Alanine dehydrogenase |
| HP1399 | Arginase involved in nitrogen metabolism | Arginase-1 |
| HP1400 | Predicted Iron(III) dicitrate transport protein | Iron(iii) dicitrate traport protein(Membrane protein |
| HP1401 | Predicted coding region HP1401 | Matrix metalloproteinase-14 |
| HP1402 | Predicted type I R-M system restriction subunit | motor subunit of type I restriction-modification complex |
| HP1403 | Predicted type I R-M system modification subunit | Type I restriction enzyme EcoKI M protein |
| HP1404 | Predicted type I R-M system modification subunit | Type I restriction-modification enzyme, S sub |
| HP1405 | Predicted coding region HP1405 with no homologue in the databases | Contractile Protein |
| HP1406 | Predicted biotin synthetase | Biotin synthase |
| HP1407 | Predicted ribonuclease N | Sphingosine 1-phosphate receptor 1, Lysozyme |
| HP1408 | Predicted coding region HP1408 with no homologue in the databases | putative lipoprotein |
| HP1409 | Predicted coding region HP1409 | DNA binding protein |
| HP1410 | Predicted coding region HP1410 with no homologue in the databases | Single-stranded DNA specific exonuclease RecJ |
| HP1411 | Predicted coding region HP1411 | Chaperone |
| HP1412 | Predicted coding region HP1412 | |  | Transcription | | --- | --- | |
| HP1413 | Predicted coding region HP1413 | NADPH-dependent 7-cyano-7-deazaguanine reductase |
| HP1414 | Predicted coding region HP1414 | 113 aa |
| HP1415 | Predicted tRNA delta(2)-isopentenylpyrophosphate transferase | tRNA delta(2)-isopentenylpyrophosphate transferas |
| HP1416 | Predicted lipopolysaccharide 1,2-glycosyltransferase | alpha-1,4-galactosyl transferase |
| HP1417 | Predicted coding region HP1417 | Alkaline Phosphatase Superfamily protein |
| HP1418 | Predicted UDP-N-acetylenolpyruvoylglucosamine reductase | UDP-N-acetylenolpyruvoylglucosamine reductase |
| HP1419 | Flagellar biosynthetic protein | flagellar biosynthesis protein FliQ |
| HP1420 | Predicted flagellar export protein ATP synthase | flagellar type III ATPase |
| HP1421 | Predicted type IV secretion system ATPase | Type iv secretion system protein virb11 |
| HP1422 | Predicted isoleucyl-tRNA synthetase | Isoleucyl-trna synthetase |
| HP1423 | Predicted coding region HP1423 | alphaL motif related to RNA binding |
| HP1424 | Predicted coding region HP1424 with no homologue in the databases | Isoleucyl-tRNA synthetase |
| HP1425 | Predicted coding region HP1425 with no homologue in the databases | L-arabinitol 4-dehydrogenase |
| HP1426 | Predicted coding region HP1426 with no homologue in the databases | Alginate lyase |
| HP1427 | Histidine-rich metal binding polypeptide | histidine-rich metal-binding protein |
| HP1428 | Predicted coding region HP1428 | Ribosomal RNA large subunit methyltransferase(oxidoreductase) |
| HP1429 | Predicted polysialic acid capsule expression protein | phosphosugar isomerase involved in capsule formation |
| HP1430 | Predicted ATP/GTP binding protein | Alkylsulfatase |
| HP1431 | Predicted 16S rRNA (adenosine-N6,N6-)-dimethyltransferase | Ribosomal RNA small subunit methyltransferase |
| HP1432 | Histidine and glutamine-rich metal-binding protein | |  | histidine and glutamine-rich protein | | --- | --- | |
| HP1433 | Predicted coding region HP1433 with no homologue in the databases | Restriction endonuclease |
| HP1434 | Predicted formyltetrahydrofolate hydrolase | Formyltetrahydrofolate deformylase |
| HP1435 | Predicted signal peptide protease IV | Signal peptide peptidase (Spp) |
| HP1436 | Predicted coding region HP1436 with no homologue in the databases | Putative uncharacterized protein |
| HP1437 | Predicted coding region HP1437 with no homologue in the databases | Putative signal transduction protein |
| HP1438 | Predicted lipoprotein | COLICIN E3 |
| HP1439 | Predicted coding region HP1439 with no homologue in the databases | Regulatory protein SIR4 |
| HP1439.1 | Predicted coding region HP1439.1 with no homologue in the databases | Importin alpha re-exporter |
| HP1440 | Predicted coding region HP1440 with no homologue in the databases | Glutamyl endopeptidase |
| HP1441 | Predicted cyclophilin-type peptidyl-prolyl cis-trans isomerase | Peptidyl-prolyl cis-trans isomerase a |
| HP1442 | Predicted carbon storage regulator | Carbon storage regulator homolog |
| HP1443 | Predicted 4-disphosphocytidyl-2-C-methyl-D-erythritol kinase involved in isoprenoid biosynthesis | 4-diphosphocytidyl-2c-methyl-d-erythritol kinase (transferase) |
| HP1444 | Predicted SsrA-binding protein involved in SsrA RNA (tmRNA) recognition of stalled mRNAs | SsrA-binding protein |
| HP1445 | Predicted biopolymer transport accessory protein | Biopolymer transport protein (exbb) |
| HP1446 | Predicted biopolymer transport accessory protein | Biopolymer transport exbd protein |
| HP1447 | Predicted ribosomal protein L34 | 23s rrna protein L34 |
| HP1448 | Predicted ribonuclease P protein component | Ribonuclease P |
| HP1449 | Predicted coding region HP1449 | NADH-quinone oxidoreductase chain 5 |
| HP1450 | Predicted inner membrane protein | Inner membrane protein oxaA |
| HP1451 | Predicted coding region HP1451 | Predicted RNA-binding protein |
| HP1452 | Predicted thiophene/furan oxidation protein | Probable tRNA modification GTPase trmE |
| HP1453 | Predicted outer membrane protein HomD | Outer membrane protein |
| HP1454 | Predicted coding region HP1454 with no homologue in the databases | TIP-alpha |
| HP1455 | Predicted coding region HP1455 with no homologue in the databases | Protein (CYTOCHROME C-553) |
| HP1456 | Membrane-associated lipoprotein | TIP-alpha |
| HP1457 | Predicted coding region HP1457 | Protein TOLB (involved in maintaining the integrity of the outer membrane) |
| HP1458 | Thioredoxin | Thioredoxin (TRXA-2) |
| HP1459 | Predicted pseudouridine synthase B involved in 23S rRNA base modifications | Ribosomal small subunit pseudouridine synthase |
| HP1460 | Predicted DNA polymerase III alpha subunit | DNA polymerase iii alpha subunit |
| HP1461 | Predicted cytochrome c551 peroxidase | Cytochrome c551 peroxidase |
| HP1462 | Predicted secreted protein | Uncharacterized protein |
| HP1463 | Predicted coding region HP1463 with no homologue in the databases | hypothetical protein |
| HP1464 | Predicted ABC transport system substrate binding protein | ABC transport system substrate-binding protein |
| HP1465 | Predicted ABC transporter ATP-binding protein | Amino acid ABC transporter |
| HP1466 | Predicted ABC transport system permease | DNA binding protein |
| HP1467 | Predicted outer membrane protein | |  | outer membrane protein family protein | | --- | --- | |
| HP1468 | Predicted branched-chain-amino-acid aminotransferase | Branched-chain amino acid aminotransferase |
| HP1469 | Outer membrane protein HorJ | outer membrane protein HorJ |
| HP1470 | Predicted DNA polymerase I | TAQ DNA polymerase |
| HP1471 | Non-functional type IIS sequence specific BcgI-like S-subunit in a silent state | type I restriction enzyme specificity protein |
| HP1472 | Non-functional type IIS sequence specific BcgI-like bifunctional restriction endonuclease-adenosine specific methylase alpha-subunit | Type I restriction-modification system methylation subunit |
| HP1473 | Predicted coding region HP1473 | Ribose-phosphate pyrophosphokinase |
| HP1474 | Predicted thymidylate kinase | Thymidylate kinase Transferase |
| HP1475 | Predicted lipopolysaccharide core biosynthesis protein | Phosphopantetheine adenylyltransferase |
| HP1476 | Predicted aromatic acid decarboxylase | Probable aromatic acid decarboxylase |
| HP1477 | Predicted coding region HP1477 | Flagella basal body P-ring formation protein |
| HP1478 | Predicted DNA helicase II involved in the the nucleotide excision repair pathway | DNA helicase |
| HP1479 | Predicted coding region HP1479 | Udp-n-acetylglucosamine—peptide n-acetylglucosaminyltransferase |
| HP1480 | Predicted seryl-tRNA synthetase | Seryl-tRNA synthetase (SerRS) class II core catalytic domain. |
| HP1481 | Predicted coding region HP1481 | Hydrolase, carbon-nitrogen family |
| HP1482 | Predicted small subunit of the single-stranded DNA-specific dexodeoxyribonuclease VII | exodeoxyribonuclease VII small subunit |
| HP1483 | Predicted methyltransferase involved in ubiquinone/menaquinone biosynthesis | Putative S-adenosyl-L-methionine-dependent Methyltransferase |
| HP1484 | Predicted integral membrane protein | JmjC domain-containing histone demethylation |
| HP1485 | Predicted coding region HP1485 | GTP-binding protein LEPA |
| HP1486 | Predicted coding region HP1486 | Putative abc type-2 transporter |
| HP1487 | Predicted coding region HP1487 | Putative abc type-2 transporter/ putative abc type-2 transporter |
| HP1488 | Predicted secreted protein | Macrolide-specific efflux protein |
| HP1489 | Predicted coding region HP1489 | Outer membrane protein tolc |
| HP1490 | Predicted integral membrane protein with a TlyC-like hemolysin domain | CBS domain-containing protein |
| HP1491 | Predicted phosphate permease | Gtp-binding nuclear protein |
| HP1492 | Predicted nifU-like protein | NifU-like protein HIRIP5 |
| HP1493 | Predicted coding region HP1493 | TPR-containing protein |
| HP1494 | Predicted UDP-MurNac-tripeptide synthetase | UDP-n-acetylmuramoyl-l-alanyl-d-glutamate—2,6-diaminopimelate ligase |
| HP1495 | Predicted transaldolase | Transaldolase |
| HP1496 | Predicted ribosomal protein L25 | 50s ribosomal protein l25 |
| HP1497 | Predicted peptidyl-tRNA hydrolase | Peptidyl-trna hydrolase |
| HP1498 | Predicted coding region HP1498 | Transforming growth factor beta regulator 1 |
| HP1499 | Predicted coding region HP1499 with no homologue in the databases | Protein (endonuclease) |
| HP1500 | Predicted coding region HP1500 with no homologue in the databases | 23aa |
| HP1501 | Outer membrane protein HorK | outer membrane protein HorK |
| HP1502 | Predicted coding region HP1502 | MG^2+^ transporter MGTE |
| HP1503 | Metal-transporting ATPase/P-type transporting ATPase involved in transition metal resistance | Plasma membrane atpase |
| HP1504 | Predicted coding region HP1504 | Putative methyltransferase |
| HP1505 | Predicted bifunctional Riboflavin-specific deaminase/5-amino-6-(5-phosphoribosylamino)uracil reductase | Diaminohydroxyphosphoribosylaminopyrimidine deami amino-6-(5-phosphoribosylamino)uracil reductase(Biosynthetic protein) |
| HP1506 | Predicted sodium/glutamate symport carrier protein | Transporter |
| HP1507 | Predicted coding region HP1507 | |  | Saccharopine dehydrogenase | | --- | --- |  |  | | --- | |
| HP1508 | Predicted ferredoxin-like protein | |  | FixG-related protein | | --- | --- | |
| HP1509 | Predicted integral membrane protein | Superoxide dismutase [Cu-Zn] |
| HP1510 | Predicted dihydroneopterin aldolase | 7,8-dihydroneopterin aldolase |
| HP1511 | Predicted FrpB-like protein | iron-regulated outer membrane protein FrpB4 |
| HP1512 | Predicted iron-regulated outer membrane protein | transferrin-binding protein a |
| HP1513 | Predicted selenocysteine synthase | Selenocysteine lyase |
| HP1514 | Predicted transcription termination factor A | N utilization substance protein A |
| HP1515 | Predicted coding region HP1515 with no homologue in the databases | outer membrane protein |
| HP1516 | Predicted coding region HP1516 with no homologue in the databases | Transcription regulator |
| HP1517 | Predicted type IIS restriction-modification protein | Adenine-N6-DNA-methyltransferase TAQI |
| HP1518 | Predicted coding region HP1518 | Three prime repair exonuclease 2 |
| HP1519 | Predicted coding region HP1519 | Holliday junction recognition protein |
| HP1520 | Predicted coding region HP1520 | Chromosome partition protein mukB, Linker |
| HP1521 | Predicted type III R-M system restriction enzyme | DNA repair protein RAD25 |
| HP1522 | Predicted type III R-M system modification enzyme | 627 aa |
| HP1523 | Predicted ATP-dependent DNA helicase | Helicase |
| HP1524 | Predicted coding region HP1524 with no homologue in the databases | Protein (phosphate system positive regulatory) |
| HP1525 | Predicted coding region HP1525 | outer membrane protein HorD |
| HP1526 | Predicted exodeoxyribonuclease | Exodeoxyribonuclease |
| HP1527 | comH, essential gene for natural transformation | Endo-1,4-beta-xylanase 3 |
| HP1528 | Predicted coding region HP1528 with no homologue in the databases | Sporulation inhibitor sda |
| HP1529 | Predicted chromosomal replication initiator protein | Chromosomal replication initiator protein dna |
| HP1530 | Predicted purine nucleoside phosphorylase | s-adenosylhomocysteine nucleosidase |
| HP1531 | Predicted coding region HP1531 with no homologue in the databases | |  | Hypothetical protein HP1531 | | --- | --- | |
| HP1532 | Predicted glucosamine fructose-6-phosphate aminotransferase | Glucosamine 6-phosphate synthase |
| HP1533 | Predicted coding region HP1533 | Thymidylate synthase THYX |
| HP1534 | Predicted IS605 transposase B | Endonuclease VIII |
| HP1535 | Predicted IS605 transposase A | Transposase |
| HP1536 | Predicted coding region HP1536 with no homologue in the databases | 18aa |
| HP1537 | Predicted coding region HP1537 with no homologue in the databases | Glycolipid-anchored surface protein 2 |
| HP1538 | Predicted ubiquinol cytochrome c oxidoreductase, cytochrome c subunit | cytochrome bc1 complex |
| HP1539 | Predicted ubiquinol cytochrome c oxidoreductase, cytochrome b subunit | cytochrome bc1 (Oxidoreductase) |
| HP1540 | Predicted ubiquinol cytochrome c oxidoreductase, 2Fe-2S subunit | Ubiquinol-cytochrome c reductase iron-sulfur |
| HP1541 | Predicted transcription-repair coupling factor | Transcription-repair coupling factor |
| HP1542 | Predicted coding region HP1542 | Regulatory protein |
| HP1543 | Predicted toxR-activated gene | Zinc peptidase |
| HP1544 | Predicted toxR-activated gene | Zinc peptidase |
| HP1545 | Predicted folylpolyglutamate synthase | Folylpolyglutamate synthase |
| HP1546 | Predicted coding region HP1546 | putative lipoprotein b   |  |  | | --- | --- | |
| HP1547 | Predicted leucyl-tRNA synthetase | leucyl-tRNA synthetase |
| HP1548 | Predicted integral membrane protein | Lysozyme |
| HP1549 | Predicted protein-export membrane protein | Probable secdf protein-export membrane protein. |
| HP1550 | Predicted protein-export membrane protein | Probable secdf protein-export membrane protein. |
| HP1551 | Predicted secreted protein | Membrane Protein/transport Protein |
| HP1552 | Predicted Na+/H+ antiporter | Na(+)/h(+) antiporter(membrane protein) |
| HP1553 | Predicted ATP-dependent nuclease | ATP-dependent helicase/nuclease subunit A |
| HP1554 | Predicted ribosomal protein S2 | 30s ribosomal protein s2 |
| HP1555 | Predicted translation elongation factor Ts | Elongation factor Ts |
| HP1556 | Predicted penicillin-binding protein 3 or FtsI involved in peptidoglycan synthesis and cell division | Penicillin-binding protein 3 |
| HP1557 | Predicted flagellar basal-body protein | flagellar hook-basal body protein FliE |
| HP1558 | Predicted flagellar basal-body rod protein (proximal rod protein) | | Flagellar hook protein flgE | | --- | |  | |
| HP1559 | Predicted flagellar basal-body rod protein (proximal rod protein) | | Flagellar hook  protein flgE | | --- | |  | |
| HP1560 | Predicted cell division protein | cell division protein   |  | | --- | |
| HP1561 | Predicted iron(III) ABC transporter, periplasmic iron-binding protein | iron(III) ABC transporter, periplasmic iron-binding protein |
| HP1562 | Predicted iron(III) ABC transporter, periplasmic iron-binding protein | Iron(iii) ABC transporter, periplasmic iron-bindi |
| HP1563 | Predicted alkyl hydroperoxide reductase | Alkyl hydroperoxide-reductase |
| HP1564 | Predicted outer membrane lipoprotein | Membrane lipoprotein tpn32 |
| HP1565 | Predicted penicillin-binding protein 2 | Penicillin-binding protein 2 |
| HP1566 | Predicted coding region HP1566 with no homologue in the databases | Ubiquitin-conjugating enzyme E2 H |
| HP1567 | Predicted GTPase involved in cell division and normal septation | GTP-binding protein |
| HP1568 | Predicted coding region HP1568 | Periplasmic lipopolysaccharide transport protein |
| HP1569 | Predicted coding region HP1569 with no homologue in the databases | Lipopolysaccharide export system protein lptC |
| HP1570 | Predicted ABC transporter system inner membrane protein | 3-deoxy-D-manno-octulosonate 8-phosphate phosphatase |
| HP1571 | Predicted rare lipoprotein A | |  | rare lipoprotein A | | --- | --- | |
| HP1572 | Predicted regulatory protein | |  | NADH-quinone oxidoreductase  subunit | | --- | --- | |
| HP1573 | Predicted DNAse of the TatD family | Putative TatD related DNAse |
| HP1574 | Predicted riboflavin synthase alpha subunit | Riboflavin synthase alpha chain |
| HP1575 | Predicted FlhB-related flagellar biosynthesis protein | |  | Flagellar biosynthetic protein flhB | | --- | --- | |
| HP1576 | ABC transporter system ATP-binding domain | D-methionine transport system permease protein |
| HP1577 | ABC transporter system permease protein | D-methionine transport system permease protein |
| HP1578 | Predicted LPS biosynthesis protein | LPS biosynthesis protein |
| HP1579 | Predicted coding region HP1579 with no homologue in the databases | Transcription |
| HP1580 | Predicted coding region HP1580 with no homologue in the databases | acid phosphatase |
| HP1581 | Predicted undecaprenyl phosphate N-acetylglucosaminyltransferase | Lipid G protein-Coupled Receptor |
| HP1582 | Predicted pyridoxal phosphate biosynthetic protein J | Pyridoxal phosphate biosynthetic protein |
| HP1583 | Predicted pyridoxal phosphate biosynthetic protein A | 4-hydroxythreonine-4-phosphate dehydrogenase 2 |
| HP1584 | Predicted O-sialoglycoprotein endopeptidase | O-sialoglycoprotein endopeptidase |
| HP1585 | Predicted flagellar basal-body rod protein | Flagellar hook protein |
| HP1586 | Predicted coding region HP1586 | Toxin |
| HP1587 | Predicted coding region HP1587 | 209 aa |
| HP1588 | Predicted coding region HP1588 | Lipid Binding Protein |
| HP1589 | Predicted coding region HP1589 | Toxin |
| HP1590 | Predicted coding region HP1590 with no homologue in the databases | 39 aa |
